# Comprehensive curation and analysis of global interaction networks in *Saccharomyces cerevisiae*

**DOI:** 10.1186/jbiol36

**Published:** 2006-06-08

**Authors:** Teresa Reguly, Ashton Breitkreutz, Lorrie Boucher, Bobby-Joe Breitkreutz, Gary C Hon, Chad L Myers, Ainslie Parsons, Helena Friesen, Rose Oughtred, Amy Tong, Chris Stark, Yuen Ho, David Botstein, Brenda Andrews, Charles Boone, Olga G Troyanskya, Trey Ideker, Kara Dolinski, Nizar N Batada, Mike Tyers

**Affiliations:** 1 Samuel Lunenfeld Research Institute, Mount Sinai Hospital, Toronto ON M5G 1X5, Canada; 2 Department of Medical Genetics and Microbiology, University of Toronto, Toronto ON M5S 1A8, Canada; 3 Department of Bioengineering, University of California San Diego, 9500 Gilman Drive, La Jolla, CA 92093-0412, USA; 4 Lewis-Sigler Institute for Integrative Genomics, Princeton University, Washington Road, Princeton, NJ 08544, USA; 5 Department of Computer Science, Princeton University, NJ 08544, USA; 6 Banting and Best Department of Medical Research, University of Toronto, Toronto ON M5G 1L6, Canada

## Abstract

**Background:**

The study of complex biological networks and prediction of gene function has been enabled by high-throughput (HTP) methods for detection of genetic and protein interactions. Sparse coverage in HTP datasets may, however, distort network properties and confound predictions. Although a vast number of well substantiated interactions are recorded in the scientific literature, these data have not yet been distilled into networks that enable system-level inference.

**Results:**

We describe here a comprehensive database of genetic and protein interactions, and associated experimental evidence, for the budding yeast *Saccharomyces cerevisiae*, as manually curated from over 31,793 abstracts and online publications. This literature-curated (LC) dataset contains 33,311 interactions, on the order of all extant HTP datasets combined. Surprisingly, HTP protein-interaction datasets currently achieve only around 14% coverage of the interactions in the literature. The LC network nevertheless shares attributes with HTP networks, including scale-free connectivity and correlations between interactions, abundance, localization, and expression. We find that essential genes or proteins are enriched for interactions with other essential genes or proteins, suggesting that the global network may be functionally unified. This interconnectivity is supported by a substantial overlap of protein and genetic interactions in the LC dataset. We show that the LC dataset considerably improves the predictive power of network-analysis approaches. The full LC dataset is available at the BioGRID () and SGD () databases.

**Conclusion:**

Comprehensive datasets of biological interactions derived from the primary literature provide critical benchmarks for HTP methods, augment functional prediction, and reveal system-level attributes of biological networks.

## Introduction

The molecular biology, biochemistry and genetics of the budding yeast *Saccharomyces cerevisiae *have been intensively studied for decades; it remains the best-understood eukaryote at the molecular genetic level. Completion of the *S. cerevisiae *genome sequence nearly a decade ago spawned a host of functional genomic tools for interrogation of gene and protein function, including DNA microarrays for global gene-expression profiling and location of DNA-binding factors, and a comprehensive set of gene deletion strains for phenotypic analysis [1,2]. In the post-genome sequence era, high-throughput (HTP) screening techniques aimed at identifying novel protein complexes and gene networks have begun to complement conventional biochemical and genetic approaches [3,4]. Systematic elucidation of protein interactions in *S. cerevisiae *has been carried out by the two-hybrid method, which detects pair-wise interactions [5-7], and by mass spectrometric (MS) analysis of purified protein complexes [8,9]. In parallel, the synthetic genetic array (SGA) and synthetic lethal analysis by microarray (dSLAM) methods have been used to systematically uncover synthetic lethal genetic interactions, in which non-lethal gene mutations combine to cause inviability [10-13]. In addition to HTP analyses of yeast protein-interaction networks, initial yeast two-hybrid maps have been generated for the nematode worm *Caenorhabditis elegans*, the fruit fly *Drosophila melanogaster *and, most recently, for humans [14-17]. The various datasets generated by these techniques have begun to unveil the global network that underlies cellular complexity.

The networks implicit in HTP datasets from yeast, and to a limited extent from other organisms, have been analyzed using graph theory. A primary attribute of biological interaction networks is a scale-free distribution of connections, as described by an apparent power-law formulation [18]. Most nodes – that is, genes or proteins – in biological networks are sparsely connected, whereas a few nodes, called hubs, are highly connected. This class of network is robust to the random disruption of individual nodes, but sensitive to an attack on specific highly connected hubs [19]. Whether this property has actually been selected for in biological networks or is a simple consequence of multilayered regulatory control is open to debate [20]. Biological networks also appear to exhibit small-world organization - namely, locally dense regions that are sparsely connected to other regions but with a short average path length [21-23]. Recurrent patterns of regulatory interactions, termed motifs, have also recently been discerned [24,25]. In conjunction with global profiles of gene expression, HTP datasets have been used in a variety of schemes to predict biological function for characterized and uncharacterized proteins [3,26-32]. These initial network approaches to system-level understanding hold considerable promise.

Despite these successes, all network analyses undertaken so far have relied exclusively on HTP datasets that are burdened with false-positive and false-negative interactions [33,34]. The inherent noise in these datasets has compromised attempts to build a comprehensive view of cellular architecture. For example, yeast two-hybrid datasets in general exhibit poor concordance [35]. The unreliability of such datasets, together with the still sparse coverage of known biological interaction space, clearly limit studies of biological networks, and may well bias conclusions obtained to date.

A vast resource of previously discovered physical and genetic interactions is recorded in the primary literature for many species, including yeast. In general, interactions reported in the literature are reliable: many have been verified by multiple experimental methods and/or more than one research group; most are based on methods of known sensitivity and reproducibility in well controlled experiments; most are reported in the context of supporting cell biological information; and all have been subjected to the scrutiny of peer review. But while publications on individual genes are readily accessed through public databases such as PubMed, the embedded interaction data have not been systematically compiled in a searchable relational database. The Yeast Proteome Database (YPD) represented the first systematic effort to compile protein-interaction and other data from the literature [36]; but although originally free of charge to academic users, YPD is now available only on a subscription basis. A number of important databases that curate protein and genetic interactions from the literature have been developed, including the Munich Information Center for Protein Sequences (MIPS) database [37], the Molecular Interactions (MINT) database [38], the IntAct database [39], the Database of Interacting Proteins (DIP) [40], the Biomolecular Interaction Network Database (BIND) [41], the Human Protein Reference Database (HPRD) [42], and the BioGRID database [43,44]. At present, however, interactions recorded in these databases represent only partial coverage of the primary literature. The efforts of these databases will be facilitated by a recently established consortium of interaction databases, termed the International Molecular Exchange Consortium (IMEx) [45], which aims both to implement a structured vocabulary to describe interaction data (the Protein Standards Initiative-Molecular Interaction, PSI-MI [46]) and to openly disseminate interaction records. A systematic international effort to codify gene function by the Gene Ontology (GO) Consortium also records protein and genetic interactions as functional evidence codes [47], which can therefore be used to infer interaction networks [48].

Despite the fact that many interactions are clearly documented in the literature, these data are not yet in a form that can be readily applied to network or system-level analysis. Manual curation of the literature specifically for gene and protein interactions poses a number of problems, including curation consistency, the myriad possible levels of annotation detail, and the sheer volume of text that must be distilled. Moreover, because structured vocabularies have not been implemented in biological publications, automated machine-learning methods are unable to reliably extract most interaction information from full-text sources [49]. Budding yeast represents an ideal test case for systematic literature curation, both because the genome is annotated to an unparalleled degree of accuracy and because a large fraction of genes are characterized [50]. Approximately 4,200 budding yeast open reading frames (ORFs) have been functionally interrogated by one means or another [51]. At the same time, because some 1,500 are currently classified by the GO term 'biological process unknown', a substantial number of gene functions remain to be assigned or inferred.

Here we report a literature-curated (LC) dataset of 33,311 protein and genetic interactions, representing 19,499 non-redundant interactions, from a total of 6,148 publications in the primary literature. The low overlap between the LC dataset and existing HTP datasets suggests that known physical and genetic interaction space may be far from saturating. Analysis of the network properties of the LC dataset supports some conclusions based on HTP data but refutes others. The systematic LC dataset improves prediction of gene function and provides a resource for future endeavors in network biology.

## Results

### Curation strategy

A search of the available online literature in PubMed yielded 53,117 publications as of November 1, 2005 that potentially contain interaction data on one or more budding yeast genes and/or proteins. A total of 5,434 of the 5,726 currently predicted proteins [52] are referred to at least once in the primary literature. All abstracts associated with yeast gene names or registered aliases were retrieved from PubMed and then examined by curators for evidence of interaction data. Where available, the full text of papers, including figures and tables, was read to capture all potential protein and genetic interactions. A curation database was constructed to house protein-protein, protein-RNA and gene-gene interactions associated with all known or predicted proteins in *S. cerevisiae*, analogous in structure to the BioGRID interaction database [43,53]. Each interaction was assigned a unique identifier that tracked the source, date of entry, and curator name. To expedite curation, we recorded the direct experimental evidence for interactions but not other potentially useful information such as strain background, mutant alleles, specific interaction domains or subcellular localization. Interactions reported in reviews or as unpublished data were not considered sufficiently validated. Protein-RNA and protein-DNA associations detected by genome-wide microarray methods were also not included in the dataset. Finally, we did not record interactions between *S. cerevisiae *genes/proteins and those of another species, even when such interactions were detected in yeast.

Abstracts were inspected with efficient web-based tools for candidate interaction data. Of the initial set of 53,117 abstracts, 21,324 were immediately designated as 'wrong organism', usually because of a direct reference to a yeast homolog or to a yeast two-hybrid screen carried out with a non-yeast bait (that is, the capturing protein) and library. This class of incorrect assignment is not easily recognized by text-mining algorithms but is readily discerned by curators. Of the remaining 31,793 yeast-specific abstracts, 9,145 were associated with accessible electronic versions of the full paper, which were then manually curated for protein and genetic interactions by directly examining data figures and tables.

We defined a minimal set of experimental method categories to describe the evidence for each recorded interaction (see Materials and methods for definitions). Physical interactions were divided into eight *in vivo *categories (affinity capture-mass spectrometry, affinity capture-western, affinity capture-RNA, co-fractionation, co-localization, co-purification, fluorescence resonance energy transfer (FRET), two-hybrid) and six *in vitro *categories (biochemical activity, co-crystal structure, far western, protein-peptide, protein-RNA, reconstituted complex). In each of these categories, except co-purification, the protein-interaction pair corresponded to that described in the experiment, typically as the bait and prey (that is, the capturing protein and the captured protein(s), respectively). For co-purification, in which a purified intact protein complex is isolated by conventional chromotography or other means, a virtual bait was assigned (see Material and methods). A final biochemical interaction category, called co-purification, was used to indicate a purified intact protein complex isolated by conventional chromatography or other means. Genetic interactions were divided into eight categories (dosage growth defect, dosage lethality, dosage rescue, phenotypic enhancement, phenotypic suppression, synthetic growth defect, synthetic lethality, synthetic rescue). Genetic interactions with RNA-encoding ORFs were not scored separately from protein-coding genes. In rare instances in which an interaction could not be readily assigned a protein or genetic interaction category, the closest substitute was chosen and an explanation of the exact experimental context was noted in a free-text qualification box.

### Curated datasets

Two protein-interaction (PI) datasets were constructed as follows. Five extant HTP protein-interaction studies [5-9], which are often used in network analysis, were combined into a dataset termed HTP-PI that contained 11,571 non-redundant interactions. All other literature-derived protein interactions formed a dataset termed LC-PI that contained 11,334 nonredundant interactions. The combined LC-PI and HTP-PI datasets contain 21,281 unique interactions (Table 1). The 428 discrete protein-RNA interactions recorded in the curation effort were not included in the LC-PI dataset, and were not analyzed further. Although a number of recent publications reported protein interactions that might have been classified as HTP-like, it was not possible to rigorously separate intertwined data types in these publications, and so by default we added all such interactions to the LC-PI dataset (see below).

Two genetic interaction (GI) datasets were constructed as follows. All data derived from systematic SGA and dSLAM approaches were grouped into a single dataset termed HTP-GI that contained 6,103 nonredundant interactions. This designation was possible because each SGA or dSLAM screen is carried out on a genome-wide scale using the same set of deletion strains [10,12,13]. We note that most SGA and dSLAM genetic interactions reported to date have been independently validated by either tetrad or random spore analysis. All other genetic interactions determined by conventional means were combined to form a dataset termed LC-GI dataset that contained 8,165 nonredundant interactions. The combined LC-GI and HTP-GI datasets contain 13,963 unique interactions (Table 1).

**Figure 1:**
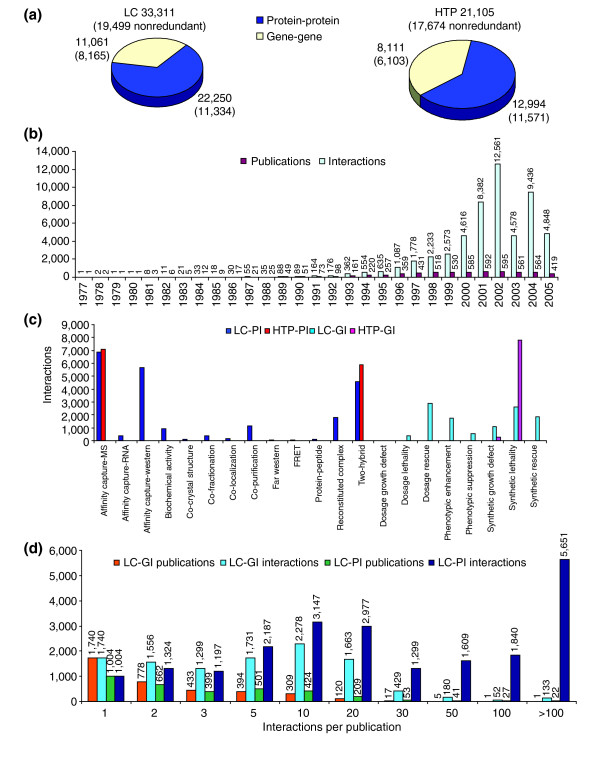
Characterization of the LC interaction dataset. **(a) **The total number of interactions in the LC dataset (left) and standard HTP datasets (right). Protein-protein interactions, blue; gene-gene interactions, yellow. **(b) **The number of publications that contain interaction data (red) and the number of interactions reported per year (light blue). **(c) **The number of interactions annotated for each experimental method. In this panel and all subsequent figures, each dataset is color coded as follows: LC-PI, blue; HTP-PI, red; LC-GI, aquamarine; HTP-GI, pink. **(d) **Number of interactions per publication in LC-GI and LC-PI datasets. Publications were binned by the number of interactions reported. The total number of papers and interactions in each bin is shown above each bar.

The analyses reported below were performed on the 1 November, 2005 versions of the LC-PI, HTP-PI, LC-GI, and HTP-GI datasets, which are summarized in Figure 1 and Table 1 (see Additional data file 1 for a full description of the datasets). For all analyses, the datasets were rendered as a spoke model network, in which the network corresponds directly to the minimal set of binary interactions defined by the raw data, as opposed to an exhaustive matrix model representation, in which all possible pair-wise combinations of interactions are inferred [34].

### Curation fidelity

To benchmark our curation effort, we assessed the overlap between the LC interaction dataset and interactions housed in the MIPS, BIND, and DIP databases [37,40,41]. Interactions attributed to 1,773 publications that were shared between at least one of these databases and the LC dataset were reinvestigated in detail. Depending on the particular comparison dataset, the false-negative rate for the LC dataset ranged from 5% to 20%, whereas the false-negative rates for other datasets varied from 36% to 50% (see Additional data files 2 and 3). To estimate our curation fidelity more precisely, 4,111 LC interactions between 1,203 nodes in a recently defined network termed the filtered yeast interactome (FYI) [54] were re-examined interaction-by-interaction and found to contain curation errors at an overall rate of around 4% (see Additional data file 3). All errors and missing interactions detected in these comparative analyses were corrected in the final dataset. Discordances between the different datasets underscore the need for parallel curation efforts in order to maximize curation coverage and accuracy.

### Overview of the LC dataset

The final LC dataset contains 33,311 physical and genetic interactions, representing 19,499 nonredundant entries derived from 6,148 different publications. The total size of the LC dataset exceeds that of all combined HTP datasets published before 1 November, 2005 (Figure 1a). The rate of growth of publications that document interactions in budding yeast has seemingly reached a plateau of about 600 publications per year, while the total number of interactions documented per year has on average continued to increase (Figure 1b). Protein interactions were supported mainly by three experimental methods: affinity capture with mass spectrometric detection, affinity capture with western blot detection, and two-hybrid assays (Figure 1c). In addition, 258 protein complexes were biochemically purified, minimally representing 1,104 interactions (see Additional data file 1 for a list of purified complexes). More arduous techniques such as FRET and structure determination of protein complexes accounted for far fewer interactions. Genetic interactions were documented by a spectrum of techniques, with some propensity towards synthetic lethal and dosage rescue interactions (Figure 1c). The numbers of interactions in each experimental method category are listed in Additional data file 1.

The distinction between HTP surveys and meticulous focused studies cannot be made by a simple cutoff in the number of interactions. Genetic interactions are usually robust, so the distinction by interaction number is less critical. Protein interactions on the other hand are inherently more variable, and as a consequence are usually validated by well controlled experiments in most focused studies. Approximately 50% of the LC-PI dataset derives from recent publications that report 50 or more protein interactions (Figure 1d). In many of these publications, interactions are interrogated via multiple bait proteins, typically by mass spectrometric or two-hybrid analysis. While not all of these interactions are individually validated in replicate experiments, in most cases there is sufficient experimental signal (for example, peptide coverage by mass spectrometry or different interacting fragments by two-hybrid) and overlap between different experiments that reasonable confidence is warranted. We designated these publications as systematic interrogation (SI) to indicate that most interactions are verified and of reasonable confidence. Five other publications designated as HTP surveys (HS) reported single broad screens that contained a total of 870 interactions, including interactions inferred from covalent modifications such as phosphorylation and conjugation of ubiquitin-like modifiers (ULMs). Systematic interrogation and HTP survey data were included in the LC-PI dataset for the purposes of network analysis below. For future applications of the dataset, publications that contain SI or HS interactions, as well as any posttranslational modifications associated with interactions, are listed in Additional data file 1. Because all interactions are documented both by PubMed identifiers and by a structured vocabulary of experimental evidence, these potentially less well substantiated interactions or data types can be readily removed from the dataset if desired.

### Replication and bias of interactions

**Figure 2:**
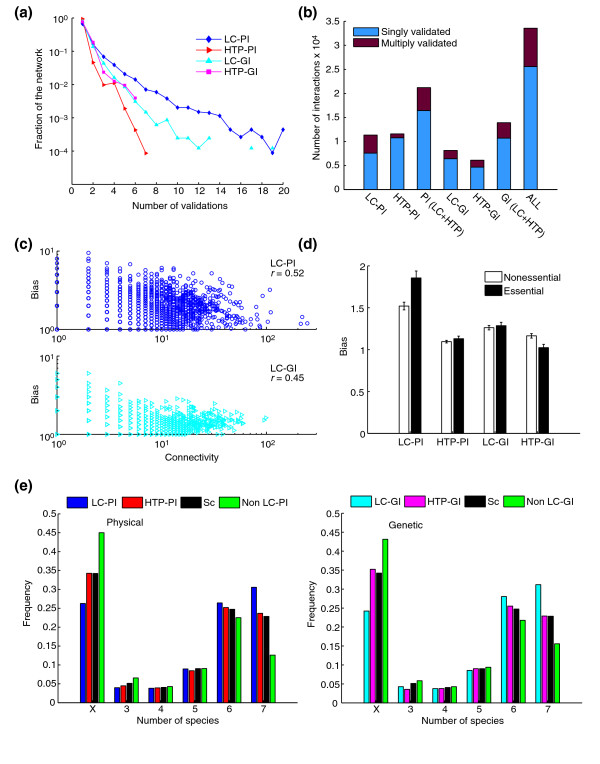
Validation of interactions within interaction datasets. **(a) **The fraction of interactions in each dataset supported by multiple validations (that is, different publications or types of experimental evidence). **(b) **The fraction of interactions in each indicated dataset supported by more than one publication or type of experimental evidence. **(c) **Better studied proteins or genes, as defined by the number of supporting publications relative to node connectivity (designated bias, see Materials and methods), tend to be more highly connected within the physical or genetic networks. **(d) **The study bias towards essential genes in each dataset. **(e) **The distribution of conserved proteins in interaction datasets. Frequency refers to fraction of the dataset in each bin. Orthologous eukaryotic clusters for seven standard species (*Arabidopsis thaliana*, *Caenorhabditis elegans*, *Drosophila melanogaster*, *Homo sapiens*, *Saccharomyces cerevisiae*, *Schizosaccharomyces pombe*, and *Encephalitozoon cuniculi*) were obtained from the COG database [96]. Sc refers to all budding yeast proteins as a reference dataset; non-LC refers to all HTP interactions except those that overlap with the LC datasets; X refers to yeast genes that were not assigned to any of the COG clusters and contains yeast-specific genes in addition to genes that have orthologs in only one of the other six species.

As all types of experimental evidence for each interaction were culled from each publication, it was possible to estimate the extent to which interactions in each dataset were overtly validated, either by more than one experimental method and/or by multiple publications. Even in the LC-PI and LC-GI datasets, most interactions were directly documented only once, with 33% and 20% of interactions in each respective dataset being reproduced by at least two publications or experimental methods (Figure 2a,b). Only a small fraction of any dataset was validated more than once (Figure 2a). These estimates of re-coverage are inherently conservative because of the minimal spoke representation used for each complex. Of particular importance, interactions that are well established in an initial publication are unlikely to be directly repeated by subsequent publications that build on the same line of enquiry.

It has been noted that persistently cited genes are not more connected than average, based on HTP networks [55]. To reveal potential bias in the extent of investigation of any given node in the LC datasets, we determined the number of total interactions (that is, including redundant interactions) in excess of connectivity for each node (see Materials and methods). Within the LC-PI and LC-GI datasets, it is evident that the more a protein or gene is studied, the more connections it is likely to exhibit (Figure 2c). A modest study bias of 23% towards essential genes was evident in the LC-PI dataset (Figure 2d). Whether these effects are due to increased coverage upon further study or the tendency of highly connected proteins to be studied in more detail is unclear.

Finally, we determined the extent to which evolutionarily conserved proteins are studied in each dataset. Each dataset was binned according to conservation of yeast proteins across seven species using the Clusters of Orthologous Groups (COG) database [56]. The HTP datasets were enriched towards nonconserved proteins, whereas the LC datasets were enriched for proteins conserved across the seven eukaryotic test species (Figure 2e). This bias probably reflects the tendency to study conserved proteins, which are more likely to be essential [57,58].

### GO coverage and coherence

**Figure 3:**
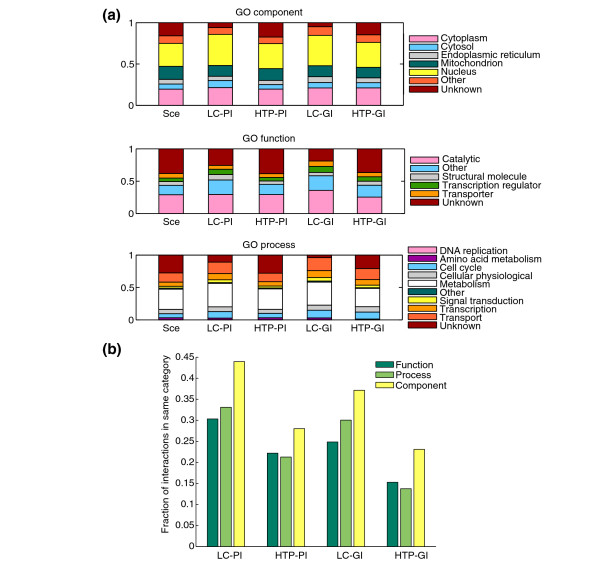
Distribution of GO terms for genes or proteins involved in genetic and physical interactions compared with genome-wide distribution. **(a) **Distribution of indicated GO cellular component, molecular function and biological process terms for nodes in each dataset. Sce refers to the distribution for all genes or proteins. **(b) **Fraction of interactions that share common GO terms in each of the three GO categories. High-level GO annotations (GO-Slim) were obtained from the SGD. The mean shared annotation is significantly higher for LC-PI than for HTP-PI for each of the three categories (Fisher's exact test, *P *< 1 × 10^-10^).

To determine how closely protein and genetic interaction pairs match existing GO descriptors of gene or protein function, we assessed high-level GO terms represented within different interaction datasets. The distribution of GO component, GO function and GO process categories for each dataset was determined and compared with the total distribution for all yeast genes (Figure 3a). Given that the GO annotation for *S. cerevisiae *is derived from the primary literature [47], it was not surprising that the LC-PI and LC-GI datasets showed a similar distribution across GO categories and terms, including under-representation for the term 'unknown' in each of the three GO categories. In contrast, the HTP-PI and HTP-GI datasets contained more genes designated as 'unknown', and a corresponding depletion in known categories. Certain specific GO categories were favored in the LC datasets, accompanied by concordance in the rank order of GO function or process terms between the LC-PI and LC-GI datasets, probably because of inherent bias in the literature towards subfields of biology (see also Additional data file 3).

To assess the coherence of each interaction dataset, we then determined the fraction of interactions that contained the same high level GO terms for each interaction partner across each of the GO categories (Figure 3b). By this criterion, the LC datasets were more coherent than the HTP datasets. This result reflects the higher false-positive rates in the HTP datasets, the higher incidence of uncharacterized genes in HTP datasets and also the potential for genome-wide approaches to identify new connections between previously unrelated pathways.

### Size estimate of the global protein-interaction network

On the basis of analysis of both two-hybrid HTP datasets and combined HTP and MIPS datasets, it has been estimated that there are on average five interaction partners per protein in the yeast proteome, and that by extrapolation the entire proteome contains 16,000–26,000 interactions [59]. Similar estimates of 20,000–30,000 interactions have been obtained by scaling the power-law connectivity distribution of an integrated data set of HTP interactions [34] and by the overlap of the HTP and MIPS datasets [33]. To reassess these estimates based on our LC-PI dataset, we began with the observation that the current LC-PI network contains roughly half of all predicted yeast proteins. We partitioned nodes into two sets, namely those nodes present in the LC-PI network (called S = seen, S × S defines the LC-PI dataset) and those nodes absent from the LC-PI network (called U = unseen). As U is about the same size as S, if the density of U × U is no more than that of S × S, then U × U will at most contain around 10,000 interactions. Similarly, because U × S is twice the size of U × U or S × S, it will contain 20,000 interactions. The sum total of all interactions predicted from LC-PI is thus 40,000. This estimate is subject to two countervailing reservations: the density of U × U may in fact be lower than for S regions (see below), while conversely, the current density of S × S may be an underestimate. The observations that well studied proteins are more highly connected and that the HTP-PI datasets undoubtedly contain *bona fide *interactions not present in S × S suggest that the density of S will certainly increase with further investigation. Extrapolations based on either mean node degree or degree distribution of LC-PI yielded values in the range of 21,000 to 40,000 interactions, again assuming that the density of S × S is saturating (data not shown).

### Coverage in HTP datasets

**Figure 4:**
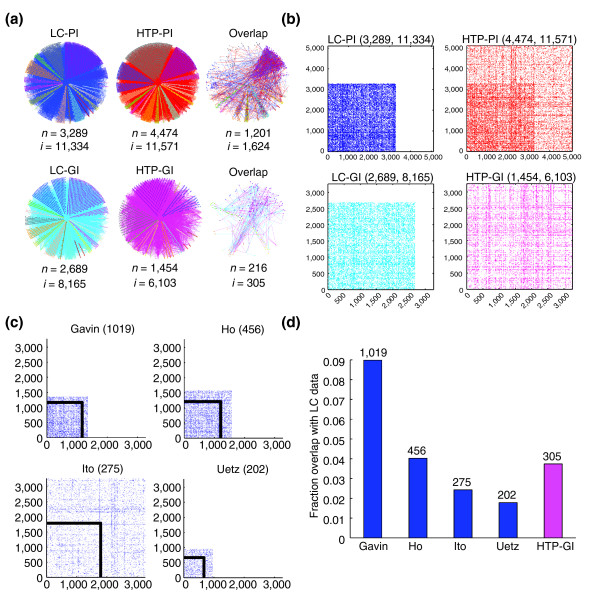
Intersection of LC and HTP datasets. **(a) **Datasets were rendered with the Osprey visualization system [65] to show overlap between indicated LC and HTP datasets. *n*, number of nodes; *i*, number of interactions. (**b) **Coverage in the HTP physical interaction dataset (collated from five major HTP studies: Uetz *et al*. [5], Ito *et al*. [6], Ito *et al*. [7], Gavin *et al*. [9], Ho *et al*. [8]) overlaps strongly with coverage in the LC dataset. Proteins present only in the LC dataset were labeled first, followed by proteins present only in the individual HTP datasets. In all plots, a dot represents interaction between proteins on the *x*- and *y*-axes. As the networks are undirected, plots are symmetric about the *x *= *y *line. Self interactions were removed. **(c) **Overlap of individual HTP datasets with the LC dataset. Dot plots show all interactions from each HTP dataset partitioned according to proteins that are present in the LC-PI dataset (inside the boxed region) and those that are not (outside the boxed region). 'Ito' indicates data from Ito *et al*. [7] rather than Ito *et al*. [7]. The protein content is different for each dataset and so ordinates are not superimposable. The number of overlapping interactions between each HTP dataset and the LC dataset is shown in parentheses. Note that only a small fraction of interactions in each boxed region actually overlaps with the LC-PI dataset because of the high false-negative rate in HTP data. **(d) **The number of LC interactions in HTP datasets.

A primary purpose of compiling the LC dataset was to provide a benchmark for HTP interaction studies. When each dataset is represented as a minimal spoke network model [34], the LC-PI network is of roughly the same size as the HTP-PI network, yet overlap between the two is only 14% (Figure 4a). To visualize the relative coverage of each dataset, dot-matrix representations of all pairwise interactions in each of the LC and HTP datasets were created and overlaid on the same ordinates. As expected, each dataset contains its own unique set of interactions (see Additional data file 3). To assess the relative distribution of interactions in the LC-PI versus HTP-PI datasets, full dot plots for each were compared, ordered first by proteins in the LC dataset then by proteins in the HTP dataset (Figure 4b). Interactions in the LC-PI dataset were uniform with respect to protein labels; that is, as expected there are no obvious areas of higher or lower interaction density across the approximately 3,000 proteins in the dataset. In the HTP-PI protein dataset, however, which contains interactions between 4,478 proteins, there were two distinct regions of interaction density: a high-density region that corresponded precisely to proteins defined in the LC-PI dataset (7.3 interactions per protein in LC-PI) and a low-density region that corresponded to interactions between proteins not in the LC-PI dataset (2.8 interactions per protein in HTP-PI). This indicates that there is a strong bias in interactions detected by HTP techniques. Analysis of each individual HTP-PI dataset revealed that bias towards previously studied proteins is inherent in the Gavin *et al*. [9], Ho *et al*. [8] and Uetz *et al*. [5] datasets (Figure 4c).

To examine the false-negative rate in HTP-PI datasets, we directly compared the LC-PI dataset to four extant HTP-PI datasets, two from large-scale two-hybrid analysis [5,7] and two from large-scale mass spectrometric identification of affinity-purified protein complexes [8,9]. Two-hybrid datasets tend to have a high rate of false-positive hits [33-35]; consistently, only 2–3% of interactions reported in two-hybrid screens have been substantiated elsewhere in the literature to date (Figure 4d). Because affinity-purification methods directly capture interaction partners in a physiological context, HTP mass spectrometric datasets fared somewhat better: around 9% of the 3,402 interactions reported by Gavin *et al*. [9] and around 4% of the 3,683 interactions reported by Ho *et al*. [8] have been documented elsewhere in the literature (Figure 4d).

Given that the HTP mass spectrometric studies were initiated with largely nonoverlapping sets of baits that represented only around 10% of the yeast proteome [8,9], we also assessed the extent to which these datasets captured known interactions for successful bait proteins. By this criterion, the Gavin datasets recapitulated around 30% of literature interactions, while the Ho dataset recapitulated around 20% of literature interactions. It was not possible to compare overall success rates for all HTP datasets because unsuccessful baits were not unambiguously identified in three of the studies [5,7,9]. We note that simple benchmark comparisons of HTP datasets may be confounded by bias in each dataset. For example, the average clustering coefficient in the LC-PI network was significantly higher for the set of baits used in the Gavin versus the Ho datasets (0.43 versus 0.39, *P *= 0.01) and so a higher rate of recovery is expected in the former.

The overlap between the LC-GI and HTP-GI datasets was also minimal at 305 interactions, or less than 5% of either dataset (Figure 4a,d). In part, this minimal overlap was due to the different nature of query genes in each dataset. In the primary literature, genetic interactions have traditionally been sought with conditional alleles of essential genes, whereas most HTP screens to date have used nonessential genes to query the haploid genome-wide deletion set, which by definition lacks all essential genes [10,12,13]. Consistently, essential nodes account for less than 6% of the overlap dataset (see Additional data file 1). In addition, because the HTP-GI dataset is composed almost entirely of synthetic lethal interactions (see Additional data file 1), whereas the LC-GI dataset contains all types of genetic interactions, the potential for overlap is further minimized. Indeed, about 80% of the overlap was accounted for by LC-GI synthetic lethal interactions (see Additional data file 1). As synthetic lethal interaction space is estimated at 200,000 interactions [12,60], both the LC-GI and HTP-GI datasets still only sparsely sample the global network.

Finally, various methods have been used to combine and refine HTP data. These methods substantially improved overlap with literature-derived interactions. For example, of about 2,500 interactions in a high-confidence distillation of HTP datasets, termed the FYI dataset [54], 60% were present in the LC-PI the dataset, while of the 2,455 interactions in another high-confidence dataset [33], 32% were present in the LC-PI dataset. While combined datasets ameliorate the problem of false-positive interactions, such combinations are by definition still prone to false-negative interactions.

### Degree distribution of the LC network

**Figure 5:**
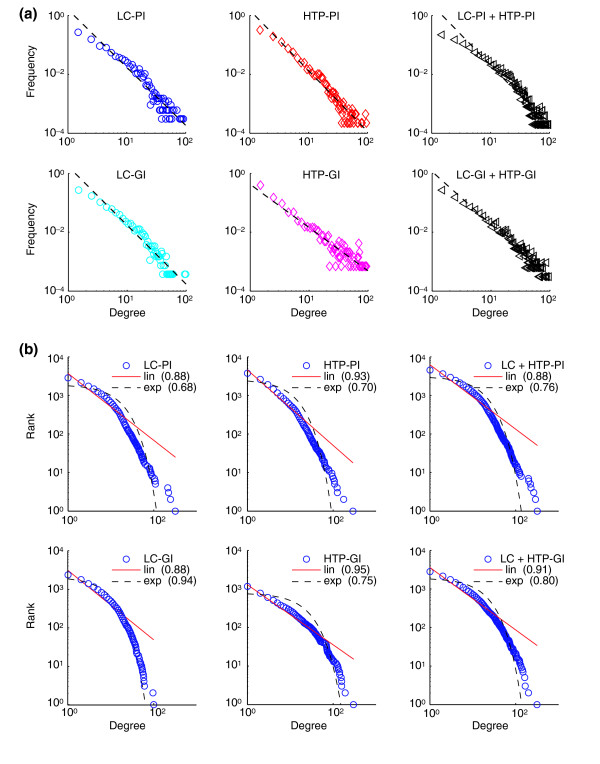
Scale-free degree distribution of physical and genetic interaction networks. **(a) **Frequency-degree plots of LC, HTP and combined networks. Degree is the connectivity (*k*) for each node, and frequency indicates the probability of finding a node with a given degree. The linear fit for each plot approximates a power-law distribution. **(b) **Rank-degree plots of LC, HTP, and combined networks. Each data point actually represents many nodes that have the same degree. The fit of the data to either linear (lin) or exponential (exp) curves is indicated for each plot and the coefficient of determination (R^2^) is reported in parentheses for each curve fit. Note that although the tail of each distribution exhibits a large deviation, only a small portion of the network is represented by the highly connected nodes in the tail region. For example, approximately 2% of nodes in the LC-PI and HTP-PI networks have connectivity greater than 30.

In a scale-free network, some nodes are highly connected whereas most nodes have few connections. Such networks follow an apparent power-law distribution that may arise as a consequence of preferential attachment of new nodes to well connected hubs, which are critical for the stability of the overall network [18,19,21-23]. Connectivity influences the way a network operates, including how it responds to catastrophic events, such as ablation of gene or protein function. Previous analysis of the yeast HTP protein-interaction dataset suggested that the overall network behaves in a scale-free manner [22,23]. Both the LC-PI and the HTP-PI datasets essentially followed a scale-free degree distribution, either alone or in combination (Figure 5a). We note, however, that the frequency-degree log plots did not yield a perfectly linear fit for the LC network, which showed a higher-than-expected concentration of nodes with connectivity of 10–12. If analysis of the LC network was restricted to nodes with connectivity less than 20 (which represent more than 95% of the data), then the log-linear fit was much better. Similarly, both the LC-GI and HTP-GI genetic networks, either alone or in combination, followed an apparent power-law distribution (Figure 5a), as shown previously for a HTP-GI network [12].

It has been argued recently that the power-law distribution observed for some biological networks is an effect of frequency-degree plots and not an intrinsic network property [61]. To assess this possibility, we reanalyzed each network as a rank-degree plot and determined goodness of fit for both linear and exponential curves. In all cases except LC-GI, a linear fit was better than an exponential fit, as judged by the coefficient of determination (Figure 5b). Even for the LC-GI network, a linear fit was nearly as good as an exponential fit. By the more stringent rank-degree plot criterion, we thus conclude that the LC and HTP networks obey a power-law distribution. Finally, it has also recently been noted that essential nodes form an exponential distribution in a HTP protein-interaction network [62]. We consistently find that the essential subnetwork of the LC-PI dataset is best fitted by an exponential distribution, whereas the residual nonessential network follows a power law (N.N.B., unpublished data).

### Essentiality, connectivity, and local density

**Figure 6:**
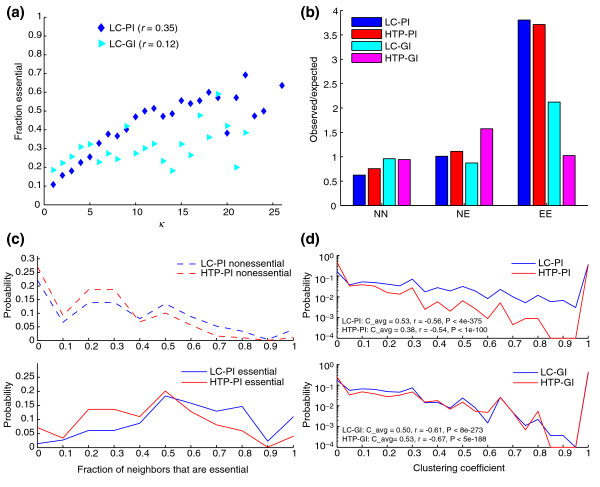
Connectivity of essential nodes. **(a) **Essential nodes tend to be more highly connected in the LC-PI and LC-GI networks. *k *is the measure of connectivity. **(b) **Essential-essential interactions are significantly enriched in the LC-PI and HTP-PI datasets but to a lesser extent in the LC-GI dataset. NN, nonessential-nonessential pairs; NE, nonessential-essential pairs, EE, essential-essential pairs. **(c) **The fraction of neighbors that are essential for LC-PI and HTP-PI networks. Only those nodes with connectivity greater than 3 were considered (*n *= 1,473 for LC-PI and *n *= 1,627 for HTP-PI). Compared with HTP-PI, a larger fraction of the immediate neighborhood of essential proteins in the LC-PI is composed of essential genes. **(d) **Clustering coefficient distribution for physical networks (top panel) and genetic networks (bottom panel). Average clustering coefficients and correlation coefficients were respectively: 0.53 and -0.56 for LC-PI, 0.38 and -0.54 for HTP-PI, 0.50 and -0.61 for LC-GI, 0.53 and -0.67 for HTP-GI. All correlations were computed using Spearman rank correlation and were statistically significant at *P *< 1e-100.

Random removal of nodes in HTP two-hybrid interaction networks does not affect the overall topology of the network, whereas deletion of highly connected nodes tends to break the network into many smaller components [22]. The likelihood that deletion of a given gene is lethal correlates with the number of interaction partners associated with it in the network. Thus, highly connected proteins with a central role in network architecture are three times more likely to be essential than are proteins with only a small number of links to other proteins. The LC-PI dataset exhibited a strong positive correlation between connectivity and essentiality, whereas the LC-GI dataset exhibited a modest positive correlation (*r *= 0.35, *P *< 1 × 10^-91 ^and *r *= 0.11, *P *< 1 × 10^-7^, respectively; Figure 6a). Indeed, in the LC-PI dataset, essential proteins had twice as many interactions on average than nonessential proteins (<*k*> = 11.7 and 5.2, respectively, *P *< 1 × 10^-100^, Mann-Whitney U test). This analysis buttresses the inference that highly connected genes are more likely to be essential [19]. Although it has been suggested that the essentiality is caused by connectivity [22], this notion seems unlikely because 44% of the proteins in the LC-PI dataset that were highly connected (*k *> 10) were nonessentials. We note that the definition of essentiality as narrowly defined by growth under optimal nutrient conditions is open to interpretation. Indeed, if the definition of essentiality is broadened to include inviability under more stressful conditions [2], the correlation with connectivity is substantially weaker, although still statistically significant (N.N.B., unpublished data).

The propensity of essential proteins to connect more frequently than nonessential proteins prompted us to reexamine the issue of essential-essential connections. From the analysis of HTP datasets, it has previously been reported that interactions between highly connected proteins appear to be suppressed [63]. In both the LC-PI and HTP-PI datasets, however, there was in fact a fourfold enrichment for essential-essential interactions (Figure 6b). The neighborhoods of essential proteins in both networks were significantly enriched in essential proteins when compared with the neighborhoods of nonessential proteins (for essentials <LC-PI> = 0.64 and <HTP-PI> = 0.48; for nonessentials <LC-PI> = 0.36 and <HTP-PI> = 0.27; *P *< 0.01 in each case). This effect has also recently been adduced from HTP data [62]. The LC-PI network exhibited a higher local density of essential interactions than the HTP-PI network as the fraction of essential neighbors in LC-PI was 35% greater than in HTP-PI and the fraction of essential proteins that were surrounded by only essential proteins in LC-PI was twice that in HTP-PI (Figure 6c). Significantly, comparison of an LC-PI subnetwork constructed of only essential proteins to an LC-PI subnetwork of nonessential proteins revealed that the former was fourfold more dense, more fully connected (91% versus 74% of nodes in the largest component), and more tightly connected (average clustering coefficient of 0.5 versus 0.3, see below). These essential-essential interactions were likely to be of functional relevance because the LC-GI dataset exhibited twice as many essential-essential interactions as expected (Figure 6b).

A primary attribute of each node is its clustering coefficient, which is a measure of local interaction density, defined as the percentage of node neighbors that also interact with each other. A clustering coefficient near 0 occurs when almost none of the neighbors is connected to each other, whereas a clustering coefficient near 1 occurs when many neighbors are connected to each other. Accordingly, proteins that are part of a multiprotein complex should have a high clustering coefficient. For all values of clustering coefficient (except 0), the mean clustering coefficient for the LC-PI network was greater than that of the HTP-PI network, often by more than one order of magnitude (Figure 6d, top). The mean clustering coefficient of the LC-PI network was 34% larger in magnitude than for the HTP-PI network. Ignoring the trivial case for nodes of degree 1, which by definition have the maximal clustering coefficient of 1 (that is, 26% of all nodes in LC-PI and 32% of all nodes in HTP-PI), 8% of all LC-PI nodes with degree higher than 2 were fully connected (that is, clustering coefficient of 1), compared with only 2% of all HTP-PI nodes. In contrast, the distributions of clustering coefficients for the LC-GI and HTP-GI networks were very similar, as was the average clustering coefficient (Figure 6d, bottom). For all four networks, the clustering coefficients were negatively correlated with connectivity, suggesting that locally dense interactions may limit the overall number of interaction partners that can access nodes within these regions.

### Overlap between protein and genetic networks

**Figure 7:**
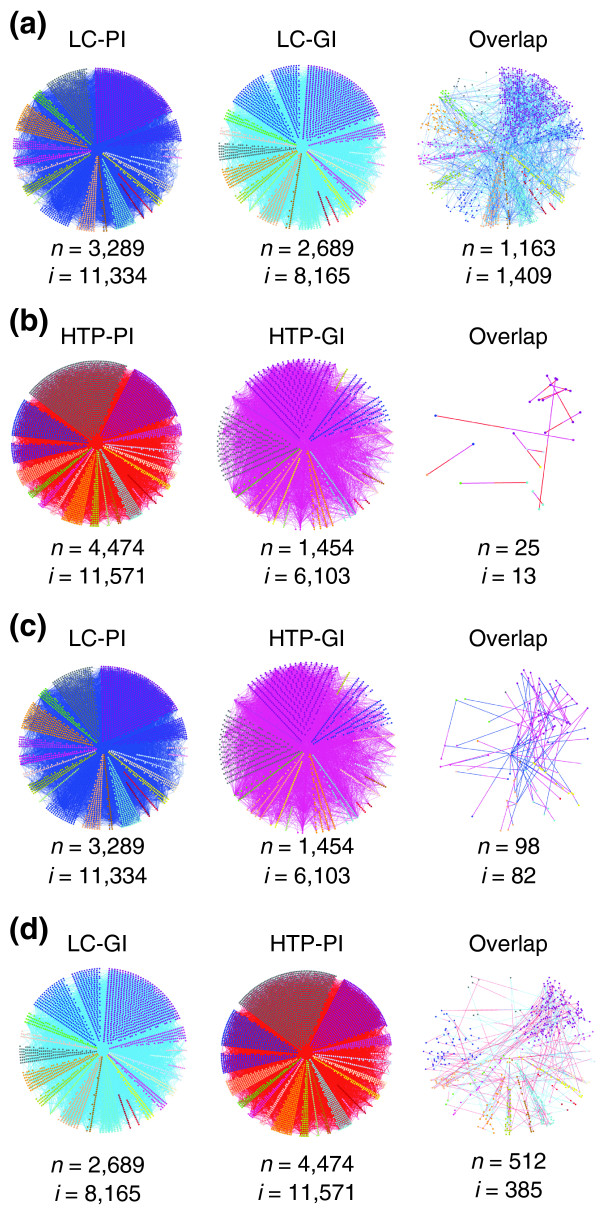
Overlap of physical and genetic interaction pairs. **(a) **Overlap between LC-PI and LC-GI datasets. **(b) **Overlap between HTP-PI and HTP-GI datasets. **(c) **Overlap between LC-PI and HTP-GI datasets. **(d) **Overlap between LC-GI and HTP-PI datasets.

Protein interactions by definition represent connections within complexes or along pathways, whereas genetic interactions typically represent functional connections of one sort or another between pathways [4,12,64]. We used the Osprey visualization tool [65] to represent and overlay protein- and genetic-interaction networks for the LC and HTP datasets. Given the perceived orthogonality of physical and genetic interaction space based on HTP studies [12], the LC-PI and LC-GI networks exhibited an unexpectedly high degree of overlap, at 12% of all protein interactions and 17% of all genetic interactions (Figure 7a). Of the 1,409 overlap pairs, 442 corresponded to interactions between essential proteins, while an additional 488 corresponded to interactions between an essential and a nonessential protein. The essential gene or protein content of the overlapping set of interactions was not substantially different from the input LC-PI and LC-GI datasets, nor was there pronounced enrichment or depletion for synthetic lethality or any other type of genetic interaction in the overlap dataset (see Additional data file 1). In striking contrast, overlap between the HTP-PI and HTP-GI networks was virtually nonexistent (Figure 7b), as has been previously noted [12]. This minimal overlap was due to the properties of the HTP-GI network, as the HTP-GI overlap with LC-PI was also minimal (Figure 7c), whereas the overlap between HTP-PI and LC-GI was significant (Figure 7d). Because essential genes were not enriched in the LC-PI/LC-GI overlap set, the under-representation of essential genes in the HTP-GI network [10,12,13] cannot explain the minimal overlap of HTP-GI with the LC-PI and HTP-PI networks (Figure 7b,c). It has been noted that proteins that exhibit more physical interactions tend also to exhibit more genetic interactions [66]. Indeed, the average number of physical connections for the nodes in the LC-PI/LC-GI overlap set was 7.7, compared with 3.2 for the remainder of the nodes in LC-PI. This feature does not, however, explain the discrepancy between the LC-GI and HTP-GI datasets because both had very similar physical connectivity distributions. Interestingly, half (706 of 1,409) of the interactions that do overlap in the LC-PI and LC-GI datasets mapped back to the same publication as each other, suggesting that investigators may often test specific interactions in order to support initial observations. This bias may help drive overlap between the LC-PI and LC-GI datasets.

### Correlations with protein abundance, localization, and expression

**Figure 8:**
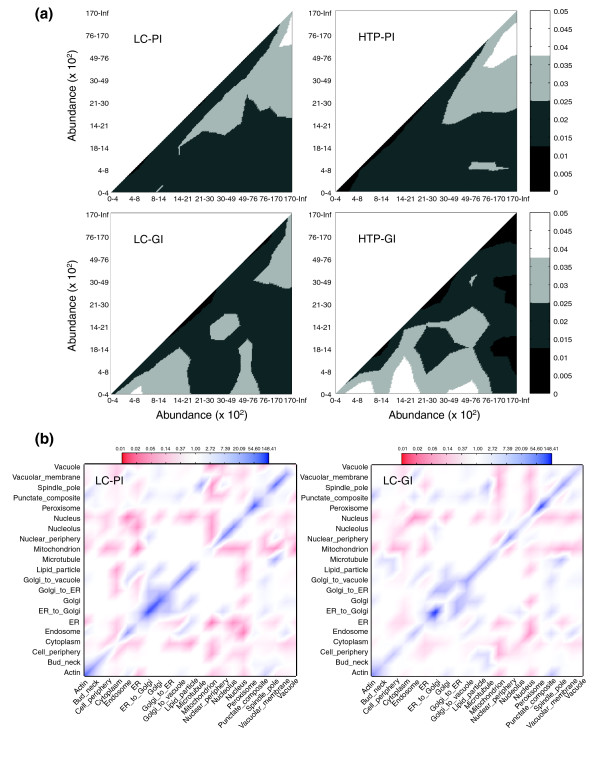
Correlation of interactions with protein abundance and localization. **(a) **Statistical enrichment of interaction pairs as a function of protein abundance for each indicated dataset. Protein or gene pairs were separated into bins representing increasing protein abundance as derived from a genome-wide analysis [67] and shaded according to enrichment over chance distribution (the scale bar indicates the fraction of total interactions, with lighter regions indicating enrichment). Inf indicates infinity. Raw abundance distributions in each dataset are provided in Additional data file 3. **(b) **Correlation ratios of interactions between proteins of different locality for LC-PI and LC-GI networks. Blue regions in the diagonal indicate that interactions within the locality group are enhanced, while the off-diagonal red regions indicate that interactions of proteins from different localities are suppressed. Nodes with multiple localities were treated as missing values. Proteome-wide localization annotation [68] was available for 1,404 proteins (around 52%) in the LC dataset. The expected number of interactions was generated using 200 iterations of randomized versions of both original networks. Random networks were generated by an edge-swapping procedure, which maintains the degree-distribution, and localization assignments were shuffled among those nodes that had a single locality (the scale bar indicates fold enrichment over chance).

The abundance of most predicted proteins in yeast has recently been determined [67]. Comparison of this dataset with all protein- and genetic-interaction datasets revealed that highly abundant proteins were more likely to exhibit detectable physical interactions, whereas low-abundance proteins were more likely to exhibit genetic interactions (Figure 8a). Both LC-PI and HTP-PI datasets exhibited a significant positive bias towards abundant proteins (*r *= 0.06, *P *= 0.0025 and *r *= 0.19, *P *= 2 × 10^-26 ^respectively, Spearman rank correlation), while LC-GI and HTP-GI exhibited a significant but weak negative bias (*r *= -0.06, *P *= 0.005 and *r *= -0.11, *P *= 9 × 10^-4 ^respectively, Spearman rank correlation). Interestingly, despite a stronger overall negative correlation with protein abundance, the systematic genetic analyses in the HTP-GI dataset were more uniformly distributed across protein-abundance bins, whereas the LC-GI interactions were more strongly represented in the lowest-abundance bins. This latter observation suggests that the phenotypes studied by conventional genetics may be focused on regulatory processes controlled by low-abundance proteins.

The localization of a large fraction of predicted proteins in yeast has also recently been determined [68]. Proteins that interact must at least partially overlap in subcellular location, and indeed, co-localization may be essential to drive interaction equilibrium for low-abundance proteins [69]. This expectation is borne out, as protein co-localization in the same compartment was significantly enriched for physical interaction pairs in the LC-PI dataset, whereas potential inter-compartment interactions were significantly under-represented (Figure 8b). Similar conclusions have been drawn previously for HTP datasets [27]. Although less pronounced, the correlation with subcellular localization also extended to genetic-interaction pairs (Figure 8b).

Analysis of HTP datasets in conjunction with genome-wide expression profiles across many experimental conditions has demonstrated that physical interaction partners are encoded by genes that tend to be co-regulated [26,70]. As judged by the Pearson correlation coefficients (PCC) for a compendium of 304 different genome-wide expression profiles [71], this propensity for co-regulation holds in the LC dataset, for both physical and genetic interactions (see Additional data file 3). Although highly statistically significant, the enrichment for positive over negative expression correlation was only around 5% for either dataset, such that this parameter only weakly predicts interactions. We also assessed the fraction of interaction partners that shared at least one transcription factor, as defined in genome-wide location studies [72]. For interaction pairs where each respective gene is bound by one or more transcription factors, 24% (397/1,637) of pairs in the LC-PI dataset had at least one shared transcription factor, compared with 15% (229/1,422) of pairs in the HTP-PI dataset. This significant difference (Fisher's exact test, *P *< 2 × 10^-8^, two-tailed) suggested that LC-PI was enriched for interactions between co-regulated proteins. For the LC-GI and HTP-GI datasets, shared transcription factors were found in 16% and 17% of pairs (229/1,422 and 117/672, respectively), a nonsignificant difference (Fisher's exact test, *P *= 0.45, two-tailed). For all datasets, these transcription factor co-location values were at least seven standard deviations from the mean calculated for a similar number of random pairs, consistent with the tendency of interacting proteins and genes to be coexpressed.

### Predictive power of the LC dataset

**Figure 9:**
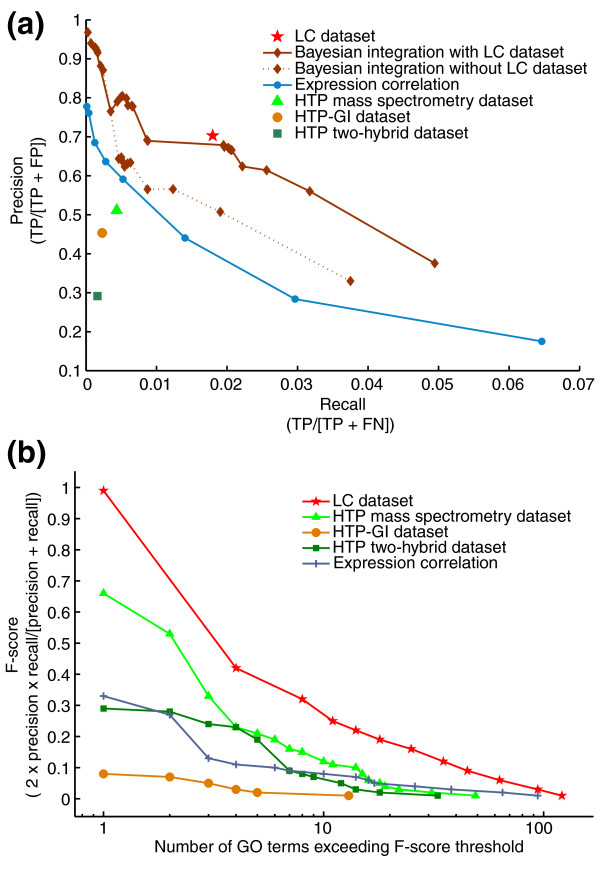
The LC dataset augments functional predictions. **(a) **Evaluation of curated literature against GO biological process as a standard. Comparisons of enrichment for functional relationships in LC dataset versus a variety of HTP datasets as scored against GO biological process are shown as the individual data points. The effect of the LC dataset on the predictive power of a Bayesian heterogeneous integration scheme [28] is shown by the curves. FN, false negatives; FP, false positives; TP, true positives. **(b) **Comparison of functional diversity in LC versus a variety of HTP datasets. The number of distinct functional groups (GO biological process terms) spanned by the LC dataset at decreasing levels of precision and recall. One hundred and forty-six independent GO terms were tested, all with fewer than 300 total annotations. A minimum F-score threshold (harmonic mean of precision and recall) was plotted against the number of GO terms needed to achieve that threshold for each of the data types.

Many different approaches have been devised to improve the power of large-scale datasets to predict gene or protein functions, including simple combinations of different datasets, Bayesian integration of multiple data sources, and inherent network properties of true versus false interactions [3,14,26-30]. To assess the capability of the LC dataset to assign new gene functions, we first evaluated the enrichment of known functional relationships in LC-PI pairs by comparing them with GO process annotations. We compared the LC pairs relative to a variety of HTP genomic data on the basis of both precision (that is, proportion of results known to be true positives) and recall (that is, proportion of known positives identified). The LC-PI dataset returned approximately 70% precision on about 14,000 pairs, as compared with 50% precision on 2,500 pairs for the HTP-PI dataset and 70% precision on only 800 true positive pairs for coexpression datasets (Figure 9a).

Recent developments in methods for gene or protein function prediction suggest that probabilistic integration of diverse genomic data is a powerful approach to the annotation of uncharacterized genes. Given its precision and substantial coverage, the LC dataset should augment these approaches. We have recently constructed a Bayesian network that integrates affinity precipitation, two-hybrid, synthetic lethality, and microarray correlation data [28]. The performance of this network was dramatically improved by the LC dataset: for a recall of 2% of a standard constructed from GO terms (about 11,000 pairs), the LC dataset improved prediction precision from 50% to 68% (Figure 9a).

Another important characteristic of any biological dataset is the diversity of functional groups covered. While precision-recall curves estimate the total number of true-positive pairs in the LC dataset, they do not specifically report the number of distinct biological processes captured by the data. To measure this diversity, we computed precision-recall statistics separately on the 146 largest GO terms under the 300-gene threshold for each data type, and counted the number of terms that meet a minimum combined precision-recall score, as measured by the commonly used F-score or harmonic mean. The diversity of coverage in the LC dataset was clearly superior to that in any of the HTP datasets (Figure 9b). For example, the LC dataset covered eight distinct biological processes at a minimum F-score threshold of 0.32, whereas the next best data type, HTP affinity precipitation, covered eight GO terms only when the F-score threshold was relaxed to 0.15. This increased diversity is an important consideration in functional prediction because the limiting factor in such analyses is often incomplete data.

### Prediction and coverage of protein complexes

**Figure 10:**
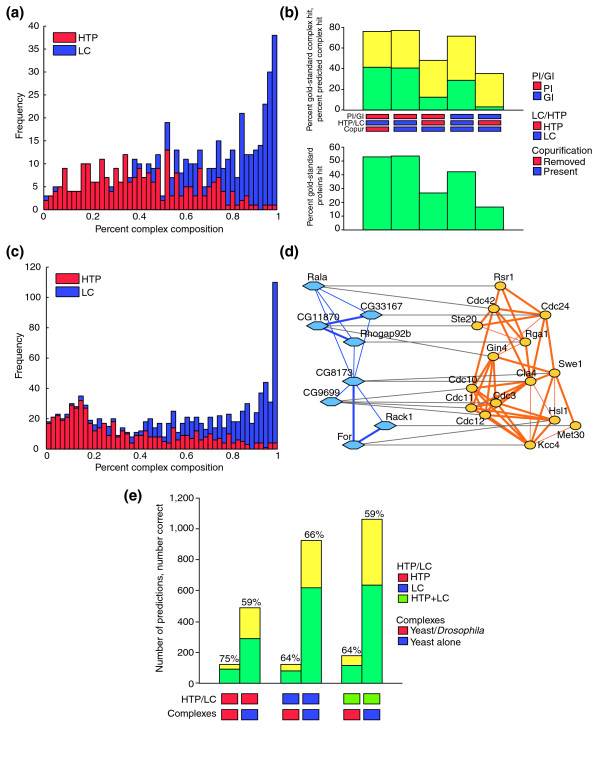
Interactions from the LC dataset dominate the composition of predicted protein complexes. **(a) **Contribution of HTP-PI and LC-PI data to predicted protein complexes. Each of the 420 predicted complexes are binned according to the percentage of LC (blue) or HTP (red) interactions it contains. The two distributions are not exact complements because some interactions are members of both LC-PI and HTP-PI. **(b) **The overlap of predicted protein complexes with actual protein complexes as defined by co-purification. For a predicted complex and a gold-standard complex, a hit is scored when the two sets of proteins produce a Jaccard similarity of ≥ 0.13. Top panel, green bars indicate the percentage of gold-standard complexes hit by some predicted complex. The sum of the green and yellow bars is the percentage of predicted complexes hit by some gold-standard complex. Bottom panel, the percentage of proteins in gold-standard complexes represented in all predicted complexes. This gives a rough upper bound on the percentage of gold-standard complexes that can be hit. **(c) **Complexes conserved between yeast and *Drosophila *are enriched in LC-PI interactions. This histogram is analogous to that shown for yeast-only complexes in Figure 10a. **(d) **Example of orthology between yeast and fly protein complexes in a cytoskeletal control network. The high degree of LC-PI interconnections between yeast proteins (orange) validates fly HTP interactions (blue) and suggests new potential connections to test between fly proteins. Thick lines indicate direct interactions, thin lines indicate interactions bridged by a common neighbor. Complex layouts were rendered in Cytoscape [97]. **(e) **Prediction of GO process annotations using conserved versus yeast-only complexes. Green bars indicate the number of correct predictions and yellow bars indicate the number of incorrect predictions, the sum of which is the total number of predictions. Complex and pathway prediction was carried out according to [31] and results were averaged over five rounds of full tenfold cross-validation.

A variety of computational approaches have been devised to infer protein complexes from partial interaction datasets [31,73-75]. We used the PathBLAST network alignment tool to identify prospective protein complexes in the combined LC-PI and HTP-PI networks as subnetworks of interactions that were significantly more densely connected than would be expected in randomized versions of the same network [31]. This method predicted a total of 539 yeast protein complexes in addition to (and excluding) the 258 definitive biochemically purified complexes already present in the LC-PI dataset (see Additional data file 1). The relative contributions of LC-PI versus HTP-PI data to the predicted complexes were assessed by counting interactions donated from each dataset (Figure 10a). As shown, the LC-PI dataset contributed the majority of interactions that formed the predicted complexes; thus, LC interactions show a greater tendency to cluster into complex-like structures. As another measure of enrichment for complexes in the LC-PI dataset, we assessed the overlap between the complexes predicted from local interaction density versus the 258 biochemically purified gold-standard complexes, again as a function of contributions from the LC versus HTP datasets (Figure 10b). Here again, the LC-PI dataset outperformed the HTP-PI dataset. The minimal overlap of locally dense regions in the LC-PI and HTP-PI datasets was also evident visually in two-dimensional hierarchical clustering maps of the combined datasets (see Additional data file 3).

### Pathway conservation

The predicted core proteome is substantially conserved across eukaryotes. For example, 37% of yeast proteins have identifiable orthologs in humans [76]. This concept has been recently extended to identify conserved protein pathways [31]. We assessed the ability of the LC-PI dataset to augment these pathway predictions, based on the current fly protein-interaction network of 20,720 unique interactions between 7,038 proteins in FlyBase [77]. We again searched the combined LC-PI and HTP-PI yeast networks for densely connected subnetworks suggestive of protein complexes, but in addition we made the requirement that the set of proteins in each complex has putative orthologs in fly that were also densely connected in the fly network. This process identified 1,412 putative conserved complexes between yeast and fly (see Additional data file 1). Like the single-species yeast complexes identified above (Figure 10a), the LC-PI dataset contributed the majority of interactions in the complexes conserved between yeast and fly (Figure 10c). As an example of such predicted complexes, a dense cytoskeletal control network in yeast corresponded to a partial network detected in the fly HTP dataset (Figure 10d). This orthologous network both buttresses known yeast interactions and suggests possible experiments to probe the cytoskeletal regulation in the fly. Finally, again based on the principle that interactions among orthologous genes are more likely to be true than those among nonorthologous genes, we used the LC-PI dataset to predict a set of 338 novel human protein interactions (see Additional data file 1).

The proteins grouped in a predicted complex are likely to share a common function. As with individual protein interactions, such co-association can be exploited to make high-quality protein functional predictions. We identified complexes that were already enriched for a particular GO function and transferred this function to all proteins in that complex (see Materials and methods). This process yielded between a hundred and a thousand new GO biological process annotations over all complexes, depending on whether HTP-PI or LC-PI data were used to identify complexes, and whether conserved yeast-only or yeast/fly complexes were specified (Figure 10e; see also Additional data file 1). LC-PI interactions resulted in substantially larger numbers of predictions than did HTP-PI interactions, at a percent accuracy that was roughly equivalent between the two (slightly higher for yeast-only complexes, slightly lower for yeast or fly complexes). Overall, the predictive power of complexes derived from the LC-PI dataset exceeds those derived from the HTP interactions.

## Discussion

Systematic curation of the *S. cerevisiae *primary literature enabled the creation of a comprehensive database that currently houses a total of 22,250 protein interactions and 11,061 genetic interactions, corresponding to 11,334 and 8,165 nonredundant interactions in the LC-PI and LC-GI datasets, respectively. This resource represents the distillation of more than three decades of yeast molecular genetics and biochemistry, as acquired by individual investigators. Because of the thorough coverage of the LC dataset, it will serve as a look-up table for gene and protein interactions and as a basis for interrogating the properties of biological networks. As shown above, the LC dataset improves the prediction of gene function and protein complexes, both within and between species. The sophisticated molecular genetics of budding yeast will facilitate definitive tests of hypotheses generated from analysis of the LC dataset.

### Interaction space: overlap between LC and HTP data

Simple comparison of the LC dataset reveals key differences between experimental data embedded in the literature as a whole and HTP data. The well known high rate of false-positive interactions in HTP physical interaction datasets is an inevitable consequence of nonspecific interactions inherent to different methods [33,34]. A more unexpected feature of the HTP datasets perhaps is the high rate of false-negative interactions in the original HTP datasets, a parameter that has not been possible to estimate until now. Thus, the overall overlap between HTP-PI and LC-PI datasets is only 14%, whereas even the most robust HTP interaction dataset contains less than 30% of known interactions for the particular baits studied. In conjunction with the observation that the better studied proteins or genes exhibit more interactions, the high false-negative rate in the HTP data suggests that interaction space may be far from saturated and that there are many more interactions to be discovered. The false-negative problem will undoubtedly be ameliorated by recent dramatic increases in mass spectrometer sensitivity [78] and application of more rigorous HTP approaches [79]. A second unexpected feature of the HTP datasets is the inherent bias towards previously studied interactions. This bias appears to derive in part from bait selection in nonsaturating studies. A final notable difference between the LC and HTP datasets is the dearth of genetic interactions in HTP screens that correspond to physical interactions. The apparent orthogonal relationship between HTP-PI and HTP-GI networks has been noted previously and explained on the basis of inter-pathway genetic interactions [12,64]. The substantial overlap between genetic and physical interactions observed in the LC datasets, although perhaps driven by investigator bias, belies a simple relationship between genetic and biochemical networks.

### Similar network properties of LC data and HTP data

The sparse coverage of true interactions in HTP datasets has numerous implications for previous network analyses, which of necessity have been based solely on HTP data. Importantly, four network properties deduced from HTP studies appear to hold in the LC-derived networks. First, the overall scale-free topology of biological networks deduced from HTP studies is supported by the LC dataset, albeit with regions of less ideal fit. This lack of fit may either reflect the bias in the LC-PI dataset, which results in enrichment of proteins with higher connectivity, or may reflect the fact that biological networks do not perfectly fit a power-law relationship [61,80]. Although there are relatively fewer hubs compared with non-hubs in the LC-PI network, this network nevertheless has significantly more highly connected hubs than other scale-free networks, such as the HTP-PI dataset. Second, the relationship between essentiality and connectivity also holds in the LC dataset. The large cohort of connections maintained by essential proteins may be a consequence of the fact that essential proteins tend to be more ancient, and have simply gained more interactions by chance. Third, protein-interaction partners tend to co-localize in the same subcellular compartment. Fourth, the modest propensity of protein-interaction partners to be coexpressed under different conditions is an attribute of both LC-PI and HTP-PI datasets.

### Essential-essential interactions unify the cellular network

The fourfold enrichment for essential-essential protein interactions observed in both the LC-PI and HTP-PI networks suggests that the global network may be unified by interactions between essential nodes. Indeed, a highly connected core of essential proteins with an exponential degree distribution has recently been noticed in HTP data [62]. This finding is buttressed by our observations that the LC-PI essential-essential interaction network is not only exponentially distributed, but is more dense, more complete and more connected than its nonessential counterpart. Although previous analysis of a HTP two-hybrid network revealed that hub-hub connections are suppressed, implying that the cellular network is modular [63], this property appears to be a consequence of the HTP dataset (N.N.B. and M.T., unpublished data). Our finding that genetic interactions between essential genes are also twofold enriched in the LC-GI dataset strongly suggests that essential-essential interactions are functionally significant. Consistently, a recent analysis indicates that essential genes may exhibit up to fivefold more synthetic lethal interactions than to nonessential genes [60]. The preponderance of essential-essential interactions has a critical bearing on the evolution of protein networks. Because essential proteins evolve more slowly than nonessential proteins [81], it seems likely that essentials are constrained to slowly coevolve with other essentials to which they are physically connected [82,83]. The properties of the global network may thus be dominated by a phalanx of interlinked essential hubs that have been co-selected by evolutionary pressure. This interconnectivity appears to be supported by the substantial overlap we observe between the LC-PI and LC-GI networks, a feature that is not evident in the HTP-GI network [12]. Unlike metabolic networks, which do exhibit modularity [84], this centralized architecture may not be readily amenable to interpretation through discrete categorization of gene and protein function.

### Network representation and bias

Static two-dimensional representations of biological networks are obviously an abstraction that artificially compresses temporally and spatially distinct regions of the network. Although the current LC dataset captures basic data about physical and genetic interactions, much other information remains to be extracted and compiled, including quantitative measures of protein and genetic interactions [67,85], spatio-temporal aspects of network organization [54,68], protein-DNA interactions [72] and the posttranslational modifications that modulate many protein interactions [86]. In addition, more complex attributes such as the directionality of interactions and functional dependencies must also be captured in a systematic manner. Much of this information is contextual in nature and depends on multiple lines of supporting evidence that is not easily codified. This information will, however, be crucial for modeling the dynamics of genetic and protein networks. For example, relationships extracted from the literature have recently been used to demonstrate that the budding yeast cell cycle behaves as a dynamic attractor [87] and to deduce patterns of information flow in a mammalian neuronal network [88]. Pathway databases such as Reactome [89] and the Kyoto Encyclopedia of Genes and Genomes (KEGG) [90] have begun to compile this type of information. The LC dataset will serve as a guidepost for curation of more complex features, from which more sophisticated global models can be built.

As noted above and elsewhere, inherent biases in methods and approaches can compromise any given dataset, whether it be in limits of detection, a propensity to recover certain classes of interaction, or study bias in the primary literature [16,17,75,91]. Comparison of various datasets can reveal biases, which can then be taken into account in interpretation of network properties. With the advent of systems-biology approaches, such integrated datasets within the same study are rapidly becoming the norm and will provide much needed internal consistency between different methods [92]. Moreover, as the sensitivity and reliability of HTP approaches continues to improve, interactions detected by these methods will dominate biological networks. The LC dataset will guide such approaches and facilitate the interpretation of new data.

### Future curation

To maximize portability and integration, systematic curation efforts will require a universal agreed upon structured vocabulary to describe interactions and associated features. The Protein Standards Initiative, a work group of the Human Proteome Organization (HUPO), has recently developed a molecular interaction record structure, called PSI-MI, for protein and genetic interaction data [46]. The PSI-MI format has been adopted by the IMEx consortium of interaction databases [45], which aims to freely distribute interaction data. The open exchange of interaction records between different databases will enable the necessary comparisons to achieve a curated dataset that is largely error free. In accord with IMEx guidelines, we are in the process of mapping our experimental evidence codes to the PSI-MI format, so that our ongoing curation efforts will conform to the PSI-MI standard.

Apart from applications in the benchmarking of HTP datasets, prediction of protein function and biological network modeling, systematic curation efforts will prove useful in other contexts. In particular, interactions curated from the literature provide a valuable independent means to assess the coherence of GO annotation. Validated interaction partners that bear discrepant GO annotations may indicate either novel biological connections, the need for harmonization of GO terms, or simply outright inconsistencies in the literature. Comprehensive LC interaction datasets allow these discrepancies to be readily found and re-evaluated. Given the considerable efforts involved in the Model Organism Database (MOD) and GO curation, a strong case can be made for linked curation of full interaction records, which already partially overlap with GO evidence codes [47,48]. We also endorse the concept of author-directed curation at the time of submission or publication; the capture of interaction data in simplified records would greatly augment systematic curation of the literature. Finally, large manually curated datasets will provide a critical benchmark for machine-based learning approaches to automate the curation of the literature [49]. Machine-assisted approaches, such as the Textpresso literature-search algorithm [93], will undoubtedly improve curation accuracy and efficiency.

## Conclusion

Comprehensive curation of reliable protein and genetic interactions from the primary biomedical literature establishes a critical benchmark for HTP datasets, augments prediction of gene or protein function and allows inference of system-level properties of biological networks. The systematic compilation of publicly available LC interaction datasets for other model organisms, including humans [42], will enable further insight into both individual gene functions and biological network features.

## Materials and methods

### Literature search and definition of datasets

The PubMed database was searched for relevant publications using the following criteria: (all yeast ORFs) + (Gene Name (all aliases)) AND + (Yeast + OR + *Saccharomyces *+ *cerevisiae*). We also read an additional 6,543 abstracts/papers curated by SGD that were missed in the original search, usually because a gene name was not present in the abstract. A total of 53,117 abstracts/papers as of 1 November, 2005 were manually curated using custom web-based tools. The curation system automatically tracked abstracts and/or full text read by each curator. Abstracts that contained '*Saccharomyces cerevisiae*' or 'yeast' and a gene name but that were not true *S. cerevisiae *publications, typically because the publication described a yeast homolog or two-hybrid interaction for another species, were designated 'Wrong Organism'.

The LC-PI dataset does not include interactions from the two extant HTP mass spectrometry studies in *S. cerevisiae *[8,9] or from the three extant HTP two-hybrid studies [5-7]. These latter five combined studies are referred to as the HTP-PI. A number of recent publications report what might be considered HTP data that has been cross-validated to various extents. These publications, designated either systematic interrogation (SI) and HTP survey (HS), were included in the LC-PI dataset for the purpose of analyses reported here, but may be readily segregated for future analysis (see Additional data file 1).

The LC-GI dataset is defined as all interactions derived from conventional genetic approaches, that is, those not based on systematic SGA and dSLAM screens of the yeast deletion set [10,12,13] All genetic interactions from systematic screens comprise the HTP-GI dataset (see Additional data file 1 for the list of publications that document HTP-GI data).

### Annotation

The experimental methods for physical interactions were classified as follows:

#### Affinity capture-MS

The bait protein is affinity captured from cell extracts by either polyclonal antibody or epitope tag and the associated interaction partner is identified by MS methods.

#### Affinity capture-western

The bait protein is affinity captured from cell extracts by either polyclonal antibody or epitope tag and the associated interaction partner is identified by western blot with a specific polyclonal antibody or a second epitope tag. This category was also used if an interacting protein was visualized directly by dye stain or radioactivity.

#### Biochemical activity

Interaction is inferred from a biochemical effect of one protein upon another, for example, GTP-GDP exchange activity or phosphorylation of a substrate by a kinase.

#### Co-crystal structure

Interaction is directly demonstrated at the atomic level by X-ray crystallography.

#### Co-fractionation

Interaction is inferred from the presence of two or more protein subunits in a partially purified protein preparation.

#### Co-localization

Interaction is inferred from two proteins that co-localize in the cell by indirect immunofluorescence, usually in a co-dependent manner. This category also includes co-dependent association of proteins with promoter DNA in chromatin immunoprecipitation experiments.

#### Co-purification

Interaction is inferred from the identification of two or more protein subunits in a purified protein complex, as obtained by classical biochemical fractionation or by affinity purification and one or more additional fractionation steps. Because the bait-prey relationship does not exist for conventional purification, in those cases where an experimentally tagged bait protein was not present, a virtual bait was defined as the most highly connected protein according to other types of experimental evidence in the data-set. Co-purified complexes are listed in Additional data file 1.

#### Far western

Interaction is detected between a protein immobilized on a membrane and a purified protein probe.

#### FRET

The close proximity of interaction partners is detected by fluorescence resonance energy transfer (FRET) between cyan fusion protein (CFP) and yellow fluorescent protein (YFP) fusion proteins *in vivo*.

#### Protein-peptide

Interaction is detected between a protein and a peptide derived from an interaction partner. This category includes phage-display experiments.

#### Protein-RNA

Interaction is detected between a purified protein and associated RNA(s) as detected by northern blot or reverse transcription-PCR. Genome-wide experiments based on microarray detection were classified as HTP, and not recorded, unless supporting documentation for specific interactions was provided.

#### Reconstituted complex

Interaction is directly detected between purified proteins *in vitro*, usually in recombinant form.

#### Two-hybrid

The bait protein is expressed as a DNA-binding domain fusion and the prey protein is expressed as a transcriptional activation domain fusion and interaction is measured by reporter gene activation. This category was also used for two-hybrid variations such as the split-ubiquitin assay.

The experimental methods for genetic interactions were classified as follows:

#### Dosage growth defect

The overexpression or increased dosage of one gene causes a growth defect in a strain that is mutated or deleted for another gene.

#### Dosage lethality

The overexpression or increased dosage of one gene causes lethality in a strain that is mutated or deleted for another gene.

#### Dosage rescue

The overexpression or increased dosage of one gene rescues the lethality or growth defect of a strain that is mutated or deleted for another gene.

#### Synthetic growth defect

Mutations or deletions in separate genes, each of which alone causes a minimal phenotype but when combined in the same cell results in a significant growth defect under a given condition.

#### Synthetic lethality

Mutations or deletions in separate genes, each of which alone causes a minimal phenotype but when combined in the same cell results in lethality under a given condition.

#### Synthetic rescue

A mutation or deletion of one gene rescues the lethality or growth defect of a strain mutated or deleted for another gene.

#### Phenotypic enhancement

The mutation, deletion, or overexpression of one gene results in enhancement of any phenotype associated with the mutation, deletion, or over-expression of another gene.

#### Phenotypic suppression

The mutation, deletion, or over-expression of one gene results in the suppression of any phenotype associated with the mutation, deletion, or over-expression of another gene.

At this stage of curation, multiple genetic dependencies and strain background context were not routinely recorded, nor was the possible directionality of genetic interactions inferred.

#### Calculations

To estimate excess publication bias in the literature dataset, a bias for a protein or gene ν was defined as the number of interactions ν is part of, minus the connectivity of ν. Thus, if the connectivity of ν is *k *and ν is seen in *k *interactions, then the bias is 0; however, if ν is seen in, for example, 2*k *interactions, the bias is 2. Bias was computed for nodes in each dataset. Fits to power-law curves [18], expression correlation analyses [26,70], clustering coefficients [21], and hierarchical clustering [94] were computed essentially as described. Standard statistical tests were used throughout.

#### Functional prediction

We evaluated the enrichment of known functional relationships in the curated literature and other HTP data using GO biological process terms as a benchmark. Specifically, we compared protein pairs identified in curation or HTP data to those annotated to the same nodes in GO. We propagated each biological process annotation up to its ancestors to ensure a general evaluation base on the full GO hierarchy. To prevent proteins co-annotated to very general terms (such as 'metabolism') from being considered true positives, the number of unique annotations per GO term was counted. Because the biological specificity of each term roughly corresponds to the number of total annotations, we choose two thresholds to define the set of positive and negative protein pairs. Protein pairs whose most specific co-annotation occurs in GO terms of 300 total annotations or less are considered positives, while pairs whose most specific co-annotation occurs in GO terms of 1000 total annotations or more are considered negatives. The positive set spans around 1,600 terms, totaling some 500,000 pairs, and the negative set spans 10 nodes, totaling around 6 million pairs. The exact choice of GO term size threshold is not critical. Evaluation results are consistent for any choices between 150 and 400 genes when the negative co-annotation term size threshold is fixed at 1,000. Details of predictive methods are provided in Additional data file 2.

#### Protein complex and pathway prediction

Identification of protein complexes was performed using the PathBLAST network alignment tools, as previously described [31]. Briefly, these methods integrate protein-interaction data from two species with protein sequence homology to generate an aligned network, in which each node represents a pair of homologous proteins (one from each species; BLAST E-value < 10^-7^) and each link represents a conserved interaction. We note that representation of the network as either a spoke or matrix model does not affect the outcome of PathBLAST predictions because computations for conserved complexes include both direct and indirect interactions. That is, proteins that are bridged by a third protein are automatically linked in the PathBLAST network and assigned only a slight penalty. PathBLAST is thus robust to possible incomplete coverage in one network versus another. Given this design, spoke versus matrix representation models yield very similar complex predictions and network topologies.

The PathBLAST network alignment was searched to identify high-scoring subnetworks, for which the score is based on the density of interactions within the subnetwork as well as the confidence estimates for each protein interaction (see below). The search was then repeated over 100 random trials, in which the interactions of both networks are reassigned while maintaining the same number of interactions per protein, resulting in a distribution of random subnetwork scores pooled over all trials. Dense subnetworks that score in the top 1% of this random score distribution are considered significant and retained as conserved complexes. To minimize redundancy, complexes are filtered against each other such that if the sets of proteins from any two complexes overlap by more than 80%, the lower-scoring complex is discarded. The search for single-species complexes is identical to the search for conserved complexes except that an individual protein network is substituted for the network alignment. This process identifies dense subnetworks constrained by the interactions of one species rather than two. In the fly, confidence estimates for each interaction were derived using a logistic regression model similar to that previously described [95]; in yeast, so as not to bias one set of interactions over the other, interactions were assigned a uniform confidence of 0.99. Given a set of significant protein complexes, these complexes are used to predict new protein functional annotations, as follows. A GO functional term *f *is assigned to protein *P *of complex *c *if: (1) at least five proteins in *c *are already annotated with *f*; (2) at least 50% of the proteins in *c *are annotated with *f*; and (3) *c *is enriched for *f *by a hypergeometric *P*-value < 0.01; and (4) *f *is a sufficiently specific term at level 4 or deeper in the GO ontology. To assess the predictive power of significant complexes, we use tenfold cross-validation. In this procedure, the set of known GO annotations is partitioned into ten equal subsets, and each of these is hidden in turn. The fraction of hidden annotations that is recapitulated using the prediction algorithm is determined.

For predicted interactions between human proteins, yeast-human orthologs were stringently identified by reciprocal best-hit BLAST scores of *e*-value < 10^-10 ^and sequence identity of > 50%. Human protein interactions were obtained from HPRD [42] and human protein sequences from the National Center for Biotechnology Information (NCBI). For each interaction in the LC-PI dataset set, if both proteins had a human ortholog and the interaction between these orthologs was not reported in HPRD, a predicted interaction was scored.

#### Distribution, updates and maintenance

The complete LC dataset is freely available at the BioGRID interaction database [44] and at the *Saccharomyces *Genome Database [51]. The LC dataset will be kept current through monthly updates and refined through re-curation and community-directed corrections. In future curation updates, all the above protein- and genetic-interaction evidence categories will be mapped to PSI-MI terms [46].

#### Note added in proof

Two comprehensive surveys of protein interactions, as determined by mass spectrometric analysis of affinity purified protein complexes, have recently been reported [109,110]. The raw dataset in Gavin *et al*. [109] overlaps with 21% of  the LC-PI dataset and 29% of the HTP-PI dataset, while the raw dataset in Krogan *et al*. [110] overlaps with 22% of the LC-PI dataset and 14% of the HTP-PI dataset. The sum total of all HTP-PI data, including recent data [109,110], overlaps with 34% of the LC-PI dataset. These comparisons suggest that protein interaction space is far from saturated in extant datasets.

## Additional data files

The following additional data files are available with this article. Additional data file 1 contains Supplementary Tables 1–11: Supplementary Table 1, LC and HTP dataset statistics; Supplementary Table 2, Co-purified complexes in the LC dataset; Supplementary Table 3, SI/HTP publications; Supplementary Table 4, Post-translational modifications associated with interactions; Supplementary Table 5, Overlap of physical and genetic interaction datasets; Supplementary Table 6, Predicted yeast complexes from yeast interaction datasets; Supplementary Table 7, Predicted yeast complexes from yeast and fly interaction datasets; Supplementary Table 8, Novel human predicted human protein interactions; Supplementary Table 9, Novel GO functional predictions for yeast proteins; Supplementary Table 10, Novel GO functional predictions for fly proteins; Supplementary Table 11, Publications documented in the HTP-GI dataset. Additional data file 2 contains a comparison of the LC dataset with other curated datasets and details of functional predictions. Additional data file 3 contains Supplementary Figures 1-6: Supplementary Figure 1, Curation benchmarks for the LC dataset; Supplementary Figure 2, Distribution of terms in GO categories in LC-PI and LC-GI dataset; Supplementary Figure 3, Relative coverage and overlap of interaction datasets; Supplementary Figure 4, Raw distributions of interactions for each indicated dataset as a function of protein abundance; Supplementary Figure 5, Expression correlation for interaction pairs in LC versus HTP datasets; Supplementary Figure 6, Dense regions in the physical interaction network. Additional data file 4 contains flat files of the main datasets.

## Supplementary Material

Additional data file 1 Supplementary Table 1, LC and HTP dataset statistics; Supplementary Table 2, Co-purified complexes in the LC dataset; Supplementary Table 3, SI/HTP publications; Supplementary Table 4, Post-translational modifications associated with interactions; Supplementary Table 5, Overlap of physical and genetic interaction datasets; Supplementary Table 6, Predicted yeast complexes from yeast interaction datasets; Supplementary Table 7, Predicted yeast complexes from yeast and fly interaction datasets; Supplementary Table 8, Novel human predicted human protein interactions; Supplementary Table 9, Novel GO functional predictions for yeast proteins; Supplementary Table 10, Novel GO functional predictions for fly proteins; Supplementary Table 11, Publications documented in the HTP-GI dataset Click here for additional data file

Additional data file 2 A comparison of the LC dataset with other curated datasets and details of functional predictions Click here for additional data file

Additional data file 3 Supplementary Figure 1, Curation benchmarks for the LC dataset; Supplementary Figure 2, Distribution of terms in GO categories in LC-PI and LC-GI dataset; Supplementary Figure 3, Relative coverage and overlap of interaction datasets; Supplementary Figure 4, Raw distributions of interactions for each indicated dataset as a function of protein abundance; Supplementary Figure 5, Expression correlation for interaction pairs in LC versus HTP datasets; Supplementary Figure 6, Dense regions in the physical interaction network Click here for additional data file

Additional data file 4 Flat files of the main datasets Click here for additional data file

## Figures and Tables

**Table 1:** Literature-curated datasets

Datasets	Number of total nodes	Number of edges	Number of baits	Number of sources
Total interactions (includes self edges, multiple sources/experimental systems, RNA genes)				
HTP-PI	4,478	12,994	2,387	5
LC-PI	3,342	22,250	2,047	3,342
HTP-GI	1,454	8,111	260	39
LC-GI	2,689	11,061	1,854	3,798
Total	5,467	54,416	3,728	6,170
Total LC (LC-PI+LC-GI)	3,904	33,311	2,635	6,148
				
Filtered interactions (excludes self edges, redundant edges, RNA genes in LC-PI)				
HTP-PI	4,474	11,571	2,353	5
LC-PI	3,289	11,334	1,969	3,202
Total PI (HTP-PI+LC-PI)	5,107	21,281	3,254	3,207
HTP-GI	1,454	6,103	260	39
LC-GI	2,689	8,165	1,854	3,796
Total GI (HTP-GI+LC-GI)	3,258	13,963	1,923	3,826
Total (Total PI + Total GI)	5,438	*35,244	3,665	5,977
Total LC (LC-PI+LC-GI)	3,863	*19,499	2,569	5,956

*Values represent the sums of the respective datasets (that is, overlap between PI and GI not removed).
