# Supplementary material for: Comprehensive curation and analysis of global interaction networks in Saccharomyces cerevisiae
Source: J Biol. 2006 Jun 8;5(4):11. doi: 10.1186/jbiol36 (PMC1561585; doi:10.1186/jbiol36)
Supplement: Additional data file 1 — Supplementary Table 1, LC and HTP dataset statistics; Supplementary Table 2, Co-purified complexes in the LC dataset; Supplementary Table 3, SI/HTP publications; Supplementary Table 4, Post-translational modifications associated with interactions; Supplementary Table 5, Overlap of physical and genetic interaction datasets; Supplementary Table 6, Predicted yeast complexes from yeast interaction datasets; Supplementary Table 7, Predicted yeast complexes from yeast and fly interaction datasets; Supplementary Table 8, Novel human predicted human protein interactions; Supplementary Table 9, Novel GO functional predictions for yeast proteins; Supplementary Table 10, Novel GO functional predictions for fly proteins; Supplementary Table 11, Publications documented in the HTP-GI dataset [file jbiol36-s1.pdf]

**Additional data file 1**

## LIST OF SUPPLEMENTARY TABLES

Supplementary Table 1. LC and HTP dataset statistics.
Supplementary Table 2. Co-purified complexes in the LC dataset.
Supplementary Table 3. SI/HTP publications
Supplementary Table 4. Post-translational modifications associated with interactions
Supplementary Table 5. Overlap of physical and genetic interaction datasets.
Supplementary Table 6. Predicted yeast complexes from yeast interaction datasets.
Supplementary Table 7. Predicted yeast complexes from yeast and fly interaction datasets.
Supplementary Table 8. Novel human predicted human protein interactions.
Supplementary Table 9. Novel GO functional predictions for yeast proteins.
Supplementary Table 10. Novel GO functional predictions for fly proteins.
Supplementary Table 11. Publications  documented in the HTP-GI dataset.

# Supplementary Table 1. LC and HTP dataset statistics.

| **Network Statistics** | | | | | | | | | |
|---|---|---|---|---|---|---|---|---|---|
| | | | | | | | | | |
| **Raw Interactions (including self edges, multiple sources/experimental systems, RNA genes)** | | | | | | | | | |
| | | | | | | | | | |
| **Datasets** | **# total nodes** | **# protein nodes** | **# rna nodes** | **# edges** | **# e-e edges** | **# of Edges with at least 1 essential Node** | **# baits** | **# essential baits** | **# source s** |
| HTP-PI | 4478 | 4478 | 0 | 12994 | 2264 | 6796 | 2387 | 559 | 5 |
| LC-PI | 3342 | 3310 | 32 | 22250 | 6657 | 14677 | 2047 | 692 | 3342 |
| HTP-GI | 1454 | 1454 | 0 | 8111 | 14 | 922 | 260 | 31 | 39 |
| LC-GI | 2689 | 2678 | 11 | 11061 | 1628 | 5147 | 1854 | 534 | 3798 |
| **Total** | **5467** | **5434** | **33** | **54416** | **10563** | **27542** | **3728** | **892** | **6170** |
| | | | | | | | | | |
| **Total LC (LC-PI+LC-GI)** | **3904** | **3871** | **33** | **33311** | **8285** | **19824** | **2635** | **780** | **6148** |
| | | | | | | | | | |
| **Filtered Interactions (excluding self edges, redundant edges, RNA genes in LC_PI only)** | | | | | | | | | |
| | | | | | | | | | |
| **Datasets** | **# total nodes** | **# protein nodes** | **# rna nodes** | **# edges** | **# e-e edges** | **# of Edges with at least 1 essential Node** | **# baits** | **# essential baits** | **# source s** |
| HTP-PI | 4474 | 4474 | 0 | 11571 | 1874 | 6094 | 2353 | 551 | 5 |
| LC-PI | 3289 | 3289 | 0 | 11334 | 3050 | 7522 | 1969 | 674 | 3202 |
| **Total PI (HTP-PI+LC-PI)** | **5107** | **5107** | **0** | **21281** | **4232** | **12455** | **3254** | **821** | **3207** |
| HTP-GI | 1454 | 1454 | 0 | 6103 | 14 | 892 | 260 | 31 | 39 |
| LC-GI | 2689 | 2678 | 11 | 8165 | 1096 | 3784 | 1854 | 534 | 3796 |
| **Total GI (HTP-GI+LC-GI)** | **3258** | **3247** | **11** | **13963** | **1109** | **4631** | **1923** | **535** | **3826** |
| **Total (Total PI + Total GI)** | **5438** | **5427** | **11** | ***35244** | **4878** | **16026** | **3665** | **884** | **5977** |
| | | | | | | | | | |
| **Total LC (LC-PI+LC-GI)** | **3863** | **3852** | **11** | ***19499** | **3696** | **10316** | **2569** | **767** | **5956** |
| *** Values represent the sums of the respective datasets (i.e., overlap between PI and GI not removed)** | | | | | | | | | |
| | | | | | | | | | |
| **Filtered Interaction categories (excluding self edges, redundant edges, RNA genes in LC_PI only)** | | | | | | | | | |
| | | | | | | | | | |
| **HTP-PI** | | | | | | | | | |

| Experimental System | # total nodes | # protein nodes | # rna nodes | # edges | # e-e edges | # of Edges with at least 1 essential Node | # baits | # essential baits | # sources |
|---|---|---|---|---|---|---|---|---|---|
| Affinity Capture-MS | 2274 | 2274 | 0 | 6627 | 1591 | 4201 | 864 | 306 | 2 |
| Two-hybrid | 3561 | 3561 | 0 | 5102 | 334 | 1980 | 1842 | 379 | 3 |
| **total** | **4474** | **4474** | **0** | **11571** | **1874** | **6094** | **2353** | **551** | **5** |
| | | | | | | | | | |
| **LC-PI** | | | | | | | | | |
| **Experimental System** | **# total nodes** | **# protein nodes** | **# rna nodes** | **# edges** | **# e-e edges** | **# of Edges with at least 1 essential Node** | **# baits** | **# essential baits** | **# sources** |
| Affinity Capture-MS | 1546 | 1546 | 0 | 4499 | 1776 | 3640 | 489 | 265 | 204 |
| Affinity Capture-Western | 1808 | 1808 | 0 | 3506 | 912 | 2132 | 1293 | 462 | 1639 |
| Two-hybrid | 2012 | 2012 | 0 | 3351 | 562 | 1752 | 998 | 344 | 995 |
| Reconstituted Complex | 992 | 992 | 0 | 1310 | 366 | 871 | 600 | 241 | 825 |
| Co-purification | 673 | 673 | 0 | 889 | 387 | 725 | 187 | 94 | 223 |
| Biochemical Activity | 497 | 497 | 0 | 760 | 103 | 418 | 159 | 47 | 322 |
| Co-fractionation | 350 | 350 | 0 | 317 | 103 | 178 | 131 | 60 | 130 |
| Co-localization | 175 | 175 | 0 | 161 | 38 | 97 | 85 | 29 | 85 |
| Co-crystal Structure | 68 | 68 | 0 | 66 | 38 | 51 | 39 | 21 | 40 |
| Protein-peptide | 65 | 65 | 0 | 62 | 5 | 21 | 26 | 7 | 26 |
| Affinity Capture-RNA | 28 | 28 | 0 | 39 | 3 | 26 | 14 | 4 | 8 |
| Far Western | 51 | 51 | 0 | 36 | 14 | 27 | 31 | 17 | 25 |
| FRET | 30 | 30 | 0 | 32 | 15 | 28 | 23 | 14 | 6 |
| Protein-RNA | 12 | 12 | 0 | 6 | 0 | 1 | 6 | 1 | 7 |
| **total** | **3289** | **3289** | **0** | **11334** | **3050** | **7522** | **1969** | **674** | **3202** |
| | | | | | | | | | |
| | | | | | | | | | |
| **HTP-GI** | | | | | | | | | |
| **Experimental System** | **# total nodes** | **# protein nodes** | **# rna nodes** | **# edges** | **# e-e edges** | **# of Edges with at least 1 essential Node** | **# baits** | **# essential baits** | **# sources** |
| Synthetic Lethality | 1402 | 1402 | 0 | 5887 | 7 | 812 | 237 | 27 | 37 |
| Synthetic Growth Defect | 218 | 218 | 0 | 261 | 7 | 65 | 17 | 7 | 10 |
| Synthetic Rescue | 14 | 14 | 0 | 13 | 0 | 10 | 8 | 6 | 3 |
| Phenotypic Suppression | 15 | 15 | 0 | 13 | 0 | 1 | 13 | 0 | 2 |

| | | | | | | | | | |
|---|---|---|---|---|---|---|---|---|---|
| Dosage Growth Defect | 9 | 9 | 0 | 8 | 0 | 8 | 8 | 0 | 1 |
| Dosage Lethality | 2 | 2 | 0 | 1 | 0 | 1 | 1 | 0 | 1 |
| Phenotypic Enhancement | 2 | 2 | 0 | 1 | 0 | 0 | 1 | 0 | 1 |
| **total** | **1454** | **1454** | **0** | **6103** | **14** | **892** | **260** | **31** | **39** |
| | | | | | | | | | |
| | | | | | | | | | |
| **LC-GI** | | | | | | | | | |
| **Experimental System** | **# total nodes** | **# protein nodes** | **# rna nodes** | **# edges** | **# e-e edges** | **# of Edges with at least 1 essential Node** | **# baits** | **# essential baits** | **# sources** |
| Dosage Rescue | 1578 | 1571 | 7 | 2456 | 432 | 1349 | 926 | 374 | 1280 |
| Synthetic Lethality | 1285 | 1280 | 5 | 2172 | 435 | 1207 | 822 | 266 | 1001 |
| Synthetic Rescue | 1106 | 1102 | 4 | 1581 | 148 | 676 | 614 | 200 | 886 |
| Phenotypic Enhancement | 1055 | 1054 | 1 | 1421 | 68 | 360 | 659 | 115 | 910 |
| Synthetic Growth Defect | 847 | 846 | 1 | 982 | 119 | 412 | 454 | 122 | 468 |
| Phenotypic Suppression | 502 | 498 | 4 | 507 | 42 | 164 | 249 | 65 | 230 |
| Dosage Lethality | 359 | 359 | 0 | 354 | 69 | 237 | 214 | 104 | 169 |
| Dosage Growth Defect | 29 | 29 | 0 | 18 | 4 | 14 | 14 | 9 | 12 |
| **total** | **2689** | **2678** | **11** | **8165** | **1096** | **3784** | **1854** | **534** | **3796** |
| | | | | | | | | | |
| | | | | | | | | | |
| | | | | | | | | | |
| **Total** | | | | | | | | | |
| **Experimental System** | **# total nodes** | **# protein nodes** | **# rna nodes** | **# edges** | **# e-e edges** | **# of Edges with at least 1 essential Node** | **# baits** | **# essential baits** | **# sources** |
| Affinity Capture-MS | 2758 | 2758 | 0 | 10316 | 2912 | 7157 | 1171 | 458 | 206 |
| Two-hybrid | 4305 | 4305 | 0 | 8193 | 826 | 3588 | 2413 | 567 | 998 |
| Synthetic Lethality | 2165 | 2160 | 5 | 7859 | 442 | 1996 | 932 | 274 | 1036 |
| Affinity Capture-Western | 1808 | 1808 | 0 | 3506 | 912 | 2132 | 1293 | 462 | 1639 |
| Dosage Rescue | 1578 | 1571 | 7 | 2456 | 432 | 1349 | 926 | 374 | 1280 |
| Synthetic Rescue | 1115 | 1111 | 4 | 1592 | 148 | 684 | 620 | 204 | 889 |
| Phenotypic Enhancement | 1055 | 1054 | 1 | 1422 | 68 | 360 | 659 | 115 | 911 |

| | | | | | | | | | |
|---|---|---|---|---|---|---|---|---|---|
| Reconstituted Complex | 992 | 992 | 0 | 1310 | 366 | 871 | 600 | 241 | 825 |
| Synthetic Growth Defect | 975 | 974 | 1 | 1241 | 126 | 477 | 462 | 124 | 477 |
| Co-purification | 673 | 673 | 0 | 889 | 387 | 725 | 187 | 94 | 223 |
| Biochemical Activity | 497 | 497 | 0 | 760 | 103 | 418 | 159 | 47 | 322 |
| Phenotypic Suppression | 510 | 506 | 4 | 519 | 42 | 165 | 258 | 65 | 232 |
| Dosage Lethality | 361 | 361 | 0 | 355 | 69 | 238 | 215 | 104 | 170 |
| Co-fractionation | 350 | 350 | 0 | 317 | 103 | 178 | 131 | 60 | 130 |
| Co-localization | 175 | 175 | 0 | 161 | 38 | 97 | 85 | 29 | 85 |
| Co-crystal Structure | 68 | 68 | 0 | 66 | 38 | 51 | 39 | 21 | 40 |
| Protein-peptide | 65 | 65 | 0 | 62 | 5 | 21 | 26 | 7 | 26 |
| Affinity Capture-RNA | 28 | 28 | 0 | 39 | 3 | 26 | 14 | 4 | 8 |
| Far Western | 51 | 51 | 0 | 36 | 14 | 27 | 31 | 17 | 25 |
| FRET | 30 | 30 | 0 | 32 | 15 | 28 | 23 | 14 | 6 |
| Dosage Growth Defect | 38 | 38 | 0 | 26 | 4 | 22 | 22 | 9 | 13 |
| Protein-RNA | 12 | 12 | 0 | 6 | 0 | 1 | 6 | 1 | 7 |
| **total** | **5442** | **5431** | **11** | **33688** | **4886** | **16100** | **3675** | **884** | **6043** |
| | | | | | | | | | |
| | | | | | | | | | |
| **Total LC (LC-PI+LC-GI)** | | | | | | | | | |
| **Experimental System** | **# total nodes** | **# protein nodes** | **# rna nodes** | **# edges** | **# e-e edges** | **# of Edges with at least 1 essential Node** | **# baits** | **# essential baits** | **# sources** |
| Affinity Capture-MS | 1546 | 1546 | 0 | 4499 | 1776 | 3640 | 489 | 265 | 204 |
| Affinity Capture-Western | 1808 | 1808 | 0 | 3506 | 912 | 2132 | 1293 | 462 | 1639 |
| Two-hybrid | 2012 | 2012 | 0 | 3351 | 562 | 1752 | 998 | 344 | 995 |
| Dosage Rescue | 1578 | 1571 | 7 | 2456 | 432 | 1349 | 926 | 374 | 1280 |
| Synthetic Lethality | 1285 | 1280 | 5 | 2172 | 435 | 1207 | 822 | 266 | 1001 |
| Synthetic Rescue | 1106 | 1102 | 4 | 1581 | 148 | 676 | 614 | 200 | 886 |
| Phenotypic Enhancement | 1055 | 1054 | 1 | 1421 | 68 | 360 | 659 | 115 | 910 |
| Reconstituted Complex | 992 | 992 | 0 | 1310 | 366 | 871 | 600 | 241 | 825 |
| Synthetic Growth Defect | 847 | 846 | 1 | 982 | 119 | 412 | 454 | 122 | 468 |
| Co-purification | 673 | 673 | 0 | 889 | 387 | 725 | 187 | 94 | 223 |

| | | | | | | | | | |
|---|---|---|---|---|---|---|---|---|---|
| Biochemical Activity | 497 | 497 | 0 | 760 | 103 | 418 | 159 | 47 | 322 |
| Phenotypic Suppression | 502 | 498 | 4 | 507 | 42 | 164 | 249 | 65 | 230 |
| Dosage Lethality | 359 | 359 | 0 | 354 | 69 | 237 | 214 | 104 | 169 |
| Co-fractionation | 350 | 350 | 0 | 317 | 103 | 178 | 131 | 60 | 130 |
| Co-localization | 175 | 175 | 0 | 161 | 38 | 97 | 85 | 29 | 85 |
| Co-crystal Structure | 68 | 68 | 0 | 66 | 38 | 51 | 39 | 21 | 40 |
| Protein-peptide | 65 | 65 | 0 | 62 | 5 | 21 | 26 | 7 | 26 |
| Affinity Capture-RNA | 28 | 28 | 0 | 39 | 3 | 26 | 14 | 4 | 8 |
| Far Western | 51 | 51 | 0 | 36 | 14 | 27 | 31 | 17 | 25 |
| FRET | 30 | 30 | 0 | 32 | 15 | 28 | 23 | 14 | 6 |
| Dosage Growth Defect | 29 | 29 | 0 | 18 | 4 | 14 | 14 | 9 | 12 |
| Protein-RNA | 12 | 12 | 0 | 6 | 0 | 1 | 6 | 1 | 7 |
| **total** | **3875** | **3864** | **11** | **18090** | **3704** | **10376** | **2585** | **767** | **6021** |

**Supplementary Table 2. Co-purified complexes in the LC dataset.**

| Complex ID | PubMed ID | YORF's |
|---|---|---|
| 1 | 12419231 | YPL228W \| YGL130W |
| 2 | 12419236 | YDR448W \| YDR176W \| YGR252W \| YOL148C \| YDR392W \| YDR145W |
| 3 | 11598206 | YOR069W \| YOR132W |
| 4 | 14581483 | YFR010W \| YFR004W |
| 5 | 9015380 | YJL002C \| YEL002C \| YOR085W \| YOR103C \| YMR149W |
| 6 | 12077334 | YBL052C \| YPL129W |
| 7 | 8663399 | YIR022W \| YJR010C-A |
| 8 | 10024884 | YDL140C \| YLR384C \| YOR151C \| YIL021W \| YJL140W \| YBR154C |
| 9 | 9388185 | YMR264W \| YMR022W |
| 10 | 8887631 | YER012W \| YGL048C \| YHR200W \| YKL145W |
| 11 | 12403464 | YDL201W \| YDR165W |
| 12 | 8135547 | YIL035C \| YOR061W \| YGL019W \| YOR039W |
| 13 | 8127913 | YOR290C \| YBR289W \| YJL176C \| YHL025W |
| 14 | 7877695 | YGR104C \| YDL140C \| YHR041C \| YER022W \| YBR253W \| YPL042C \| YNL025C |
| 15 | 10791972 | YOR341W \| YDL014W \| YMR229C \| YML074C \| YBR049C |
| 16 | 1406674 | YPL248C \| YML051W |
| 17 | 10791972 | YOR340C \| YMR229C \| YOR341W \| YPR010C \| YLR175W \| YLR449W \| YML074C \| YNL248C \| YMR230W \| YOR096W \| YDL208W \| YDR156W |
| 18 | 12408819 | YFR010W \| YHL030W \| YGL141W |
| 19 | 12408819 | YFR004W \| YHR200W \| YKL145W \| YFR010W |
| 20 | 11032814 | YNL102W \| YKL125W |
| 21 | 10323869 | YBR160W \| YBR135W \| YER012W \| YER021W \| YKL145W \| YGL048C |
| 22 | 10567526 | YBL023C \| YPR019W \| YGL201C \| YBR202W |
| 23 | 11412995 | YBR010W \| YJL115W \| YBR009C |
| 24 | 8530381 | YIL022W \| YJR045C \| YOR232W |
| 25 | 9321403 | YLR293C \| YER110C |
| 26 | 11369780 | YGL087C \| YDR092W |
| 27 | 7565784 | YKL145W \| YIL075C |
| 28 | 11950928 | YDR356W \| YHR172W \| YLR212C \| YNL126W |
| 29 | 10480869 | YOL090W \| YDR097C |
| 30 | 7961739 | YER171W \| YIL143C \| YLR005W \| YDR311W |
| 31 | 9671501 | YMR309C \| YOR361C \| YMR146C \| YPR041W |
| 32 | 8816473 | YOL090W \| YDR097C |
| 33 | 7688296 | YJL041W \| YGL172W \| YFR002W |
| 34 | 12882976 | YHR005C-A \| YEL020W-A |
| 35 | 8324825 | YER022W \| YER148W \| YHR041C \| YGR104C \| YBR253W |
| 36 | 12543930 | YER165W \| YPL169C |
| 37 | 8812838 | YGR005C \| YGR186W \| YPL129W \| YLR418C \| YGL043W \| YBR279W \| YPR086W |

| | | |
|---|---|---|
| 38 | 12543930 | YDL116W \| YKL057C \| YLR208W \| YJR042W \| YGL100W \| YGL092W \| YKR082W |
| 39 | 12543930 | YLR335W \| YNL189W \| YLR347C |
| 40 | 2000150 | YOR254C \| YLR378C \| YPL094C |
| 41 | 7925282 | YML105C \| YAL015C \| YPR088C \| YDL092W \| YKL122C \| YPL243W \| YPL210C |
| 42 | 15099519 | YEL056W \| YPL001W \| YLL022C |
| 43 | 9311989 | YAL021C \| YNR052C \| YGR092W |
| 44 | 8414971 | YER148W \| YPR086W |
| 45 | 14627716 | YLR080W \| YFL048C |
| 46 | 14981506 | YLR008C \| YJL104W |
| 47 | 11756442 | YNL262W \| YPR175W \| YBR278W \| YDR121W |
| 48 | 9382859 | YDR170C \| YDR238C \| YPR181C |
| 49 | 8631896 | YER171W \| YIL143C \| YDR311W \| YLR005W \| YDL108W |
| 50 | 10779558 | YNL062C \| YJL125C |
| 51 | 9755168 | YPL153C \| YDR217C |
| 52 | 8631896 | YIL021W \| YDL140C \| YOR151C \| YJL140W \| YBR154C \| YPR187W \| YDR404C \| YOR224C |
| 53 | 8628401 | YPR103W \| YER012W \| YOR362C \| YJL001W \| YFR050C \| YMR314W \| YOL038W \| YBL041W \| YML092C \| YGR135W \| YOR157C \| YGR253C \| YER094C \| YDL020C \| YBR173C \| YHR052W \| YDR069C \| YHL030W \| YOR056C \| YGL058W \| YDR177W \| YBR082C \| YDR059C \| YGR184C \| YGL048C |
| 54 | 8628401 | YGL048C \| YKL145W |
| 55 | 11741545 | YOR005C \| YGL090W |
| 56 | 12543930 | YBR017C \| YOL123W |
| 57 | 12543930 | YNL189W \| YLR347C |
| 58 | 10675336 | YOR194C \| YKL058W |
| 59 | 12446794 | YBR081C \| YGL252C \| YHR099W \| YDR176W \| YOL148C \| YDR392W \| YGR252W \| YPL254W \| YDR448W \| YDR167W \| YMR236W \| YBR198C \| YDR145W \| YGL112C |
| 60 | 7851434 | YNL098C \| YLL016W |
| 61 | 12446794 | YDR167W \| YBR081C \| YHR099W \| YDR176W \| YLR055C \| YOL148C \| YDR392W \| YGR252W \| YPL254W \| YDR448W \| YMR236W \| YBR198C \| YDR145W \| YGL112C |
| 62 | 9234719 | YHR058C \| YDL140C \| YER022W \| YHR041C \| YGR104C \| YOL051W |
| 63 | 9234719 | YLR071C \| YHR041C \| YGR104C \| YHR058C \| YDL140C \| YOR151C \| YIL021W \| YJL140W \| YBR154C \| YPR187W \| YDR404C \| YOL051W \| YNL236W \| YER022W |
| 64 | 9819445 | YOL090W \| YDR097C |
| 65 | 10487762 | YOR244W \| YHR099W |
| 66 | 9154821 | YDR448W \| YPL254W \| YDR176W \| YOL148C \| YGR252W |
| 67 | 8816488 | YKL025C \| YGL094C |
| 68 | 15014446 | YIL126W \| YGR056W \| YLR357W \| YDR303C \| YKR008W \| YPR034W \| YMR033W \| YCR052W \| YFR037C \| YML127W \| YLR321C |

| | | |
|---|---|---|
| 69 | 8181570 | YEL002C \| YMR149W |
| 70 | 7929382 | YFL036W \| YMR228W |
| 71 | 10385523 | YOR106W \| YBL050W \| YLR093C \| YGL212W \| YMR197C \| YKL196C |
| 72 | 11336698 | YGR056W \| YPR034W \| YLR357W \| YDR303C \| YKR008W \| YIL126W \| YLR321C \| YMR033W \| YHR056C |
| 73 | 11979277 | YNL139C \| YDR138W \| YML062C \| YHR167W \| YDL084W |
| 74 | 10026213 | YDR225W \| YDR448W \| YGR252W \| YBL003C \| YBR009C \| YNL030W \| YBR010W \| YNL031C \| YDR224C \| YBL002W |
| 75 | 9391047 | YLR141W \| YBL025W \| YMR270C |
| 76 | 11741545 | YNL250W \| YDR369C \| YMR224C |
| 77 | 1321337 | YLR433C \| YML057W |
| 78 | 11964151 | YML051W \| YDR009W \| YBR020W |
| 79 | 7623818 | YDR073W \| YHL025W |
| 80 | 8565072 | YDL116W \| YKL057C \| YJR042W \| YDL195W \| YLR208W |
| 81 | 10964916 | YDL017W \| YDR052C |
| 82 | 11032818 | YBR130C \| YAL029C |
| 83 | 9130700 | YNL126W \| YHR172W \| YLR212C |
| 84 | 11839796 | YPR025C \| YDR311W \| YDL108W \| YDR460W |
| 85 | 7768886 | YER171W \| YIL143C \| YLR005W \| YDR311W |
| 86 | 2832951 | YHR187W \| YBL021C |
| 87 | 12925749 | YAR033W \| YGL051W |
| 88 | 12925749 | YAR031W \| YGL053W |
| 89 | 11390369 | YGR200C \| YLR384C \| YPL086C \| YHR187W \| YPL101W \| YMR312W |
| 90 | 10069815 | YKL145W \| YDR069C |
| 91 | 9252322 | YOR254C \| YLR378C \| YPL094C \| YBR171W \| YLR292C \| YER087C-B \| YDR086C |
| 92 | 10089880 | YGL087C \| YDR092W |
| 93 | 9368761 | YMR167W \| YNL082W |
| 94 | 14634024 | YAR007C \| YNL312W \| YJL173C |
| 95 | 11858722 | YOR232W \| YDL003W |
| 96 | 11435442 | YPL086C \| YPL101W \| YHR187W \| YMR312W |
| 97 | 9420330 | YOL135C \| YOL051W \| YPR070W \| YLR071C \| YNL236W \| YER022W \| YDL005C \| YGL025C \| YOR174W \| YHR058C \| YBR193C \| YBL093C \| YHR041C \| YDR308C \| YGR104C \| YBR253W |
| 98 | 11238944 | YDR225W \| YBR010W \| YBR009C \| YDR224C |
| 99 | 11118453 | YJL006C \| YML112W |
| 100 | 7559750 | YJL041W \| YFR002W \| YGL172W \| YGR119C |
| 101 | 9584156 | YHR027C \| YOR362C \| YHR200W \| YFR004W \| YFR052W \| YIL075C \| YER021W \| YDL147W \| YDL097C \| YPR108W \| YOR261C \| YDR427W \| YKL145W \| YDL007W \| YDR394W \| YOR259C \| YOR117W \| YGL048C |
| 102 | 8657139 | YOR194C \| YKL058W |
| 103 | 8657139 | YER148W \| YPR086W \| YPL082C |
| 104 | 8657139 | YPR056W \| YDR079C-A \| YLR005W \| YDR460W \| YPL122C \| YPR025C \| YPR086W \| YDL140C \| YHR041C \| YER022W \| YGR104C \| YBR253W \| YER148W \| YCR042C \| YGR186W \| YGR005C \| YPL129W |

| | | |
|---|---|---|
| 105 | 8657139 | YGR186W \| YDL140C \| YGR005C \| YPL129W \| YKL028W \| YKR062W |
| 106 | 11500379 | YDL042C \| YDR227W |
| 107 | 8187178 | YER022W \| YGR005C \| YOL051W \| YGL048C \| YHR041C \| YGR104C \| YBR253W \| YPL129W \| YGR186W |
| 108 | 11500379 | YDL042C \| YJL076W |
| 109 | 11383511 | YGR104C \| YER022W \| YHR041C \| YPL042C \| YOL051W \| YNL236W \| YIL021W \| YPR086W |
| 110 | 9741626 | YHR027C \| YHR200W \| YIL075C \| YKL145W \| YDL007W \| YDR394W \| YOR259C \| YOR117W \| YGL048C |
| 111 | 10559961 | YOR106W \| YPL195W \| YGR261C |
| 112 | 9674426 | YBR198C \| YBR081C \| YLR055C \| YOL148C \| YDR392W \| YDR176W \| YPL254W \| YDR448W \| YGR252W \| YDR145W \| YGL112C \| YDR167W \| YMR236W |
| 113 | 9822593 | YEL020W-A \| YHR005C-A |
| 114 | 9154831 | YIL126W \| YCR052W |
| 115 | 11752412 | YHR119W \| YLR015W \| YPL138C \| YKL018W \| YDR469W |
| 116 | 14530260 | YJR068W \| YOR217W \| YNL290W \| YOL094C \| YBR087W |
| 117 | 15664196 | YKL138C-A \| YKL052C \| YGR113W \| YKR037C \| YGL061C \| YDR201W \| YKR083C \| YDR016C \| YBR233W-A \| YDR320C-A |
| 118 | 10852917 | YDR211W \| YGR083C \| YOR260W \| YLR291C \| YKR026C |
| 119 | 16183906 | YLR370C \| YJR065C \| YDL029W \| YNR035C \| YKL013C \| YIL062C |
| 120 | 11687631 | YHR119W \| YLR015W \| YAR003W \| YPL138C \| YKL018W \| YBR175W \| YDR469W |
| 121 | 10817755 | YBL052C \| YPL129W |
| 122 | 9463387 | YCR093W \| YAL021C \| YNR052C |
| 123 | 10352012 | YDR328C \| YMR094W |
| 124 | 8253836 | YOR254C \| YBR171W \| YLR292C |
| 125 | 9792654 | YJR052W \| YBR114W |
| 126 | 9858534 | YLR055C \| YDR448W \| YDR392W \| YGL112C \| YDR145W \| YBR198C |
| 127 | 11859374 | YKL054C \| YJR035W |
| 128 | 15163346 | YNL262W \| YPR175W \| YBR278W \| YDR121W |
| 129 | 11777910 | YGL112C \| YDR448W \| YDR145W \| YDR176W \| YOL148C |
| 130 | 9813094 | YLR314C \| YCR002C \| YJR076C \| YHR107C |
| 131 | 9228276 | YHR200W \| YER012W \| YGL048C \| YKL145W |
| 132 | 9724628 | YDR394W \| YER021W \| YKL145W \| YDL007W \| YOR259C \| YOR117W |
| 133 | 8995429 | YJR068W \| YOR217W \| YNL290W \| YOL094C \| YBR087W |
| 134 | 8017102 | YDR225W \| YBL003C \| YDR224C \| YBL002W \| YBR010W \| YNL031C \| YBR009C \| YNL030W |
| 135 | 10748175 | YGL225W |
| 136 | 12138208 | YGR274C \| YER148W \| YCR042C \| YMR236W \| YML098W \| YDR167W \| YPL129W \| YML015C \| YPL011C \| YMR005W \| YGL112C \| YDR145W \| YML114C \| YMR227C \| YBR198C |
| 137 | 16183906 | YIL062C \| YJR065C \| YDL029W \| YNR035C \| YKL013C \| YLR370C |
| 138 | 16207085 | YCR002C \| YLR314C \| YJR076C \| YHR107C |

| | | |
|---|---|---|
| 139 | 10684247 | YMR047C \| YAR002W \| YBL079W \| YDL088C \| YDL116W \| YDL207W \| YDR192C \| YER105C \| YER107C \| YFR002W \| YGL092W \| YGL100W \| YGL172W \| YGR119C \| YIL115C \| YJL039C \| YJL041W \| YJL061W \| YJR042W \| YKL057C \| YKL068W \| YKR082W \| YLR018C \| YML031W \| YML103C \| YMR129W \| YMR153W \| YOR098C \| YOR257W \| YER110C \| YGL016W \| YIL063C \| YLR335W \| YLR347C \| YNL189W \| YPL125W \| YPL169C \| YBR025C \| YBR194W \| YCL059C \| YDL121C \| YDL148C \| YDR190C \| YDR412W \| YEL026W \| YER006W \| YER049W \| YER126C \| YGR103W \| YLR074C \| YHR127W \| YHR052W \| YMR131C \| YMR290C \| YNR053C \| YOL145C \| YOR051C \| YOR091W \| YOR145C \| YOR206W \| YPL093W \| YPL146C \| YPL217C \| YPR144C |
| 140 | 16137930 | YOR368W \| YPL022W \| YLR288C |
| 141 | 11024051 | YOL004W \| YNL330C \| YIL084C |
| 142 | 16027163 | YNL328C \| YJL104W \| YLR008C \| YIL022W |
| 143 | 9428766 | YPL218W \| YGR191W \| YGL200C \| YLR268W \| YPR181C \| YIL109C |
| 144 | 15591051 | YOR297C \| YLR348C |
| 145 | 15723534 | YPR019W \| YGL201C \| YBR202W |
| 146 | 11432854 | YJR068W \| YOR217W \| YNL290W \| YOL094C \| YBR087W |
| 147 | 11239395 | YAL021C \| YNR052C |
| 148 | 9892641 | YOR174W \| YPR070W \| YDL005C \| YHR058C \| YOL135C \| YBR193C |
| 149 | 12820877 | YOL090W \| YDR097C |
| 150 | 11007479 | YER171W \| YLR005W \| YDR311W \| YPL122C \| YDR460W |
| 151 | 7958901 | YBL014C \| YJL025W |
| 152 | 11604499 | YOR244W \| YHR099W \| YJL081C \| YHR090C \| YDR359C \| YFL024C \| YGR002C \| YPR023C \| YFL039C \| YJR082C \| YEL018W |
| 153 | 15907469 | YER012W \| YER021W \| YFR052W \| YHR200W \| YKL145W \| YOR117W |
| 154 | 10490601 | YOR023C \| YDR448W \| YGR252W \| YDR176W \| YBL015W |
| 155 | 15799968 | YDR468C \| YOL018C \| YMR197C \| YOR036W |
| 156 | 14563679 | YDR448W \| YGR252W \| YMR223W \| YLR055C \| YDR392W |
| 157 | 10377396 | YER172C \| YKL173W \| YBR055C \| YOR308C \| YGR091W \| YDR473C \| YPR178W \| YGR075C \| YDL098C \| YER029C \| YER112W \| YPR082C \| YGR074W \| YJR022W \| YLR147C \| YDR378C \| YEL026W \| YLR275W \| YBL026W \| YER146W \| YOR159C \| YPR182W \| YFL017W-A |
| 158 | 10449419 | YER172C \| YHR165C \| YKL173W \| YBR055C \| YOR308C \| YGR091W \| YDR473C \| YPR178W \| YBR152W \| YGR075C \| YGR006W \| YDL098C \| YPR082C \| YEL026W \| YER029C \| YGR074W \| YLR275W \| YLR147C \| YOR159C \| YPR182W \| YFL017W-A \| YBL026W \| YLR438C-A \| YER112W \| YER146W \| YDR378C \| YNL147W \| YJR022W |
| 159 | 15014082 | YER173W \| YJR068W \| YNL290W \| YOL094C \| YBR087W |
| 160 | 10675611 | YKL145W \| YOR056C \| YFR052W |

| | | |
|---|---|---|
| 161 | 11804584 | YLL036C \| YML046W \| YGR013W \| YKL012W \| YDR235W \| YHR086W \| YDR240C \| YIL061C \| YBR119W \| YLR298C \| YDL087C \| YML049C \| YMR288W \| YDL030W \| YMR240C \| YJL203W \| YNL286W \| YDL043C \| YPL213W \| YOR319W \| YIR005W \| YIR009W \| YHR165C \| YER172C \| YKL173W \| YBR055C \| YOR308C \| YGR091W \| YDR473C \| YPR178W \| YBR152W \| YGR075C \| YDL098C \| YPR082C \| YEL026W \| YDR416W \| YLR117C \| YMR213W \| YFR005C \| YPL151C \| YAL032C \| YBR065C \| YDL209C \| YJR050W \| YGR129W \| YPR101W \| YBR188C \| YER029C \| YGR074W \| YLR275W \| YLR147C \| YOR159C \| YPR182W \| YFL017W-A \| YBL026W \| YLR438C-A \| YER112W \| YDR378C \| YNL147W \| YJR022W \| YER111C \| YDL056W \| YLR182W \| YGL173C \| YCR077C \| YJL124C \| YOR204W \| YAL005C \| YDL229W \| YMR116C \| YDL160C \| YHR216W \| YLR432W \| YML056C \| YAR073W \| YPR080W \| YDL014W \| YNL007C \| YJL052W \| YKL152C \| YLR016C \| YCR063W \| YDR428C \| YGR214W \| YLR441C \| YGL123W \| YNL178W \| YHR203C \| YJR123W \| YOR096W \| YBR189W \| YOR369C \| YDR064W \| YCR031C \| YDL083C \| YDR450W \| YNL302C \| YHL015W |
| 162 | 1325440 | YJL034W |
| 163 | 11952896 | YMR117C \| YGL075C |
| 164 | 8621533 | YER171W \| YIL143C \| YDR311W \| YLR005W |
| 165 | 1400380 | YGL155W \| YKL019W |
| 166 | 11904415 | YGR200C \| YPL086C \| YPL101W \| YHR187W \| YMR312W \| YLR384C |
| 167 | 12192046 | YBR088C \| YBL019W |
| 168 | 11304548 | YLR357W \| YIL126W \| YGR056W \| YDR303C \| YKR008W |
| 169 | 12390024 | YJR068W \| YOR217W \| YNL290W \| YOL094C \| YBR087W |
| 170 | 10921896 | YML105C \| YPL243W \| YPR088C \| YKL122C \| YDL092W |
| 171 | 9545323 | YOL090W \| YMR167W \| YDR097C |
| 172 | 11018040 | YLL036C \| YMR213W \| YJR050W \| YPR101W \| YBR188C |
| 173 | 12215531 | YGR274C \| YCR093W |
| 174 | 12215531 | YMR227C \| YBR198C \| YPL011C \| YDR167W |
| 175 | 1985924 | YDR404C \| YJL140W |
| 176 | 11867522 | YEL020W-A \| YHR005C-A |
| 177 | 2007593 | YHR024C \| YLR163C |
| 178 | 7479899 | YER022W \| YNL236W \| YGR104C |
| 179 | 2643110 | YFL039C \| YNL079C |
| 180 | 3888995 | YIR008C \| YNL102W \| YNL262W \| YKL045W |
| 181 | 7479899 | YOL051W \| YNL236W \| YGR104C |
| 182 | 7479899 | YER022W \| YGR104C \| YHR041C \| YBR253W |
| 183 | 6088500 | YNL088W |
| 184 | 8034743 | YFL039C \| YDR129C |
| 185 | 11389845 | YEL037C \| YOR117W \| YGL048C \| YOR259C |
| 186 | 8168496 | YJR045C \| YOR232W |
| 187 | 9195975 | YOR332W \| YBR127C \| YPR036W \| YEL051W |
| 188 | 8194532 | YLL036C |
| 189 | 7565723 | YKL139W \| YJL006C \| YML112W |
| 190 | 10921930 | YJR074W \| YLR293C |
| 191 | 9632766 | YPL240C \| YNL064C |

| | | |
|---|---|---|
| 192 | 12832395 | YBR228W \| YLR135W |
| 193 | 8159677 | YOR290C \| YBR289W \| YHL025W \| YJL176C \| YPL016W |
| 194 | 12719426 | YFR031C \| YLR086W |
| 195 | 11370741 | YMR026C \| YDR265W \| YLR191W \| YDR244W \| YGL153W |
| 196 | 11237696 | YMR167W \| YNL082W \| YAL005C |
| 197 | 9268290 | YJR052W \| YBR114W |
| 198 | 10848595 | YER012W \| YBR082C |
| 199 | 8995225 | YDL140C \| YER022W \| YBL093C |
| 200 | 10788499 | YGL070C \| YDL140C \| YOR151C \| YIL021W \| YJL140W \| YBR154C \| YPR187W \| YDR404C \| YOR224C \| YOL005C |
| 201 | 9343382 | YGR252W \| YDR176W |
| 202 | 8253836 | YBR171W \| YLR292C |
| 203 | 10873636 | YDL097C \| YHR027C \| YHR200W \| YIL075C \| YER021W \| YDL147W \| YPR108W \| YDR427W \| YFR004W \| YFR052W |
| 204 | 8421056 | YLL050C \| YFL039C |
| 205 | 8756702 | YPR176C \| YJL031C |
| 206 | 8943361 | YFL016C \| YJR045C |
| 207 | 9420330 | YGL025C \| YPR070W \| YOL051W \| YDL005C \| YOR174W \| YHR058C \| YOL135C \| YBR193C \| YLR071C \| YBL093C \| YNL236W \| YHR041C \| YER022W \| YDR308C \| YCR081W |
| 208 | 9545323 | YOL090W \| YDR097C |
| 209 | 8063853 | YIL004C \| YLR078C \| YLR268W |
| 210 | 9545323 | YMR167W \| YNL082W |
| 211 | 8063853 | YBR210W \| YGR284C |
| 212 | 9585415 | YDR356W \| YKR037C \| YPL124W \| YMR117C \| YER018C \| YDR201W \| YDL220C \| YBR109C \| YBR009C \| YNL030W \| YBL034C \| YMR129W \| YGL093W \| YDL140C \| YBL063W \| YOR373W \| YLR045C \| YHR172W \| YML085C \| YFL037W \| YOL069W \| YPL255W \| YKL042W \| YLR127C \| YNL001W \| YGL075C \| YHR076W \| YNL126W \| YFR002W \| YPR141C \| YAL047C \| YJR042W \| YER016W \| YIL144W \| YPR161C \| YAL005C \| YDL229W \| YNL225C |
| 213 | 9741626 | YDL097C \| YER021W \| YHR200W \| YDL147W \| YPR108W \| YOR261C \| YDR427W \| YFR004W \| YFR052W |
| 214 | 14614818 | YFL008W \| YDL003W \| YJL074C |
| 215 | 10949041 | YGL025C \| YOL051W \| YGL151W \| YLR071C \| YNL236W \| YER022W \| YPR070W \| YDL005C \| YOR174W \| YHR058C \| YGR104C \| YOL135C \| YBR193C \| YBL093C \| YHR041C |
| 216 | 12604615 | YOR027W \| YPL240C |
| 217 | 8942643 | YHR005C \| YOR212W |
| 218 | 12110181 | YGR103W \| YLL004W \| YGL201C |
| 219 | 11504715 | YDR092W \| YGL087C |
| 220 | 11036083 | YOR244W \| YHR099W \| YPR023C |
| 221 | 11018020 | YBR079C \| YMR309C \| YOR361C \| YPR041W \| YMR146C \| YDR429C |
| 222 | 11089979 | YHR041C \| YHR058C \| YOL051W \| YDL108W \| YKR062W \| YER022W \| YBR198C \| YER148W \| YGR005C \| YPR086W \| YIL021W |
| 223 | 7721780 | YPR052C \| YBR089C-A |
| 224 | 10377407 | YJR065C \| YDL029W \| YBR234C |

| | | |
|---|---|---|
| 225 | 9121458 | YGL115W \| YDR477W |
| 226 | 9867807 | YPL078C \| YBL099W \| YJR121W \| YDR322C-A \| YLR295C \| YPL271W \| YDL004W \| YDR377W \| YML081C-A \| YPR020W \| YBR039W \| YDR298C \| YKL016C \| Q0080 \| Q0130 \| YDL181W |
| 227 | 10352012 | YMR168C |
| 228 | 10426947 | YMR038C |
| 229 | 14500720 | YDR311W \| YER171W \| YPL122C \| YLR005W |
| 230 | 10430890 | YPL046C |
| 231 | 8557668 | YER171W \| YIL143C \| YDR311W \| YPL122C \| YLR005W \| YPR025C \| YDR460W \| YDL108W |
| 232 | 10449236 | YBL099W \| YJR121W |
| 233 | 14613943 | YJL074C \| YFL008W \| YDR174W |
| 234 | 10777588 | YGR200C \| YOR151C \| YLR384C \| YDL140C \| YJL140W \| YIL021W \| YPL086C \| YBR154C |
| 235 | 11387228 | YOR361C \| YBR079C \| YMR309C \| YMR146C \| YDR429C \| YNL244C \| YPR041W |
| 236 | 14532110 | YNR017W \| YJL143W \| YLR259C \| YIL022W \| YPL063W \| YOR232W \| YJR045C \| YNL055C \| YNL131W \| YMR203W |
| 237 | 8016655 | YOR290C \| YPL016W \| YJL176C \| YHL025W \| YBR289W |
| 238 | 12631735 | YKL203C \| YOL078W \| YNL006W \| YJR066W |
| 239 | 9677405 | YJR043C \| YJR006W |
| 240 | 9490418 | YEL037C \| YKL145W \| YER162C |
| 241 | 9660974 | YDL140C \| YOL051W \| YER022W \| YGR104C |
| 242 | 8855246 | YGR258C \| YIL143C \| YER171W \| YLR005W \| YDR311W |
| 243 | 10878005 | YNL262W \| YBR278W |
| 244 | 10878005 | YDR121W \| YNL262W |
| 245 | 10428870 | YNR017W \| YJL143W \| YIL022W \| YNL131W \| YMR203W |
| 246 | 9649439 | YNL248C \| YKL125W \| YOR341W \| YPR010C \| YOR340C |
| 247 | 11433034 | YPL167C \| YIL139C |
| 248 | 11251072 | YOR254C \| YLR378C \| YBR171W \| YPL094C \| YLR292C |
| 249 | 10713261 | YLR026C \| YPL050C \| YNL238W \| YDR483W \| YLR268W \| YBR171W \| YML115C \| YFL038C |
| 250 | 12727870 | YHL020C \| YER120W |
| 251 | 10490639 | YGL134W \| YLR258W |
| 252 | 9885573 | YMR236W \| YHR099W \| YBR198C \| YDR145W \| YGL112C \| YDR167W \| YLR055C \| YBR081C \| YDR392W \| YGR252W \| YOL148C \| YDR176W \| YDR448W \| YPL254W |
| 253 | 11598206 | YJL053W \| YJL154C |
| 254 | 11741545 | YMR224C \| YDR369C |
| 255 | 11884586 | YLR418C \| YPR086W \| YOR151C |
| 256 | 8602515 | YLR342W \| YPR165W |
| 257 | 10753924 | YNL230C \| YPL046C |
| 258 | 9303317 | YLR115W \| YDR301W \| YLR277C \| YAL043C \| YKR002W \| YJR093C |

## Supplementary Table 3. SI/HTP publications

| PubmedID | HTP/SI | number of interactions |
|---|---|---|
| 14759368 | SI | 890 |
| 12150911 | SI | 509 |
| 11804584 | SI | 568 |
| 12052880 | SI | 483 |
| 10900456 | SI | 268 |
| 11743162 | SI | 250 |
| 15766533 | SI | 223 |
| 11489916 | SI | 206 |
| 11884590 | SI | 200 |
| 14690591 | SI | 169 |
| 15590687 | SI | 158 |
| 12374754 | SI | 148 |
| 10449419 | SI | 147 |
| 15879519 | SI | 127 |
| 11387327 | SI | 125 |
| 14729968 | SI | 122 |
| 10377396 | SI | 119 |
| 12556496 | SI | 110 |
| 12242279 | SI | 90 |
| 12819204 | SI | 88 |
| 11029046 | SI | 85 |
| 15798213 | SI | 84 |
| 12808088 | SI | 80 |
| 15045029 | SI | 75 |
| 15200949 | SI | 74 |
| 12543930 | SI | 66 |
| 10684247 | SI | 65 |
| 15226434 | SI | 63 |
| 12837249 | SI | 55 |
| 11595789 | SI | 55 |
| **Total** | **SI** | **5702** |
| 14574415 | HTP | 369 |
| 11087867 | HTP | 177 |
| 9207794 | HTP | 169 |
| 15292183 | HTP | 117 |
| 11474067 | HTP | 38 |
| **Total** | **HTP** | **870** |

## Supplementary Table 4. Post-translational modifications associated with interactions

| GeneA | GeneB | Experimental System | Modification | PubMed ID |
|---|---|---|---|---|
| YFR028C | YJR053W | Reconstituted Complex | Dephosphorylation | 11970961 |
| YFR028C | YLR079W | Reconstituted Complex | Dephosphorylation | 9885559 |
| YER133W | YLR263W | Reconstituted Complex | Dephosphorylation | 10786836 |
| YOR208W | YHR030C | Reconstituted Complex | Dephosphorylation | 10523653 |
| YER075C | YBL016W | Reconstituted Complex | Dephosphorylation | 10557209 |
| YER075C | YHR030C | Reconstituted Complex | Dephosphorylation | 10523653 |
| YPR161C | YDL140C | Reconstituted Complex | Phosphorylation | 11390638 |
| YFL029C | YBR160W | Reconstituted Complex | Phosphorylation | 8752211 |
| YBR160W | YDR113C | Reconstituted Complex | Phosphorylation | 12050115 |
| YBR160W | YJL194W | Reconstituted Complex | Phosphorylation | 8930895 |
| YBR160W | YPL256C | Reconstituted Complex | Phosphorylation | 8599119 |
| YBR160W | YJL157C | Reconstituted Complex | Phosphorylation | 8395009 |
| YMR001C | YDR052C | Reconstituted Complex | Phosphorylation | 8943332 |
| YDL017W | YDR052C | Reconstituted Complex | Phosphorylation | 8943332 |
| YBR274W | YDR113C | Reconstituted Complex | Phosphorylation | 11390356 |
| YBR274W | YDR113C | Reconstituted Complex | Phosphorylation | 10550056 |
| YBL016W | YLR310C | Reconstituted Complex | Phosphorylation | 12867033 |
| YBL016W | YDR388W | Reconstituted Complex | Phosphorylation | 12857883 |
| YLR113W | YNL167C | Reconstituted Complex | Phosphorylation | 12086627 |
| YBR136W | YDR499W | Reconstituted Complex | Phosphorylation | 10950868 |
| YDL028C | YIL106W | Reconstituted Complex | Phosphorylation | 9436989 |
| YPL153C | YDR217C | Reconstituted Complex | Phosphorylation | 12917350 |
| YPL153C | YDL101C | Reconstituted Complex | Phosphorylation | 12556502 |
| YNR031C | YJL128C | Reconstituted Complex | Phosphorylation | 12853477 |
| YGR040W | YHR084W | Reconstituted Complex | Phosphorylation | 9393860 |
| YFR028C | YBR156C | Affinity Capture-Western | Dephosphorylation | 14605209 |
| YNL289W | YHR042W | Reconstituted Complex | Phosphorylation | 15082539 |
| YNL289W | YOR032C | Reconstituted Complex | Phosphorylation | 15082539 |
| YNL289W | YJR072C | Reconstituted Complex | Phosphorylation | 15082539 |
| YPL256C | YHL007C | Affinity Capture-MS | Phosphorylation | 10359756 |
| YDR432W | YER133W | Affinity Capture-Western | Dephosphorylation | 14759366 |
| YDR283C | YDR283C | Affinity Capture-Western | Phosphorylation | 8798780 |
| YDL008W | YBR082C | Reconstituted Complex | Ubiquitination | 10888670 |
| YBR010W | YDR440W | Affinity Capture-Western | Methylation | 12080090 |
| YNL031C | YDR440W | Affinity Capture-Western | Methylation | 12080090 |
| YOL001W | YFR034C | Affinity Capture-Western | Phosphorylation | 15057567 |
| YOL001W | YGR233C | Affinity Capture-Western | Phosphorylation | 15057567 |
| YPL031C | YFR034C | Affinity Capture-Western | Phosphorylation | 15057567 |
| YPL031C | YGR233C | Affinity Capture-Western | Phosphorylation | 15057567 |
| YPL031C | YOL001W | Affinity Capture-Western | Phosphorylation | 15057567 |
| YPL031C | YOL001W | Reconstituted Complex | Phosphorylation | 15057567 |
| YOL001W | YFR034C | Reconstituted Complex | Phosphorylation | 14659740 |
| YPL031C | YFR034C | Reconstituted Complex | Phosphorylation | 14659740 |
| YIL035C | YKL112W | Reconstituted Complex | Phosphorylation | 7608180 |
| YGL134W | YLR258W | Reconstituted Complex | Phosphorylation | 10490639 |
| YLR258W | YGL134W | Protein-peptide | Phosphorylation | 10490639 |
| YLR258W | YPL031C | Protein-peptide | Phosphorylation | 10490639 |
| YDL108W | YOR174W | Affinity Capture-Western | Phosphorylation | 15126497 |
| YPL042C | YDL005C | Reconstituted Complex | Phosphorylation | 14988503 |
| YPR066W | YDR139C | Reconstituted Complex | Nedd8(Rub1)ylation | 9545234 |

| | | | | |
|---|---|---|---|---|
| YLR306W | YDR139C | Reconstituted Complex | Nedd8(Rub1)ylation | 9545234 |
| YNL222W | YDL140C | Protein-peptide | Dephosphorylation | 15125841 |
| YKL048C | YKL048C | Affinity Capture-Western | Phosphorylation | 9180279 |
| YJR094C | YMR139W | Affinity Capture-Western | Phosphorylation | 15282298 |
| YBL016W | YDR480W | Reconstituted Complex | Phosphorylation | 9094309 |
| YBL016W | YPL049C | Reconstituted Complex | Phosphorylation | 9094309 |
| YDL159W | YBL016W | Reconstituted Complex | Phosphorylation | 15456892 |
| YBR160W | YNL068C | Reconstituted Complex | Phosphorylation | 15509804 |
| YBL016W | YDR103W | Reconstituted Complex | Phosphorylation | 15322134 |
| YMR277W | YDL140C | Protein-peptide | Dephosphorylation | 15563457 |
| YLR362W | YBL016W | Affinity Capture-Western | Phosphorylation | 15713635 |
| YDR138W | YER125W | Reconstituted Complex | Ubiquitination | 15713680 |
| YBR136W | YPR010C | Reconstituted Complex | Phosphorylation | 14642562 |
| YGR003W | YDR139C | Biochemical Activity | Nedd8(Rub1)ylation | 14519104 |
| YJL047C | YDR139C | Biochemical Activity | Nedd8(Rub1)ylation | 14519104 |
| YDL132W | YDR139C | Biochemical Activity | Nedd8(Rub1)ylation | 12446563 |
| YHR111W | YIL008W | Biochemical Activity | Nedd8(Rub1)ylation | 10713047 |
| YFR028C | YLR182W | Biochemical Activity | Dephosphorylation | 14993267 |
| YFR028C | YAL024C | Biochemical Activity | Dephosphorylation | 12432084 |
| YFR028C | YJR053W | Biochemical Activity | Dephosphorylation | 11970961 |
| YFR028C | YAR019C | Biochemical Activity | Dephosphorylation | 11267871 |
| YFR028C | YGL003C | Biochemical Activity | Dephosphorylation | 10074450 |
| YFR028C | YLR079W | Biochemical Activity | Dephosphorylation | 9885559 |
| YFR028C | YDR146C | Biochemical Activity | Dephosphorylation | 9885559 |
| YLR433C | YDR184C | Biochemical Activity | Dephosphorylation | 10102375 |
| YER133W | YLR263W | Biochemical Activity | Dephosphorylation | 10786836 |
| YNL053W | YHR030C | Biochemical Activity | Dephosphorylation | 11923319 |
| YNL053W | YHR030C | Biochemical Activity | Dephosphorylation | 10625705 |
| YNL053W | YBL016W | Biochemical Activity | Dephosphorylation | 8306972 |
| YJR017C | YDL140C | Biochemical Activity | Dephosphorylation | 11904169 |
| YDL006W | YLR113W | Biochemical Activity | Dephosphorylation | 11113180 |
| YER089C | YLR113W | Biochemical Activity | Dephosphorylation | 12477803 |
| YER089C | YHR079C | Biochemical Activity | Dephosphorylation | 9528768 |
| YDL230W | YML074C | Biochemical Activity | Dephosphorylation | 7559654 |
| YOR208W | YLR113W | Biochemical Activity | Dephosphorylation | 12477803 |
| YOR208W | YHR030C | Biochemical Activity | Dephosphorylation | 11923319 |
| YOR208W | YBR160W | Biochemical Activity | Dephosphorylation | 10580002 |
| YOR208W | YHR030C | Biochemical Activity | Dephosphorylation | 10523653 |
| YER075C | YHR030C | Biochemical Activity | Dephosphorylation | 11923319 |
| YER075C | YBR160W | Biochemical Activity | Dephosphorylation | 10580002 |
| YER075C | YBL016W | Biochemical Activity | Dephosphorylation | 10557209 |
| YER075C | YHR030C | Biochemical Activity | Dephosphorylation | 10523653 |
| YER075C | YBL016W | Biochemical Activity | Dephosphorylation | 9224718 |
| YIL113W | YHR030C | Biochemical Activity | Dephosphorylation | 11923319 |
| YDL047W | YDR468C | Biochemical Activity | Dephosphorylation | 12006655 |
| YDL047W | YOL018C | Biochemical Activity | Dephosphorylation | 12006655 |
| YIL113W | YHR030C | Biochemical Activity | Dephosphorylation | 12220658 |
| YER151C | YDL159W | Biochemical Activity | Deubiquitination | 11864977 |
| YJL137C | YJL137C | Biochemical Activity | Glucosylation | 8900126 |
| YBR034C | YHR089C | Biochemical Activity | Methylation | 12756332 |
| YBR034C | YOL123W | Biochemical Activity | Methylation | 12756332 |
| YBR034C | YGL122C | Biochemical Activity | Methylation | 12756332 |
| YBR034C | YDL014W | Biochemical Activity | Methylation | 12756332 |
| YBR034C | YDR432W | Biochemical Activity | Methylation | 12756332 |
| YBR034C | YGR159C | Biochemical Activity | Methylation | 12756332 |

| | | | | |
|---|---|---|---|---|
| YBR034C | YDR432W | Biochemical Activity | Methylation | 8668183 |
| YBR133C | YDR225W | Biochemical Activity | Methylation | 12925763 |
| YGL058W | YBR010W | Biochemical Activity | Methylation | 12077605 |
| YGL058W | YBR010W | Biochemical Activity | Methylation | 12070136 |
| YGR188C | YOR026W | Biochemical Activity | Phosphorylation | 7969164 |
| YPR161C | YDL140C | Biochemical Activity | Phosphorylation | 12972617 |
| YPR161C | YDL140C | Biochemical Activity | Phosphorylation | 11390638 |
| YFL029C | YBR160W | Biochemical Activity | Phosphorylation | 10793138 |
| YFL029C | YDL108W | Biochemical Activity | Phosphorylation | 10373527 |
| YFL029C | YDL108W | Biochemical Activity | Phosphorylation | 9774652 |
| YFL029C | YBR160W | Biochemical Activity | Phosphorylation | 9393434 |
| YFL029C | YBR160W | Biochemical Activity | Phosphorylation | 8752211 |
| YFL029C | YBR160W | Biochemical Activity | Phosphorylation | 8752210 |
| YAR019C | YGR092W | Biochemical Activity | Phosphorylation | 11404483 |
| YAR019C | YIL106W | Biochemical Activity | Phosphorylation | 11404483 |
| YBR160W | YLR182W | Biochemical Activity | Phosphorylation | 14993267 |
| YBR160W | YOR372C | Biochemical Activity | Phosphorylation | 14521842 |
| YBR160W | YOR372C | Biochemical Activity | Phosphorylation | 12865300 |
| YBR160W | YNL309W | Biochemical Activity | Phosphorylation | 12832490 |
| YBR160W | YKL185W | Biochemical Activity | Phosphorylation | 12695666 |
| YBR160W | YPL269W | Biochemical Activity | Phosphorylation | 12600318 |
| YBR160W | YPL269W | Biochemical Activity | Phosphorylation | 12554645 |
| YBR160W | YAL024C | Biochemical Activity | Phosphorylation | 12432084 |
| YBR160W | YLR314C | Biochemical Activity | Phosphorylation | 12429908 |
| YBR160W | YJL187C | Biochemical Activity | Phosphorylation | 12388757 |
| YBR160W | YDR113C | Biochemical Activity | Phosphorylation | 12050115 |
| YBR160W | YDR113C | Reconstituted Complex | Phosphorylation | 12050115 |
| YBR160W | YHR118C | Biochemical Activity | Phosphorylation | 11572976 |
| YBR160W | YDL106C | Biochemical Activity | Phosphorylation | 10884387 |
| YBR160W | YLR102C | Biochemical Activity | Phosphorylation | 10871279 |
| YBR160W | YKL022C | Biochemical Activity | Phosphorylation | 10871279 |
| YBR160W | YHR166C | Biochemical Activity | Phosphorylation | 10871279 |
| YBR160W | YBL084C | Biochemical Activity | Phosphorylation | 10871279 |
| YBR160W | YNL309W | Biochemical Activity | Phosphorylation | 10409718 |
| YBR160W | YGL003C | Biochemical Activity | Phosphorylation | 10074450 |
| YBR160W | YHL007C | Biochemical Activity | Phosphorylation | 9774429 |
| YBR160W | YDR309C | Biochemical Activity | Phosphorylation | 9736614 |
| YBR160W | YJL157C | Biochemical Activity | Phosphorylation | 9367986 |
| YBR160W | YLR079W | Biochemical Activity | Phosphorylation | 9346238 |
| YBR160W | YJL194W | Biochemical Activity | Phosphorylation | 8930895 |
| YBR160W | YPL256C | Biochemical Activity | Phosphorylation | 8599119 |
| YBR160W | YJL157C | Biochemical Activity | Phosphorylation | 8500168 |
| YBR160W | YER111C | Biochemical Activity | Phosphorylation | 8402888 |
| YBR160W | YJL157C | Biochemical Activity | Phosphorylation | 8395009 |
| YBR160W | YAL040C | Biochemical Activity | Phosphorylation | 7823941 |
| YBR160W | YAL040C | Biochemical Activity | Phosphorylation | 1316273 |
| YMR001C | YJR053W | Biochemical Activity | Phosphorylation | 12637549 |
| YMR001C | YFR028C | Biochemical Activity | Phosphorylation | 11960554 |
| YMR001C | YJL076W | Biochemical Activity | Phosphorylation | 11960554 |
| YMR001C | YJR053W | Biochemical Activity | Phosphorylation | 11733064 |
| YMR001C | YDL003W | Biochemical Activity | Phosphorylation | 11371343 |
| YMR001C | YLR102C | Biochemical Activity | Phosphorylation | 10871279 |
| YMR001C | YKL022C | Biochemical Activity | Phosphorylation | 10871279 |
| YMR001C | YNL102W | Biochemical Activity | Phosphorylation | 10871279 |
| YMR001C | YBL084C | Biochemical Activity | Phosphorylation | 10871279 |

| | | | | |
|---|---|---|---|---|
| YMR001C | YDR052C | Biochemical Activity | Phosphorylation | 8943332 |
| YDL017W | YBL023C | Biochemical Activity | Phosphorylation | 9407029 |
| YDL017W | YEL032W | Biochemical Activity | Phosphorylation | 9407029 |
| YDL017W | YPR019W | Biochemical Activity | Phosphorylation | 9407029 |
| YDL017W | YGL201C | Biochemical Activity | Phosphorylation | 9407029 |
| YDL017W | YDR052C | Biochemical Activity | Phosphorylation | 8943332 |
| YBR274W | YDR113C | Biochemical Activity | Phosphorylation | 11390356 |
| YBR274W | YDR113C | Biochemical Activity | Phosphorylation | 10550056 |
| YIL035C | YIL035C | Biochemical Activity | Phosphorylation | 12748292 |
| YIL035C | YGL035C | Biochemical Activity | Phosphorylation | 12748292 |
| YIL035C | YNL189W | Biochemical Activity | Phosphorylation | 9258433 |
| YOR061W | YDR168W | Biochemical Activity | Phosphorylation | 12435747 |
| YOR061W | YER148W | Biochemical Activity | Phosphorylation | 11551505 |
| YNL298W | YLR362W | Biochemical Activity | Phosphorylation | 12588977 |
| YNL298W | YAL041W | Biochemical Activity | Phosphorylation | 11113154 |
| YNL298W | YKL129C | Biochemical Activity | Phosphorylation | 9388196 |
| YLR248W | YLR248W | Biochemical Activity | Phosphorylation | 8939941 |
| YKL139W | YDL140C | Biochemical Activity | Phosphorylation | 12972617 |
| YDL101C | YML058W | Biochemical Activity | Phosphorylation | 14684746 |
| YDL101C | YML058W | Biochemical Activity | Phosphorylation | 11904430 |
| YBL016W | YLR310C | Biochemical Activity | Phosphorylation | 12867033 |
| YBL016W | YDR388W | Biochemical Activity | Phosphorylation | 12857883 |
| YBL016W | YJL157C | Biochemical Activity | Phosphorylation | 11525741 |
| YBL016W | YJL157C | Biochemical Activity | Phosphorylation | 8500168 |
| YBL016W | YJL157C | Biochemical Activity | Phosphorylation | 8334305 |
| YBL016W | YHR084W | Biochemical Activity | Phosphorylation | 8334305 |
| YBL016W | YDR103W | Biochemical Activity | Phosphorylation | 8314085 |
| YBL016W | YBL016W | Biochemical Activity | Phosphorylation | 8049522 |
| YDR507C | YDL225W | Biochemical Activity | Phosphorylation | 12058072 |
| YLR113W | YMR172W | Biochemical Activity | Phosphorylation | 12743037 |
| YLR113W | YBR182C | Biochemical Activity | Phosphorylation | 12482976 |
| YLR113W | YLR248W | Biochemical Activity | Phosphorylation | 11344302 |
| YLR113W | YNL167C | Biochemical Activity | Phosphorylation | 11230135 |
| YLR113W | YLR248W | Biochemical Activity | Phosphorylation | 10805732 |
| YPL204W | YNL027W | Biochemical Activity | Phosphorylation | 14597664 |
| YPL204W | YLR182W | Biochemical Activity | Phosphorylation | 9012827 |
| YKL101W | YBR133C | Biochemical Activity | Phosphorylation | 11408575 |
| YJL106W | YHR124W | Biochemical Activity | Phosphorylation | 14680970 |
| YJL106W | YNL312W | Biochemical Activity | Phosphorylation | 14634024 |
| YJL106W | YJL106W | Biochemical Activity | Phosphorylation | 14612412 |
| YJL106W | YHR124W | Biochemical Activity | Phosphorylation | 14612412 |
| YJL106W | YHR124W | Biochemical Activity | Phosphorylation | 12783856 |
| YJL106W | YHR124W | Biochemical Activity | Phosphorylation | 12242283 |
| YJL106W | YJR094C | Biochemical Activity | Phosphorylation | 11884593 |
| YPL209C | YGR113W | Biochemical Activity | Phosphorylation | 11724818 |
| YPL209C | YBR156C | Biochemical Activity | Phosphorylation | 11724818 |
| YPL209C | YBR010W | Biochemical Activity | Phosphorylation | 10975519 |
| YPL209C | YGR140W | Biochemical Activity | Phosphorylation | 10072382 |
| YHR079C | YHR079C | Biochemical Activity | Phosphorylation | 14564015 |
| YDL108W | YOR174W | Biochemical Activity | Phosphorylation | 14749387 |
| YDL108W | YLR071C | Biochemical Activity | Phosphorylation | 14749387 |
| YDL108W | YDL140C | Biochemical Activity | Phosphorylation | 12972617 |
| YDL108W | YDL140C | Biochemical Activity | Phosphorylation | 10594013 |
| YDL108W | YDL140C | Biochemical Activity | Phosphorylation | 9702190 |
| YDL108W | YDL140C | Biochemical Activity | Phosphorylation | 7760796 |

| | | | | |
|---|---|---|---|---|
| YGR040W | YLR310C | Biochemical Activity | Phosphorylation | 12867033 |
| YGR040W | YPL049C | Biochemical Activity | Phosphorylation | 8918885 |
| YNL307C | YNL307C | Biochemical Activity | Phosphorylation | 11877433 |
| YNL307C | YKL101W | Biochemical Activity | Phosphorylation | 11230131 |
| YNL307C | YAL038W | Biochemical Activity | Phosphorylation | 9209064 |
| YNL307C | YGR140W | Biochemical Activity | Phosphorylation | 7854321 |
| YBR136W | YAR007C | Biochemical Activity | Phosphorylation | 14642562 |
| YBR136W | YNL312W | Biochemical Activity | Phosphorylation | 14642562 |
| YBR136W | YDL101C | Biochemical Activity | Phosphorylation | 12967660 |
| YBR136W | YAR007C | Biochemical Activity | Phosphorylation | 12967660 |
| YBR136W | YNL312W | Biochemical Activity | Phosphorylation | 12967660 |
| YBR136W | YDR499W | Biochemical Activity | Phosphorylation | 10950868 |
| YHR058C | YDL140C | Biochemical Activity | Phosphorylation | 12972617 |
| YOR351C | YIL072W | Biochemical Activity | Phosphorylation | 9832507 |
| YOR351C | YLR263W | Biochemical Activity | Phosphorylation | 9832507 |
| YHR030C | YLR442C | Biochemical Activity | Phosphorylation | 12640455 |
| YHR030C | YPL089C | Biochemical Activity | Phosphorylation | 9111331 |
| YHR030C | YER111C | Biochemical Activity | Phosphorylation | 9065400 |
| YHR030C | YLR182W | Biochemical Activity | Phosphorylation | 9065400 |
| YDL028C | YKL042W | Biochemical Activity | Phosphorylation | 11827982 |
| YDL028C | YDR356W | Biochemical Activity | Phosphorylation | 11278681 |
| YDL028C | YIL106W | Biochemical Activity | Phosphorylation | 9436989 |
| YDL028C | YDL028C | Biochemical Activity | Phosphorylation | 9436989 |
| YPL031C | YMR311C | Biochemical Activity | Phosphorylation | 12407105 |
| YPL031C | YFR034C | Biochemical Activity | Phosphorylation | 12174300 |
| YPL031C | YEL009C | Biochemical Activity | Phosphorylation | 12101234 |
| YPL031C | YJL084C | Biochemical Activity | Phosphorylation | 12098764 |
| YPL031C | YEL009C | Biochemical Activity | Phosphorylation | 10712509 |
| YPL031C | YDR146C | Biochemical Activity | Phosphorylation | 10692159 |
| YPL031C | YDR388W | Biochemical Activity | Phosphorylation | 9843683 |
| YPL031C | YLR079W | Biochemical Activity | Phosphorylation | 9725902 |
| YPL031C | YLR258W | Biochemical Activity | Phosphorylation | 9584169 |
| YPL031C | YFR034C | Biochemical Activity | Phosphorylation | 9584169 |
| YPL031C | YFR034C | Biochemical Activity | Phosphorylation | 8539622 |
| YPL031C | YFR034C | Biochemical Activity | Phosphorylation | 8108735 |
| YPL031C | YFR034C | Biochemical Activity | Phosphorylation | 7973731 |
| YBL105C | YBL039C | Biochemical Activity | Phosphorylation | 12709422 |
| YBL105C | YBL039C | Biochemical Activity | Phosphorylation | 8626655 |
| YBL105C | YJL095W | Biochemical Activity | Phosphorylation | 7874200 |
| YDR490C | YPL004C | Biochemical Activity | Phosphorylation | 15016821 |
| YDR490C | YGR086C | Biochemical Activity | Phosphorylation | 15016821 |
| YDR490C | YKL126W | Biochemical Activity | Phosphorylation | 10074427 |
| YOL100W | YPL004C | Biochemical Activity | Phosphorylation | 15016821 |
| YOL100W | YGR086C | Biochemical Activity | Phosphorylation | 15016821 |
| YOL100W | YBL105C | Biochemical Activity | Phosphorylation | 10567559 |
| YIL095W | YOR329C | Biochemical Activity | Phosphorylation | 13679512 |
| YIL095W | YBL007C | Biochemical Activity | Phosphorylation | 11739778 |
| YIL095W | YIR006C | Biochemical Activity | Phosphorylation | 9885245 |
| YER171W | YBR029C | Biochemical Activity | Phosphorylation | 9857181 |
| YER171W | YBR274W | Biochemical Activity | Phosphorylation | 9857181 |
| YPL153C | YPL153C | Biochemical Activity | Phosphorylation | 12917350 |
| YPL153C | YDR217C | Biochemical Activity | Phosphorylation | 12917350 |
| YPL153C | YLR182W | Biochemical Activity | Phosphorylation | 12724400 |
| YPL153C | YDL101C | Biochemical Activity | Phosphorylation | 12556502 |
| YPL153C | YLR182W | Biochemical Activity | Phosphorylation | 9367985 |

| | | | | |
|---|---|---|---|---|
| YMR139W | YDR207C | Biochemical Activity | Phosphorylation | 9372955 |
| YMR139W | YJR094C | Biochemical Activity | Phosphorylation | 7969131 |
| YFL033C | YFL033C | Biochemical Activity | Phosphorylation | 9744870 |
| YOR119C | YOR119C | Biochemical Activity | Phosphorylation | 11972772 |
| YIL113W | YHR030C | Biochemical Activity | Dephosphorylation | 11923319 |
| YHR206W | YIL147C | Biochemical Activity | Phosphorylation | 9843501 |
| YHR206W | YDL235C | Biochemical Activity | Phosphorylation | 9843501 |
| YMR216C | YDR432W | Biochemical Activity | Phosphorylation | 11233987 |
| YMR216C | YDR432W | Biochemical Activity | Phosphorylation | 10952997 |
| YMR216C | YDR432W | Biochemical Activity | Phosphorylation | 10318902 |
| YDR477W | YER028C | Biochemical Activity | Phosphorylation | 14993292 |
| YDR477W | YGL073W | Biochemical Activity | Phosphorylation | 14612437 |
| YDR477W | YGL035C | Biochemical Activity | Phosphorylation | 12684376 |
| YDR477W | YGL035C | Biochemical Activity | Phosphorylation | 12393914 |
| YDR477W | YER040W | Biochemical Activity | Phosphorylation | 11809814 |
| YDR477W | YBR010W | Biochemical Activity | Phosphorylation | 11498592 |
| YDR477W | YGL035C | Biochemical Activity | Phosphorylation | 10403407 |
| YPL042C | YLR399C | Biochemical Activity | Phosphorylation | 14749387 |
| YPL042C | YCR042C | Biochemical Activity | Phosphorylation | 14749387 |
| YPL042C | YDL140C | Biochemical Activity | Phosphorylation | 12972617 |
| YPL042C | YHR084W | Biochemical Activity | Phosphorylation | 12520306 |
| YPL042C | YEL009C | Biochemical Activity | Phosphorylation | 11331604 |
| YPL042C | YMR037C | Biochemical Activity | Phosphorylation | 11331604 |
| YPL042C | YDL140C | Biochemical Activity | Phosphorylation | 10594013 |
| YPL042C | YDL140C | Biochemical Activity | Phosphorylation | 9702190 |
| YLR006C | YNR031C | Biochemical Activity | Phosphorylation | 9482735 |
| YNR031C | YJL128C | Biochemical Activity | Phosphorylation | 9482735 |
| YNR031C | YNR031C | Biochemical Activity | Phosphorylation | 9482735 |
| YNR031C | YJL128C | Biochemical Activity | Phosphorylation | 9180081 |
| YLR362W | YCL032W | Biochemical Activity | Phosphorylation | 10397774 |
| YLR362W | YJL128C | Biochemical Activity | Phosphorylation | 9180081 |
| YLR362W | YDL159W | Biochemical Activity | Phosphorylation | 8159759 |
| YHL007C | YLR362W | Biochemical Activity | Phosphorylation | 12588977 |
| YHL007C | YLR362W | Biochemical Activity | Phosphorylation | 10837245 |
| YHL007C | YKL129C | Biochemical Activity | Phosphorylation | 9388196 |
| YHL007C | YLR362W | Biochemical Activity | Phosphorylation | 7608157 |
| YDL159W | YBL016W | Biochemical Activity | Phosphorylation | 8384702 |
| YDL159W | YBL016W | Biochemical Activity | Phosphorylation | 8159759 |
| YBL088C | YDL101C | Biochemical Activity | Phosphorylation | 12967660 |
| YBL088C | YAR007C | Biochemical Activity | Phosphorylation | 12967660 |
| YBL088C | YNL312W | Biochemical Activity | Phosphorylation | 12967660 |
| YBL088C | YDR369C | Biochemical Activity | Phosphorylation | 12967660 |
| YKL203C | YMR028W | Biochemical Activity | Phosphorylation | 10329624 |
| YGL179C | YGL035C | Biochemical Activity | Phosphorylation | 12748292 |
| YGL179C | YGL179C | Biochemical Activity | Phosphorylation | 12748292 |
| YJL164C | YAL038W | Biochemical Activity | Phosphorylation | 12063246 |
| YJL164C | YOR347C | Biochemical Activity | Phosphorylation | 12063246 |
| YJL164C | YDR468C | Biochemical Activity | Phosphorylation | 12006655 |
| YJL164C | YOL018C | Biochemical Activity | Phosphorylation | 12006655 |
| YJL164C | YAL038W | Biochemical Activity | Phosphorylation | 11877433 |
| YJL164C | YPL232W | Biochemical Activity | Phosphorylation | 11157748 |
| YPL203W | YOR140W | Biochemical Activity | Phosphorylation | 12024012 |
| YPL203W | YKL166C | Biochemical Activity | Phosphorylation | 12024012 |
| YJL141C | YNR052C | Biochemical Activity | Phosphorylation | 11358866 |
| YHR135C | YDL140C | Biochemical Activity | Phosphorylation | 10594013 |

| | | | | |
|---|---|---|---|---|
| YER129W | YDR477W | Biochemical Activity | Phosphorylation | 12748292 |
| YER129W | YER129W | Biochemical Activity | Phosphorylation | 9341678 |
| YKL067W | YKL067W | Biochemical Activity | Phosphorylation | 12472466 |
| YDL235C | YIL147C | Biochemical Activity | Phosphorylation | 9882653 |
| YDL235C | YLR006C | Biochemical Activity | Phosphorylation | 9882653 |
| YFR027W | YFR027W | Biochemical Activity | Acetylation | 11864574 |
| YFR027W | YMR076C | Biochemical Activity | Acetylation | 11864574 |
| YFR027W | YDL003W | Biochemical Activity | Acetylation | 11864574 |
| YFR027W | YIL026C | Biochemical Activity | Acetylation | 11864574 |
| YOR156C | YLR314C | Biochemical Activity | Sumoylation | 12761287 |
| YJR076C | YDR510W | Biochemical Activity | Sumoylation | 10579719 |
| YLR314C | YDR510W | Biochemical Activity | Sumoylation | 10579719 |
| YDL225W | YDR510W | Biochemical Activity | Sumoylation | 10579719 |
| YDR054C | YJL157C | Biochemical Activity | Ubiquitination | 11080155 |
| YDR054C | YJL194W | Biochemical Activity | Ubiquitination | 10512865 |
| YDR054C | YMR199W | Biochemical Activity | Ubiquitination | 10213692 |
| YDR054C | YJL187C | Biochemical Activity | Ubiquitination | 9716410 |
| YDR054C | YLR079W | Biochemical Activity | Ubiquitination | 9346239 |
| YDR054C | YLR079W | Biochemical Activity | Ubiquitination | 9346238 |
| YDR054C | YLR079W | Biochemical Activity | Ubiquitination | 9334303 |
| YDR054C | YDR054C | Biochemical Activity | Ubiquitination | 8383676 |
| YDR054C | YPL256C | Biochemical Activity | Ubiquitination | 7835341 |
| YDR054C | YGR140W | Biochemical Activity | Ubiquitination | 7651401 |
| YOL013C | YOL013C | Biochemical Activity | Ubiquitination | 11146622 |
| YGL058W | YDR224C | Biochemical Activity | Ubiquitination | 10642555 |
| YGL058W | YEL009C | Biochemical Activity | Ubiquitination | 7813440 |
| YHR088W | YDL140C | Biochemical Activity | Ubiquitination | 10490634 |
| YER125W | YDL140C | Biochemical Activity | Ubiquitination | 9108033 |
| YER151C | YPR181C | Biochemical Activity | Deubiquitination | 12778054 |
| YBL016W | YPL049C | Biochemical Activity | Phosphorylation | 11525741 |
| YBL016W | YDR480W | Biochemical Activity | Phosphorylation | 11525741 |
| YBL016W | YHR084W | Biochemical Activity | Phosphorylation | 11525741 |
| YGR040W | YPL049C | Biochemical Activity | Phosphorylation | 11525741 |
| YGR040W | YDR480W | Biochemical Activity | Phosphorylation | 11525741 |
| YGR040W | YHR084W | Biochemical Activity | Phosphorylation | 11525741 |
| YBL016W | YNL271C | Biochemical Activity | Phosphorylation | 15067022 |
| YMR069W | YBR009C | Biochemical Activity | Acetylation | 12915400 |
| YNL289W | YHR042W | Biochemical Activity | Phosphorylation | 15082539 |
| YNL289W | YOR032C | Biochemical Activity | Phosphorylation | 15082539 |
| YNL289W | YJR072C | Biochemical Activity | Phosphorylation | 15082539 |
| YHR135C | YCL032W | Biochemical Activity | Phosphorylation | 14555477 |
| YDR507C | YCL032W | Biochemical Activity | Phosphorylation | 14555477 |
| YMR139W | YCL032W | Biochemical Activity | Phosphorylation | 14555477 |
| YBL016W | YDL159W | Biochemical Activity | Phosphorylation | 8455599 |
| YPL042C | YPL248C | Biochemical Activity | Phosphorylation | 10360183 |
| YFR028C | YAR019C | Biochemical Activity | Dephosphorylation | 10837230 |
| YFR028C | YAR019C | Biochemical Activity | Dephosphorylation | 10744974 |
| YGR252W | YBR010W | Biochemical Activity | Acetylation | 8805705 |
| YGR252W | YBR009C | Biochemical Activity | Acetylation | 8805705 |
| YNL298W | YLR314C | Biochemical Activity | Phosphorylation | 14993234 |
| YNL298W | YCR002C | Biochemical Activity | Phosphorylation | 14993234 |
| YNL298W | YJR076C | Biochemical Activity | Phosphorylation | 14993234 |
| YNL298W | YHR107C | Biochemical Activity | Phosphorylation | 14993234 |
| YPR119W | YJR092W | Biochemical Activity | Phosphorylation | 14574415 |
| YPR119W | YAL019W | Biochemical Activity | Phosphorylation | 14574415 |

| | | | | |
|---|---|---|---|---|
| YPR119W | YHL035C | Biochemical Activity | Phosphorylation | 14574415 |
| YPR119W | YJR054W | Biochemical Activity | Phosphorylation | 14574415 |
| YPR119W | YNL339C | Biochemical Activity | Phosphorylation | 14574415 |
| YPR119W | YPL250C | Biochemical Activity | Phosphorylation | 14574415 |
| YPR119W | YDR097C | Biochemical Activity | Phosphorylation | 14574415 |
| YPR119W | YDL239C | Biochemical Activity | Phosphorylation | 14574415 |
| YPR119W | YOR066W | Biochemical Activity | Phosphorylation | 14574415 |
| YPR119W | YNL278W | Biochemical Activity | Phosphorylation | 14574415 |
| YPR119W | YOR195W | Biochemical Activity | Phosphorylation | 14574415 |
| YPR119W | YHR164C | Biochemical Activity | Phosphorylation | 14574415 |
| YPR119W | YNL271C | Biochemical Activity | Phosphorylation | 14574415 |
| YPR119W | YOR098C | Biochemical Activity | Phosphorylation | 14574415 |
| YPR119W | YGR238C | Biochemical Activity | Phosphorylation | 14574415 |
| YPR119W | YLR183C | Biochemical Activity | Phosphorylation | 14574415 |
| YPR119W | YGL124C | Biochemical Activity | Phosphorylation | 14574415 |
| YPR119W | YPL194W | Biochemical Activity | Phosphorylation | 14574415 |
| YPR119W | YHR098C | Biochemical Activity | Phosphorylation | 14574415 |
| YPR119W | YDR227W | Biochemical Activity | Phosphorylation | 14574415 |
| YPR119W | YBL105C | Biochemical Activity | Phosphorylation | 14574415 |
| YPR119W | YNL257C | Biochemical Activity | Phosphorylation | 14574415 |
| YPR119W | YBR038W | Biochemical Activity | Phosphorylation | 14574415 |
| YPR119W | YDR052C | Biochemical Activity | Phosphorylation | 14574415 |
| YPR119W | YDR146C | Biochemical Activity | Phosphorylation | 14574415 |
| YPR119W | YPR030W | Biochemical Activity | Phosphorylation | 14574415 |
| YPR119W | YJL076W | Biochemical Activity | Phosphorylation | 14574415 |
| YPR119W | YJL187C | Biochemical Activity | Phosphorylation | 14574415 |
| YPR119W | YGR296W | Biochemical Activity | Phosphorylation | 14574415 |
| YPR119W | YAL024C | Biochemical Activity | Phosphorylation | 14574415 |
| YPR119W | YHR149C | Biochemical Activity | Phosphorylation | 14574415 |
| YPR119W | YLR187W | Biochemical Activity | Phosphorylation | 14574415 |
| YPR119W | YER008C | Biochemical Activity | Phosphorylation | 14574415 |
| YPR119W | YOR188W | Biochemical Activity | Phosphorylation | 14574415 |
| YPR119W | YNL068C | Biochemical Activity | Phosphorylation | 14574415 |
| YPR119W | YOL058W | Biochemical Activity | Phosphorylation | 14574415 |
| YPR119W | YOR014W | Biochemical Activity | Phosphorylation | 14574415 |
| YPR119W | YAL040C | Biochemical Activity | Phosphorylation | 14574415 |
| YPR119W | YKL043W | Biochemical Activity | Phosphorylation | 14574415 |
| YPR119W | YLR035C | Biochemical Activity | Phosphorylation | 14574415 |
| YPR119W | YGR221C | Biochemical Activity | Phosphorylation | 14574415 |
| YPR119W | YCL051W | Biochemical Activity | Phosphorylation | 14574415 |
| YPR119W | YLR278C | Biochemical Activity | Phosphorylation | 14574415 |
| YPR119W | YCL027W | Biochemical Activity | Phosphorylation | 14574415 |
| YPR119W | YGR270W | Biochemical Activity | Phosphorylation | 14574415 |
| YPR119W | YDL189W | Biochemical Activity | Phosphorylation | 14574415 |
| YPR119W | YLR086W | Biochemical Activity | Phosphorylation | 14574415 |
| YPR119W | YHR118C | Biochemical Activity | Phosphorylation | 14574415 |
| YPR119W | YML119W | Biochemical Activity | Phosphorylation | 14574415 |
| YPR119W | YJR083C | Biochemical Activity | Phosphorylation | 14574415 |
| YPR119W | YPL267W | Biochemical Activity | Phosphorylation | 14574415 |
| YPR119W | YDR501W | Biochemical Activity | Phosphorylation | 14574415 |
| YPR119W | YLR425W | Biochemical Activity | Phosphorylation | 14574415 |
| YPR119W | YDR507C | Biochemical Activity | Phosphorylation | 14574415 |
| YPR119W | YOR075W | Biochemical Activity | Phosphorylation | 14574415 |
| YPR119W | YER158C | Biochemical Activity | Phosphorylation | 14574415 |
| YPR119W | YMR036C | Biochemical Activity | Phosphorylation | 14574415 |

| | | | | |
|---|---|---|---|---|
| YPR119W | YER032W | Biochemical Activity | Phosphorylation | 14574415 |
| YPR119W | YMR129W | Biochemical Activity | Phosphorylation | 14574415 |
| YPR119W | YLL003W | Biochemical Activity | Phosphorylation | 14574415 |
| YPR119W | YLR190W | Biochemical Activity | Phosphorylation | 14574415 |
| YPR119W | YDR027C | Biochemical Activity | Phosphorylation | 14574415 |
| YPR119W | YIL106W | Biochemical Activity | Phosphorylation | 14574415 |
| YPR119W | YDR217C | Biochemical Activity | Phosphorylation | 14574415 |
| YPR119W | YPL115C | Biochemical Activity | Phosphorylation | 14574415 |
| YPR119W | YIR023W | Biochemical Activity | Phosphorylation | 14574415 |
| YPR119W | YDR123C | Biochemical Activity | Phosphorylation | 14574415 |
| YPR119W | YOR058C | Biochemical Activity | Phosphorylation | 14574415 |
| YPR119W | YKL129C | Biochemical Activity | Phosphorylation | 14574415 |
| YPR119W | YNL321W | Biochemical Activity | Phosphorylation | 14574415 |
| YPR119W | YOR127W | Biochemical Activity | Phosphorylation | 14574415 |
| YPR119W | YLR079W | Biochemical Activity | Phosphorylation | 14574415 |
| YPR119W | YAL031C | Biochemical Activity | Phosphorylation | 14574415 |
| YPR119W | YLR430W | Biochemical Activity | Phosphorylation | 14574415 |
| YPR119W | YLR457C | Biochemical Activity | Phosphorylation | 14574415 |
| YPR119W | YDR130C | Biochemical Activity | Phosphorylation | 14574415 |
| YPR119W | YFR027W | Biochemical Activity | Phosphorylation | 14574415 |
| YPR119W | YLR182W | Biochemical Activity | Phosphorylation | 14574415 |
| YPR119W | YFR046C | Biochemical Activity | Phosphorylation | 14574415 |
| YPR119W | YLR096W | Biochemical Activity | Phosphorylation | 14574415 |
| YPR119W | YER167W | Biochemical Activity | Phosphorylation | 14574415 |
| YPR119W | YNR047W | Biochemical Activity | Phosphorylation | 14574415 |
| YPR119W | YBR060C | Biochemical Activity | Phosphorylation | 14574415 |
| YPR119W | YDR348C | Biochemical Activity | Phosphorylation | 14574415 |
| YPR119W | YDL025C | Biochemical Activity | Phosphorylation | 14574415 |
| YPR119W | YIL112W | Biochemical Activity | Phosphorylation | 14574415 |
| YPR119W | YOR001W | Biochemical Activity | Phosphorylation | 14574415 |
| YPR119W | YKR095W | Biochemical Activity | Phosphorylation | 14574415 |
| YPR119W | YGL003C | Biochemical Activity | Phosphorylation | 14574415 |
| YPR119W | YER129W | Biochemical Activity | Phosphorylation | 14574415 |
| YPR119W | YCR065W | Biochemical Activity | Phosphorylation | 14574415 |
| YPR119W | YHR159W | Biochemical Activity | Phosphorylation | 14574415 |
| YPR119W | YLL021W | Biochemical Activity | Phosphorylation | 14574415 |
| YPR119W | YJL084C | Biochemical Activity | Phosphorylation | 14574415 |
| YPR119W | YGL116W | Biochemical Activity | Phosphorylation | 14574415 |
| YPR119W | YPR141C | Biochemical Activity | Phosphorylation | 14574415 |
| YPR119W | YOR372C | Biochemical Activity | Phosphorylation | 14574415 |
| YPR119W | YDL113C | Biochemical Activity | Phosphorylation | 14574415 |
| YPR119W | YIL122W | Biochemical Activity | Phosphorylation | 14574415 |
| YPR119W | YML065W | Biochemical Activity | Phosphorylation | 14574415 |
| YPR119W | YBR138C | Biochemical Activity | Phosphorylation | 14574415 |
| YPR119W | YGR092W | Biochemical Activity | Phosphorylation | 14574415 |
| YPR119W | YJL060W | Biochemical Activity | Phosphorylation | 14574415 |
| YPR119W | YPR111W | Biochemical Activity | Phosphorylation | 14574415 |
| YPR119W | YLR223C | Biochemical Activity | Phosphorylation | 14574415 |
| YPR119W | YMR001C | Biochemical Activity | Phosphorylation | 14574415 |
| YPR119W | YDR285W | Biochemical Activity | Phosphorylation | 14574415 |
| YPR119W | YOL070C | Biochemical Activity | Phosphorylation | 14574415 |
| YPR119W | YPL255W | Biochemical Activity | Phosphorylation | 14574415 |
| YPR119W | YDR113C | Biochemical Activity | Phosphorylation | 14574415 |
| YPR119W | YEL032W | Biochemical Activity | Phosphorylation | 14574415 |
| YPR119W | YLR238W | Biochemical Activity | Phosphorylation | 14574415 |

| | | | | |
|---|---|---|---|---|
| YPR119W | YJL092W | Biochemical Activity | Phosphorylation | 14574415 |
| YPR119W | YKR089C | Biochemical Activity | Phosphorylation | 14574415 |
| YPR119W | YOR177C | Biochemical Activity | Phosphorylation | 14574415 |
| YPR119W | YOR037W | Biochemical Activity | Phosphorylation | 14574415 |
| YPR119W | YDR223W | Biochemical Activity | Phosphorylation | 14574415 |
| YPR119W | YKL108W | Biochemical Activity | Phosphorylation | 14574415 |
| YPR119W | YGL075C | Biochemical Activity | Phosphorylation | 14574415 |
| YPR119W | YIL050W | Biochemical Activity | Phosphorylation | 14574415 |
| YPR119W | YNL309W | Biochemical Activity | Phosphorylation | 14574415 |
| YPR119W | YNL042W | Biochemical Activity | Phosphorylation | 14574415 |
| YPR119W | YML034W | Biochemical Activity | Phosphorylation | 14574415 |
| YPR119W | YOR315W | Biochemical Activity | Phosphorylation | 14574415 |
| YPR119W | YOR081C | Biochemical Activity | Phosphorylation | 14574415 |
| YPR119W | YKR090W | Biochemical Activity | Phosphorylation | 14574415 |
| YPR119W | YLR394W | Biochemical Activity | Phosphorylation | 14574415 |
| YPR119W | YJL157C | Biochemical Activity | Phosphorylation | 14574415 |
| YPR119W | YPL155C | Biochemical Activity | Phosphorylation | 14574415 |
| YPR119W | YLR006C | Biochemical Activity | Phosphorylation | 14574415 |
| YPR119W | YPR171W | Biochemical Activity | Phosphorylation | 14574415 |
| YPR119W | YML027W | Biochemical Activity | Phosphorylation | 14574415 |
| YPR119W | YGR186W | Biochemical Activity | Phosphorylation | 14574415 |
| YPR119W | YOL100W | Biochemical Activity | Phosphorylation | 14574415 |
| YPR119W | YDL089W | Biochemical Activity | Phosphorylation | 14574415 |
| YPR119W | YBR200W | Biochemical Activity | Phosphorylation | 14574415 |
| YPR119W | YER041W | Biochemical Activity | Phosphorylation | 14574415 |
| YPR119W | YPL209C | Biochemical Activity | Phosphorylation | 14574415 |
| YPR119W | YKL185W | Biochemical Activity | Phosphorylation | 14574415 |
| YPR119W | YBL013W | Biochemical Activity | Phosphorylation | 14574415 |
| YPR119W | YKR078W | Biochemical Activity | Phosphorylation | 14574415 |
| YPR119W | YOR178C | Biochemical Activity | Phosphorylation | 14574415 |
| YPR119W | YLR131C | Biochemical Activity | Phosphorylation | 14574415 |
| YPR119W | YOR104W | Biochemical Activity | Phosphorylation | 14574415 |
| YPR119W | YPR174C | Biochemical Activity | Phosphorylation | 14574415 |
| YPR119W | YDR093W | Biochemical Activity | Phosphorylation | 14574415 |
| YPR119W | YJR033C | Biochemical Activity | Phosphorylation | 14574415 |
| YPR119W | YMR005W | Biochemical Activity | Phosphorylation | 14574415 |
| YPR119W | YEL065W | Biochemical Activity | Phosphorylation | 14574415 |
| YPR119W | YDR001C | Biochemical Activity | Phosphorylation | 14574415 |
| YPR119W | YAL028W | Biochemical Activity | Phosphorylation | 14574415 |
| YPR119W | YGR014W | Biochemical Activity | Phosphorylation | 14574415 |
| YPR119W | YNL102W | Biochemical Activity | Phosphorylation | 14574415 |
| YPR119W | YBR098W | Biochemical Activity | Phosphorylation | 14574415 |
| YPR119W | YHL022C | Biochemical Activity | Phosphorylation | 14574415 |
| YPR119W | YIL140W | Biochemical Activity | Phosphorylation | 14574415 |
| YPR119W | YBR102C | Biochemical Activity | Phosphorylation | 14574415 |
| YPR119W | YDR439W | Biochemical Activity | Phosphorylation | 14574415 |
| YPR119W | YPR175W | Biochemical Activity | Phosphorylation | 14574415 |
| YPR119W | YDR379W | Biochemical Activity | Phosphorylation | 14574415 |
| YPR119W | YKR091W | Biochemical Activity | Phosphorylation | 14574415 |
| YPR119W | YJR059W | Biochemical Activity | Phosphorylation | 14574415 |
| YPR119W | YKL116C | Biochemical Activity | Phosphorylation | 14574415 |
| YPR119W | YIL101C | Biochemical Activity | Phosphorylation | 14574415 |
| YPR119W | YNL058C | Biochemical Activity | Phosphorylation | 14574415 |
| YPR119W | YLR401C | Biochemical Activity | Phosphorylation | 14574415 |
| YPR119W | YPR018W | Biochemical Activity | Phosphorylation | 14574415 |

| | | | | |
|---|---|---|---|---|
| YPR119W | YGL216W | Biochemical Activity | Phosphorylation | 14574415 |
| YPR119W | YIL031W | Biochemical Activity | Phosphorylation | 14574415 |
| YPR119W | YKL048C | Biochemical Activity | Phosphorylation | 14574415 |
| YPR119W | YDR389W | Biochemical Activity | Phosphorylation | 14574415 |
| YPR119W | YGL235W | Biochemical Activity | Phosphorylation | 14574415 |
| YPR119W | YGR035C | Biochemical Activity | Phosphorylation | 14574415 |
| YPR119W | YHR158C | Biochemical Activity | Phosphorylation | 14574415 |
| YPR119W | YLR319C | Biochemical Activity | Phosphorylation | 14574415 |
| YPR119W | YLR219W | Biochemical Activity | Phosphorylation | 14574415 |
| YPR119W | YOL036W | Biochemical Activity | Phosphorylation | 14574415 |
| YPR119W | YAR002W | Biochemical Activity | Phosphorylation | 14574415 |
| YPR119W | YGL097W | Biochemical Activity | Phosphorylation | 14574415 |
| YPR119W | YLR045C | Biochemical Activity | Phosphorylation | 14574415 |
| YPR119W | YHL050C | Biochemical Activity | Phosphorylation | 14574415 |
| YPR119W | YJR091C | Biochemical Activity | Phosphorylation | 14574415 |
| YPR119W | YDR356W | Biochemical Activity | Phosphorylation | 14574415 |
| YPR119W | YCL014W | Biochemical Activity | Phosphorylation | 14574415 |
| YPR119W | YOR083W | Biochemical Activity | Phosphorylation | 14574415 |
| YPR120C | YGL003C | Biochemical Activity | Phosphorylation | 9831566 |
| YDL155W | YGL003C | Biochemical Activity | Phosphorylation | 9831566 |
| YPR119W | YGL003C | Biochemical Activity | Phosphorylation | 9831566 |
| YNL053W | YHR030C | Biochemical Activity | Dephosphorylation | 14703512 |
| YHR030C | YNL053W | Biochemical Activity | Phosphorylation | 14703512 |
| YHR030C | YLR442C | Biochemical Activity | Phosphorylation | 12504006 |
| YBR160W | YDL017W | Biochemical Activity | Phosphorylation | 8382976 |
| YMR001C | YJL187C | Biochemical Activity | Phosphorylation | 15037762 |
| YNL298W | YJL187C | Biochemical Activity | Phosphorylation | 15037762 |
| YMR199W | YLR079W | Biochemical Activity | Phosphorylation | 12820958 |
| YPL256C | YLR079W | Biochemical Activity | Phosphorylation | 12820958 |
| YAL040C | YLR079W | Biochemical Activity | Phosphorylation | 12820958 |
| YBR160W | YLR079W | Biochemical Activity | Phosphorylation | 12820958 |
| YDR054C | YLR079W | Biochemical Activity | Ubiquitination | 9285816 |
| YFL009W | YLR079W | Biochemical Activity | Ubiquitination | 9285816 |
| YPL256C | YLR079W | Biochemical Activity | Ubiquitination | 9285816 |
| YDL017W | YBL023C | Biochemical Activity | Phosphorylation | 10373538 |
| YPL031C | YKL185W | Biochemical Activity | Phosphorylation | 11703659 |
| YDL008W | YBR082C | Biochemical Activity | Ubiquitination | 10888670 |
| YBR160W | YPR175W | Biochemical Activity | Phosphorylation | 14747467 |
| YDR440W | YBR010W | Biochemical Activity | Methylation | 12080090 |
| YDR440W | YNL031C | Biochemical Activity | Methylation | 12080090 |
| YBR136W | YDR217C | Biochemical Activity | Phosphorylation | 15060150 |
| YDL017W | YBL023C | Biochemical Activity | Phosphorylation | 10508166 |
| YDL017W | YEL032W | Biochemical Activity | Phosphorylation | 10508166 |
| YDL017W | YPR019W | Biochemical Activity | Phosphorylation | 10508166 |
| YDL017W | YLR274W | Biochemical Activity | Phosphorylation | 10508166 |
| YDL017W | YGL201C | Biochemical Activity | Phosphorylation | 10508166 |
| YDL017W | YBR202W | Biochemical Activity | Phosphorylation | 10508166 |
| YGR252W | YBR010W | Biochemical Activity | Acetylation | 9733731 |
| YGR252W | YNL031C | Biochemical Activity | Acetylation | 9733731 |
| YGR252W | YDR225W | Biochemical Activity | Acetylation | 9733731 |
| YGR252W | YBL003C | Biochemical Activity | Acetylation | 9733731 |
| YGR252W | YDR224C | Biochemical Activity | Acetylation | 9733731 |
| YGR252W | YBL002W | Biochemical Activity | Acetylation | 9733731 |
| YGR252W | YBR009C | Biochemical Activity | Acetylation | 9733731 |
| YGR252W | YNL030W | Biochemical Activity | Acetylation | 9733731 |

| | | | | |
|---|---|---|---|---|
| YPL031C | YOL001W | Biochemical Activity | Phosphorylation | 15057567 |
| YOL001W | YFR034C | Biochemical Activity | Phosphorylation | 14659740 |
| YPL031C | YFR034C | Biochemical Activity | Phosphorylation | 14659740 |
| YDL028C | YGL086W | Biochemical Activity | Phosphorylation | 8688079 |
| YIL035C | YKL112W | Biochemical Activity | Phosphorylation | 7608180 |
| YOL001W | YFR034C | Biochemical Activity | Phosphorylation | 11237614 |
| YPL031C | YFR034C | Biochemical Activity | Phosphorylation | 11237614 |
| YDL127W | YFR034C | Biochemical Activity | Phosphorylation | 10588725 |
| YPL031C | YFR034C | Biochemical Activity | Phosphorylation | 10588725 |
| YGL134W | YLR258W | Biochemical Activity | Phosphorylation | 10490639 |
| YPL031C | YLR258W | Biochemical Activity | Phosphorylation | 10490639 |
| YBR034C | YDR432W | Biochemical Activity | Methylation | 9499403 |
| YBR034C | YOL123W | Biochemical Activity | Methylation | 9499403 |
| YBR034C | YNL004W | Biochemical Activity | Methylation | 9499403 |
| YPL042C | YDL005C | Biochemical Activity | Phosphorylation | 14988503 |
| YIL147C | YDL235C | Biochemical Activity | Phosphorylation | 12455952 |
| YIL147C | YHR206W | Biochemical Activity | Phosphorylation | 12455952 |
| YIL147C | YHR206W | Biochemical Activity | Phosphorylation | 11073911 |
| YIL147C | YLR006C | Biochemical Activity | Phosphorylation | 11073911 |
| YKL210W | YBR082C | Biochemical Activity | Ubiquitination | 10089879 |
| YKL010C | YBR082C | Biochemical Activity | Ubiquitination | 10089879 |
| YDL122W | YIL148W | Biochemical Activity | Deubiquitination | 2050695 |
| YDL122W | YKR094C | Biochemical Activity | Deubiquitination | 2050695 |
| YDL122W | YLR167W | Biochemical Activity | Deubiquitination | 2050695 |
| YOR124C | YKR094C | Biochemical Activity | Deubiquitination | 1429680 |
| YOR124C | YLR167W | Biochemical Activity | Deubiquitination | 1429680 |
| YER151C | YKR094C | Biochemical Activity | Deubiquitination | 1429680 |
| YER151C | YLR167W | Biochemical Activity | Deubiquitination | 1429680 |
| YDL122W | YKR094C | Biochemical Activity | Deubiquitination | 1429680 |
| YDL122W | YLR167W | Biochemical Activity | Deubiquitination | 1429680 |
| YJR099W | YKR094C | Biochemical Activity | Deubiquitination | 1429680 |
| YJR099W | YLR167W | Biochemical Activity | Deubiquitination | 1429680 |
| YDR440W | YBR010W | Biochemical Activity | Methylation | 12097318 |
| YBR034C | YBR009C | Biochemical Activity | Methylation | 12097318 |
| YDR440W | YNL031C | Biochemical Activity | Methylation | 12097318 |
| YBR034C | YNL030W | Biochemical Activity | Methylation | 12097318 |
| YDR440W | YBR010W | Biochemical Activity | Methylation | 12086673 |
| YDR440W | YNL031C | Biochemical Activity | Methylation | 12086673 |
| YOR244W | YDR225W | Biochemical Activity | Acetylation | 10082517 |
| YOR244W | YBL003C | Biochemical Activity | Acetylation | 10082517 |
| YOR244W | YBR010W | Biochemical Activity | Acetylation | 10082517 |
| YOR244W | YNL031C | Biochemical Activity | Acetylation | 10082517 |
| YOR244W | YBR009C | Biochemical Activity | Acetylation | 10082517 |
| YOR244W | YNL030W | Biochemical Activity | Acetylation | 10082517 |
| YGR252W | YBR010W | Biochemical Activity | Acetylation | 10082517 |
| YGR252W | YNL031C | Biochemical Activity | Acetylation | 10082517 |
| YBR034C | YGL122C | Biochemical Activity | Methylation | 11779864 |
| YBR034C | YDR432W | Biochemical Activity | Methylation | 10652296 |
| YBR034C | YOL123W | Biochemical Activity | Methylation | 10652296 |
| YIL035C | YML074C | Biochemical Activity | Phosphorylation | 9148902 |
| YOR061W | YML074C | Biochemical Activity | Phosphorylation | 9148902 |
| YGL019W | YML074C | Biochemical Activity | Phosphorylation | 9148902 |
| YOR039W | YML074C | Biochemical Activity | Phosphorylation | 9148902 |
| YJL164C | YPL232W | Biochemical Activity | Phosphorylation | 12925750 |
| YPR176C | YOR370C | Biochemical Activity | Prenalyation | 8183917 |

| | | | | |
|---|---|---|---|---|
| YPR176C | YFL038C | Biochemical Activity | Prenalyation | 8183917 |
| YPR176C | YJL030W | Biochemical Activity | Prenalyation | 8183917 |
| YOR370C | YPR176C | Biochemical Activity | Prenalyation | 8183917 |
| YOR370C | YJL030W | Biochemical Activity | Prenalyation | 8183917 |
| YOR370C | YFL038C | Biochemical Activity | Prenalyation | 8183917 |
| YJL030W | YPR176C | Biochemical Activity | Prenalyation | 8183917 |
| YJL030W | YOR370C | Biochemical Activity | Prenalyation | 8183917 |
| YJL030W | YFL038C | Biochemical Activity | Prenalyation | 8183917 |
| YOL045W | YKL035W | Biochemical Activity | Phosphorylation | 12372297 |
| YOL045W | YOR276W | Biochemical Activity | Phosphorylation | 12372297 |
| YOL045W | YKR059W | Biochemical Activity | Phosphorylation | 12372297 |
| YOL045W | YCL037C | Biochemical Activity | Phosphorylation | 12372297 |
| YLR113W | YLR138W | Biochemical Activity | Phosphorylation | 15294160 |
| YLR113W | YJL093C | Biochemical Activity | Phosphorylation | 15294160 |
| YMR216C | YDR432W | Biochemical Activity | Phosphorylation | 15145958 |
| YBR160W | YOR083W | Biochemical Activity | Phosphorylation | 15210111 |
| YBL016W | YDR480W | Biochemical Activity | Phosphorylation | 9094309 |
| YBL016W | YPL049C | Biochemical Activity | Phosphorylation | 9094309 |
| YDL042C | YBR010W | Biochemical Activity | Deacetylation | 10693811 |
| YDL042C | YNL031C | Biochemical Activity | Deacetylation | 10693811 |
| YDL042C | YBR009C | Biochemical Activity | Deacetylation | 10693811 |
| YDL042C | YNL030W | Biochemical Activity | Deacetylation | 10693811 |
| YDR140W | YBR143C | Biochemical Activity | Methylation | 15509572 |
| YDL159W | YBL016W | Biochemical Activity | Phosphorylation | 15456892 |
| YBR160W | YNL068C | Biochemical Activity | Phosphorylation | 15509804 |
| YPR073C | YML074C | Biochemical Activity | Dephosphorylation | 15358193 |
| YPR119W | YJL076W | Biochemical Activity | Phosphorylation | 15273393 |
| YBL016W | YBR083W | Biochemical Activity | Phosphorylation | 15620356 |
| YLR096W | YGR009C | Biochemical Activity | Phosphorylation | 15563607 |
| YBL016W | YDR103W | Biochemical Activity | Phosphorylation | 15322134 |
| YBL016W | YBR083W | Biochemical Activity | Phosphorylation | 15558284 |
| YJL141C | YDR223W | Biochemical Activity | Phosphorylation | 15620355 |
| YHL007C | YDR224C | Biochemical Activity | Phosphorylation | 15652479 |
| YHL007C | YBL002W | Biochemical Activity | Phosphorylation | 15652479 |
| YIL095W | YIR006C | Biochemical Activity | Phosphorylation | 15711538 |
| YPL042C | YPL248C | Biochemical Activity | Phosphorylation | 15687503 |
| YNL025C | YPL248C | Biochemical Activity | Phosphorylation | 15687503 |
| YIL035C | YFR034C | Biochemical Activity | Phosphorylation | 12606059 |
| YOR061W | YFR034C | Biochemical Activity | Phosphorylation | 12606059 |
| YEL019C | YOL034W | Biochemical Activity | Sumoylation | 15738391 |
| YEL019C | YMR284W | Biochemical Activity | Sumoylation | 15738391 |
| YBR160W | YER041W | Biochemical Activity | Phosphorylation | 15744308 |
| YBR160W | YEL032W | Biochemical Activity | Phosphorylation | 15744308 |
| YBR160W | YDR356W | Biochemical Activity | Phosphorylation | 15744308 |
| YBR160W | YGL075C | Biochemical Activity | Phosphorylation | 15744308 |
| YBR160W | YJL194W | Biochemical Activity | Phosphorylation | 15744308 |
| YBR160W | YDR130C | Biochemical Activity | Phosphorylation | 15744308 |
| YBR160W | YBR060C | Biochemical Activity | Phosphorylation | 15744308 |
| YBR160W | YFR046C | Biochemical Activity | Phosphorylation | 15744308 |
| YBR160W | YHR118C | Biochemical Activity | Phosphorylation | 15744308 |
| YBR160W | YGL003C | Biochemical Activity | Phosphorylation | 15744308 |
| YBR160W | YPL194W | Biochemical Activity | Phosphorylation | 15744308 |
| YDR138W | YER125W | Biochemical Activity | Ubiquitination | 15713680 |
| YDR113C | YGR225W | Biochemical Activity | Ubiquitination | 15797379 |
| YJL164C | YNL027W | Biochemical Activity | Phosphorylation | 15470242 |

| | | | | |
|---|---|---|---|---|
| YHR004C | YMR165C | Biochemical Activity | Dephosphorylation | 15889145 |
| YAL009W | YMR165C | Biochemical Activity | Dephosphorylation | 15889145 |
| Q0160 | YBL075C | Biochemical Activity | Phosphorylation | 15618627 |
| YDR490C | YMR104C | Biochemical Activity | Phosphorylation | 15840588 |
| YDR490C | YKL126W | Biochemical Activity | Phosphorylation | 15840588 |
| YDR490C | YHR205W | Biochemical Activity | Phosphorylation | 15840588 |
| YLR452C | YBL016W | Biochemical Activity | Phosphorylation | 15924435 |
| YKL203C | YMR104C | Biochemical Activity | Phosphorylation | 16055732 |
| YLR128W | YDL132W | Biochemical Activity | Nedd8(Rub1)ylation | 15988528 |
| YER125W | YKL020C | Biochemical Activity | Ubiquitination | 15933713 |
| YOR124C | YKL020C | Biochemical Activity | Deubiquitination | 15933713 |
| YBR136W | YPL153C | Biochemical Activity | Phosphorylation | 16085488 |
| YFL029C | YKL139W | Biochemical Activity | Phosphorylation | 15870265 |
| YKL210W | YDL140C | Biochemical Activity | Ubiquitination | 15960978 |
| YDR059C | YDL140C | Biochemical Activity | Ubiquitination | 15960978 |
| YER125W | YDL140C | Biochemical Activity | Ubiquitination | 15960978 |
| YKL054C | YDL140C | Biochemical Activity | Ubiquitination | 15960978 |
| YOR061W | YOR254C | Biochemical Activity | Phosphorylation | 15671059 |
| YER129W | YGL035C | Biochemical Activity | Phosphorylation | 16201971 |
| YER129W | YDR477W | Biochemical Activity | Phosphorylation | 16201971 |
| YPR119W | YEL032W | Biochemical Activity | Phosphorylation | 16093348 |
| YBR136W | YNL312W | Biochemical Activity | Phosphorylation | 16118184 |
| YIL035C | YLR079W | Biochemical Activity | Phosphorylation | 16168390 |
| YOR061W | YLR079W | Biochemical Activity | Phosphorylation | 16168390 |
| YGL019W | YLR079W | Biochemical Activity | Phosphorylation | 16168390 |
| YOR039W | YLR079W | Biochemical Activity | Phosphorylation | 16168390 |
| YIL035C | YLR079W | Biochemical Activity | Phosphorylation | 16168390 |
| YOR061W | YLR079W | Biochemical Activity | Phosphorylation | 16168390 |
| YBR160W | YJL187C | Biochemical Activity | Phosphorylation | 16096060 |
| YHR135C | YDR301W | Biochemical Activity | Phosphorylation | 16137619 |
| YHR135C | YLR277C | Biochemical Activity | Phosphorylation | 16137619 |
| YHR135C | YJR093C | Biochemical Activity | Phosphorylation | 16137619 |
| YHR135C | YAL043C | Biochemical Activity | Phosphorylation | 16137619 |
| YER133W | YDR301W | Biochemical Activity | Dephosphorylation | 16137619 |
| YER133W | YLR277C | Biochemical Activity | Dephosphorylation | 16137619 |
| YER133W | YJR093C | Biochemical Activity | Dephosphorylation | 16137619 |
| YER133W | YAL043C | Biochemical Activity | Dephosphorylation | 16137619 |
| YMR001C | YPR007C | Biochemical Activity | Phosphorylation | 16269332 |
| YDR510W | YNL088W | Biochemical Activity | Sumoylation | 16204216 |
| YOR244W | YBR009C | Biochemical Activity | Acetylation | 16135807 |
| YFL024C | YBR009C | Biochemical Activity | Acetylation | 16135807 |
| YOR244W | YNL030W | Biochemical Activity | Acetylation | 16135807 |
| YFL024C | YNL030W | Biochemical Activity | Acetylation | 16135807 |
| YOR039W | YBR009C | Biochemical Activity | Phosphorylation | 16135807 |
| YOR039W | YNL030W | Biochemical Activity | Phosphorylation | 16135807 |
| YBR136W | YPR010C | Biochemical Activity | Phosphorylation | 14642562 |
| YBR160W | YJL157C | Biochemical Activity | Phosphorylation | 14574415 |
| YBR160W | YAL040C | Biochemical Activity | Phosphorylation | 14574415 |
| YBR160W | YIL050W | Biochemical Activity | Phosphorylation | 14574415 |
| YBR160W | YNL309W | Biochemical Activity | Phosphorylation | 14574415 |
| YBR160W | YGL003C | Biochemical Activity | Phosphorylation | 14574415 |
| YBR160W | YKL108W | Biochemical Activity | Phosphorylation | 14574415 |
| YBR160W | YDR113C | Biochemical Activity | Phosphorylation | 14574415 |
| YBR160W | YJL187C | Biochemical Activity | Phosphorylation | 14574415 |
| YBR160W | YLR079W | Biochemical Activity | Phosphorylation | 14574415 |

| | | | | |
|---|---|---|---|---|
| YBR160W | YER129W | Biochemical Activity | Phosphorylation | 14574415 |
| YBR160W | YJR092W | Biochemical Activity | Phosphorylation | 14574415 |
| YBR160W | YAL019W | Biochemical Activity | Phosphorylation | 14574415 |
| YBR160W | YHL035C | Biochemical Activity | Phosphorylation | 14574415 |
| YBR160W | YJR054W | Biochemical Activity | Phosphorylation | 14574415 |
| YBR160W | YNL339C | Biochemical Activity | Phosphorylation | 14574415 |
| YBR160W | YPL250C | Biochemical Activity | Phosphorylation | 14574415 |
| YBR160W | YDR097C | Biochemical Activity | Phosphorylation | 14574415 |
| YBR160W | YDL239C | Biochemical Activity | Phosphorylation | 14574415 |
| YBR160W | YOR066W | Biochemical Activity | Phosphorylation | 14574415 |
| YBR160W | YNL278W | Biochemical Activity | Phosphorylation | 14574415 |
| YBR160W | YOR195W | Biochemical Activity | Phosphorylation | 14574415 |
| YBR160W | YHR164C | Biochemical Activity | Phosphorylation | 14574415 |
| YBR160W | YNL271C | Biochemical Activity | Phosphorylation | 14574415 |
| YBR160W | YGR238C | Biochemical Activity | Phosphorylation | 14574415 |
| YBR160W | YLR183C | Biochemical Activity | Phosphorylation | 14574415 |
| YBR160W | YGL124C | Biochemical Activity | Phosphorylation | 14574415 |
| YBR160W | YPL194W | Biochemical Activity | Phosphorylation | 14574415 |
| YBR160W | YHR098C | Biochemical Activity | Phosphorylation | 14574415 |
| YBR160W | YDR227W | Biochemical Activity | Phosphorylation | 14574415 |
| YBR160W | YBL105C | Biochemical Activity | Phosphorylation | 14574415 |
| YBR160W | YNL257C | Biochemical Activity | Phosphorylation | 14574415 |
| YBR160W | YBR038W | Biochemical Activity | Phosphorylation | 14574415 |
| YBR160W | YDR052C | Biochemical Activity | Phosphorylation | 14574415 |
| YBR160W | YDR146C | Biochemical Activity | Phosphorylation | 14574415 |
| YBR160W | YPR030W | Biochemical Activity | Phosphorylation | 14574415 |
| YBR160W | YJL076W | Biochemical Activity | Phosphorylation | 14574415 |
| YBR160W | YGR296W | Biochemical Activity | Phosphorylation | 14574415 |
| YBR160W | YAL024C | Biochemical Activity | Phosphorylation | 14574415 |
| YBR160W | YHR149C | Biochemical Activity | Phosphorylation | 14574415 |
| YBR160W | YLR187W | Biochemical Activity | Phosphorylation | 14574415 |
| YBR160W | YER008C | Biochemical Activity | Phosphorylation | 14574415 |
| YBR160W | YOR188W | Biochemical Activity | Phosphorylation | 14574415 |
| YBR160W | YKR095W | Biochemical Activity | Phosphorylation | 14574415 |
| YBR160W | YNL068C | Biochemical Activity | Phosphorylation | 14574415 |
| YBR160W | YOL058W | Biochemical Activity | Phosphorylation | 14574415 |
| YBR160W | YOR014W | Biochemical Activity | Phosphorylation | 14574415 |
| YBR160W | YKL043W | Biochemical Activity | Phosphorylation | 14574415 |
| YBR160W | YLR035C-A | Biochemical Activity | Phosphorylation | 14574415 |
| YBR160W | YGR221C | Biochemical Activity | Phosphorylation | 14574415 |
| YBR160W | YCL051W | Biochemical Activity | Phosphorylation | 14574415 |
| YBR160W | YLR278C | Biochemical Activity | Phosphorylation | 14574415 |
| YBR160W | YCL027W | Biochemical Activity | Phosphorylation | 14574415 |
| YBR160W | YGR270W | Biochemical Activity | Phosphorylation | 14574415 |
| YBR160W | YDL189W | Biochemical Activity | Phosphorylation | 14574415 |
| YBR160W | YLR086W | Biochemical Activity | Phosphorylation | 14574415 |
| YBR160W | YHR118C | Biochemical Activity | Phosphorylation | 14574415 |
| YBR160W | YHR118C | Biochemical Activity | Phosphorylation | 14574415 |
| YBR160W | YML119W | Biochemical Activity | Phosphorylation | 14574415 |
| YBR160W | YJR083C | Biochemical Activity | Phosphorylation | 14574415 |
| YBR160W | YPL267W | Biochemical Activity | Phosphorylation | 14574415 |
| YBR160W | YDR501W | Biochemical Activity | Phosphorylation | 14574415 |
| YBR160W | YLR425W | Biochemical Activity | Phosphorylation | 14574415 |
| YBR160W | YDR507C | Biochemical Activity | Phosphorylation | 14574415 |
| YBR160W | YOR075W | Biochemical Activity | Phosphorylation | 14574415 |

| | | | | |
|---|---|---|---|---|
| YBR160W | YER158C | Biochemical Activity | Phosphorylation | 14574415 |
| YBR160W | YMR036C | Biochemical Activity | Phosphorylation | 14574415 |
| YBR160W | YER032W | Biochemical Activity | Phosphorylation | 14574415 |
| YBR160W | YMR129W | Biochemical Activity | Phosphorylation | 14574415 |
| YBR160W | YLL003W | Biochemical Activity | Phosphorylation | 14574415 |
| YBR160W | YLR190W | Biochemical Activity | Phosphorylation | 14574415 |
| YBR160W | YDR027C | Biochemical Activity | Phosphorylation | 14574415 |
| YBR160W | YIL106W | Biochemical Activity | Phosphorylation | 14574415 |
| YBR160W | YDR217C | Biochemical Activity | Phosphorylation | 14574415 |
| YBR160W | YPL115C | Biochemical Activity | Phosphorylation | 14574415 |
| YBR160W | YIR023W | Biochemical Activity | Phosphorylation | 14574415 |
| YBR160W | YDR123C | Biochemical Activity | Phosphorylation | 14574415 |
| YBR160W | YOR058C | Biochemical Activity | Phosphorylation | 14574415 |
| YBR160W | YKL129C | Biochemical Activity | Phosphorylation | 14574415 |
| YBR160W | YNL321W | Biochemical Activity | Phosphorylation | 14574415 |
| YBR160W | YOR127W | Biochemical Activity | Phosphorylation | 14574415 |
| YBR160W | YAL031C | Biochemical Activity | Phosphorylation | 14574415 |
| YBR160W | YLR430W | Biochemical Activity | Phosphorylation | 14574415 |
| YBR160W | YLR457C | Biochemical Activity | Phosphorylation | 14574415 |
| YBR160W | YLR457C | Biochemical Activity | Phosphorylation | 14574415 |
| YBR160W | YDR130C | Biochemical Activity | Phosphorylation | 14574415 |
| YBR160W | YDR130C | Biochemical Activity | Phosphorylation | 14574415 |
| YBR160W | YFR027W | Biochemical Activity | Phosphorylation | 14574415 |
| YBR160W | YLR182W | Biochemical Activity | Phosphorylation | 14574415 |
| YBR160W | YFR046C | Biochemical Activity | Phosphorylation | 14574415 |
| YBR160W | YLR096W | Biochemical Activity | Phosphorylation | 14574415 |
| YBR160W | YER167W | Biochemical Activity | Phosphorylation | 14574415 |
| YBR160W | YNR047W | Biochemical Activity | Phosphorylation | 14574415 |
| YBR160W | YBR060C | Biochemical Activity | Phosphorylation | 14574415 |
| YBR160W | YDR348C | Biochemical Activity | Phosphorylation | 14574415 |
| YBR160W | YDL025C | Biochemical Activity | Phosphorylation | 14574415 |
| YBR160W | YIL112W | Biochemical Activity | Phosphorylation | 14574415 |
| YBR160W | YOR001W | Biochemical Activity | Phosphorylation | 14574415 |
| YBR160W | YCR065W | Biochemical Activity | Phosphorylation | 14574415 |
| YBR160W | YHR159W | Biochemical Activity | Phosphorylation | 14574415 |
| YBR160W | YLL021W | Biochemical Activity | Phosphorylation | 14574415 |
| YBR160W | YJL084C | Biochemical Activity | Phosphorylation | 14574415 |
| YBR160W | YGL116W | Biochemical Activity | Phosphorylation | 14574415 |
| YBR160W | YPR141C | Biochemical Activity | Phosphorylation | 14574415 |
| YBR160W | YOR372C | Biochemical Activity | Phosphorylation | 14574415 |
| YBR160W | YDL113C | Biochemical Activity | Phosphorylation | 14574415 |
| YBR160W | YIL122W | Biochemical Activity | Phosphorylation | 14574415 |
| YBR160W | YML065W | Biochemical Activity | Phosphorylation | 14574415 |
| YBR160W | YBR138C | Biochemical Activity | Phosphorylation | 14574415 |
| YBR160W | YGR092W | Biochemical Activity | Phosphorylation | 14574415 |
| YBR160W | YJL060W | Biochemical Activity | Phosphorylation | 14574415 |
| YBR160W | YPR111W | Biochemical Activity | Phosphorylation | 14574415 |
| YBR160W | YLR223C | Biochemical Activity | Phosphorylation | 14574415 |
| YBR160W | YMR001C | Biochemical Activity | Phosphorylation | 14574415 |
| YBR160W | YDR285W | Biochemical Activity | Phosphorylation | 14574415 |
| YBR160W | YOL070C | Biochemical Activity | Phosphorylation | 14574415 |
| YBR160W | YPL255W | Biochemical Activity | Phosphorylation | 14574415 |
| YBR160W | YEL032W | Biochemical Activity | Phosphorylation | 14574415 |
| YBR160W | YLR238W | Biochemical Activity | Phosphorylation | 14574415 |
| YBR160W | YJL092W | Biochemical Activity | Phosphorylation | 14574415 |

| | | | | |
|---|---|---|---|---|
| YBR160W | YKR089C | Biochemical Activity | Phosphorylation | 14574415 |
| YBR160W | YOR177C | Biochemical Activity | Phosphorylation | 14574415 |
| YBR160W | YOR037W | Biochemical Activity | Phosphorylation | 14574415 |
| YBR160W | YDR223W | Biochemical Activity | Phosphorylation | 14574415 |
| YBR160W | YGL075C | Biochemical Activity | Phosphorylation | 14574415 |
| YBR160W | YNL042W | Biochemical Activity | Phosphorylation | 14574415 |
| YBR160W | YML034W | Biochemical Activity | Phosphorylation | 14574415 |
| YBR160W | YOR315W | Biochemical Activity | Phosphorylation | 14574415 |
| YBR160W | YOR081C | Biochemical Activity | Phosphorylation | 14574415 |
| YBR160W | YKR090W | Biochemical Activity | Phosphorylation | 14574415 |
| YBR160W | YLR394W | Biochemical Activity | Phosphorylation | 14574415 |
| YBR160W | YPL155C | Biochemical Activity | Phosphorylation | 14574415 |
| YBR160W | YLR006C | Biochemical Activity | Phosphorylation | 14574415 |
| YBR160W | YPR171W | Biochemical Activity | Phosphorylation | 14574415 |
| YBR160W | YML027W | Biochemical Activity | Phosphorylation | 14574415 |
| YBR160W | YGR186W | Biochemical Activity | Phosphorylation | 14574415 |
| YBR160W | YOL100W | Biochemical Activity | Phosphorylation | 14574415 |
| YBR160W | YDL089W | Biochemical Activity | Phosphorylation | 14574415 |
| YBR160W | YBR200W | Biochemical Activity | Phosphorylation | 14574415 |
| YBR160W | YER041W | Biochemical Activity | Phosphorylation | 14574415 |
| YBR160W | YPL209C | Biochemical Activity | Phosphorylation | 14574415 |
| YBR160W | YKL185W | Biochemical Activity | Phosphorylation | 14574415 |
| YBR160W | YBL013W | Biochemical Activity | Phosphorylation | 14574415 |
| YBR160W | YKR078W | Biochemical Activity | Phosphorylation | 14574415 |
| YBR160W | YOR178C | Biochemical Activity | Phosphorylation | 14574415 |
| YBR160W | YLR131C | Biochemical Activity | Phosphorylation | 14574415 |
| YBR160W | YOR104W | Biochemical Activity | Phosphorylation | 14574415 |
| YBR160W | YPR174C | Biochemical Activity | Phosphorylation | 14574415 |
| YBR160W | YDR093W | Biochemical Activity | Phosphorylation | 14574415 |
| YBR160W | YJR033C | Biochemical Activity | Phosphorylation | 14574415 |
| YBR160W | YMR005W | Biochemical Activity | Phosphorylation | 14574415 |
| YBR160W | YEL065W | Biochemical Activity | Phosphorylation | 14574415 |
| YBR160W | YDR001C | Biochemical Activity | Phosphorylation | 14574415 |
| YBR160W | YAL028W | Biochemical Activity | Phosphorylation | 14574415 |
| YBR160W | YGR014W | Biochemical Activity | Phosphorylation | 14574415 |
| YBR160W | YNL102W | Biochemical Activity | Phosphorylation | 14574415 |
| YBR160W | YBR098W | Biochemical Activity | Phosphorylation | 14574415 |
| YBR160W | YHL022C | Biochemical Activity | Phosphorylation | 14574415 |
| YBR160W | YIL140W | Biochemical Activity | Phosphorylation | 14574415 |
| YBR160W | YBR102C | Biochemical Activity | Phosphorylation | 14574415 |
| YBR160W | YDR439W | Biochemical Activity | Phosphorylation | 14574415 |
| YBR160W | YPR175W | Biochemical Activity | Phosphorylation | 14574415 |
| YBR160W | YDR379W | Biochemical Activity | Phosphorylation | 14574415 |
| YBR160W | YKR091W | Biochemical Activity | Phosphorylation | 14574415 |
| YBR160W | YJR059W | Biochemical Activity | Phosphorylation | 14574415 |
| YBR160W | YKL116C | Biochemical Activity | Phosphorylation | 14574415 |
| YBR160W | YIL101C | Biochemical Activity | Phosphorylation | 14574415 |
| YBR160W | YNL058C | Biochemical Activity | Phosphorylation | 14574415 |
| YBR160W | YLR401C | Biochemical Activity | Phosphorylation | 14574415 |
| YBR160W | YPR018W | Biochemical Activity | Phosphorylation | 14574415 |
| YBR160W | YGL216W | Biochemical Activity | Phosphorylation | 14574415 |
| YBR160W | YIL031W | Biochemical Activity | Phosphorylation | 14574415 |
| YBR160W | YKL048C | Biochemical Activity | Phosphorylation | 14574415 |
| YBR160W | YDR389W | Biochemical Activity | Phosphorylation | 14574415 |
| YBR160W | YGL235W | Biochemical Activity | Phosphorylation | 14574415 |

| | | | | |
|---|---|---|---|---|
| YBR160W | YGR035C | Biochemical Activity | Phosphorylation | 14574415 |
| YBR160W | YHR158C | Biochemical Activity | Phosphorylation | 14574415 |
| YBR160W | YLR319C | Biochemical Activity | Phosphorylation | 14574415 |
| YBR160W | YLR219W | Biochemical Activity | Phosphorylation | 14574415 |
| YBR160W | YOL036W | Biochemical Activity | Phosphorylation | 14574415 |
| YBR160W | YAR002W | Biochemical Activity | Phosphorylation | 14574415 |
| YBR160W | YGL097W | Biochemical Activity | Phosphorylation | 14574415 |
| YBR160W | YLR045C | Biochemical Activity | Phosphorylation | 14574415 |
| YBR160W | YHL050C | Biochemical Activity | Phosphorylation | 14574415 |
| YBR160W | YJR091C | Biochemical Activity | Phosphorylation | 14574415 |
| YBR160W | YDR356W | Biochemical Activity | Phosphorylation | 14574415 |
| YBR160W | YCL014W | Biochemical Activity | Phosphorylation | 14574415 |
| YBR160W | YOR083W | Biochemical Activity | Phosphorylation | 14574415 |
| YBR136W | YPL153C | Biochemical Activity | Phosphorylation | 12917350 |
| YBR160W | YKL108W | Biochemical Activity | Phosphorylation | 11807498 |
| YPL209C | YBR010W | Biochemical Activity | Phosphorylation | 11498592 |
| YDL017W | YBL023C | Biochemical Activity | Phosphorylation | 10964916 |

## Supplementary Table 5. Overlap of physical and genetic interaction datasets.

| Overlap Statistics | | | | | |
|---|---|---|---|---|---|
| | | | | | |
| **LC-GI** | **LC-PI** | | | | |
| | **# Nodes** | **# Essential Nodes** | **# Edges** | **# e-e Edges** | **# of Edges with at least 1 essential Node** |
| Dosage Rescue | 668 | 326 | 566 | 222 | 423 |
| Synthetic Lethality | 484 | 259 | 477 | 203 | 384 |
| Synthetic Rescue | 354 | 128 | 261 | 55 | 150 |
| Phenotypic Enhancement | 294 | 65 | 216 | 17 | 79 |
| Synthetic Growth Defect | 253 | 97 | 172 | 44 | 102 |
| Phenotypic Suppression | 143 | 48 | 93 | 13 | 44 |
| Dosage Lethality | 132 | 74 | 89 | 36 | 66 |
| Dosage Growth Defect | 10 | 5 | 6 | 1 | 6 |
| **total** | **1163** | **458** | **1409** | **442** | **930** |
| | | | | | |
| **LC-GI** | **HTP-PI** | | | | |
| | **# Nodes** | **# Essential Nodes** | **# Edges** | **# e-e Edges** | **# of Edges with at least 1 essential Node** |
| Dosage Rescue | 272 | 150 | 169 | 73 | 123 |
| Synthetic Lethality | 201 | 114 | 131 | 49 | 100 |
| Phenotypic Enhancement | 109 | 17 | 63 | 5 | 14 |
| Synthetic Rescue | 104 | 38 | 62 | 14 | 33 |
| Synthetic Growth Defect | 77 | 25 | 45 | 9 | 19 |
| Phenotypic Suppression | 46 | 12 | 24 | 4 | 8 |
| Dosage Lethality | 24 | 15 | 14 | 6 | 12 |
| Dosage Growth Defect | 2 | 1 | 1 | 0 | 1 |
| **total** | **512** | **235** | **385** | **130** | **237** |
| | | | | | |
| **HTP-GI** | **LC-PI** | | | | |
| | **# Nodes** | **# Essential Nodes** | **# Edges** | **# e-e Edges** | **# of Edges with at least 1 essential Node** |
| Synthetic Lethality | 90 | 11 | 78 | 0 | 24 |
| Synthetic Rescue | 4 | 1 | 2 | 0 | 1 |
| Phenotypic Suppression | 2 | 1 | 1 | 0 | 1 |
| Phenotypic Enhancement | 2 | 0 | 1 | 0 | 0 |
| Dosage Lethality | 0 | 0 | 0 | 0 | 0 |
| Dosage Growth Defect | 0 | 0 | 0 | 0 | 0 |
| Synthetic Growth Defect | 0 | 0 | 0 | 0 | 0 |
| **total** | **98** | **13** | **82** | **0** | **26** |
| | | | | | |
| **HTP-GI** | **HTP-PI** | | | | |
| | **# Nodes** | **# Essential Nodes** | **# Edges** | **# e-e Edges** | **# of Edges with at least 1 essential Node** |
| Synthetic Lethality | 21 | 4 | 11 | 0 | 4 |
| Synthetic Rescue | 2 | 1 | 1 | 0 | 1 |
| Synthetic Growth Defect | 2 | 0 | 1 | 0 | 0 |
| Dosage Lethality | 0 | 0 | 0 | 0 | 0 |

| | | | | | |
|---|---|---|---|---|---|
| Dosage Growth Defect | 0 | 0 | 0 | 0 | 0 |
| Phenotypic Suppression | 0 | 0 | 0 | 0 | 0 |
| Phenotypic Enhancement | 0 | 0 | 0 | 0 | 0 |
| **total** | **25** | **5** | **13** | **0** | **5** |

| **LC-PI** | **LC-GI** | | | | |
|---|---|---|---|---|---|
| | **# Nodes** | **# Essential Nodes** | **# Edges** | **# e-e Edges** | **# of Edges with at least 1 essential Node** |
| Affinity Capture-Western | 856 | 366 | 889 | 286 | 597 |
| Two-hybrid | 620 | 260 | 540 | 171 | 336 |
| Reconstituted Complex | 462 | 231 | 402 | 161 | 299 |
| Affinity Capture-MS | 339 | 193 | 282 | 128 | 210 |
| Co-purification | 208 | 120 | 149 | 73 | 114 |
| Biochemical Activity | 171 | 59 | 141 | 31 | 76 |
| Co-fractionation | 133 | 73 | 89 | 44 | 62 |
| Co-localization | 65 | 26 | 43 | 9 | 24 |
| Co-crystal Structure | 34 | 22 | 24 | 14 | 20 |
| Far Western | 33 | 22 | 18 | 9 | 16 |
| Protein-peptide | 19 | 7 | 12 | 4 | 7 |
| FRET | 15 | 12 | 10 | 8 | 9 |
| Protein-RNA | 8 | 0 | 4 | 0 | 0 |
| Affinity Capture-RNA | 2 | 0 | 1 | 0 | 0 |
| **total** | **1163** | **458** | **1409** | **442** | **930** |

| **LC-PI** | **HTP-GI** | | | | |
|---|---|---|---|---|---|
| | **# Nodes** | **# Essential Nodes** | **# Edges** | **# e-e Edges** | **# of Edges with at least 1 essential Node** |
| Two-hybrid | 53 | 7 | 38 | 0 | 12 |
| Affinity Capture-Western | 43 | 6 | 28 | 0 | 13 |
| Affinity Capture-MS | 25 | 1 | 21 | 0 | 1 |
| Reconstituted Complex | 19 | 3 | 12 | 0 | 7 |
| Biochemical Activity | 9 | 2 | 5 | 0 | 2 |
| Co-purification | 5 | 0 | 3 | 0 | 0 |
| FRET | 3 | 0 | 2 | 0 | 0 |
| Protein-peptide | 2 | 0 | 1 | 0 | 0 |
| Far Western | 2 | 0 | 1 | 0 | 0 |
| Co-localization | 2 | 1 | 1 | 0 | 1 |
| Co-fractionation | 0 | 0 | 0 | 0 | 0 |
| Affinity Capture-RNA | 0 | 0 | 0 | 0 | 0 |
| Co-crystal Structure | 0 | 0 | 0 | 0 | 0 |
| Protein-RNA | 0 | 0 | 0 | 0 | 0 |
| **total** | **98** | **13** | **82** | **0** | **26** |

| **HTP-PI** | **LC-GI** | | | | |
|---|---|---|---|---|---|
| | **# Nodes** | **# Essential Nodes** | **# Edges** | **# e-e Edges** | **# of Edges with at least 1 essential Node** |
| Affinity Capture-MS | 414 | 186 | 312 | 99 | 188 |
| Two-hybrid | 183 | 98 | 113 | 49 | 74 |
| **total** | **512** | **235** | **385** | **130** | **237** |

| **HTP-PI** | **HTP-GI** |
|---|---|

| | # Nodes | # Essential Nodes | # Edges | # e-e Edges | # of Edges with at least 1 essential Node |
|---|---|---|---|---|---|
| Affinity Capture-MS | 16 | 5 | 8 | 0 | 5 |
| Two-hybrid | 12 | 1 | 6 | 0 | 1 |
| **total** | **25** | **5** | **13** | **0** | **5** |
| | | | | | |
| **HTP-PI** | **LC-PI** | | | | |
| | **# Nodes** | **# Essential Nodes** | **# Edges** | **# e-e Edges** | **# of Edges with at least 1 essential Node** |
| Affinity Capture-MS | 946 | 443 | 1344 | 618 | 1007 |
| Two-hybrid | 520 | 198 | 396 | 119 | 224 |
| **total** | **1201** | **512** | **1624** | **692** | **1161** |
| | | | | | |
| **LC-PI** | **HTP-PI** | | | | |
| | **# Nodes** | **# Essential Nodes** | **# Edges** | **# e-e Edges** | **# of Edges with at least 1 essential Node** |
| Affinity Capture-MS | 566 | 345 | 866 | 486 | 728 |
| Affinity Capture-Western | 740 | 307 | 727 | 259 | 472 |
| Two-hybrid | 602 | 238 | 487 | 145 | 289 |
| Reconstituted Complex | 351 | 168 | 275 | 108 | 187 |
| Co-purification | 262 | 147 | 248 | 120 | 190 |
| Co-fractionation | 111 | 59 | 79 | 37 | 53 |
| Biochemical Activity | 81 | 28 | 52 | 12 | 22 |
| Co-localization | 37 | 13 | 21 | 6 | 8 |
| Co-crystal Structure | 24 | 15 | 16 | 7 | 12 |
| Far Western | 23 | 13 | 13 | 6 | 9 |
| Protein-peptide | 15 | 3 | 11 | 1 | 5 |
| FRET | 8 | 6 | 4 | 2 | 4 |
| Affinity Capture-RNA | 0 | 0 | 0 | 0 | 0 |
| Protein-RNA | 0 | 0 | 0 | 0 | 0 |
| **total** | **1201** | **512** | **1624** | **692** | **1161** |
| | | | | | |
| **HTP-GI** | **LC-GI** | | | | |
| | **# Nodes** | **# Essential Nodes** | **# Edges** | **# e-e Edges** | **# of Edges with at least 1 essential Node** |
| Synthetic Lethality | 206 | 20 | 298 | 1 | 42 |
| Synthetic Growth Defect | 12 | 0 | 9 | 0 | 0 |
| Synthetic Rescue | 7 | 3 | 4 | 0 | 3 |
| Phenotypic Suppression | 4 | 1 | 2 | 0 | 1 |
| Phenotypic Enhancement | 2 | 0 | 1 | 0 | 0 |
| Dosage Lethality | 0 | 0 | 0 | 0 | 0 |
| Dosage Growth Defect | 0 | 0 | 0 | 0 | 0 |
| **total** | **216** | **23** | **305** | **1** | **45** |
| | | | | | |
| **LC-GI** | **HTP-GI** | | | | |
| | **# Nodes** | **# Essential Nodes** | **# Edges** | **# e-e Edges** | **# of Edges with at least 1 essential Node** |
| Synthetic Lethality | 161 | 16 | 201 | 0 | 24 |
| Synthetic Growth Defect | 59 | 2 | 51 | 0 | 2 |
| Dosage Rescue | 63 | 9 | 48 | 0 | 16 |

| | | | | | |
|---|---|---|---|---|---|
| Phenotypic Enhancement | 54 | 4 | 45 | 0 | 4 |
| Synthetic Rescue | 27 | 6 | 15 | 0 | 7 |
| Dosage Lethality | 20 | 6 | 12 | 1 | 6 |
| Phenotypic Suppression | 4 | 0 | 2 | 0 | 0 |
| Dosage Growth Defect | 2 | 0 | 1 | 0 | 0 |
| **total** | **216** | **23** | **305** | **1** | **45** |

**Supplementary Table 6. Predicted yeast complexes from yeast interaction datasets.**

| | |
|---|---|
| complex_0 | YBR081C \| YBR111W-A \| YDR167W \| YDR176W \| YMR223W \| YMR236W \| YBR198C \| YDR145W \| YDR392W \| YGL112C \| YLR055C \| YOL148C \| YPL047W \| YPL254W \| YDR448W |
| complex_1 | YCR052W \| YER148W \| YFR037C \| YGR056W \| YLR033W \| YLR321C \| YLR357W \| YMR033W \| YDR303C \| YGR275W \| YIL126W \| YKR008W \| YML127W \| YMR091C \| YPR034W |
| complex_100 | YCR052W \| YFR037C \| YGR056W \| YLR033W \| YLR357W \| YMR033W \| YDR303C \| YGR275W \| YIL126W \| YKR008W \| YML127W \| YMR091C \| YPR034W \| YHR056C \| YFL067W |
| complex_1001 | YBL041W \| YDL020C \| YOL038W \| YPR103W \| YDL188C \| YER012W \| YGL011C \| YGR135W \| YMR314W \| YOR362C \| YHR200W \| YGL058W \| YLR024C \| YOL080C \| YML092C |
| complex_1002 | YBL050W \| YDR189W \| YGL098W \| YKL196C \| YLR026C \| YLR268W \| YOR075W \| YLR440C \| YDR498C \| YNL258C \| YER157W \| YGR120C \| YGL145W \| YPR105C \| YGL223C |
| complex_1003 | YBR154C \| YJL148W \| YJR063W \| YNL113W \| YPR010C \| YPR110C \| YER004W \| YER009W \| YOR210W \| YKL144C \| YML010W \| YNL248C \| YOR340C \| YOR341W \| YPR187W |
| complex_1004 | YBL050W \| YDR189W \| YGL098W \| YKL196C \| YLR026C \| YLR268W \| YOR075W \| YLR440C \| YDR498C \| YNL258C \| YGL145W \| YHL031C \| YKL006C-A \| YMR020W \| YPL244C |
| complex_1005 | YBL050W \| YDR189W \| YGL098W \| YKL196C \| YLR026C \| YLR268W \| YOR075W \| YLR440C \| YDR498C \| YNL258C \| YGL145W \| YGR263C \| YNL066W \| YHL031C \| YKL006C-A |
| complex_1006 | YAL042W \| YAR002C-A \| YML067C \| YGL054C \| YGL200C \| YGR284C \| YIL004C \| YLR026C \| YML012W \| YDL195W \| YIL109C \| YLR268W \| YPR181C \| YLR078C \| YPL218W |
| complex_1007 | YBL014C \| YKL125W \| YBR154C \| YJL148W \| YJR063W \| YNL113W \| YPR010C \| YPR110C \| YOR210W \| YKL144C \| YML010W \| YNL248C \| YOR340C \| YOR341W \| YPR187W |
| complex_101 | YBR065C \| YDL209C \| YLL036C \| YLR424W \| YMR001C \| YMR213W \| YPR101W \| YBR188C \| YDR416W \| YGR129W \| YJR050W \| YLR117C \| YKL095W \| YKL173W \| YPL151C |
| complex_1013 | YDL145C \| YDL192W \| YFL048C \| YGL137W \| YIL004C \| YNL284C \| YNL287W \| YPL010W \| YDR170C \| YDR238C \| YER122C \| YLR268W \| YFR051C \| YIL076W \| YPR181C |
| complex_1016 | YBR020W \| YML051W \| YCL011C \| YDL084W \| YDR138W \| YDR381W \| YDR432W \| YHR167W \| YKL139W \| YNL112W \| YNL253W \| YCL045C \| YML062C \| YNL004W \| YNL139C |
| complex_1019 | YBL084C \| YDL008W \| YDR118W \| YGL240W \| YIR025W \| YKL022C \| YLR102C \| YLR127C \| YMR001C \| YDL102W \| YJR006W \| YJR043C \| YGR225W \| YNL102W \| YOR249C |
| complex_1020 | YDL065C \| YGL153W \| YDR142C \| YDR244W \| YGR239C \| YHR160C \| YIL160C \| YLR191W \| YNL214W \| YGR077C \| YMR026C \| YDR265W \| YJL210W \| YLR049C \| YAL054C |

| | |
|---|---|
| complex_1021 | YBR152W \| YMR288W \| YBR162W-A \| YLR090W \| YDL030W \| YFL017W-A \| YLL036C \| YML049C \| YMR001C \| YMR213W \| YMR240C \| YOR159C \| YPR182W \| YPR094W \| YPL213W |
| complex_1023 | YDL153C \| YOR252W \| YER002W \| YGR103W \| YLR009W \| YMR049C \| YPL093W \| YER126C \| YKR081C \| YNL110C \| YGR245C \| YNL002C \| YHR066W \| YPR016C \| YPR152C |
| complex_1024 | YDL147W \| YER012W \| YGL011C \| YHR200W \| YJL001W \| YPR103W \| YDL188C \| YER094C \| YFR050C \| YML092C \| YNL085W \| YOL038W \| YOR157C \| YGR067C \| YJL080C |
| complex_1025 | YBR055C \| YER172C \| YGR091W \| YHR165C \| YLL036C \| YLR438C-A \| YNL147W \| YPR178W \| YDR378C \| YDR473C \| YKL173W \| YJR022W \| YLR133W \| YGL021W \| YMR139W |
| complex_1026 | YAR033W \| YKL174C \| YCR035C \| YDR280W \| YGR095C \| YGR195W \| YOL021C \| YOR001W \| YDL111C \| YGR158C \| YHR069C \| YNL232W \| YLR345W \| YLR321C \| YPL036W |
| complex_1028 | YBR146W \| YBR251W \| YHL004W \| YGR215W \| YDR036C \| YDR041W \| YDR175C \| YDR347W \| YGL129C \| YIL093C \| YJR101W \| YJR113C \| YKL155C \| YNL137C \| YPL118W |
| complex_1029 | YAL035W \| YMR260C \| YOR361C \| YPR041W \| YBR079C \| YLR192C \| YDR429C \| YER025W \| YMR146C \| YGR102C \| YLR241W \| YMR309C \| YOL087C \| YNL244C \| YPL237W |
| complex_103 | YCR052W \| YFR037C \| YGR056W \| YLR033W \| YLR357W \| YMR033W \| YDR303C \| YGR275W \| YIL126W \| YKR008W \| YML127W \| YMR091C \| YPR034W \| YGL184C \| YMR224C |
| complex_1031 | YAL035W \| YMR260C \| YOR361C \| YPR041W \| YBR079C \| YLR192C \| YDR429C \| YER025W \| YMR146C \| YLR039C \| YMR309C \| YOL087C \| YNL244C \| YLR359W \| YPL237W |
| complex_1032 | YBR154C \| YDL140C \| YGR005C \| YPL129W \| YBR157C \| YGL070C \| YDR056C \| YHR114W \| YDR404C \| YJL140W \| YOL005C \| YGR104C \| YIL021W \| YJL168C \| YOR224C |
| complex_1033 | YCL024W \| YNL078W \| YCR002C \| YDR507C \| YDR510W \| YHR107C \| YLR314C \| YNL166C \| YNL298W \| YDL064W \| YDR409W \| YJL092W \| YOR156C \| YDL225W \| YJR076C |
| complex_1035 | YBL026W \| YER172C \| YLR275W \| YMR268C \| YNL147W \| YDR378C \| YER146W \| YOL149W \| YER112W \| YGL251C \| YNL127W \| YJL124C \| YJR022W \| YLR438C-A \| YPL151C |
| complex_1038 | YBL023C \| YBR202W \| YCL061C \| YGL201C \| YIL150C \| YJL194W \| YLR103C \| YLR274W \| YMR048W \| YDL017W \| YEL032W \| YNL273W \| YPR019W \| YJL091C \| YER087C-A |
| complex_104 | YBL093C \| YDR308C \| YGL025C \| YOL135C \| YBR193C \| YBR253W \| YDL005C \| YGR104C \| YHR058C \| YLR071C \| YNR010W \| YOR174W \| YPR070W \| YGL151W \| YPR048W |
| complex_1044 | YBL023C \| YBR202W \| YCL061C \| YGL201C \| YIL150C \| YJL194W \| YLR103C \| YLR274W \| YMR048W \| YDL017W \| YEL032W \| YNL273W \| YPR019W \| YPL113C \| YDR143C |
| complex_1045 | YAL011W \| YDR334W \| YGR002C \| YIR018W \| YJL081C \| YDL002C \| YFL039C \| YGL150C \| YPL129W \| YPL235W \| YDR190C \| YNL215W \| YNL059C \| YNL107W \| YOR141C |

| | |
|---|---|
| complex_1046 | YBR202W \| YBL023C \| YMR048W \| YCL061C \| YLR103C \| YDL017W \| YGL201C \| YLR274W \| YEL032W \| YIL150C \| YNL273W \| YPR019W \| YJL194W \| YER063W \| YPL172C |
| complex_1048 | YDL209C \| YER029C \| YHR165C \| YDR088C \| YER013W \| YER172C \| YGR006W \| YGR074W \| YKR086W \| YLL036C \| YLR433C \| YMR001C \| YMR213W \| YPR101W \| YLR147C |
| complex_1049 | YAL009W \| YDR211W \| YBR079C \| YMR309C \| YPR041W \| YKR026C \| YER025W \| YGR083C \| YJR007W \| YLR291C \| YOR260W \| YIL131C \| YJL010C \| YNL265C \| YPL237W |
| complex_105 | YDL007W \| YDR363W-A \| YFR004W \| YFR052W \| YGL004C \| YGL048C \| YHR027C \| YDL097C \| YDL147W \| YDR394W \| YHR200W \| YKL145W \| YOR117W \| YOR259C \| YKL010C |
| complex_1051 | YBR267W \| YHR170W \| YCR072C \| YDR101C \| YER006W \| YER126C \| YLR009W \| YLR074C \| YPL093W \| YGL099W \| YIR026C \| YLR325C \| YGR245C \| YHR197W \| YHR052W |
| complex_1052 | YBL023C \| YBR202W \| YCL061C \| YGL201C \| YIL150C \| YJL194W \| YLR103C \| YLR274W \| YMR048W \| YDL017W \| YEL032W \| YNL273W \| YPR019W \| YER088C \| YGL113W |
| complex_1053 | YBL026W \| YCR077C \| YNL147W \| YNL145W \| YDR378C \| YER146W \| YOL149W \| YER112W \| YHL008C \| YGL143C \| YER107C \| YGL173C \| YJL124C \| YLR438C-A \| YJR022W |
| complex_1054 | YBR211C \| YAL034W-A \| YGR179C \| YKL049C \| YPL233W \| YDL035C \| YPL268W \| YDR254W \| YGR140W \| YGR113W \| YJR060W \| YMR168C \| YIR010W \| YMR094W \| YPL018W |
| complex_1055 | YCR057C \| YDR449C \| YJR002W \| YLR129W \| YMR093W \| YOR310C \| YDR324C \| YER082C \| YLR222C \| YJL069C \| YKL099C \| YLR409C \| YML093W \| YPL157W \| YNR004W |
| complex_1056 | YAL034W-A \| YBR211C \| YDR254W \| YIR010W \| YPL018W \| YPR046W \| YBR107C \| YGR179C \| YJR135C \| YLR381W \| YEL077C \| YFR053C \| YIL111W \| Q0045 \| YPL233W |
| complex_1057 | YAL030W \| YER143W \| YGR009C \| YPR032W \| YBL050W \| YDR468C \| YMR197C \| YOL018C \| YPL232W \| YDR122W \| YDR164C \| YOR327C \| YGL095C \| YMR183C \| YNR049C |
| complex_1059 | YAL025C \| YGR103W \| YLR009W \| YDR087C \| YKR081C \| YNL061W \| YPL093W \| YER002W \| YMR049C \| YHR052W \| YLR325C \| YOL077C \| YHR066W \| YIL085C \| YOR272W |
| complex_106 | YBL093C \| YDR308C \| YGL025C \| YOL135C \| YPR168W \| YBR193C \| YBR253W \| YDL005C \| YGR104C \| YLR071C \| YMR112C \| YNR010W \| YOR174W \| YPR070W \| YPL276W |
| complex_1060 | YBR181C \| YJR002W \| YCR057C \| YDR449C \| YLR129W \| YMR093W \| YOR310C \| YDR324C \| YER082C \| YLR222C \| YJL069C \| YJL191W \| YKL099C \| YLR409C \| YML093W |
| complex_1061 | YBR020W \| YML051W \| YCL011C \| YDL084W \| YDR138W \| YDR381W \| YHR167W \| YKL139W \| YNL112W \| YNL253W \| YDR009W \| YML062C \| YNL004W \| YNL139C \| YPL248C |
| complex_1062 | YBR279W \| YDL140C \| YGL244W \| YLR418C \| YOL145C \| YOR123C \| YGL019W \| YGL207W \| YGR063C \| YGR116W \| YIL035C \| YML010W \| YML069W \| YHR009C \| YOR074C |

| | |
|---|---|
| complex_1063 | YBR279W \| YDL140C \| YGL244W \| YLR418C \| YOL145C \| YOR123C \| YGL019W \| YGL207W \| YGR063C \| YGR116W \| YIL035C \| YML010W \| YML069W \| YBR195C \| YNL183C |
| complex_1065 | YCR035C \| YHR034C \| YPL211W \| YCR072C \| YDR101C \| YER006W \| YER126C \| YLR009W \| YPL093W \| YKR081C \| YGR245C \| YHR197W \| YPL146C \| YHR052W \| YAL026C |
| complex_1067 | YBL002W \| YDR225W \| YGL150C \| YGL241W \| YKR048C \| YBL003C \| YBR009C \| YBR010W \| YDR224C \| YGR252W \| YJL081C \| YOL012C \| YKL089W \| YOL054W \| YOR141C |
| complex_1068 | YAL056W \| YDR138W \| YER020W \| YGL121C \| YCL011C \| YDL084W \| YDR381W \| YHR167W \| YKL139W \| YNL112W \| YNL253W \| YKL214C \| YML062C \| YNL004W \| YNL139C |
| complex_107 | YDL097C \| YDL147W \| YDR363W-A \| YDR394W \| YER021W \| YFR004W \| YFR052W \| YGL004C \| YHR027C \| YHR200W \| YKL145W \| YOR261C \| YPR108W \| YDR427W \| YML057W |
| complex_1070 | YBR131W \| YDL077C \| YGL124C \| YOR106W \| YDR080W \| YLR148W \| YAL002W \| YDR323C \| YLR396C \| YMR291W \| YML001W \| YMR231W \| YPL045W \| YPL195W \| YPL262W |
| complex_1071 | YBR079C \| YDR429C \| YLR192C \| YMR146C \| YMR309C \| YOL087C \| YPR041W \| YDR504C \| YJL070C \| YER025W \| YNL244C \| YOR361C \| YPL105C \| YPL237W \| YPR086W |
| complex_1072 | YBR154C \| YJL148W \| YJR063W \| YNL113W \| YPR010C \| YPR110C \| YDL042C \| YJL076W \| YDR110W \| YFR028C \| YOR210W \| YNL248C \| YOR340C \| YOR341W \| YPR187W |
| complex_1073 | YBL049W \| YCL039W \| YBR154C \| YOR116C \| YPR110C \| YDL150W \| YKR025W \| YDR045C \| YOR207C \| YDR255C \| YJL011C \| YKL144C \| YNL151C \| YNR003C \| YPR190C |
| complex_1075 | YCR063W \| YDL209C \| YLL036C \| YMR001C \| YMR213W \| YPR101W \| YDR163W \| YDR416W \| YGR129W \| YGL174W \| YIR005W \| YLR016C \| YGL216W \| YJR050W \| YPL151C |
| complex_1076 | YBR131W \| YDL077C \| YGL124C \| YOR106W \| YDR080W \| YLR148W \| YKL025C \| YGL094C \| YAL002W \| YDR323C \| YLR396C \| YML001W \| YMR231W \| YPL045W \| YPL195W |
| complex_1077 | YBR131W \| YDL077C \| YGL124C \| YOR106W \| YDR080W \| YDR251W \| YPL057C \| YLR148W \| YAL002W \| YDR323C \| YLR396C \| YML001W \| YMR231W \| YPL045W \| YPL195W |
| complex_1078 | YBR087W \| YFR027W \| YOR144C \| YBR088C \| YOR217W \| YER173W \| YIL139C \| YJR068W \| YNL290W \| YOL094C \| YPL194W \| YHR191C \| YPL167C \| YMR078C \| YOR146W |
| complex_1079 | YER164W \| YGL019W \| YOR039W \| YKL112W \| YLR079W \| YOL145C \| YGL207W \| YGL244W \| YOR061W \| YGR116W \| YIL035C \| YML010W \| YML069W \| YKL160W \| YKL036C |
| complex_1080 | YBL008W \| YOR038C \| YOR290C \| YBR289W \| YDR073W \| YEL009C \| YFL049W \| YHL025W \| YJL176C \| YKL109W \| YMR033W \| YNR023W \| YPL016W \| YPL129W \| YDR370C |
| complex_1081 | YDR323C \| YOR089C \| YDR425W \| YGL198W \| YER031C \| YFL038C \| YNL044W \| YNL263C \| YER136W \| YKR014C \| YLR262C \| YNL093W \| YFL005W \| YGR172C \| YPL280W |

| | |
|---|---|
| complex_1083 | YBL023C \| YBR202W \| YCL061C \| YGL201C \| YIL150C \| YLR103C \| YLR274W \| YMR048W \| YDL017W \| YEL032W \| YNL273W \| YPR019W \| YGL001C \| YER044C \| YGR060W |
| complex_1085 | YBL050W \| YDR468C \| YKL196C \| YLR026C \| YMR197C \| YOL018C \| YOR036W \| YOR106W \| YDR323C \| YGL095C \| YDR321W \| YGL212W \| YHL031C \| YKL006C-A \| YLR093C |
| complex_1087 | YCR057C \| YLR175W \| YOR310C \| YDL014W \| YDL208W \| YDR365C \| YGR159C \| YHR089C \| YHR196W \| YJL033W \| YKL014C \| YLR197W \| YNL124W \| YGL078C \| YHR072W-A |
| complex_1091 | YBR279W \| YDL140C \| YER012W \| YGL244W \| YLR418C \| YOL145C \| YOR123C \| YGL058W \| YOL080C \| YGL207W \| YGR063C \| YGR116W \| YIL035C \| YML010W \| YML069W |
| complex_1094 | YBR267W \| YHR170W \| YCR072C \| YDR101C \| YER006W \| YER126C \| YLR074C \| YPL093W \| YPR017C \| YGL099W \| YGR245C \| YHR197W \| YHR052W \| YJL122W \| YLR397C |
| complex_1099 | YBR154C \| YDL140C \| YGR005C \| YPL129W \| YDR404C \| YGL070C \| YJL140W \| YDR527W \| YOR210W \| YGR186W \| YIL021W \| YOR151C \| YJL168C \| YNL266W \| YOL131W |
| complex_110 | YDL232W \| YDR086C \| YER019C-A \| YER087C-B \| YGL022W \| YJL002C \| YLR378C \| YML019W \| YOR103C \| YOR254C \| YEL002C \| YBL105C \| YGL226C-A \| YMR149W \| YOR085W |
| complex_1100 | YCL059C \| YCR057C \| YDL148C \| YDR449C \| YJR002W \| YHR196W \| YDL014W \| YLL011W \| YML010W \| YOL102C \| YPR137W \| YPR144C \| YGR054W \| YMR229C \| YNL132W |
| complex_1104 | YAL064W \| YMR149W \| YDL232W \| YDR086C \| YGL022W \| YJL002C \| YLR378C \| YML019W \| YDR310C \| YOL068C \| YOR279C \| YEL002C \| YGL226C-A \| YKR011C \| YOR085W |
| complex_1105 | YBR234C \| YDL029W \| YIR006C \| YJR065C \| YKL013C \| YKL129C \| YLR370C \| YNR035C \| YOR181W \| YDR063W \| YGL239C \| YIL062C \| YBR109C \| YLR337C \| YMR109W |
| complex_1106 | YBR079C \| YPR041W \| YDR211W \| YKR026C \| YER025W \| YGR083C \| YJR007W \| YKL205W \| YLR215C \| YLR291C \| YOL142W \| YOR260W \| YIL131C \| YNL265C \| YPL237W |
| complex_1109 | YBR247C \| YDL148C \| YGR090W \| YHR203C \| YCL059C \| YCR057C \| YDR447C \| YIL069C \| YJL109C \| YJR145C \| YPR144C \| YLR441C \| YOR119C \| YML024W \| YPR137W |
| complex_1111 | YBR127C \| YDL185W \| YOR270C \| YEL051W \| YER072W \| YGR020C \| YHR039C-A \| YHR060W \| YLR447C \| YOR332W \| YPR036W \| YPL019C \| YJL012C \| YKL119C \| YMR054W |
| complex_1113 | YDL065C \| YGL153W \| YDR142C \| YDR244W \| YGR239C \| YHR160C \| YLR191W \| YNL214W \| YGR077C \| YMR026C \| YDR265W \| YJL210W \| YML035C \| YLR465C \| YPR071W |
| complex_1114 | YBR079C \| YDR429C \| YLR192C \| YMR146C \| YMR309C \| YPR041W \| YDL216C \| YOL117W \| YER025W \| YJR007W \| YIL071C \| YDR021W \| YNL244C \| YOR361C \| YPL237W |
| complex_1115 | YAL042W \| YAR002C-A \| YML067C \| YML130C \| YAL007C \| YGL200C \| YGR284C \| YIL004C \| YLR026C \| YML012W \| YDL195W \| YIL109C \| YLR208W \| YPR181C \| YPL218W |

| | |
|---|---|
| complex_1117 | YBL026W \| YCR077C \| YLR264W \| YDR378C \| YOL149W \| YEL015W \| YER112W \| YGL173C \| YJL124C \| YLR438C-A \| YNL118C \| YJR022W \| YLR269C \| YOR167C \| YJR091C |
| complex_1118 | YBL093C \| YDR308C \| YGL025C \| YOL135C \| YBR193C \| YER022W \| YGL127C \| YHR058C \| YLR071C \| YNR010W \| YOR174W \| YCR030C \| YMR132C \| YLL065W \| YJR126C |
| complex_1119 | YDL089W \| YPR028W \| YER136W \| YFL038C \| YKR014C \| YLR262C \| YNL093W \| YOR089C \| YFL005W \| YGL198W \| YGR172C \| YNL044W \| YNL263C \| YGR004W \| YLR324W |
| complex_1122 | YCL009C \| YER012W \| YER094C \| YMR108W \| YDL188C \| YFR050C \| YGL011C \| YGR135W \| YML092C \| YOL038W \| YOR157C \| YPR103W \| YJL001W \| YMR106C \| YNL128W |
| complex_1123 | YCL059C \| YCR057C \| YDR449C \| YHR148W \| YJR002W \| YMR093W \| YDR324C \| YJL109C \| YLR061W \| YJL153C \| YJL190C \| YLR441C \| YOR078W \| YPL126W \| YPR137W |
| complex_1124 | YBR234C \| YDL029W \| YIR006C \| YJR065C \| YKL013C \| YKL129C \| YLR241W \| YLR370C \| YNL040W \| YNR035C \| YOR181W \| YIL062C \| YBR109C \| YLR337C \| YMR109W |
| complex_1125 | YBR171W \| YLR292C \| YOR254C \| YDL232W \| YDR086C \| YER087C-B \| YJL002C \| YLR378C \| YML019W \| YOR103C \| YEL002C \| YIL162W \| YLR301W \| YOR085W \| YPL094C |
| complex_1127 | YDL116W \| YGL092W \| YDL207W \| YMR047C \| YDR192C \| YJR042W \| YKR082W \| YEL024W \| YGL100W \| YLR208W \| YGL172W \| YGR119C \| YHR036W \| YKL057C \| YKL068W |
| complex_1128 | YAL043C \| YGR156W \| YAR003W \| YKL018W \| YBR175W \| YBR258C \| YLR015W \| YDR140W \| YIL151C \| YDR469W \| YHR119W \| YKL059C \| YNL317W \| YPL138C \| YPR107C |
| complex_1129 | YBL023C \| YBR202W \| YCL061C \| YGL201C \| YIL150C \| YLR103C \| YLR274W \| YDL017W \| YEL032W \| YNL273W \| YPR019W \| YIR008C \| YBL035C \| YKL045W \| YNR052C |
| complex_1131 | YGR103W \| YKR081C \| YHR052W \| YDR194C \| YKL172W \| YLL008W \| YMR049C \| YNL061W \| YNL175C \| YNL230C \| YPL043W \| YPL093W \| YHR066W \| YHR180W \| YOR272W |
| complex_1132 | YBL093C \| YDR308C \| YGL025C \| YOL135C \| YBR134W \| YOR128C \| YBR193C \| YER022W \| YGL127C \| YHR058C \| YLR071C \| YNR010W \| YOR174W \| YLL065W \| YNL159C |
| complex_1133 | YAL007C \| YPL211W \| YDR060W \| YGR103W \| YKR081C \| YMR049C \| YNL061W \| YOL077C \| YDR087C \| YKL014C \| YMR138W \| YOR063W \| YHR052W \| YMR290C \| YHR066W |
| complex_1136 | YDL116W \| YGL092W \| YFR002W \| YMR047C \| YGL100W \| YLR208W \| YGL172W \| YGR119C \| YJR042W \| YKL057C \| YGR161C-C \| YML103C \| YOR142W-A \| YKL068W \| YKR082W |
| complex_1137 | YAL034W-A \| YER018C \| YGL093W \| YIL144W \| YIR010W \| YJR112W \| YOL069W \| YDR318W \| YMR117C \| YDR356W \| YDL028C \| YKL042W \| YFL008W \| YGL086W \| YKR054C |
| complex_1138 | YBR264C \| YER136W \| YGL198W \| YNL044W \| YNL263C \| YOR370C \| YER031C \| YFL038C \| YKR014C \| YNL093W \| YFL005W \| YGR172C \| YJL030W \| YJL031C \| YPR176C |

| | |
|---|---|
| complex_1139 | YAL043C \| YGR156W \| YAR003W \| YBR175W \| YBR258C \| YKL018W \| YLR015W \| YCR038C \| YPR122W \| YDR469W \| YHR119W \| YKL059C \| YNL317W \| YPL138C \| YPR107C |
| complex_1140 | YAL032C \| YDL209C \| YDR416W \| YKL095W \| YKL173W \| YLR117C \| YLR424W \| YMR213W \| YPR101W \| YDR247W \| YGL120C \| YHR161C \| YPL077C \| YDR489W \| YPL151C |
| complex_1145 | YBL050W \| YKL196C \| YMR197C \| YOR036W \| YOR106W \| YDR080W \| YDR323C \| YGL095C \| YDR321W \| YGL212W \| YLR093C \| YLR148W \| YLR396C \| YML001W \| YMR231W |
| complex_1146 | YDR271C \| YOR244W \| YDR359C \| YGR002C \| YHR090C \| YDR509W \| YEL018W \| YFL024C \| YJL081C \| YKR021W \| YOL014W \| YNL107W \| YPR023C \| YNL136W \| YOR128C |
| complex_1147 | YDL007W \| YBR272C \| YDR363W-A \| YFR004W \| YFR052W \| YGL004C \| YDL097C \| YDR394W \| YER021W \| YHR200W \| YOR261C \| YEL034W \| YMR284W \| YEL019C \| YFL022C |
| complex_1148 | YBR154C \| YJL148W \| YJR063W \| YNL113W \| YOR116C \| YPR010C \| YPR110C \| YKL144C \| YNL248C \| YOR340C \| YKL213C \| YOR341W \| YPR187W \| YDR143C \| YKL067W |
| complex_1151 | YBR142W \| YLR009W \| YMR049C \| YNL061W \| YPL093W \| YER133W \| YGL111W \| YGR103W \| YLR449W \| YOR227W \| YKR081C \| YHR052W \| YHR066W \| YHR051W \| YOR272W |
| complex_1152 | YCR063W \| YDL209C \| YLL036C \| YMR001C \| YMR213W \| YPR101W \| YDR163W \| YDR341C \| YIR005W \| YDR416W \| YGR129W \| YGL174W \| YJR050W \| YMR057C \| YPL151C |
| complex_1155 | YBR142W \| YLR009W \| YMR049C \| YNL061W \| YPL093W \| YDR412W \| YER133W \| YGL111W \| YGR103W \| YLR449W \| YGL023C \| YKR081C \| YHR052W \| YHR066W \| YOR272W |
| complex_1156 | YAL034W-A \| YER018C \| YGL093W \| YIL144W \| YOL069W \| YDR016C \| YDR201W \| YGL061C \| YGR113W \| YKR037C \| YKR083C \| YDR356W \| YER016W \| YMR117C \| YKL052C |
| complex_1157 | YER164W \| YGL019W \| YOR039W \| YOL145C \| YGL207W \| YGL244W \| YGR063C \| YOR061W \| YGR116W \| YIL035C \| YML010W \| YML069W \| YLR410W \| YPR052C \| YOR292C |
| complex_1161 | YBR048W \| YBR247C \| YCL059C \| YDL148C \| YHR196W \| YGR090W \| YHR203C \| YCR057C \| YDR074W \| YAR066W \| YJL109C \| YJR145C \| YPR144C \| YLR441C \| YPR137W |
| complex_1162 | YAL030W \| YGR009C \| YPR032W \| YBR102C \| YPR055W \| YDR027C \| YGL233W \| YLR166C \| YDR164C \| YPL232W \| YDR166C \| YJL085W \| YER008C \| YNR049C \| YIL068C |
| complex_1165 | YBL004W \| YDL148C \| YHR089C \| YCL059C \| YCR057C \| YDL213C \| YDL014W \| YHR196W \| YJL109C \| YOL010W \| YPR137W \| YPR144C \| YEL055C \| YER100W \| YPL217C |
| complex_1166 | YER126C \| YGR103W \| YKR081C \| YLR009W \| YLR074C \| YNR053C \| YGL099W \| YIR026C \| YNL002C \| YPL093W \| YPL131W \| YGR245C \| YDR034C \| YKL180W \| YPR016C |
| complex_1167 | YBR193C \| YBR253W \| YDL140C \| YER022W \| YGR104C \| YHR041C \| YHR058C \| YLR071C \| YOL051W \| YPL129W \| YGR005C \| YOL042W \| YPR056W \| YLR172C \| YOR159C |

| | |
|---|---|
| complex_123 | YBL093C \| YCR081W \| YGL025C \| YOL135C \| YBR193C \| YBR253W \| YDL005C \| YGR104C \| YLR071C \| YNL236W \| YNR010W \| YOL051W \| YPR070W \| YGL151W \| YNL025C |
| complex_125 | YDL031W \| YER006W \| YGR103W \| YKR081C \| YDR101C \| YLR009W \| YLR074C \| YER126C \| YNL110C \| YGR245C \| YHR197W \| YNL002C \| YPL093W \| YHR052W \| YPR016C |
| complex_127 | YBR119W \| YDR235W \| YDR240C \| YFL017W-A \| YGR013W \| YHR086W \| YIL061C \| YKL012W \| YLL036C \| YLR298C \| YML046W \| YMR125W \| YDL087C \| YGR074W \| YNL210W |
| complex_128 | YCL059C \| YCR057C \| YDR449C \| YGR090W \| YJR002W \| YLR129W \| YMR093W \| YDR324C \| YJL109C \| YER082C \| YLR222C \| YPL126W \| YJL069C \| YLR409C \| YPR144C |
| complex_129 | YBL022C \| YCR052W \| YML029W \| YMR091C \| YCR020W-B \| YFR037C \| YIL126W \| YLR033W \| YLR357W \| YMR033W \| YDR303C \| YGR275W \| YKR008W \| YML127W \| YPR034W |
| complex_130 | YCR020W-B \| YFR037C \| YIL126W \| YLR033W \| YMR091C \| YCR052W \| YLR357W \| YMR033W \| YPR052C \| YDR303C \| YGR275W \| YKR008W \| YML127W \| YPR034W \| YGL071W |
| complex_136 | YCR072C \| YDR101C \| YER006W \| YER126C \| YLR009W \| YLR074C \| YLR106C \| YNL182C \| YPL093W \| YGR103W \| YNL110C \| YGR245C \| YHR197W \| YHR052W \| YHR085W |
| complex_140 | YBR198C \| YCR042C \| YDR145W \| YDR167W \| YGL112C \| YGR274C \| YML114C \| YMR005W \| YMR227C \| YMR236W \| YPL011C \| YPL254W \| YML015C \| YML098W \| YGR208W |
| complex_141 | YAL043C \| YGR156W \| YDR195W \| YJR093C \| YKL018W \| YKL059C \| YKR002W \| YLR115W \| YLR277C \| YNL222W \| YNL317W \| YPL008W \| YPR107C \| YMR260C \| YOL150C |
| complex_142 | YDL040C \| YDL097C \| YDL147W \| YER021W \| YGL048C \| YIL075C \| YOR261C \| YDR363W-A \| YFR004W \| YFR052W \| YGL004C \| YHR027C \| YHR200W \| YKL145W \| YLR421C |
| complex_143 | YAL043C \| YGR156W \| YDR195W \| YJR093C \| YKL018W \| YKL059C \| YKR002W \| YLR115W \| YLR277C \| YNL222W \| YNL317W \| YPL008W \| YPR107C \| YJL072C \| YOR344C |
| complex_147 | YAL032C \| YDL209C \| YDR416W \| YGL128C \| YKL095W \| YKL173W \| YLL036C \| YLR117C \| YLR424W \| YMR001C \| YMR213W \| YPR101W \| YHR165C \| YGL120C \| YPL151C |
| complex_150 | YAL034W-A \| YBR211C \| YDR254W \| YIR010W \| YPL018W \| YPR046W \| YBR107C \| YDR383C \| YGR179C \| YJR135C \| YLR381W \| YDR318W \| YKL089W \| YKL049C \| YPL233W |
| complex_155 | YBR081C \| YBR111W-A \| YDR167W \| YDR176W \| YMR223W \| YMR236W \| YDR145W \| YDR392W \| YOL148C \| YPL047W \| YPL254W \| YPL181W \| YDR448W \| YLR055C \| YPL139C |
| complex_156 | YCR052W \| YFR037C \| YGR056W \| YLR033W \| YLR321C \| YLR357W \| YMR033W \| YDR303C \| YGR275W \| YIL126W \| YKR008W \| YMR091C \| YPR034W \| YLR345W \| YKL174C |
| complex_157 | YBR198C \| YCR042C \| YDR145W \| YDR167W \| YER148W \| YGR274C \| YKL058W \| YML114C \| YMR005W \| YMR227C \| YMR236W \| YPL011C \| YOR194C \| YML015C \| YML098W |

| | |
|---|---|
| complex_158 | YDL097C \| YDL147W \| YDR363W-A \| YER012W \| YFR004W \| YGL004C \| YGL048C \| YHR027C \| YHR200W \| YIL075C \| YKL145W \| YLR421C \| YOR261C \| YDR427W \| YGR232W |
| complex_162 | YBL084C \| YDL008W \| YDR118W \| YDR260C \| YFR036W \| YGL240W \| YHR166C \| YIR025W \| YKL022C \| YLR102C \| YLR127C \| YJR091C \| YNL176C \| YDL135C \| YOR249C |
| complex_166 | YCL059C \| YCR057C \| YDR449C \| YGR090W \| YJR002W \| YMR093W \| YOR310C \| YDR324C \| YJL109C \| YER082C \| YPL126W \| YGL171W \| YGR128C \| YLR409C \| YOR078W |
| complex_168 | YAL032C \| YDL209C \| YDR364C \| YDR416W \| YKL095W \| YKL173W \| YLL036C \| YLR117C \| YLR424W \| YMR001C \| YMR213W \| YPR101W \| YBR065C \| YDR088C \| YPL151C |
| complex_170 | YDL232W \| YDR086C \| YER019C-A \| YER087C-B \| YGL022W \| YJL002C \| YLR378C \| YML019W \| YOR103C \| YMR149W \| YEL002C \| YGL226C-A \| YML055W \| YJR010C-A \| YOR085W |
| complex_171 | YBR065C \| YDL209C \| YLL036C \| YMR001C \| YMR213W \| YPR101W \| YBR188C \| YDR416W \| YGR129W \| YCR063W \| YHR165C \| YDR163W \| YJR050W \| YLR117C \| YPL151C |
| complex_173 | YER126C \| YER006W \| YGR103W \| YKR081C \| YLR009W \| YMR049C \| YMR290C \| YNL061W \| YNL110C \| YGL111W \| YHR088W \| YPL093W \| YHR066W \| YPR016C \| YOR272W |
| complex_174 | YDL007W \| YBR272C \| YDR363W-A \| YFR004W \| YFR052W \| YGL004C \| YGL048C \| YHR027C \| YDL097C \| YDL147W \| YDR394W \| YKL145W \| YOR117W \| YOR259C \| YNL311C |
| complex_175 | YCR035C \| YDR280W \| YGR095C \| YGR195W \| YOL021C \| YOR001W \| YDL111C \| YGR158C \| YHR069C \| YNL232W \| YOL142W \| YHR081W \| YLR163C \| YHR120W \| YOR076C |
| complex_176 | YCR035C \| YDR280W \| YGR095C \| YGR195W \| YOL021C \| YOR001W \| YDL111C \| YGR158C \| YHR069C \| YNL232W \| YOL142W \| YHR081W \| YNL265C \| YGR194C \| YOR076C |
| complex_180 | YBR089C-A \| YFR037C \| YLR033W \| YLR321C \| YPR052C \| YCR052W \| YLR357W \| YMR033W \| YDR303C \| YGR275W \| YIL126W \| YKR008W \| YMR091C \| YPR034W \| YGL071W |
| complex_181 | YAL034W-A \| YBR211C \| YDR254W \| YGL093W \| YIR010W \| YJR112W \| YPL018W \| YPR046W \| YBR107C \| YGR179C \| YLR381W \| YDR318W \| YKL089W \| YKL049C \| YPL233W |
| complex_182 | YBR081C \| YBR111W-A \| YDR176W \| YMR223W \| YBR198C \| YDR145W \| YGL112C \| YGR252W \| YLR055C \| YPL047W \| YCL010C \| YDR448W \| YOL072W \| YGL066W \| YDR229W |
| complex_183 | YCR035C \| YDR280W \| YGR095C \| YGR195W \| YOL021C \| YOR001W \| YDL111C \| YGR158C \| YHR069C \| YNL232W \| YPR189W \| YGL213C \| YHR081W \| YLR398C \| YOR076C |
| complex_186 | YCR035C \| YDR280W \| YGR095C \| YGR195W \| YOL021C \| YOR001W \| YDL111C \| YGR158C \| YHR069C \| YNL232W \| YOL142W \| YHR081W \| YLR345W \| YKL174C \| YOR076C |
| complex_188 | YCR035C \| YDR280W \| YGR095C \| YGR195W \| YHR034C \| YOL021C \| YOR001W \| YCR060W \| YDL111C \| YGR158C \| YHR069C \| YNL232W \| YOL142W \| YHR081W \| YOR076C |

| | |
|---|---|
| complex_189 | YBR081C \| YBR111W-A \| YDR176W \| YMR223W \| YBR198C \| YDR145W \| YGL112C \| YGR252W \| YLR055C \| YPL047W \| YCL010C \| YDR448W \| YGL066W \| YGL252C \| YNL076W |
| complex_19 | YAL043C \| YGR156W \| YDR195W \| YDR301W \| YER133W \| YJR093C \| YKL018W \| YKL059C \| YKR002W \| YLR115W \| YLR277C \| YNL222W \| YNL317W \| YPR107C \| YBR205W |
| complex_196 | YAL064W \| YMR149W \| YDL232W \| YDR086C \| YER019C-A \| YER087C-B \| YGL022W \| YJL002C \| YLR378C \| YML019W \| YOR103C \| YEL002C \| YGL226C-A \| YOR279C \| YOR085W |
| complex_197 | YDL232W \| YDR086C \| YER019C-A \| YER087C-B \| YGL022W \| YJL002C \| YLR378C \| YML019W \| YOR103C \| YDR021W \| YML130C \| YMR149W \| YEL002C \| YGL226C-A \| YOR085W |
| complex_198 | YCR057C \| YDR449C \| YJR002W \| YLR129W \| YMR093W \| YOR310C \| YDR324C \| YJL109C \| YER082C \| YLR222C \| YPL126W \| YGR128C \| YJL069C \| YKL099C \| YLR409C |
| complex_199 | YBR081C \| YBR111W-A \| YDR176W \| YMR223W \| YBR198C \| YDR145W \| YGL112C \| YGR252W \| YPL047W \| YCL010C \| YDR159W \| YDR448W \| YOL072W \| YOR257W \| YGL066W |
| complex_20 | YBL093C \| YDR308C \| YGL025C \| YOL135C \| YPL129W \| YPR168W \| YBR193C \| YBR253W \| YDL005C \| YGR104C \| YHR058C \| YLR071C \| YNR010W \| YOR174W \| YPR070W |
| complex_202 | YBL093C \| YDR308C \| YGL025C \| YOL135C \| YBR193C \| YBR253W \| YDL005C \| YGR104C \| YHR058C \| YLR071C \| YNR010W \| YOR174W \| YPR070W \| YCL016C \| YKL023W |
| complex_204 | YBL004W \| YDL148C \| YGR090W \| YJR002W \| YBR247C \| YCL059C \| YCR057C \| YOL010W \| YDR324C \| YJL109C \| YMR128W \| YOR078W \| YPR144C \| YPL217C \| YPR137W |
| complex_205 | YAL011W \| YDR334W \| YJL081C \| YOL012C \| YBR231C \| YDR190C \| YGR002C \| YLR385C \| YML041C \| YPL235W \| YDR485C \| YFL024C \| YFL039C \| YLR399C \| YNL107W |
| complex_208 | YAL043C \| YGL044C \| YGR156W \| YMR039C \| YDR195W \| YDR301W \| YJR093C \| YKL059C \| YKR002W \| YLR115W \| YLR277C \| YMR061W \| YNL317W \| YDR228C \| YMR316C-B |
| complex_21 | YBR081C \| YBR111W-A \| YDR167W \| YDR176W \| YMR223W \| YMR236W \| YBR198C \| YDR145W \| YGL112C \| YGR252W \| YLR055C \| YPL047W \| YCL010C \| YDR448W \| YGL066W |
| complex_210 | YDR359C \| YGR002C \| YHR090C \| YJL098W \| YJR082C \| YEL018W \| YFL024C \| YJL081C \| YOR244W \| YFL039C \| YHR099W \| YOL012C \| YNL107W \| YPR023C \| YNL136W |
| complex_220 | YBL093C \| YDR308C \| YGL025C \| YOL135C \| YPR168W \| YBR193C \| YBR253W \| YGR104C \| YHR058C \| YLR071C \| YNR010W \| YOR174W \| YPR070W \| YKL157W \| YPL276W |
| complex_228 | YBR081C \| YBR111W-A \| YDR167W \| YDR176W \| YMR223W \| YMR236W \| YBR198C \| YDR145W \| YGR252W \| YLR055C \| YPL047W \| YCL010C \| YDL006W \| YDR448W \| YIR023W |
| complex_229 | YBL004W \| YDL148C \| YGR090W \| YJR002W \| YBR247C \| YHR203C \| YCL059C \| YCR057C \| YHR196W \| YDR064W \| YJL109C \| YJR145C \| YPR144C \| YPL217C \| YPR137W |

| | |
|---|---|
| complex_230 | YDL007W \| YDR363W-A \| YFR004W \| YFR052W \| YGL048C \| YHR027C \| YDL097C \| YDR394W \| YHR200W \| YKL145W \| YOR261C \| YDL227C \| YER143W \| YDR427W \| YEL037C |
| complex_233 | YCR035C \| YDR280W \| YGR095C \| YGR195W \| YNL189W \| YOL021C \| YOR001W \| YDL111C \| YGR158C \| YHR069C \| YNL232W \| YHR081W \| YLR447C \| YIR035C \| YOR076C |
| complex_241 | YBL093C \| YDR308C \| YGL025C \| YOL135C \| YBR193C \| YBR253W \| YGR104C \| YHR058C \| YLR071C \| YMR112C \| YNR010W \| YOR174W \| YPR070W \| YJR110W \| YDR425W |
| complex_242 | YDL007W \| YDR363W-A \| YFR004W \| YFR052W \| YGL048C \| YHR027C \| YDL097C \| YDL147W \| YDR394W \| YHR200W \| YKL145W \| YOR261C \| YPR131C \| YDR427W \| YIR011C |
| complex_244 | YBR154C \| YDL140C \| YGR005C \| YPL129W \| YDR404C \| YGL070C \| YJL140W \| YOL005C \| YPR093C \| YGR104C \| YIL021W \| YJL168C \| YOR151C \| YOR224C \| YPR187W |
| complex_245 | YBL093C \| YDR308C \| YGL025C \| YOL135C \| YBR193C \| YBR253W \| YGR104C \| YHR058C \| YLR071C \| YMR112C \| YNR010W \| YOR174W \| YPR070W \| YDR267C \| YIL128W |
| complex_250 | YDL097C \| YDL147W \| YDR363W-A \| YDR394W \| YFR052W \| YGL048C \| YHR027C \| YHR200W \| YKL145W \| YOR117W \| YOR259C \| YDR427W \| YMR276W \| YEL037C \| YPL096W |
| complex_254 | YBR119W \| YDR235W \| YDR240C \| YFL017W-A \| YGR013W \| YHR086W \| YIL061C \| YKL012W \| YLL036C \| YLR298C \| YML046W \| YMR125W \| YDL087C \| YLR116W \| YIL130W |
| complex_258 | YBL084C \| YDR118W \| YDR260C \| YFR036W \| YGL116W \| YGL240W \| YHR166C \| YIR025W \| YKL022C \| YLR127C \| YNL172W \| YDR113C \| YIL005W \| YGR225W \| YOR249C |
| complex_260 | YBR055C \| YBR152W \| YER172C \| YFL017W-A \| YGR091W \| YHR165C \| YLL036C \| YLR438C-A \| YNL147W \| YOR308C \| YPR082C \| YPR178W \| YDR473C \| YKL173W \| YJR022W |
| complex_261 | YBL022C \| YCR052W \| YMR091C \| YFR037C \| YIL084C \| YLR033W \| YLR321C \| YLR357W \| YMR033W \| YNL202W \| YDR303C \| YGR275W \| YIL126W \| YKR008W \| YPR034W |
| complex_263 | YCR072C \| YER006W \| YER126C \| YLR074C \| YPL093W \| YDR101C \| YGR245C \| YHR197W \| YKR081C \| YLR009W \| YFL002C \| YJR082C \| YNL002C \| YHR052W \| YNL110C |
| complex_265 | YBR065C \| YDL209C \| YLL036C \| YLR424W \| YMR213W \| YPR101W \| YBR188C \| YDR416W \| YGR129W \| YDL144C \| YDR088C \| YHR165C \| YJR050W \| YLR117C \| YPL151C |
| complex_267 | YCR052W \| YFR037C \| YGR056W \| YLR321C \| YLR357W \| YMR033W \| YDR303C \| YGR275W \| YIL126W \| YKR008W \| YMR091C \| YPR034W \| YJR031C \| YHR056C \| YFL067W |
| complex_268 | YAL025C \| YGR103W \| YLR009W \| YER002W \| YMR049C \| YPL093W \| YER126C \| YER006W \| YKR081C \| YNL110C \| YGR245C \| YNL002C \| YHR052W \| YHR066W \| YPR016C |
| complex_269 | YCR057C \| YDR449C \| YHR196W \| YJR002W \| YMR093W \| YDR324C \| YJL109C \| YER082C \| YEL055C \| YPL126W \| YGR128C \| YDR079C-A \| YPR144C \| YMR229C \| YPR137W |

| | |
|---|---|
| complex_270 | YER002W \| YGL237C \| YGR103W \| YLR009W \| YMR049C \| YPL093W \| YER126C \| YKR081C \| YNL110C \| YGR245C \| YNL002C \| YHR052W \| YHR066W \| YPR016C \| YOR272W |
| complex_271 | YBL093C \| YDR308C \| YGL025C \| YOL135C \| YBR193C \| YBR253W \| YGL127C \| YGR104C \| YHR058C \| YLR071C \| YNR010W \| YOR174W \| YPR070W \| YCR063W \| YDR408C |
| complex_272 | YER126C \| YER006W \| YGR103W \| YKR081C \| YLR009W \| YMR049C \| YMR290C \| YNL061W \| YNL110C \| YGR245C \| YNL002C \| YPL093W \| YKL009W \| YEL041W \| YPR016C |
| complex_273 | YCR042C \| YGL112C \| YML114C \| YMR005W \| YMR236W \| YDL070W \| YJL115W \| YLR399C \| YDR145W \| YMR227C \| YER148W \| YGR274C \| YML015C \| YML098W \| YPL011C |
| complex_276 | YBR119W \| YDR240C \| YFL017W-A \| YGR013W \| YHR086W \| YIL061C \| YKL012W \| YLL036C \| YLR298C \| YML046W \| YMR125W \| YDL087C \| YLR275W \| YOR159C \| YEL049W |
| complex_277 | YCR072C \| YDR101C \| YER006W \| YER126C \| YLR009W \| YLR074C \| YPL093W \| YGR103W \| YKR081C \| YLL045C \| YNL110C \| YGR245C \| YHR197W \| YHR052W \| YOL041C |
| complex_278 | YCR035C \| YDR280W \| YGR095C \| YGR195W \| YOL021C \| YOR001W \| YDL111C \| YGR158C \| YHR069C \| YNL232W \| YDR083W \| YOL142W \| YFL046W \| YMR131C \| YHR081W |
| complex_280 | YBR173C \| YGL011C \| YPR103W \| YDL188C \| YER012W \| YER094C \| YFR050C \| YGR135W \| YMR314W \| YOL038W \| YOR157C \| YOR362C \| YHR200W \| YJL001W \| YML092C |
| complex_283 | YBR154C \| YDL140C \| YGR005C \| YPL129W \| YDR404C \| YGL070C \| YJL140W \| YOL005C \| YGR104C \| YGR186W \| YIL021W \| YOR151C \| YJL168C \| YHL021C \| YPR187W |
| complex_284 | YDL097C \| YDL147W \| YDR363W-A \| YER021W \| YFR004W \| YFR052W \| YHR027C \| YHR200W \| YIL075C \| YKL145W \| YOR261C \| YDR427W \| YFR010W \| YGL141W \| YKL021C |
| complex_289 | YAL011W \| YDR334W \| YGR002C \| YJL081C \| YOL012C \| YBR231C \| YDR190C \| YFL039C \| YLR085C \| YLR385C \| YML041C \| YPL235W \| YDL237W \| YDR485C \| YNL107W |
| complex_292 | YAL032C \| YDL209C \| YDR364C \| YDR416W \| YGR278W \| YKL095W \| YKL173W \| YLR117C \| YLR424W \| YMR001C \| YMR213W \| YPR101W \| YHR165C \| YGR049W \| YPL151C |
| complex_3 | YAL043C \| YGR156W \| YDR195W \| YDR301W \| YJR093C \| YKL018W \| YKL059C \| YKR002W \| YLR115W \| YLR277C \| YNL222W \| YNL317W \| YOR179C \| YPL008W \| YPR107C |
| complex_302 | YAL032C \| YDL209C \| YDR416W \| YGL128C \| YKL095W \| YKL173W \| YLR117C \| YLR424W \| YMR213W \| YPR101W \| YBR190W \| YHR165C \| YGL120C \| YNL245C \| YPL151C |
| complex_305 | YBR171W \| YLR292C \| YOR254C \| YDL232W \| YDR086C \| YER087C-B \| YGL022W \| YJL002C \| YLR378C \| YML019W \| YOR103C \| YEL002C \| YMR149W \| YOR085W \| YPL094C |
| complex_306 | YAL005C \| YIL016W \| YPL106C \| YCR052W \| YFR037C \| YLR033W \| YLR321C \| YLR357W \| YMR033W \| YDL229W \| YDR303C \| YGR275W \| YIL126W \| YKR008W \| YMR091C |

| | |
|---|---|
| complex_308 | YBR081C \| YBR111W-A \| YMR223W \| YMR236W \| YDR145W \| YDR392W \| YOL148C \| YPL047W \| YPL254W \| YDR448W \| YGR252W \| YHR079C \| YLR055C \| YFL031W \| YER089C |
| complex_310 | YBL093C \| YDR308C \| YGL025C \| YOL135C \| YBR193C \| YER022W \| YGL127C \| YGR104C \| YHR058C \| YLR071C \| YNR010W \| YOR174W \| YPR070W \| YLL065W \| YBL009W |
| complex_314 | YBL026W \| YCR077C \| YDL160C \| YLL036C \| YMR268C \| YNL147W \| YDR378C \| YER146W \| YOL149W \| YOR320C \| YER112W \| YGL173C \| YJL124C \| YLR438C-A \| YJR022W |
| complex_315 | YBR231C \| YDR190C \| YDR334W \| YFL039C \| YGR002C \| YJL081C \| YML041C \| YOL012C \| YPL235W \| YDL002C \| YGL150C \| YPL129W \| YNL215W \| YNL107W \| YOR141C |
| complex_318 | YBR247C \| YDL148C \| YGR090W \| YHR203C \| YCL059C \| YCR057C \| YHR196W \| YOR310C \| YDR064W \| YJL109C \| YDR447C \| YML024W \| YJR145C \| YPR144C \| YPR137W |
| complex_319 | YCR057C \| YDR449C \| YGR090W \| YJR002W \| YLR129W \| YMR093W \| YDR324C \| YJL109C \| YER082C \| YPL126W \| YGR128C \| YJL069C \| YLR409C \| YMR242C \| YNR054C |
| complex_320 | YAL043C \| YGR156W \| YDR228C \| YDR301W \| YNL222W \| YKL018W \| YIL035C \| YHL035C \| YKL059C \| YKR002W \| YLR277C \| YNL317W \| YLR115W \| YDL094C \| YPR107C |
| complex_321 | YBL084C \| YDL008W \| YDR118W \| YDR260C \| YGL240W \| YHR166C \| YIR025W \| YKL022C \| YLR102C \| YLR127C \| YKR055W \| YDL135C \| YMR092C \| YNL176C \| YOR249C |
| complex_322 | YBL050W \| YDR189W \| YKL196C \| YLR026C \| YLR268W \| YMR197C \| YOR036W \| YOR106W \| YOR327C \| YGL212W \| YHL031C \| YIL004C \| YKL006C-A \| YLR078C \| YLR093C |
| complex_325 | YBR127C \| YDL185W \| YDR328C \| YOR270C \| YDR202C \| YEL051W \| YGR020C \| YHR039C-A \| YHR060W \| YLR447C \| YOR332W \| YPR036W \| YJR033C \| YKL080W \| YMR054W |
| complex_331 | YAL043C \| YGR156W \| YDR195W \| YDR301W \| YJR093C \| YKL059C \| YKR002W \| YLR115W \| YLR277C \| YMR061W \| YNL317W \| YDR225W \| YHL001W \| YDR228C \| YMR182C |
| complex_332 | YBR119W \| YDR235W \| YDR240C \| YGR013W \| YHR086W \| YIL061C \| YKL012W \| YLL036C \| YLR298C \| YML046W \| YMR125W \| YDL087C \| YKL074C \| YBR172C \| YLR116W |
| complex_333 | YDL097C \| YDL147W \| YDR363W-A \| YFR004W \| YFR052W \| YGL048C \| YHR027C \| YHR200W \| YIL075C \| YKL145W \| YLR421C \| YOR261C \| YDR427W \| YGR184C \| YPL177C |
| complex_334 | YAL034W-A \| YBR211C \| YDR254W \| YIR010W \| YPL018W \| YPR046W \| YAR035W \| YLR381W \| YBR107C \| YDR383C \| YGR179C \| YJR135C \| YKL049C \| YMR168C \| YPL233W |
| complex_335 | YBR055C \| YBR152W \| YER172C \| YGR091W \| YHR165C \| YLL036C \| YLR438C-A \| YNL147W \| YOR308C \| YPR178W \| YDL098C \| YDR473C \| YKL173W \| YGR075C \| YJR022W |
| complex_340 | YBR154C \| YDL140C \| YGR005C \| YPL129W \| YDR404C \| YGL070C \| YJL140W \| YPR056W \| YGR104C \| YGR186W \| YIL021W \| YOR151C \| YLR071C \| YLR005W \| YPR187W |

| | |
|---|---|
| complex_342 | YER126C \| YER006W \| YGR103W \| YKR081C \| YLR074C \| YNL110C \| YLR106C \| YGR245C \| YHR197W \| YNL002C \| YPL093W \| YHR052W \| YLL008W \| YNL175C \| YPL043W |
| complex_343 | YAL034W-A \| YBR211C \| YDR254W \| YIR010W \| YPL018W \| YPR046W \| YBR107C \| YDR383C \| YGR179C \| YJR135C \| YLR381W \| YDR318W \| YLR315W \| YHR102W \| YPL233W |
| complex_347 | YBR188C \| YDL209C \| YDR416W \| YGR129W \| YJR050W \| YLL036C \| YLR117C \| YLR297W \| YMR001C \| YMR213W \| YPR101W \| YKL095W \| YPL151C \| YPL213W \| YIL125W |
| complex_348 | YBR065C \| YDL209C \| YLL036C \| YLR424W \| YMR001C \| YMR213W \| YPR101W \| YBR188C \| YDR416W \| YGR129W \| YJR050W \| YLR117C \| YLR297W \| YGR003W \| YPL151C |
| complex_349 | YDL040C \| YDL097C \| YDL147W \| YGL048C \| YOR117W \| YOR253W \| YOR259C \| YOR261C \| YDR363W-A \| YDR394W \| YFR004W \| YFR052W \| YHR027C \| YKL145W \| YKL010C |
| complex_35 | YBR198C \| YCR042C \| YDR145W \| YDR167W \| YER148W \| YGL112C \| YGR274C \| YML114C \| YMR005W \| YMR227C \| YMR236W \| YPL011C \| YPL129W \| YML015C \| YML098W |
| complex_352 | YBR188C \| YDL209C \| YDR416W \| YGR129W \| YCR063W \| YHR165C \| YLL036C \| YMR001C \| YMR213W \| YPR101W \| YJR050W \| YLR117C \| YLR426W \| YPL151C \| YGL251C |
| complex_353 | YBR189W \| YCR057C \| YHR196W \| YJR002W \| YMR138W \| YOR310C \| YDR449C \| YLR129W \| YMR093W \| YDR324C \| YJL109C \| YER082C \| YPL126W \| YGR128C \| YLR409C |
| complex_354 | YBL093C \| YCR081W \| YGL025C \| YOL135C \| YBR193C \| YBR253W \| YDL005C \| YGR104C \| YLR071C \| YNL236W \| YNR010W \| YPR070W \| YGL024W \| YOL130W \| YGL151W |
| complex_357 | YBR065C \| YDL209C \| YLL036C \| YLR424W \| YMR001C \| YMR213W \| YPR101W \| YBR188C \| YDR416W \| YGR129W \| YDR247W \| YJR050W \| YLR117C \| YPL077C \| YPL151C |
| complex_358 | YBR188C \| YDL209C \| YDR416W \| YGR129W \| YCR063W \| YLL036C \| YMR001C \| YMR213W \| YPR101W \| YDR163W \| YDR482C \| YJR050W \| YLR117C \| YPL064C \| YPL151C |
| complex_359 | YCR072C \| YDR101C \| YER006W \| YER126C \| YLR009W \| YLR074C \| YPL093W \| YKR081C \| YER083C \| YNL110C \| YGR245C \| YHR197W \| YNL002C \| YHR052W \| YHR204W |
| complex_360 | YBL093C \| YCR081W \| YGL025C \| YOL135C \| YBR169C \| YNL127W \| YBR193C \| YBR253W \| YDL005C \| YGR104C \| YLR071C \| YNL236W \| YNR010W \| YPR070W \| YGL151W |
| complex_362 | YAL034W-A \| YBR211C \| YDR254W \| YIR010W \| YPL018W \| YPR046W \| YBR107C \| YDR383C \| YGR179C \| YJR135C \| YLR381W \| YEL077C \| YIL111W \| YKL049C \| YPL233W |
| complex_363 | YAL034W-A \| YBR211C \| YDR254W \| YIR010W \| YPL018W \| YPR046W \| YBR107C \| YDR383C \| YGR179C \| YJR135C \| YLR381W \| YKL049C \| YOR026W \| Q0092 \| YPL233W |
| complex_364 | YBR119W \| YDR235W \| YDR240C \| YGR013W \| YHR086W \| YIL061C \| YKL012W \| YLL036C \| YLR298C \| YML046W \| YMR125W \| YDL087C \| YGR214W \| YIL009W \| YNL210W |

| | |
|---|---|
| complex_366 | YER126C \| YER006W \| YGR103W \| YKR081C \| YLR009W \| YMR049C \| YMR290C \| YNL061W \| YNL110C \| YHR052W \| YKL009W \| YKL021C \| YPL093W \| YGL141W \| YPR016C |
| complex_367 | YCR072C \| YER006W \| YER126C \| YLR009W \| YLR074C \| YLR106C \| YNL182C \| YPL093W \| YNL110C \| YFL002C \| YGR245C \| YHR197W \| YHR052W \| YHR085W \| YLR356W |
| complex_368 | YAL021C \| YDR443C \| YGR092W \| YIL038C \| YNL288W \| YNR052C \| YPL042C \| YPR072W \| YCR093W \| YGR134W \| YNL025C \| YDL165W \| YER068W \| YKR036C \| YML098W |
| complex_37 | YAL043C \| YGR156W \| YCL037C \| YJL033W \| YDR195W \| YDR301W \| YJR093C \| YKL018W \| YKL059C \| YKR002W \| YLR115W \| YLR277C \| YMR061W \| YNL317W \| YPR107C |
| complex_370 | YAL043C \| YGL044C \| YGR156W \| YDR228C \| YDR301W \| YGR047C \| YKL059C \| YKR002W \| YLR277C \| YMR061W \| YNL317W \| YLR115W \| YOR171C \| YOR185C \| YOR250C |
| complex_371 | YAL043C \| YGL044C \| YGR156W \| YBR105C \| YMR061W \| YDR228C \| YDR301W \| YGR047C \| YKL059C \| YKR002W \| YLR277C \| YNL317W \| YLR082C \| YLR115W \| YOR250C |
| complex_372 | YDL097C \| YDL147W \| YDR394W \| YER012W \| YER021W \| YFR004W \| YHR027C \| YHR200W \| YIL075C \| YKL145W \| YOR261C \| YDR427W \| YFR010W \| YGL141W \| YKL021C |
| complex_374 | YBR055C \| YBR152W \| YER172C \| YGR091W \| YHR165C \| YLL036C \| YLR438C-A \| YNL147W \| YOR308C \| YPR178W \| YDR378C \| YDR473C \| YKL173W \| YLR305C \| YJR022W |
| complex_375 | YBR188C \| YDL209C \| YDR416W \| YGR129W \| YCR063W \| YDR408C \| YHR165C \| YLL036C \| YMR001C \| YMR213W \| YPR101W \| YOR174W \| YJR050W \| YLR117C \| YPL151C |
| complex_38 | YBL084C \| YDL008W \| YDR118W \| YDR260C \| YFR036W \| YGL240W \| YHR166C \| YIR025W \| YKL022C \| YLR102C \| YLR127C \| YNL172W \| YGR052W \| YOR249C \| YPL060W |
| complex_382 | YBR188C \| YDL209C \| YDR416W \| YGR129W \| YCR063W \| YLL036C \| YMR001C \| YMR213W \| YPR101W \| YDR163W \| YJR050W \| YLR117C \| YLR323C \| YDR315C \| YPL151C |
| complex_384 | YCL054W \| YER006W \| YGR103W \| YKR081C \| YDL031W \| YER126C \| YLR009W \| YLR074C \| YNL110C \| YGR245C \| YHR197W \| YNL002C \| YHR052W \| YNL154C \| YPR016C |
| complex_385 | YBR081C \| YDR167W \| YDR176W \| YER164W \| YMR223W \| YMR236W \| YBR198C \| YDR145W \| YGR252W \| YPL047W \| YCL010C \| YDR448W \| YGL252C \| YNL076W \| YOR304W |
| complex_39 | YBL084C \| YDL008W \| YDR118W \| YDR260C \| YFR036W \| YGL240W \| YHR166C \| YIR025W \| YKL022C \| YLR102C \| YLR127C \| YNL172W \| YBR082C \| YKL034W \| YOR249C |
| complex_391 | YBR048W \| YBR247C \| YCL059C \| YDL148C \| YHR196W \| YGR090W \| YHR203C \| YCR057C \| YDR064W \| YJL109C \| YJR145C \| YPR144C \| YLR441C \| YPL217C \| YPR137W |
| complex_394 | YAL034C \| YGR274C \| YBR198C \| YCR042C \| YDR145W \| YDR167W \| YML114C \| YMR005W \| YMR227C \| YMR236W \| YPL011C \| YKL201C \| YPL128C \| YML015C \| YML098W |

| | |
|---|---|
| complex_396 | YCR072C \| YDR101C \| YER006W \| YER126C \| YLR009W \| YLR074C \| YLR106C \| YNL182C \| YPL093W \| YNL110C \| YGR245C \| YHR197W \| YLL008W \| Q0032 \| YPR016C |
| complex_397 | YCL059C \| YCR057C \| YDR449C \| YGR090W \| YJR002W \| YKR060W \| YLR129W \| YOR310C \| YDR324C \| YJL109C \| YER082C \| YLR222C \| YJL069C \| YDR179C \| YLR409C |
| complex_398 | YBL103C \| YOL067C \| YCR042C \| YDR167W \| YML114C \| YMR005W \| YMR236W \| YDR145W \| YMR227C \| YER148W \| YGR274C \| YML015C \| YML098W \| YPL011C \| YOL108C |
| complex_399 | YCR035C \| YDR280W \| YGR095C \| YGR195W \| YOL021C \| YOR001W \| YDL111C \| YGR158C \| YHR069C \| YNL232W \| YHR024C \| YHR120W \| YIL066C \| YLR163C \| YHR081W |
| complex_40 | YBL084C \| YDL008W \| YDR118W \| YDR260C \| YFR036W \| YGL240W \| YHR166C \| YIR025W \| YKL022C \| YLR102C \| YLR127C \| YNL172W \| YDL044C \| YLL051C \| YOR249C |
| complex_401 | YBL042C \| YER021W \| YBR207W \| YDL040C \| YDL097C \| YDL147W \| YGL048C \| YOR261C \| YDR363W-A \| YFR004W \| YFR052W \| YHR027C \| YHR200W \| YKL145W \| YDR427W |
| complex_402 | YAL032C \| YDL209C \| YDR364C \| YDR416W \| YGR278W \| YKL095W \| YLL036C \| YLR117C \| YLR424W \| YMR001C \| YMR213W \| YPR101W \| YDR163W \| YGR049W \| YPL151C |
| complex_403 | YBR154C \| YDL140C \| YGR005C \| YPL129W \| YDR404C \| YGL070C \| YJL140W \| YGR104C \| YGR186W \| YIL021W \| YJR017C \| YML010W \| YOR151C \| YDR457W \| YPR187W |
| complex_404 | YAL034W-A \| YBR211C \| YDR254W \| YIR010W \| YJR112W \| YPL018W \| YPR046W \| YBR107C \| YGR179C \| YLR052W \| YOR269W \| YDR318W \| YKL089W \| YKL049C \| YPL233W |
| complex_405 | YAL032C \| YDL209C \| YDR416W \| YGL128C \| YKL095W \| YKL173W \| YLR117C \| YLR424W \| YMR213W \| YPR101W \| YHR165C \| YKR022C \| YGL120C \| YJL099W \| YPL151C |
| complex_406 | YCR072C \| YDR101C \| YER006W \| YER126C \| YLR009W \| YLR074C \| YLR106C \| YNL182C \| YPL093W \| YNL110C \| YGR245C \| YHR197W \| YHR085W \| YOR252W \| YPR152C |
| complex_407 | YDL030W \| YBR152W \| YDL043C \| YFL017W-A \| YJL203W \| YLL036C \| YML049C \| YMR001C \| YMR213W \| YMR240C \| YOR159C \| YPR182W \| YMR288W \| YPR094W \| YPL213W |
| complex_409 | YBR204C \| YPL096W \| YDL097C \| YDL147W \| YDR363W-A \| YER021W \| YFR004W \| YFR052W \| YGL004C \| YHR027C \| YHR200W \| YIL075C \| YKL145W \| YLR421C \| YOR261C |
| complex_410 | YAL032C \| YDL209C \| YDR416W \| YGL128C \| YKL095W \| YKL173W \| YLR117C \| YLR424W \| YMR176W \| YMR213W \| YPR101W \| YHR165C \| YDR213W \| YGL120C \| YPL151C |
| complex_411 | YBR154C \| YDL140C \| YGR005C \| YPL129W \| YDR404C \| YGL070C \| YJL140W \| YOL005C \| YHR143W-A \| YGR104C \| YIL021W \| YJL168C \| YOR151C \| YOR224C \| YPL073C |
| complex_413 | YDL025C \| YAL049C \| YDL097C \| YDL147W \| YER021W \| YFR004W \| YFR052W \| YHR027C \| YHR200W \| YDR363W-A \| YGL004C \| YIL075C \| YLR421C \| YKL145W \| YOR261C |

| | |
|---|---|
| complex_414 | YCR072C \| YDR101C \| YER006W \| YER126C \| YLR009W \| YLR074C \| YPL093W \| YGR103W \| YPR017C \| YNL110C \| YGR245C \| YHR197W \| YHR052W \| YJL122W \| YLR397C |
| complex_415 | YCR057C \| YDR449C \| YJR002W \| YMR093W \| YOR310C \| YDR324C \| YJL109C \| YOL108C \| YER082C \| YPL126W \| YGR128C \| YJL069C \| YLR409C \| YMR229C \| YMR317W |
| complex_417 | YBL050W \| YDR189W \| YGL098W \| YKL196C \| YLR026C \| YLR268W \| YOR075W \| YLR440C \| YDR498C \| YNL258C \| YGL145W \| YHL031C \| YIL004C \| YKL006C-A \| YLR078C |
| complex_42 | YBL084C \| YDL008W \| YDR118W \| YDR260C \| YFR036W \| YGL240W \| YHR166C \| YIR025W \| YKL022C \| YLR102C \| YLR127C \| YNL172W \| YMR092C \| YNL176C \| YOR249C |
| complex_421 | YBL041W \| YDL020C \| YOL038W \| YDL188C \| YER012W \| YER094C \| YFR050C \| YGL011C \| YGR135W \| YML092C \| YMR314W \| YOR362C \| YPR103W \| YHR200W \| YBL025W |
| complex_422 | YBR181C \| YJR002W \| YCL059C \| YCR057C \| YDR449C \| YLR129W \| YMR093W \| YDR324C \| YJL109C \| YER082C \| YLR222C \| YJL069C \| YLR409C \| YPL081W \| YPR144C |
| complex_425 | YDL097C \| YDL147W \| YDR363W-A \| YDR394W \| YER021W \| YFR004W \| YGL004C \| YHR027C \| YHR200W \| YKL145W \| YOR261C \| YPR108W \| YDL144C \| YDR088C \| YGR232W |
| complex_427 | YAL034W-A \| YBR073W \| YBR211C \| YDR254W \| YGL093W \| YIR010W \| YJR112W \| YML032C \| YPL018W \| YBR107C \| YGR179C \| YDR318W \| YKL089W \| YKL049C \| YPL233W |
| complex_428 | YAL055W \| YDL065C \| YGR133W \| YGL153W \| YDR142C \| YDR244W \| YHR160C \| YLR191W \| YNL214W \| YGR077C \| YMR026C \| YDR265W \| YDR329C \| YJL210W \| YOL044W |
| complex_433 | YAL055W \| YDL065C \| YGL153W \| YOL147C \| YDL078C \| YDR244W \| YDR142C \| YLR191W \| YNL214W \| YGR077C \| YMR026C \| YDR265W \| YDR329C \| YJL210W \| YOL044W |
| complex_434 | YCR072C \| YDR101C \| YER006W \| YER126C \| YLR009W \| YLR074C \| YLR106C \| YNL182C \| YPL093W \| YNL110C \| YGR245C \| YHR197W \| YHR085W \| YLR356W \| Q0032 |
| complex_438 | YDR271C \| YOR244W \| YDR359C \| YGR002C \| YHR090C \| YJR082C \| YEL018W \| YFL024C \| YJL081C \| YFL039C \| YHR099W \| YKR021W \| YNL107W \| YPR023C \| YNL136W |
| complex_439 | YBR289W \| YDR073W \| YEL009C \| YHL025W \| YJL176C \| YKL109W \| YMR033W \| YNR023W \| YOR290C \| YPL016W \| YPL129W \| YFR037C \| YGR275W \| YIL126W \| YPR034W |
| complex_44 | YDL097C \| YDL147W \| YDR363W-A \| YDR394W \| YER012W \| YER021W \| YFR004W \| YFR052W \| YHR027C \| YHR200W \| YIL075C \| YKL145W \| YOR261C \| YDR427W \| YFR010W |
| complex_443 | YAL034W-A \| YBR211C \| YDR254W \| YIR010W \| YPL018W \| YGR179C \| YDR318W \| YJR060W \| YKL089W \| YGR113W \| YGR140W \| YMR094W \| YKL049C \| YPL233W \| YMR168C |
| complex_452 | YAL043C \| YGR156W \| YAR003W \| YBR175W \| YBR258C \| YKL018W \| YLR015W \| YDR195W \| YKL059C \| YNL222W \| YNL317W \| YPR107C \| YDR469W \| YHR119W \| YPL138C |

| | |
|---|---|
| complex_457 | YBR279W \| YDL140C \| YGL244W \| YLR418C \| YOL145C \| YOR123C \| YER164W \| YGL019W \| YGL207W \| YGR063C \| YOR061W \| YGR116W \| YIL035C \| YML010W \| YML069W |
| complex_459 | YBR119W \| YDR235W \| YDR240C \| YFL017W-A \| YGR013W \| YHR086W \| YIL061C \| YKL012W \| YLL036C \| YLR298C \| YMR125W \| YDL087C \| YLR226W \| YPR161C \| YPR057W |
| complex_46 | YBR081C \| YBR111W-A \| YDR167W \| YMR223W \| YMR236W \| YBR198C \| YDR145W \| YDR392W \| YGR252W \| YLR055C \| YOL148C \| YPL047W \| YPL254W \| YDR448W \| YHR079C |
| complex_461 | YCL059C \| YCR057C \| YDR449C \| YJR002W \| YLR129W \| YMR093W \| YDL166C \| YLR222C \| YDR324C \| YJL109C \| YER082C \| YPL126W \| YJL069C \| YLR208W \| YLR409C |
| complex_462 | YCL059C \| YCR057C \| YDR449C \| YHR148W \| YJR002W \| YMR093W \| YCR099C \| YDR324C \| YER122C \| YGR128C \| YJL109C \| YPL126W \| YMR229C \| YOR078W \| YPR137W |
| complex_464 | YBR065C \| YDL209C \| YLL036C \| YLR424W \| YMR001C \| YMR213W \| YPR101W \| YDR088C \| YER013W \| YER172C \| YGR074W \| YKR086W \| YLR117C \| YKL173W \| YPL151C |
| complex_466 | YDL097C \| YDL147W \| YDR363W-A \| YER021W \| YFR004W \| YFR052W \| YHL030W \| YHR200W \| YKL145W \| YOR261C \| YMR314W \| YDR256C \| YDR427W \| YFR010W \| YDL084W |
| complex_467 | YCR052W \| YER148W \| YFR037C \| YGR056W \| YLR033W \| YLR321C \| YLR357W \| YDR303C \| YIL126W \| YKR008W \| YML127W \| YPR034W \| YEL009C \| YOR298C-A \| YDR099W |
| complex_468 | YBR119W \| YDR240C \| YFL017W-A \| YGR013W \| YHR086W \| YIL061C \| YKL012W \| YLL036C \| YLR298C \| YML046W \| YMR125W \| YDL087C \| YGR214W \| YIL009W \| YOL130W |
| complex_471 | YCL009C \| YER012W \| YER094C \| YMR108W \| YDL188C \| YFR050C \| YGL011C \| YGR135W \| YML092C \| YMR314W \| YOL038W \| YOR157C \| YPR103W \| YJL001W \| YHR200W |
| complex_472 | YBL093C \| YCR081W \| YGL025C \| YOL135C \| YBR253W \| YDL005C \| YDR443C \| YGR104C \| YNL236W \| YNR010W \| YPR070W \| YGL151W \| YNL025C \| YOR140W \| YPL203W |
| complex_473 | YBR142W \| YDR060W \| YKL014C \| YMR049C \| YMR290C \| YNL061W \| YPL043W \| YDL213C \| YGR103W \| YKR081C \| YHR052W \| YLL008W \| YHR066W \| YJR041C \| YOR272W |
| complex_474 | YCL059C \| YCR057C \| YDR449C \| YGR090W \| YJR002W \| YLR129W \| YMR093W \| YDR324C \| YJL109C \| YER064C \| YPL212C \| YER082C \| YLR222C \| YJL069C \| YLR409C |
| complex_475 | YBL087C \| YGR103W \| YDR060W \| YKR081C \| YMR049C \| YNL061W \| YER126C \| YER006W \| YLR009W \| YMR290C \| YNL110C \| YOR206W \| YLR002C \| YPL093W \| YPR016C |
| complex_479 | YBR267W \| YHR170W \| YCR072C \| YDR101C \| YER006W \| YER126C \| YLR009W \| YLR074C \| YPL093W \| YNL110C \| YGL099W \| YGR245C \| YHR197W \| YHR052W \| YJL122W |
| complex_485 | YDL196W \| YDL094C \| YER071C \| YDR195W \| YGR156W \| YJR093C \| YKL018W \| YKL059C \| YKR002W \| YLR115W \| YLR277C \| YNL222W \| YNL317W \| YPR107C \| YHL035C |

| | |
|---|---|
| complex_486 | YBR055C \| YBR152W \| YER172C \| YFL017W-A \| YGR091W \| YLL036C \| YLR438C-A \| YOR159C \| YPR178W \| YPR182W \| YDL098C \| YMR288W \| YDR473C \| YKL173W \| YGR075C |
| complex_487 | YCR052W \| YFR037C \| YGR056W \| YLR321C \| YLR357W \| YMR033W \| YDL044C \| YDR303C \| YGR275W \| YIL126W \| YKR008W \| YPR034W \| YHR056C \| YFL067W \| YMR224C |
| complex_491 | YBL074C \| YDL208W \| YHR165C \| YBR055C \| YBR152W \| YDR283C \| YER172C \| YGR091W \| YLL036C \| YLR438C-A \| YOR308C \| YPR178W \| YKL173W \| YDR473C \| YJR022W |
| complex_496 | YDL007W \| YDR363W-A \| YFR004W \| YFR052W \| YGL048C \| YHR027C \| YDL097C \| YDL147W \| YDR394W \| YHR200W \| YKL145W \| YPR131C \| YDR427W \| YKR024C \| YOL076W |
| complex_50 | YCR020W-B \| YFR037C \| YIL126W \| YLR033W \| YMR091C \| YCR052W \| YLR321C \| YLR357W \| YMR033W \| YDR303C \| YGR275W \| YKR008W \| YML127W \| YPR034W \| YGL184C |
| complex_500 | YBR247C \| YDR449C \| YJR002W \| YCL059C \| YCR057C \| YMR093W \| YDR324C \| YER102W \| YDR459C \| YOR078W \| YPR144C \| YGL171W \| YGR128C \| YJL109C \| YPL126W |
| complex_501 | YAR002C-A \| YGL200C \| YIL004C \| YLR026C \| YML012W \| YDL195W \| YDL212W \| YIL109C \| YLR208W \| YLR268W \| YPL085W \| YPR181C \| YHL031C \| YLR078C \| YPL218W |
| complex_505 | YCL059C \| YCR057C \| YDR449C \| YJR002W \| YLR129W \| YMR093W \| YDR283C \| YNL213C \| YDR324C \| YJL109C \| YER082C \| YLR222C \| YPL126W \| YJL069C \| YLR409C |
| complex_506 | YBL093C \| YDR308C \| YGL025C \| YOL135C \| YBR193C \| YGR104C \| YHR041C \| YHR058C \| YLR071C \| YNR010W \| YOL051W \| YOR174W \| YER066W \| YHR147C \| YMR154C |
| complex_507 | YBR154C \| YJL148W \| YJR063W \| YNL113W \| YOR116C \| YPR010C \| YPR110C \| YOR210W \| YKL144C \| YOR224C \| YML010W \| YNL248C \| YOR340C \| YOR341W \| YPR187W |
| complex_508 | YBL008W \| YOR038C \| YOR290C \| YBR289W \| YDR073W \| YEL009C \| YHL025W \| YJL176C \| YKL109W \| YMR033W \| YNR023W \| YPL016W \| YPL129W \| YGR275W \| YPR034W |
| complex_511 | YBL026W \| YCR077C \| YIL048W \| YLL036C \| YMR268C \| YNL147W \| YDR378C \| YER146W \| YOL149W \| YER112W \| YGL173C \| YJL124C \| YLR438C-A \| YJR022W \| YNL297C |
| complex_513 | YBL026W \| YCR077C \| YLL036C \| YMR268C \| YNL147W \| YCR024C \| YER146W \| YJL124C \| YDR378C \| YOL149W \| YER112W \| YFL066C \| YGL173C \| YLR438C-A \| YJR022W |
| complex_514 | YBR173C \| YGL011C \| YPR103W \| YDL188C \| YER012W \| YER094C \| YFR050C \| YGR135W \| YMR314W \| YOL038W \| YOR362C \| YGL048C \| YHR200W \| YGR184C \| YML092C |
| complex_515 | YDL065C \| YGL153W \| YDR142C \| YDR244W \| YHR160C \| YLR191W \| YNL214W \| YGR077C \| YMR026C \| YDR265W \| YDR329C \| YOR180C \| YJL210W \| YLR284C \| YOL044W |
| complex_517 | YDL065C \| YGL153W \| YDR142C \| YDR244W \| YGR239C \| YHR160C \| YIL160C \| YLR191W \| YNL214W \| YGR077C \| YMR026C \| YDR265W \| YDR329C \| YJL210W \| YOL044W |

| | |
|---|---|
| complex_519 | YAL025C \| YGR103W \| YJR044C \| YLR009W \| YOR005C \| YGL111W \| YHR088W \| YKR081C \| YMR049C \| YNL061W \| YNL110C \| YPL093W \| YHR066W \| YPR016C \| YOR272W |
| complex_521 | YDL188C \| YER012W \| YER094C \| YFR050C \| YGL011C \| YGR135W \| YML092C \| YMR314W \| YOL038W \| YOR362C \| YPR103W \| YJL001W \| YHR200W \| YOR043W \| YLL010C |
| complex_524 | YDL188C \| YER012W \| YER094C \| YFR050C \| YGL011C \| YGR135W \| YMR314W \| YOL038W \| YOR362C \| YPR103W \| YDR256C \| YHR200W \| YJL001W \| YML092C \| YLR076C |
| complex_527 | YBR154C \| YNL113W \| YOR116C \| YPR110C \| YDL150W \| YKR025W \| YDR005C \| YDR045C \| YOR207C \| YJL011C \| YKL144C \| YNL151C \| YNR003C \| YPR190C \| YOR224C |
| complex_542 | YDL188C \| YER012W \| YER094C \| YFR050C \| YGL011C \| YGR135W \| YML092C \| YMR314W \| YOL038W \| YOR157C \| YPR103W \| YJL001W \| YHR200W \| YDR428C \| YER144C |
| complex_544 | YBL093C \| YCR081W \| YGL025C \| YOL135C \| YBL101C \| YJL203W \| YBR193C \| YBR253W \| YDL005C \| YGR104C \| YNL236W \| YNR010W \| YPR070W \| YGL151W \| YNL025C |
| complex_545 | YCR057C \| YDR449C \| YER082C \| YGR128C \| YJR002W \| YMR093W \| YMR229C \| YPL126W \| YPR144C \| YDR324C \| YHR196W \| YEL055C \| YER100W \| YGL043W \| YJL109C |
| complex_546 | YBL093C \| YCR081W \| YGL025C \| YOL135C \| YPL129W \| YBR253W \| YDL005C \| YGR104C \| YNL236W \| YNR010W \| YPR070W \| YGL151W \| YNL025C \| YHR143W-A \| YIL025C |
| complex_547 | YBL093C \| YGL025C \| YOL135C \| YPL129W \| YBR193C \| YBR253W \| YDL005C \| YGR104C \| YHR041C \| YHR058C \| YLR071C \| YOL051W \| YER066W \| YHR147C \| YMR154C |
| complex_548 | YGL111W \| YGR103W \| YKR081C \| YLR009W \| YMR049C \| YNL061W \| YNL110C \| YPL093W \| YHR052W \| YKL172W \| YNL230C \| YOL077C \| YHR066W \| YOR272W \| YOR294W |
| complex_552 | YDL188C \| YER012W \| YER094C \| YFR050C \| YGL011C \| YGR135W \| YMR314W \| YOL038W \| YOR157C \| YPR103W \| YHR200W \| YJL001W \| YER145C \| YML092C \| YPR180W |
| complex_553 | YBR231C \| YDR190C \| YDR334W \| YFL039C \| YGR002C \| YJL081C \| YML041C \| YOL012C \| YPL235W \| YCR060W \| YHR034C \| YGL150C \| YNL215W \| YPL129W \| YNL107W |
| complex_555 | YBR231C \| YDR190C \| YDR334W \| YFL039C \| YGR002C \| YJL081C \| YLR385C \| YML041C \| YOL012C \| YPL235W \| YDR485C \| YHL003C \| YKL008C \| YMR298W \| YNL107W |
| complex_556 | YBR231C \| YDR190C \| YDR334W \| YFL039C \| YGR002C \| YJL081C \| YLR385C \| YML041C \| YOL012C \| YPL235W \| YCR060W \| YHR034C \| YNL215W \| YNL107W \| YBR287W |
| complex_557 | YBL041W \| YOL038W \| YPR103W \| YDL188C \| YER012W \| YER094C \| YFR050C \| YGL011C \| YGR135W \| YMR314W \| YOR362C \| YHR200W \| YKL010C \| YKR069W \| YML092C |
| complex_559 | YBR019C \| YGL252C \| YLR055C \| YBR081C \| YBR111W-A \| YDR176W \| YMR223W \| YDR145W \| YDR392W \| YOL148C \| YPL047W \| YPL254W \| YDR448W \| YCR005C \| YPL245W |

| | |
|---|---|
| complex_56 | YBR081C \| YDR167W \| YDR176W \| YMR223W \| YMR236W \| YBR198C \| YDR145W \| YDR392W \| YER164W \| YGR252W \| YPL047W \| YPL254W \| YCL010C \| YDR448W \| YGL252C |
| complex_561 | YBL066C \| YGR117C \| YBR127C \| YDL185W \| YDR328C \| YOR270C \| YDR202C \| YEL051W \| YGR020C \| YHR039C-A \| YOR332W \| YPR036W \| YJR033C \| YKL080W \| YMR054W |
| complex_563 | YDL097C \| YDR363W-A \| YDR394W \| YFR052W \| YGL048C \| YHR027C \| YHR200W \| YKL145W \| YOR117W \| YOR259C \| YDR427W \| YMR276W \| YNR059W \| YEL037C \| YIL148W |
| complex_564 | YBR142W \| YDR060W \| YLR009W \| YMR049C \| YMR290C \| YNL061W \| YOL041C \| YPL043W \| YPL093W \| YOL077C \| YHR052W \| YPL259C \| YHR066W \| YLR196W \| YOR272W |
| complex_567 | YBR019C \| YGL252C \| YLR055C \| YBR081C \| YBR111W-A \| YDR176W \| YGL043W \| YMR223W \| YMR236W \| YCL010C \| YDR145W \| YGR252W \| YPL047W \| YDR448W \| YCR005C |
| complex_569 | YBR160W \| YER012W \| YFR030W \| YGL048C \| YHR027C \| YBR217W \| YDL097C \| YDL147W \| YDR394W \| YER021W \| YFR004W \| YHR200W \| YKL145W \| YPR108W \| YGR262C |
| complex_57 | YDL097C \| YDL147W \| YDR363W-A \| YDR394W \| YER021W \| YFR004W \| YFR052W \| YGL004C \| YGL048C \| YHR027C \| YHR200W \| YKL145W \| YLR421C \| YOR261C \| YPR108W |
| complex_570 | YBL093C \| YDR308C \| YGL025C \| YOL135C \| YBR193C \| YER022W \| YGR104C \| YLR071C \| YMR112C \| YNR010W \| YOR174W \| YPR070W \| YKL051W \| YLR305C \| YNL055C |
| complex_571 | YBL093C \| YDR308C \| YGL025C \| YOL135C \| YBR193C \| YGL127C \| YGR104C \| YHR058C \| YLR071C \| YNR010W \| YOR174W \| YPR070W \| YDR408C \| YNR039C \| YDR205W |
| complex_575 | YAL005C \| YLR256W \| YNL064C \| YAL043C \| YGR156W \| YCL037C \| YJL033W \| YDR195W \| YJR093C \| YKL059C \| YKR002W \| YLR115W \| YLR277C \| YMR061W \| YNL317W |
| complex_576 | YBR154C \| YDL140C \| YGR005C \| YPL129W \| YDR404C \| YGL070C \| YJL070C \| YJL140W \| YOL005C \| YGR104C \| YIL021W \| YJL168C \| YNL251C \| YOR151C \| YOR224C |
| complex_577 | YBR065C \| YDL209C \| YLL036C \| YMR001C \| YMR213W \| YPR101W \| YBR190W \| YLR117C \| YDR088C \| YER013W \| YKR086W \| YDR416W \| YGR129W \| YJR050W \| YPL151C |
| complex_58 | YAL043C \| YGL044C \| YGR156W \| YDR195W \| YDR301W \| YJR093C \| YKL059C \| YKR002W \| YLR115W \| YLR277C \| YMR061W \| YNL317W \| YDR228C \| YGR047C \| YOR250C |
| complex_581 | YBR127C \| YDL185W \| YOR270C \| YEL027W \| YEL051W \| YGR020C \| YHR039C-A \| YLR447C \| YOR332W \| YPR036W \| YGR105W \| YHR026W \| YKL080W \| YMR054W \| YPL234C |
| complex_583 | YBR109C \| YFR004W \| YDL007W \| YGL048C \| YHR027C \| YDL097C \| YDL147W \| YDR394W \| YHR200W \| YKL145W \| YOR261C \| YDR427W \| YFR010W \| YGL141W \| YKL021C |
| complex_586 | YAL021C \| YDR443C \| YFL028C \| YGR092W \| YIL038C \| YNL288W \| YNR052C \| YPL042C \| YPR072W \| YCR093W \| YGR134W \| YNL025C \| YDL165W \| YER068W \| YOR140W |

| | |
|---|---|
| complex_588 | YBR264C \| YER136W \| YGL198W \| YNL044W \| YNL263C \| YOR370C \| YER031C \| YFL038C \| YGL210W \| YKR014C \| YNL093W \| YNL304W \| YOR089C \| YFL005W \| YGR172C |
| complex_589 | YBL026W \| YCR077C \| YLL036C \| YMR268C \| YNL147W \| YDR378C \| YER146W \| YOL149W \| YER112W \| YIL029C \| YKL209C \| YGL173C \| YJL124C \| YLR438C-A \| YJR022W |
| complex_59 | YDL097C \| YDL147W \| YDR363W-A \| YER012W \| YER021W \| YFR052W \| YGL048C \| YHR027C \| YHR200W \| YIL075C \| YKL145W \| YLR421C \| YOR261C \| YPR108W \| YFR010W |
| complex_590 | YBR079C \| YDR429C \| YLR192C \| YMR146C \| YMR309C \| YOL087C \| YPR041W \| YDR091C \| YER025W \| YJR007W \| YNL062C \| YNL244C \| YOR361C \| YPL237W \| YPR086W |
| complex_594 | YBR087W \| YBL035C \| YFR027W \| YOR144C \| YBR088C \| YOR217W \| YCL016C \| YHR191C \| YMR078C \| YER173W \| YDR394W \| YJR068W \| YNL290W \| YOL094C \| YJL115W |
| complex_597 | YCR072C \| YDR101C \| YER006W \| YER126C \| YLR009W \| YLR074C \| YPL093W \| YKR081C \| YER083C \| YNL110C \| YGR245C \| YHR197W \| YHR052W \| YMR038C \| YPR175W |
| complex_598 | YAL034W-A \| YBR211C \| YDR254W \| YIR010W \| YPL018W \| YPR046W \| YBR107C \| YDR383C \| YGR179C \| YLR381W \| YKL049C \| YLR083C \| YLR315W \| YOL104C \| YPL233W |
| complex_599 | YBR198C \| YCR042C \| YDR145W \| YER148W \| YGR274C \| YKL058W \| YML114C \| YMR005W \| YMR227C \| YPL011C \| YOR194C \| YGL016W \| YML015C \| YML098W \| YJL174W |
| complex_603 | YDR271C \| YOR244W \| YDR359C \| YGR002C \| YHR090C \| YJR082C \| YDR509W \| YEL018W \| YFL024C \| YJL081C \| YHR099W \| YNL107W \| YPR023C \| YNL136W \| YOR128C |
| complex_604 | YAL034W-A \| YBR211C \| YDR254W \| YIR010W \| YPL018W \| YPR046W \| YBR107C \| YDR383C \| YGR179C \| YLR381W \| YKL049C \| YLR315W \| YLR395C \| YFR008W \| YPL233W |
| complex_605 | YBR119W \| YDR240C \| YGR013W \| YHR086W \| YIL061C \| YKL012W \| YLR298C \| YML046W \| YMR125W \| YPL178W \| YDL087C \| YER044C-A \| YJR021C \| YMR133W \| YNL189W |
| complex_607 | YBL041W \| YOL038W \| YDL188C \| YER012W \| YER094C \| YFR050C \| YGL011C \| YML092C \| YOR157C \| YOR362C \| YPR103W \| YJL001W \| YHR200W \| YER105C \| YJL030W |
| complex_610 | YBR079C \| YDR429C \| YMR146C \| YMR309C \| YPR041W \| YDR211W \| YKR026C \| YER025W \| YGR083C \| YJR007W \| YLR291C \| YOR260W \| YIL131C \| YNL265C \| YPL237W |
| complex_614 | YCL059C \| YCR057C \| YDR299W \| YDR449C \| YER127W \| YGR090W \| YGR145W \| YJR002W \| YLR129W \| YLR409C \| YPR144C \| YJL069C \| YDR324C \| YDR365C \| YNR054C |
| complex_616 | YAL032C \| YDL209C \| YDR364C \| YDR416W \| YKL095W \| YLL036C \| YLR117C \| YLR424W \| YMR213W \| YPR101W \| YBR065C \| YDL144C \| YDR088C \| YPL151C \| YPL178W |
| complex_618 | YAL034W-A \| YBR211C \| YDR254W \| YIR010W \| YJR112W \| YPL018W \| YGR179C \| YLR381W \| YDR318W \| YKL089W \| YJR135C \| YLR202C \| YKL049C \| YPL233W \| YLR288C |

| | |
|---|---|
| complex_619 | YBR087W \| YBL035C \| YFR027W \| YOR144C \| YBR088C \| YKL113C \| YOR217W \| YDL164C \| YER173W \| YJR068W \| YNL290W \| YOL094C \| YHR191C \| YJL115W \| YMR078C |
| complex_62 | YBR119W \| YDR235W \| YDR240C \| YFL017W-A \| YGR013W \| YHR086W \| YIL061C \| YKL012W \| YLL036C \| YLR298C \| YML046W \| YMR125W \| YPL178W \| YDL087C \| YLR275W |
| complex_620 | YDL097C \| YDR363W-A \| YER012W \| YER021W \| YFR004W \| YFR052W \| YHR027C \| YHR200W \| YIL075C \| YKL145W \| YOR261C \| YER007W \| YJL120W \| YFR010W \| YKL171W |
| complex_621 | YBR127C \| YDL185W \| YOR270C \| YDR202C \| YEL051W \| YGR020C \| YHR039C-A \| YLR447C \| YOR332W \| YPR036W \| YGR048W \| YDR390C \| YJR033C \| YKL080W \| YMR054W |
| complex_622 | YCL059C \| YCR057C \| YDR449C \| YGR090W \| YJR002W \| YMR093W \| YDR324C \| YER082C \| YJL109C \| YPL126W \| YGL171W \| YGR128C \| YLR409C \| YBR281C \| YNL191W |
| complex_624 | YBR231C \| YDR190C \| YDR334W \| YFL039C \| YGR002C \| YJL081C \| YLR385C \| YLR399C \| YML041C \| YOL012C \| YPL235W \| YDL070W \| YJL115W \| YGL063W \| YNL107W |
| complex_625 | YBR154C \| YDL140C \| YGR005C \| YPL129W \| YBR236C \| YDR404C \| YGR063C \| YJL140W \| YPR133C \| YGR116W \| YML010W \| YGR186W \| YIL021W \| YOR151C \| YPR187W |
| complex_630 | YCR057C \| YDR449C \| YJR002W \| YLR129W \| YMR093W \| YOR310C \| YDR324C \| YJL109C \| YER082C \| YLR222C \| YJL069C \| YKR096W \| YKL099C \| YLR409C \| YML093W |
| complex_632 | YAL034W-A \| YDR254W \| YER018C \| YGL093W \| YIL144W \| YIR010W \| YJR112W \| YOL069W \| YDR318W \| YKL089W \| YMR117C \| YDR356W \| YKL049C \| YPL233W \| YKR054C |
| complex_634 | YBR154C \| YDL140C \| YGR005C \| YPL129W \| YDR404C \| YGL070C \| YJL140W \| YOL005C \| YGR104C \| YIL021W \| YJL168C \| YOR151C \| YOR224C \| YGR089W \| YJL061W |
| complex_635 | YGL111W \| YDR120C \| YGR103W \| YHR088W \| YKR081C \| YLR009W \| YMR049C \| YNL061W \| YNL110C \| YPL093W \| YHR066W \| YMR290C \| YPR016C \| YLR035C \| YOR272W |
| complex_637 | YDL116W \| YGL092W \| YDR192C \| YJR042W \| YKR082W \| YMR047C \| YOR160W \| YFR002W \| YGL100W \| YLR208W \| YGL172W \| YGR119C \| YKL057C \| YML103C \| YKL068W |
| complex_64 | YAL043C \| YGR156W \| YDR195W \| YDR301W \| YJR093C \| YKL018W \| YKL059C \| YKR002W \| YLR115W \| YLR277C \| YNL222W \| YNL317W \| YPR107C \| YGR187C \| YBR282W |
| complex_641 | YBR279W \| YGL244W \| YOL145C \| YOR123C \| YER164W \| YGL019W \| YOR039W \| YGL207W \| YGR063C \| YOR061W \| YGR116W \| YIL035C \| YML010W \| YML069W \| YPR052C |
| complex_643 | YER157W \| YFL038C \| YGR120C \| YKL196C \| YLR026C \| YNL041C \| YNL051W \| YGL005C \| YML071C \| YGL223C \| YHL031C \| YKL006C-A \| YLR078C \| YLR268W \| YPR105C |
| complex_647 | YDL195W \| YDL212W \| YGL200C \| YIL004C \| YIL109C \| YLR208W \| YLR268W \| YML012W \| YPL085W \| YPR181C \| YLR026C \| YMR307W \| YLR078C \| YKR039W \| YPL218W |

| | |
|---|---|
| complex_648 | YCL059C \| YCR057C \| YDL148C \| YGR090W \| YHR203C \| YJR002W \| YHR196W \| YDR091C \| YJR145C \| YDR324C \| YJL109C \| YNL034W \| YPR144C \| YPL126W \| YPR137W |
| complex_656 | YBR142W \| YDR496C \| YLR009W \| YMR049C \| YNL061W \| YPL093W \| YKR081C \| YFR001W \| YGR103W \| YHR052W \| YNL230C \| YHR066W \| YPR016C \| YKL185W \| YOR272W |
| complex_657 | YBR278W \| YMR290C \| YGL111W \| YGR103W \| YHR088W \| YKR081C \| YLR009W \| YMR049C \| YNL061W \| YNL110C \| YPL093W \| YHR066W \| YPR016C \| YAL007C \| YOR272W |
| complex_658 | YDL065C \| YDR034C \| YGL153W \| YGR242W \| YLR063W \| YDR142C \| YDR244W \| YLR191W \| YNL214W \| YGR077C \| YMR026C \| YDR265W \| YDR329C \| YJL210W \| YOL044W |
| complex_659 | YBR119W \| YDR240C \| YGR013W \| YHR086W \| YIL061C \| YKL012W \| YLR298C \| YML046W \| YMR125W \| YDL087C \| YKL074C \| YBR172C \| YLR116W \| YNL210W \| YPL105C |
| complex_662 | YAL021C \| YDR443C \| YFL028C \| YGR092W \| YIL038C \| YNL288W \| YNR052C \| YPL042C \| YPR072W \| YCR093W \| YGR134W \| YDL165W \| YER068W \| YML098W \| YNL278W |
| complex_665 | YAL032C \| YDL209C \| YDR416W \| YKL095W \| YLR117C \| YLR424W \| YMR213W \| YPR101W \| YBR188C \| YGR129W \| YHR161C \| YPL077C \| YJR050W \| YDR489W \| YPL151C |
| complex_666 | YCL059C \| YCR057C \| YDL148C \| YGR090W \| YHR203C \| YJR002W \| YHR196W \| YDL014W \| YJL109C \| YPR137W \| YPR144C \| YDR091C \| YJR145C \| YDR117C \| YPL126W |
| complex_667 | YBR189W \| YBR247C \| YCR057C \| YJR002W \| YOR310C \| YGR090W \| YHR148W \| YLR367W \| YNL075W \| YDL014W \| YJL109C \| YPR137W \| YDR324C \| YOR078W \| YPL126W |
| complex_668 | YBR017C \| YGL092W \| YDL116W \| YDR192C \| YJR042W \| YKR082W \| YMR047C \| YOR160W \| YGL100W \| YLR208W \| YGL172W \| YGR119C \| YPL125W \| YKL057C \| YKL068W |
| complex_670 | YBL072C \| YDR449C \| YHR196W \| YOR078W \| YOR310C \| YDR012W \| YDR091C \| YDR324C \| YJL109C \| YJR002W \| YMR093W \| YER082C \| YPL126W \| YER102W \| YGR128C |
| complex_672 | YBL021C \| YGL237C \| YOR358W \| YDR359C \| YGR002C \| YHR090C \| YEL018W \| YFL024C \| YJL081C \| YOR244W \| YHR099W \| YKL109W \| YNL107W \| YPR023C \| YNL136W |
| complex_673 | YDR323C \| YOR089C \| YER031C \| YFL038C \| YGL198W \| YNL044W \| YNL263C \| YER136W \| YKR014C \| YLR262C \| YNL093W \| YFL005W \| YGR172C \| YPR028W \| YML001W |
| complex_674 | YBR087W \| YBL035C \| YFR027W \| YOR144C \| YBR088C \| YDR510W \| YOR217W \| YER173W \| YJR068W \| YNL290W \| YOL094C \| YHR020W \| YHR191C \| YJL115W \| YMR078C |
| complex_677 | YBR081C \| YBR111W-A \| YMR223W \| YDR145W \| YDR392W \| YOL148C \| YPL047W \| YPL254W \| YDR448W \| YGR252W \| YHR079C \| YLR055C \| YFL031W \| YDR532C \| YIL134W |
| complex_68 | YAL043C \| YGR156W \| YDR195W \| YDR301W \| YJR093C \| YKL018W \| YKL059C \| YKR002W \| YLR115W \| YLR277C \| YNL222W \| YNL317W \| YPR107C \| YDL094C \| YHL035C |

| | |
|---|---|
| complex_680 | YBL041W \| YDL020C \| YOL038W \| YDL188C \| YER012W \| YFR050C \| YGL011C \| YGR135W \| YML092C \| YMR314W \| YOR362C \| YPR103W \| YHR200W \| YIL007C \| YLR024C |
| complex_682 | YCR077C \| YDL065C \| YGL153W \| YDL139C \| YDR142C \| YDR244W \| YLR191W \| YNL214W \| YGR077C \| YMR026C \| YDR265W \| YDR329C \| YCR036W \| YJL210W \| YOL044W |
| complex_683 | YAR002C-A \| YAL007C \| YGL200C \| YIL004C \| YLR026C \| YML012W \| YDL195W \| YIL109C \| YLR208W \| YLR268W \| YPL085W \| YPR181C \| YMR307W \| YLR078C \| YPL218W |
| complex_684 | YBR231C \| YDR190C \| YDR334W \| YFL039C \| YGR002C \| YJL081C \| YLR085C \| YLR385C \| YML041C \| YOL012C \| YPL235W \| YML012W \| YGL142C \| YLR124W \| YNL107W |
| complex_690 | YBL072C \| YBR247C \| YCL059C \| YDR449C \| YOR078W \| YPR144C \| YJR002W \| YMR093W \| YDR091C \| YDR324C \| YJL109C \| YER102W \| YPL126W \| YPL198W \| YPR137W |
| complex_7 | YBL084C \| YDL008W \| YDR118W \| YDR260C \| YFR036W \| YGL003C \| YGL116W \| YGL240W \| YHR166C \| YIR025W \| YKL022C \| YLR102C \| YLR127C \| YNL172W \| YOR249C |
| complex_700 | YBL026W \| YCR077C \| YLR264W \| YNL147W \| YDR378C \| YER146W \| YOL149W \| YEL015W \| YER112W \| YJL124C \| YJR022W \| YLR438C-A \| YNL118C \| YOR167C \| YBR094W |
| complex_701 | YBL004W \| YDL148C \| YGR090W \| YBR247C \| YCL059C \| YCR057C \| YER127W \| YJL109C \| YOL010W \| YML060W \| YMR128W \| YOR078W \| YPR144C \| YPL217C \| YPR137W |
| complex_702 | YBR142W \| YLR009W \| YMR049C \| YMR163C \| YMR290C \| YNL061W \| YPL093W \| YGL111W \| YGR103W \| YHR088W \| YKR081C \| YNL110C \| YHR066W \| YLR398C \| YOR272W |
| complex_703 | YAL043C \| YGR156W \| YLL045C \| YDR195W \| YER133W \| YJR093C \| YKL018W \| YKL059C \| YNL222W \| YNL317W \| YOR179C \| YPR107C \| YHL001W \| YKL006W \| YMR182C |
| complex_706 | YDL031W \| YGR103W \| YKR081C \| YOR080W \| YHR052W \| YHR088W \| YLL008W \| YMR290C \| YNL061W \| YNL110C \| YPL093W \| YHR066W \| YPR016C \| YKL176C \| YOR272W |
| complex_707 | YCL011C \| YDL084W \| YDR138W \| YDR381W \| YDR432W \| YHR167W \| YKL139W \| YNL112W \| YNL189W \| YNL253W \| YMR125W \| YKL214C \| YML062C \| YNL004W \| YNL139C |
| complex_709 | YBL050W \| YDR468C \| YKL196C \| YLR026C \| YLR268W \| YMR197C \| YOL018C \| YOR036W \| YOR106W \| YBL078C \| YGL095C \| YGL212W \| YHL031C \| YKL006C-A \| YLR093C |
| complex_710 | YDL007W \| YBR272C \| YDR363W-A \| YFR004W \| YFR052W \| YGL048C \| YDL097C \| YDL147W \| YDR394W \| YHR200W \| YOR261C \| YDR273W \| YDR427W \| YJR133W \| YLR295C |
| complex_711 | YAL034W-A \| YBR211C \| YDR254W \| YIR010W \| YPL018W \| YPR046W \| YBR107C \| YGR179C \| YLR381W \| YEL077C \| YFR053C \| YIL111W \| YKL049C \| YKL089W \| YPL233W |
| complex_712 | YER126C \| YER006W \| YGR103W \| YKR081C \| YLR009W \| YMR049C \| YMR290C \| YNL061W \| YHR052W \| YKL009W \| YKL021C \| YPL093W \| YIL096C \| YJR091C \| YPR016C |

| | |
|---|---|
| complex_713 | YBL026W \| YCR077C \| YNL145W \| YDL184C \| YER146W \| YDR378C \| YOL149W \| YDR382W \| YER112W \| YGL173C \| YJL124C \| YLR438C-A \| YNL118C \| YJR022W \| YPL220W |
| complex_714 | YCR072C \| YDR101C \| YER006W \| YER126C \| YLR009W \| YLR074C \| YPL093W \| YKR081C \| YPL211W \| YGL081W \| YGR245C \| YHR197W \| YPL146C \| YHR052W \| YAL026C |
| complex_715 | YAL021C \| YDR443C \| YGR092W \| YIL038C \| YNL288W \| YNR052C \| YPL042C \| YPR072W \| YCR093W \| YGR134W \| YDL165W \| YDR252W \| YJR055W \| YER068W \| YML098W |
| complex_716 | YAL021C \| YDR443C \| YGR092W \| YIL038C \| YNL288W \| YNR052C \| YPL042C \| YPR072W \| YCR093W \| YDR376W \| YGR134W \| YDL165W \| YER068W \| YIR024C \| YML098W |
| complex_717 | YAL021C \| YDR443C \| YGR092W \| YIL038C \| YNL288W \| YNR052C \| YPL042C \| YPR072W \| YCR093W \| YGR134W \| YDL165W \| YER068W \| YML098W \| YNL091W \| YNL164C |
| complex_723 | YAL021C \| YDR443C \| YGR092W \| YIL038C \| YNL288W \| YNR052C \| YPL042C \| YPR072W \| YCR093W \| YGR134W \| YDL165W \| YER068W \| YLR125W \| YML098W \| YNL315C |
| complex_725 | YBR247C \| YDR449C \| YGR090W \| YJR002W \| YLR129W \| YCL059C \| YCR057C \| YDR324C \| YHR196W \| YER102W \| YDR459C \| YPR144C \| YJL109C \| YLR186W \| YPL081W |
| complex_726 | YCL054W \| YER006W \| YGR103W \| YKR081C \| YOR080W \| YDL031W \| YDR312W \| YPL211W \| YHR052W \| YKL014C \| YLL008W \| YNL061W \| YNL110C \| YHR066W \| YPR016C |
| complex_728 | YAL032C \| YDL209C \| YGL128C \| YGR061C \| YKL095W \| YKL173W \| YLR117C \| YLR386W \| YLR424W \| YMR213W \| YPR101W \| YBR055C \| YHR165C \| YGL120C \| YPL151C |
| complex_73 | YBR081C \| YBR111W-A \| YDR167W \| YDR176W \| YMR223W \| YMR236W \| YBR198C \| YDR145W \| YGR252W \| YLR055C \| YPL047W \| YPL254W \| YCL010C \| YKL023W \| YDR448W |
| complex_731 | YDR027C \| YDR468C \| YDR484W \| YKR020W \| YDR359C \| YGR002C \| YHR090C \| YEL018W \| YFL024C \| YJL081C \| YOR244W \| YJL029C \| YNL107W \| YPR023C \| YNL136W |
| complex_734 | YAL021C \| YDR443C \| YIL038C \| YNL288W \| YNR052C \| YPL042C \| YPR072W \| YCR093W \| YDR376W \| YGR134W \| YNL025C \| YDL165W \| YER068W \| YIR024C \| YKR036C |
| complex_735 | YCR052W \| YER148W \| YGR056W \| YLR033W \| YLR357W \| YMR033W \| YDR303C \| YGR275W \| YIL126W \| YKR008W \| YPR034W \| YEL009C \| YBL019W \| YHR145C \| YOR298C-A |
| complex_736 | YBL093C \| YDR308C \| YOL135C \| YPL129W \| YPR168W \| YBR253W \| YDL005C \| YGR104C \| YLR071C \| YNR010W \| YPR070W \| YDR167W \| YKL157W \| YPL276W \| YDR494W |
| complex_737 | YBR119W \| YDR240C \| YFL017W-A \| YGR013W \| YHR086W \| YIL061C \| YKL012W \| YLR298C \| YML046W \| YMR125W \| YDL087C \| YLR226W \| YJL158C \| YPR161C \| YPR057W |
| complex_738 | YBR087W \| YBL035C \| YFR027W \| YOR144C \| YBR088C \| YOR217W \| YCL042W \| YNL290W \| YER173W \| YJR068W \| YOL094C \| YHR191C \| YJL115W \| YMR078C \| YBL091C |

| | |
|---|---|
| complex_739 | YCR057C \| YDR449C \| YGR090W \| YJR002W \| YLR129W \| YOR310C \| YDR324C \| YJL109C \| YER082C \| YLR222C \| YJL069C \| YKR060W \| YLR409C \| YOR312C \| YDR101C |
| complex_740 | YBR087W \| YBL035C \| YFR027W \| YLR390W \| YOR144C \| YBR088C \| YOR217W \| YER173W \| YJR068W \| YNL290W \| YOL094C \| YHR191C \| YJL115W \| YMR078C \| YMR181C |
| complex_741 | YBR234C \| YDL029W \| YIR006C \| YJR065C \| YKL013C \| YKL129C \| YLR370C \| YNR035C \| YOR181W \| YIL062C \| YJL020C \| YLR337C \| YBR109C \| YMR032W \| YMR109W |
| complex_742 | YAL032C \| YDL209C \| YDR416W \| YGL128C \| YGR061C \| YKL095W \| YKL173W \| YLR117C \| YLR386W \| YLR424W \| YMR213W \| YPR101W \| YGL120C \| YIL054W \| YPL151C |
| complex_743 | YDL065C \| YGL153W \| YLR191W \| YDR244W \| YGR077C \| YMR026C \| YDR265W \| YDR329C \| YOR180C \| YGR263C \| YNL066W \| YJL210W \| YLR284C \| YNL214W \| YOL044W |
| complex_744 | YBR079C \| YDR429C \| YLR192C \| YMR146C \| YMR309C \| YOL087C \| YPR041W \| YER025W \| YAL035W \| YJL125C \| YNL062C \| YNL244C \| YOR361C \| YPL237W \| YPR086W |
| complex_745 | YBR087W \| YBL035C \| YFR027W \| YOR144C \| YBR088C \| YOR217W \| YDR121W \| YER173W \| YJR068W \| YNL290W \| YOL094C \| YHR156C \| YHR191C \| YJL115W \| YMR078C |
| complex_748 | YBL023C \| YBR202W \| YCL061C \| YGL201C \| YIL150C \| YJL194W \| YLR103C \| YLR274W \| YNL261W \| YBR060C \| YDL017W \| YMR048W \| YEL032W \| YNL273W \| YPR019W |
| complex_75 | YDL097C \| YDL147W \| YDR363W-A \| YER021W \| YFR004W \| YFR052W \| YGL004C \| YHR027C \| YHR200W \| YIL075C \| YKL145W \| YLR421C \| YOR261C \| YDR427W \| YFR010W |
| complex_751 | YBL050W \| YDR189W \| YKL196C \| YLR026C \| YLR268W \| YMR197C \| YOR106W \| YBR203W \| YKL006C-A \| YOR327C \| YHL031C \| YIL004C \| YLR078C \| YLR093C \| YMR316W |
| complex_752 | YBL026W \| YER172C \| YHR165C \| YLL036C \| YNL147W \| YBR055C \| YER146W \| YLR438C-A \| YOR308C \| YPR178W \| YDR378C \| YKL173W \| YDL098C \| YFL066C \| YJR022W |
| complex_756 | YBR044C \| YKL141W \| YBR154C \| YDL140C \| YGR005C \| YPL129W \| YDR404C \| YGL070C \| YJL140W \| YOL005C \| YER022W \| YGR104C \| YIL021W \| YOR151C \| YPR187W |
| complex_759 | YBR154C \| YOR116C \| YPR110C \| YDL150W \| YKR025W \| YDR005C \| YDR045C \| YOR207C \| YGR246C \| YNL039W \| YJL011C \| YKL144C \| YNL151C \| YNR003C \| YPR190C |
| complex_760 | YDL065C \| YGL153W \| YLR191W \| YOL147C \| YDL078C \| YDR244W \| YDR142C \| YNL214W \| YGR077C \| YMR026C \| YDR265W \| YDR329C \| YJL210W \| YLR284C \| YPL240C |
| complex_764 | YAL030W \| YER143W \| YGR009C \| YOR036W \| YBL050W \| YDR468C \| YMR197C \| YOL018C \| YPL232W \| YDR164C \| YOR327C \| YGL095C \| YMR017W \| YMR183C \| YNR049C |
| complex_765 | YBL014C \| YMR236W \| YCR042C \| YML114C \| YMR005W \| YDR145W \| YMR227C \| YER148W \| YPL101W \| YGR274C \| YML015C \| YML098W \| YPL011C \| YNL099C \| YNL167C |

| | |
|---|---|
| complex_767 | YBR154C \| YDL140C \| YGR005C \| YPL129W \| YDR404C \| YGL070C \| YJL140W \| YER007C-A \| YKL028W \| YGR104C \| YGR186W \| YIL021W \| YOR151C \| YDL115C \| YPR187W |
| complex_768 | YBL050W \| YDR189W \| YKL196C \| YLR026C \| YLR268W \| YMR197C \| YOR106W \| YOR327C \| YGL147C \| YLR078C \| YHL031C \| YIL004C \| YKL006C-A \| YML055W \| YLR093C |
| complex_769 | YBR264C \| YER136W \| YGL198W \| YIL034C \| YNL044W \| YNL263C \| YOR370C \| YER031C \| YFL038C \| YKR014C \| YLR262C \| YNL093W \| YOR089C \| YFL005W \| YGR172C |
| complex_774 | YBR081C \| YBR111W-A \| YMR223W \| YCL010C \| YDR145W \| YGL112C \| YGR252W \| YPL047W \| YDR159W \| YOR046C \| YDR448W \| YOL072W \| YOR257W \| YGL066W \| YDL011C |
| complex_775 | YDL040C \| YDL097C \| YDL147W \| YGL048C \| YOR117W \| YOR259C \| YDR363W-A \| YFR004W \| YFR052W \| YHR027C \| YKL145W \| YDR427W \| YDR127W \| YJL008C \| YJL184W |
| complex_777 | YBR154C \| YDL140C \| YGR005C \| YPL129W \| YDR404C \| YGL070C \| YJL140W \| YOL005C \| YHR143W-A \| YGR104C \| YIL021W \| YJL168C \| YIL025C \| YKL025C \| YOR224C |
| complex_778 | YBL026W \| YCR077C \| YNL145W \| YNR053C \| YDR378C \| YER146W \| YOL149W \| YER112W \| YGL103W \| YGL173C \| YJL124C \| YLR438C-A \| YNL118C \| YJR022W \| YPL220W |
| complex_779 | YBR279W \| YDL140C \| YGL244W \| YLR418C \| YOL145C \| YOR123C \| YGL130W \| YGL019W \| YGL207W \| YGR063C \| YGR116W \| YIL035C \| YML010W \| YML069W \| YPL228W |
| complex_782 | YCR057C \| YDL148C \| YGR090W \| YHR196W \| YJR002W \| YOR310C \| YDR324C \| YER064C \| YPL212C \| YER082C \| YPL126W \| YGR128C \| YIL069C \| YMR229C \| YOR078W |
| complex_783 | YBR079C \| YDR429C \| YLR192C \| YMR146C \| YMR309C \| YOL087C \| YPR041W \| YER025W \| YJR007W \| YIL071C \| YOL117W \| YNL244C \| YOR361C \| YPL237W \| YPR086W |
| complex_784 | YBR081C \| YBR111W-A \| YMR223W \| YCL010C \| YDR145W \| YGL112C \| YGR252W \| YPL047W \| YDR159W \| YDR448W \| YBR278W \| YOL072W \| YOR257W \| YGL066W \| YIL006W |
| complex_787 | YBR247C \| YDL148C \| YGR090W \| YCL059C \| YCR057C \| YHR203C \| YJR123W \| YDR064W \| YHR196W \| YGL246C \| YBR084C-A \| YJR145C \| YJL109C \| YPR144C \| YPR137W |
| complex_792 | YBL050W \| YKL196C \| YOR036W \| YOR106W \| YDL077C \| YDR080W \| YDR323C \| YGL095C \| YGL124C \| YLR148W \| YAL002W \| YLR396C \| YML001W \| YMR231W \| YPL045W |
| complex_793 | YAL032C \| YDL209C \| YGL128C \| YKL095W \| YKL173W \| YLR117C \| YLR424W \| YMR213W \| YPR101W \| YBL010C \| YKR022C \| YBR086C \| YHR165C \| YGL120C \| YPL151C |
| complex_794 | YBL093C \| YDR308C \| YBR193C \| YBR253W \| YDL140C \| YGR104C \| YNR010W \| YOR174W \| YPR070W \| YGL043W \| YDR443C \| YGR097W \| YNL025C \| YPL042C \| YPL248C |
| complex_796 | YBR173C \| YGL011C \| YPR103W \| YDL188C \| YER012W \| YER094C \| YFR050C \| YGR135W \| YML092C \| YOL038W \| YOR157C \| YJL001W \| YHR200W \| YFL021W \| YLR068W |

| | |
|---|---|
| complex_797 | YBR113W \| YDL043C \| YDL030W \| YBR152W \| YFL017W-A \| YJL203W \| YLL036C \| YML049C \| YMR001C \| YMR213W \| YMR240C \| YPR182W \| YNL286W \| YMR288W \| YPL213W |
| complex_798 | YDL195W \| YDL212W \| YGL200C \| YIL004C \| YIL109C \| YLR208W \| YLR268W \| YML012W \| YPL085W \| YPR181C \| YLR026C \| YLR078C \| YJL004C \| YPL051W \| YPL218W |
| complex_80 | YCR052W \| YFR037C \| YGR056W \| YLR033W \| YLR321C \| YLR357W \| YMR033W \| YDR303C \| YGR275W \| YIL126W \| YKR008W \| YMR091C \| YPR034W \| YHR056C \| YMR224C |
| complex_802 | YBR096W \| YIL034C \| YBR173C \| YGL011C \| YPR103W \| YDL188C \| YER012W \| YER094C \| YFR050C \| YGR135W \| YOL038W \| YOR157C \| YHR200W \| YJL001W \| YML092C |
| complex_803 | YBR082C \| YER012W \| YKL010C \| YPR103W \| YBR173C \| YGL011C \| YDL188C \| YFR050C \| YGR135W \| YMR314W \| YOL038W \| YOR362C \| YGL048C \| YHR200W \| YML092C |
| complex_805 | YAL035W \| YMR260C \| YBR079C \| YDR429C \| YLR192C \| YMR146C \| YMR309C \| YPR041W \| YER025W \| YGR156W \| YNL062C \| YNL244C \| YOR361C \| YPL237W \| YPR086W |
| complex_806 | YBR247C \| YDR449C \| YJR002W \| YCL059C \| YCR057C \| YMR093W \| YDR324C \| YJL109C \| YDR447C \| YLR061W \| YLL011W \| YMR229C \| YPR144C \| YPL240C \| YPR137W |
| complex_809 | YBR089C-A \| YFR037C \| YGL133W \| YKR001C \| YLR033W \| YPL082C \| YBR245C \| YDL002C \| YLR176C \| YLR357W \| YMR091C \| YOR304W \| YDR303C \| YBR049C \| YIL126W |
| complex_810 | YER031C \| YFL038C \| YGL198W \| YNL044W \| YNL263C \| YER136W \| YGL210W \| YKR014C \| YNL093W \| YOR089C \| YFL005W \| YGR172C \| YNL272C \| YOR070C \| YML001W |
| complex_812 | YDR060W \| YGR103W \| YKR081C \| YMR049C \| YNL061W \| YOL077C \| YPL043W \| YPL211W \| YHR052W \| YKL014C \| YLL008W \| YNL110C \| YHR066W \| YNL226W \| YOL144W |
| complex_817 | YBL003C \| YGL150C \| YGL241W \| YHR099W \| YBR009C \| YBR010W \| YDR224C \| YDR225W \| YGR252W \| YJL081C \| YKR048C \| YOL012C \| YOR244W \| YFL024C \| YOR141C |
| complex_82 | YCR035C \| YDR280W \| YGR095C \| YGR195W \| YNL189W \| YOL021C \| YOR001W \| YDL111C \| YGR158C \| YHR069C \| YNL232W \| YDR083W \| YOL142W \| YHR081W \| YOR076C |
| complex_821 | YBR231C \| YDR190C \| YDR334W \| YFL039C \| YGR002C \| YJL081C \| YML041C \| YPL235W \| YDL002C \| YGL150C \| YPL129W \| YNL215W \| YNL059C \| YOR141C \| YBR287W |
| complex_826 | YBR289W \| YDR073W \| YEL009C \| YFL049W \| YHL025W \| YJL176C \| YKL109W \| YMR033W \| YNR023W \| YOR290C \| YPL016W \| YPL129W \| YDR370C \| YGR275W \| YPR034W |
| complex_828 | YDR060W \| YGR103W \| YKR081C \| YMR049C \| YNL061W \| YPL043W \| YPL211W \| YHR052W \| YKL014C \| YLL008W \| YMR290C \| YNL110C \| YHR066W \| YOL144W \| YML121W |
| complex_829 | YBR237W \| YDR073W \| YBR289W \| YEL009C \| YHL025W \| YJL176C \| YKL109W \| YMR033W \| YNR023W \| YOR290C \| YPL016W \| YPL129W \| YGR275W \| YPR034W \| YML068W |

| | |
|---|---|
| complex_830 | YBL093C \| YCR081W \| YGL025C \| YOL135C \| YBR253W \| YDL005C \| YDR443C \| YGR104C \| YNL236W \| YPR070W \| YER066W \| YHR147C \| YGL151W \| YNL025C \| YOR140W |
| complex_832 | YBR119W \| YDR235W \| YDR240C \| YGR013W \| YHR086W \| YIL061C \| YKL012W \| YLL036C \| YLR298C \| YML046W \| YDL087C \| YDR099W \| YGR214W \| YIL009W \| YOL130W |
| complex_833 | YBR082C \| YER012W \| YPR103W \| YDL188C \| YFR050C \| YGL011C \| YGR135W \| YGR253C \| YML092C \| YMR314W \| YOL038W \| YOR362C \| YDR177W \| YDR226W \| YHR200W |
| complex_837 | YBR154C \| YJL148W \| YJR063W \| YNL113W \| YPR010C \| YPR110C \| YDR156W \| YOR210W \| YKL144C \| YML010W \| YNL248C \| YOR340C \| YMR230W \| YOR341W \| YPR187W |
| complex_843 | YBR086C \| YDL209C \| YBR188C \| YDR416W \| YGR129W \| YCR063W \| YLL036C \| YMR001C \| YMR213W \| YPR101W \| YDR163W \| YJR050W \| YLR323C \| YPL151C \| YPR086W |
| complex_844 | YBL026W \| YCR077C \| YMR268C \| YNL147W \| YDR378C \| YER146W \| YOL149W \| YER112W \| YER131W \| YLL027W \| YFL066C \| YGL173C \| YJL124C \| YLR438C-A \| YJR022W |
| complex_845 | YCL059C \| YCR057C \| YDR449C \| YJR002W \| YLR129W \| YMR093W \| YDL166C \| YLR222C \| YDR324C \| YJL109C \| YER082C \| YIL019W \| YMR258C \| YJL069C \| YLR409C |
| complex_847 | YCL011C \| YDL084W \| YDR138W \| YDR381W \| YDR432W \| YHR167W \| YKL139W \| YNL112W \| YNL253W \| YPL169C \| YKL214C \| YML062C \| YNL004W \| YMR123W \| YNL139C |
| complex_848 | YBR288C \| YPL016W \| YBR289W \| YDR073W \| YEL009C \| YHL025W \| YJL176C \| YKL109W \| YMR033W \| YNR023W \| YOR290C \| YPL129W \| YGR275W \| YPR034W \| YGR006W |
| complex_849 | YCR067C \| YNL171C \| YDL195W \| YDL212W \| YGL200C \| YIL004C \| YIL109C \| YLR208W \| YLR268W \| YML012W \| YPL085W \| YPR181C \| YLR026C \| YLR078C \| YPL218W |
| complex_852 | YBR154C \| YDL140C \| YGR005C \| YPL129W \| YDR176W \| YDR457W \| YDR404C \| YJL140W \| YOL005C \| YGR186W \| YIL021W \| YJR017C \| YML010W \| YOR151C \| YPR187W |
| complex_854 | YDL232W \| YER019C-A \| YER087C-B \| YGL022W \| YJL002C \| YML019W \| YOR103C \| YEL002C \| YMR149W \| YJR091C \| YML055W \| YJR010C-A \| YOL016C \| YOR007C \| YOR085W |
| complex_857 | YBL072C \| YBR247C \| YCL059C \| YDR449C \| YOR078W \| YPR144C \| YJR002W \| YMR093W \| YDR324C \| YJL109C \| YER102W \| YLR140W \| YNL320W \| YPL126W \| YPR137W |
| complex_861 | YAL014C \| YAL030W \| YKL196C \| YMR197C \| YOR036W \| YBL050W \| YDR468C \| YLR026C \| YOL018C \| YOR106W \| YDR027C \| YGL095C \| YGL212W \| YHL031C \| YLR093C |
| complex_864 | YAL030W \| YER143W \| YGR009C \| YOR036W \| YPR032W \| YBL050W \| YDR468C \| YMR197C \| YOL018C \| YPL232W \| YDR027C \| YDR164C \| YOR327C \| YMR183C \| YNR049C |
| complex_865 | YBR131W \| YDL077C \| YGL124C \| YOR106W \| YDR080W \| YKL196C \| YLR148W \| YLR093C \| YAL002W \| YDR323C \| YLR396C \| YML001W \| YMR231W \| YPL045W \| YPL195W |

| | |
|---|---|
| complex_871 | YAL021C \| YDR443C \| YIL038C \| YNL288W \| YNR052C \| YPL042C \| YPR072W \| YCR093W \| YGR134W \| YDL165W \| YER068W \| YML098W \| YPL203W \| YLR177W \| YPL231W |
| complex_872 | YCR057C \| YDL148C \| YHR196W \| YJR002W \| YOR310C \| YDL014W \| YNL075W \| YPR137W \| YPR144C \| YDR324C \| YGR128C \| YPL126W \| YMR229C \| YDR300C \| YGL146C |
| complex_873 | YBR231C \| YDR190C \| YDR334W \| YFL039C \| YGR002C \| YJL081C \| YML041C \| YOL012C \| YPL235W \| YCR060W \| YHR034C \| YNL215W \| YLR241W \| YKR065C \| YNL107W |
| complex_874 | YER157W \| YFL038C \| YGR120C \| YLR026C \| YNL041C \| YNL051W \| YNL287W \| YGL005C \| YML071C \| YGL038C \| YGL223C \| YHL031C \| YLR078C \| YLR268W \| YPR105C |
| complex_875 | YDL195W \| YDL212W \| YGL200C \| YIL004C \| YIL109C \| YLR208W \| YLR268W \| YML012W \| YPL085W \| YPR181C \| YLR026C \| YHL039W \| YLR078C \| YDL166C \| YPL218W |
| complex_877 | YBR231C \| YDR190C \| YDR334W \| YFL039C \| YGR002C \| YJL081C \| YML041C \| YOL012C \| YPL235W \| YCR060W \| YHR034C \| YBR054W \| YNL215W \| YNL107W \| YOR040W |
| complex_878 | YAR007C \| YBL023C \| YDL029W \| YLR103C \| YLR274W \| YBR202W \| YCL061C \| YGL201C \| YIL150C \| YMR048W \| YDL017W \| YDR143C \| YEL032W \| YNL273W \| YPR019W |
| complex_879 | YBR154C \| YNL113W \| YOR116C \| YPR110C \| YDL150W \| YKR025W \| YDR045C \| YOR207C \| YJL011C \| YKL144C \| YNL151C \| YNR003C \| YPR190C \| YPR015C \| YHR046C |
| complex_880 | YCL011C \| YBR020W \| YDL084W \| YDR138W \| YDR381W \| YDR432W \| YHR167W \| YKL139W \| YNL112W \| YNL253W \| YKL214C \| YML062C \| YNL004W \| YNL139C \| YOL015W |
| complex_882 | YBR131W \| YDL077C \| YGL124C \| YOR106W \| YBR288C \| YGR261C \| YPL195W \| YDR080W \| YLR148W \| YAL002W \| YDR323C \| YLR396C \| YML001W \| YMR231W \| YPL045W |
| complex_883 | YBR154C \| YNL113W \| YOR116C \| YPR110C \| YDL150W \| YKR025W \| YDR045C \| YOR207C \| YDR330W \| YBL034C \| YJL011C \| YKL144C \| YNL151C \| YNR003C \| YPR190C |
| complex_884 | YAL032C \| YDL209C \| YDR364C \| YDR416W \| YGR278W \| YKL095W \| YLR117C \| YLR424W \| YMR001C \| YMR213W \| YPR101W \| YHR193C \| YDR252W \| YPL037C \| YPL151C |
| complex_885 | YBR087W \| YFR027W \| YOR144C \| YBR088C \| YOR217W \| YER173W \| YJR068W \| YNL290W \| YOL094C \| YGL234W \| YHR191C \| YJL115W \| YBL015W \| YMR078C \| YOR298W |
| complex_886 | YBR154C \| YFL023W \| YNL113W \| YOR116C \| YPR110C \| YCL017C \| YDL150W \| YKR025W \| YDR045C \| YOR207C \| YJL011C \| YKL144C \| YNL151C \| YNR003C \| YPR190C |
| complex_887 | YAL021C \| YDR443C \| YGR092W \| YIL038C \| YNL288W \| YNR052C \| YPL042C \| YPR072W \| YCR093W \| YGR134W \| YDL165W \| YER068W \| YNL278W \| YLR216C \| YPR119W |
| complex_891 | YBR247C \| YDL148C \| YGR090W \| YHR203C \| YCR057C \| YHR196W \| YOR310C \| YDR447C \| YER148W \| YIL069C \| YJL109C \| YJR145C \| YPR144C \| YML024W \| YPR137W |

| | |
|---|---|
| complex_893 | YCL059C \| YCR057C \| YDR449C \| YGR090W \| YJR002W \| YLR129W \| YDR324C \| YJL109C \| YER082C \| YLR222C \| YPL212C \| YJL069C \| YJL135W \| YGL057C \| YLR409C |
| complex_894 | YER031C \| YFL038C \| YGL198W \| YNL044W \| YNL263C \| YER136W \| YGL210W \| YKR014C \| YLR262C \| YNL093W \| YOR089C \| YFL005W \| YGR172C \| YNL272C \| YJL204C |
| complex_898 | YAL055W \| YDL065C \| YGL153W \| YLR191W \| YOL147C \| YDL078C \| YDR244W \| YMR026C \| YDR265W \| YDR329C \| YJL210W \| YLR284C \| YNL117W \| YNL214W \| YOL044W |
| complex_900 | YBL041W \| YDL020C \| YOL038W \| YPR103W \| YDL188C \| YER012W \| YGL011C \| YGR135W \| YMR314W \| YOR362C \| YHR200W \| YFR052W \| YDR273W \| YLR024C \| YML092C |
| complex_901 | YBL023C \| YBR202W \| YCL061C \| YGL201C \| YIL150C \| YJL194W \| YLR103C \| YLR274W \| YMR048W \| YCR086W \| YDL017W \| YEL032W \| YNL273W \| YPR019W \| YHR134W |
| complex_904 | YBR127C \| YDL185W \| YDR328C \| YOR270C \| YDR202C \| YEL051W \| YGR020C \| YHR039C-A \| YOR332W \| YJR033C \| YJR076C \| YKL048C \| YKL080W \| YMR054W \| YPR119W |
| complex_905 | YAL042W \| YAR002C-A \| YML067C \| YML130C \| YGL200C \| YIL004C \| YLR026C \| YML012W \| YIL109C \| YLR208W \| YPR181C \| YLR078C \| YLR268W \| YPL085W \| YPL218W |
| complex_908 | YBR231C \| YDR190C \| YDR334W \| YFL039C \| YJL081C \| YML041C \| YPL235W \| YDL002C \| YER092W \| YGL150C \| YLR052W \| YPL129W \| YNL215W \| YNL059C \| YOR141C |
| complex_912 | YBR142W \| YDR060W \| YKL014C \| YLR009W \| YMR049C \| YMR290C \| YNL061W \| YPL043W \| YBR269C \| YLR276C \| YDL164C \| YKR081C \| YHR052W \| YHR066W \| YOR272W |
| complex_913 | YAL054C \| YHR041C \| YLR049C \| YNL189W \| YBL093C \| YGL025C \| YOL051W \| YOL135C \| YBR193C \| YBR253W \| YDL005C \| YGR104C \| YHR058C \| YLR071C \| YEL066W |
| complex_918 | YAL034W-A \| YBR211C \| YDR254W \| YIR010W \| YPL018W \| YPR046W \| YBR107C \| YER081W \| YGR179C \| YJR135C \| YLR381W \| YEL077C \| YFR053C \| YAL060W \| YPL233W |
| complex_919 | YBL026W \| YCR077C \| YDL160C \| YDR381W \| YMR237W \| YNL147W \| YDR378C \| YER146W \| YOL149W \| YER112W \| YGL173C \| YJL124C \| YLR438C-A \| YJR022W \| YLR120C |
| complex_92 | YBL093C \| YDR308C \| YGL025C \| YOL135C \| YBR193C \| YBR253W \| YDL005C \| YGL253W \| YGR104C \| YHR058C \| YLR071C \| YMR112C \| YNR010W \| YOR174W \| YPR070W |
| complex_921 | YDL195W \| YGL200C \| YIL004C \| YIL109C \| YLR208W \| YLR268W \| YML012W \| YPL085W \| YPR181C \| YDR192C \| YNL049C \| YLR026C \| YHR098C \| YLR078C \| YPL218W |
| complex_928 | YBR079C \| YDR429C \| YLR192C \| YMR146C \| YMR309C \| YOL087C \| YPR041W \| YER025W \| YLR359W \| YBR276C \| YNL062C \| YNL244C \| YOR361C \| YPL237W \| YPR086W |
| complex_931 | YBR204C \| YPL096W \| YDL097C \| YDR363W-A \| YDR394W \| YFR052W \| YGL048C \| YHR027C \| YKL145W \| YOR117W \| YOR259C \| YDR427W \| YMR276W \| YEL037C \| YDR314C |

| | |
|---|---|
| complex_932 | YBR079C \| YLR192C \| YPR041W \| YDR021W \| YJR007W \| YDR429C \| YER025W \| YMR146C \| YIL071C \| YCR053W \| YMR309C \| YOL087C \| YOR361C \| YNL244C \| YPL237W |
| complex_933 | YAL055W \| YDL065C \| YGR133W \| YDR034C \| YGL153W \| YOL147C \| YDR244W \| YLR191W \| YMR026C \| YDR265W \| YDR329C \| YGR218W \| YJL210W \| YNL214W \| YOL044W |
| complex_934 | YBR131W \| YDL077C \| YGL124C \| YOR106W \| YDR080W \| YKL196C \| YLR148W \| YGL212W \| YGR261C \| YPL195W \| YLR093C \| YLR396C \| YML001W \| YMR231W \| YPL045W |
| complex_936 | YBL050W \| YDR189W \| YDR468C \| YKL196C \| YLR026C \| YLR268W \| YMR197C \| YOL018C \| YOR036W \| YOR106W \| YGL095C \| YGL212W \| YHL031C \| YLR093C \| YFL056C |
| complex_937 | YAL034W-A \| YER018C \| YGL093W \| YIL144W \| YIR010W \| YJR112W \| YOL069W \| YDR318W \| YKL089W \| YMR117C \| YDR356W \| YFL008W \| YGR130C \| YKR054C \| YPL233W |
| complex_938 | YDL116W \| YGL092W \| YDR192C \| YJR042W \| YKR082W \| YMR047C \| YGL100W \| YLR208W \| YGL172W \| YGR119C \| YPL125W \| YPR083W \| YKL057C \| YKL068W \| YMR294W |
| complex_941 | YDL007W \| YDR363W-A \| YFR004W \| YFR052W \| YGL048C \| YHR027C \| YDL097C \| YHR200W \| YKL145W \| YDL227C \| YER143W \| YLL013C \| YML088W \| YDR427W \| YEL037C |
| complex_950 | YBL050W \| YDR189W \| YGL098W \| YKL196C \| YLR026C \| YLR268W \| YOR075W \| YLR440C \| YDR498C \| YHL031C \| YIL004C \| YKL006C-A \| YLR078C \| YMR316W \| YER125W |
| complex_951 | YBL079W \| YMR047C \| YOR098C \| YDL116W \| YGL092W \| YDR192C \| YJR042W \| YKR082W \| YGL100W \| YLR208W \| YGL172W \| YGR119C \| YKL057C \| YML103C \| YKL068W |
| complex_952 | YDL188C \| YER012W \| YFR050C \| YGL011C \| YGR135W \| YGR253C \| YML092C \| YMR314W \| YOL038W \| YOR362C \| YPR103W \| YDR328C \| YHR200W \| YGL149W \| YIL009C-A |
| complex_954 | YBL026W \| YCR077C \| YLR264W \| YDR378C \| YOL149W \| YEL015W \| YER112W \| YGL173C \| YJL124C \| YLR438C-A \| YNL118C \| YGL222C \| YJR022W \| YOR167C \| YBR094W |
| complex_956 | YBR188C \| YDL209C \| YDR416W \| YGR129W \| YGL070C \| YDR199W \| YER053C \| YJR050W \| YLL036C \| YLR117C \| YMR001C \| YMR213W \| YPR101W \| YLR326W \| YPL151C |
| complex_958 | YAL009W \| YHR004C \| YBR289W \| YDR073W \| YEL009C \| YJL176C \| YKL109W \| YMR033W \| YNR023W \| YOR290C \| YPL016W \| YPL129W \| YHL025W \| YPR034W \| YMR165C |
| complex_959 | YBR289W \| YDR073W \| YEL009C \| YHL025W \| YJL176C \| YKL109W \| YMR033W \| YNR023W \| YOR290C \| YPL016W \| YPL129W \| YER161C \| YLR389C \| YPL125W \| YPR034W |
| complex_961 | YBR146W \| YBR251W \| YHL004W \| YDR036C \| YDR041W \| YDR175C \| YDR347W \| YGL129C \| YIL093C \| YDR337W \| YKL155C \| YJR113C \| YNL137C \| YNL306W \| YPL118W |
| complex_968 | YCL011C \| YDL084W \| YDR138W \| YDR381W \| YDR432W \| YHR167W \| YKL139W \| YNL112W \| YNL253W \| YFL036W \| YMR228W \| YKL214C \| YML062C \| YNL004W \| YNL139C |

| | |
|---|---|
| complex_969 | YCL011C \| YDL084W \| YDR138W \| YDR381W \| YDR432W \| YHR167W \| YKL139W \| YNL112W \| YNL253W \| YKL214C \| YML062C \| YNL004W \| YNL097C \| YNL139C \| YOL020W |
| complex_97 | YCR057C \| YDR449C \| YGR090W \| YHR196W \| YJR002W \| YMR093W \| YOR310C \| YDR324C \| YJL109C \| YER082C \| YPL126W \| YGR128C \| YJL069C \| YLR409C \| YMR229C |
| complex_972 | YCL011C \| YBR020W \| YDL084W \| YDR120C \| YDR138W \| YDR381W \| YDR432W \| YHR167W \| YJR123W \| YKL139W \| YNL112W \| YNL253W \| YML062C \| YNL004W \| YNL139C |
| complex_973 | YBR231C \| YDR190C \| YDR334W \| YFL039C \| YGR002C \| YJL081C \| YML041C \| YOL012C \| YPL235W \| YFL024C \| YNR031C \| YNL079C \| YDL072C \| YNL107W \| YPL240C |
| complex_976 | YDR510W \| YER041W \| YKR081C \| YFL002C \| YLR009W \| YMR049C \| YNL061W \| YGL111W \| YGR103W \| YNL110C \| YPL093W \| YHR052W \| YOL077C \| YHR066W \| YLR449W |
| complex_978 | YBL020W \| YDL140C \| YBR154C \| YGR005C \| YPL129W \| YDR404C \| YJL140W \| YGR186W \| YIL021W \| YJR017C \| YML010W \| YMR277W \| YOR151C \| YPR086W \| YPR187W |
| complex_98 | YAL032C \| YDL209C \| YDR416W \| YKL095W \| YLL036C \| YLR117C \| YLR424W \| YMR001C \| YMR213W \| YPR101W \| YBR188C \| YGR129W \| YJR050W \| YPL151C \| YPL213W |
| complex_980 | YBR119W \| YDR240C \| YGR013W \| YHR086W \| YIL061C \| YKL012W \| YLR298C \| YMR125W \| YPL178W \| YDR432W \| YGL049C \| YIR001C \| YNL016W \| YPL190C \| YNL251C |
| complex_981 | YAL043C \| YGL044C \| YGR156W \| YDR228C \| YDR301W \| YPR049C \| YGL180W \| YGR047C \| YKL059C \| YLR115W \| YLR277C \| YMR061W \| YLR423C \| YPR185W \| YOR250C |
| complex_985 | YAL014C \| YAL030W \| YKL196C \| YMR197C \| YOR036W \| YER143W \| YBL050W \| YDR189W \| YDR468C \| YLR026C \| YOL018C \| YPL232W \| YOR327C \| YGL095C \| YMR017W |
| complex_99 | YAL043C \| YGR156W \| YDR195W \| YDR301W \| YJR093C \| YKL059C \| YKR002W \| YLR115W \| YLR277C \| YMR061W \| YNL317W \| YPR107C \| YDR228C \| YML030W \| YOR250C |
| complex_995 | YAR042W \| YMR047C \| YER006W \| YLR074C \| YER126C \| YKR081C \| YLR009W \| YNL110C \| YNR053C \| YGR245C \| YNL002C \| YPL093W \| YLR397C \| YPL013C \| YPR016C |

**Supplementary Table 7. Predicted yeast complexes from yeast and fly interaction datasets.**

| | |
|---|---|
| complex_3298 | YDL007W \| YGL004C \| YDL029W \| YPL140C \| YDR394W \| YGL048C \| YGR232W \| YKL145W \| YMR216C \| YOR117W \| YOR259C \| YLR433C \| YKL190W \| YML057W \| YPR178W |
| complex_3295 | YBL045C \| YPR054W \| YPR191W \| YDL029W \| YNL189W \| YDR394W \| YOR027W \| YGL137W \| YHR030C \| YLR216C \| YPL240C \| YLR069C \| YJL138C \| YNR011C \| YPR178W |
| complex_3294 | YBL075C \| YLR362W \| YNL298W \| YBR160W \| YCR065W \| YDL155W \| YGR092W \| YHL007C \| YIL106W \| YJL187C \| YKL129C \| YNL102W \| YPR119W \| YHR018C \| YMR001C |
| complex_3290 | YBL030C \| YDL029W \| YGL137W \| YOR212W \| YDR188W \| YBR198C \| YDR142C \| YJL106W \| YJL164C \| YKL166C \| YNL317W \| YPL203W \| YDR212W \| YIL142W \| YJR064W |
| complex_3288 | YBL016W \| YGL158W \| YBR160W \| YHL007C \| YKL129C \| YNL298W \| YOR127W \| YOR204W \| YDL159W \| YAL041W \| YLR362W \| YGR159C \| YJL187C \| YLR229C \| YNL053W |
| complex_3287 | YBL045C \| YKL166C \| YDR490C \| YER165W \| YPL203W \| YGL137W \| YLR216C \| YNR032W \| YPL031C \| YHR205W \| YKL126W \| YIL033C \| YMR022W \| YJL164C \| YFL033C |
| complex_3284 | YBL045C \| YBR028C \| YDL101C \| YER165W \| YGL096W \| YPL203W \| YGL137W \| YLR216C \| YHR135C \| YIL033C \| YMR022W \| YJL164C \| YKL166C \| YMR226C \| YPL153C |
| complex_3283 | YBL045C \| YGR159C \| YBR160W \| YDR285W \| YPR119W \| YDR432W \| YER165W \| YHR086W \| YIR001C \| YNL016W \| YOR204W \| YGL049C \| YGR162W \| YDR235W \| YJR059W |
| complex_3282 | YBR160W \| YER008C \| YIL046W \| YMR001C \| YNL298W \| YOR127W \| YCL024W \| YJL187C \| YGR092W \| YGR152C \| YAL041W \| YLR229C \| YHL007C \| YLR212C \| YNL007C |
| complex_3281 | YBL105C \| YBR160W \| YJL095W \| YOR231W \| YPR119W \| YCR065W \| YER165W \| YGL116W \| YHR030C \| YDL029W \| YMR109W \| YJL194W \| YKL129C \| YOL100W \| YPL140C |
| complex_2199 | YBL105C \| YJL095W \| YOR231W \| YBR160W \| YCR065W \| YDL155W \| YGR092W \| YLR131C \| YPR119W \| YDR216W \| YDL029W \| YIL106W \| YNL161W \| YMR109W \| YKL129C |
| complex_2198 | YBL075C \| YBR085W \| YBR155W \| YER081W \| YLR310C \| YOR254C \| YEL030W \| YNL161W \| YHL007C \| YHR102W \| YHR030C \| YKL073W \| YJL034W \| YNL007C \| YER036C |
| complex_2196 | YDL029W \| YDR394W \| YOR259C \| YPL140C \| YDL047W \| YGL004C \| YDL007W \| YGL048C \| YKL145W \| YOR117W \| YGR232W \| YHR030C \| YJL095W \| YKL129C \| YNL250W |
| complex_2195 | YBL075C \| YHL007C \| YBR160W \| YJL187C \| YKL210W \| YBR200W \| YPR119W \| YCR065W \| YKL129C \| YHR018C \| YKL116C \| YLR096W \| YLR362W \| YNL298W \| YPL119C |
| complex_2194 | YBL105C \| YOR231W \| YBR160W \| YHL007C \| YCR065W \| YDR356W \| YPR119W \| YER165W \| YJL095W \| YAL041W \| YJL187C \| YNL298W \| YKL129C \| YOR127W \| YOL100W |
| complex_2193 | YBL105C \| YOL100W \| YBR160W \| YNL317W \| YOL123W \| YPR119W \| YDR432W \| YGR159C \| YHR086W \| YNL016W \| YOR204W \| YER165W \| YJL033W \| YKL025C \| YGL049C |
| complex_2192 | YAL005C \| YDR477W \| YLR310C \| YNL007C \| YNL209W \| YNR047W \| YBR160W \| YCR065W \| YHR018C \| YKL129C \| YPR119W \| YCR091W \| YER129W \| YLL024C \| YPL106C |

| | |
|---|---|
| complex_2191 | YBR109C \| YOR039W \| YOR326W \| YDL195W \| YGL019W \| YIL035C \| YIL131C \| YDR356W \| YLR212C \| YOR061W \| YIL118W \| YLR399C \| YOL004W \| YOL145C \| YPL110C |
| complex_2190 | YAL005C \| YDL229W \| YER165W \| YNL007C \| YPL106C \| YPR119W \| YDR477W \| YDR432W \| YGR159C \| YKR095W \| YOR204W \| YER028C \| YOL123W \| YFL018C |
| complex_3278 | YAL005C \| YBL075C \| YLR310C \| YNL064C \| YPL106C \| YBL016W \| YBL047C \| YBR109C \| YBR094W \| YOR061W \| YEL034W \| YER165W \| YPL204W \| YLL024C \| YCR088W |
| complex_3277 | YBL084C \| YGL003C \| YMR001C \| YBL105C \| YJL095W \| YOR231W \| YBR160W \| YDL155W \| YGR092W \| YJL060W \| YJL187C \| YOL100W \| YPR119W \| YER165W \| YPL151C |
| complex_3273 | YAL005C \| YLR310C \| YNL007C \| YBL016W \| YMR139W \| YBL075C \| YER129W \| YJR032W \| YLR362W \| YDL159W \| YGR040W \| YDR477W \| YGL158W \| YLL039C \| YGR159C |
| complex_3272 | YBL105C \| YBR160W \| YER165W \| YGL003C \| YJL095W \| YPR119W \| YER008C \| YJL187C \| YMR001C \| YMR304W \| YFL005W \| YHR030C \| YJL060W \| YJL141C \| YKR048C |
| complex_3271 | YBL045C \| YCR065W \| YMR001C \| YBR160W \| YGL003C \| YJL187C \| YER165W \| YKR036C \| YPL042C \| YPR119W \| YGL137W \| YGR092W \| YAR019C \| YIR005W \| YJL060W |
| complex_2189 | YBL045C \| YPR054W \| YPR191W \| YBL105C \| YBR160W \| YDL017W \| YDR394W \| YLR216C \| YOR117W \| YER165W \| YKL166C \| YGL137W \| YHR030C \| YGR092W \| YOR027W |
| complex_2187 | YBR160W \| YDL029W \| YLR131C \| YCR065W \| YDL155W \| YDR146C \| YPR119W \| YGR092W \| YHL007C \| YIL106W \| YNL161W \| YPR111W \| YKL048C \| YKL129C \| YMR109W |
| complex_2182 | YCR065W \| YBR160W \| YDL155W \| YDR146C \| YNL288W \| YPR119W \| YDR285W \| YDL028C \| YDR356W \| YIL149C \| YGR092W \| YIL106W \| YPR111W \| YKL129C \| YKR095W |
| complex_2181 | YBL045C \| YBR160W \| YGL137W \| YOL123W \| YBL105C \| YER165W \| YOL100W \| YPR119W \| YDR432W \| YGR159C \| YHR086W \| YIR001C \| YOR204W \| YGR092W \| YNL016W |
| complex_1099 | YAL005C \| YLL024C \| YLR310C \| YNL007C \| YPL106C \| YGR040W \| YLL039C \| YHL002W \| YIL033C \| YPL203W \| YJL164C \| YKL166C \| YLR216C \| YNL027W \| YNL209W |
| complex_1098 | YBL075C \| YAL005C \| YER081W \| YOR027W \| YBR155W \| YBR160W \| YDL017W \| YDL229W \| YDR356W \| YDR477W \| YPL106C \| YPR119W \| YLL024C \| YNL209W \| YEL030W |
| complex_1097 | YAL005C \| YBR155W \| YIL061C \| YOR254C \| YPL106C \| YPL203W \| YDL229W \| YHR079C \| YIL033C \| YJL034W \| YJL164C \| YKL166C \| YMR022W \| YKL073W \| YNL027W |
| complex_1091 | YAL005C \| YCL011C \| YLR310C \| YNL007C \| YOR027W \| YPL106C \| YBL016W \| YBL075C \| YCR073C \| YJL128C \| YDR146C \| YLL024C \| YLR113W \| YLR362W \| YNR031C |
| complex_1090 | YBR160W \| YGR092W \| YJL187C \| YMR001C \| YNL298W \| YCR002C \| YCL024W \| YDL225W \| YDR507C \| YHR107C \| YAR019C \| YKL101W \| YIL106W \| YJR076C \| YLR314C |
| complex_3268 | YBL105C \| YJL095W \| YOR231W \| YPL140C \| YBR109C \| YMR109W \| YDR394W \| YDL029W \| YJR065C \| YKL073W \| YHR018C \| YHR030C \| YKL129C \| YPR119W \| YLR096W |
| complex_3267 | YDR394W \| YKL145W \| YLR216C \| YOR117W \| YGL011C \| YMR314W \| YOL038W \| YGL137W \| YBR080C \| YNR032W \| YOR362C \| YML092C \| YLL024C \| YMR308C \| YOR027W |
| complex_3266 | YAL005C \| YLL024C \| YLR310C \| YBL016W \| YDL159W \| YGL158W \| YLR362W \| YBL075C \| YCR073C \| YJR032W \| YGR040W \| YER165W \| YLR113W \| YJL128C \| YNR031C |

| | |
|---|---|
| complex_3264 | YBR160W \| YBR200W \| YCR065W \| YDL155W \| YGR092W \| YHL007C \| YJL187C \| YKL129C \| YLR131C \| YLR362W \| YMR001C \| YNL102W \| YNL298W \| YPR119W \| YNL161W |
| complex_3263 | YAL005C \| YLR310C \| YNL064C \| YPL106C \| YBL016W \| YGR058W \| YLR113W \| YLR362W \| YBL075C \| YGL158W \| YHR018C \| YGR040W \| YJL128C \| YLL024C \| YLR248W |
| complex_3262 | YAR019C \| YGR092W \| YBL045C \| YCR065W \| YBR160W \| YJL187C \| YCR084C \| YPL042C \| YER165W \| YKR036C \| YPR119W \| YIL106W \| YJL060W \| YMR001C \| YPR111W |
| complex_3261 | YBL030C \| YGL137W \| YBR160W \| YER129W \| YGL003C \| YPR120C \| YDR212W \| YDR477W \| YJL106W \| YJL164C \| YKR036C \| YNL317W \| YGR092W \| YIL142W \| YPL042C |
| complex_2177 | YBL105C \| YBR160W \| YGL003C \| YCR065W \| YOR231W \| YER165W \| YJL095W \| YPR119W \| YPR120C \| YPR191W \| YHR030C \| YJL060W \| YLR096W \| YNL068C \| YOL100W |
| complex_2174 | YDL175C \| YOR204W \| YPR191W \| YDR432W \| YER165W \| YGR159C \| YIL061C \| YIR001C \| YNL016W \| YOL123W \| YPR119W \| YGL049C \| YKR059W \| YGR162W \| YHR086W |
| complex_2173 | YBL105C \| YBR160W \| YER165W \| YIL035C \| YCR065W \| YML010W \| YOR061W \| YGL019W \| YGL254W \| YIL118W \| YIL131C \| YML074C \| YOR039W \| YLR069C \| YOL145C |
| complex_2172 | YBL045C \| YBR160W \| YCR065W \| YBL105C \| YOL100W \| YER165W \| YGR159C \| YJR059W \| YPL209C \| YPR119W \| YJL095W \| YKL129C \| YNR047W \| YCR091W \| YOR231W |
| complex_2170 | YBL105C \| YER165W \| YPL151C \| YBR034C \| YIL079C \| YOL123W \| YBR065C \| YMR001C \| YPR119W \| YDR432W \| YOR204W \| YGL044C \| YGR159C \| YKL025C \| YOL100W |
| complex_1089 | YAL005C \| YDR477W \| YER129W \| YGL011C \| YGR135W \| YGR253C \| YML092C \| YMR314W \| YNL209W \| YOL038W \| YOR362C \| YER012W \| YLR276C \| YCR057C \| YMR308C |
| complex_1087 | YAL005C \| YBL075C \| YBR155W \| YJR032W \| YLR310C \| YOR027W \| YPL153C \| YDL229W \| YDR477W \| YNL209W \| YLL024C \| YPL106C \| YPL240C \| YLR347C \| YMR190C |
| complex_1086 | YAL005C \| YDL229W \| YLR310C \| YNL007C \| YNL209W \| YDR477W \| YDR523C \| YIL033C \| YLR216C \| YER028C \| YLL024C \| YPL106C \| YKL166C \| YLR347C \| YPL203W |
| complex_1085 | YAL005C \| YBL075C \| YBR155W \| YDL229W \| YDR477W \| YLL024C \| YLR310C \| YNL209W \| YOR027W \| YPL106C \| YER081W \| YEL030W \| YOR101W \| YPR054W |
| complex_1084 | YAL005C \| YBR155W \| YDL229W \| YER165W \| YLR310C \| YNL007C \| YPL106C \| YPR128C \| YMR216C \| YDR432W \| YGR159C \| YKR095W \| YDR477W \| YER028C |
| complex_1081 | YAL005C \| YDR356W \| YLL016W \| YLL024C \| YLR310C \| YNL064C \| YOR027W \| YBL016W \| YNL209W \| YPL106C \| YBL075C \| YDL229W \| YDR477W \| YNL098C \| YOR101W |
| complex_1080 | YAL005C \| YNL007C \| YNL209W \| YBR160W \| YDR146C \| YDR356W \| YKR095W \| YNL041C \| YOR195W \| YPR119W \| YDL229W \| YPL106C \| YDR477W \| YFR031C \| YIL149C |
| complex_3259 | YBL045C \| YDR477W \| YOL123W \| YPR054W \| YDR432W \| YGL173C \| YGR159C \| YHR086W \| YKL139W \| YOR204W \| YPR119W \| YFL029C \| YGL096W \| YML046W \| YOR319W |
| complex_3258 | YDL074C \| YFR031C \| YJL074C \| YKR009C \| YOL069W \| YOR195W \| YDR356W \| YBR160W \| YFL037W \| YML085C \| YPR119W \| YFL008W \| YPR141C \| YIL144W \| YLR086W |
| complex_3254 | YAL032C \| YBL105C \| YER025W \| YKL095W \| YLR117C \| YMR001C \| YMR213W \| YPL151C \| YAR019C \| YPL240C \| YGL120C \| YHR169W \| YJL138C \| YLR347C \| YNL189W |

| | |
|---|---|
| complex_3252 | YBR160W \| YCR065W \| YDL155W \| YER008C \| YGL003C \| YGR092W \| YJR059W \| YKL129C \| YMR001C \| YNL102W \| YPR119W \| YIL106W \| YIL112W \| YJL187C \| YNL298W |
| complex_3251 | YBL045C \| YGR159C \| YIL131C \| YDL155W \| YBR160W \| YGR092W \| YDR432W \| YHR086W \| YOL123W \| YOR204W \| YER165W \| YKL025C \| YKR036C \| YJL060W \| YPL042C |
| complex_3250 | YBR109C \| YGL019W \| YOR039W \| YOR061W \| YGL254W \| YIL035C \| YIL118W \| YIL131C \| YML010W \| YML074C \| YPL141C \| YHR205W \| YDR490C \| YKL126W \| YOL145C |
| complex_2166 | YBL105C \| YJL095W \| YKR024C \| YPR119W \| YDR432W \| YER165W \| YGL173C \| YKL139W \| YOR204W \| YHR082C \| YGR159C \| YDL029W \| YMR109W \| YKL129C \| YOR231W |
| complex_2163 | YAR019C \| YHR169W \| YBR160W \| YCR065W \| YDL155W \| YKL129C \| YNL102W \| YDR146C \| YPR119W \| YGR092W \| YIL106W \| YJL187C \| YNL068C \| YPR111W \| YMR001C |
| complex_2160 | YAR019C \| YGR092W \| YNL161W \| YPL131W \| YBR160W \| YCR065W \| YHL007C \| YKL129C \| YLR131C \| YDL155W \| YDR146C \| YNL288W \| YPR119W \| YFL034C-B \| YIL106W |
| complex_1077 | YDL195W \| YGL019W \| YIL131C \| YOR061W \| YER007C-A \| YJR014W \| YGL254W \| YIL118W \| YLR399C \| YML010W \| YOL004W \| YOR039W \| YIL035C \| YOL145C \| YPL204W |
| complex_3249 | YBR160W \| YCR065W \| YHR018C \| YJL187C \| YKL129C \| YPR119W \| YBR200W \| YCL024W \| YDR054C \| YHL007C \| YHR064C \| YAR018C \| YIL046W \| YLR362W \| YNL298W |
| complex_3248 | YBR160W \| YAL019W \| YBL105C \| YER008C \| YGR238C \| YHR158C \| YIL046W \| YJL187C \| YLR096W \| YOL100W \| YCL024W \| YDR146C \| YPR119W \| YER165W \| YJL060W |
| complex_199 | YAL005C \| YBR085W \| YDR356W \| YER081W \| YER165W \| YLL024C \| YLR310C \| YNL064C \| YOR027W \| YPL106C \| YBL075C \| YDL229W \| YDR477W \| YNL041C \| YER028C |
| complex_3245 | YAL005C \| YBL075C \| YDR477W \| YER129W \| YLR310C \| YMR104C \| YNL209W \| YOR027W \| YPL106C \| YDL229W \| YDR356W \| YPR119W \| YER028C \| YPL240C \| YLL024C |
| complex_3243 | YBR160W \| YER008C \| YGL003C \| YMR001C \| YNL298W \| YPR119W \| YPR120C \| YMR304W \| YER165W \| YHL035C \| YJL060W \| YJL187C \| YKL101W \| YKL190W \| YKR048C |
| complex_3242 | YBL045C \| YPR054W \| YPR191W \| YBR160W \| YFL029C \| YLR096W \| YDL108W \| YLR113W \| YGL137W \| YKL081W \| YML057W \| YHR030C \| YHR086W \| YKL173W \| YLR147C |
| complex_3241 | YBL016W \| YDL159W \| YNL178W \| YGR040W \| YDR212W \| YIL142W \| YJL106W \| YKR036C \| YLL011W \| YOR212W \| YER020W \| YJR064W \| YHR005C \| YJL164C \| YLR362W |
| complex_3240 | YAR019C \| YGR092W \| YNL161W \| YBR160W \| YDL155W \| YHL007C \| YIL131C \| YKL129C \| YLR131C \| YPR111W \| YPR119W \| YDR146C \| YFL034C-B \| YIL106W \| YDL029W |
| complex_2159 | YAL005C \| YBL075C \| YLL024C \| YLR310C \| YNL064C \| YPR119W \| YBR160W \| YCR065W \| YJL187C \| YKL129C \| YCL024W \| YHR018C \| YDR054C \| YIL046W \| YNL298W |
| complex_2154 | YBR200W \| YBR160W \| YHL007C \| YJL187C \| YLR362W \| YNL298W \| YPR119W \| YCR065W \| YDL155W \| YGR092W \| YIL106W \| YNL161W \| YPR111W \| YKL129C \| YMR109W |
| complex_2153 | YBL105C \| YBR160W \| YOL100W \| YPL151C \| YBR065C \| YMR001C \| YPR119W \| YGR092W \| YNL102W \| YCR065W \| YER165W \| YJL060W \| YJL187C \| YKL025C \| YKL129C |
| complex_1068 | YBL045C \| YBR160W \| YCR065W \| YBL105C \| YER165W \| YJL095W \| YOL100W \| YOR231W \| YPR119W \| YBR200W \| YHL007C \| YJL187C \| YNL298W \| YKL129C \| YLR362W |

| | |
|---|---|
| complex_1067 | YBL105C \| YJL095W \| YOR231W \| YPL151C \| YBR065C \| YLR117C \| YLR424W \| YMR213W \| YER013W \| YKL173W \| YMR001C \| YGL120C \| YHR086W \| YLR147C \| YDR364C |
| complex_1066 | YAL005C \| YBL075C \| YLR310C \| YNL064C \| YOR027W \| YPL106C \| YPL240C \| YAL040C \| YHR018C \| YJL013C \| YBL016W \| YDL159W \| YGR040W \| YLL024C \| YLR362W |
| complex_1061 | YBR109C \| YGL019W \| YLR212C \| YOR039W \| YFR024C-A \| YML010W \| YGL254W \| YIL035C \| YIL118W \| YIL131C \| YML074C \| YOL004W \| YJR014W \| YOL145C \| YOR061W |
| complex_3239 | YBR079C \| YDR146C \| YBR160W \| YDL108W \| YFL029C \| YGL003C \| YGR092W \| YDL155W \| YER165W \| YPR119W \| YIL106W \| YMR001C \| YPR111W \| YJL060W \| YLR113W |
| complex_3237 | YBR065C \| YMR001C \| YPR119W \| YBR160W \| YER008C \| YGL003C \| YGR092W \| YIL046W \| YJL187C \| YNL068C \| YPL209C \| YPR120C \| YMR304W \| YKL025C \| YIL126W |
| complex_3233 | YBR109C \| YGL019W \| YGR270W \| YKL210W \| YLR212C \| YOR039W \| YOR061W \| YGL254W \| YIL035C \| YIL131C \| YML010W \| YML074C \| YOL145C |
| complex_3232 | YDL195W \| YGL019W \| YIL035C \| YIL131C \| YOR061W \| YER148W \| YLR399C \| YOR039W \| YIL118W \| YOL004W \| YHR193C \| YPL037C \| YPL204W \| YOR326W \| YOL145C |
| complex_3231 | YBR160W \| YCR065W \| YKL129C \| YMR001C \| YNL102W \| YDL003W \| YDL074C \| YJL074C \| YFL008W \| YFR031C \| YJL187C \| YNL298W \| YPR119W \| YLR086W \| YOR195W |
| complex_2142 | YAR019C \| YGR092W \| YBR160W \| YDL007W \| YGL048C \| YKL145W \| YLR424W \| YOR259C \| YDL029W \| YNL161W \| YDL155W \| YDR394W \| YOR117W \| YIL007C \| YIL106W |
| complex_2140 | YAL024C \| YBR160W \| YPL209C \| YBL045C \| YCR065W \| YBL105C \| YER165W \| YJR059W \| YOL100W \| YPR119W \| YGL003C \| YNR047W \| YJL060W \| YMR001C \| YMR036C |
| complex_1059 | YBL016W \| YAL041W \| YBR200W \| YDL159W \| YLR362W \| YOR127W \| YBR031W \| YBR160W \| YNL298W \| YGR152C \| YLR229C \| YDR379W \| YHL007C \| YJL187C \| YKL129C |
| complex_1057 | YDL140C \| YGR116W \| YJR017C \| YML010W \| YDL195W \| YGL019W \| YIL035C \| YIL131C \| YDR356W \| YLR399C \| YOL004W \| YOR039W \| YJR014W \| YOL145C \| YOR061W |
| complex_1055 | YAL005C \| YDR477W \| YER081W \| YLL024C \| YLR310C \| YNL209W \| YOR027W \| YPL106C \| YPR119W \| YBL075C \| YDL229W \| YDR356W \| YEL030W \| YDL017W \| YER103W |
| complex_3229 | YDL101C \| YJL138C \| YJL164C \| YKL095W \| YDR212W \| YFL033C \| YHR135C \| YIL033C \| YJR104C \| YKL166C \| YNL093W \| YPL203W \| YLR347C \| YNL027W \| YPL031C |
| complex_3226 | YBR160W \| YGL048C \| YGR092W \| YDL007W \| YDR394W \| YFR010W \| YLR424W \| YGL137W \| YNL250W \| YIL007C \| YLL034C \| YKL145W \| YOR117W \| YOR259C \| YLR309C |
| complex_3225 | YBL045C \| YFL033C \| YCR008W \| YPL031C \| YDR432W \| YER165W \| YGR159C \| YPR119W \| YGL137W \| YHR135C \| YIL033C \| YPL203W \| YJL164C \| YKL166C |
| complex_3224 | YBR160W \| YER008C \| YFL009W \| YGL003C \| YGR092W \| YJL187C \| YDL155W \| YDL185W \| YMR304W \| YDR146C \| YLR131C \| YPR119W \| YOR117W \| YNL016W \| YNL161W |
| complex_3222 | YAL032C \| YLR117C \| YMR001C \| YMR213W \| YPL151C \| YDL175C \| YIL079C \| YDR432W \| YGR159C \| YER013W \| YKR024C \| YGL120C \| YHR082C \| YNL189W \| YOR204W |
| complex_3221 | YAL021C \| YDR477W \| YGR092W \| YKR036C \| YPL042C \| YBR066C \| YER028C \| YPR119W \| YBR160W \| YDR216W \| YNL027W \| YER129W \| YGL035C \| YMR037C |

| | |
|---|---|
| complex_2138 | YDL017W \| YLL039C \| YDR490C \| YFL033C \| YHR135C \| YHR205W \| YKL166C \| YGL137W \| YLR216C \| YIL033C \| YMR022W \| YJL164C \| YNL093W \| YDR457W \| YPL203W |
| complex_2137 | YBR109C \| YJR065C \| YKL129C \| YMR109W \| YCR088W \| YDL029W \| YHL007C \| YPL140C \| YHR018C \| YKL073W \| YNL007C \| YHR030C \| YHR102W \| YJL095W \| YNL161W |
| complex_2498 | YBR160W \| YBR200W \| YCR065W \| YGR092W \| YJL187C \| YKL129C \| YMR001C \| YNL102W \| YNL298W \| YPR119W \| YDR054C \| YIL046W \| YIL106W \| YJL060W \| YOR319W |
| complex_2497 | YBR160W \| YDR507C \| YGL003C \| YPR120C \| YDR212W \| YJL106W \| YKR036C \| YNL317W \| YOR212W \| YHL007C \| YPL022W \| YIL142W \| YJR064W \| YML095C \| YMR201C |
| complex_2134 | YBR109C \| YOR039W \| YOR061W \| YOR326W \| YDL195W \| YIL035C \| YGL019W \| YIL118W \| YIL131C \| YLR399C \| YML010W \| YOL004W \| YGL106W \| YJR014W \| YOL145C |
| complex_2496 | YBL105C \| YJL095W \| YBR160W \| YHR018C \| YPR119W \| YDR394W \| YDL029W \| YHR030C \| YJR065C \| YKL073W \| YPL140C \| YMR109W \| YKL129C \| YOR231W \| YOL100W |
| complex_2133 | YBL105C \| YBR160W \| YGL003C \| YJL187C \| YNL102W \| YCR065W \| YPR119W \| YER165W \| YPR120C \| YHL035C \| YJL060W \| YKL129C \| YMR001C \| YNL068C \| YNL298W |
| complex_2495 | YBL105C \| YBR160W \| YER165W \| YHR018C \| YJL095W \| YOL100W \| YOR231W \| YPR119W \| YCR065W \| YGL003C \| YER103W \| YKL025C \| YMR109W \| YKL129C \| YDL029W |
| complex_2493 | YAL005C \| YLR310C \| YOR027W \| YBL016W \| YDL159W \| YGR040W \| YLR362W \| YBL075C \| YBR155W \| YJR032W \| YPL240C \| YDR477W \| YLL024C \| YER028C \| YNL096C |
| complex_2130 | YAR019C \| YGR092W \| YBR160W \| YCR065W \| YGL003C \| YKL129C \| YLR131C \| YPR111W \| YPR120C \| YDL155W \| YDL185W \| YMR304W \| YIL106W \| YNL068C \| YPR119W |
| complex_2492 | YAL005C \| YLR310C \| YBL075C \| YJL128C \| YLR362W \| YNL064C \| YOR027W \| YHR018C \| YCR073C \| YLL024C \| YPL106C \| YPL240C \| YLR113W \| YLR248W \| YNR031C |
| complex_1049 | YBR055C \| YDR283C \| YLR153C \| YPR178W \| YDL087C \| YDR235W \| YHR086W \| YIL061C \| YKL173W \| YLR147C \| YML046W \| YPR182W \| YDL208W \| YMR001C \| YNL147W |
| complex_1047 | YBR160W \| YBR200W \| YCR065W \| YJL187C \| YNL298W \| YOR127W \| YPR119W \| YAL041W \| YGR152C \| YLR229C \| YLR362W \| YHL007C \| YKL129C \| YIL046W \| YDR054C |
| complex_1046 | YBR160W \| YBR200W \| YCR065W \| YJL187C \| YKL129C \| YMR001C \| YNL102W \| YNL298W \| YOR127W \| YPR119W \| YAL041W \| YLR229C \| YLR362W \| YHL007C \| YOR204W |
| complex_1044 | YBR081C \| YER164W \| YGR252W \| YMR223W \| YBR198C \| YDR145W \| YDR167W \| YDR216W \| YDR448W \| YDR477W \| YGL035C \| YER129W \| YIL035C \| YLL039C \| YMR022W |
| complex_1041 | YBR133C \| YHL007C \| YHR086W \| YJL187C \| YBR160W \| YMR001C \| YBR200W \| YLR362W \| YPR119W \| YCR065W \| YGR092W \| YIL106W \| YPR111W \| YKL129C \| YNL298W |
| complex_3219 | YBL016W \| YBR160W \| YBL045C \| YCR065W \| YBL105C \| YER165W \| YLR310C \| YOL100W \| YPR119W \| YDR507C \| YGR040W \| YMR139W \| YGR159C \| YKL129C \| YMR036C |
| complex_3218 | YBL045C \| YOR231W \| YPR191W \| YBL105C \| YDL029W \| YPL140C \| YDR394W \| YER165W \| YGL096W \| YHL036W \| YGL137W \| YHR030C \| YJL095W \| YPL151C \| YPR054W |
| complex_3216 | YAL005C \| YDR477W \| YGL158W \| YJL128C \| YLL024C \| YLR310C \| YLR362W \| YNL064C \| YOR027W \| YBL016W \| YBL075C \| YDL159W \| YHR018C \| YLR113W \| YPL240C |

| | |
|---|---|
| complex_168 | YAL005C \| YNL308C \| YBL075C \| YER107C \| YLR310C \| YNL007C \| YNL175C \| YBR155W \| YDL229W \| YDR477W \| YLL024C \| YER028C \| YPL106C \| YGL070C \| YNL209W |
| complex_3215 | YAL005C \| YER165W \| YJL164C \| YNL308C \| YPL106C \| YDL101C \| YKL166C \| YIL035C \| YIL033C \| YMR022W \| YMR226C \| YNL027W \| YNL209W \| YPL153C \| YPL203W |
| complex_3214 | YAL005C \| YBL075C \| YDR172W \| YDR477W \| YER129W \| YER165W \| YLR310C \| YNL209W \| YOR027W \| YPL106C \| YDL229W \| YHR015W \| YLL024C \| YPL240C \| YPR119W |
| complex_165 | YAL005C \| YBR085W \| YDR477W \| YER081W \| YER107C \| YER165W \| YLR310C \| YNL064C \| YNL209W \| YOR027W \| YPL106C \| YBL075C \| YLL024C \| YNL041C \| YPL042C |
| complex_3212 | YAL005C \| YBR155W \| YER081W \| YOR362C \| YBR274W \| YJR045C \| YKL073W \| YLL024C \| YDR283C \| YPR178W \| YEL030W \| YFL016C \| YOR222W \| YGL096W \| YOR319W |
| complex_3211 | YBL075C \| YLR362W \| YBR160W \| YBR200W \| YCR065W \| YHL007C \| YHR018C \| YJL187C \| YKL129C \| YNL298W \| YPR119W \| YBL016W \| YCL024W \| YDL159W \| YDR054C |
| complex_2129 | YAR019C \| YGR092W \| YBL075C \| YNL064C \| YPR111W \| YBR160W \| YCR065W \| YDL155W \| YKL129C \| YHR018C \| YIL106W \| YPR119W \| YKR036C \| YMR001C \| YPL042C |
| complex_2128 | YBL045C \| YKL139W \| YCL011C \| YDL084W \| YDR432W \| YNL112W \| YOR204W \| YPR119W \| YDL108W \| YFL029C \| YER165W \| YGL173C \| YGR159C \| YDR477W \| YPR054W |
| complex_2127 | YAL005C \| YBL075C \| YLR310C \| YNL064C \| YPL106C \| YBL016W \| YDL229W \| YDR356W \| YDR477W \| YDL192W \| YGL158W \| YER165W \| YLR113W \| YLL024C \| YNL209W |
| complex_2124 | YBR160W \| YDL155W \| YGL003C \| YGR092W \| YGR238C \| YHR158C \| YIL106W \| YLR096W \| YMR001C \| YNL068C \| YPR119W \| YPR120C \| YHL035C \| YJL187C \| YPR111W |
| complex_2486 | YAL024C \| YBR160W \| YBL045C \| YPR054W \| YPR191W \| YDL108W \| YDR432W \| YER165W \| YGL173C \| YKL139W \| YPR119W \| YFL029C \| YGL137W \| YHR030C \| YMR036C |
| complex_2121 | YAL005C \| YBL075C \| YBR155W \| YDR477W \| YER129W \| YJR032W \| YLR310C \| YOR027W \| YDR471W \| YDR194C \| YGL035C \| YPL240C \| YLL024C \| YPL106C \| YLR347C |
| complex_2120 | YDR075W \| YML010W \| YGL019W \| YGL254W \| YIL118W \| YIL131C \| YOL004W \| YOR039W \| YGR270W \| YIL035C \| YOR061W \| YJL074C \| YPL153C \| YOL145C |
| complex_2481 | YAL005C \| YJL187C \| YNL007C \| YNR047W \| YBL075C \| YBR160W \| YBR200W \| YCR065W \| YHL007C \| YHR018C \| YKL129C \| YNL298W \| YPR119W \| YCR091W \| YLR362W |
| complex_1039 | YBR160W \| YGR092W \| YHL007C \| YJL187C \| YMR001C \| YNL298W \| YCR002C \| YCL024W \| YDR507C \| YJR076C \| YLR229C \| YDL225W \| YHR107C \| YKL129C \| YLR314C |
| complex_2480 | YBR160W \| YER008C \| YJL187C \| YNL102W \| YCR065W \| YDL155W \| YGR092W \| YKL129C \| YPR119W \| YGR108W \| YHL035C \| YIL106W \| YMR001C \| YNL068C \| YNL298W |
| complex_1038 | YAL005C \| YDR356W \| YER081W \| YLL024C \| YLR310C \| YNL007C \| YBR160W \| YDL028C \| YPR141C \| YDL229W \| YBL063W \| YFL037W \| YLR212C \| YML085C \| YPL106C |
| complex_1035 | YBR160W \| YCL024W \| YGR092W \| YKL101W \| YLR314C \| YNL298W \| YOR204W \| YCR002C \| YDL225W \| YDR507C \| YHR107C \| YPR119W \| YMR001C \| YJL060W \| YJR076C |
| complex_1033 | YAL005C \| YBL075C \| YER012W \| YLL024C \| YLR310C \| YOR027W \| YER103W \| YGL011C \| YGR135W \| YJL001W \| YML092C \| YMR314W \| YOL038W \| YPR103W \| YPR054W |

| | |
|---|---|
| complex_1032 | YDL195W \| YGL019W \| YIL131C \| YOR061W \| YGL254W \| YIL118W \| YLR399C \| YML010W \| YML074C \| YOL004W \| YOR039W \| YGL171W \| YIL035C \| YOL145C \| YPL204W |
| complex_1394 | YBL045C \| YCR065W \| YDR477W \| YBR160W \| YDL229W \| YER129W \| YDR432W \| YER165W \| YGL173C \| YGR159C \| YKL139W \| YOR204W \| YPR119W \| YFL029C \| YPR054W |
| complex_1393 | YBL045C \| YGR159C \| YJL187C \| YBL105C \| YOL100W \| YBR160W \| YGL003C \| YGR092W \| YJL060W \| YKR095W \| YMR001C \| YPR119W \| YDR432W \| YER165W \| YOR204W |
| complex_1030 | YAL005C \| YBL075C \| YER165W \| YGL254W \| YLR310C \| YOR027W \| YOR254C \| YBR160W \| YOR061W \| YPR119W \| YDR146C \| YER103W \| YJL034W \| YKL073W \| YLL024C |
| complex_1392 | YAL005C \| YNL308C \| YBR017C \| YLR293C \| YOR185C \| YBR155W \| YCR038C \| YDL229W \| YDR356W \| YDR477W \| YGL070C \| YPL106C \| YNL209W \| YEL030W \| YIL095W |
| complex_3209 | YDR394W \| YDL007W \| YGL048C \| YKL145W \| YOR117W \| YOR259C \| YFR010W \| YMR216C \| YMR314W \| YGL011C \| YCR057C \| YOL038W \| YLL011W \| YML092C \| YNL250W |
| complex_3208 | YBR160W \| YJL141C \| YJL187C \| YMR001C \| YNL298W \| YDR054C \| YFL009W \| YGL096W \| YOR319W \| YHL002W \| YHR108W \| YIL046W \| YOL133W \| YPR184W |
| complex_1390 | YBL105C \| YBR160W \| YJL095W \| YOL100W \| YPL151C \| YPR119W \| YPR120C \| YDL155W \| YER008C \| YGL003C \| YIL106W \| YMR001C \| YMR304W \| YGR092W \| YJL187C |
| complex_3207 | YDL134C \| YDR523C \| YHR135C \| YPR040W \| YDL188C \| YGR253C \| YML092C \| YMR314W \| YOR362C \| YPR103W \| YGL137W \| YIL033C \| YKL166C \| YJL164C \| YPL203W |
| complex_3206 | YAR019C \| YGL003C \| YGR092W \| YBR160W \| YDL028C \| YMR001C \| YDL108W \| YFL029C \| YDL155W \| YDR146C \| YPR119W \| YER165W \| YIL106W \| YJL060W \| YPR111W |
| complex_3205 | YAL005C \| YBL075C \| YLR310C \| YPL106C \| YBL016W \| YBL047C \| YBR109C \| YBR094W \| YOR061W \| YDR477W \| YDL192W \| YEL034W \| YER165W \| YPL204W \| YLL024C |
| complex_3204 | YBR160W \| YCR065W \| YDL155W \| YGR092W \| YHL007C \| YJL187C \| YNL298W \| YPR119W \| YDR054C \| YLR362W \| YIL106W \| YNL161W \| YKL129C \| YDL029W \| YMR109W |
| complex_3203 | YAL005C \| YBR160W \| YIL035C \| YIL131C \| YNL112W \| YDL195W \| YGL019W \| YIL118W \| YLR399C \| YOL004W \| YOR039W \| YHR018C \| YMR308C \| YOL145C \| YOR061W |
| complex_3200 | YDL134C \| YDR523C \| YIL045W \| YDL188C \| YDL029W \| YGL011C \| YOL038W \| YGR135W \| YOR261C \| YGR253C \| YJR017C \| YOR362C \| YLL041C \| YML092C \| YMR314W |
| complex_2116 | YAL005C \| YBL016W \| YBR160W \| YLR310C \| YNL064C \| YLR362W \| YBL075C \| YCR065W \| YKL129C \| YDL159W \| YHR018C \| YGR040W \| YLL024C \| YNL298W \| YPR119W |
| complex_2477 | YAL029C \| YBR109C \| YFL005W \| YFL039C \| YGL106W \| YMR139W \| YOR035C \| YJR065C \| YOR326W \| YDL029W \| YHR023W \| YJL095W \| YKL129C \| YMR109W \| YPL140C |
| complex_2476 | YAR019C \| YGR092W \| YHR018C \| YIL106W \| YBR160W \| YDL029W \| YLR131C \| YCR065W \| YDL124W \| YDL155W \| YKL129C \| YPR119W \| YHL007C \| YNL161W \| YPR111W |
| complex_2474 | YAL005C \| YER081W \| YLL016W \| YLR310C \| YPL106C \| YBL016W \| YDL159W \| YGL158W \| YLR362W \| YBL075C \| YDR343C \| YGR040W \| YHL007C \| YMR139W \| YLL024C |
| complex_2471 | YBR160W \| YGL003C \| YGL137W \| YGR092W \| YIL046W \| YMR001C \| YPL115C \| YPR119W \| YPR120C \| YDR212W \| YIL142W \| YKR036C \| YGR040W \| YJL187C \| YNL298W |

| | |
|---|---|
| complex_1029 | YAL005C \| YDR477W \| YER165W \| YLR310C \| YPL106C \| YBL075C \| YDL159W \| YBL016W \| YDR146C \| YLL024C \| YGR040W \| YLR362W \| YHL007C \| YJR032W |
| complex_1028 | YAL005C \| YDR477W \| YLR310C \| YNL064C \| YNL209W \| YPL106C \| YBL075C \| YPR119W \| YDR146C \| YLL024C \| YER129W \| YDR507C \| YBR260C \| YHR018C \| YMR139W |
| complex_1026 | YAL005C \| YBL016W \| YGR040W \| YLR310C \| YLR362W \| YBL075C \| YBR160W \| YHL007C \| YNL298W \| YBR200W \| YPR119W \| YDL159W \| YER165W \| YJL187C \| YKL129C |
| complex_1385 | YAL005C \| YBL016W \| YBL075C \| YBR155W \| YLR310C \| YNL064C \| YOR027W \| YPL141C \| YDL159W \| YGR040W \| YHR018C \| YLR362W \| YJR032W \| YPL240C \| YLL024C |
| complex_1021 | YAL005C \| YBR155W \| YDR477W \| YER081W \| YFL016C \| YNL209W \| YOR222W \| YPR119W \| YBR274W \| YEL030W \| YJR045C \| YER129W \| YGL035C \| YHR018C \| YLL024C |
| complex_2109 | YBL016W \| YHR018C \| YLR362W \| YBL075C \| YBR160W \| YHL007C \| YJL128C \| YCR065W \| YBR200W \| YPR119W \| YDL159W \| YJL187C \| YKL129C \| YNL298W \| YLR229C |
| complex_2105 | YCR002C \| YCL024W \| YDR507C \| YLR229C \| YLR314C \| YNL298W \| YDL225W \| YHR107C \| YGL127C \| YGR092W \| YKL101W \| YMR001C \| YJR076C \| YPR080W \| YPR119W |
| complex_2104 | YCR084C \| YOL004W \| YDL195W \| YGL019W \| YIL035C \| YIL131C \| YIL118W \| YML010W \| YML074C \| YOR039W \| YJL081C \| YOR244W \| YNL189W \| YOL145C \| YOR061W |
| complex_2466 | YBL045C \| YCR065W \| YOR231W \| YBL105C \| YJL095W \| YBR160W \| YKL129C \| YPL140C \| YER165W \| YPR119W \| YHR030C \| YDL029W \| YLR309C \| YMR109W \| YOL100W |
| complex_2103 | YBL045C \| YBR160W \| YCR065W \| YBL105C \| YCR008W \| YDL025C \| YDR432W \| YER165W \| YGL173C \| YGR159C \| YOR204W \| YPR119W \| YKL129C \| YOL100W \| YPL031C |
| complex_2465 | YBR160W \| YCR065W \| YER008C \| YGL003C \| YKL129C \| YMR001C \| YNL102W \| YNL298W \| YPL209C \| YLR362W \| YMR304W \| YGR040W \| YHL007C \| YJL187C \| YPR119W |
| complex_2463 | YBR160W \| YBR200W \| YGL003C \| YGR092W \| YJR059W \| YKL129C \| YMR001C \| YNL068C \| YNL102W \| YNL298W \| YPR119W \| YPR120C \| YDL145C \| YJL187C \| YIL046W |
| complex_2100 | YBR031W \| YBR160W \| YOR127W \| YCR065W \| YJL187C \| YOR204W \| YGR152C \| YAL041W \| YHL007C \| YLR229C \| YDL029W \| YKL129C \| YMR109W \| YPR119W \| YNL298W |
| complex_2461 | YBL045C \| YPL140C \| YPR054W \| YPR191W \| YBR160W \| YGR092W \| YKL129C \| YDL029W \| YDL047W \| YDL145C \| YGL137W \| YHR030C \| YDR394W \| YMR109W \| YJL095W |
| complex_2460 | YDR188W \| YDR142C \| YDR212W \| YGL137W \| YNL317W \| YOR212W \| YGL116W \| YGR092W \| YJL106W \| YJL164C \| YKL166C \| YIL142W \| YJL014W \| YJR064W \| YPL203W |
| complex_1017 | YAL005C \| YBL075C \| YDR477W \| YER165W \| YLL024C \| YLR310C \| YNL007C \| YOR027W \| YPL106C \| YER103W \| YHR030C \| YKL073W \| YPR054W \| YJL034W \| YNL027W |
| complex_1016 | YAL005C \| YLR310C \| YNL007C \| YNL175C \| YOR027W \| YPL106C \| YBL075C \| YLR362W \| YHL007C \| YNL161W \| YHR030C \| YPR191W \| YHR102W \| YLL024C \| YPR054W |
| complex_1378 | YBR160W \| YJL187C \| YNL102W \| YBR200W \| YPR119W \| YCR065W \| YDL155W \| YGR092W \| YAR019C \| YHR018C \| YIL106W \| YPR111W \| YKL129C \| YMR001C \| YNL298W |
| complex_1376 | YDR364C \| YKL095W \| YMR213W \| YDR473C \| YKL173W \| YPR178W \| YER013W \| YLR117C \| YMR001C \| YFL017W-A \| YGL120C \| YPL151C \| YIR005W \| YMR240C \| YNL189W |

| | |
|---|---|
| complex_1011 | YBR160W \| YDR507C \| YHR018C \| YJL187C \| YKL129C \| YLR314C \| YMR001C \| YNL298W \| YPR119W \| YCR002C \| YCL024W \| YHR107C \| YKL101W \| YJR076C \| YCR088W |
| complex_1373 | YBR109C \| YGL019W \| YGL106W \| YGR270W \| YOR039W \| YOR061W \| YBR160W \| YIL118W \| YIL131C \| YML010W \| YML074C \| YNL027W \| YIL035C \| YGL254W \| YOL145C |
| complex_1010 | YBR160W \| YGR092W \| YJL187C \| YMR001C \| YNL189W \| YNL298W \| YCL024W \| YCR002C \| YDL225W \| YDR507C \| YHR107C \| YLR314C \| YKL101W \| YJR076C \| YMR304W |
| complex_1372 | YAL005C \| YBL075C \| YBR155W \| YJR032W \| YJR059W \| YLR310C \| YOR027W \| YDL229W \| YDR477W \| YNL209W \| YOR204W \| YLL024C \| YPL106C \| YPL240C \| YLR347C |
| complex_1370 | YBL045C \| YCR065W \| YNR047W \| YBR160W \| YDL017W \| YDL155W \| YGL003C \| YGR092W \| YIL106W \| YJL187C \| YMR001C \| YPR111W \| YPR119W \| YGL137W \| YHR086W |
| complex_497 | YCR034W \| YHR030C \| YPL140C \| YDL007W \| YFR004W \| YDL097C \| YDR394W \| YGL048C \| YGR262C \| YKL145W \| YOR117W \| YOR259C \| YOR261C \| YDL147W \| YDL029W |
| complex_493 | YDL007W \| YFR004W \| YDL097C \| YDR394W \| YGL048C \| YGR262C \| YHR030C \| YKL145W \| YOR117W \| YOR259C \| YOR261C \| YDL147W \| YDL029W \| YGR092W \| YNL250W |
| complex_491 | YAL005C \| YBL075C \| YDR477W \| YIR005W \| YJR032W \| YLL024C \| YLR310C \| YNL064C \| YNL209W \| YOR027W \| YPL106C \| YPR128C \| YHR064C \| YER028C \| YPL042C |
| complex_2458 | YAL005C \| YLL016W \| YLR310C \| YNL064C \| YOR027W \| YPL106C \| YBL016W \| YBL075C \| YDR477W \| YJL005W \| YNL098C \| YLL024C \| YMR139W \| YOR101W \| YNL209W |
| complex_2457 | YBL026W \| YER112W \| YER146W \| YBR274W \| YDL145C \| YER028C \| YGL137W \| YJL124C \| YDR477W \| YGR092W \| YNL178W \| YPL042C \| YPR182W \| YJR022W \| YNL147W |
| complex_2456 | YAL024C \| YBR160W \| YDR507C \| YBL045C \| YCR065W \| YBL105C \| YOL100W \| YGL003C \| YER165W \| YMR139W \| YPR119W \| YJL060W \| YJR059W \| YMR001C \| YMR036C |
| complex_2455 | YAL024C \| YBR160W \| YBL105C \| YER165W \| YOL100W \| YPR119W \| YCR065W \| YKL116C \| YCR091W \| YNR047W \| YDR432W \| YOL123W \| YPR191W \| YJR059W \| YMR036C |
| complex_2454 | YBL016W \| YBR200W \| YDL145C \| YDL159W \| YLR362W \| YOR212W \| YBR160W \| YHL007C \| YJL187C \| YNL298W \| YOR362C \| YAL041W \| YMR304W \| YLR229C \| YMR001C |
| complex_2453 | YAL005C \| YBR155W \| YDL229W \| YER081W \| YHR030C \| YNL007C \| YPL106C \| YBR059C \| YIL095W \| YNL161W \| YEL030W \| YHR102W \| YLR131C \| YFL034C-B \| YHL007C |
| complex_2452 | YBR160W \| YAL041W \| YBR200W \| YDL240W \| YDR379W \| YHL007C \| YJL187C \| YKL129C \| YNL298W \| YOR127W \| YPR119W \| YGR152C \| YLR229C \| YOL113W \| YPR165W |
| complex_1009 | YAL005C \| YCL011C \| YDL229W \| YLR310C \| YNL007C \| YPR128C \| YKL139W \| YPL106C \| YDR432W \| YNL112W \| YDR477W \| YER028C \| YOL123W \| YFL018C |
| complex_1008 | YAL005C \| YDR477W \| YJR032W \| YLL024C \| YLR310C \| YLR362W \| YOR027W \| YBL075C \| YBR155W \| YPL240C \| YDL229W \| YPL042C \| YLR347C \| YNL209W \| YPL106C |
| complex_1007 | YBR160W \| YDR507C \| YMR001C \| YCL024W \| YCR002C \| YHR107C \| YJR076C \| YJR105W \| YNL298W \| YDL225W \| YKL101W \| YJL187C \| YDR218C \| YKL129C \| YLR314C |
| complex_1005 | YBR160W \| YBR200W \| YCR065W \| YJL187C \| YNL298W \| YOR127W \| YPR119W \| YAL041W \| YGR152C \| YLR229C \| YLR362W \| YHL007C \| YKL129C \| YJL095W \| YMR109W |

| | |
|---|---|
| complex_1004 | YBL026W \| YER112W \| YER146W \| YJL124C \| YGL011C \| YFR052W \| YMR314W \| YOL038W \| YGR135W \| YJR022W \| YLR147C \| YPR182W \| YML092C \| YOR362C \| YNL147W |
| complex_1003 | YAL005C \| YER165W \| YLR310C \| YNL007C \| YOR185C \| YPR128C \| YDL229W \| YDR477W \| YPL106C \| YNL209W \| YER028C \| YLR131C \| YNL161W \| YOL128C \| YLR293C |
| complex_1365 | YBR160W \| YDL155W \| YER008C \| YGL003C \| YIL106W \| YMR001C \| YPL209C \| YPR111W \| YPR119W \| YPR120C \| YMR304W \| YGR092W \| YJL187C \| YNL298W \| YNL161W |
| complex_1002 | YAL005C \| YBL075C \| YER165W \| YLR310C \| YPL106C \| YPR119W \| YDL229W \| YDR172W \| YDR477W \| YJL164C \| YDR146C \| YNL027W \| YGL254W \| YLL024C \| YNL209W |
| complex_1363 | YAL005C \| YLR310C \| YBL016W \| YDL159W \| YHR018C \| YLR362W \| YBL075C \| YHL007C \| YNL064C \| YOR027W \| YGR040W \| YPL240C \| YLL024C \| YPL106C \| YNL161W |
| complex_1000 | YAL005C \| YDL229W \| YER081W \| YER165W \| YLR310C \| YPL106C \| YCR008W \| YIL033C \| YFL033C \| YJL164C \| YPL203W \| YKL166C \| YNL027W \| YNL209W \| YPL031C |
| complex_1362 | YBR200W \| YAL041W \| YBR160W \| YLR229C \| YLR362W \| YNL298W \| YPR119W \| YCR065W \| YDR054C \| YHL007C \| YKL129C \| YOR127W \| YJL187C \| YMR109W \| YOR204W |
| complex_1361 | YBL016W \| YCL024W \| YBR160W \| YNL298W \| YOR127W \| YBR200W \| YAL041W \| YLR229C \| YLR362W \| YCR002C \| YDL159W \| YHL007C \| YKL129C \| YJL187C \| YLR314C |
| complex_487 | YAL021C \| YDR477W \| YGR092W \| YKR036C \| YPL042C \| YDL007W \| YFR004W \| YDL097C \| YDR394W \| YGL048C \| YKL145W \| YOR117W \| YOR259C \| YOR261C \| YDL147W |
| complex_486 | YBR160W \| YGL048C \| YGR092W \| YHR027C \| YJR059W \| YKL145W \| YDL097C \| YDL007W \| YDR394W \| YOR117W \| YOR259C \| YDL147W \| YFR010W \| YGL137W \| YNL250W |
| complex_482 | YAL005C \| YDL229W \| YER081W \| YER165W \| YLL024C \| YLR310C \| YNL007C \| YPL106C \| YPR119W \| YPR128C \| YBL105C \| YOL100W \| YDR146C \| YNL209W \| YNL041C |
| complex_481 | YAL032C \| YBL105C \| YDR364C \| YKL173W \| YLL036C \| YLR117C \| YLR424W \| YMR001C \| YMR213W \| YPL151C \| YDL030W \| YER013W \| YGL120C \| YJL187C \| YMR240C |
| complex_2449 | YAR019C \| YKR036C \| YBL075C \| YML092C \| YPL042C \| YPR111W \| YBR160W \| YCR065W \| YDL155W \| YKL129C \| YGR092W \| YPR119W \| YHR018C \| YIL106W \| YMR001C |
| complex_2448 | YAR019C \| YGR092W \| YBR160W \| YJL187C \| YKL129C \| YNL102W \| YPR111W \| YPR119W \| YDL155W \| YHR018C \| YPR104C \| YGL137W \| YHL035C \| YIL106W \| YMR001C |
| complex_2447 | YBR094W \| YOR061W \| YPL240C \| YBR109C \| YGL019W \| YOR039W \| YGL254W \| YIL035C \| YIL131C \| YML010W \| YML074C \| YGL035C \| YOL145C |
| complex_2445 | YAL041W \| YHL007C \| YOR212W \| YBR160W \| YER008C \| YGL003C \| YIL046W \| YNL298W \| YPL115C \| YPR120C \| YDR212W \| YIL142W \| YMR304W \| YJL187C \| YMR001C |
| complex_2444 | YAL005C \| YLL016W \| YLR310C \| YNL064C \| YOR027W \| YPL106C \| YBL016W \| YLR362W \| YBL075C \| YPL240C \| YJL005W \| YNL098C \| YLL024C \| YMR139W \| YOR101W |
| complex_2442 | YBL105C \| YJL095W \| YOR231W \| YBR109C \| YJR065C \| YMR109W \| YCR088W \| YDL029W \| YHR018C \| YKL073W \| YNL007C \| YHR030C \| YBR260C \| YKL129C \| YPL140C |
| complex_2440 | YBR160W \| YLR210W \| YNL317W \| YCR091W \| YKR036C \| YLL011W \| YOR212W \| YDR188W \| YBR198C \| YDR212W \| YJL106W \| YIL142W \| YJR064W \| YJR059W \| YNR047W |

| | |
|---|---|
| complex_1356 | YBR160W \| YGR092W \| YJL187C \| YKL048C \| YLR314C \| YMR001C \| YNL189W \| YNL298W \| YCL024W \| YCR002C \| YDR507C \| YLR229C \| YDL225W \| YHR107C \| YJR076C |
| complex_1355 | YBL075C \| YDL159W \| YLR310C \| YLR362W \| YBR160W \| YBR200W \| YCR065W \| YHL007C \| YHR018C \| YJL187C \| YKL129C \| YNL298W \| YPR119W \| YBL016W \| YGR040W |
| complex_1353 | YBL045C \| YCR065W \| YGR159C \| YMR139W \| YBL105C \| YBR160W \| YER165W \| YOL100W \| YPR119W \| YDR507C \| YGL003C \| YDR432W \| YOR204W \| YJL060W \| YKR095W |
| complex_1350 | YAL005C \| YBL075C \| YLR310C \| YNL064C \| YPR119W \| YBL016W \| YLR362W \| YBR200W \| YHL007C \| YDL159W \| YGR040W \| YMR290C \| YHR018C \| YKL129C \| YLR347C |
| complex_119 | YDL007W \| YFR004W \| YDL097C \| YDR394W \| YGL048C \| YGR040W \| YGR262C \| YHR027C \| YKL145W \| YOR117W \| YOR259C \| YOR261C \| YDL147W \| YDL029W \| YIL142W |
| complex_479 | YAL005C \| YER081W \| YER165W \| YLL024C \| YLR310C \| YNL007C \| YNL209W \| YPR128C \| YDL229W \| YDR356W \| YPR161C \| YDR477W \| YER028C \| YPL106C \| YNL041C |
| complex_478 | YBR160W \| YGR092W \| YDL007W \| YFR004W \| YDL097C \| YDR394W \| YGL048C \| YGR262C \| YKL145W \| YOR117W \| YOR259C \| YOR261C \| YDL147W \| YDL029W \| YDR356W |
| complex_115 | YAL005C \| YDR477W \| YLL024C \| YLR310C \| YNL064C \| YNL175C \| YNL209W \| YNL308C \| YOR027W \| YPL106C \| YPR128C \| YBL075C \| YGL070C \| YER028C \| YER103W |
| complex_112 | YAL032C \| YKL173W \| YLL036C \| YLR117C \| YLR424W \| YMR001C \| YMR213W \| YPL151C \| YBR065C \| YDL030W \| YER013W \| YGL120C \| YHR086W \| YLR147C \| YMR240C |
| complex_472 | YAL005C \| YAR018C \| YBL075C \| YDL229W \| YER081W \| YIR005W \| YLR310C \| YNL007C \| YNL209W \| YNL308C \| YOR027W \| YPL106C \| YPR128C \| YHR064C \| YLL024C |
| complex_471 | YAL005C \| YBL075C \| YBR085W \| YDL229W \| YER165W \| YLR310C \| YNL064C \| YOR027W \| YOR388C \| YPL106C \| YBR160W \| YDL159W \| YDR146C \| YLL024C \| YNL041C |
| complex_2438 | YAL024C \| YKL048C \| YBL045C \| YCR065W \| YBL105C \| YBR160W \| YDL025C \| YLL019C \| YMR036C \| YOL100W \| YPR119W \| YDR432W \| YER165W \| YGR159C \| YKL025C |
| complex_2436 | YAR019C \| YGR092W \| YBL045C \| YBR160W \| YCR065W \| YJL187C \| YDL017W \| YDL155W \| YKL129C \| YER165W \| YPR119W \| YGL137W \| YIL106W \| YPR111W \| YMR001C |
| complex_2435 | YCR057C \| YGL011C \| YGR040W \| YGR135W \| YLR129W \| YLR409C \| YOL038W \| YDL159W \| YDL188C \| YML092C \| YMR314W \| YOR362C \| YPR034W \| YLR222C \| YLR362W |
| complex_2795 | YDL007W \| YEL037C \| YER122C \| YGL048C \| YKL145W \| YLR424W \| YDL047W \| YDL029W \| YHR030C \| YDR394W \| YGL137W \| YGR092W \| YOR117W \| YOR259C \| YIL007C |
| complex_2431 | YDR432W \| YER165W \| YIR001C \| YKR095W \| YNL016W \| YPR119W \| YER133W \| YJL033W \| YJR093C \| YKL059C \| YKR002W \| YPR107C \| YGL049C \| YGR162W \| YNL189W |
| complex_2793 | YAR019C \| YDL108W \| YGR092W \| YBR160W \| YBR135W \| YCR065W \| YDR146C \| YGL003C \| YKL129C \| YPR120C \| YDL155W \| YIL106W \| YNL068C \| YPR119W \| YKL025C |
| complex_2430 | YBL045C \| YCR065W \| YPR054W \| YPR191W \| YBR160W \| YNL102W \| YLR096W \| YDL108W \| YFL029C \| YLR113W \| YER165W \| YPR119W \| YGL137W \| YHR030C \| YKL129C |
| complex_2790 | YBR055C \| YDR283C \| YLR153C \| YPR178W \| YDL087C \| YKL173W \| YDL208W \| YDR235W \| YDR473C \| YHR086W \| YML046W \| YIL061C \| YLR147C \| YMR001C \| YNL147W |

| | |
|---|---|
| complex_1346 | YAL005C \| YLR310C \| YNL308C \| YAR018C \| YBL075C \| YPL106C \| YER081W \| YNL175C \| YOR027W \| YER103W \| YHR064C \| YLL024C \| YPR054W \| YNL209W |
| complex_1345 | YAL005C \| YBL075C \| YLR310C \| YNL064C \| YAR019C \| YGR092W \| YHR018C \| YIL106W \| YLR362W \| YPL042C \| YBR160W \| YKL129C \| YDL155W \| YHL007C \| YNL161W |
| complex_1344 | YAL005C \| YGL011C \| YGR135W \| YML092C \| YMR314W \| YOL038W \| YOR362C \| YCR057C \| YLR129W \| YLR222C \| YLR409C \| YDL188C \| YDR523C \| YDR283C \| YMR308C |
| complex_1342 | YAR019C \| YGR092W \| YBR160W \| YKL129C \| YDL007W \| YDR394W \| YEL037C \| YDL155W \| YDL029W \| YGL048C \| YOR117W \| YIL106W \| YNL161W \| YKL145W \| YOR259C |
| complex_106 | YAL005C \| YDL229W \| YER081W \| YER107C \| YER165W \| YLL024C \| YLR310C \| YNL007C \| YNL209W \| YOR027W \| YPR128C \| YDR477W \| YER028C \| YPL106C \| YNL041C |
| complex_105 | YAL005C \| YBL075C \| YDL229W \| YER081W \| YER165W \| YLL024C \| YLR310C \| YNL064C \| YOR027W \| YPR128C \| YDR477W \| YNL209W \| YER028C \| YPL106C \| YNL041C |
| complex_466 | YAL032C \| YKL173W \| YLL036C \| YLR117C \| YLR424W \| YMR001C \| YMR213W \| YPL151C \| YDL030W \| YER013W \| YGL120C \| YGR136W \| YOR319W \| YIR005W \| YMR240C |
| complex_465 | YAL005C \| YBR155W \| YER081W \| YFL016C \| YIL061C \| YLL024C \| YLR310C \| YOR222W \| YDR477W \| YJR045C \| YLR369W \| YEL030W \| YOR232W \| YFL037W \| YNL027W |
| complex_462 | YAL005C \| YDL229W \| YER081W \| YER165W \| YLL024C \| YLR310C \| YNL007C \| YPL106C \| YPR128C \| YDR477W \| YHR030C \| YNL209W \| YER028C \| YLR216C \| YNL041C |
| complex_2429 | YAL005C \| YBR155W \| YER081W \| YNL209W \| YPR119W \| YBR160W \| YLR131C \| YNL161W \| YDL155W \| YDR146C \| YEL030W \| YFL034C-B \| YHR102W \| YGR092W \| YIL106W |
| complex_2788 | YBR034C \| YGR159C \| YIL079C \| YOL123W \| YDL175C \| YOR204W \| YPR191W \| YDR432W \| YER165W \| YIR001C \| YNL016W \| YPR119W \| YPR104C \| YGL049C \| YGR162W |
| complex_2425 | YBL045C \| YJL095W \| YPR054W \| YPR191W \| YBL105C \| YOR231W \| YER165W \| YGL022W \| YPR119W \| YHR030C \| YDL029W \| YPL140C \| YHR082C \| YKL129C \| YPL151C |
| complex_2787 | YBR160W \| YDR285W \| YHL007C \| YJL187C \| YKR095W \| YMR001C \| YOR127W \| YPR119W \| YOR204W \| YDR379W \| YGR152C \| YLR229C \| YAL041W \| YKL129C \| YNL298W |
| complex_2786 | YBR160W \| YER008C \| YGL003C \| YJL187C \| YMR001C \| YNL068C \| YPL209C \| YPR119W \| YPR120C \| YMR304W \| YIL046W \| YKL101W \| YKR048C \| YDR364C \| YNL298W |
| complex_2423 | YAL005C \| YBL075C \| YDR477W \| YLR310C \| YNL209W \| YOR027W \| YPL106C \| YNL308C \| YDL229W \| YDR356W \| YGL070C \| YLL024C \| YPL042C \| YNL064C |
| complex_2422 | YBL105C \| YBR160W \| YER008C \| YJL095W \| YOL100W \| YOR231W \| YJL187C \| YBR200W \| YLR229C \| YNL298W \| YPR119W \| YCR065W \| YGR108W \| YHL007C \| YKL129C |
| complex_2421 | YAL005C \| YCR008W \| YLR310C \| YNL209W \| YOR027W \| YPL031C \| YPL106C \| YBL075C \| YDL229W \| YDR477W \| YHR018C \| YLL024C \| YFL033C \| YPL240C \| YPR119W |
| complex_2783 | YBR160W \| YGL003C \| YLR401C \| YCR065W \| YDL028C \| YDR356W \| YDL155W \| YPR119W \| YGR092W \| YIL106W \| YNL068C \| YPR111W \| YKL129C \| YPR120C \| YPR141C |
| complex_2782 | YBR160W \| YER008C \| YIL046W \| YJL187C \| YKL129C \| YNL298W \| YOR127W \| YBR200W \| YAL041W \| YLR229C \| YPR119W \| YHL007C \| YHR098C \| YJR048W \| YNL007C |

| | |
|---|---|
| complex_1339 | YAL021C \| YAR019C \| YGR092W \| YKR036C \| YBR160W \| YGL048C \| YGL137W \| YIL106W \| YKL145W \| YDL007W \| YEL037C \| YDL147W \| YDR394W \| YOR117W \| YOR259C |
| complex_2780 | YBL075C \| YLR362W \| YNL298W \| YBR160W \| YBR200W \| YCR065W \| YGR108W \| YHL007C \| YHR018C \| YJL187C \| YKL129C \| YNR047W \| YPR119W \| YCR091W \| YPL119C |
| complex_1337 | YBL032W \| YER165W \| YLR175W \| YDR432W \| YGR159C \| YIR001C \| YOL123W \| YOR204W \| YPR119W \| YGL044C \| YGL049C \| YNL016W \| YCR057C \| YGR162W \| YJL033W |
| complex_1699 | YAL005C \| YLR310C \| YBL016W \| YLR362W \| YBL075C \| YHL007C \| YNL064C \| YOR027W \| YDL159W \| YFL034C-B \| YNL161W \| YGR040W \| YHR018C \| YPL240C \| YLL024C |
| complex_1336 | YBR160W \| YDR507C \| YJL187C \| YMR001C \| YNL298W \| YPL115C \| YCR002C \| YCL024W \| YHR107C \| YLR229C \| YDR212W \| YJL164C \| YKL101W \| YJR076C \| YLR314C |
| complex_1335 | YAL005C \| YLR310C \| YNL007C \| YNL175C \| YOR027W \| YPL106C \| YAR019C \| YGR092W \| YBL075C \| YBR160W \| YDR146C \| YDR507C \| YLL024C \| YBL016W \| YMR139W |
| complex_1697 | YDL029W \| YDR394W \| YOR259C \| YDL047W \| YDL147W \| YDL007W \| YDR523C \| YGL048C \| YJL008C \| YKL145W \| YOR117W \| YOR261C \| YKL139W \| YLL034C \| YLR309C |
| complex_1334 | YBR160W \| YER008C \| YGL003C \| YIL046W \| YJL187C \| YNL298W \| YPR120C \| YMR304W \| YHL007C \| YAL041W \| YLR229C \| YLR362W \| YMR001C \| YNL007C \| YNL161W |
| complex_1696 | YBL045C \| YPR054W \| YPR191W \| YDL017W \| YDL029W \| YPL140C \| YDR394W \| YER165W \| YKL173W \| YGL137W \| YHR030C \| YLR216C \| YJL095W \| YOR027W \| YPR178W |
| complex_1695 | YBL105C \| YOL100W \| YPL151C \| YBR065C \| YMR001C \| YPR119W \| YBR160W \| YER008C \| YOL123W \| YDR432W \| YGR159C \| YOR204W \| YER165W \| YGL044C \| YKL025C |
| complex_1332 | YBL016W \| YIL169C \| YBR160W \| YJL187C \| YNL298W \| YOR127W \| YBR200W \| YAL041W \| YLR229C \| YLR362W \| YPR119W \| YDL159W \| YHL007C \| YKL129C \| YIL106W |
| complex_1694 | YAL005C \| YBL075C \| YLL024C \| YLR310C \| YNL064C \| YNL209W \| YBL016W \| YLR362W \| YDL159W \| YGR040W \| YDL229W \| YDR356W \| YDR477W \| YMR139W \| YPL106C |
| complex_1693 | YDL208W \| YKL173W \| YPR178W \| YDR283C \| YDR473C \| YLR424W \| YFL017W-A \| YLR117C \| YLR147C \| YMR001C \| YPL151C \| YGL120C \| YHR086W \| YLR086W \| YNL147W |
| complex_459 | YAL005C \| YDL229W \| YER081W \| YER165W \| YLL024C \| YLR310C \| YNL007C \| YPL106C \| YPR128C \| YDR477W \| YGL158W \| YER028C \| YNL209W \| YLR113W \| YNL041C |
| complex_2419 | YBL045C \| YPL203W \| YDR490C \| YER165W \| YKL166C \| YGL137W \| YLR113W \| YLR216C \| YNR032W \| YHR135C \| YIL033C \| YMR022W \| YJL164C \| YJL128C \| YNR031C |
| complex_2417 | YAL005C \| YCL011C \| YDL229W \| YER165W \| YDL084W \| YKL139W \| YDR432W \| YNL112W \| YER133W \| YJL033W \| YJR093C \| YKL059C \| YPR107C \| YNL004W \| YPL106C |
| complex_2779 | YDR394W \| YDL007W \| YDL029W \| YGL048C \| YKL145W \| YNL250W \| YOR117W \| YOR259C \| YFR010W \| YMR216C \| YOR261C \| YGL137W \| YLR309C \| YNL025C \| YNL290W |
| complex_2778 | YAL005C \| YLR362W \| YNL064C \| YOR027W \| YPR111W \| YBL075C \| YBR160W \| YCR065W \| YIL106W \| YJL187C \| YKL129C \| YNL298W \| YPR119W \| YHR018C \| YPL240C |
| complex_2414 | YBR094W \| YBR109C \| YOR061W \| YDL195W \| YGL019W \| YIL035C \| YIL131C \| YIL118W \| YLR399C \| YOL004W \| YOR039W \| YOL145C \| YOR254C \| YPL131W \| YOR326W |

| | |
|---|---|
| complex_2776 | YBR109C \| YGL019W \| YOR039W \| YOR061W \| YBR160W \| YDL108W \| YFL029C \| YIL131C \| YGL254W \| YIL035C \| YIL118W \| YML010W \| YML074C \| YOR267C \| YOL145C |
| complex_2413 | YBR034C \| YGR159C \| YIL079C \| YOL123W \| YCL011C \| YDL084W \| YDL226C \| YDR432W \| YER165W \| YFL039C \| YKL139W \| YNL112W \| YDL192W \| YOR204W \| YNL004W |
| complex_2411 | YDL007W \| YEL037C \| YLR424W \| YOR259C \| YDR394W \| YGL048C \| YGL137W \| YGR092W \| YKL145W \| YOR027W \| YOR117W \| YPL096W \| YGR040W \| YPR034W \| YIL007C |
| complex_2410 | YBL045C \| YOR231W \| YPR054W \| YPR191W \| YBL105C \| YDL029W \| YHR030C \| YGL096W \| YHL002W \| YHR108W \| YMR109W \| YHR086W \| YPL140C \| YJL095W \| YKL129C |
| complex_2772 | YBL016W \| YIL169C \| YBR160W \| YCR065W \| YJL187C \| YNL298W \| YBR200W \| YLR362W \| YPR119W \| YDL159W \| YHL007C \| YIL106W \| YKL129C \| YJL095W \| YMR109W |
| complex_2771 | YAL029C \| YBR081C \| YLR249W \| YOR290C \| YER164W \| YGR252W \| YBR198C \| YDR145W \| YDR216W \| YPR034W \| YDR146C \| YGR123C \| YOR257W \| YDR448W \| YLR291C |
| complex_1688 | YBL105C \| YBR160W \| YJL095W \| YOL100W \| YGL003C \| YCR065W \| YER165W \| YGR159C \| YKL025C \| YPR119W \| YJL060W \| YDL029W \| YKL129C \| YMR109W \| YOR231W |
| complex_1323 | YBR160W \| YGR092W \| YMR001C \| YNL298W \| YCR002C \| YCL024W \| YDR507C \| YDL225W \| YGL127C \| YHR107C \| YIL112W \| YKL101W \| YJR076C \| YLR314C \| YPR119W |
| complex_447 | YAL005C \| YBR155W \| YER081W \| YLR310C \| YPL204W \| YBL075C \| YFL016C \| YIL061C \| YOR222W \| YEL030W \| YOR232W \| YJR045C \| YLR369W \| YMR139W \| YNL027W |
| complex_442 | YAL032C \| YKL173W \| YLL036C \| YLR117C \| YLR424W \| YMR001C \| YMR213W \| YBR065C \| YDL030W \| YDL208W \| YER013W \| YGL120C \| YPL151C \| YKR024C \| YLR147C |
| complex_441 | YAL005C \| YER107C \| YGR159C \| YLL024C \| YLR310C \| YNL007C \| YNL209W \| YPR128C \| YDR477W \| YDL192W \| YER129W \| YER028C \| YPL106C \| YER165W \| YNL041C |
| complex_2408 | YBL075C \| YBR160W \| YCR065W \| YJL187C \| YKL129C \| YCR088W \| YDL029W \| YDR054C \| YHL007C \| YLR362W \| YHR018C \| YIL046W \| YJL194W \| YNL298W \| YPR119W |
| complex_2407 | YAL005C \| YBL075C \| YLL024C \| YLR310C \| YNL064C \| YNL209W \| YPR119W \| YBL016W \| YLR362W \| YFL029C \| YPR054W \| YGR040W \| YHR018C \| YOR027W \| YPL106C |
| complex_2769 | YAL005C \| YBL075C \| YBR155W \| YDL101C \| YJR032W \| YLR310C \| YOR027W \| YPL153C \| YMR190C \| YDL229W \| YEL030W \| YLL024C \| YPL106C \| YPL240C \| YLR347C |
| complex_2406 | YAL024C \| YNR047W \| YBL045C \| YCR065W \| YBL105C \| YBR160W \| YCR091W \| YER165W \| YOL100W \| YPR119W \| YER129W \| YJR059W \| YKL116C \| YKL129C \| YMR036C |
| complex_2405 | YBR109C \| YGL019W \| YOR039W \| YOR061W \| YDL195W \| YIL131C \| YER148W \| YFL039C \| YLR399C \| YIL118W \| YOL004W \| YIL035C \| YOR326W \| YPL204W \| YGL171W |
| complex_2404 | YBR160W \| YGR238C \| YJL187C \| YKL210W \| YBR200W \| YPR119W \| YDR146C \| YHL035C \| YPR120C \| YHR158C \| YLR096W \| YKL129C \| YNL068C \| YNL298W \| YPL119C |
| complex_2765 | YAL005C \| YBR155W \| YER081W \| YFL016C \| YLL024C \| YOR222W \| YBR274W \| YEL030W \| YDR155C \| YCR072C \| YFL037W \| YJR045C \| YKL073W \| YOR362C \| YLR009W |
| complex_2764 | YAR019C \| YBR160W \| YDL029W \| YLR131C \| YCR065W \| YDL155W \| YGR092W \| YKL129C \| YDR146C \| YPR119W \| YDR216W \| YPR110C \| YIL106W \| YNL161W \| YPR111W |

| | |
|---|---|
| complex_2760 | YAL032C \| YBL105C \| YLR117C \| YMR001C \| YMR213W \| YPL151C \| YDR432W \| YER165W \| YGL173C \| YGR159C \| YOR204W \| YPR119W \| YER013W \| YGL120C \| YKR024C |
| complex_1318 | YAR019C \| YNL161W \| YBR160W \| YCR065W \| YDL155W \| YHL007C \| YHR018C \| YKL129C \| YPR111W \| YPR119W \| YDR146C \| YLR131C \| YER103W \| YGR092W \| YIL106W |
| complex_1317 | YCR065W \| YBR160W \| YDL108W \| YFL029C \| YDR432W \| YER165W \| YGL173C \| YGR159C \| YKL139W \| YOR204W \| YPR119W \| YPR191W \| YPR054W \| YHR030C \| YLR096W |
| complex_1679 | YER148W \| YFL039C \| YGL019W \| YLR399C \| YOR039W \| YOR061W \| YIL118W \| YOL004W \| YHR023W \| YIL035C \| YIL131C \| YIL106W \| YDL028C \| YOL145C \| YOR326W |
| complex_1313 | YBR109C \| YDR356W \| YGL019W \| YOR039W \| YDL195W \| YIL131C \| YER007C-A \| YJR014W \| YGR116W \| YLR399C \| YML010W \| YOL004W \| YIL035C \| YOL145C \| YOR061W |
| complex_1312 | YAL005C \| YLR310C \| YNL064C \| YBL016W \| YBL075C \| YLL024C \| YNL209W \| YPR119W \| YDL229W \| YDR477W \| YPL106C \| YHR018C \| YLR362W \| YOR027W \| YPR054W |
| complex_1311 | YBR160W \| YIL046W \| YMR001C \| YCR002C \| YCL024W \| YDR507C \| YHR107C \| YNL298W \| YPR119W \| YGR092W \| YMR304W \| YJL060W \| YLR314C \| YJL187C \| YJR076C |
| complex_439 | YAL005C \| YER081W \| YER107C \| YER165W \| YLR310C \| YNL007C \| YNL209W \| YPR128C \| YCR008W \| YLL024C \| YDR146C \| YPL031C \| YPL106C \| YPR119W \| YNL041C |
| complex_1670 | YAL005C \| YDR477W \| YLR310C \| YNL007C \| YDL192W \| YDL229W \| YBL016W \| YER165W \| YGL158W \| YLR113W \| YHR018C \| YLL024C \| YPL106C \| YMR139W \| YNL209W |
| complex_438 | YAL005C \| YBR085W \| YDL229W \| YER165W \| YLR310C \| YNL064C \| YOR027W \| YOR388C \| YPL106C \| YBL075C \| YCR073C \| YJL128C \| YDR146C \| YLL024C \| YNL041C |
| complex_437 | YAL005C \| YBL075C \| YBR085W \| YER165W \| YLR310C \| YNL064C \| YNL209W \| YOR027W \| YOR388C \| YPL106C \| YDR146C \| YLL024C \| YJL187C \| YNL298W \| YNL041C |
| complex_797 | YAL005C \| YDL229W \| YER165W \| YKL139W \| YLR310C \| YNL007C \| YPL106C \| YBR169C \| YCL011C \| YDR432W \| YDL084W \| YNL004W \| YDR477W \| YER028C \| YNL112W |
| complex_434 | YAL032C \| YDR364C \| YKL173W \| YLL036C \| YLR117C \| YLR424W \| YMR001C \| YMR213W \| YPL151C \| YDL030W \| YER013W \| YGL120C \| YGL174W \| YMR240C \| YNL189W |
| complex_795 | YBR160W \| YGL003C \| YKL048C \| YMR001C \| YPR120C \| YCL024W \| YCR002C \| YHR107C \| YNL298W \| YDR507C \| YJR076C \| YLR314C \| YMR304W \| YKL101W \| YJL187C |
| complex_433 | YBR160W \| YGR092W \| YCR034W \| YDL007W \| YFR004W \| YDL097C \| YDR394W \| YGL048C \| YHR030C \| YKL145W \| YOR117W \| YOR259C \| YOR261C \| YDL147W \| YDL029W |
| complex_794 | YAL005C \| YBR155W \| YDL229W \| YER081W \| YER165W \| YLR310C \| YMR216C \| YNL007C \| YPL106C \| YPR128C \| YDR146C \| YDR432W \| YKL073W \| YHR030C \| YJL034W |
| complex_793 | YAL032C \| YKL173W \| YLL036C \| YLR117C \| YLR424W \| YMR213W \| YPL151C \| YBR160W \| YDL030W \| YER013W \| YMR001C \| YGL120C \| YGR136W \| YIL112W \| YMR240C |
| complex_2759 | YBR160W \| YGL048C \| YGR092W \| YKL145W \| YDR394W \| YDL007W \| YGL137W \| YOR117W \| YOR259C \| YFR010W \| YKL166C \| YML092C \| YMR001C \| YMR146C \| YNL250W |
| complex_2758 | YBL045C \| YPL203W \| YPR054W \| YPR191W \| YDR490C \| YKL166C \| YER165W \| YGL137W \| YHR030C \| YLR216C \| YHR205W \| YKL126W \| YIL033C \| YMR022W \| YJL164C |

| | |
|---|---|
| complex_2753 | YBL045C \| YCR065W \| YLR362W \| YBR133C \| YER165W \| YHL007C \| YJL187C \| YBR160W \| YBL105C \| YGL003C \| YMR001C \| YPR119W \| YJL060W \| YNL298W \| YOL100W |
| complex_2752 | YAL024C \| YBR160W \| YPR054W \| YBL105C \| YGL003C \| YCR065W \| YDL108W \| YFL029C \| YLR113W \| YER165W \| YPR119W \| YPR191W \| YJL060W \| YMR036C \| YOL100W |
| complex_2751 | YBL045C \| YBR160W \| YCR065W \| YBL105C \| YDL017W \| YER165W \| YJR059W \| YPR119W \| YGL137W \| YHR030C \| YJL095W \| YKL129C \| YNR047W \| YCR091W \| YOL100W |
| complex_1308 | YBR160W \| YBL105C \| YGR092W \| YMR001C \| YNL298W \| YCR065W \| YDR054C \| YER165W \| YPR119W \| YIL046W \| YIL106W \| YOL100W \| YPR111W \| YJL187C \| YKL129C |
| complex_1669 | YAL005C \| YDL229W \| YDR477W \| YLL024C \| YNL007C \| YNL209W \| YNL308C \| YPL106C \| YPR119W \| YNL175C \| YER028C \| YER129W \| YIL035C \| YPL131W |
| complex_1306 | YBR109C \| YGL019W \| YGL106W \| YOR039W \| YOR061W \| YDR216W \| YML010W \| YGL254W \| YIL118W \| YIL131C \| YML074C \| YIL035C \| YOL145C \| YOR326W |
| complex_1305 | YAR019C \| YBR160W \| YBR133C \| YER165W \| YHL007C \| YDL155W \| YGR092W \| YKL129C \| YNL161W \| YPR119W \| YLR362W \| YIL106W \| YJL187C \| YMR001C \| YNL298W |
| complex_1304 | YBR160W \| YGR092W \| YJL187C \| YLR314C \| YMR001C \| YNL298W \| YCL024W \| YLR259C \| YCR002C \| YDR507C \| YDL225W \| YHR107C \| YPR119W \| YHR098C \| YJR076C |
| complex_1665 | YAR019C \| YHR169W \| YBR160W \| YCR065W \| YDL155W \| YGL003C \| YGR092W \| YKL129C \| YLR131C \| YMR001C \| YNL102W \| YPR111W \| YPR119W \| YDR146C \| YIL106W |
| complex_1664 | YBR160W \| YDL155W \| YER008C \| YGL003C \| YGR092W \| YIL106W \| YJL187C \| YLR212C \| YMR001C \| YPR111W \| YPR119W \| YNL161W \| YMR304W \| YAR019C \| YML064C |
| complex_1300 | YAL005C \| YDR477W \| YER129W \| YJR032W \| YLL024C \| YLR310C \| YOR027W \| YBL075C \| YBR155W \| YPL240C \| YDL229W \| YMR290C \| YLR347C \| YNL209W \| YPL106C |
| complex_789 | YAL005C \| YLR310C \| YNL308C \| YBL075C \| YGL254W \| YNL007C \| YNL175C \| YNR047W \| YOR027W \| YCR091W \| YDR146C \| YER103W \| YNL209W \| YPL106C \| YLL024C |
| complex_787 | YAL005C \| YBL075C \| YDL229W \| YDR356W \| YDR477W \| YLL024C \| YLR310C \| YNL209W \| YOR027W \| YPL106C \| YPR119W \| YER081W \| YDR146C \| YBR160W \| YER103W |
| complex_425 | YAL005C \| YBR155W \| YER081W \| YFL016C \| YIL061C \| YLL024C \| YLR310C \| YOR222W \| YPL106C \| YDR146C \| YEL030W \| YJR045C \| YLR369W \| YDR477W \| YOR232W |
| complex_786 | YAL005C \| YDL229W \| YER081W \| YER165W \| YLR310C \| YNL007C \| YPL106C \| YPR119W \| YPR128C \| YBR160W \| YLR096W \| YDR146C \| YNL209W \| YHL002W \| YNL041C |
| complex_424 | YAL005C \| YBR085W \| YER081W \| YER107C \| YER165W \| YLR310C \| YNL064C \| YNL209W \| YOR027W \| YPL106C \| YBL075C \| YLL024C \| YNL041C \| YPL141C \| YLR113W |
| complex_785 | YAL005C \| YER107C \| YER165W \| YLR310C \| YNL064C \| YOR027W \| YPL106C \| YAR019C \| YBL075C \| YBR160W \| YGR092W \| YPL042C \| YDR146C \| YLL024C \| YKR036C |
| complex_423 | YAL005C \| YBL075C \| YBR085W \| YER081W \| YER107C \| YER165W \| YLL016W \| YLR310C \| YNL064C \| YOR027W \| YPL106C \| YGR040W \| YLR362W \| YLL024C \| YNL041C |
| complex_783 | YAL005C \| YER081W \| YER107C \| YER165W \| YHR030C \| YLL024C \| YLR090W \| YLR310C \| YNL041C \| YOR027W \| YPL106C \| YPR128C \| YBL045C \| YPR054W |

| | |
|---|---|
| complex_421 | YAL005C \| YBL075C \| YER081W \| YER165W \| YLL024C \| YLR310C \| YNL064C \| YOR027W \| YPR128C \| YDR146C \| YNL209W \| YPL106C \| YDR490C \| YHR205W \| YNL041C |
| complex_780 | YBR160W \| YCR065W \| YDR507C \| YKL129C \| YLR314C \| YMR001C \| YCL024W \| YCR002C \| YDL225W \| YHR107C \| YKL101W \| YNL298W \| YJL187C \| YPR119W \| YJR076C |
| complex_2748 | YAL024C \| YBR160W \| YDL025C \| YBL045C \| YCR065W \| YPR191W \| YBL105C \| YER165W \| YJR059W \| YOL100W \| YPR119W \| YNR047W \| YKL116C \| YMR036C \| YPL209C |
| complex_2747 | YAL005C \| YER165W \| YLR310C \| YNL064C \| YPL106C \| YPR112C \| YBL016W \| YDL159W \| YDR388W \| YFR024C-A \| YLR362W \| YBL075C \| YGR040W \| YJL128C \| YLL024C |
| complex_2744 | YBL016W \| YLR210W \| YOR212W \| YBR160W \| YDL159W \| YGR040W \| YLR362W \| YDR212W \| YBR198C \| YIL142W \| YJL106W \| YJR064W \| YNL317W \| YPL115C \| YNL298W |
| complex_2740 | YAL024C \| YDL101C \| YBL045C \| YCR065W \| YPR054W \| YPR191W \| YBL105C \| YBR160W \| YER165W \| YHR030C \| YOL100W \| YPR119W \| YJR059W \| YMR036C \| YDR155C |
| complex_1659 | YAL005C \| YDL229W \| YER165W \| YLR310C \| YAL017W \| YDR477W \| YOL045W \| YPL042C \| YBL075C \| YDR216W \| YNL209W \| YER028C \| YLL024C \| YPL106C |
| complex_1658 | YAL005C \| YBL075C \| YLL024C \| YLR310C \| YBL016W \| YLR362W \| YDL159W \| YGR040W \| YDL229W \| YDR477W \| YPL106C \| YGL158W \| YER036C \| YNL064C \| YNL209W |
| complex_1653 | YAL005C \| YDL229W \| YDR172W \| YER165W \| YNL209W \| YPR119W \| YBL105C \| YDR432W \| YGR159C \| YKR095W \| YOR204W \| YDR477W \| YGR162W \| YLL024C \| YPL106C |
| complex_1652 | YAL005C \| YDR356W \| YLL024C \| YLR310C \| YNL064C \| YNL209W \| YPR119W \| YBL016W \| YBL075C \| YDL229W \| YDR477W \| YPL106C \| YDL192W \| YER165W \| YMR139W |
| complex_779 | YAL005C \| YCL011C \| YDL229W \| YLR310C \| YNL007C \| YPL106C \| YPR128C \| YDR477W \| YER028C \| YNL209W \| YER133W \| YJR093C \| YKL059C \| YPR107C \| YNL041C |
| complex_417 | YAL005C \| YBR085W \| YER081W \| YER107C \| YER165W \| YLL016W \| YLR310C \| YNL064C \| YOR027W \| YPL106C \| YBL075C \| YDR507C \| YMR139W \| YLL024C \| YNL041C |
| complex_778 | YDL029W \| YDR394W \| YNL189W \| YOR259C \| YDL097C \| YDL007W \| YGL048C \| YGR040W \| YHR027C \| YHR030C \| YKL145W \| YOR117W \| YOR261C \| YDL147W \| YPL140C |
| complex_777 | YAL032C \| YKL173W \| YLL036C \| YLR117C \| YLR424W \| YMR001C \| YMR213W \| YDL030W \| YDL145C \| YDL208W \| YER013W \| YGL120C \| YJL187C \| YMR240C \| YPL151C |
| complex_776 | YAL005C \| YER107C \| YER165W \| YLL016W \| YLR310C \| YNL007C \| YNL209W \| YDR477W \| YER129W \| YLL024C \| YMR104C \| YDR490C \| YER028C \| YPL106C \| YHR205W |
| complex_775 | YDL007W \| YFR004W \| YDL097C \| YDR394W \| YGL048C \| YKL145W \| YOR117W \| YOR259C \| YOR261C \| YDL147W \| YDL029W \| YMR104C \| YHR205W \| YDR490C \| YKL126W |
| complex_774 | YAL005C \| YBL075C \| YBR155W \| YDL229W \| YDR477W \| YGL070C \| YLL024C \| YLR310C \| YNL064C \| YNL209W \| YNL308C \| YPL106C \| YAR018C \| YMR116C \| YNL175C |
| complex_773 | YAL005C \| YBL075C \| YBR085W \| YBR155W \| YER081W \| YER165W \| YLR310C \| YNL041C \| YNL064C \| YPL042C \| YCR084C \| YDR146C \| YEL030W \| YLR216C |
| complex_772 | YAR019C \| YER028C \| YGR092W \| YKR036C \| YDL007W \| YFR004W \| YDL097C \| YDR394W \| YGL048C \| YKL145W \| YOR117W \| YOR259C \| YOR261C \| YDL147W \| YPL026C |

| | |
|---|---|
| complex_410 | YAL032C \| YKL173W \| YLL036C \| YLR117C \| YLR424W \| YMR001C \| YMR213W \| YPL151C \| YDL030W \| YDR473C \| YER013W \| YGL120C \| YGL174W \| YIR005W \| YMR240C |
| complex_2739 | YAL005C \| YJL095W \| YOL038W \| YBL105C \| YBR160W \| YER165W \| YOL100W \| YOR231W \| YPL151C \| YPR119W \| YNL102W \| YCR065W \| YJL060W \| YKL129C \| YMR001C |
| complex_2737 | YCR065W \| YBR160W \| YDL229W \| YNL298W \| YDR356W \| YPR119W \| YDR379W \| YGR152C \| YLR229C \| YER129W \| YAL041W \| YOR127W \| YHL007C \| YKL129C \| YJL187C |
| complex_2732 | YBL045C \| YMR104C \| YDR490C \| YKL166C \| YER165W \| YJL164C \| YGL137W \| YHR030C \| YLR216C \| YHR205W \| YKL126W \| YIL033C \| YJR017C \| YNL027W \| YPL203W |
| complex_2731 | YBR160W \| YGR092W \| YIL112W \| YJL187C \| YLR314C \| YMR001C \| YNL298W \| YCR002C \| YDL225W \| YDR507C \| YHR107C \| YJL060W \| YJR076C \| YKL048C \| YNL189W |
| complex_1649 | YBL105C \| YJL095W \| YOL100W \| YBR160W \| YCR065W \| YDL155W \| YGR092W \| YKL129C \| YDR146C \| YLR131C \| YPR119W \| YIL106W \| YNL161W \| YPR111W \| YMR109W |
| complex_1646 | YBL075C \| YGR092W \| YNL064C \| YPL042C \| YBR160W \| YCR065W \| YDL155W \| YKL129C \| YNL102W \| YAR019C \| YHR018C \| YIL106W \| YPR111W \| YPR119W \| YMR001C |
| complex_1645 | YCL024W \| YLR314C \| YNL189W \| YCR002C \| YDR507C \| YDL225W \| YDR148C \| YHR107C \| YDR395W \| YER165W \| YLR335W \| YMR001C \| YJR076C \| YKL068W \| YMR047C |
| complex_1643 | YDL081C \| YIL035C \| YIL118W \| YLR340W \| YDL195W \| YGL019W \| YIL131C \| YOR061W \| YER148W \| YFL039C \| YLR399C \| YOR039W \| YOL004W \| YOL145C \| YOR326W |
| complex_769 | YAL032C \| YDR364C \| YKL173W \| YLR117C \| YLR424W \| YMR001C \| YMR213W \| YBR065C \| YDL030W \| YER013W \| YER029C \| YHR086W \| YLR275W \| YMR240C \| YPL151C |
| complex_407 | YAL005C \| YDL229W \| YDR356W \| YER081W \| YER165W \| YGR159C \| YLL024C \| YLR310C \| YNL007C \| YNL209W \| YPR128C \| YDR477W \| YER028C \| YPL106C \| YNL041C |
| complex_767 | YAL005C \| YBL075C \| YBR155W \| YDL229W \| YDR477W \| YGL070C \| YLL024C \| YLR310C \| YNL064C \| YNL209W \| YNL308C \| YPL106C \| YNL175C \| YMR116C \| YAR018C |
| complex_766 | YAL005C \| YDL229W \| YIL061C \| YLR310C \| YNL007C \| YPR128C \| YIL033C \| YPL203W \| YJL164C \| YPL031C \| YKL166C \| YNL027W \| YNL209W \| YPL106C \| YNL041C |
| complex_403 | YAL005C \| YER081W \| YER165W \| YLR310C \| YNL007C \| YNL209W \| YOR027W \| YPL106C \| YPR128C \| YDL229W \| YDR477W \| YNL041C \| YER028C \| YGL158W \| YLL024C |
| complex_2727 | YDL087C \| YHR086W \| YIL061C \| YKL012W \| YKL173W \| YDL208W \| YDR416W \| YLR117C \| YMR001C \| YPL151C \| YER029C \| YGL120C \| YNL245C \| YLR147C \| YOR319W |
| complex_2726 | YAL024C \| YBR160W \| YJL141C \| YBL105C \| YER165W \| YJL095W \| YLR096W \| YOL100W \| YPR119W \| YGL003C \| YMR001C \| YJL187C \| YMR304W \| YHR158C \| YJL060W |
| complex_2723 | YBR034C \| YIL061C \| YDL087C \| YHR086W \| YKL012W \| YDR432W \| YKR095W \| YER029C \| YPL151C \| YLR117C \| YLR298C \| YMR001C \| YMR240C \| YNL189W \| YNL245C |
| complex_2722 | YBL045C \| YGR092W \| YDL007W \| YDL029W \| YDL047W \| YDR394W \| YGL048C \| YKL145W \| YOR117W \| YOR259C \| YEL037C \| YGL137W \| YKL166C \| YMR216C \| YIL007C |
| complex_1639 | YAR019C \| YBR160W \| YGR092W \| YDL029W \| YDL007W \| YEL037C \| YKL145W \| YDR394W \| YGL048C \| YOR117W \| YOR259C \| YIL106W \| YPR111W \| YPR119W \| YJL194W |

| | |
|---|---|
| complex_1636 | YAL041W \| YJL187C \| YLR229C \| YNL298W \| YBR160W \| YIL112W \| YCL024W \| YCR002C \| YDR507C \| YHR107C \| YHL007C \| YLR362W \| YJR076C \| YLR314C \| YKL129C |
| complex_1635 | YBR160W \| YFL009W \| YGR092W \| YIL046W \| YJL187C \| YKL129C \| YDL132W \| YDL155W \| YDR054C \| YIL106W \| YPR119W \| YJL060W \| YOR319W \| YNL298W \| YOL133W |
| complex_1994 | YAL005C \| YBL075C \| YBR155W \| YJR032W \| YLR310C \| YNL064C \| YOR027W \| YPL153C \| YHR018C \| YDL101C \| YLL024C \| YPL106C \| YPL240C \| YLR347C \| YMR190C |
| complex_1993 | YBL045C \| YBR160W \| YCR065W \| YBL105C \| YGL003C \| YDL155W \| YGR092W \| YDR054C \| YER165W \| YNL298W \| YPR119W \| YIL046W \| YJL060W \| YJL187C \| YMR001C |
| complex_1992 | YBR160W \| YGL003C \| YNR047W \| YPR120C \| YCR091W \| YDR212W \| YBR198C \| YJL106W \| YJL164C \| YKR036C \| YLL011W \| YNL317W \| YIL142W \| YJR064W \| YJL014W |
| complex_759 | YAL032C \| YBL105C \| YKL173W \| YLL036C \| YLR117C \| YLR424W \| YMR213W \| YDL030W \| YER013W \| YGL120C \| YKL181W \| YHL011C \| YMR001C \| YMR240C \| YPL151C |
| complex_756 | YAL005C \| YLR310C \| YBL016W \| YBL075C \| YDL159W \| YLR362W \| YNL064C \| YOR027W \| YDL229W \| YDR477W \| YGL070C \| YPL106C \| YGR040W \| YPL240C \| YLL024C |
| complex_753 | YAL032C \| YBL105C \| YKL173W \| YLL036C \| YLR117C \| YLR424W \| YMR213W \| YDL030W \| YER013W \| YGL120C \| YGL174W \| YMR001C \| YNL189W \| YMR240C \| YPL151C |
| complex_750 | YBR160W \| YAL041W \| YJL187C \| YLR314C \| YNL298W \| YCL024W \| YCR002C \| YIL046W \| YJR076C \| YDL225W \| YHR107C \| YNL007C \| YDR507C \| YKL101W \| YMR304W |
| complex_2718 | YBR160W \| YBR200W \| YGL003C \| YJL187C \| YKL116C \| YKL129C \| YMR001C \| YNL068C \| YNL102W \| YNL298W \| YNR047W \| YPR119W \| YPR120C \| YHL035C \| YJR059W |
| complex_2717 | YCR084C \| YPL042C \| YDR477W \| YGR092W \| YGL019W \| YGL254W \| YIL035C \| YIL118W \| YIL131C \| YKR036C \| YML010W \| YML074C \| YOR039W \| YOL145C \| YOR061W |
| complex_2715 | YAL017W \| YBR160W \| YBL045C \| YCR065W \| YGR159C \| YBL105C \| YPL151C \| YOL100W \| YDR432W \| YER165W \| YPR119W \| YKL025C \| YNL068C \| YOL045W |
| complex_2714 | YAL005C \| YBR155W \| YIL061C \| YOR254C \| YHR079C \| YIL033C \| YJL034W \| YLR248W \| YPL203W \| YJL164C \| YKL073W \| YKL166C \| YLR113W \| YNL027W |
| complex_2711 | YDL155W \| YLR131C \| YMR109W \| YNL161W \| YDR216W \| YDL029W \| YJL095W \| YNL007C \| YEL030W \| YHR030C \| YHR102W \| YFL034C-B \| YHL007C \| YKL129C \| YPL140C |
| complex_2710 | YBR160W \| YDR212W \| YGR092W \| YJL164C \| YJL187C \| YMR001C \| YDL145C \| YDR075W \| YGL137W \| YKL166C \| YNL317W \| YIL033C \| YPL203W \| YIL142W \| YLR216C |
| complex_1629 | YBR109C \| YGL019W \| YOR039W \| YOR061W \| YDL014W \| YML010W \| YDR216W \| YER007C-A \| YJR014W \| YGL254W \| YIL118W \| YIL131C \| YML074C \| YIL035C \| YOL145C |
| complex_1628 | YAL005C \| YLR310C \| YPL106C \| YBL016W \| YDL159W \| YGL158W \| YLR362W \| YBL075C \| YDL229W \| YMR139W \| YDR477W \| YLL024C \| YNL209W \| YPL042C \| YGR040W |
| complex_1626 | YAL005C \| YLR310C \| YBL075C \| YOR027W \| YPL042C \| YPL240C \| YPR119W \| YDL229W \| YER081W \| YDR477W \| YLL024C \| YOR317W \| YGR092W \| YNL209W \| YPL106C |
| complex_1988 | YAL029C \| YBR109C \| YFL005W \| YFL039C \| YGL106W \| YHR023W \| YOR035C \| YLR433C \| YML057W \| YHR082C \| YJL095W \| YKL129C \| YKL190W \| YMR109W \| YOR326W |

| | |
|---|---|
| complex_1625 | YAL005C \| YLR310C \| YBL016W \| YDL159W \| YGR040W \| YBL075C \| YNL064C \| YOR027W \| YHR018C \| YLL024C \| YLR362W \| YNL298W \| YPL240C \| YCR088W \| YPR119W |
| complex_1987 | YBR109C \| YGL019W \| YLR212C \| YOR039W \| YOR061W \| YDL195W \| YIL035C \| YIL131C \| YIL118W \| YJL164C \| YIL033C \| YKL166C \| YNL027W \| YPL204W \| YPL203W |
| complex_1986 | YBL105C \| YBR160W \| YCR091W \| YER165W \| YOL100W \| YPR119W \| YNR047W \| YIL035C \| YML010W \| YGL019W \| YIL131C \| YML074C \| YOR039W \| YOR061W \| YOL145C |
| complex_1623 | YBR109C \| YGL019W \| YLR212C \| YOR039W \| YOR061W \| YDL130W \| YIL035C \| YIL118W \| YER165W \| YML010W \| YGL254W \| YIL131C \| YML074C \| YOL145C \| YOR326W |
| complex_1984 | YAR019C \| YGR092W \| YBR160W \| YDR394W \| YDL007W \| YDL029W \| YGL048C \| YKL145W \| YOR117W \| YOR259C \| YEL037C \| YPL096W \| YIL106W \| YNL161W \| YPR111W |
| complex_1982 | YBL075C \| YLR362W \| YOR362C \| YBR160W \| YHL007C \| YIL046W \| YJL187C \| YBR200W \| YAL041W \| YBL016W \| YDL159W \| YDR054C \| YKL129C \| YNL298W \| YNL007C |
| complex_747 | YAL005C \| YER107C \| YER165W \| YLL016W \| YLR310C \| YNL064C \| YPL106C \| YBL075C \| YDL159W \| YBL016W \| YGL158W \| YGR040W \| YLL024C \| YMR139W \| YLR362W |
| complex_744 | YBR065C \| YLR117C \| YMR001C \| YMR213W \| YPL151C \| YPR182W \| YDL087C \| YKL173W \| YDL208W \| YDR235W \| YER029C \| YGL120C \| YHR086W \| YIL061C \| YLR147C |
| complex_742 | YAL005C \| YER081W \| YFL016C \| YLL024C \| YLR310C \| YMR001C \| YNL041C \| YNL175C \| YPL106C \| YPR128C \| YDR088C \| YDR146C \| YJR045C \| YLR369W \| YOR232W |
| complex_2708 | YBR160W \| YBR200W \| YER008C \| YGL003C \| YGR092W \| YJL187C \| YKL129C \| YLR210W \| YMR001C \| YNL298W \| YPL209C \| YPR119W \| YDL145C \| YMR304W \| YIL046W |
| complex_2707 | YBR160W \| YDR507C \| YER008C \| YGL003C \| YJL187C \| YLR210W \| YMR001C \| YPL209C \| YPR119W \| YIL046W \| YKR048C \| YMR304W \| YHL035C \| YJR059W \| YMR139W |
| complex_2706 | YAL005C \| YBL075C \| YLR310C \| YPR119W \| YBL105C \| YGR092W \| YBR160W \| YDL108W \| YDL155W \| YDR146C \| YGL003C \| YJL187C \| YMR001C \| YER165W \| YJL060W |
| complex_2704 | YBL016W \| YBR200W \| YDL159W \| YLR362W \| YBL075C \| YNL298W \| YBR160W \| YCR065W \| YHR018C \| YPR119W \| YFL039C \| YGR040W \| YHR023W \| YJL187C \| YOR326W |
| complex_2703 | YBL026W \| YER112W \| YER146W \| YBR274W \| YDL145C \| YGL137W \| YJL124C \| YMR304W \| YFR052W \| YPR182W \| YJL187C \| YNL178W \| YJR022W \| YNL147W \| YOR362C |
| complex_2701 | YCR065W \| YBR160W \| YDR379W \| YGR152C \| YLR229C \| YPR119W \| YDR507C \| YAL041W \| YLR314C \| YOR127W \| YGR108W \| YHL007C \| YKL129C \| YJL187C \| YNL298W |
| complex_1616 | YBL016W \| YBR274W \| YDL159W \| YLR362W \| YBL026W \| YER112W \| YER146W \| YJL124C \| YBL075C \| YHR018C \| YGR040W \| YJR022W \| YFR052W \| YPR182W \| YNL147W |
| complex_1976 | YBL105C \| YBR160W \| YOL100W \| YPL151C \| YPR119W \| YPR120C \| YAL019W \| YER008C \| YGL003C \| YMR001C \| YPL209C \| YMR304W \| YJL187C \| YNL298W \| YKR048C |
| complex_1613 | YAL005C \| YLR310C \| YNL007C \| YOR027W \| YPL106C \| YBL016W \| YDL159W \| YGR040W \| YBL075C \| YLR362W \| YJL128C \| YLL024C \| YLL039C \| YMR022W \| YNR031C |
| complex_1975 | YBL105C \| YBR160W \| YJL095W \| YPR119W \| YPR120C \| YER008C \| YGL003C \| YGR238C \| YHR030C \| YHR158C \| YJL187C \| YLR096W \| YMR304W \| YOL100W \| YPL140C |

| | |
|---|---|
| complex_1612 | YBR109C \| YGL019W \| YGR270W \| YOR039W \| YOR061W \| YGL254W \| YIL118W \| YIL131C \| YML010W \| YML074C \| YMR116C \| YJR014W \| YIL035C \| YOL145C |
| complex_1974 | YBR160W \| YHL007C \| YJL187C \| YLR314C \| YNL298W \| YCL024W \| YCR002C \| YDR507C \| YLR229C \| YDL225W \| YHR107C \| YGR152C \| YDL240W \| YJR076C \| YKL129C |
| complex_1973 | YBL105C \| YPL151C \| YPR119W \| YPR120C \| YBR160W \| YBR200W \| YGL003C \| YGR092W \| YJL187C \| YKL129C \| YMR001C \| YNL068C \| YNL102W \| YNL298W \| YOL100W |
| complex_1972 | YAL005C \| YBL105C \| YLR310C \| YNL007C \| YBL075C \| YPR119W \| YBR160W \| YJL095W \| YCR065W \| YER165W \| YHR018C \| YHR030C \| YKL129C \| YLR096W \| YOL100W |
| complex_1971 | YAL005C \| YLR310C \| YNL007C \| YBL075C \| YCL024W \| YJL187C \| YLR362W \| YDL159W \| YBL016W \| YGR040W \| YDR054C \| YIL046W \| YLL039C \| YLL024C \| YNL298W |
| complex_739 | YAL005C \| YBR160W \| YER081W \| YER165W \| YLL024C \| YLR310C \| YNL041C \| YOR027W \| YOR254C \| YPL106C \| YPR128C \| YJL034W \| YKL073W \| YDR146C \| YNR047W |
| complex_737 | YAL005C \| YBL075C \| YDR477W \| YLL024C \| YLR310C \| YNL064C \| YNL175C \| YNL209W \| YNL308C \| YOR027W \| YPL042C \| YPL106C \| YDL229W \| YGL070C |
| complex_731 | YAL005C \| YDL229W \| YER081W \| YER165W \| YLR310C \| YNL007C \| YPL106C \| YPR119W \| YPR128C \| YBR160W \| YDR507C \| YFR027W \| YDR146C \| YNL209W \| YNL041C |
| complex_1605 | YAL005C \| YDR477W \| YLR310C \| YBL075C \| YOR027W \| YPL042C \| YPL240C \| YPR119W \| YDL229W \| YER081W \| YDR356W \| YER028C \| YLL024C \| YNL209W \| YPL106C |
| complex_1966 | YAL005C \| YBL075C \| YBR155W \| YJR032W \| YKL171W \| YLR310C \| YOR027W \| YDL229W \| YDR477W \| YKR059W \| YLL024C \| YPL106C \| YPL240C \| YLR347C \| YOL045W |
| complex_1965 | YBR160W \| YDR054C \| YER008C \| YHL007C \| YLR362W \| YMR001C \| YBR200W \| YAL041W \| YLR229C \| YNL298W \| YIL046W \| YMR304W \| YJL095W \| YJL187C \| YKL129C |
| complex_1602 | YDR432W \| YER165W \| YGR159C \| YIR001C \| YOL123W \| YOR204W \| YPR119W \| YGL049C \| YLR175W \| YNL016W \| YCR057C \| YGR162W \| YHR086W \| YJL033W \| YKL139W |
| complex_729 | YAL005C \| YBR155W \| YER081W \| YFL016C \| YIL061C \| YLR310C \| YOR222W \| YBR143C \| YDR477W \| YEL030W \| YLR369W \| YOR232W \| YJR045C \| YLL024C |
| complex_1960 | YAL024C \| YBR160W \| YMR001C \| YNL102W \| YER165W \| YGR092W \| YLR096W \| YPR191W \| YGL019W \| YIL131C \| YNL068C \| YHR158C \| YPR119W \| YJL187C \| YML064C |
| complex_726 | YAL005C \| YDL229W \| YER165W \| YLR310C \| YNL007C \| YOR027W \| YPR128C \| YDR432W \| YPL263C \| YDR477W \| YER103W \| YER028C \| YPL106C \| YGR159C \| YKL139W |
| complex_724 | YAL005C \| YBR085W \| YBR155W \| YER081W \| YIL061C \| YLL024C \| YLR310C \| YNL007C \| YNL209W \| YDR146C \| YEL030W \| YPL106C \| YHR030C \| YPL140C \| YJL095W |
| complex_723 | YDL007W \| YFR004W \| YDL097C \| YDR394W \| YGL048C \| YGR262C \| YKL145W \| YOR117W \| YOR259C \| YOR261C \| YDL147W \| YGR092W \| YHR129C \| YPL026C \| YGL084C |
| complex_722 | YBL075C \| YBR085W \| YBR155W \| YER081W \| YFL016C \| YIL061C \| YLR310C \| YBL105C \| YJR045C \| YLR369W \| YDR146C \| YEL030W \| YNL041C \| YOR232W \| YOR231W |
| complex_721 | YAL032C \| YKL173W \| YLL036C \| YLR117C \| YLR424W \| YMR001C \| YMR213W \| YDL030W \| YER013W \| YGL120C \| YGL174W \| YGR092W \| YHR114W \| YMR240C \| YPL151C |

| | |
|---|---|
| complex_1959 | YAR019C \| YGR092W \| YMR139W \| YBR160W \| YCR065W \| YDR507C \| YER021W \| YIL106W \| YKL145W \| YPR111W \| YDL097C \| YDR394W \| YFR004W \| YOR261C \| YDL147W |
| complex_1956 | YAR019C \| YOR061W \| YBR160W \| YPR111W \| YGL019W \| YIL035C \| YIL118W \| YLR399C \| YOL004W \| YOR039W \| YGR092W \| YNL288W \| YIL106W \| YIL131C \| YOL145C |
| complex_1955 | YBR160W \| YER008C \| YGL003C \| YGL137W \| YGR238C \| YHR158C \| YLR096W \| YPR119W \| YPR120C \| YMR304W \| YGR092W \| YMR001C \| YHR030C \| YJL095W \| YJL187C |
| complex_1952 | YAL005C \| YBL075C \| YBR085W \| YER165W \| YLR310C \| YNL064C \| YBL016W \| YDL159W \| YJL128C \| YDR388W \| YFR024C-A \| YGR040W \| YLR362W |
| complex_716 | YAL005C \| YBR085W \| YER165W \| YLR310C \| YNL007C \| YOR027W \| YPL106C \| YBL075C \| YLR362W \| YBR160W \| YHL007C \| YDR146C \| YLL024C \| YNL041C \| YNL064C |
| complex_715 | YAL005C \| YER081W \| YER165W \| YLR310C \| YNL007C \| YNL209W \| YPR128C \| YBR160W \| YPR119W \| YPR161C \| YDL229W \| YDR146C \| YPL106C \| YDR356W \| YNL041C |
| complex_710 | YAL005C \| YLR310C \| YNL308C \| YBL075C \| YOR011W \| YOR027W \| YDL229W \| YDR477W \| YPL106C \| YPL240C \| YNL209W \| YER028C \| YGL070C \| YLL024C \| YNL064C |
| complex_1947 | YBR020W \| YNL004W \| YCL011C \| YDL084W \| YDR432W \| YGL008C \| YKL139W \| YNL112W \| YPR191W \| YGL173C \| YOL123W \| YFL029C \| YFL039C \| YHR030C \| YPR054W |
| complex_1946 | YDR378C \| YER112W \| YER146W \| YJL124C \| YJR022W \| YLR275W \| YNL147W \| YPR182W \| YMR213W \| YMR001C \| YOR159C |
| complex_1943 | YBR160W \| YFL029C \| YLR096W \| YNL102W \| YCR065W \| YPR054W \| YDL108W \| YDR432W \| YGL173C \| YHR086W \| YKL139W \| YPR119W \| YPR191W \| YHR030C \| YKL129C |
| complex_1942 | YAL005C \| YBR155W \| YDR477W \| YIL061C \| YLL024C \| YLR248W \| YLR310C \| YOR254C \| YPL106C \| YDL047W \| YHR079C \| YIL033C \| YJL034W \| YKL073W \| YNL027W |
| complex_709 | YAL032C \| YKL173W \| YLL036C \| YLR117C \| YLR424W \| YMR213W \| YDL030W \| YDL214C \| YEL013W \| YMR001C \| YER013W \| YGL120C \| YGL174W \| YMR240C \| YPL151C |
| complex_1940 | YBL045C \| YNL016W \| YBL105C \| YER165W \| YPL151C \| YPR119W \| YBR233W \| YDR432W \| YHR086W \| YIR001C \| YOR204W \| YGL049C \| YGR159C \| YIL035C \| YGR162W |
| complex_706 | YAL032C \| YKL173W \| YLL036C \| YLR117C \| YLR424W \| YMR213W \| YDL030W \| YDR283C \| YER013W \| YMR001C \| YFR024C-A \| YGL120C \| YGL174W \| YMR240C \| YPL151C |
| complex_705 | YAL005C \| YBR155W \| YLR293C \| YLR310C \| YNL007C \| YNL308C \| YPL106C \| YBL075C \| YBR017C \| YOR185C \| YDL229W \| YDR477W \| YLL024C \| YGL070C \| YNL209W |
| complex_703 | YAL005C \| YNL308C \| YBL075C \| YER081W \| YLR310C \| YNL064C \| YBR155W \| YDL229W \| YDR356W \| YDR477W \| YGL070C \| YPL106C \| YLL024C \| YNL209W \| YOR185C |
| complex_700 | YAL005C \| YBL075C \| YER165W \| YLR310C \| YNL025C \| YNL064C \| YNL209W \| YOR027W \| YCR084C \| YOL004W \| YDR477W \| YER028C \| YLL024C \| YMR037C \| YPL042C |
| complex_1939 | YAL005C \| YLR310C \| YNL064C \| YPL106C \| YBL016W \| YCR088W \| YDL159W \| YHR018C \| YBL075C \| YNL298W \| YGR040W \| YLL024C \| YMR139W \| YLR362W \| YOR326W |
| complex_1938 | YAL005C \| YDR477W \| YIL013C \| YLL024C \| YLR310C \| YNL209W \| YOR027W \| YBL075C \| YDL074C \| YJL074C \| YDL229W \| YNL308C \| YER103W \| YNL064C \| YPL106C |
| complex_1937 | YBR109C \| YGL019W \| YGR270W \| YOR039W \| YOR061W \| YGL254W \| YIL118W \| YIL131C \| YML010W \| YML074C \| YIL035C \| YOL145C \| YOR254C \| |

| | |
|---|---|
| | YOR326W |
| complex_1934 | YBL075C \| YDR523C \| YNL064C \| YBR160W \| YCR065W \| YCR088W \| YHR018C \| YKL129C \| YNL298W \| YPR119W \| YBR200W \| YDL029W \| YHL007C \| YJL187C \| YLR362W |
| complex_1933 | YAL005C \| YBL016W \| YLR310C \| YNL025C \| YOR027W \| YOR388C \| YBL075C \| YPL042C \| YDL229W \| YPL240C \| YPR119W \| YDR477W \| YLL024C \| YNL209W \| YLR362W |
| complex_1932 | YDL007W \| YEL037C \| YHR027C \| YDR216W \| YGR123C \| YPL240C \| YDR394W \| YGL048C \| YKL145W \| YOR027W \| YOR117W \| YOR259C \| YHR169W \| YNR011C \| YIL148W |
| complex_1931 | YBL105C \| YBR160W \| YER165W \| YJR059W \| YOL100W \| YPR119W \| YDL025C \| YIL035C \| YML010W \| YGL019W \| YIL131C \| YML074C \| YOR039W \| YOR061W \| YOL145C |
| complex_1930 | YBR160W \| YER021W \| YGL048C \| YGR232W \| YIL112W \| YKL145W \| YOL004W \| YDL007W \| YDL029W \| YDR394W \| YOR259C \| YDL047W \| YGL004C \| YOR117W \| YKL129C |
| complex_1929 | YAL005C \| YBL075C \| YBR155W \| YJR032W \| YLR310C \| YNL064C \| YOR027W \| YPL141C \| YGR040W \| YHR018C \| YLL024C \| YPL240C \| YLR347C \| YJL138C \| YLR362W |
| complex_1927 | YBR160W \| YHL007C \| YJL187C \| YLR314C \| YNL298W \| YCL024W \| YCR002C \| YDR507C \| YLR229C \| YDL225W \| YHR107C \| YJR076C \| YKL129C \| YLR362W \| YOL113W |
| complex_1924 | YCL011C \| YDL084W \| YDR432W \| YKL139W \| YPR191W \| YDL029W \| YHR030C \| YGL173C \| YNL016W \| YFL029C \| YPR054W \| YJL095W \| YKL129C \| YPL140C \| YMR109W |
| complex_1921 | YDR477W \| YER129W \| YGL011C \| YGL158W \| YGR135W \| YML092C \| YMR104C \| YMR314W \| YNL209W \| YOL038W \| YOR362C \| YER012W \| YCR057C \| YDR490C \| YMR308C |
| complex_1919 | YBL045C \| YCR065W \| YPR054W \| YBR160W \| YDL108W \| YFL029C \| YNL102W \| YPR119W \| YKL139W \| YDR432W \| YER165W \| YGL173C \| YGR159C \| YOR204W \| YOR184W |
| complex_1917 | YBL045C \| YCR065W \| YNL016W \| YDR432W \| YER165W \| YGR159C \| YHR086W \| YIR001C \| YOR204W \| YPR119W \| YDR489W \| YGL049C \| YGR162W \| YKR059W \| YKL025C |
| complex_1916 | YAR019C \| YBR160W \| YGR092W \| YJL187C \| YNL102W \| YCR065W \| YDL155W \| YDL185W \| YKL048C \| YHR018C \| YIL106W \| YPR111W \| YPR119W \| YKL129C \| YMR001C |
| complex_1915 | YBL045C \| YCR065W \| YOL100W \| YBL105C \| YBR160W \| YJL095W \| YOR231W \| YNL298W \| YBR200W \| YHL007C \| YPR119W \| YER165W \| YKL129C \| YHR098C \| YJL187C |
| complex_1914 | YBL045C \| YBR160W \| YIL131C \| YBL105C \| YDR432W \| YER165W \| YGL173C \| YGR159C \| YOR204W \| YPR119W \| YJL095W \| YOR061W \| YGL019W \| YOL100W \| YOR231W |
| complex_1910 | YAL005C \| YDL229W \| YDR172W \| YDR356W \| YLR335W \| YDR477W \| YER081W \| YGL076C \| YLL024C \| YNL209W \| YER165W \| YPR119W \| YJL164C \| YPL106C \| YNL027W |
| complex_1909 | YBL105C \| YBR160W \| YER165W \| YGL003C \| YPR119W \| YPR120C \| YBR133C \| YJL187C \| YKL101W \| YNL298W \| YMR304W \| YGR092W \| YJL060W \| YMR001C \| YNL068C |
| complex_1906 | YBL045C \| YCR065W \| YPR054W \| YPR191W \| YBL105C \| YLR096W \| YBR160W \| YDL108W \| YFL029C \| YNL102W \| YPR119W \| YER165W \| YHL036W \| YGL137W \| YHR030C |
| complex_1904 | YAL005C \| YAR018C \| YBR155W \| YDL229W \| YER081W \| YNL175C \| YNL209W \| YNL308C \| YPL106C \| YDR356W \| YFL037W \| YHR064C \| YLL024C |

| | |
|---|---|
| complex_1903 | YBR160W \| YDR507C \| YER008C \| YGL003C \| YGR092W \| YMR001C \| YNL068C \| YPL209C \| YPR119W \| YPR120C \| YDL145C \| YMR304W \| YKR048C \| YHL035C \| YJL187C |
| complex_1902 | YBR160W \| YGL003C \| YCR065W \| YDL155W \| YDR146C \| YPR119W \| YER008C \| YNL068C \| YER165W \| YGR092W \| YIL106W \| YDL028C \| YPR111W \| YJL060W \| YKL129C |
| complex_1901 | YBL105C \| YJL095W \| YOR231W \| YPL151C \| YBR065C \| YLR117C \| YMR213W \| YPR119W \| YER165W \| YKL025C \| YGL120C \| YKL173W \| YHR086W \| YMR001C \| YDR364C |
| complex_1900 | YBL045C \| YPR054W \| YPR191W \| YDL087C \| YDR235W \| YHR086W \| YIL061C \| YKL173W \| YLR147C \| YML046W \| YDR432W \| YFL029C \| YHR030C \| YJL095W \| YPL140C |
| complex_3198 | YAL005C \| YNL007C \| YPL106C \| YCL011C \| YIL035C \| YIL033C \| YJL164C \| YKL166C \| YMR022W \| YLL039C \| YLR216C \| YLR347C \| YNL027W \| YNL209W \| YPL203W |
| complex_3196 | YAL032C \| YLR117C \| YMR001C \| YMR213W \| YBL105C \| YJL095W \| YPR119W \| YBR065C \| YCL011C \| YGL120C \| YNL189W \| YKL025C \| YPL151C \| YGL238W \| YOR231W |
| complex_3194 | YBL105C \| YGR232W \| YJL095W \| YOR231W \| YPL151C \| YDL007W \| YGL004C \| YDL029W \| YDR394W \| YOR259C \| YGL048C \| YKL145W \| YOR117W \| YHR030C \| YPL140C |
| complex_3193 | YDR188W \| YDR212W \| YGL137W \| YNL317W \| YOR212W \| YGL116W \| YGR092W \| YJL106W \| YJL164C \| YKL166C \| YIL033C \| YIL142W \| YJR064W \| YNL027W \| YPL203W |
| complex_3192 | YBR160W \| YGL003C \| YBR169C \| YDR477W \| YFL029C \| YGL011C \| YML092C \| YMR314W \| YOL038W \| YOR362C \| YDL108W \| YDR471W \| YDL084W \| YKL139W \| YMR308C |
| complex_3190 | YBR160W \| YGL003C \| YGR238C \| YHR158C \| YIL046W \| YJL187C \| YNL298W \| YPL115C \| YPR119W \| YPR120C \| YGR092W \| YHL035C \| YIL142W \| YLR096W \| YMR001C |
| complex_3189 | YAL005C \| YHR030C \| YML092C \| YBL105C \| YER165W \| YJL095W \| YOR231W \| YPL151C \| YPR119W \| YDL029W \| YPL140C \| YMR109W \| YKL129C \| YNL161W \| YNL007C |
| complex_3188 | YBR133C \| YER165W \| YHL007C \| YJL187C \| YBR160W \| YCL024W \| YDR054C \| YLR362W \| YGR152C \| YAL041W \| YOR127W \| YIL046W \| YKL129C \| YLR229C \| YNL298W |
| complex_3185 | YBR160W \| YGR092W \| YDL007W \| YER122C \| YFR010W \| YLR424W \| YOR259C \| YDL145C \| YGL137W \| YOR117W \| YDR394W \| YGL048C \| YKL145W \| YLL034C \| YNL250W |
| complex_3183 | YBL105C \| YBR160W \| YOL100W \| YOR231W \| YPL151C \| YPR119W \| YJL187C \| YNL102W \| YCR065W \| YER165W \| YJL060W \| YJL095W \| YKL129C \| YMR001C \| YNL298W |
| complex_3182 | YBL105C \| YBR160W \| YER165W \| YJL095W \| YOL100W \| YOR231W \| YPR119W \| YPR120C \| YGL003C \| YCR065W \| YGR108W \| YJL060W \| YKL025C \| YKL129C \| YPL119C |
| complex_2098 | YAL041W \| YOR212W \| YBR160W \| YER008C \| YJL187C \| YNL298W \| YOR127W \| YDR379W \| YGR152C \| YLR229C \| YPR119W \| YMR304W \| YHL007C \| YLR131C \| YNL161W |
| complex_2097 | YBR160W \| YCR065W \| YDL155W \| YDR146C \| YER129W \| YGR092W \| YHR018C \| YIL106W \| YKL129C \| YPR119W \| YPL042C \| YDR477W \| YAR019C \| YKR036C \| YNL209W |
| complex_2095 | YBR160W \| YKL129C \| YLR131C \| YNL161W \| YDL155W \| YHR018C \| YLR310C \| YDR216W \| YDL029W \| YNL007C \| YEL030W \| YFL034C-B \| YHL007C \| YHR030C \| YHR102W |
| complex_2092 | YDL087C \| YKL173W \| YDL208W \| YDR283C \| YMR001C \| YGR092W \| YHR030C \| YDL029W \| YJL095W \| YPR191W \| YHR086W \| YLR147C \| YNL147W \| YPL140C \| YPR178W |

| | |
|---|---|
| complex_2091 | YAL005C \| YBL075C \| YLL024C \| YLR310C \| YNL064C \| YOR027W \| YOR388C \| YPL240C \| YPR119W \| YJL128C \| YLR362W \| YDR146C \| YDL108W \| YHR018C \| YLR113W |
| complex_2090 | YBR065C \| YLR117C \| YLR424W \| YMR001C \| YDL208W \| YKL173W \| YDR473C \| YER029C \| YGL120C \| YGR270W \| YHR086W \| YDR283C \| YPL031C \| YPL151C \| YPR178W |
| complex_3178 | YAL005C \| YLL016W \| YLR310C \| YNL064C \| YOR027W \| YPL106C \| YBL016W \| YBL075C \| YDR477W \| YGR092W \| YJL005W \| YLL024C \| YOR101W \| YNL098C \| YNL209W |
| complex_3172 | YAL032C \| YBL105C \| YKL173W \| YLR117C \| YMR001C \| YMR213W \| YBR160W \| YJL187C \| YKR095W \| YCL011C \| YER013W \| YNL189W \| YHR086W \| YGL238W \| YPL151C |
| complex_3171 | YBR160W \| YGL003C \| YCR065W \| YPL152W \| YPR119W \| YDL155W \| YNL068C \| YDR146C \| YGR092W \| YIL106W \| YDL028C \| YPR111W \| YJL060W \| YKR095W \| YKL129C |
| complex_3170 | YBR160W \| YDL155W \| YER008C \| YGL003C \| YGR092W \| YJL060W \| YJL187C \| YMR001C \| YNL298W \| YPR119W \| YPR120C \| YDL145C \| YMR304W \| YHL035C \| YIL046W |
| complex_2089 | YAR019C \| YGR092W \| YBL075C \| YLR362W \| YNL298W \| YBR160W \| YDL155W \| YHL007C \| YHR018C \| YIL106W \| YKL129C \| YCR088W \| YDL029W \| YNL161W \| YPL042C |
| complex_2088 | YAR019C \| YGL003C \| YIL106W \| YLR210W \| YPR120C \| YBR135W \| YBR160W \| YDL155W \| YLR131C \| YNL068C \| YPR111W \| YPR119W \| YDR146C \| YGR092W \| YNL161W |
| complex_2087 | YBL105C \| YBR160W \| YPR119W \| YPR120C \| YPR165W \| YDR507C \| YER008C \| YGL003C \| YOR212W \| YPL209C \| YKR048C \| YMR139W \| YMR304W \| YJL187C \| YMR001C |
| complex_2083 | YBL105C \| YBR160W \| YGL003C \| YOL100W \| YPR119W \| YPR120C \| YAL019W \| YER008C \| YIL112W \| YNL068C \| YPL209C \| YPR175W \| YMR304W \| YJL187C \| YMR001C |
| complex_2082 | YBL105C \| YBR160W \| YGL003C \| YLR096W \| YCR065W \| YDR432W \| YER165W \| YPR119W \| YPR191W \| YHR030C \| YMR304W \| YJL060W \| YJL095W \| YPL140C \| YOL100W |
| complex_2080 | YAL032C \| YDR364C \| YKL173W \| YLR117C \| YMR001C \| YMR213W \| YDL074C \| YJL074C \| YFL008W \| YFR031C \| YHR086W \| YLR275W \| YLR086W \| YLR147C \| YPL151C |
| complex_3169 | YBL030C \| YDL029W \| YGL137W \| YBR198C \| YDR216W \| YOR212W \| YDR188W \| YDR142C \| YDR212W \| YIL142W \| YJR064W \| YNL317W \| YHL007C \| YNL161W \| YJL014W |
| complex_3167 | YBR160W \| YER008C \| YGL003C \| YIL046W \| YJL187C \| YPL209C \| YMR304W \| YHL007C \| YLR362W \| YJL095W \| YMR109W \| YKL129C \| YNL298W \| YPR119W \| YMR001C |
| complex_3165 | YBL045C \| YGR159C \| YIL061C \| YCL043C \| YHR086W \| YIR001C \| YDR432W \| YER165W \| YNL016W \| YOR204W \| YPR119W \| YGL049C \| YGR162W \| YJL033W \| YKL025C |
| complex_3161 | YAL005C \| YLR310C \| YBL016W \| YDL159W \| YBL075C \| YLR362W \| YPR119W \| YGR040W \| YJL128C \| YGR159C \| YOR204W \| YJR032W \| YLL024C \| YMR139W \| YNR031C |
| complex_3160 | YBR160W \| YCL024W \| YJL187C \| YNL298W \| YCR065W \| YDL145C \| YHR030C \| YDR054C \| YKL173W \| YPR191W \| YHR086W \| YPR119W \| YIL046W \| YLR147C \| YMR001C |
| complex_2078 | YBL105C \| YOR231W \| YBR160W \| YCR065W \| YGL003C \| YLR096W \| YDR432W \| YER165W \| YPR119W \| YPR191W \| YJL095W \| YHR030C \| YPR054W \| YJL060W \| YOL100W |
| complex_2077 | YAR019C \| YJR059W \| YBR160W \| YCR065W \| YDL155W \| YGR092W \| YKL129C \| YLR131C \| YNL068C \| YPR111W \| YPR119W \| YDR146C \| YIL106W \| YNL161W \| YDL029W |

| | |
|---|---|
| complex_2076 | YBL105C \| YBR160W \| YER165W \| YOL100W \| YPR119W \| YPR120C \| YER008C \| YGL003C \| YJL187C \| YMR001C \| YBR233W \| YDL025C \| YMR304W \| YGR159C \| YHL035C |
| complex_2074 | YBL016W \| YLR310C \| YLR362W \| YMR139W \| YBL075C \| YNL298W \| YBR160W \| YCR065W \| YHR018C \| YJL187C \| YKL129C \| YPR119W \| YBR200W \| YDR507C \| YHL007C |
| complex_3158 | YBR160W \| YBR200W \| YGL003C \| YJL187C \| YKL129C \| YMR001C \| YNL068C \| YNL102W \| YNL298W \| YNR047W \| YPR119W \| YPR120C \| YDR507C \| YMR139W \| YHL035C |
| complex_3157 | YAL032C \| YBL105C \| YBR118W \| YBL045C \| YKL139W \| YCL011C \| YDL084W \| YDR432W \| YNL112W \| YPR119W \| YER165W \| YGL173C \| YGR159C \| YOR204W |
| complex_3156 | YDR212W \| YFL033C \| YGL116W \| YHR135C \| YIL142W \| YJL164C \| YNL154C \| YDR264C \| YFR024C-A \| YPL203W \| YIL033C \| YNL027W \| YJL138C \| YNL093W \| YKL166C |
| complex_3155 | YAL005C \| YLR113W \| YOR388C \| YBR160W \| YCR065W \| YDL108W \| YDL155W \| YDR146C \| YLR131C \| YPR111W \| YPR119W \| YHR018C \| YGR092W \| YIL106W \| YNL161W |
| complex_3153 | YAR019C \| YHR169W \| YBL099W \| YJR121W \| YNL315C \| YBR160W \| YCR065W \| YDL155W \| YGR092W \| YKL129C \| YNL102W \| YIL106W \| YPR111W \| YPR119W \| YMR001C |
| complex_3152 | YAR019C \| YGR092W \| YNL229C \| YBR160W \| YCR065W \| YDL155W \| YKL129C \| YPR111W \| YDL185W \| YKL048C \| YDL029W \| YHL007C \| YIL106W \| YNL161W \| YPR119W |
| complex_2067 | YBR160W \| YCR065W \| YDL155W \| YGL003C \| YGR092W \| YJL187C \| YKL129C \| YMR001C \| YNL068C \| YNL102W \| YNL298W \| YPR119W \| YDR054C \| YIL046W \| YIL106W |
| complex_2060 | YBR160W \| YER008C \| YGL003C \| YGR092W \| YJL187C \| YNL068C \| YNL102W \| YNL298W \| YPL209C \| YPR119W \| YPR120C \| YDL145C \| YMR304W \| YIL046W \| YMR001C |
| complex_3149 | YBR160W \| YDL155W \| YGR092W \| YLR113W \| YNL068C \| YCR065W \| YPR119W \| YDL108W \| YGR058W \| YDR146C \| YLR131C \| YIL106W \| YNL161W \| YPR111W \| YKL129C |
| complex_3148 | YDL087C \| YKL173W \| YDL208W \| YPR178W \| YDR283C \| YER013W \| YLR117C \| YMR001C \| YER029C \| YJR064W \| YPL151C \| YGL120C \| YHR086W \| YLR147C \| YNL147W |
| complex_3147 | YAR019C \| YBR160W \| YGR092W \| YNL102W \| YCR065W \| YDL025C \| YGL076C \| YPR119W \| YDL155W \| YDR146C \| YIL106W \| YNL068C \| YPR111W \| YKL129C \| YMR001C |
| complex_3146 | YAL003W \| YKL081W \| YBL105C \| YPR054W \| YDL029W \| YHR030C \| YER165W \| YHR082C \| YLR096W \| YPR191W \| YIL046W \| YML057W \| YJL095W \| YOR231W \| YPL140C |
| complex_3143 | YBR160W \| YCR065W \| YKL129C \| YNL298W \| YPL209C \| YBR200W \| YHL007C \| YLR362W \| YPR119W \| YDR356W \| YEL026W \| YIL149C \| YJL187C \| YKR095W \| YOR195W |
| complex_3142 | YBR160W \| YCR065W \| YER129W \| YKL129C \| YNL068C \| YPR111W \| YPR119W \| YPR120C \| YDR477W \| YGR092W \| YHR018C \| YIL106W \| YHL035C \| YNL209W \| YDL028C |
| complex_3141 | YAL005C \| YDL229W \| YDR477W \| YLL024C \| YNL007C \| YNL209W \| YNL308C \| YPL106C \| YNL175C \| YER028C \| YER129W \| YIL035C \| YPL131W \| YHR102W \| YNL161W |
| complex_3140 | YAR019C \| YBR160W \| YGR092W \| YGL003C \| YCR034W \| YNR047W \| YCR065W \| YCR091W \| YDL155W \| YIL106W \| YNL068C \| YPR111W \| YPR119W \| YKL129C \| YMR001C |
| complex_2059 | YBR160W \| YCR065W \| YGL003C \| YHR158C \| YIL131C \| YKL129C \| YMR001C \| YNL102W \| YPR119W \| YDL155W \| YGR092W \| YDR146C \| YHL035C \| YIL106W \| YJL187C |

| | |
|---|---|
| complex_2055 | YAL040C \| YBR160W \| YCR065W \| YHR018C \| YJL013C \| YKL129C \| YNL064C \| YPR119W \| YBL075C \| YLR362W \| YNL298W \| YBR200W \| YHL007C \| YHL035C \| YJL187C |
| complex_2054 | YBL045C \| YBR160W \| YIL131C \| YBL105C \| YDL195W \| YGL019W \| YIL035C \| YER165W \| YPL203W \| YOR039W \| YIL033C \| YMR022W \| YJL164C \| YKL166C \| YOR061W |
| complex_2052 | YBR160W \| YDR356W \| YER008C \| YHL007C \| YJL187C \| YLR212C \| YNL298W \| YOR127W \| YPR119W \| YGR152C \| YAL041W \| YLR229C \| YIL046W \| YLR362W \| YNL007C |
| complex_2051 | YBL016W \| YDL159W \| YLR210W \| YLR362W \| YGR040W \| YDR188W \| YIL142W \| YKR036C \| YLL011W \| YNL317W \| YOR212W \| YDR212W \| YBR198C \| YJR064W \| YOR153W |
| complex_2050 | YAL005C \| YBR155W \| YDR477W \| YER129W \| YJR032W \| YLL024C \| YLR310C \| YLR362W \| YBL075C \| YDL229W \| YPL240C \| YGL035C \| YDR194C \| YLR347C \| YPL106C |
| complex_3137 | YBR160W \| YNL102W \| YCR065W \| YDL155W \| YGR092W \| YKL129C \| YHL035C \| YLR210W \| YPR119W \| YIL106W \| YNL068C \| YPR111W \| YJL187C \| YMR001C \| YNL298W |
| complex_3136 | YDR188W \| YBR198C \| YDR142C \| YFL033C \| YIL035C \| YJL164C \| YLR196W \| YNL317W \| YOR212W \| YDR212W \| YIL142W \| YFR024C-A \| YNL027W \| YJL014W \| YJR064W |
| complex_3134 | YDL029W \| YOR212W \| YDR216W \| YLR131C \| YNL007C \| YNL161W \| YFL034C-B \| YHL007C \| YKL129C \| YHR030C \| YHR102W \| YJL095W \| YPL140C \| YMR109W \| YJR064W |
| complex_3131 | YBL105C \| YOR231W \| YBR109C \| YJR065C \| YKL129C \| YNR035C \| YOR358W \| YDR432W \| YER165W \| YGL173C \| YKL139W \| YPR119W \| YJL095W \| YDL029W \| YMR109W |
| complex_3130 | YBR155W \| YAL005C \| YDL229W \| YDR356W \| YDR477W \| YPL106C \| YLL024C \| YNL209W \| YEL030W \| YER081W \| YIL095W \| YPL031C \| YJR104C \| YCR008W \| YOR185C |
| complex_2049 | YBL105C \| YER165W \| YNL317W \| YPR119W \| YBR233W \| YDR432W \| YGR159C \| YIR001C \| YOL123W \| YOR204W \| YGL044C \| YGL049C \| YNL016W \| YGR162W \| YJL033W |
| complex_2046 | YBR160W \| YPR141C \| YCR065W \| YDL028C \| YDR356W \| YDL155W \| YFL037W \| YGR092W \| YIL106W \| YNL068C \| YPR111W \| YPR119W \| YKL129C \| YML085C \| YPR120C |
| complex_2045 | YBL045C \| YGR159C \| YMR139W \| YBL105C \| YBR160W \| YOL100W \| YDR507C \| YER008C \| YGL003C \| YDR432W \| YER165W \| YOR204W \| YPR119W \| YJL060W \| YJR059W |
| complex_2044 | YBR160W \| YGL003C \| YNL102W \| YCR065W \| YDL025C \| YPL131W \| YPR119W \| YDL155W \| YGR092W \| YKL129C \| YDR146C \| YNL288W \| YIL106W \| YPR111W \| YMR001C |
| complex_2043 | YBL075C \| YHL007C \| YBR109C \| YKL129C \| YKL210W \| YBR160W \| YJL187C \| YBR200W \| YNL298W \| YPR119W \| YCR065W \| YHR018C \| YJR059W \| YLR362W \| YPL119C |
| complex_2041 | YBL045C \| YCR065W \| YNR047W \| YBL105C \| YOL100W \| YBR160W \| YJR059W \| YKL048C \| YKL116C \| YKL129C \| YPR119W \| YER165W \| YGR159C \| YJL095W \| YOR231W |
| complex_2040 | YDL029W \| YOR212W \| YDL097C \| YDR142C \| YFR004W \| YOR261C \| YDL147W \| YDR188W \| YBR198C \| YDR523C \| YGL137W \| YNL317W \| YDR212W \| YIL142W \| YJR064W |
| complex_3128 | YDL007W \| YEL037C \| YDL160C \| YER081W \| YGL137W \| YKL078W \| YDR394W \| YGL048C \| YGR092W \| YKL145W \| YOR117W \| YOR259C \| YPL096W \| YPL240C \| YOR027W |
| complex_3125 | YGL137W \| YFL033C \| YKL166C \| YLR216C \| YHR135C \| YIL033C \| YNL027W \| YMR022W \| YIL035C \| YLL039C \| YJL164C \| YNL093W \| YKR014C \| YOR089C \| YPL203W |

| | |
|---|---|
| complex_3122 | YBR160W \| YDL108W \| YER008C \| YGL003C \| YPL209C \| YPR119W \| YPR120C \| YLR113W \| YMR304W \| YFL029C \| YHL035C \| YJL060W \| YJL187C \| YKR048C \| YMR001C |
| complex_3121 | YBR160W \| YCR065W \| YKL129C \| YLR131C \| YNL161W \| YDL155W \| YDR146C \| YPR119W \| YFL034C-B \| YHR102W \| YNL068C \| YGR092W \| YHL007C \| YIL106W \| YDL029W |
| complex_3120 | YBL105C \| YJL095W \| YOL100W \| YPR120C \| YBR160W \| YER008C \| YGL003C \| YHL007C \| YDR212W \| YOR212W \| YMR304W \| YLR229C \| YNL161W \| YOR231W \| YPR165W |
| complex_2039 | YBR160W \| YJL187C \| YKR095W \| YLR086W \| YLR314C \| YMR001C \| YNL189W \| YNL298W \| YCL024W \| YCR002C \| YDR507C \| YHR107C \| YJR076C \| YDL225W \| YER172C |
| complex_90 | YAL032C \| YDR364C \| YKL173W \| YLL036C \| YLR117C \| YLR424W \| YMR001C \| YMR213W \| YBR065C \| YDL030W \| YER013W \| YGL120C \| YPL151C \| YLR147C \| YMR240C |
| complex_2038 | YAL017W \| YKL166C \| YPR160W \| YBL045C \| YPL204W \| YDR432W \| YER133W \| YER165W \| YGL173C \| YGR159C \| YMR216C \| YOR204W \| YPR119W \| YGL137W \| YGR092W |
| complex_2037 | YBR160W \| YDL145C \| YGR092W \| YIL046W \| YJL187C \| YMR001C \| YNL298W \| YCL024W \| YCR002C \| YHR107C \| YDR054C \| YDR328C \| YGL137W \| YJR076C \| YLR314C |
| complex_2399 | YBL045C \| YOR231W \| YPR054W \| YPR191W \| YBL105C \| YER165W \| YGL096W \| YPL140C \| YHL002W \| YHR108W \| YMR109W \| YHR030C \| YDL029W \| YJL095W \| YKL213C |
| complex_2035 | YBR066C \| YDL155W \| YDR477W \| YER028C \| YPR119W \| YBR160W \| YDR146C \| YER129W \| YGL003C \| YGL035C \| YNL027W \| YDR216W \| YMR304W \| YLR131C |
| complex_2396 | YAL021C \| YDR188W \| YGR092W \| YKR036C \| YPL042C \| YBR198C \| YDR212W \| YIL142W \| YJL014W \| YJR064W \| YGL116W \| YGL137W \| YLL011W \| YNL317W \| YOR212W |
| complex_2393 | YAL032C \| YBL105C \| YKL173W \| YLR117C \| YMR213W \| YBR160W \| YKR095W \| YER013W \| YMR001C \| YHR086W \| YIL149C \| YPL151C \| YPR154W \| YNL189W \| YGL238W |
| complex_2390 | YAL029C \| YBR109C \| YFL005W \| YFL039C \| YGL106W \| YOR035C \| YBL105C \| YBR160W \| YJL095W \| YOR231W \| YGL240W \| YHR023W \| YOR326W \| YKL129C \| YMR109W |
| complex_3119 | YBL105C \| YBR160W \| YOL100W \| YPR119W \| YPR120C \| YDL155W \| YGL003C \| YGR092W \| YIL106W \| YNL068C \| YNL102W \| YHL035C \| YJL187C \| YMR001C \| YOR195W |
| complex_3118 | YCL043C \| YFL039C \| YHR086W \| YIR001C \| YDR432W \| YER165W \| YGR159C \| YJL033W \| YNL016W \| YOR204W \| YPR119W \| YGL049C \| YGR162W \| YKL025C \| YKR095W |
| complex_3117 | YBR160W \| YCR065W \| YDL155W \| YDR146C \| YGR092W \| YHL007C \| YIL106W \| YJR059W \| YKL129C \| YLR131C \| YNL068C \| YNR047W \| YPR111W \| YPR119W \| YNL161W |
| complex_87 | YDL007W \| YFR004W \| YDL025C \| YBR160W \| YDL097C \| YDR394W \| YGR262C \| YHR027C \| YOR117W \| YOR259C \| YOR261C \| YDL147W \| YGR092W \| YGL048C \| YKL145W |
| complex_3114 | YBL016W \| YGL137W \| YBR160W \| YLR210W \| YNL298W \| YPL115C \| YDL159W \| YGR040W \| YDR212W \| YIL142W \| YJL106W \| YLL011W \| YOR212W \| YHL007C \| YLR362W |
| complex_3112 | YCL011C \| YDL226C \| YDR432W \| YER165W \| YFL039C \| YGL120C \| YKL139W \| YDL084W \| YGR159C \| YNL004W \| YDL192W \| YDL229W \| YAL005C \| YDR477W \| YNL112W |
| complex_84 | YAL005C \| YBR085W \| YER081W \| YER107C \| YER165W \| YLL024C \| YLR310C \| YNL064C \| YNL209W \| YOR027W \| YBL075C \| YDR477W \| YER028C \| YPL106C \| YNL041C |

| | |
|---|---|
| complex_3473 | YDL134C \| YDR523C \| YHR135C \| YDL188C \| YGL137W \| YGR040W \| YKL166C \| YLR216C \| YLR310C \| YPR191W \| YHR030C \| YHR086W \| YIL033C \| YJL164C \| YPL203W |
| complex_3110 | YAR019C \| YGL003C \| YMR001C \| YBR160W \| YAL019W \| YDL155W \| YGR092W \| YIL106W \| YPR111W \| YPR119W \| YPR120C \| YGR159C \| YER165W \| YHL035C \| YJL060W |
| complex_3470 | YAL005C \| YDL101C \| YJR032W \| YLL024C \| YLR310C \| YOR027W \| YPL153C \| YBL075C \| YBR155W \| YPL240C \| YKL095W \| YMR190C \| YJR064W \| YLR347C \| YMR001C |
| complex_2029 | YBR160W \| YDL029W \| YCR065W \| YDL028C \| YDR356W \| YDL155W \| YPR119W \| YGR092W \| YIL106W \| YNL161W \| YNL298W \| YKL129C \| YLR131C \| YOR127W \| YPR111W |
| complex_2028 | YBR160W \| YGL003C \| YDL108W \| YDL155W \| YNL161W \| YDR146C \| YLR131C \| YPR119W \| YER165W \| YMR304W \| YGR092W \| YHL007C \| YIL106W \| YHR102W \| YJL060W |
| complex_2389 | YBR031W \| YBR160W \| YLR362W \| YOR127W \| YCR065W \| YJL187C \| YBR200W \| YPR119W \| YDR054C \| YGR152C \| YLR229C \| YHL007C \| YIL046W \| YKL129C \| YNL298W |
| complex_2024 | YBL045C \| YKL139W \| YNL016W \| YBL105C \| YDR432W \| YER165W \| YGL078C \| YGL173C \| YGR159C \| YHR086W \| YIR001C \| YOR204W \| YPR119W \| YGL049C \| YGR162W |
| complex_2386 | YBR160W \| YCR065W \| YIL046W \| YJL187C \| YKL129C \| YNL102W \| YNL298W \| YBR200W \| YAL041W \| YLR362W \| YPR119W \| YDR054C \| YHL007C \| YML092C \| YMR001C |
| complex_2023 | YBR160W \| YIL035C \| YNL068C \| YNL112W \| YDL195W \| YGL019W \| YIL118W \| YLR399C \| YOL004W \| YOR039W \| YOR061W \| YIL106W \| YIL131C \| YOR326W \| YOL145C |
| complex_2385 | YBL075C \| YLR362W \| YNL298W \| YBR135W \| YBR160W \| YJL187C \| YBR200W \| YPR119W \| YGR108W \| YNL068C \| YHL007C \| YKL129C \| YHL035C \| YHR018C \| YPR120C |
| complex_2022 | YAL032C \| YBL105C \| YKL173W \| YLR117C \| YMR001C \| YMR213W \| YCL024W \| YJL187C \| YPR154W \| YER013W \| YHR086W \| YGL238W \| YMR240C \| YPL151C \| YNL189W |
| complex_2021 | YAL005C \| YBL075C \| YJR032W \| YLR293C \| YLR310C \| YNL064C \| YOR027W \| YBR155W \| YHR193C \| YDR252W \| YPR111W \| YPL037C \| YLL024C \| YLR347C \| YOR185C |
| complex_2381 | YBR160W \| YCR065W \| YDR364C \| YGL003C \| YJL187C \| YKL129C \| YNL102W \| YNL298W \| YBR200W \| YAL041W \| YLR362W \| YPR119W \| YHL007C \| YML092C \| YMR001C |
| complex_2380 | YDL029W \| YOR212W \| YDL097C \| YDR142C \| YFR004W \| YHR030C \| YOR261C \| YDL147W \| YDR523C \| YDR188W \| YHR102W \| YNL161W \| YNL317W \| YJL014W \| YJR064W |
| complex_1299 | YGR091W \| YGR083C \| YLR275W \| YJL124C \| YER146W \| YLR147C \| YPR182W \| YJR022W \| YPR178W \| YNL147W \| YOR159C \| YNL189W \| YIL106W \| YOR310C |
| complex_1296 | YBL075C \| YHL007C \| YBR160W \| YGR238C \| YJL187C \| YBR200W \| YNL298W \| YPR119W \| YCR065W \| YDR146C \| YKL129C \| YHR018C \| YHR158C \| YLR096W \| YLR362W |
| complex_3107 | YBL105C \| YBR160W \| YDL145C \| YIL046W \| YJL095W \| YOL100W \| YPR119W \| YER008C \| YJL187C \| YMR001C \| YNL298W \| YMR304W \| YER165W \| YKL129C \| YPR165W |
| complex_3468 | YDL007W \| YGL004C \| YDL134C \| YDR394W \| YDR523C \| YGL048C \| YHR030C \| YKL145W \| YOR014W \| YOR117W \| YOR259C \| YPR040W \| YDL188C \| YDL029W \| YGR232W |
| complex_3106 | YDL087C \| YHR086W \| YIL061C \| YKL012W \| YKL173W \| YDR416W \| YLR117C \| YMR001C \| YPL151C \| YER029C \| YGL120C \| YHR064C \| YIR005W \| YNL245C \| YLR147C |

| | |
|---|---|
| complex_3466 | YBR160W \| YGL003C \| YGR092W \| YJL187C \| YMR001C \| YNL317W \| YPR120C \| YDL108W \| YDR212W \| YGL137W \| YIL142W \| YJL106W \| YKR036C \| YFL029C \| YPR054W |
| complex_3103 | YGR095C \| YBR109C \| YGR195W \| YIL106W \| YNL189W \| YJL124C \| YER146W \| YLR147C \| YPR182W \| YJR022W \| YLR275W \| YNL147W \| YOR159C \| YOR310C \| YGR083C |
| complex_3464 | YCL011C \| YDL084W \| YDR432W \| YFL018C \| YKL139W \| YNL112W \| YPR119W \| YPR191W \| YDL175C \| YIL079C \| YER165W \| YGR159C \| YMR216C \| YOL123W \| YOR204W |
| complex_3102 | YDL029W \| YPR178W \| YDR394W \| YGL137W \| YHR030C \| YML057W \| YKL173W \| YLR147C \| YHR086W \| YJL124C \| YPR182W \| YJR022W \| YLR433C \| YKL190W \| YNL147W |
| complex_3462 | YBL105C \| YDL029W \| YJL095W \| YBR160W \| YCR065W \| YKL129C \| YER165W \| YPL140C \| YPR119W \| YHR030C \| YIL106W \| YHR082C \| YMR109W \| YOL100W \| YOR231W |
| complex_71 | YDL097C \| YDL007W \| YDR394W \| YEL037C \| YER012W \| YGL048C \| YHR027C \| YKL145W \| YOR117W \| YOR259C \| YOR261C \| YDL147W \| YPL096W \| YBR198C \| YNL016W |
| complex_2018 | YBL045C \| YCR065W \| YLR113W \| YPR054W \| YBL105C \| YBR160W \| YNL102W \| YDL108W \| YER165W \| YPR119W \| YFL029C \| YHR030C \| YJL095W \| YKL129C \| YOL100W |
| complex_2017 | YBR160W \| YDL155W \| YER008C \| YGL003C \| YGR092W \| YJL187C \| YNL068C \| YNL298W \| YPL209C \| YPR119W \| YPR120C \| YMR304W \| YIL046W \| YHL035C \| YMR001C |
| complex_2016 | YAL005C \| YER081W \| YER165W \| YJL095W \| YNL209W \| YOR027W \| YOR254C \| YPL106C \| YBL105C \| YHR030C \| YGL116W \| YKL073W \| YJL034W \| YOR231W \| YPL140C |
| complex_2378 | YBR160W \| YNL102W \| YCR065W \| YKL139W \| YDL108W \| YFL029C \| YLR113W \| YDR432W \| YER165W \| YPR119W \| YPR191W \| YDR477W \| YER129W \| YHR030C \| YPR054W |
| complex_2015 | YBR160W \| YGR092W \| YJL187C \| YCL024W \| YDL132W \| YFL009W \| YIL046W \| YDL155W \| YNL161W \| YOR326W \| YDR054C \| YMR304W \| YIL106W \| YOL133W \| YOR319W |
| complex_2377 | YAR019C \| YGR092W \| YBR160W \| YCR065W \| YKL129C \| YLR131C \| YDL155W \| YDR146C \| YNL288W \| YPR119W \| YDR285W \| YDL029W \| YHL007C \| YIL106W \| YNL161W |
| complex_2013 | YBR160W \| YDL155W \| YIL131C \| YKL129C \| YPR054W \| YDL029W \| YHR030C \| YMR109W \| YNL161W \| YHL007C \| YJL095W \| YHR086W \| YHR102W \| YPR191W \| YPL140C |
| complex_2375 | YBR160W \| YCR065W \| YJL187C \| YKL129C \| YMR001C \| YNL102W \| YNL298W \| YDL074C \| YJL074C \| YDL208W \| YFL008W \| YFR031C \| YLR086W \| YPR119W \| YOR195W |
| complex_2012 | YAL005C \| YBL075C \| YER012W \| YLL024C \| YLR310C \| YOR027W \| YDL017W \| YGL137W \| YER103W \| YGL011C \| YJL001W \| YML092C \| YOR362C \| YPR103W \| YPR054W |
| complex_2374 | YBL105C \| YJL095W \| YBR160W \| YCR065W \| YGL003C \| YGR108W \| YKL129C \| YEL002C \| YOL100W \| YER165W \| YGR159C \| YPR119W \| YJL060W \| YKL025C \| YOR231W |
| complex_2011 | YBL045C \| YCR065W \| YPR054W \| YPR191W \| YBR160W \| YDL108W \| YFL029C \| YLR096W \| YPR119W \| YDR432W \| YER165W \| YGR159C \| YHL036W \| YGL137W \| YHR030C |
| complex_2010 | YBL105C \| YJL095W \| YOL100W \| YOR231W \| YBR160W \| YER129W \| YKL129C \| YPR119W \| YDR432W \| YER165W \| YGL173C \| YKL139W \| YOR204W \| YGR159C \| YMR109W |
| complex_2370 | YBR160W \| YGL003C \| YMR001C \| YPR120C \| YDR188W \| YGL137W \| YLL011W \| YNL317W \| YOR212W \| YPL140C \| YDR212W \| YJL106W \| YGR092W \| YIL142W \| YJR064W |

| | |
|---|---|
| complex_1289 | YAL005C \| YBL075C \| YBR155W \| YJR032W \| YLR310C \| YOR027W \| YBR017C \| YLR293C \| YOR185C \| YDL229W \| YDR477W \| YLL024C \| YPL106C \| YPL240C \| YMR308C |
| complex_1288 | YAL005C \| YER165W \| YLR310C \| YNL064C \| YPL106C \| YBL075C \| YPR119W \| YBR160W \| YDR146C \| YDL108W \| YDL229W \| YDR172W \| YNL027W \| YGL254W \| YLL024C |
| complex_1287 | YDR188W \| YDR212W \| YGL137W \| YKR036C \| YNL317W \| YOR212W \| YGL116W \| YGR092W \| YJL106W \| YJL164C \| YKL166C \| YPL203W \| YOR317W \| YIL142W \| YJR064W |
| complex_1286 | YDR364C \| YKL095W \| YMR213W \| YDR473C \| YKL173W \| YER013W \| YER003C \| YLR117C \| YMR001C \| YFL017W-A \| YPL151C \| YPR178W \| YGL120C \| YMR240C \| YNL189W |
| complex_1282 | YAL005C \| YDR477W \| YER081W \| YLL024C \| YLR310C \| YNL209W \| YPL106C \| YDL029W \| YDR216W \| YDL229W \| YGL158W \| YDR194C \| YGL035C \| YER129W \| YGL179C |
| complex_1281 | YBR160W \| YIL046W \| YJL187C \| YMR001C \| YNL298W \| YCL024W \| YCR002C \| YDR054C \| YDR507C \| YHR107C \| YKL101W \| YAL041W \| YJR076C \| YLR314C \| YNL007C |
| complex_3459 | YAL029C \| YBR031W \| YDR507C \| YFR027W \| YMR139W \| YBR160W \| YCR065W \| YPR119W \| YPL131W \| YHR098C \| YJR059W \| YIL106W \| YNL068C \| YPR111W \| YNL027W |
| complex_3456 | YCL043C \| YFL039C \| YIR001C \| YDR432W \| YER165W \| YGR159C \| YHR086W \| YNL016W \| YOR204W \| YPR119W \| YNL138W \| YGL049C \| YKR059W \| YGR162W \| YKR095W |
| complex_3455 | YDR212W \| YIL142W \| YGL116W \| YJL014W \| YJR064W \| YGL137W \| YDR188W \| YGR092W \| YKR036C \| YJL164C \| YPL203W \| YKL166C \| YLL011W \| YNL317W \| YOR212W |
| complex_3453 | YAL029C \| YBR081C \| YFL039C \| YGL106W \| YHR023W \| YLR249W \| YGR252W \| YBR198C \| YDR145W \| YDR216W \| YGR274C \| YDR448W \| YHR079C \| YLR291C \| YGR083C |
| complex_3452 | YBR160W \| YBL105C \| YCR065W \| YDR356W \| YIL112W \| YLR096W \| YPR119W \| YBR109C \| YKL190W \| YJL095W \| YMR109W \| YKL129C \| YLR433C \| YML057W \| YOR195W |
| complex_62 | YAL005C \| YNL308C \| YBL075C \| YER107C \| YLR310C \| YNL064C \| YNL175C \| YOR027W \| YDL229W \| YDR477W \| YNL209W \| YER028C \| YLL024C \| YGL070C \| YPL106C |
| complex_2009 | YBL105C \| YBR160W \| YHR018C \| YJL095W \| YPR119W \| YBR109C \| YKL210W \| YMR109W \| YLR096W \| YKL073W \| YHR030C \| YDL029W \| YJL138C \| YKL129C \| YPL140C |
| complex_2008 | YBL045C \| YKL139W \| YNL016W \| YBL105C \| YOL100W \| YDR432W \| YER165W \| YGL078C \| YGL173C \| YGR159C \| YIR001C \| YOL123W \| YOR204W \| YPR119W \| YGR162W |
| complex_2007 | YBL045C \| YJL164C \| YPR054W \| YPR191W \| YDR490C \| YER165W \| YKL166C \| YGL137W \| YHR030C \| YLR216C \| YHR135C \| YIL033C \| YMR022W \| YNR031C \| YPL203W |
| complex_2369 | YBR160W \| YCR065W \| YDL155W \| YGR092W \| YJL187C \| YDR054C \| YIL046W \| YIL106W \| YNL298W \| YPR111W \| YPR119W \| YDL029W \| YJL194W \| YKL129C \| YMR109W |
| complex_2006 | YER029C \| YMR001C \| YPL151C \| YGR092W \| YDR283C \| YHR030C \| YDL029W \| YJL095W \| YKL173W \| YPR054W \| YPR191W \| YHR086W \| YLR147C \| YPL140C \| YPR178W |
| complex_2366 | YBR160W \| YAL019W \| YER008C \| YGL003C \| YGR092W \| YGR238C \| YHR158C \| YIL112W \| YJL187C \| YLR096W \| YMR001C \| YDL155W \| YDR146C \| YLR131C \| YPR119W |
| complex_2365 | YBL105C \| YJL095W \| YBR160W \| YER008C \| YGL003C \| YMR001C \| YOL100W \| YMR304W \| YER165W \| YPR119W \| YJL060W \| YJL187C \| YKL101W \| YKL129C \| YNL016W |

| | |
|---|---|
| complex_2000 | YBR160W \| YGR092W \| YKL048C \| YLR314C \| YMR001C \| YNL298W \| YCL024W \| YCR002C \| YDR507C \| YHR107C \| YJR076C \| YDL225W \| YDR218C \| YKL101W \| YNL307C |
| complex_2361 | YBL105C \| YOL100W \| YPL151C \| YPR119W \| YPR120C \| YBR065C \| YDL155W \| YBR160W \| YGL003C \| YGR092W \| YIL106W \| YJL187C \| YNL068C \| YMR001C \| YPR111W |
| complex_1279 | YBR160W \| YDR507C \| YGR092W \| YJL187C \| YKL048C \| YMR001C \| YNL298W \| YCL024W \| YCR002C \| YDL225W \| YHR107C \| YLR314C \| YKL101W \| YJR076C \| YMR139W |
| complex_1278 | YBR034C \| YGR159C \| YIL079C \| YNL004W \| YOL123W \| YCL011C \| YDR432W \| YKL139W \| YNL112W \| YPR119W \| YPR191W \| YDL084W \| YDL175C \| YER165W \| YOR204W |
| complex_1277 | YDL195W \| YGL019W \| YIL131C \| YOR061W \| YER148W \| YFL039C \| YLR399C \| YOR039W \| YGL254W \| YIL118W \| YML010W \| YOL004W \| YJR014W \| YIL035C \| YJL140W |
| complex_1274 | YBL105C \| YJL095W \| YBR160W \| YER008C \| YGL003C \| YJL187C \| YMR001C \| YNL298W \| YMR304W \| YER165W \| YPR119W \| YGR092W \| YJL060W \| YKR095W \| YOL100W |
| complex_1271 | YDR075W \| YGR192C \| YKR036C \| YDR188W \| YDR142C \| YGL137W \| YIL142W \| YNL317W \| YOR212W \| YDR212W \| YBR198C \| YJL106W \| YGR092W \| YJL014W \| YJR064W |
| complex_1270 | YBL105C \| YDR283C \| YDL087C \| YKL173W \| YDL208W \| YPR178W \| YER029C \| YMR001C \| YHR030C \| YPR191W \| YHR086W \| YJL095W \| YLR147C \| YNL147W \| YPL151C |
| complex_3448 | YBR160W \| YER008C \| YGL003C \| YJL187C \| YJR059W \| YKL116C \| YLR210W \| YMR001C \| YPL209C \| YPR119W \| YMR304W \| YHL035C \| YIL046W \| YKR048C \| YNR047W |
| complex_398 | YAL005C \| YBR155W \| YDL229W \| YDR477W \| YER081W \| YLR310C \| YNL064C \| YNL308C \| YPL106C \| YBL075C \| YPL240C \| YGL070C \| YLL024C \| YPL042C \| YNL209W |
| complex_397 | YAL005C \| YBL075C \| YDL229W \| YDR477W \| YER165W \| YLR310C \| YNL025C \| YNL064C \| YNL209W \| YPL106C \| YBR085W \| YOR388C \| YPL042C \| YNL041C \| YER028C |
| complex_396 | YAL005C \| YDL229W \| YER081W \| YER165W \| YLR310C \| YNL025C \| YNL064C \| YNL209W \| YPR128C \| YBL075C \| YPL042C \| YDR477W \| YER028C \| YPL106C \| YNL041C |
| complex_3443 | YAL005C \| YNL209W \| YOR388C \| YBR160W \| YDL108W \| YDR146C \| YDR356W \| YLR113W \| YPR119W \| YDL229W \| YDR477W \| YLL024C \| YJL128C \| YPL106C \| YNL027W |
| complex_394 | YDL087C \| YKL173W \| YDL208W \| YPR178W \| YDR235W \| YPR182W \| YDR283C \| YER146W \| YHR086W \| YLR275W \| YML046W \| YIL061C \| YLR147C \| YNL147W \| YOR159C |
| complex_3441 | YBL105C \| YBR160W \| YPL151C \| YCR065W \| YDR054C \| YHR086W \| YKL173W \| YMR001C \| YNL298W \| YPR119W \| YIL046W \| YJL060W \| YJL187C \| YCL024W \| YLR117C |
| complex_51 | YDL007W \| YFR004W \| YDL097C \| YDR394W \| YGL048C \| YGR262C \| YHR027C \| YHR030C \| YKL145W \| YOR117W \| YOR259C \| YOR261C \| YDL147W \| YDL029W \| YJL008C |
| complex_392 | YAL005C \| YER081W \| YER165W \| YLL024C \| YLR310C \| YNL007C \| YNL209W \| YPR128C \| YDL229W \| YDR356W \| YIL149C \| YDR477W \| YER028C \| YPL106C \| YNL041C |
| complex_390 | YAL005C \| YBR085W \| YER165W \| YLR310C \| YNL064C \| YOR027W \| YOR388C \| YPL106C \| YBL075C \| YDR477W \| YER028C \| YLL024C \| YNL041C \| YPL042C \| YCR088W |
| complex_2356 | YBL105C \| YBR160W \| YER165W \| YGL003C \| YOL100W \| YPL151C \| YPR119W \| YER008C \| YJL187C \| YMR001C \| YBR233W \| YMR304W \| YGR159C \| YHL035C \| YPR165W |

| | |
|---|---|
| complex_2352 | YCR002C \| YDR507C \| YLR314C \| YDL029W \| YDR394W \| YHR030C \| YOR259C \| YDL225W \| YHR107C \| YNL064C \| YLR216C \| YJR076C \| YKL073W \| YOR027W \| YPR054W |
| complex_2351 | YBL045C \| YGR159C \| YBL105C \| YBR160W \| YER165W \| YPR119W \| YKR095W \| YDR432W \| YHR086W \| YIR001C \| YNL016W \| YOR204W \| YGL049C \| YGR162W \| YDR235W |
| complex_2350 | YAL005C \| YBL075C \| YLL024C \| YLR310C \| YBL016W \| YLR362W \| YDL159W \| YDR267C \| YDL229W \| YDR477W \| YPL106C \| YER028C \| YNL209W \| YGL158W \| YNL064C |
| complex_1269 | YAL005C \| YLR310C \| YAL021C \| YDR477W \| YGR092W \| YKR036C \| YOR027W \| YPL042C \| YAR019C \| YPL240C \| YBL075C \| YGR135W \| YNL025C \| YLL024C \| YNL064C |
| complex_1267 | YAL005C \| YBL075C \| YLL024C \| YLR310C \| YNL064C \| YPL240C \| YPR119W \| YAL029C \| YMR139W \| YBL016W \| YHR018C \| YLR362W \| YDR507C \| YDL159W \| YGR040W |
| complex_1263 | YAL005C \| YBL016W \| YBR155W \| YLL024C \| YLR310C \| YLR362W \| YNL064C \| YOR027W \| YDL159W \| YBL075C \| YGR040W \| YPL240C \| YHR018C \| YJR032W \| YPL106C |
| complex_1260 | YDL087C \| YKL173W \| YML049C \| YGL120C \| YKL095W \| YLR117C \| YLR424W \| YPL151C \| YGR092W \| YHR086W \| YDR364C \| YJR064W \| YMR001C \| YLR147C \| YNL147W |
| complex_3439 | YAL005C \| YDR172W \| YDR356W \| YER165W \| YNL209W \| YOL123W \| YBL105C \| YBR160W \| YLR096W \| YPR119W \| YDL229W \| YDR432W \| YGL044C \| YFR024C-A \| YPL106C |
| complex_389 | YAL005C \| YDR356W \| YER107C \| YER165W \| YLL024C \| YLR310C \| YNL007C \| YNL041C \| YNL209W \| YPL106C \| YPR128C \| YPR161C \| YDL229W \| YDR477W \| YER028C |
| complex_387 | YAL005C \| YDL229W \| YDR172W \| YDR477W \| YER081W \| YER165W \| YLL024C \| YLR310C \| YNL007C \| YNL041C \| YNL209W \| YPL106C \| YPR119W \| YPR128C \| YER028C |
| complex_386 | YAL005C \| YBL075C \| YBR085W \| YER107C \| YER165W \| YLL016W \| YLR310C \| YNL064C \| YOR027W \| YPL106C \| YDR477W \| YER028C \| YLL024C \| YNL041C \| YNL098C |
| complex_3434 | YBL045C \| YDR523C \| YPR054W \| YPR191W \| YDL029W \| YDL047W \| YDR283C \| YDR394W \| YOR259C \| YGL137W \| YHR030C \| YLR216C \| YKL173W \| YOR027W \| YPR178W |
| complex_384 | YAL005C \| YBR155W \| YDL229W \| YDR477W \| YGL070C \| YLL024C \| YLR310C \| YNL007C \| YNL209W \| YNL308C \| YOR185C \| YPL106C \| YEL030W \| YER107C \| YNL175C |
| complex_3431 | YBL045C \| YBR160W \| YCR065W \| YBL105C \| YCR091W \| YNR047W \| YDR432W \| YER165W \| YOL123W \| YPR119W \| YJR059W \| YGR108W \| YKL048C \| YNL068C \| YOL100W |
| complex_41 | YBL026W \| YER112W \| YER146W \| YDR378C \| YJL124C \| YJR022W \| YLR275W \| YNL147W \| YFR052W \| YKL173W \| YPR182W \| YPR178W \| YLR147C \| YMR314W \| YOR159C |
| complex_2348 | YBL045C \| YCR065W \| YNR047W \| YBL105C \| YOL100W \| YBR160W \| YDR507C \| YJR059W \| YER165W \| YGR159C \| YPR119W \| YJL095W \| YKL129C \| YMR139W \| YOR231W |
| complex_2347 | YBR160W \| YNL102W \| YCR065W \| YDL155W \| YGL240W \| YNL298W \| YGR092W \| YIL106W \| YPR111W \| YPR119W \| YJL187C \| YMR001C \| YKL129C \| YLR131C \| YOR127W |
| complex_2346 | YBR160W \| YER008C \| YGL003C \| YKL048C \| YPL209C \| YPR119W \| YPR120C \| YMR304W \| YER165W \| YHL035C \| YJL060W \| YJL187C \| YKR048C \| YMR001C \| YNL298W |
| complex_2345 | YBR160W \| YDL155W \| YER008C \| YGL003C \| YGR092W \| YIL106W \| YJL187C \| YLR131C \| YPR119W \| YPR120C \| YNL161W \| YMR304W \| YFL005W \| YKR048C \| YHR102W |

| | |
|---|---|
| complex_2344 | YBL045C \| YCR065W \| YOR231W \| YBL105C \| YJL095W \| YBR160W \| YGL003C \| YJL187C \| YMR001C \| YER165W \| YKL173W \| YPR119W \| YJL060W \| YOL100W \| YPL151C |
| complex_2342 | YBR094W \| YBR109C \| YOR061W \| YDL195W \| YGL019W \| YIL035C \| YIL131C \| YFR014C \| YHR018C \| YIL118W \| YLR399C \| YOL004W \| YOR039W \| YOL145C \| YOR326W |
| complex_2341 | YDR432W \| YER165W \| YGR159C \| YHR086W \| YIR001C \| YMR290C \| YNL016W \| YOR204W \| YPR119W \| YPR191W \| YGL049C \| YGR162W \| YHR030C \| YPR054W \| YLR096W |
| complex_3428 | YBL016W \| YGR040W \| YDL017W \| YLL039C \| YDL159W \| YDR358W \| YNR006W \| YGR159C \| YHL002W \| YER125W \| YLR310C \| YMR022W \| YLR362W \| YJL128C \| YNR031C |
| complex_3426 | YBR160W \| YGL003C \| YGL137W \| YGR092W \| YIL046W \| YMR001C \| YPL115C \| YPR120C \| YDR212W \| YIL142W \| YJL106W \| YKR036C \| YLL011W \| YJL187C \| YNL298W |
| complex_36 | YDL007W \| YDR394W \| YDL097C \| YEL037C \| YFR004W \| YGL048C \| YHR027C \| YOR117W \| YOR259C \| YOR261C \| YDL147W \| YGR092W \| YHR098C \| YIL007C \| YKL145W |
| complex_3425 | YAL041W \| YLR229C \| YOR212W \| YBR160W \| YHL007C \| YJL187C \| YLR210W \| YOR127W \| YPL115C \| YDL145C \| YGL137W \| YDR212W \| YGR040W \| YLR362W \| YNL298W |
| complex_375 | YDL007W \| YFR004W \| YDL097C \| YDR394W \| YGL048C \| YGR262C \| YKL145W \| YOR117W \| YOR259C \| YOR261C \| YDL147W \| YDL029W \| YDR523C \| YGR092W \| YJL008C |
| complex_374 | YAL005C \| YBR085W \| YER081W \| YER107C \| YIR005W \| YLR310C \| YNL064C \| YNL209W \| YOR027W \| YPL106C \| YBL075C \| YJL187C \| YMR001C \| YLL024C \| YNL041C |
| complex_3422 | YAL005C \| YER165W \| YLR310C \| YBL016W \| YDL159W \| YLR362W \| YBL075C \| YCR073C \| YNR031C \| YGR040W \| YIL033C \| YMR022W \| YJL128C \| YLL024C \| YNL027W |
| complex_3420 | YBL084C \| YBR160W \| YDR356W \| YLR127C \| YMR001C \| YNL298W \| YDR054C \| YGL003C \| YHL002W \| YNR006W \| YIL046W \| YLL039C \| YIL156W \| YJL187C \| YNL243W |
| complex_370 | YAL005C \| YBL075C \| YBR085W \| YDR477W \| YER081W \| YER165W \| YLL024C \| YLR310C \| YNL064C \| YNL209W \| YOR027W \| YJL128C \| YLR362W \| YNL041C \| YPL106C |
| complex_2338 | YDL155W \| YJL095W \| YKL129C \| YLR131C \| YNL161W \| YDR216W \| YDL029W \| YNL007C \| YFL009W \| YFL034C-B \| YHR102W \| YHR030C \| YJR065C \| YMR109W \| YPL140C |
| complex_2698 | YBL105C \| YBR160W \| YHR082C \| YJL095W \| YOL100W \| YGL003C \| YCR065W \| YER165W \| YGR159C \| YPR119W \| YJL060W \| YKL025C \| YKL129C \| YKR024C \| YOR231W |
| complex_2335 | YER103W \| YER081W \| YIL061C \| YOR027W \| YOR061W \| YOR254C \| YHR079C \| YJL034W \| YIL131C \| YIL035C \| YKL073W \| YPL204W \| YNL027W |
| complex_2332 | YBR160W \| YER008C \| YGL003C \| YGR092W \| YJL187C \| YMR001C \| YNL298W \| YPL209C \| YMR304W \| YHL007C \| YIL106W \| YNL161W \| YPR119W \| YKL129C \| YLR362W |
| complex_2331 | YDL007W \| YGL004C \| YGL048C \| YDL029W \| YHR030C \| YJR065C \| YNL138W \| YOL004W \| YOR259C \| YDL047W \| YDR394W \| YKL145W \| YOR117W \| YGR232W \| YPL140C |
| complex_2691 | YAL005C \| YDR477W \| YER081W \| YNL209W \| YPL106C \| YDL229W \| YMR001C \| YDR012W \| YER129W \| YDR194C \| YGL035C \| YDR216W \| YBR160W \| YLR347C \| YMR104C |
| complex_2690 | YAL005C \| YLR310C \| YNL064C \| YPL106C \| YAL029C \| YDR507C \| YBL016W \| YDL159W \| YLR362W \| YBL075C \| YPR119W \| YGR040W \| YKR048C \| YLL024C \| YMR139W |

| | |
|---|---|
| complex_1247 | YDL195W \| YGL019W \| YIL131C \| YOR061W \| YGL254W \| YIL118W \| YLR399C \| YML010W \| YML074C \| YOL004W \| YOR039W \| YIL035C \| YJL056C \| YHL027W \| YOL145C |
| complex_1246 | YAL005C \| YBL075C \| YDL229W \| YDR477W \| YLL024C \| YLR310C \| YPR119W \| YBR160W \| YDR356W \| YER129W \| YDR146C \| YNL209W \| YDR507C \| YHR018C \| YMR139W |
| complex_1244 | YBR160W \| YJR059W \| YLR210W \| YDR188W \| YDR142C \| YDR212W \| YGL137W \| YJR064W \| YKR036C \| YLL011W \| YNL317W \| YOR212W \| YJL106W \| YGR092W \| YIL142W |
| complex_1241 | YBL105C \| YER008C \| YOL100W \| YOR231W \| YPL151C \| YBR160W \| YGL003C \| YKL129C \| YMR001C \| YPR119W \| YPR120C \| YMR304W \| YJL095W \| YJL187C \| YPR165W |
| complex_1240 | YBR160W \| YDL155W \| YGL003C \| YNL068C \| YPR119W \| YPR120C \| YNL161W \| YER008C \| YMR304W \| YGR092W \| YIL106W \| YMR001C \| YJL060W \| YJL187C \| YKL101W |
| complex_3418 | YDR212W \| YBR198C \| YFL033C \| YGL137W \| YJL106W \| YJL164C \| YNL317W \| YOR212W \| YPL203W \| YIL033C \| YKL166C \| YNL027W \| YNL093W \| YIL142W \| YJR064W |
| complex_369 | YDL087C \| YKL173W \| YDL208W \| YDR235W \| YPR182W \| YER029C \| YMR001C \| YPL151C \| YHR086W \| YLR275W \| YIL061C \| YER146W \| YLR147C \| YMR213W \| YNL147W |
| complex_28 | YAL032C \| YKL095W \| YKL173W \| YLL036C \| YLR117C \| YLR424W \| YMR213W \| YBR065C \| YDL030W \| YDR364C \| YER013W \| YMR001C \| YFL017W-A \| YGL120C \| YPL151C |
| complex_368 | YAL005C \| YBL075C \| YBR085W \| YER081W \| YER107C \| YER165W \| YLR310C \| YNL064C \| YNL209W \| YOR027W \| YPL106C \| YAR019C \| YGR092W \| YLL024C \| YNL041C |
| complex_3415 | YBL105C \| YJL095W \| YOR212W \| YOR231W \| YPL151C \| YDR188W \| YDR142C \| YDR212W \| YIL142W \| YJR064W \| YNL317W \| YBR198C \| YDR075W \| YKL095W \| YJL014W |
| complex_3414 | YAL029C \| YMR139W \| YBR160W \| YCR065W \| YDL155W \| YGL003C \| YGR092W \| YMR001C \| YNL102W \| YDR507C \| YKR048C \| YIL106W \| YNL068C \| YPR111W \| YPR119W |
| complex_3413 | YBL026W \| YER112W \| YER146W \| YJL124C \| YHL002W \| YLR362W \| YNR006W \| YOR076C \| YJR022W \| YFR052W \| YLR147C \| YPR182W \| YKL213C \| YLL039C \| YNL147W |
| complex_3412 | YDL047W \| YDL029W \| YOR259C \| YPR178W \| YDR394W \| YHR030C \| YLR216C \| YGL137W \| YJL095W \| YPL140C \| YKL073W \| YKL129C \| YMR109W \| YPR054W \| YOR027W |
| complex_22 | YBR160W \| YGL048C \| YGR092W \| YKL145W \| YDL097C \| YDL007W \| YDR394W \| YEL037C \| YER012W \| YHR027C \| YOR117W \| YOR259C \| YOR261C \| YDL147W \| YNL016W |
| complex_3410 | YDR188W \| YBR198C \| YDR142C \| YGL137W \| YJL106W \| YJL164C \| YNL154C \| YNL317W \| YOR212W \| YDR212W \| YDR075W \| YIL142W \| YJR064W \| YDR264C \| YJL014W |
| complex_361 | YAL005C \| YBL075C \| YBR085W \| YER081W \| YER107C \| YER165W \| YLR310C \| YNL064C \| YNL209W \| YOR027W \| YPL106C \| YCL024W \| YJL187C \| YLL024C \| YNL041C |
| complex_360 | YAL005C \| YBR085W \| YER081W \| YER107C \| YER165W \| YLR310C \| YNL064C \| YNL209W \| YOR027W \| YPL106C \| YBL075C \| YJL128C \| YNR031C \| YLL024C \| YNL041C |
| complex_2329 | YBL105C \| YBR160W \| YGL003C \| YNR047W \| YCR065W \| YDL155W \| YDR356W \| YPR119W \| YER165W \| YJR059W \| YGR092W \| YIL106W \| YOL100W \| YKL129C \| YPR111W |
| complex_2689 | YCR065W \| YBR160W \| YDR356W \| YPR119W \| YDR379W \| YGR152C \| YLR229C \| YGR108W \| YAL041W \| YOR127W \| YHL007C \| YKL129C \| YJL187C \| YNL298W \| YNR047W |

| | |
|---|---|
| complex_2326 | YBL105C \| YER165W \| YGL003C \| YPR119W \| YPR120C \| YBR133C \| YHL007C \| YJL187C \| YKL101W \| YBR160W \| YJL060W \| YMR001C \| YNL068C \| YMR304W \| YNL307C |
| complex_2325 | YBL105C \| YBR160W \| YER165W \| YHR018C \| YJL095W \| YOL100W \| YPR119W \| YCR065W \| YKL129C \| YLR096W \| YKL073W \| YHR030C \| YDL029W \| YPL140C \| YMR109W |
| complex_2687 | YBL016W \| YLR362W \| YBR160W \| YBR200W \| YDL159W \| YPR119W \| YDL029W \| YOR212W \| YHL007C \| YJL095W \| YMR109W \| YMR304W \| YJL187C \| YKL129C \| YNL298W |
| complex_2324 | YAL005C \| YLR310C \| YPL106C \| YBL016W \| YLR362W \| YBL075C \| YDL159W \| YDR477W \| YGL158W \| YLL024C \| YLR113W \| YNL209W \| YER028C \| YJL128C \| YNL064C |
| complex_2686 | YAL024C \| YKL116C \| YBL045C \| YCR065W \| YPR191W \| YBL105C \| YER165W \| YOL100W \| YPR119W \| YBR160W \| YER129W \| YJR059W \| YKL048C \| YMR036C \| YNR047W |
| complex_2322 | YDL007W \| YGL004C \| YDL029W \| YPL140C \| YDL047W \| YDR394W \| YGL048C \| YHR030C \| YKL145W \| YMR216C \| YOR117W \| YOR259C \| YGR232W \| YPR191W \| YPR178W |
| complex_2321 | YBL075C \| YLR362W \| YNL298W \| YBR160W \| YCR065W \| YER008C \| YGR108W \| YHR018C \| YJL187C \| YJR059W \| YKL129C \| YPR119W \| YBR200W \| YHL007C \| YNR047W |
| complex_2320 | YBL045C \| YGR159C \| YDR432W \| YER165W \| YHR086W \| YIL061C \| YIR001C \| YNL016W \| YOR204W \| YPR119W \| YGL049C \| YKR059W \| YMR246W \| YGR162W \| YDR235W |
| complex_1238 | YAL005C \| YBL075C \| YLR310C \| YPR119W \| YBL016W \| YDL159W \| YGR040W \| YBR200W \| YBR160W \| YJL187C \| YNL298W \| YER165W \| YHL007C \| YLR362W \| YKL129C |
| complex_1237 | YAL005C \| YBL075C \| YDL017W \| YER081W \| YGR253C \| YLR310C \| YOR027W \| YBR160W \| YGL137W \| YDL229W \| YDR477W \| YNL209W \| YLL024C \| YPL106C \| YPL240C |
| complex_1597 | YAL005C \| YLR310C \| YBL016W \| YDL159W \| YLR362W \| YBL075C \| YNL064C \| YOR027W \| YGR040W \| YDL229W \| YPL106C \| YHR018C \| YLL024C \| YPL240C \| YPR119W |
| complex_1234 | YBR160W \| YDL155W \| YGL003C \| YJL187C \| YMR001C \| YNL068C \| YPR119W \| YPR120C \| YCR034W \| YMR304W \| YGR092W \| YAR019C \| YIL106W \| YNL161W \| YNL298W |
| complex_1233 | YAL005C \| YBL075C \| YLR310C \| YNL064C \| YOR027W \| YPL240C \| YBL016W \| YDL159W \| YHR018C \| YLR362W \| YNL298W \| YPL042C \| YCR088W \| YGR040W \| YLL024C |
| complex_19 | YBL026W \| YER112W \| YER146W \| YJL124C \| YDR378C \| YJR022W \| YLR275W \| YNL147W \| YER029C \| YLR147C \| YKL173W \| YPR178W \| YPR182W \| YMR001C \| YOR159C |
| complex_359 | YAL005C \| YDR172W \| YER165W \| YLL024C \| YLR310C \| YNL064C \| YOR027W \| YPL106C \| YBL075C \| YDR477W \| YPR119W \| YPR128C \| YDL229W \| YNL209W \| YER028C |
| complex_1590 | YBR160W \| YCR065W \| YDL155W \| YGR092W \| YJR059W \| YKL129C \| YDR146C \| YLR131C \| YPR119W \| YIL106W \| YNL161W \| YPR111W \| YDL029W \| YJL095W \| YMR109W |
| complex_3405 | YAL005C \| YBR155W \| YDL229W \| YDR356W \| YDR477W \| YLR310C \| YNL209W \| YOR185C \| YPL106C \| YEL030W \| YER081W \| YHR102W \| YFL037W \| YLL024C \| YNL161W |
| complex_356 | YAL005C \| YBR085W \| YER081W \| YER107C \| YER165W \| YLR310C \| YNL064C \| YNL209W \| YOR027W \| YPL106C \| YBL075C \| YKL101W \| YNL307C \| YLL024C \| YNL041C |
| complex_15 | YBL026W \| YER112W \| YER146W \| YDR378C \| YJL124C \| YJR022W \| YLR275W \| YNL147W \| YKL173W \| YPR182W \| YPR178W \| YLR147C \| YMR213W \| YMR001C \| YOR159C |

| | |
|---|---|
| complex_3403 | YBL105C \| YBR160W \| YGL003C \| YPR119W \| YDR146C \| YDR432W \| YPL263C \| YNL016W \| YER165W \| YIR001C \| YGR159C \| YMR216C \| YOR204W \| YGR162W \| YKR095W |
| complex_354 | YDL007W \| YFR004W \| YDL097C \| YDR394W \| YGL048C \| YGR262C \| YKL145W \| YOR117W \| YOR259C \| YOR261C \| YDL147W \| YDL029W \| YHR102W \| YGR232W \| YHR030C |
| complex_3402 | YBL045C \| YBL105C \| YER165W \| YGL022W \| YGR159C \| YHR174W \| YIL035C \| YOL100W \| YOR061W \| YPR119W \| YGL019W \| YOR039W \| YGL044C \| YIL131C \| YOL145C |
| complex_352 | YAL005C \| YBL075C \| YBR085W \| YER081W \| YER107C \| YER165W \| YLR310C \| YNL064C \| YNL209W \| YOR027W \| YPL106C \| YCR091W \| YNR047W \| YLL024C \| YNL041C |
| complex_2317 | YER081W \| YFL016C \| YIL061C \| YGR040W \| YJR045C \| YMR290C \| YIL035C \| YNL308C \| YLR369W \| YOR232W \| YPL204W \| YNL027W |
| complex_2315 | YBR160W \| YGL003C \| YNL102W \| YCR065W \| YDL155W \| YGR092W \| YKL129C \| YDR146C \| YPR119W \| YIL106W \| YDL028C \| YNL068C \| YPR111W \| YMR001C \| YPR120C |
| complex_2677 | YDR188W \| YBR198C \| YDR142C \| YFL033C \| YGL137W \| YJL106W \| YJL164C \| YNL317W \| YOR212W \| YDR212W \| YDR075W \| YIL142W \| YJR064W \| YJL014W \| YNL027W |
| complex_2314 | YBR160W \| YGL003C \| YNL102W \| YCR065W \| YDL155W \| YER165W \| YPR119W \| YPR120C \| YGR092W \| YMR001C \| YIL106W \| YNL068C \| YPR111W \| YJR059W \| YKL129C |
| complex_2673 | YBR160W \| YFL029C \| YGL003C \| YGR092W \| YMR001C \| YPR120C \| YDL108W \| YDR212W \| YBR198C \| YGL137W \| YKR036C \| YNL317W \| YPR054W \| YIL142W \| YJR064W |
| complex_2310 | YBR160W \| YGL003C \| YNL102W \| YBR200W \| YLR362W \| YPR119W \| YCR065W \| YHL007C \| YKL129C \| YHL035C \| YJL187C \| YMR001C \| YNL068C \| YNL298W \| YPR120C |
| complex_2671 | YAL005C \| YFL033C \| YLL024C \| YNL064C \| YNL209W \| YOR027W \| YPL031C \| YBL075C \| YDL229W \| YPR119W \| YDR477W \| YHR018C \| YPL042C \| YJL164C \| YPL106C |
| complex_1228 | YAL021C \| YGR092W \| YKR036C \| YMR001C \| YPL042C \| YBR160W \| YGL048C \| YGL137W \| YKL145W \| YDL007W \| YEL037C \| YDL147W \| YDR394W \| YOR117W \| YOR259C |
| complex_1589 | YAL005C \| YBL075C \| YGR092W \| YLR310C \| YNL007C \| YNL209W \| YPR119W \| YBR160W \| YCR065W \| YDL155W \| YKL129C \| YLR131C \| YNL161W \| YDR146C \| YER103W |
| complex_1221 | YBR109C \| YFR014C \| YGL019W \| YOR039W \| YOR061W \| YDL195W \| YIL035C \| YIL131C \| YGL254W \| YIL118W \| YML010W \| YML074C \| YOL145C \| YPL131W \| YOR326W |
| complex_1581 | YAR019C \| YGR092W \| YHR018C \| YIL106W \| YBL075C \| YLR310C \| YNL064C \| YPR111W \| YBR160W \| YCR065W \| YKL129C \| YDL155W \| YNL161W \| YPR119W \| YMR001C |
| complex_349 | YAL005C \| YBL075C \| YBR085W \| YER107C \| YER165W \| YLR310C \| YNL064C \| YOR027W \| YPL106C \| YPR119W \| YBR160W \| YJR059W \| YDR146C \| YLL024C \| YNL041C |
| complex_1580 | YAL005C \| YML092C \| YPR111W \| YBR160W \| YCR065W \| YDL155W \| YGL003C \| YAR019C \| YER165W \| YPR119W \| YGR092W \| YHR018C \| YIL106W \| YJL060W \| YMR001C |
| complex_348 | YAL005C \| YDL229W \| YER081W \| YER165W \| YLL024C \| YLR310C \| YNL007C \| YOR027W \| YPL106C \| YPR128C \| YDR146C \| YNL209W \| YPL031C \| YDR477W \| YNL041C |
| complex_346 | YBR160W \| YDR507C \| YER021W \| YGL048C \| YGR232W \| YKL145W \| YDL007W \| YFR004W \| YDL097C \| YDR394W \| YOR117W \| YOR259C \| YOR261C \| YDL147W \| YDL029W |

| | |
|---|---|
| complex_344 | YDL097C \| YDL007W \| YDR394W \| YEL037C \| YGL048C \| YHR027C \| YHR030C \| YKL145W \| YOR117W \| YOR259C \| YOR261C \| YDL147W \| YGR092W \| YPL240C \| YOR027W |
| complex_343 | YAL005C \| YBL075C \| YBR085W \| YER081W \| YER107C \| YER165W \| YLR310C \| YNL064C \| YOR027W \| YPL106C \| YBR160W \| YPR054W \| YDR146C \| YLL024C \| YNL041C |
| complex_2308 | YBR160W \| YFL029C \| YLR210W \| YDL108W \| YDR188W \| YBR198C \| YDR142C \| YGL137W \| YLL011W \| YNL317W \| YOR212W \| YDR212W \| YPR054W \| YIL142W \| YJR064W |
| complex_2307 | YBL045C \| YBL105C \| YDR432W \| YGL173C \| YGR159C \| YIR001C \| YNL016W \| YOR204W \| YPR119W \| YER165W \| YJL033W \| YGL049C \| YGR162W \| YJL095W \| YOR231W |
| complex_2669 | YAR019C \| YGR092W \| YNR047W \| YBR160W \| YCR065W \| YDL155W \| YKL129C \| YLR131C \| YNL068C \| YPR119W \| YCR091W \| YDR146C \| YIL106W \| YNL161W \| YDL029W |
| complex_2306 | YBR160W \| YGR092W \| YJL194W \| YDR394W \| YDL007W \| YDL029W \| YFR010W \| YGL048C \| YKL145W \| YOR117W \| YOR259C \| YLL034C \| YGL137W \| YIL007C \| YLR309C |
| complex_2668 | YDL007W \| YDL029W \| YDL047W \| YHR030C \| YOR259C \| YDR394W \| YGL048C \| YGR092W \| YKL145W \| YOR117W \| YEL037C \| YPL096W \| YLL034C \| YIL007C \| YOR027W |
| complex_2667 | YDL155W \| YKL129C \| YLR131C \| YNL161W \| YDR216W \| YDL029W \| YER148W \| YNL007C \| YFL034C-B \| YHR102W \| YHR030C \| YJL095W \| YJR065C \| YMR109W \| YPL140C |
| complex_2303 | YBR160W \| YCR065W \| YDL155W \| YGR092W \| YKL116C \| YKL129C \| YDR146C \| YLR131C \| YPR119W \| YIL106W \| YDL028C \| YNL161W \| YPR111W \| YDL029W \| YMR109W |
| complex_2665 | YAL021C \| YDR188W \| YGR040W \| YGR092W \| YKR036C \| YGL137W \| YGL190C \| YNL317W \| YDR212W \| YIL142W \| YJL106W \| YER020W \| YNL178W \| YMR146C \| YJR064W |
| complex_2664 | YBR109C \| YJR065C \| YCR065W \| YHR082C \| YDL029W \| YHR030C \| YJL095W \| YMR109W \| YKL129C \| YKL190W \| YLR433C \| YPR119W \| YML057W \| YLR096W \| YPL140C |
| complex_2301 | YAR019C \| YGR092W \| YBR160W \| YCR065W \| YKL129C \| YPR111W \| YDL147W \| YDL029W \| YFR004W \| YOR261C \| YDL155W \| YDR394W \| YIL106W \| YNL161W \| YPR119W |
| complex_2663 | YAL005C \| YBL075C \| YBR155W \| YJR032W \| YLR310C \| YMR314W \| YNL307C \| YOR027W \| YDL229W \| YKL101W \| YGL003C \| YLL024C \| YPL106C \| YPL240C \| YMR308C |
| complex_2300 | YBL045C \| YCR065W \| YGR092W \| YBR160W \| YBL105C \| YDL017W \| YIL106W \| YJL187C \| YMR001C \| YOL100W \| YPR119W \| YDR054C \| YER165W \| YGL137W \| YIL046W |
| complex_1216 | YBR087W \| YJR068W \| YMR078C \| YNL290W \| YOR217W \| YDR394W \| YDL007W \| YGL048C \| YKL145W \| YNL250W \| YOR259C \| YFR010W \| YOR261C \| YGL137W \| YOL094C |
| complex_1577 | YBR160W \| YCR065W \| YER008C \| YGL003C \| YHL007C \| YJL187C \| YKL129C \| YLR362W \| YMR001C \| YNL102W \| YNL298W \| YOR127W \| YBR200W \| YLR229C \| YPR119W |
| complex_1576 | YAL005C \| YLL024C \| YLR310C \| YDL229W \| YPL240C \| YDR216W \| YER028C \| YDR477W \| YER129W \| YGL035C \| YGL158W \| YLR113W \| YNL209W \| YPL106C |
| complex_1212 | YBL045C \| YBR160W \| YCR065W \| YGR159C \| YBL105C \| YER165W \| YNR047W \| YOL100W \| YPR119W \| YGL003C \| YDR432W \| YOR204W \| YJR059W \| YJL060W \| YKR095W |
| complex_1573 | YDL042C \| YGR192C \| YDR075W \| YKR036C \| YPR040W \| YDR188W \| YDR142C \| YDR212W \| YNL317W \| YOR212W \| YBR198C \| YJL106W \| YIL142W \| YJL014W \| YJR064W |

| | |
|---|---|
| complex_1572 | YAL005C \| YJL128C \| YLL024C \| YLR310C \| YLR362W \| YNL064C \| YOR027W \| YBL075C \| YDL229W \| YPL240C \| YDR477W \| YHR018C \| YPL042C \| YCR073C \| YPL106C |
| complex_1571 | YBL105C \| YBR160W \| YER165W \| YOL100W \| YPR119W \| YPR120C \| YER008C \| YGL003C \| YNL102W \| YMR304W \| YKL025C \| YJL187C \| YMR001C \| YNL068C \| YKL101W |
| complex_338 | YAL005C \| YBR155W \| YDR477W \| YER081W \| YLR310C \| YNL064C \| YNL209W \| YBL075C \| YOR222W \| YDL229W \| YNL308C \| YPL042C \| YEL030W \| YLL024C \| YPL106C |
| complex_698 | YAL005C \| YER081W \| YER165W \| YHR030C \| YLL024C \| YLR310C \| YNL041C \| YOR027W \| YOR254C \| YPL106C \| YPR128C \| YDR146C \| YJL034W \| YKL073W \| YJL095W |
| complex_697 | YAL005C \| YDL229W \| YIR005W \| YLR310C \| YNL007C \| YOR027W \| YPR128C \| YAR018C \| YHR064C \| YPL106C \| YDR146C \| YLL024C \| YNL209W \| YDR477W \| YNL041C |
| complex_694 | YAL032C \| YKL173W \| YLL036C \| YLR117C \| YLR424W \| YMR001C \| YMR213W \| YDL030W \| YER013W \| YGL120C \| YPL151C \| YJL187C \| YGL238W \| YMR240C \| YNL189W |
| complex_692 | YAL005C \| YBL075C \| YER081W \| YIR005W \| YLL024C \| YLR310C \| YNL007C \| YNL209W \| YOR027W \| YPL106C \| YPR128C \| YAR018C \| YDR088C \| YDR146C \| YHR064C |
| complex_2659 | YAL005C \| YBL075C \| YLL024C \| YLR310C \| YNL064C \| YPR119W \| YBL016W \| YBR160W \| YCR065W \| YJL187C \| YKL129C \| YCL024W \| YBR260C \| YHR018C \| YNL298W |
| complex_2658 | YDL195W \| YGL019W \| YIL035C \| YIL131C \| YNL112W \| YIL118W \| YLR399C \| YOL004W \| YOR039W \| YHR018C \| YJL034W \| YOR254C \| YOR326W \| YPL042C \| YOR061W |
| complex_2657 | YBL045C \| YPL203W \| YDR490C \| YER165W \| YCR008W \| YFL033C \| YGL137W \| YKL166C \| YLR216C \| YPL031C \| YHR135C \| YIL033C \| YMR022W \| YJL164C \| YNL027W |
| complex_2656 | YBR160W \| YCR065W \| YDL108W \| YDL155W \| YGL003C \| YKL129C \| YPR119W \| YDR146C \| YNL288W \| YGR092W \| YHL035C \| YIL106W \| YNL068C \| YPR111W \| YPR120C |
| complex_2652 | YBR160W \| YDL017W \| YGR092W \| YHR135C \| YMR001C \| YKL166C \| YDR358W \| YGL137W \| YLR216C \| YIL033C \| YMR022W \| YJL164C \| YNL093W \| YLL039C \| YPL203W |
| complex_2651 | YAR019C \| YGR092W \| YCR065W \| YBR160W \| YDL108W \| YDL155W \| YKL129C \| YDR146C \| YDR379W \| YPR119W \| YMR036C \| YHL035C \| YIL106W \| YNL161W \| YPR111W |
| complex_2650 | YDR432W \| YER165W \| YGR159C \| YHR086W \| YIL061C \| YIR001C \| YJL033W \| YKR095W \| YNL016W \| YOR204W \| YPR119W \| YPR191W \| YGL049C \| YGR162W \| YNL189W |
| complex_1207 | YBR065C \| YLR117C \| YMR001C \| YMR213W \| YPL151C \| YDL087C \| YKL173W \| YML049C \| YDR235W \| YER029C \| YGL120C \| YHR086W \| YLR147C \| YOR319W \| YGL096W |
| complex_1567 | YBL045C \| YOL123W \| YPR054W \| YCL011C \| YDL084W \| YDR432W \| YOR204W \| YPR119W \| YDL108W \| YFL029C \| YER165W \| YGL173C \| YGR159C \| YGL044C \| YKL139W |
| complex_1204 | YBL105C \| YBR160W \| YER165W \| YIL035C \| YBR109C \| YGL019W \| YOR039W \| YOR061W \| YNL102W \| YML010W \| YGL254W \| YIL118W \| YIL131C \| YML074C \| YOL145C |
| complex_1566 | YAL005C \| YLR310C \| YBL075C \| YOR027W \| YDL147W \| YDL007W \| YDR356W \| YDR394W \| YGL004C \| YGL048C \| YKL145W \| YOR117W \| YOR259C \| YGR092W \| YGR232W |
| complex_1565 | YAR019C \| YBR160W \| YML064C \| YGL003C \| YJL187C \| YNL102W \| YDL155W \| YKL129C \| YNL068C \| YGR092W \| YMR001C \| YPR119W \| YIL106W \| YPR111W \| YPR120C |

| | |
|---|---|
| complex_1202 | YBL105C \| YOR231W \| YBR160W \| YCR065W \| YKL129C \| YPR054W \| YER165W \| YHR030C \| YPR119W \| YPR191W \| YDL029W \| YJL095W \| YMR109W \| YPL140C \| YOL100W |
| complex_1201 | YAL005C \| YBL075C \| YDL229W \| YDR356W \| YDR477W \| YLL024C \| YLR310C \| YNL209W \| YOR027W \| YPL106C \| YLL016W \| YPL042C \| YGR040W \| YNL098C \| YOR101W |
| complex_1200 | YAL041W \| YOR212W \| YBL016W \| YDL159W \| YBR160W \| YBR200W \| YCR065W \| YHL007C \| YJL187C \| YKL129C \| YNL298W \| YOR127W \| YPR119W \| YLR229C \| YLR362W |
| complex_1562 | YBL105C \| YBR160W \| YGL003C \| YOL100W \| YPR119W \| YPR120C \| YER008C \| YNL068C \| YNL102W \| YPL209C \| YPR175W \| YMR304W \| YJL095W \| YJL187C \| YMR001C |
| complex_328 | YAL005C \| YDL229W \| YER081W \| YLL024C \| YLR310C \| YNL007C \| YNL175C \| YOR027W \| YPL106C \| YPR128C \| YDR477W \| YMR001C \| YNL209W \| YER028C \| YNL041C |
| complex_321 | YAL032C \| YDR364C \| YKL173W \| YLL036C \| YLR117C \| YLR424W \| YMR001C \| YMR213W \| YDL030W \| YER013W \| YGL120C \| YPL151C \| YHR086W \| YLR275W \| YMR240C |
| complex_680 | YAL005C \| YER081W \| YHR030C \| YIL061C \| YLL024C \| YLR310C \| YNL041C \| YOR027W \| YOR254C \| YPL106C \| YPR128C \| YDR146C \| YJL034W \| YKL073W \| YHR079C |
| complex_2647 | YDL007W \| YEL037C \| YHR027C \| YDR216W \| YGR123C \| YPL240C \| YDR394W \| YGL048C \| YKL145W \| YOR117W \| YOR259C \| YIL148W \| YLL039C \| YDR457W \| YNR011C |
| complex_2643 | YDR188W \| YDR142C \| YDR212W \| YNL317W \| YOR212W \| YBR198C \| YGL116W \| YJL106W \| YJL164C \| YKL166C \| YPL203W \| YFL033C \| YIL142W \| YJL014W \| YJR064W |
| complex_2642 | YBR160W \| YAL019W \| YDL108W \| YER008C \| YGL003C \| YNL298W \| YPL209C \| YPR119W \| YPR120C \| YMR304W \| YFL029C \| YHL035C \| YJL187C \| YKR048C \| YMR001C |
| complex_2640 | YBR160W \| YGL003C \| YNL102W \| YCR065W \| YCR091W \| YDL155W \| YGR092W \| YPR119W \| YIL106W \| YNL068C \| YPR111W \| YKL129C \| YMR001C \| YNR047W \| YPR120C |
| complex_1559 | YCR065W \| YBR160W \| YGR092W \| YDL155W \| YKL129C \| YDR379W \| YMR036C \| YPR119W \| YDL029W \| YHL007C \| YHL035C \| YIL106W \| YNL161W \| YPR111W \| YLR131C |
| complex_1558 | YAL005C \| YCL011C \| YDL229W \| YDR477W \| YNL209W \| YPL106C \| YPR119W \| YBR169C \| YER028C \| YLL024C \| YER129W \| YFL029C \| YGL158W \| YHR018C \| YKL139W |
| complex_1556 | YBR034C \| YIL079C \| YOL123W \| YDL175C \| YNL189W \| YOR204W \| YPR191W \| YDR432W \| YER165W \| YHR086W \| YPR119W \| YGL044C \| YLR335W \| YGR159C \| YGL003C |
| complex_1553 | YCR065W \| YBR160W \| YGR092W \| YDL155W \| YKL129C \| YHL007C \| YHL035C \| YPR119W \| YIL106W \| YNL161W \| YPR111W \| YLR131C \| YOR127W \| YMR036C \| YNL298W |
| complex_1552 | YBR160W \| YDL108W \| YFL029C \| YIL131C \| YNL102W \| YER165W \| YIL035C \| YML010W \| YOR061W \| YGL019W \| YGL254W \| YIL118W \| YML074C \| YOR039W \| YOL145C |
| complex_1551 | YAL005C \| YBL075C \| YER081W \| YGR253C \| YLR310C \| YOR027W \| YPL042C \| YDL229W \| YDR477W \| YER012W \| YGL137W \| YLL024C \| YPL106C \| YPL240C \| YNL209W |
| complex_1550 | YBL030C \| YDL029W \| YGL137W \| YDR394W \| YHR030C \| YOR259C \| YDL097C \| YDR142C \| YFR004W \| YOR261C \| YDL147W \| YGR092W \| YDR188W \| YJR064W \| YDR523C |
| complex_316 | YAL005C \| YNL308C \| YBL075C \| YLR310C \| YNL064C \| YOR027W \| YPL240C \| YDL229W \| YDR477W \| YER028C \| YNL209W \| YGL070C \| YLL024C \| YPL042C \| YPL106C |

| | |
|---|---|
| complex_314 | YAL005C \| YER081W \| YLR310C \| YNL064C \| YOR027W \| YBL075C \| YGL070C \| YNL308C \| YDL229W \| YNL175C \| YPL106C \| YDR477W \| YNL209W \| YER103W \| YLL024C |
| complex_312 | YAL005C \| YBR085W \| YER081W \| YER107C \| YER165W \| YLR310C \| YNL064C \| YNL209W \| YOR027W \| YPL106C \| YBL075C \| YBL105C \| YOL100W \| YLL024C \| YNL041C |
| complex_311 | YBL105C \| YJL095W \| YOR231W \| YDL007W \| YFR004W \| YDL097C \| YDR394W \| YGL048C \| YHR027C \| YHR030C \| YKL145W \| YOR117W \| YOR259C \| YOR261C \| YDL147W |
| complex_672 | YAL005C \| YDR172W \| YER165W \| YGL254W \| YLL024C \| YNL007C \| YNL209W \| YOR027W \| YPR119W \| YBR160W \| YER103W \| YDL229W \| YPL106C \| YDR146C \| YDR477W |
| complex_2637 | YBL045C \| YJL095W \| YPR054W \| YPR191W \| YBL105C \| YOR231W \| YDL029W \| YPL140C \| YDR394W \| YER165W \| YGL022W \| YKL173W \| YHR030C \| YHR082C \| YPL151C |
| complex_2997 | YBR160W \| YCR065W \| YDR054C \| YKL129C \| YMR001C \| YDL132W \| YFL009W \| YIL046W \| YDL155W \| YGR092W \| YPR119W \| YIL106W \| YPR111W \| YJL187C \| YNL298W |
| complex_2633 | YAL005C \| YLR310C \| YBR160W \| YDL155W \| YDR146C \| YDR356W \| YGR270W \| YKR095W \| YLR131C \| YNL041C \| YNL298W \| YOR127W \| YPR119W \| YDL229W |
| complex_2632 | YAL024C \| YJL187C \| YBR160W \| YBL105C \| YGR092W \| YIL106W \| YJR059W \| YKL129C \| YMR001C \| YNL298W \| YOL100W \| YPR111W \| YPR119W \| YER165W \| YNL175C |
| complex_2993 | YBL105C \| YBR160W \| YER008C \| YGL003C \| YOL100W \| YOR231W \| YAL019W \| YIL112W \| YKL129C \| YMR001C \| YPR175W \| YER165W \| YPR119W \| YJL060W \| YJL095W |
| complex_2992 | YDR188W \| YDR142C \| YDR212W \| YGL137W \| YIL142W \| YJR064W \| YNL317W \| YOR212W \| YBR198C \| YDR075W \| YHL007C \| YJL014W \| YML095C \| YMR201C \| YPL022W |
| complex_1549 | YAL005C \| YIR005W \| YLR310C \| YNL007C \| YOR027W \| YPL106C \| YBL016W \| YDL159W \| YBL075C \| YDR283C \| YLR362W \| YMR001C \| YGR040W \| YLL024C \| YMR139W |
| complex_1547 | YAL005C \| YDL229W \| YPL106C \| YPR119W \| YDR477W \| YIL033C \| YJL164C \| YLL024C \| YNL209W \| YFL033C \| YGL116W \| YNL093W \| YKL166C \| YPL203W \| YNL027W |
| complex_1546 | YAL041W \| YBR200W \| YLR229C \| YNL298W \| YBR031W \| YBR160W \| YLR362W \| YOR127W \| YCR065W \| YPR119W \| YHL007C \| YJL187C \| YKL129C \| YJL095W \| YMR109W |
| complex_1545 | YBR160W \| YDL155W \| YDR507C \| YER008C \| YGL003C \| YIL106W \| YPL209C \| YPR119W \| YPR120C \| YKR048C \| YMR139W \| YMR304W \| YGR092W \| YMR001C \| YJL187C |
| complex_1540 | YDL029W \| YDR394W \| YDR523C \| YHR030C \| YOR212W \| YOR259C \| YDL097C \| YDR142C \| YFR004W \| YOR261C \| YDL147W \| YNL037C \| YJL014W \| YJR064W \| YPL140C |
| complex_309 | YAL032C \| YKL095W \| YKL173W \| YLL036C \| YLR117C \| YLR424W \| YMR213W \| YER013W \| YER003C \| YMR001C \| YFL017W-A \| YPL151C \| YGL120C \| YNL189W \| YGL238W |
| complex_669 | YAL005C \| YBL075C \| YBR160W \| YER165W \| YLL024C \| YLR310C \| YNL064C \| YNL209W \| YOR027W \| YPR119W \| YDL155W \| YER103W \| YDR146C \| YLR131C \| YPL031C |
| complex_668 | YAL032C \| YBL105C \| YKL173W \| YLL036C \| YLR117C \| YLR424W \| YMR213W \| YDL030W \| YER013W \| YGL120C \| YGL174W \| YKR024C \| YMR001C \| YMR240C \| YPL151C |
| complex_664 | YAL005C \| YBL016W \| YBL075C \| YBR085W \| YBR155W \| YER081W \| YFL016C \| YIL061C \| YLR310C \| YJR045C \| YLR369W \| YEL030W \| YOR232W \| YLR362W \| YNL027W |

| | |
|---|---|
| complex_2629 | YAL021C \| YGR092W \| YKR036C \| YPL042C \| YDR188W \| YDR142C \| YGL137W \| YHL007C \| YNL317W \| YOR212W \| YPL151C \| YDR212W \| YIL142W \| YJL014W \| YJR064W |
| complex_2628 | YBL105C \| YBR160W \| YER081W \| YGL076C \| YJL095W \| YOL100W \| YOR231W \| YPL151C \| YPR119W \| YCR065W \| YDR146C \| YFL016C \| YER165W \| YKL129C \| YMR109W |
| complex_2627 | YBL016W \| YMR139W \| YBL045C \| YCR065W \| YPL204W \| YBR160W \| YBL105C \| YDR507C \| YNR047W \| YPR119W \| YDR432W \| YER165W \| YGR159C \| YOR204W \| YLR310C |
| complex_2989 | YDL007W \| YEL037C \| YGL048C \| YLR424W \| YDL047W \| YDL029W \| YHR030C \| YDR394W \| YGR092W \| YKL145W \| YOR117W \| YOR259C \| YIL007C \| YLL034C \| YLR309C |
| complex_2626 | YBR160W \| YDL155W \| YGL003C \| YGL116W \| YMR001C \| YOR317W \| YPR119W \| YPR120C \| YDR212W \| YJL187C \| YMR304W \| YGR092W \| YIL142W \| YKL101W \| YKR048C |
| complex_2625 | YBR160W \| YNL102W \| YCR065W \| YDL025C \| YPL131W \| YPR119W \| YDL155W \| YGR092W \| YIL106W \| YIL112W \| YJL187C \| YNL298W \| YKL129C \| YMR001C \| YNL288W |
| complex_2987 | YBR160W \| YDL145C \| YDR146C \| YER008C \| YER129W \| YJL187C \| YLR131C \| YMR001C \| YPR119W \| YDR216W \| YDR477W \| YER028C \| YGL035C \| YLR212C \| YNL027W |
| complex_2621 | YBL105C \| YBR160W \| YER008C \| YJL095W \| YOR231W \| YJR065C \| YNL102W \| YCR065W \| YDL029W \| YKL129C \| YMR109W \| YDR394W \| YHR030C \| YPL140C \| YPR119W |
| complex_2983 | YBR160W \| YER008C \| YGR238C \| YHR158C \| YJL187C \| YLR096W \| YMR001C \| YDL155W \| YGR092W \| YDR146C \| YLR131C \| YPR119W \| YDR285W \| YGR270W \| YOR195W |
| complex_1539 | YBL045C \| YGR159C \| YBL105C \| YER165W \| YNL317W \| YOL123W \| YPR119W \| YDR432W \| YHR086W \| YIR001C \| YOR204W \| YJL033W \| YGL049C \| YNL016W \| YGR162W |
| complex_1898 | YBL045C \| YCR065W \| YMR001C \| YBR160W \| YGL003C \| YGR092W \| YJL187C \| YNL102W \| YER165W \| YGR159C \| YOR204W \| YPR119W \| YIR005W \| YKL025C \| YKL129C |
| complex_1896 | YAL005C \| YER081W \| YIL061C \| YLR310C \| YNL064C \| YPL106C \| YBL016W \| YDL159W \| YBL075C \| YGR040W \| YNL127W \| YLL024C \| YMR139W \| YLR362W \| YNL189W |
| complex_1533 | YAR019C \| YGR092W \| YPR111W \| YBR160W \| YCR065W \| YDL155W \| YGL003C \| YKL129C \| YDR146C \| YPR119W \| YER165W \| YIL106W \| YNL068C \| YJL060W \| YKR095W |
| complex_1895 | YBL075C \| YOR027W \| YDL007W \| YEL037C \| YHR027C \| YDR394W \| YGL048C \| YKL145W \| YOR117W \| YOR259C \| YPL240C \| YHR169W \| YNR011C \| YIL148W \| YNL027W |
| complex_1894 | YBL045C \| YDL108W \| YIL131C \| YBR160W \| YBL105C \| YFL029C \| YDL195W \| YGL019W \| YOR061W \| YDR432W \| YER165W \| YGR159C \| YPR119W \| YOR039W \| YIL035C |
| complex_1530 | YAL005C \| YBL075C \| YER165W \| YLL024C \| YLR310C \| YNL007C \| YOL100W \| YPR119W \| YBL016W \| YLR362W \| YBL105C \| YBR160W \| YHL007C \| YDL159W \| YGR040W |
| complex_1892 | YAL005C \| YER165W \| YLR310C \| YNL007C \| YBR160W \| YEL030W \| YHL007C \| YJL034W \| YJL187C \| YDR054C \| YHR030C \| YNL161W \| YHR102W \| YIL046W \| YKL073W |
| complex_1890 | YDL007W \| YDR394W \| YFR010W \| YGL048C \| YKL145W \| YLR424W \| YDL029W \| YHR030C \| YGR092W \| YGL137W \| YIL007C \| YLL034C \| YLR309C \| YOR117W \| YOR259C |
| complex_656 | YAL005C \| YBL075C \| YDL229W \| YDR172W \| YER165W \| YGL254W \| YLL024C \| YLR310C \| YNL007C \| YOR027W \| YPR054W \| YPR119W \| YDR146C \| YER103W \| YPL106C |

| | |
|---|---|
| complex_655 | YAL005C \| YBR085W \| YER081W \| YFL016C \| YIL061C \| YLR310C \| YOR027W \| YBL075C \| YCL024W \| YNL041C \| YJR045C \| YLR369W \| YDR146C \| YJL187C \| YOR232W |
| complex_654 | YDL007W \| YFR004W \| YDL097C \| YDR394W \| YGL048C \| YGR262C \| YKL145W \| YOR117W \| YOR259C \| YOR261C \| YDL147W \| YDL029W \| YDR356W \| YPL026C \| YDL058W |
| complex_653 | YBL075C \| YBR085W \| YBR155W \| YER081W \| YFL016C \| YIL061C \| YLR310C \| YDR146C \| YEL030W \| YJR045C \| YLR369W \| YDR507C \| YMR139W \| YNL041C \| YOR232W |
| complex_2617 | YBR160W \| YDL029W \| YDL047W \| YDR394W \| YGL004C \| YGL048C \| YHR030C \| YKL145W \| YOR117W \| YOR259C \| YOR267C \| YJL095W \| YPL140C \| YMR109W \| YKL129C |
| complex_2979 | YAL005C \| YER081W \| YLR310C \| YNL064C \| YPL106C \| YBL016W \| YDL159W \| YLR362W \| YBL075C \| YGR040W \| YDR343C \| YDR477W \| YLL024C \| YMR139W \| YNL209W |
| complex_2616 | YBR160W \| YDL145C \| YER008C \| YER129W \| YGL003C \| YJL187C \| YLR131C \| YPR119W \| YMR304W \| YDR216W \| YDR477W \| YER028C \| YGL035C \| YJL060W \| YNL027W |
| complex_2977 | YBR109C \| YGL019W \| YOR039W \| YOR061W \| YCR084C \| YPL042C \| YDL042C \| YGR192C \| YGL254W \| YIL035C \| YIL118W \| YIL131C \| YML010W \| YML074C \| YOL145C |
| complex_2614 | YBR031W \| YDR477W \| YBR160W \| YCR065W \| YDL155W \| YGR092W \| YKL129C \| YLR131C \| YDR216W \| YDL029W \| YFR027W \| YPR119W \| YIL106W \| YMR109W \| YPL042C |
| complex_2976 | YAR019C \| YGR092W \| YHR169W \| YIL106W \| YML064C \| YPR120C \| YBR160W \| YGL003C \| YDL155W \| YDR212W \| YGL137W \| YIL142W \| YKR036C \| YPL042C \| YMR001C |
| complex_2613 | YBR160W \| YER008C \| YFL009W \| YGL003C \| YMR001C \| YDL132W \| YDL155W \| YDR054C \| YDR328C \| YIL046W \| YJL187C \| YMR304W \| YHL035C \| YNL298W \| YPR119W |
| complex_2975 | YBL045C \| YPL140C \| YPR054W \| YDL029W \| YDL047W \| YKL139W \| YDL145C \| YHR030C \| YKL129C \| YMR109W \| YER165W \| YFL029C \| YGL137W \| YJL095W \| YBR109C |
| complex_2970 | YAL005C \| YJL128C \| YLL024C \| YLR310C \| YNL064C \| YNR031C \| YOR027W \| YBL016W \| YDL159W \| YHR018C \| YLR362W \| YBL075C \| YCR073C \| YPL240C \| YMR022W |
| complex_1527 | YAL005C \| YER165W \| YLR310C \| YPL106C \| YBL016W \| YBR160W \| YBL075C \| YDR146C \| YLL024C \| YDR507C \| YLR362W \| YMR139W \| YJR032W \| YNL298W |
| complex_1525 | YAR019C \| YGR092W \| YBR160W \| YKL145W \| YPR111W \| YDR394W \| YDL007W \| YFR010W \| YGL048C \| YOR117W \| YOR259C \| YGL137W \| YNL250W \| YIL007C \| YIL106W |
| complex_1887 | YBL105C \| YBR160W \| YER165W \| YJL095W \| YLR210W \| YOL100W \| YOR231W \| YPL151C \| YPR119W \| YER008C \| YGL003C \| YJL187C \| YMR001C \| YMR304W \| YGR159C |
| complex_1524 | YDL097C \| YDL007W \| YDR394W \| YEL037C \| YGL048C \| YHR027C \| YHR030C \| YKL145W \| YOR117W \| YOR259C \| YGR159C \| YER165W \| YNL053W \| YKL073W \| YOR208W |
| complex_1886 | YBL045C \| YCR065W \| YHR082C \| YBL105C \| YBR160W \| YOL100W \| YDR432W \| YER165W \| YGR159C \| YPR119W \| YGL022W \| YJL095W \| YKL129C \| YOR231W \| YPL151C |
| complex_1882 | YAR019C \| YGR092W \| YHR169W \| YBR160W \| YCR065W \| YGL003C \| YKL129C \| YNL102W \| YPR120C \| YDL155W \| YIL106W \| YNL068C \| YPR111W \| YPR119W \| YMR001C |
| complex_1881 | YBL105C \| YBR160W \| YER165W \| YGL003C \| YJL095W \| YPR119W \| YCR065W \| YER008C \| YJL187C \| YKL129C \| YMR001C \| YNL102W \| YPL209C \| YMR304W \| YNL298W |

| | |
|---|---|
| complex_1880 | YAL005C \| YLR310C \| YNL064C \| YPL106C \| YBL016W \| YDL159W \| YLR362W \| YBL075C \| YGR040W \| YHR018C \| YDL229W \| YDR477W \| YGL158W \| YLL024C \| YNL209W |
| complex_647 | YAL032C \| YKL173W \| YLL036C \| YLR117C \| YLR424W \| YMR213W \| YDL030W \| YER013W \| YMR001C \| YGL120C \| YGR092W \| YGR136W \| YJL074C \| YMR240C \| YPL151C |
| complex_645 | YAL005C \| YDL229W \| YER081W \| YIR005W \| YLL024C \| YLR310C \| YNL007C \| YOR027W \| YPL106C \| YPR128C \| YDR146C \| YNL209W \| YDR283C \| YMR001C \| YNL041C |
| complex_641 | YAL005C \| YER107C \| YER165W \| YLL016W \| YLR310C \| YNL064C \| YOR027W \| YPL106C \| YBL016W \| YDL159W \| YLR362W \| YBL075C \| YGR040W \| YJL128C \| YLL024C |
| complex_2609 | YBL105C \| YBR160W \| YHR018C \| YJL095W \| YOL100W \| YPR119W \| YCR065W \| YJL187C \| YKL129C \| YNL298W \| YPL140C \| YKL073W \| YHR030C \| YDL029W \| YMR109W |
| complex_2608 | YBR031W \| YBR160W \| YLR362W \| YOR127W \| YCR065W \| YNL298W \| YBR200W \| YLR229C \| YPR119W \| YHL007C \| YKL129C \| YJL187C \| YDL029W \| YMR109W \| YNR047W |
| complex_2607 | YAL005C \| YLR310C \| YNL064C \| YPL106C \| YBL016W \| YDL159W \| YLR362W \| YNL127W \| YBL075C \| YGR040W \| YDR477W \| YLL024C \| YJR068W \| YLR371W \| YNL209W |
| complex_2969 | YAL005C \| YBR155W \| YER081W \| YEL030W \| YGL011C \| YCR057C \| YMR314W \| YOL038W \| YGR135W \| YGR253C \| YOR362C \| YHR064C \| YAR018C \| YML092C \| YMR308C |
| complex_2605 | YAR019C \| YGR092W \| YBR160W \| YDL007W \| YLR424W \| YDL029W \| YDR394W \| YGL048C \| YKL145W \| YOR117W \| YOR259C \| YIL007C \| YIL106W \| YPR111W \| YPR119W |
| complex_2604 | YAL005C \| YLL024C \| YLR310C \| YNL007C \| YOR027W \| YBL075C \| YJL128C \| YDL159W \| YBL016W \| YGR040W \| YGL158W \| YHL007C \| YNL161W \| YHR102W \| YLR362W |
| complex_2966 | YBR109C \| YGL019W \| YGR270W \| YOR039W \| YOR061W \| YER081W \| YGL254W \| YFL037W \| YIL035C \| YML010W \| YML074C \| YHL027W \| YIL131C \| YJL056C \| YOL145C |
| complex_2601 | YBR160W \| YDR356W \| YGL048C \| YGR092W \| YKL145W \| YOR127W \| YDL029W \| YDR394W \| YOR259C \| YDL047W \| YGL004C \| YDL147W \| YDL007W \| YOR117W \| YGR232W |
| complex_2963 | YAL005C \| YGL011C \| YGR135W \| YML092C \| YMR314W \| YOL038W \| YOR362C \| YCR057C \| YLR129W \| YDR283C \| YLR222C \| YLR409C \| YNL030W \| YDR477W \| YMR308C |
| complex_2961 | YCR084C \| YOL004W \| YOR061W \| YPL042C \| YDL195W \| YGL019W \| YIL035C \| YIL131C \| YNL112W \| YDR477W \| YER028C \| YGL035C \| YIL118W \| YLR399C \| YOR039W |
| complex_1879 | YAL005C \| YDL229W \| YER165W \| YAL024C \| YBR160W \| YBL105C \| YER129W \| YOL100W \| YNL308C \| YDR477W \| YNL209W \| YER028C \| YLL024C \| YPL106C \| YPL209C |
| complex_1878 | YAL005C \| YJL187C \| YNL007C \| YOR027W \| YPL209C \| YBL075C \| YBR200W \| YBR160W \| YHL007C \| YPR119W \| YCR065W \| YHR018C \| YKL129C \| YLR362W \| YNL298W |
| complex_1877 | YDL029W \| YDR394W \| YOR259C \| YDL047W \| YGL004C \| YDL147W \| YDL007W \| YGL048C \| YKL145W \| YOR117W \| YPL140C \| YGR092W \| YLR309C \| YGR232W \| YHR030C |
| complex_1514 | YAL005C \| YGR159C \| YLR310C \| YBL075C \| YPR119W \| YBL105C \| YDL229W \| YDR172W \| YDR146C \| YOR069W \| YDR432W \| YER165W \| YPL031C \| YLL024C \| YPL106C |
| complex_1876 | YAL005C \| YBL075C \| YJR032W \| YLL024C \| YLR310C \| YMR139W \| YNL007C \| YBL016W \| YDL159W \| YGR040W \| YDR477W \| YGL158W \| YLL039C \| YLR362W \| YNL298W |

| | |
|---|---|
| complex_1875 | YBL045C \| YCR065W \| YLR362W \| YBL075C \| YBL105C \| YBR160W \| YHL007C \| YBR133C \| YER165W \| YNL298W \| YPR119W \| YJL187C \| YCL024W \| YKL129C \| YOL100W |
| complex_1873 | YAR019C \| YML064C \| YMR001C \| YBR160W \| YGL003C \| YIL131C \| YJL187C \| YKL129C \| YNL102W \| YPR119W \| YDL155W \| YDR146C \| YGR092W \| YIL106W \| YPR111W |
| complex_1510 | YBR160W \| YCL024W \| YMR001C \| YNL298W \| YKR048C \| YCR002C \| YDR507C \| YHR107C \| YDL225W \| YDR054C \| YKL101W \| YJL187C \| YJR076C \| YLR314C \| YPL150W |
| complex_1870 | YBR160W \| YAL019W \| YER008C \| YGL003C \| YGR238C \| YHR158C \| YJL187C \| YMR001C \| YDL155W \| YNL161W \| YDR146C \| YLR131C \| YPR119W \| YGR092W \| YIL106W |
| complex_638 | YAL005C \| YER081W \| YIR005W \| YLL024C \| YLR310C \| YNL007C \| YNL209W \| YOR027W \| YPR128C \| YDR088C \| YDR146C \| YHR064C \| YPL106C \| YMR001C \| YNL041C |
| complex_637 | YAL005C \| YER081W \| YER165W \| YLL024C \| YLR310C \| YNL007C \| YNL209W \| YOR027W \| YPL106C \| YPR128C \| YDL229W \| YER129W \| YNL041C \| YDR477W \| YER028C |
| complex_636 | YAL005C \| YBL075C \| YBR085W \| YER081W \| YER165W \| YLL016W \| YLL024C \| YLR310C \| YNL064C \| YNL209W \| YDR146C \| YPL106C \| YGR040W \| YLR362W \| YNL041C |
| complex_995 | YAL005C \| YBL075C \| YLL024C \| YLR310C \| YNL064C \| YNL209W \| YPR119W \| YAL029C \| YMR139W \| YBL016W \| YDR146C \| YPL106C \| YDR507C \| YHR018C \| YGR040W |
| complex_633 | YDL007W \| YFR004W \| YDL097C \| YDR394W \| YGL048C \| YGR262C \| YKL145W \| YOR117W \| YOR259C \| YOR261C \| YDL147W \| YDL029W \| YJL008C \| YMR104C \| YDR477W |
| complex_994 | YBR160W \| YDR507C \| YFL009W \| YIL046W \| YJL187C \| YMR001C \| YNL298W \| YCR002C \| YCL024W \| YHR107C \| YDR054C \| YDR328C \| YKL101W \| YJR076C \| YLR314C |
| complex_993 | YDL195W \| YGL019W \| YIL035C \| YIL131C \| YGR116W \| YIL118W \| YLR399C \| YML010W \| YOL004W \| YOR039W \| YOR326W \| YJR014W \| YJR017C \| YOL145C \| YOR061W |
| complex_992 | YAL005C \| YBL075C \| YDL229W \| YDR356W \| YDR477W \| YLL024C \| YLR310C \| YNL209W \| YOR027W \| YPL106C \| YPR119W \| YER081W \| YER103W \| YHR030C \| YPR054W |
| complex_991 | YAL005C \| YDL229W \| YDR172W \| YDR356W \| YER165W \| YLR310C \| YNL007C \| YOR351C \| YPL106C \| YPR119W \| YPR128C \| YBR160W \| YAL024C \| YDR146C |
| complex_2957 | YBL075C \| YLR362W \| YBR160W \| YAL041W \| YDR379W \| YHL007C \| YJL187C \| YKL129C \| YMR001C \| YNL298W \| YOR127W \| YPR119W \| YGR152C \| YLR229C \| YOR204W |
| complex_2956 | YBR160W \| YCR065W \| YDR356W \| YJL187C \| YNL102W \| YNL298W \| YOR127W \| YPR119W \| YGR152C \| YAL041W \| YHL007C \| YKL129C \| YLR229C \| YMR109W \| YMR001C |
| complex_2955 | YAL005C \| YDL229W \| YNL209W \| YPR119W \| YDL192W \| YDL145C \| YGL137W \| YDR477W \| YER081W \| YGL076C \| YER122C \| YER165W \| YJL164C \| YNL027W \| YPL106C |
| complex_2954 | YAR019C \| YBR160W \| YGR092W \| YDL029W \| YDL007W \| YEL037C \| YGL048C \| YDR394W \| YKL145W \| YOR117W \| YOR259C \| YIL045W \| YIL106W \| YPR111W \| YLL034C |
| complex_2952 | YDL042C \| YGR192C \| YDR075W \| YKR036C \| YDR188W \| YDR212W \| YNL317W \| YOR212W \| YBR198C \| YFL033C \| YJL164C \| YPL203W \| YIL142W \| YJL014W \| YJR064W |
| complex_1509 | YBR160W \| YDL155W \| YGL003C \| YGR092W \| YIL106W \| YJL060W \| YJL187C \| YMR001C \| YPR111W \| YPR119W \| YNL161W \| YMR304W \| YAR019C \| YNL016W \| YNL189W |

| | |
|---|---|
| complex_2950 | YBR082C \| YBR160W \| YKL210W \| YBR109C \| YMR109W \| YDL029W \| YHR018C \| YHR030C \| YJL095W \| YOR208W \| YKL129C \| YPR119W \| YLR096W \| YPL119C \| YPL140C |
| complex_1507 | YAL005C \| YBL075C \| YLL024C \| YLR310C \| YNL064C \| YNL209W \| YBL016W \| YDL159W \| YLR362W \| YDR477W \| YHR018C \| YGL158W \| YGR040W \| YJL128C \| YPL106C |
| complex_1506 | YBL016W \| YBR200W \| YLR362W \| YBL075C \| YNL298W \| YBR160W \| YCR065W \| YHL007C \| YHR018C \| YJL187C \| YKL129C \| YMR001C \| YNL102W \| YPR119W \| YDL159W |
| complex_1867 | YBL075C \| YLR362W \| YNL064C \| YNR047W \| YBR160W \| YBR200W \| YCR065W \| YHL007C \| YHR018C \| YJL187C \| YKL129C \| YNL298W \| YPR119W \| YCR091W \| YPL240C |
| complex_1865 | YAL005C \| YLR310C \| YNL209W \| YOR388C \| YPL106C \| YBL075C \| YPR119W \| YDL108W \| YBR160W \| YDL155W \| YNL161W \| YDR146C \| YLL024C \| YLR131C \| YLR113W |
| complex_1502 | YAL005C \| YBR155W \| YBR160W \| YDL229W \| YDR477W \| YER081W \| YLR310C \| YBL075C \| YPR119W \| YDR356W \| YLL024C \| YNL209W \| YOL100W \| YNR047W \| YPL106C |
| complex_1864 | YDL007W \| YDR394W \| YKL145W \| YLR424W \| YDL029W \| YHR030C \| YPL140C \| YGR092W \| YFR010W \| YGL048C \| YOR117W \| YGL137W \| YNL250W \| YIL007C \| YOR259C |
| complex_1861 | YBR160W \| YDL145C \| YER008C \| YHL007C \| YIL046W \| YJL187C \| YNL298W \| YOR127W \| YCR002C \| YLR229C \| YNL007C \| YMR304W \| YAL041W \| YJR076C \| YNL161W |
| complex_1860 | YAL005C \| YLR310C \| YNL209W \| YBL016W \| YLL024C \| YDL229W \| YDR356W \| YDR477W \| YPL106C \| YDL192W \| YER028C \| YER129W \| YGL035C \| YGL158W \| YER165W |
| complex_989 | YAL005C \| YBR155W \| YDL229W \| YDR477W \| YER081W \| YER165W \| YNL209W \| YOR254C \| YDR146C \| YJL034W \| YKL073W \| YPL106C \| YHR030C \| YJL095W \| YPL140C |
| complex_988 | YAL005C \| YBL075C \| YER081W \| YLR310C \| YOR027W \| YOR362C \| YPL042C \| YDL229W \| YFL016C \| YDR477W \| YNL209W \| YLL024C \| YPL106C \| YPL240C \| YGL137W |
| complex_986 | YAL005C \| YDR477W \| YER081W \| YER165W \| YHR030C \| YLL024C \| YNL209W \| YOR027W \| YOR254C \| YPL106C \| YPL140C \| YDR146C \| YJL034W \| YKL073W \| YJL095W |
| complex_621 | YAL005C \| YAR018C \| YBL075C \| YIR005W \| YJR032W \| YLL024C \| YLR310C \| YNL064C \| YOR027W \| YPR119W \| YPR128C \| YHR064C \| YDR146C \| YDR477W \| YPL042C |
| complex_2948 | YAL005C \| YBL075C \| YDR477W \| YLL024C \| YLR310C \| YNL007C \| YNL064C \| YNL209W \| YOR027W \| YPL106C \| YPR119W \| YDL229W \| YDR356W \| YER028C |
| complex_2947 | YAL005C \| YDR477W \| YNL064C \| YNL209W \| YBL075C \| YBR031W \| YDR216W \| YDL029W \| YFR027W \| YJL095W \| YPR119W \| YHR018C \| YKL129C \| YMR109W \| YPL042C |
| complex_2946 | YBR160W \| YGL003C \| YGR092W \| YNL161W \| YDL155W \| YDR054C \| YLR131C \| YER165W \| YHR102W \| YPR119W \| YFL009W \| YIL046W \| YKL025C \| YJL187C \| YMR001C |
| complex_2945 | YBR028C \| YHR135C \| YDL017W \| YLL039C \| YDR490C \| YFL033C \| YKL166C \| YPL240C \| YJL164C \| YGL137W \| YLR216C \| YIL033C \| YMR022W \| YDR457W \| YPL203W |
| complex_2944 | YAR019C \| YGR092W \| YNL161W \| YBR160W \| YCR065W \| YDR146C \| YKL129C \| YLR131C \| YDL155W \| YHR018C \| YER103W \| YOR388C \| YFL034C-B \| YIL106W \| YPR119W |
| complex_2942 | YBL105C \| YNL307C \| YBR160W \| YGL003C \| YMR001C \| YPR119W \| YPR120C \| YCL024W \| YER165W \| YKL101W \| YMR304W \| YJL060W \| YJL095W \| YJL187C \| YKR048C |

| | |
|---|---|
| complex_2941 | YAR019C \| YBR109C \| YHR018C \| YKL129C \| YBR160W \| YDL029W \| YDL155W \| YGR092W \| YIL106W \| YMR001C \| YPR119W \| YHL007C \| YNL161W \| YMR109W \| YOR358W |
| complex_1857 | YAL005C \| YBL075C \| YJR032W \| YLR310C \| YNL064C \| YOR027W \| YOR185C \| YBR155W \| YDR356W \| YIL149C \| YFR002W \| YOR257W \| YLL024C \| YLR293C \| YLR347C |
| complex_1854 | YAL005C \| YDR477W \| YLR310C \| YNL064C \| YPL106C \| YBL075C \| YDL159W \| YBL016W \| YHR018C \| YGL158W \| YGR040W \| YLR362W \| YLL024C \| YMR139W \| YOR326W |
| complex_1853 | YAR019C \| YGR092W \| YBR160W \| YCR065W \| YDL155W \| YKL129C \| YLR131C \| YPR119W \| YJL095W \| YDR146C \| YDL029W \| YIL106W \| YDL028C \| YNL161W \| YMR109W |
| complex_1851 | YBR109C \| YGL019W \| YLR212C \| YOR039W \| YOR061W \| YER165W \| YIL035C \| YML010W \| YNL307C \| YGL254W \| YIL118W \| YIL131C \| YML074C \| YKL101W \| YOL145C |
| complex_619 | YAL005C \| YBL075C \| YER081W \| YIL061C \| YLL024C \| YLR310C \| YOR027W \| YOR222W \| YOR254C \| YPL106C \| YHR079C \| YJL034W \| YOR061W \| YKL073W \| YNL027W |
| complex_1850 | YAL041W \| YBR200W \| YHL007C \| YLR229C \| YNL298W \| YBR160W \| YCR065W \| YKL129C \| YOR127W \| YPR119W \| YLR362W \| YDR054C \| YIL046W \| YJL187C \| YPR141C |
| complex_617 | YAL005C \| YBL075C \| YDL229W \| YGL070C \| YLL024C \| YLR310C \| YNL064C \| YNL209W \| YNL308C \| YOR027W \| YPL106C \| YPL240C \| YLR362W \| YER103W \| YGR040W |
| complex_616 | YAL005C \| YER165W \| YNL308C \| YBR155W \| YDL229W \| YDR477W \| YLR310C \| YNL007C \| YPL106C \| YHR064C \| YLL024C \| YER028C \| YAR018C \| YMR116C \| YNL209W |
| complex_615 | YDL007W \| YFR004W \| YDL029W \| YNL161W \| YDL097C \| YDR394W \| YGL048C \| YKL145W \| YOR117W \| YOR259C \| YOR261C \| YDL147W \| YHR102W \| YFL034C-B \| YHL007C |
| complex_613 | YAL005C \| YCL011C \| YDL229W \| YLR310C \| YNL007C \| YNL209W \| YDL192W \| YER165W \| YDR432W \| YKL139W \| YDR477W \| YLL024C \| YER028C \| YPL106C \| YNL004W |
| complex_612 | YDL007W \| YFR004W \| YDL097C \| YDR394W \| YGL048C \| YGR262C \| YKL145W \| YOR117W \| YOR259C \| YOR261C \| YDL147W \| YCR073C \| YDL029W \| YJL008C \| YJL128C |
| complex_972 | YDR477W \| YLL024C \| YMR139W \| YEL030W \| YER081W \| YFL016C \| YIL061C \| YLR310C \| YNL308C \| YOR232W \| YGR254W \| YHR023W \| YJR045C \| YLR369W \| YNL027W |
| complex_610 | YAL005C \| YLR310C \| YNL308C \| YBL075C \| YJL128C \| YLR362W \| YNL064C \| YOR027W \| YDL229W \| YDR477W \| YNL209W \| YGL070C \| YPL106C \| YLL024C \| YPL240C |
| complex_2938 | YDR432W \| YER165W \| YGR159C \| YHR086W \| YIR001C \| YKR095W \| YNL016W \| YOR204W \| YPR119W \| YGL049C \| YKL059C \| YGR162W \| YJL033W \| YNL317W \| YKL025C |
| complex_2937 | YBL045C \| YJL187C \| YPR054W \| YPR191W \| YDL029W \| YDL047W \| YDL145C \| YGL137W \| YHR030C \| YDR394W \| YKL129C \| YMR109W \| YJL095W \| YNL298W \| YPL140C |
| complex_2933 | YBL084C \| YBR160W \| YGL003C \| YBR135W \| YER008C \| YJL187C \| YGR108W \| YKL129C \| YNL068C \| YHL007C \| YLR210W \| YMR001C \| YNL298W \| YPR119W \| YPR120C |
| complex_2932 | YAR019C \| YBR160W \| YGR092W \| YGL003C \| YNL102W \| YCR065W \| YDL155W \| YER008C \| YIL106W \| YNL068C \| YPR111W \| YPR119W \| YKL129C \| YMR001C \| YNR047W |
| complex_2931 | YBL045C \| YNL016W \| YDR432W \| YER165W \| YGL173C \| YGR159C \| YHR086W \| YIL061C \| YIR001C \| YOR204W \| YPR119W \| YDR489W \| YGL049C \| YGR162W \| YMR246W |

| | |
|---|---|
| complex_1849 | YAR019C \| YBR160W \| YNL298W \| YDL029W \| YCR065W \| YDL155W \| YGR092W \| YIL106W \| YOR127W \| YPR119W \| YHL007C \| YKL129C \| YNL161W \| YPR111W \| YLR131C |
| complex_1846 | YBL075C \| YLR362W \| YNL298W \| YBR160W \| YJL187C \| YNL102W \| YBR200W \| YPR119W \| YCR065W \| YGR108W \| YHL007C \| YKL129C \| YHL035C \| YHR018C \| YMR001C |
| complex_1843 | YCL011C \| YDR432W \| YER165W \| YGL120C \| YKL139W \| YDL084W \| YGR159C \| YNL004W \| YDL192W \| YDL229W \| YAL005C \| YDR477W \| YER028C \| YPL106C \| YNL112W |
| complex_1842 | YAL024C \| YBR160W \| YNL298W \| YBL105C \| YJL187C \| YCR065W \| YER165W \| YLR096W \| YPR119W \| YGR108W \| YHR158C \| YIL106W \| YPR111W \| YKL129C \| YOL100W |
| complex_968 | YAL005C \| YBL075C \| YLL024C \| YLR310C \| YNL064C \| YNL209W \| YPR119W \| YAL029C \| YBL016W \| YLR362W \| YDL159W \| YGR040W \| YHR018C \| YMR139W \| YPL106C |
| complex_605 | YAL005C \| YBR155W \| YER081W \| YLR310C \| YBL075C \| YFL016C \| YIL061C \| YOR222W \| YCL024W \| YJR045C \| YLR369W \| YEL030W \| YOR232W \| YJL187C \| YNL027W |
| complex_966 | YDL087C \| YKL173W \| YDL208W \| YPR178W \| YDR235W \| YPR182W \| YDR283C \| YGL078C \| YGL120C \| YHR086W \| YML046W \| YIL061C \| YLR147C \| YNL147W \| YOR159C |
| complex_965 | YBR160W \| YDR507C \| YIL046W \| YNL298W \| YCR002C \| YCL024W \| YHR107C \| YNL007C \| YDR054C \| YKL101W \| YMR001C \| YMR304W \| YJL187C \| YJR076C \| YLR314C |
| complex_600 | YAL005C \| YER081W \| YGR159C \| YLL024C \| YLR310C \| YNL007C \| YPR128C \| YCL043C \| YER165W \| YDL229W \| YDR477W \| YNL041C \| YPL106C \| YNL209W \| YER028C |
| complex_961 | YAL005C \| YER165W \| YLL016W \| YLL024C \| YLR310C \| YNL064C \| YBL016W \| YDL159W \| YJL128C \| YLR362W \| YBL075C \| YCR073C \| YGR040W \| YNR031C |
| complex_2929 | YAL029C \| YBR109C \| YGL106W \| YKL190W \| YMR139W \| YFL005W \| YML057W \| YGR095C \| YGR195W \| YOR326W \| YHR023W \| YLR433C \| YKL101W \| YOR358W |
| complex_2928 | YBL045C \| YAR019C \| YGR159C \| YDL155W \| YGR092W \| YNL161W \| YDR432W \| YER165W \| YMR216C \| YDR477W \| YMR104C \| YKR036C \| YGL137W \| YIL106W \| YPL042C |
| complex_2927 | YDL007W \| YGL004C \| YGL048C \| YGR119C \| YOR117W \| YDL047W \| YDL029W \| YHR030C \| YLR248W \| YDR394W \| YKL145W \| YOR259C \| YJL041W \| YIL007C \| YPR178W |
| complex_2926 | YCR084C \| YOL004W \| YPL042C \| YDL195W \| YGL019W \| YIL035C \| YIL131C \| YNL112W \| YLR399C \| YOR039W \| YJL081C \| YOR244W \| YNL189W \| YOL145C \| YOR061W |
| complex_2925 | YBR160W \| YDL108W \| YDR146C \| YDR356W \| YER129W \| YGL035C \| YPR119W \| YPR141C \| YDR216W \| YER028C \| YDR477W \| YLR131C \| YNL027W \| YML085C |
| complex_2924 | YBR160W \| YDL155W \| YGL003C \| YGR092W \| YJL187C \| YMR001C \| YPR119W \| YPR120C \| YDR212W \| YGL137W \| YIL142W \| YKR036C \| YPL042C \| YHL035C \| YCR084C |
| complex_2921 | YBL105C \| YBR160W \| YER165W \| YGL003C \| YOL100W \| YPR119W \| YAL019W \| YCR065W \| YER008C \| YIL112W \| YPL209C \| YPR175W \| YGR159C \| YJL060W \| YKL025C |
| complex_2920 | YAL005C \| YDR356W \| YJL095W \| YBR109C \| YKL129C \| YBR160W \| YBL105C \| YCR065W \| YHR018C \| YPR119W \| YER165W \| YOR231W \| YPR165W \| YKL025C \| YOL100W |
| complex_1839 | YAL041W \| YOR212W \| YBR160W \| YER008C \| YGL003C \| YHL007C \| YJL187C \| YOR127W \| YMR304W \| YGR152C \| YLR229C \| YLR362W \| YNL298W \| YPR119W \| YPR165W |

| | |
|---|---|
| complex_1838 | YBL075C \| YHL007C \| YBR160W \| YER008C \| YJL187C \| YBR200W \| YPR119W \| YCR065W \| YGR108W \| YKL129C \| YHR018C \| YKL048C \| YLR362W \| YMR001C \| YNL298W |
| complex_1836 | YBR160W \| YBL105C \| YGL003C \| YMR001C \| YDL087C \| YKL173W \| YDL208W \| YER029C \| YGR092W \| YHR086W \| YPR119W \| YJL187C \| YLR147C \| YOL100W \| YPL151C |
| complex_1835 | YAL005C \| YER165W \| YLR310C \| YBR160W \| YDL229W \| YDR146C \| YLL024C \| YNL027W \| YDR172W \| YOR069W \| YDR477W \| YGL035C \| YER129W \| YPL106C |
| complex_1832 | YAL005C \| YBL075C \| YBR155W \| YGR092W \| YJR032W \| YLR310C \| YMR001C \| YOR027W \| YDL229W \| YDR477W \| YMR290C \| YLL024C \| YPL106C \| YPL240C \| YLR347C |
| complex_1831 | YAL024C \| YBR160W \| YJL187C \| YBL105C \| YCR065W \| YER165W \| YNL298W \| YOR269W \| YPR119W \| YHR158C \| YJL060W \| YJL095W \| YKL129C \| YLR096W \| YOL100W |
| complex_958 | YAL005C \| YLR310C \| YBL075C \| YDR283C \| YOR027W \| YOR388C \| YPL204W \| YDL229W \| YDR356W \| YPR119W \| YDR477W \| YNL209W \| YLL024C \| YPL106C \| YPL240C |
| complex_957 | YAL005C \| YBL075C \| YER165W \| YLR310C \| YPL106C \| YPR119W \| YBL016W \| YDL229W \| YDR172W \| YDR356W \| YDR146C \| YGL254W \| YLL024C \| YNL209W \| YLR362W |
| complex_956 | YBR160W \| YCR065W \| YJL187C \| YKL129C \| YOR127W \| YPL119C \| YBR200W \| YAL041W \| YGR152C \| YLR229C \| YPR119W \| YDR379W \| YHL007C \| YLR362W \| YNL298W |
| complex_2919 | YAL005C \| YBL016W \| YLR310C \| YNL064C \| YGL158W \| YBL075C \| YDR216W \| YDL029W \| YDR477W \| YHR018C \| YLL024C \| YKL129C \| YLR113W \| YLR362W \| YPR119W |
| complex_2918 | YAR019C \| YGR092W \| YBR160W \| YCR065W \| YDL155W \| YHL007C \| YIL106W \| YKL129C \| YLR131C \| YPR111W \| YPR119W \| YNL161W \| YDR216W \| YDL029W \| YFL034C-B |
| complex_2916 | YBL105C \| YBR160W \| YJL095W \| YKL210W \| YCR065W \| YDR432W \| YER165W \| YGR108W \| YGR159C \| YOR204W \| YKL129C \| YPR119W \| YHL035C \| YLR096W \| YOL100W |
| complex_2915 | YDL145C \| YJL187C \| YDR054C \| YCL024W \| YGL019W \| YGL254W \| YIL035C \| YIL131C \| YML010W \| YML074C \| YOR039W \| YIL046W \| YNL298W \| YOL145C \| YOR061W |
| complex_2913 | YBL050W \| YDR189W \| YLR026C \| YDL159W \| YER151C \| YGL200C \| YLR208W \| YHR098C \| YJR048W \| YPL204W \| YPR181C \| YIL109C \| YML012W \| YPL218W \| YNL049C |
| complex_2911 | YAL005C \| YBL075C \| YDR477W \| YER129W \| YGR253C \| YNL209W \| YOR027W \| YPL106C \| YBR160W \| YPR119W \| YDL229W \| YDR356W \| YER012W \| YLL024C \| YMR116C |
| complex_1827 | YAR019C \| YJR059W \| YBR160W \| YCR065W \| YDL155W \| YDR146C \| YGR092W \| YHR018C \| YIL106W \| YKL129C \| YLR131C \| YNR047W \| YPR111W \| YPR119W \| YNL161W |
| complex_1822 | YBL105C \| YBR160W \| YDL029W \| YFL029C \| YCR065W \| YDL108W \| YPR054W \| YDR432W \| YER165W \| YPR119W \| YPR191W \| YHR030C \| YJL095W \| YLR096W \| YKL129C |
| complex_945 | YBR160W \| YGR092W \| YMR290C \| YNL298W \| YCL024W \| YJL187C \| YCR002C \| YDL225W \| YDR507C \| YHR107C \| YPR119W \| YKL101W \| YMR001C \| YJR076C \| YLR314C |
| complex_943 | YAL005C \| YBR085W \| YBR155W \| YER081W \| YIR005W \| YJL141C \| YLR310C \| YNL007C \| YNL209W \| YBR160W \| YLR131C \| YDR146C \| YEL030W \| YHR064C \| YNL161W |
| complex_940 | YAL005C \| YBL075C \| YER081W \| YHR030C \| YLR310C \| YOR027W \| YPR054W \| YDL229W \| YDR477W \| YKL073W \| YNL209W \| YDR394W \| YLL024C \| YPL106C \| YPL240C |

| | |
|---|---|
| complex_2909 | YBL105C \| YJL095W \| YBR160W \| YCR065W \| YGR108W \| YHR018C \| YJL187C \| YKL129C \| YPR119W \| YBR200W \| YER103W \| YOR231W \| YHL007C \| YNL298W \| YOL100W |
| complex_2908 | YAR019C \| YBR160W \| YGR092W \| YKR095W \| YCR065W \| YDL155W \| YDR285W \| YPR119W \| YGL116W \| YIL112W \| YIL106W \| YNL068C \| YPR111W \| YKL129C \| YOR195W |
| complex_2906 | YBL105C \| YDL029W \| YBR160W \| YKL145W \| YCR065W \| YER165W \| YGR159C \| YPR119W \| YGR108W \| YOR231W \| YJL095W \| YMR109W \| YJL194W \| YKL129C \| YOL100W |
| complex_2904 | YBL045C \| YFL033C \| YDR490C \| YER165W \| YKL166C \| YGL137W \| YLR216C \| YNR032W \| YPL031C \| YHR135C \| YIL033C \| YMR022W \| YJL164C \| YNR031C \| YPL203W |
| complex_2903 | YBR160W \| YGR092W \| YBR198C \| YHR030C \| YDL007W \| YDR394W \| YEL037C \| YOR117W \| YDL047W \| YDL029W \| YGL048C \| YGL137W \| YIL007C \| YKL145W \| YOR259C |
| complex_1815 | YBL016W \| YBR200W \| YDL159W \| YLR310C \| YLR362W \| YBL075C \| YNL298W \| YBR160W \| YJL187C \| YDL047W \| YGR040W \| YLL039C \| YHL007C \| YIL046W \| YKL129C |
| complex_1810 | YDL047W \| YBR198C \| YKR036C \| YLR248W \| YDR188W \| YDR142C \| YJL106W \| YLR113W \| YNL317W \| YOR212W \| YDR212W \| YDR075W \| YIL142W \| YJL014W \| YJR064W |
| complex_938 | YAL005C \| YBR155W \| YDL229W \| YER081W \| YIR005W \| YLR310C \| YNL007C \| YPR128C \| YBR160W \| YJL141C \| YDR146C \| YHR064C \| YLL024C \| YNL209W \| YDR477W |
| complex_937 | YAL005C \| YDL229W \| YER165W \| YLR310C \| YNL007C \| YPL106C \| YPR112C \| YPR128C \| YBL105C \| YPR154W \| YHL002W \| YJL095W \| YNL041C \| YNL209W \| YOR231W |
| complex_936 | YAL005C \| YBL075C \| YLL024C \| YLR310C \| YNL064C \| YPR119W \| YBL016W \| YLR362W \| YBR200W \| YHL007C \| YNL298W \| YDL159W \| YGR040W \| YHR018C \| YKL129C |
| complex_935 | YBR160W \| YDL064W \| YKL101W \| YMR001C \| YNL298W \| YCL024W \| YHR107C \| YCR002C \| YDR507C \| YLR314C \| YDL225W \| YJL187C \| YJR076C \| YKL129C \| YNL307C |
| complex_1804 | YBL105C \| YBR160W \| YLR210W \| YOL100W \| YPL151C \| YGR092W \| YDR188W \| YGL137W \| YKR036C \| YNL317W \| YOR212W \| YPL042C \| YDR212W \| YIL142W \| YJR064W |
| complex_1802 | YBR160W \| YGL048C \| YGR092W \| YKL145W \| YDL029W \| YDR394W \| YDL007W \| YFR010W \| YLR424W \| YOR117W \| YOR259C \| YGL137W \| YNL250W \| YIL007C \| YIL106W |
| complex_1801 | YDL047W \| YBR198C \| YER155C \| YKR036C \| YOR267C \| YDR188W \| YDR142C \| YJL106W \| YNL317W \| YOR212W \| YDR212W \| YDR075W \| YIL142W \| YJL014W \| YJR064W |
| complex_926 | YBR109C \| YGL019W \| YOR039W \| YOR061W \| YOR326W \| YGL254W \| YIL035C \| YIL118W \| YIL131C \| YLR399C \| YML010W \| YML074C \| YOL004W \| YJR014W \| YOL145C |
| complex_925 | YAL029C \| YBR081C \| YER164W \| YGR252W \| YMR223W \| YBR198C \| YDR145W \| YDR146C \| YDR167W \| YDR216W \| YDR448W \| YDR477W \| YER028C \| YLL039C \| YMR022W |
| complex_924 | YAL005C \| YER081W \| YER165W \| YHR030C \| YLR310C \| YNL041C \| YOR254C \| YPL106C \| YPR128C \| YDR146C \| YJL034W \| YKL073W \| YJL095W \| YPL140C \| YGL116W |
| complex_922 | YAL005C \| YBR155W \| YDR477W \| YER165W \| YIL074C \| YLL024C \| YLR310C \| YNL007C \| YPL106C \| YEL030W \| YER129W \| YER028C \| YJR045C \| YFL037W \| YMR047C |
| complex_920 | YAL005C \| YER165W \| YLR310C \| YNL007C \| YOR185C \| YPR128C \| YDL229W \| YDR477W \| YNL041C \| YPL106C \| YNL209W \| YER028C \| YLR293C \| YNL189W \| YOL128C |

| | |
|---|---|
| complex_3099 | YDL017W \| YBR160W \| YDR394W \| YDL007W \| YGL048C \| YGR092W \| YKL145W \| YLR216C \| YOR117W \| YOR259C \| YGL137W \| YNR032W \| YHR030C \| YLR096W \| YOR027W |
| complex_3098 | YBL045C \| YCR065W \| YGL137W \| YPL153C \| YBL105C \| YBR160W \| YDL101C \| YDR432W \| YER165W \| YGR159C \| YPR119W \| YGL022W \| YKL025C \| YOL100W \| YPL151C |
| complex_3097 | YAL005C \| YBL075C \| YER165W \| YGR159C \| YLR310C \| YNL007C \| YOR027W \| YBL105C \| YKL073W \| YDR507C \| YFR024C-A \| YMR139W \| YEL030W \| YNL161W \| YHR030C |
| complex_919 | YAL005C \| YLR310C \| YNL308C \| YBL075C \| YGL070C \| YNL064C \| YOR027W \| YPL042C \| YDR477W \| YER165W \| YGL044C \| YNL209W \| YPL106C \| YLL024C \| YOL123W |
| complex_3091 | YAL029C \| YBR031W \| YHR023W \| YKL166C \| YJL164C \| YFL039C \| YNL027W \| YNR031C \| YHR135C \| YIL033C \| YNL093W \| YPL203W \| YMR022W \| YNL227C \| YPL131W |
| complex_915 | YAL005C \| YCL011C \| YLR310C \| YBR169C \| YNL127W \| YDL084W \| YKL139W \| YDL229W \| YDR432W \| YNL112W \| YDR477W \| YLL024C \| YER028C \| YPL106C \| YNL004W |
| complex_3087 | YAL021C \| YGR092W \| YPL042C \| YBL030C \| YGL137W \| YDR188W \| YDR477W \| YJL005W \| YJL106W \| YNL317W \| YOR212W \| YDR212W \| YIL142W \| YJR064W \| YKR036C |
| complex_3086 | YAL041W \| YOR212W \| YGR152C \| YLR229C \| YOR127W \| YHL007C \| YJL187C \| YIL046W \| YMR304W \| YLR362W \| YNL007C \| YAL005C \| YNL161W \| YNL298W \| YPR165W |
| complex_3081 | YBR160W \| YGL003C \| YGL137W \| YJL187C \| YMR001C \| YNL298W \| YPL115C \| YPR119W \| YPR120C \| YDL017W \| YDL160C \| YDR212W \| YIL142W \| YKR036C \| YGR092W |
| complex_3080 | YAR019C \| YHR111W \| YBR160W \| YCR065W \| YDL155W \| YGL003C \| YGR092W \| YKL129C \| YMR001C \| YNL102W \| YPR111W \| YPR119W \| YHR086W \| YIL106W \| YMR290C |
| complex_904 | YDL195W \| YGL019W \| YIL131C \| YOR061W \| YGL254W \| YIL118W \| YLR399C \| YML010W \| YML074C \| YOL004W \| YOR039W \| YJR014W \| YIL035C \| YOL145C \| YOR326W |
| complex_903 | YAL005C \| YBR155W \| YER081W \| YGR159C \| YLR310C \| YNL007C \| YPR128C \| YCL011C \| YDR432W \| YER165W \| YMR216C \| YDL229W \| YDR146C \| YKL073W \| YPL106C |
| complex_900 | YAL005C \| YLR310C \| YBL016W \| YBL075C \| YGR040W \| YLR362W \| YOR027W \| YOR388C \| YDL159W \| YDL229W \| YDR477W \| YNL209W \| YLL024C \| YPL106C \| YPL240C |
| complex_3079 | YBL105C \| YBR160W \| YCR057C \| YMR290C \| YOR061W \| YDL195W \| YGL019W \| YIL035C \| YIL131C \| YDL208W \| YKL129C \| YER165W \| YPR119W \| YOR039W \| YOL100W |
| complex_3076 | YAL005C \| YLR310C \| YPL106C \| YBL016W \| YDL159W \| YDR388W \| YGL158W \| YHR016C \| YLR362W \| YBL075C \| YDR477W \| YJL128C \| YLL024C \| YNL209W \| YGR040W |
| complex_3068 | YAL005C \| YLR310C \| YNL064C \| YPL106C \| YBL016W \| YDL159W \| YLR362W \| YBL075C \| YPR119W \| YGR040W \| YDR507C \| YKR048C \| YMR139W \| YLL024C \| YNL209W |
| complex_3067 | YBL045C \| YCR065W \| YKL101W \| YBR133C \| YER165W \| YHL007C \| YJL187C \| YBR160W \| YCL024W \| YNL307C \| YDR054C \| YPR119W \| YIL046W \| YKL129C \| YNL298W |
| complex_3066 | YBR065C \| YMR001C \| YPR119W \| YBR160W \| YGL003C \| YNL102W \| YCR065W \| YER165W \| YJL060W \| YJL187C \| YNL298W \| YPR120C \| YKL025C \| YKL129C \| YPL119C |
| complex_3061 | YBL105C \| YMR001C \| YPL151C \| YBR160W \| YAL019W \| YGL003C \| YPR119W \| YPR120C \| YER165W \| YGL035C \| YGR092W \| YHL035C \| YJL060W \| YJL187C \| YOL100W |

| | |
|---|---|
| complex_3058 | YBR198C \| YDR216W \| YDL029W \| YDR523C \| YHR030C \| YOR212W \| YDL097C \| YDR142C \| YFR004W \| YOR261C \| YDL147W \| YIL142W \| YJR064W \| YJL014W \| YPL140C |
| complex_3057 | YDL155W \| YLR131C \| YLR310C \| YMR109W \| YDR216W \| YDL029W \| YHR102W \| YNL007C \| YFL034C-B \| YHR030C \| YNL161W \| YPL140C \| YJL095W \| YKL129C \| YOR257W |
| complex_3056 | YBL045C \| YNL068C \| YOL123W \| YDR432W \| YER165W \| YGR159C \| YIR001C \| YJL033W \| YOR204W \| YPR120C \| YBR160W \| YGL049C \| YNL016W \| YGR162W \| YKL129C |
| complex_3055 | YBL105C \| YBR160W \| YER165W \| YJL095W \| YOL038W \| YOR231W \| YPL151C \| YPR119W \| YJL187C \| YMR001C \| YNL102W \| YCR065W \| YJL060W \| YKL129C \| YOL100W |
| complex_3054 | YAL005C \| YNL298W \| YBL105C \| YHR018C \| YJL095W \| YPR119W \| YBR160W \| YCR065W \| YDR285W \| YJR059W \| YKL129C \| YER165W \| YJL187C \| YKL025C \| YOL100W |
| complex_3053 | YBR031W \| YBR160W \| YOR127W \| YLR131C \| YNL161W \| YNL298W \| YDR216W \| YDL029W \| YFL034C-B \| YHR102W \| YHL007C \| YAL041W \| YKL129C \| YLR229C \| YJL187C |
| complex_3052 | YBR160W \| YAL041W \| YCR065W \| YGR092W \| YHL007C \| YJL187C \| YKL129C \| YMR001C \| YNL298W \| YOR127W \| YPR119W \| YGR152C \| YLR229C \| YLR362W \| YOR204W |
| complex_3051 | YAR019C \| YDR356W \| YML064C \| YBR160W \| YIL112W \| YNL102W \| YCR065W \| YDL155W \| YPR119W \| YGR092W \| YMR001C \| YIL106W \| YPR111W \| YKL129C \| YKL145W |
| complex_3050 | YBL084C \| YBR160W \| YGL003C \| YNL102W \| YCR065W \| YPR119W \| YDL155W \| YNL068C \| YER165W \| YJL060W \| YJL187C \| YMR001C \| YNL298W \| YPR120C \| YKL129C |
| complex_3048 | YBL105C \| YBR160W \| YJL095W \| YGL003C \| YNL102W \| YCR065W \| YER165W \| YGR159C \| YKL025C \| YOL100W \| YPR119W \| YMR001C \| YJL060W \| YKL129C \| YPL119C |
| complex_3047 | YAR019C \| YBR160W \| YDL029W \| YLR131C \| YCR065W \| YDL155W \| YGR092W \| YKL129C \| YDR216W \| YNL007C \| YHL007C \| YOR212W \| YIL106W \| YNL161W \| YPR119W |
| complex_3046 | YBL105C \| YER008C \| YGL003C \| YJL187C \| YOL100W \| YBR160W \| YLR212C \| YMR001C \| YPL209C \| YPR119W \| YPR141C \| YCR084C \| YDR356W \| YER165W \| YGR159C |
| complex_3045 | YAL005C \| YBL075C \| YCL011C \| YER081W \| YLL024C \| YDL229W \| YDR477W \| YER129W \| YPL240C \| YDR146C \| YDR216W \| YNL209W \| YER028C \| YGL035C \| YGL158W |
| complex_3044 | YBR160W \| YGL003C \| YMR001C \| YNL298W \| YPL115C \| YPR120C \| YDR212W \| YBR198C \| YJL106W \| YJL164C \| YNL317W \| YOR212W \| YIL142W \| YJR064W \| YJL187C |
| complex_3043 | YBR160W \| YDR507C \| YER008C \| YGL003C \| YGR092W \| YJL187C \| YNL068C \| YPR119W \| YPR120C \| YDL145C \| YMR304W \| YBR009C \| YHL035C \| YIL035C \| YMR001C |
| complex_3041 | YAL021C \| YAR019C \| YGR092W \| YKR036C \| YPL042C \| YBR160W \| YER008C \| YIL106W \| YJL187C \| YLR212C \| YMR001C \| YPR119W \| YPR141C \| YDR356W \| YML064C |
| complex_3038 | YBR160W \| YER008C \| YGL003C \| YGR092W \| YIL112W \| YJL187C \| YLR212C \| YMR001C \| YNL068C \| YNL102W \| YPL209C \| YPR119W \| YPR120C \| YPR141C \| YDR356W |
| complex_3037 | YBR109C \| YGL019W \| YGR270W \| YOR039W \| YOR061W \| YBR160W \| YIL131C \| YER103W \| YGL254W \| YIL035C \| YML010W \| YML074C \| YOL145C |
| complex_3399 | YAL029C \| YFL039C \| YJL095W \| YMR139W \| YOR035C \| YBR109C \| YFR014C \| YGL106W \| YOR326W \| YFL005W \| YHR023W \| YKL129C \| YPR119W \| YMR109W \| YMR304W |

| | |
|---|---|
| complex_3398 | YBR135W \| YBR160W \| YGL003C \| YKL210W \| YGR108W \| YKL129C \| YNL068C \| YGR159C \| YOR204W \| YPR119W \| YHL035C \| YPR120C \| YJL060W \| YKL025C \| YLR096W |
| complex_3035 | YBL026W \| YER112W \| YER146W \| YJL124C \| YDR356W \| YFR002W \| YIL149C \| YDL185W \| YKR095W \| YLL015W \| YJR022W \| YFR052W \| YLR147C \| YPR182W \| YNL147W |
| complex_3397 | YBL016W \| YDL159W \| YLR310C \| YLR362W \| YBL045C \| YCR065W \| YPR054W \| YBL075C \| YHR030C \| YKL166C \| YGL137W \| YGR040W \| YLR216C \| YPL240C \| YHL036W |
| complex_3034 | YBR198C \| YIL142W \| YJL014W \| YJR064W \| YDL145C \| YDL192W \| YDR188W \| YER122C \| YKR036C \| YLL011W \| YNL317W \| YOR212W \| YDR212W \| YGL003C \| YGL137W |
| complex_3396 | YAL021C \| YKR036C \| YMR001C \| YAR019C \| YBR160W \| YGL048C \| YIL106W \| YKL145W \| YDL007W \| YEL037C \| YDR394W \| YOR117W \| YOR259C \| YGR092W \| YPL042C |
| complex_3395 | YAL032C \| YLR117C \| YMR213W \| YBR160W \| YJL187C \| YKR095W \| YDL030W \| YER013W \| YMR001C \| YER029C \| YHR086W \| YGR136W \| YIR005W \| YMR240C \| YNL189W |
| complex_3032 | YBL045C \| YBL105C \| YNL016W \| YBR160W \| YDR390C \| YDR432W \| YER165W \| YGR159C \| YOR204W \| YPR119W \| YGL022W \| YJL033W \| YOL100W \| YGR162W |
| complex_3031 | YAL041W \| YOR212W \| YBR160W \| YER008C \| YJL187C \| YNL298W \| YOR127W \| YGR152C \| YLR229C \| YHL007C \| YDL240W \| YKL129C \| YLR362W \| YPR119W \| YPR165W |
| complex_3030 | YAL032C \| YBL105C \| YKL173W \| YLR117C \| YMR001C \| YMR213W \| YDL003W \| YDL074C \| YJL074C \| YFL008W \| YFR031C \| YHR086W \| YLR086W \| YLR147C \| YPL151C |
| complex_3390 | YER028C \| YDR477W \| YER129W \| YGL019W \| YGL179C \| YIL035C \| YIL118W \| YIL131C \| YML010W \| YML074C \| YOR039W \| YOR061W \| YGL035C \| YGL158W \| YOL145C |
| complex_3029 | YBR160W \| YCR065W \| YGR108W \| YJL187C \| YKL129C \| YNL102W \| YDL155W \| YGR092W \| YDR054C \| YER008C \| YIL046W \| YIL106W \| YPR119W \| YMR001C \| YNL298W |
| complex_3028 | YCL011C \| YDL084W \| YDR432W \| YFL039C \| YKL139W \| YNL004W \| YNL112W \| YOR204W \| YPR191W \| YDL175C \| YIL079C \| YHR086W \| YIL061C \| YKR095W \| YNL189W |
| complex_3023 | YCR002C \| YDR218C \| YDR507C \| YHR107C \| YJR076C \| YDL225W \| YDL029W \| YDL226C \| YBR118W \| YKL101W \| YMR001C \| YKL129C \| YMR109W \| YPR119W \| YLR314C |
| complex_3383 | YBL105C \| YBR160W \| YGR254W \| YJL095W \| YOR134W \| YOR231W \| YPR119W \| YPR165W \| YER008C \| YGL003C \| YCR065W \| YER165W \| YOL100W \| YGR159C \| YKL129C |
| complex_3382 | YDL195W \| YLR429W \| YNL112W \| YOR061W \| YER148W \| YFL039C \| YGL019W \| YLR399C \| YOR039W \| YIL118W \| YIL131C \| YOL004W \| YIL035C \| YOR326W \| YPL204W |
| complex_3381 | YAR019C \| YBR160W \| YHR169W \| YNL161W \| YCR065W \| YDL025C \| YDR212W \| YPR119W \| YDL155W \| YKL129C \| YDR146C \| YLR131C \| YGR092W \| YHL007C \| YIL106W |
| complex_2297 | YAR019C \| YGR092W \| YPR111W \| YBR160W \| YDL155W \| YGL003C \| YKL129C \| YMR001C \| YNL068C \| YNL102W \| YCR065W \| YPR119W \| YER165W \| YIL106W \| YKR095W |
| complex_2294 | YBL084C \| YGL003C \| YBR160W \| YBR200W \| YHL007C \| YJL187C \| YML092C \| YMR001C \| YOR127W \| YPR120C \| YAL041W \| YLR229C \| YMR304W \| YLR362W \| YNL298W |
| complex_2293 | YAR019C \| YGR092W \| YBL075C \| YGR135W \| YNL064C \| YPR111W \| YBR160W \| YCR065W \| YDL155W \| YKL129C \| YKR036C \| YHR018C \| YIL106W \| YPR119W \| YPL042C |

| | |
|---|---|
| complex_3019 | YBL105C \| YBR160W \| YOR231W \| YCR065W \| YER165W \| YGR254W \| YPR119W \| YBR260C \| YHR018C \| YHR030C \| YDL029W \| YPL140C \| YJL095W \| YKL129C \| YMR109W |
| complex_3016 | YBL016W \| YDL159W \| YBR160W \| YHL007C \| YNL298W \| YOR127W \| YOR204W \| YGR040W \| YGR159C \| YPR119W \| YAL041W \| YJL187C \| YKL129C \| YLR310C \| YLR362W |
| complex_3015 | YAL005C \| YAR019C \| YBR155W \| YGR092W \| YJR032W \| YLL024C \| YLR310C \| YOR027W \| YHR169W \| YBL075C \| YLR347C \| YMR001C \| YPL042C \| YPL106C \| YPL240C |
| complex_3377 | YBR109C \| YJR065C \| YKL210W \| YMR109W \| YDL029W \| YDL125C \| YDR394W \| YHR030C \| YKL073W \| YJL095W \| YPL140C \| YHR082C \| YKL129C \| YLR096W \| YML057W |
| complex_3376 | YAR019C \| YGR092W \| YMR001C \| YBR160W \| YCR065W \| YGR108W \| YKL129C \| YNL102W \| YNL298W \| YPR111W \| YPR119W \| YIL106W \| YJL187C \| YMR036C \| YOR127W |
| complex_3375 | YBR160W \| YCR065W \| YDL028C \| YDL155W \| YGR092W \| YKL129C \| YLR131C \| YPR111W \| YPR119W \| YPR141C \| YDR356W \| YPR161C \| YIL106W \| YNL161W \| YGL058W |
| complex_3011 | YBL016W \| YLR362W \| YBL075C \| YGR092W \| YBR160W \| YCR065W \| YDL155W \| YHL007C \| YKL129C \| YDL159W \| YGR040W \| YHR018C \| YIL106W \| YPR119W \| YNL298W |
| complex_3010 | YAL005C \| YDR172W \| YDR477W \| YNL025C \| YNL209W \| YBL075C \| YPL042C \| YDL229W \| YDR385W \| YER129W \| YER165W \| YPR119W \| YJR032W \| YML068W \| YMR037C |
| complex_3372 | YBL105C \| YOL100W \| YOR231W \| YBR160W \| YCR065W \| YIL112W \| YOR063W \| YPR119W \| YPR154W \| YDR388W \| YMR109W \| YER165W \| YJL095W \| YKL129C \| YPR112C |
| complex_3370 | YBL045C \| YCR065W \| YKL126W \| YDR490C \| YMR104C \| YER165W \| YKL166C \| YGL137W \| YHR135C \| YIL033C \| YHR205W \| YPL131W \| YJL164C \| YNL027W \| YPL203W |
| complex_2288 | YBL045C \| YPR054W \| YPR191W \| YDL087C \| YDR235W \| YHR086W \| YIL061C \| YKL173W \| YLR147C \| YML046W \| YDL108W \| YFL029C \| YLR096W \| YDR432W \| YHR030C |
| complex_2283 | YDL014W \| YML010W \| YER007C-A \| YJR014W \| YER165W \| YGR159C \| YIL035C \| YGL019W \| YGL254W \| YIL118W \| YOR039W \| YIL131C \| YOL145C \| YOR061W |
| complex_2282 | YAL005C \| YBL075C \| YDL229W \| YLL024C \| YLR310C \| YPR119W \| YBL016W \| YMR139W \| YBR160W \| YHR018C \| YDR146C \| YNL209W \| YDR477W \| YDR507C \| YLR362W |
| complex_1199 | YBR160W \| YGL003C \| YMR001C \| YPR120C \| YDR212W \| YBR198C \| YIL142W \| YJL106W \| YKR036C \| YLL011W \| YNL317W \| YGR092W \| YPL042C \| YJL014W \| YJR064W |
| complex_1196 | YAL005C \| YBL016W \| YLR310C \| YNL007C \| YNL298W \| YLR362W \| YBL075C \| YBR160W \| YBR200W \| YHL007C \| YHR018C \| YKL129C \| YPR119W \| YDL159W \| YGR040W |
| complex_1195 | YBL105C \| YBR160W \| YER165W \| YJL095W \| YJR059W \| YOL100W \| YPR119W \| YER008C \| YGL003C \| YNR047W \| YMR304W \| YJL060W \| YJL187C \| YKL129C \| YMR001C |
| complex_1191 | YAL005C \| YLR310C \| YBL016W \| YBR200W \| YDL159W \| YLR362W \| YBL075C \| YNL064C \| YOR027W \| YPL042C \| YPR119W \| YGR040W \| YHR018C \| YPL240C \| YLL024C |
| complex_1190 | YAL005C \| YCL011C \| YLR310C \| YPL106C \| YBL075C \| YDL159W \| YBL016W \| YGR040W \| YDR146C \| YLL024C \| YGL158W \| YJL128C \| YJR032W \| YLR362W |
| complex_3369 | YBL075C \| YJL128C \| YLR310C \| YBR160W \| YDL155W \| YKL129C \| YLR131C \| YNL161W \| YHR018C \| YFL034C-B \| YHR102W \| YHL007C \| YLR362W \| YNL007C \| YIL106W |

| | |
|---|---|
| complex_3006 | YBR109C \| YGL019W \| YOR039W \| YOR061W \| YBR260C \| YFR024C-A \| YIL118W \| YPL140C \| YML010W \| YGL254W \| YIL035C \| YIL131C \| YML074C \| YNL189W \| YOL145C |
| complex_3367 | YBL045C \| YGR159C \| YNL307C \| YBR133C \| YER165W \| YJL187C \| YKL101W \| YBR160W \| YGL003C \| YCL024W \| YDR054C \| YDR432W \| YPR119W \| YMR304W \| YIL046W |
| complex_3004 | YAR019C \| YGR092W \| YBR160W \| YCR065W \| YKL129C \| YLR131C \| YDL155W \| YDL029W \| YHL007C \| YIL106W \| YNL161W \| YPR119W \| YKR036C \| YMR109W \| YPL042C |
| complex_3003 | YAR019C \| YGR092W \| YBL075C \| YPL042C \| YBR160W \| YDL155W \| YHR018C \| YIL106W \| YKL129C \| YPR119W \| YPR104C \| YNL161W \| YKR036C \| YPL131W \| YNL288W |
| complex_3365 | YBR160W \| YGR092W \| YDL007W \| YDR394W \| YEL037C \| YLR424W \| YDL029W \| YDL225W \| YOR117W \| YGL048C \| YLL034C \| YIL007C \| YKL145W \| YOR259C \| YLR309C |
| complex_3000 | YBL045C \| YGR092W \| YOL123W \| YDL017W \| YDR394W \| YDL029W \| YDR432W \| YER165W \| YGR159C \| YMR216C \| YOR204W \| YPR119W \| YKL166C \| YMR146C \| YGL137W |
| complex_2279 | YBR109C \| YGL019W \| YOR039W \| YOR061W \| YBR260C \| YFR024C-A \| YIL118W \| YML010W \| YGL254W \| YIL131C \| YML074C \| YIL035C \| YNL304W \| YOL145C \| YOR326W |
| complex_2276 | YAL029C \| YBR109C \| YFL005W \| YFL039C \| YGL106W \| YOR035C \| YBL105C \| YJL095W \| YOR231W \| YJR065C \| YDL029W \| YHR023W \| YOR326W \| YKL129C \| YMR109W |
| complex_2275 | YBR160W \| YCR065W \| YDL155W \| YER008C \| YGL003C \| YJL187C \| YKL129C \| YMR001C \| YNL102W \| YNL298W \| YPR111W \| YPR119W \| YGR092W \| YIL106W \| YNL068C |
| complex_2274 | YAL005C \| YLR310C \| YNL209W \| YPL106C \| YBL016W \| YIL126W \| YLR362W \| YBL075C \| YNL064C \| YDL159W \| YGR040W \| YDL229W \| YDR477W \| YJL128C \| YLL024C |
| complex_2272 | YBR160W \| YGL003C \| YGR092W \| YJR059W \| YMR001C \| YPR120C \| YDR212W \| YBR198C \| YJL164C \| YKR036C \| YNL317W \| YIL142W \| YJL106W \| YJR064W \| YJL014W |
| complex_2270 | YBR109C \| YJR065C \| YKL129C \| YMR109W \| YCR088W \| YDL029W \| YDR523C \| YDR216W \| YNL007C \| YPL140C \| YHR018C \| YKL073W \| YHR030C \| YJL095W \| YNL298W |
| complex_1189 | YBR160W \| YGL003C \| YMR001C \| YPR120C \| YDR188W \| YBR198C \| YJL106W \| YKR036C \| YNL317W \| YOR212W \| YDR212W \| YIL142W \| YJR064W \| YGR092W \| YJL014W |
| complex_1188 | YDL029W \| YDR394W \| YHR030C \| YOR259C \| YPL140C \| YDL097C \| YDL007W \| YEL037C \| YGL048C \| YHR027C \| YKL145W \| YOR117W \| YJL095W \| YKL073W \| YNL007C |
| complex_1187 | YBR160W \| YER008C \| YJL187C \| YBR200W \| YAL041W \| YGR152C \| YHL007C \| YLR229C \| YNL298W \| YPR119W \| YCR065W \| YDR379W \| YGR108W \| YKL129C \| YOR127W |
| complex_1186 | YBR065C \| YLR117C \| YLR424W \| YMR001C \| YMR213W \| YPL151C \| YPR119W \| YPR178W \| YDL030W \| YDR364C \| YDR473C \| YKL173W \| YER013W \| YGL120C \| YIR005W |
| complex_1180 | YAL005C \| YBL075C \| YDR477W \| YLL024C \| YLR310C \| YNL064C \| YOR027W \| YPL240C \| YJL128C \| YDL159W \| YBL016W \| YHR018C \| YDR216W \| YGR040W \| YLR362W |
| complex_3358 | YDR212W \| YFL033C \| YGL137W \| YHR135C \| YJL106W \| YJL164C \| YKL166C \| YNL317W \| YNL027W \| YPL203W \| YIL033C \| YMR022W \| YNL093W \| YIL142W \| YOR089C |
| complex_3353 | YCR088W \| YFL039C \| YPL204W \| YDL195W \| YGL019W \| YIL035C \| YIL131C \| YDR252W \| YPL037C \| YPR111W \| YLR399C \| YOR039W \| YHR193C \| YOL145C \| YOR061W |

| | |
|---|---|
| complex_2269 | YAL005C \| YBL075C \| YDR477W \| YLR310C \| YNL209W \| YPL106C \| YDL192W \| YDL229W \| YMR139W \| YBL016W \| YER165W \| YGL158W \| YLR113W \| YLL024C \| YLR362W |
| complex_2268 | YAL021C \| YCR093W \| YGR092W \| YNL288W \| YOR027W \| YPL042C \| YDR394W \| YDL007W \| YGL048C \| YKL145W \| YOR117W \| YOR259C \| YEL037C \| YPL096W \| YMR186W |
| complex_2267 | YBL105C \| YAL041W \| YBR160W \| YJL095W \| YOR127W \| YPR119W \| YBR031W \| YCR065W \| YJL187C \| YNL298W \| YDR216W \| YDL029W \| YFR027W \| YMR109W \| YKL129C |
| complex_2263 | YBL050W \| YGL095C \| YOL018C \| YOR036W \| YBR080C \| YDL007W \| YGL004C \| YDL047W \| YDR394W \| YGL048C \| YKL145W \| YOR117W \| YOR259C \| YDR323C \| YGR232W |
| complex_2262 | YAL024C \| YBR160W \| YNL102W \| YGL019W \| YIL131C \| YMR001C \| YNL068C \| YGR092W \| YJL187C \| YPR119W \| YML064C \| YPR191W \| YMR036C \| YNL016W \| YPR120C |
| complex_1177 | YAL041W \| YBR200W \| YGR152C \| YLR229C \| YNL298W \| YBR031W \| YBR160W \| YLR362W \| YOR127W \| YCR065W \| YPR119W \| YDR379W \| YHL007C \| YJL187C \| YKL129C |
| complex_1174 | YAL005C \| YBL075C \| YBR155W \| YJR032W \| YLR310C \| YOR027W \| YBR017C \| YDR062W \| YOR185C \| YDL229W \| YDR477W \| YNL209W \| YLL024C \| YPL106C \| YPL240C |
| complex_1173 | YGL019W \| YGL137W \| YGL254W \| YIL118W \| YIL131C \| YKL166C \| YML010W \| YML074C \| YOR039W \| YIL033C \| YJL164C \| YPL203W \| YIL035C \| YOR061W \| YOL145C |
| complex_1172 | YAL005C \| YDL229W \| YER165W \| YLR310C \| YNL007C \| YPL106C \| YPR112C \| YPR128C \| YBL105C \| YJL095W \| YOR063W \| YPR154W \| YNL209W \| YOL100W \| YOR231W |
| complex_1171 | YBR160W \| YDL108W \| YLR210W \| YBR198C \| YDR188W \| YKR036C \| YLL011W \| YNL317W \| YOR212W \| YDR212W \| YJL106W \| YFL029C \| YIL142W \| YJR064W \| YJL014W |
| complex_3347 | YAL005C \| YBR155W \| YDL229W \| YIL061C \| YNL007C \| YOR254C \| YDR477W \| YJL034W \| YHR030C \| YPR191W \| YHR079C \| YKL073W \| YLL024C \| YPL106C \| YNL027W |
| complex_3343 | YDR188W \| YBR198C \| YDR142C \| YFL033C \| YGL137W \| YJL164C \| YKL166C \| YNL317W \| YOR212W \| YPL203W \| YDR212W \| YDR075W \| YIL142W \| YJL014W \| YJR064W |
| complex_2259 | YBR160W \| YCR065W \| YDL155W \| YGR092W \| YKL129C \| YNR047W \| YCR091W \| YDR146C \| YLR131C \| YPR119W \| YIL106W \| YNL161W \| YPR111W \| YDL029W \| YMR109W |
| complex_2256 | YAR019C \| YBR160W \| YGR092W \| YBL084C \| YGL003C \| YCR034W \| YLR096W \| YCR065W \| YDL147W \| YDL155W \| YIL106W \| YPR111W \| YPR119W \| YKL129C \| YMR001C |
| complex_2250 | YBR031W \| YBR160W \| YLR362W \| YOR127W \| YCR065W \| YJL187C \| YNL102W \| YNL298W \| YBR200W \| YLR229C \| YPR119W \| YHL007C \| YKL129C \| YMR001C \| YMR109W |
| complex_1169 | YBR160W \| YER129W \| YGL011C \| YGR135W \| YML092C \| YMR314W \| YOL038W \| YOR362C \| YER012W \| YNL209W \| YDL084W \| YDR477W \| YCR057C \| YKL139W \| YMR308C |
| complex_1166 | YBL105C \| YBR160W \| YGL003C \| YJL095W \| YOL100W \| YPR119W \| YER008C \| YJL187C \| YNL102W \| YMR304W \| YKL129C \| YMR001C \| YNL068C \| YNL298W \| YPR120C |
| complex_1163 | YDL097C \| YDR394W \| YFR004W \| YHR030C \| YOR259C \| YOR261C \| YDL147W \| YDL029W \| YJR065C \| YKL073W \| YDR523C \| YJL095W \| YPL140C \| YMR109W \| YKL129C |
| complex_3334 | YBR160W \| YBL084C \| YGL003C \| YGR092W \| YJL187C \| YNL298W \| YPR120C \| YDL145C \| YMR304W \| YDR054C \| YDR328C \| YFL009W \| YIL046W \| YGL137W \| YMR001C |

| | |
|---|---|
| complex_3333 | YAL005C \| YBR160W \| YNL064C \| YBL075C \| YCR065W \| YJL187C \| YKL129C \| YCL024W \| YHR018C \| YDR054C \| YHL007C \| YIL046W \| YLR362W \| YNL298W \| YPR119W |
| complex_3332 | YBR160W \| YGL116W \| YGR092W \| YDR212W \| YGL137W \| YJL106W \| YJL164C \| YNL317W \| YOR317W \| YIL142W \| YIL033C \| YJR064W \| YKL166C \| YPL203W \| YNL027W |
| complex_3330 | YAL005C \| YLR310C \| YNL007C \| YBL016W \| YBL075C \| YDL159W \| YJR032W \| YLR362W \| YGR040W \| YDR477W \| YGL158W \| YLL039C \| YDR507C \| YIL046W \| YMR139W |
| complex_2246 | YBR160W \| YGL003C \| YJL187C \| YNL298W \| YPL115C \| YPR120C \| YDL159W \| YGR040W \| YDR212W \| YIL142W \| YJL106W \| YKR036C \| YOR212W \| YHL007C \| YLR362W |
| complex_2243 | YAR019C \| YGR092W \| YBR160W \| YGL003C \| YCR065W \| YDL155W \| YKL129C \| YDL185W \| YKL048C \| YMR304W \| YDR146C \| YPR119W \| YIL106W \| YNL068C \| YPR120C |
| complex_2242 | YAL005C \| YDL229W \| YPL106C \| YPR119W \| YFL033C \| YGL116W \| YIL033C \| YKL166C \| YJL164C \| YNL093W \| YLR216C \| YNL027W \| YNL209W \| YPL031C \| YPL203W |
| complex_2240 | YAR019C \| YDL029W \| YBR160W \| YCR065W \| YDL155W \| YKL129C \| YLR131C \| YPR111W \| YDR216W \| YER008C \| YGR092W \| YPR119W \| YHL007C \| YIL106W \| YNL161W |
| complex_1158 | YBR160W \| YGR092W \| YHL007C \| YMR001C \| YNL298W \| YBR200W \| YLR362W \| YPR119W \| YDR054C \| YHR086W \| YIL046W \| YIL106W \| YPR111W \| YJL187C \| YKL129C |
| complex_1155 | YBL045C \| YBR160W \| YIL131C \| YBL105C \| YDL195W \| YGL019W \| YOR061W \| YDR432W \| YER165W \| YGR159C \| YNL112W \| YPR119W \| YOL100W \| YOR039W \| YIL035C |
| complex_1154 | YAL005C \| YBL075C \| YBR155W \| YDR283C \| YJR032W \| YLR310C \| YOR027W \| YDL229W \| YJL138C \| YDR477W \| YNL209W \| YLL024C \| YPL106C \| YPL240C \| YLR347C |
| complex_1153 | YAL005C \| YHL007C \| YNL007C \| YBR160W \| YCR065W \| YDL155W \| YDR146C \| YGR092W \| YHR018C \| YIL106W \| YKL129C \| YLR131C \| YPR111W \| YPR119W \| YNL161W |
| complex_1152 | YAL005C \| YDL229W \| YDR477W \| YER081W \| YLL024C \| YLR310C \| YOR027W \| YPL106C \| YPL240C \| YBL075C \| YHR064C \| YAR018C \| YPR054W \| YNL209W \| YER103W |
| complex_3329 | YBL045C \| YPL140C \| YPR054W \| YPR191W \| YBL105C \| YJL095W \| YDL029W \| YHR030C \| YDR394W \| YER165W \| YGL022W \| YGR254W \| YGL137W \| YGR040W \| YOR231W |
| complex_3328 | YBL045C \| YGL137W \| YNL016W \| YDR432W \| YER165W \| YGR159C \| YIR001C \| YOR204W \| YPR119W \| YAL005C \| YOR027W \| YGL049C \| YGR162W \| YHR086W |
| complex_3327 | YAL024C \| YBR160W \| YLR113W \| YBL045C \| YCR065W \| YPR054W \| YBL105C \| YGL003C \| YDL108W \| YDR432W \| YER165W \| YPR119W \| YFL029C \| YJL060W \| YOL100W |
| complex_3326 | YBL016W \| YLR362W \| YDL159W \| YGR040W \| YDR212W \| YBR198C \| YIL142W \| YJL106W \| YNL317W \| YOR212W \| YER020W \| YFR024C-A \| YHL007C \| YHR005C \| YJR064W |
| complex_3323 | YBL105C \| YCL024W \| YBR160W \| YGL003C \| YHR158C \| YJL060W \| YJL187C \| YLR096W \| YMR001C \| YNL298W \| YPR119W \| YDR054C \| YER165W \| YMR304W \| YIL046W |
| complex_3321 | YBR160W \| YCR065W \| YDR356W \| YJL187C \| YKL129C \| YNL102W \| YNL298W \| YOR127W \| YPR119W \| YGR092W \| YHR018C \| YMR001C \| YAL041W \| YNL189W \| YOR204W |
| complex_272 | YAL005C \| YER107C \| YER165W \| YLR310C \| YNL064C \| YNL209W \| YOR027W \| YPL106C \| YPR128C \| YBL075C \| YDR477W \| YER028C \| YLL024C \| YNL041C \| YCR057C |

| | |
|---|---|
| complex_2239 | YAL024C \| YBR160W \| YPR111W \| YBL105C \| YER165W \| YOL100W \| YPR119W \| YHR030C \| YPR191W \| YHR158C \| YJL187C \| YHR193C \| YPL037C \| YLR096W \| YMR036C |
| complex_2238 | YBL045C \| YCR065W \| YGR159C \| YOL100W \| YBL105C \| YJL095W \| YOR231W \| YPL151C \| YBR160W \| YLL019C \| YPR119W \| YDR432W \| YER165W \| YKL025C |
| complex_2236 | YAL005C \| YCL024W \| YMR001C \| YNL007C \| YBR160W \| YCR065W \| YHR018C \| YJL187C \| YKL129C \| YNL102W \| YPR119W \| YBR200W \| YDR054C \| YIL046W \| YNL298W |
| complex_2598 | YAR019C \| YBR160W \| YGL003C \| YCR065W \| YDL028C \| YDR356W \| YDL155W \| YPR119W \| YGR092W \| YIL106W \| YNL068C \| YPR111W \| YJL060W \| YKR095W \| YKL129C |
| complex_2233 | YBR160W \| YCR065W \| YDR146C \| YGR238C \| YHR158C \| YJL187C \| YKL129C \| YLR096W \| YMR001C \| YNL298W \| YPR119W \| YDR054C \| YCL024W \| YER165W \| YIL046W |
| complex_2595 | YAR019C \| YBR160W \| YGL003C \| YNL102W \| YCR065W \| YDL155W \| YDR507C \| YKR048C \| YMR139W \| YPR119W \| YGR092W \| YIL106W \| YPR111W \| YKL129C \| YMR001C |
| complex_2232 | YBL075C \| YLR362W \| YNL298W \| YBR160W \| YJL187C \| YNL102W \| YBR200W \| YPR119W \| YCR065W \| YGR092W \| YHR018C \| YGR270W \| YHL007C \| YKL129C \| YMR001C |
| complex_2230 | YCL024W \| YLR314C \| YNL189W \| YCR002C \| YDR507C \| YDL225W \| YDR148C \| YHR107C \| YBR160W \| YPR119W \| YER165W \| YLR335W \| YMR001C \| YJR076C \| YMR304W |
| complex_2592 | YBR160W \| YAL019W \| YER008C \| YGL003C \| YGR238C \| YHR158C \| YIL112W \| YJL187C \| YLR096W \| YPL209C \| YDL155W \| YDR146C \| YLR131C \| YPR119W \| YMR304W |
| complex_2591 | YBR160W \| YAL019W \| YER008C \| YGL003C \| YJL187C \| YMR001C \| YNL298W \| YPR119W \| YPR120C \| YCL024W \| YKR048C \| YMR304W \| YER165W \| YHL035C \| YJL060W |
| complex_2590 | YCR065W \| YBR160W \| YDR379W \| YGR152C \| YLR229C \| YPR119W \| YGR108W \| YAL041W \| YOR127W \| YHL007C \| YKL129C \| YIL106W \| YJL187C \| YNL298W \| YJR059W |
| complex_1147 | YAL005C \| YAR019C \| YNL007C \| YPR111W \| YBR160W \| YLR131C \| YCR065W \| YDL155W \| YGR092W \| YHL007C \| YHR018C \| YKL129C \| YIL106W \| YPR119W \| YNL161W |
| complex_1144 | YBR160W \| YDL017W \| YLR210W \| YGL137W \| YDR188W \| YKR036C \| YLL011W \| YNL317W \| YOR212W \| YDR212W \| YJL106W \| YGR092W \| YIL142W \| YJR064W \| YJL014W |
| complex_269 | YAL005C \| YBR085W \| YER081W \| YER107C \| YER165W \| YLR310C \| YNL064C \| YNL209W \| YOR027W \| YPL106C \| YBL016W \| YLR362W \| YBL075C \| YLL024C \| YNL041C |
| complex_3312 | YBR066C \| YDR477W \| YMR001C \| YPR120C \| YBR160W \| YGL003C \| YDL155W \| YKL129C \| YNL068C \| YDR146C \| YPR119W \| YER129W \| YGR092W \| YIL106W \| YPR111W |
| complex_3311 | YBR160W \| YGL048C \| YGR092W \| YKL145W \| YDL007W \| YER122C \| YFR010W \| YDR069C \| YDR394W \| YOR117W \| YOR259C \| YGL137W \| YNL250W \| YIL007C \| YLL034C |
| complex_3310 | YAL005C \| YBL075C \| YLL024C \| YLR310C \| YMR139W \| YBL016W \| YDL159W \| YGL158W \| YLR362W \| YBL045C \| YPR054W \| YGL137W \| YGR040W \| YPR034W \| YOR027W |
| complex_2229 | YAR019C \| YER008C \| YGL003C \| YMR001C \| YBR160W \| YDL155W \| YGR092W \| YIL106W \| YJL187C \| YPR119W \| YPR120C \| YDL185W \| YMR304W \| YKR048C \| YNL161W |
| complex_2226 | YAL041W \| YOR212W \| YBR160W \| YER008C \| YGL003C \| YHL007C \| YLR362W \| YPR120C \| YMR304W \| YLR229C \| YJL187C \| YNL298W \| YMR001C \| YNL161W \| YPR165W |

| | |
|---|---|
| complex_2225 | YBL105C \| YBR160W \| YER165W \| YIL112W \| YJL095W \| YOL100W \| YOR231W \| YPR119W \| YCR065W \| YGL003C \| YGR159C \| YKL025C \| YMR109W \| YKL129C \| YDL029W |
| complex_2587 | YAL005C \| YOR231W \| YBL045C \| YCR065W \| YMR104C \| YBL105C \| YJL095W \| YBR160W \| YER129W \| YDR477W \| YDR490C \| YER165W \| YPR119W \| YKL129C \| YOL100W |
| complex_2224 | YAL005C \| YJR059W \| YMR001C \| YAR019C \| YHR018C \| YCR065W \| YBR160W \| YDL155W \| YGR092W \| YIL106W \| YPR119W \| YPR111W \| YOR204W \| YKL129C \| YLR347C |
| complex_2586 | YAL029C \| YBR081C \| YBR109C \| YFL039C \| YGL106W \| YHR023W \| YLR249W \| YOR035C \| YGR252W \| YBR198C \| YDR145W \| YDR216W \| YGR274C \| YDR448W \| YHR079C |
| complex_2585 | YAL005C \| YLR310C \| YNL064C \| YPL106C \| YAL021C \| YCR093W \| YGR092W \| YNL288W \| YPL042C \| YBL016W \| YBL075C \| YDR477W \| YJL005W \| YLL024C \| YNL209W |
| complex_2222 | YAR019C \| YGR092W \| YPL042C \| YBL075C \| YDR477W \| YNL025C \| YBR160W \| YCR065W \| YDL155W \| YHR018C \| YKL129C \| YPR119W \| YIL106W \| YKR036C \| YPR111W |
| complex_2584 | YAR019C \| YHR169W \| YBR160W \| YBL084C \| YPR111W \| YCR065W \| YDL097C \| YDR394W \| YFR004W \| YGL048C \| YOR261C \| YDL147W \| YGR092W \| YIL106W \| YMR001C |
| complex_2221 | YBR160W \| YBR200W \| YCR065W \| YER008C \| YHL007C \| YJL187C \| YKL129C \| YMR001C \| YNL102W \| YNL298W \| YPR119W \| YDR379W \| YGR152C \| YLR229C \| YOR127W |
| complex_2582 | YAL005C \| YIL061C \| YLR310C \| YNL209W \| YDR477W \| YLL024C \| YER028C \| YNL096C \| YPL106C \| YGL058W \| YBL016W \| YOR194C \| YGL158W \| YKL058W \| YMR139W |
| complex_1137 | YDL007W \| YDL029W \| YGL004C \| YPL140C \| YDL047W \| YDR394W \| YGL048C \| YHR030C \| YKL145W \| YOR117W \| YOR259C \| YEL037C \| YPR191W \| YIL007C \| YPR178W |
| complex_1499 | YAL005C \| YBR155W \| YDR477W \| YER081W \| YER129W \| YJR032W \| YNL209W \| YPL106C \| YBL075C \| YDL229W \| YPL240C \| YDR194C \| YGL035C \| YLL024C \| YLR347C |
| complex_1136 | YAL005C \| YBL075C \| YLR310C \| YNL064C \| YPR119W \| YBL016W \| YHL007C \| YBR160W \| YCR065W \| YHR018C \| YKL129C \| YNL298W \| YBR200W \| YJL187C \| YLR362W |
| complex_1498 | YCL059C \| YLR129W \| YLR222C \| YLR409C \| YCR057C \| YGL011C \| YGR135W \| YOL038W \| YDL188C \| YML092C \| YMR314W \| YNL178W \| YOR362C \| YDR283C \| YIL035C |
| complex_1135 | YAL005C \| YDR477W \| YER129W \| YLL024C \| YLR310C \| YLR362W \| YNL025C \| YOR027W \| YBL075C \| YDL229W \| YPL240C \| YPR119W \| YNL209W \| YPL042C \| YPL106C |
| complex_1134 | YBR160W \| YIL046W \| YLR362W \| YNL298W \| YOR127W \| YBR200W \| YAL041W \| YLR229C \| YCL024W \| YJL187C \| YCR002C \| YNL007C \| YHL007C \| YJR076C \| YLR314C |
| complex_1133 | YBR160W \| YCL024W \| YDR507C \| YGR092W \| YKL101W \| YMR001C \| YNL298W \| YCR002C \| YDL225W \| YHR107C \| YJR076C \| YDR218C \| YGL127C \| YJL187C \| YLR314C |
| complex_1495 | YBL045C \| YCR065W \| YNR047W \| YBR160W \| YBL105C \| YDL155W \| YGR092W \| YIL106W \| YJR059W \| YKL116C \| YKL129C \| YOL100W \| YPR111W \| YPR119W \| YER165W |
| complex_1132 | YAL005C \| YBL075C \| YLR310C \| YNL064C \| YOR027W \| YPL106C \| YBL050W \| YGL095C \| YOR036W \| YDR323C \| YGR040W \| YHR018C \| YLL024C \| YLR362W \| YPL240C |
| complex_1131 | YBL105C \| YBR160W \| YER008C \| YGL003C \| YOL100W \| YGR092W \| YDR507C \| YKR048C \| YPR119W \| YMR304W \| YER165W \| YMR001C \| YJL060W \| YJL187C \| YCL024W |

| | |
|---|---|
| complex_1491 | YBL105C \| YBR160W \| YJL095W \| YOL100W \| YOR231W \| YPL151C \| YPR119W \| YPR120C \| YBR065C \| YER008C \| YGL003C \| YMR304W \| YJL187C \| YMR001C \| YKR048C |
| complex_3308 | YBR160W \| YJR059W \| YDR188W \| YBR198C \| YDR142C \| YDR379W \| YGL137W \| YJL106W \| YNL317W \| YOR212W \| YDR212W \| YDR075W \| YIL142W \| YJR064W \| YJL014W |
| complex_1490 | YAL005C \| YLR310C \| YNL064C \| YPL106C \| YBL075C \| YDL159W \| YBL016W \| YGR040W \| YGL158W \| YHR193C \| YPL037C \| YJL128C \| YLL024C \| YLR113W \| YLR362W |
| complex_3304 | YDL175C \| YIL079C \| YDR394W \| YDL007W \| YGL048C \| YKL145W \| YOR117W \| YOR259C \| YDR432W \| YFR010W \| YMR216C \| YGL137W \| YNL250W \| YGR159C \| YOR204W |
| complex_3303 | YAL005C \| YBL075C \| YDR477W \| YGL158W \| YLR113W \| YLR310C \| YNL209W \| YOR027W \| YPL106C \| YDL229W \| YDR356W \| YPR119W \| YER129W \| YLL024C \| YPL240C |
| complex_3302 | YBL105C \| YBR160W \| YER165W \| YIL112W \| YJL095W \| YOL100W \| YOR231W \| YPR119W \| YCR065W \| YGR108W \| YJL187C \| YKL129C \| YBR200W \| YMR109W \| YNL298W |
| complex_3301 | YBL045C \| YCR065W \| YLR362W \| YBR133C \| YER165W \| YHL007C \| YBR160W \| YGL003C \| YMR001C \| YNL298W \| YPR119W \| YIR005W \| YJL060W \| YJL187C \| YCL024W |
| complex_2219 | YAL021C \| YAR019C \| YDR188W \| YGR092W \| YKR036C \| YPL042C \| YGL137W \| YLL011W \| YNL317W \| YOR212W \| YDR212W \| YIL142W \| YJR064W \| YMR037C \| YNL178W |
| complex_2217 | YBR109C \| YGL019W \| YLR212C \| YOR039W \| YOR061W \| YBR034C \| YGL254W \| YIL035C \| YIL118W \| YIL131C \| YML010W \| YML074C \| YOL123W \| YOL145C \| YOR326W |
| complex_2579 | YAL005C \| YDR477W \| YER081W \| YNL209W \| YPL106C \| YDL229W \| YGL179C \| YDR012W \| YER129W \| YDR194C \| YGL035C \| YDR216W \| YGL158W \| YLR347C \| YMR104C |
| complex_2216 | YBR160W \| YGR092W \| YNL161W \| YCR065W \| YDL155W \| YKL129C \| YDR146C \| YLR131C \| YPR119W \| YER165W \| YHR102W \| YHL007C \| YIL106W \| YPR111W \| YKL145W |
| complex_2215 | YAR019C \| YGR092W \| YHR018C \| YIL106W \| YBR109C \| YKL129C \| YCR065W \| YBR160W \| YDL155W \| YDR146C \| YLR131C \| YPR119W \| YNL161W \| YPR111W \| YOR358W |
| complex_2577 | YBR109C \| YGL019W \| YOR039W \| YOR061W \| YBR160W \| YIL035C \| YIL131C \| YGL254W \| YIL118W \| YML010W \| YML074C \| YNL307C \| YIL033C \| YKL101W \| YOL145C |
| complex_2576 | YBL105C \| YBR160W \| YER008C \| YJL095W \| YOL100W \| YBR109C \| YMR109W \| YDR356W \| YIL149C \| YKR095W \| YLR212C \| YNL126W \| YPR119W \| YPR141C \| YKL129C |
| complex_2575 | YAL005C \| YBR155W \| YDR477W \| YER081W \| YIL035C \| YLR310C \| YPL106C \| YBL075C \| YPR119W \| YDL229W \| YER129W \| YLL024C \| YNL209W \| YOR061W \| YOR185C |
| complex_2574 | YBL105C \| YBR160W \| YJL095W \| YOL100W \| YPR119W \| YGL003C \| YCR065W \| YDR432W \| YGR159C \| YOR204W \| YER165W \| YJL060W \| YKL129C \| YJR059W \| YMR109W |
| complex_2211 | YAR019C \| YGR092W \| YBR160W \| YCR065W \| YDL155W \| YGL003C \| YIL106W \| YJL187C \| YKL129C \| YMR001C \| YNL102W \| YNL298W \| YPR111W \| YPR119W \| YGL240W |
| complex_2210 | YAR019C \| YGR092W \| YML064C \| YBR160W \| YCR065W \| YDL155W \| YGL003C \| YHL007C \| YKL129C \| YDL029W \| YIL106W \| YMR001C \| YNL161W \| YPR111W \| YPR119W |
| complex_2571 | YAL047C \| YDR356W \| YHR172W \| YIL149C \| YLR212C \| YNL126W \| YBR109C \| YBR160W \| YFL037W \| YKR095W \| YML085C \| YOR257W \| YKL129C \| YPR119W \| YPR141C |

| | |
|---|---|
| complex_1128 | YBL075C \| YBR085W \| YBR155W \| YER081W \| YIL061C \| YLR310C \| YOR254C \| YEL030W \| YPL140C \| YJL034W \| YJL095W \| YHR030C \| YKL073W \| YNL007C \| YNL027W |
| complex_1127 | YBL045C \| YBL105C \| YCR065W \| YGR159C \| YBR160W \| YGL003C \| YJL187C \| YOL100W \| YER165W \| YOR231W \| YPR119W \| YMR304W \| YJL060W \| YJL095W \| YKL025C |
| complex_1489 | YDR490C \| YHR205W \| YER165W \| YKL126W \| YML010W \| YOR061W \| YGL019W \| YGL254W \| YIL118W \| YIL131C \| YLR399C \| YML074C \| YOR039W \| YIL035C \| YOL145C |
| complex_1126 | YBL105C \| YPL151C \| YBR065C \| YLR117C \| YLR424W \| YMR001C \| YMR213W \| YPR119W \| YPR178W \| YER165W \| YGR092W \| YKL173W \| YGL120C \| YKL025C \| YJL187C |
| complex_1487 | YDR364C \| YKL095W \| YMR213W \| YER013W \| YER003C \| YKL173W \| YLR117C \| YMR001C \| YFL017W-A \| YPL151C \| YGL120C \| YJR064W \| YKR024C \| YMR240C \| YNL189W |
| complex_1486 | YBR109C \| YGL019W \| YOR039W \| YOR061W \| YGL254W \| YIL035C \| YIL118W \| YIL131C \| YML010W \| YML074C \| YHR023W \| YIL061C \| YOR326W \| YOL145C |
| complex_1485 | YAL005C \| YBL075C \| YBR155W \| YDR507C \| YLR310C \| YOR027W \| YDL229W \| YDR477W \| YNL209W \| YJR032W \| YPL240C \| YLL024C \| YPL106C \| YLR347C \| YMR139W |
| complex_1121 | YBL045C \| YCR065W \| YGR159C \| YOL100W \| YBL105C \| YBR160W \| YER165W \| YJL095W \| YOR231W \| YPR119W \| YGL003C \| YMR001C \| YDR432W \| YJL060W \| YKL025C |
| complex_1481 | YBL105C \| YJL095W \| YDL175C \| YIL079C \| YOR204W \| YPR191W \| YDR432W \| YER165W \| YGL173C \| YGR159C \| YKL139W \| YPR119W \| YFL029C \| YPR054W \| YHR030C |
| complex_1480 | YBR160W \| YGL003C \| YGL048C \| YGR092W \| YKL145W \| YMR001C \| YNL068C \| YPR120C \| YDR394W \| YDL007W \| YOR117W \| YOR259C \| YEL037C \| YER012W \| YNL016W |
| complex_2208 | YBL084C \| YGL003C \| YBR160W \| YDL155W \| YER008C \| YJL187C \| YLR131C \| YNL068C \| YPR120C \| YDL147W \| YGR092W \| YMR001C \| YMR304W \| YIL106W \| YPR119W |
| complex_2207 | YAL005C \| YDL229W \| YLR310C \| YNL064C \| YPL106C \| YBL016W \| YBL075C \| YPR119W \| YDR356W \| YFL029C \| YPR054W \| YHR018C \| YLL024C \| YOR027W \| YLR362W |
| complex_2568 | YBR160W \| YDL155W \| YGL003C \| YIL106W \| YNL068C \| YNL102W \| YPR119W \| YPR120C \| YDR146C \| YGR092W \| YMR001C \| YHL035C \| YJL187C \| YOR195W \| YPR111W |
| complex_2566 | YBR160W \| YGL003C \| YGR092W \| YMR001C \| YNL317W \| YPR120C \| YDL108W \| YFL029C \| YDR188W \| YGL137W \| YKR036C \| YLL011W \| YDR212W \| YIL142W \| YPR054W |
| complex_2203 | YBR160W \| YCR065W \| YER008C \| YGL003C \| YJL187C \| YKL129C \| YNL102W \| YNL298W \| YPR119W \| YDL155W \| YGR092W \| YMR001C \| YHL007C \| YIL106W \| YLR210W |
| complex_2564 | YBR133C \| YER165W \| YJL187C \| YKL101W \| YBR160W \| YGL003C \| YMR001C \| YPR120C \| YCL024W \| YPR119W \| YMR304W \| YJL060W \| YKR048C \| YNL298W \| YNL307C |
| complex_2201 | YBR160W \| YGL003C \| YGR238C \| YHR158C \| YJL187C \| YKL129C \| YLR096W \| YMR001C \| YNL068C \| YNL102W \| YPR119W \| YPR120C \| YCR008W \| YDR146C \| YPL031C |
| complex_2200 | YBR109C \| YGL019W \| YJR065C \| YMR109W \| YOR039W \| YOR061W \| YOR326W \| YDL195W \| YIL131C \| YER148W \| YLR399C \| YIL118W \| YML010W \| YOL004W \| YIL035C |
| complex_2562 | YBR160W \| YER008C \| YGL003C \| YHL007C \| YMR001C \| YNL298W \| YPR120C \| YDR212W \| YIL142W \| YOR212W \| YMR304W \| YLR362W \| YJL187C \| YLR229C \| YNL161W |

| | |
|---|---|
| complex_2561 | YDL029W \| YOR212W \| YDL097C \| YDR142C \| YFR004W \| YHR030C \| YOR261C \| YDL147W \| YDR188W \| YJR064W \| YNL317W \| YDR523C \| YNL037C \| YJL014W \| YPL140C |
| complex_1119 | YBR160W \| YCL024W \| YMR001C \| YNL298W \| YCR002C \| YDR507C \| YHR107C \| YLR314C \| YKL101W \| YER165W \| YIL046W \| YDR054C \| YJL187C \| YNL007C \| YJR076C |
| complex_2560 | YAL024C \| YBR160W \| YDL028C \| YGL003C \| YMR001C \| YDL155W \| YNL161W \| YER165W \| YPR111W \| YPR119W \| YGR092W \| YAR019C \| YIL106W \| YJL060W \| YLR131C |
| complex_1479 | YBR160W \| YER008C \| YHL007C \| YIL046W \| YJL187C \| YOR127W \| YBR200W \| YAL041W \| YPR119W \| YCL024W \| YDR054C \| YKL129C \| YNL298W \| YLR229C \| YLR362W |
| complex_1478 | YAL005C \| YBL075C \| YDR477W \| YLR310C \| YMR104C \| YNL064C \| YNL209W \| YOR027W \| YPL106C \| YDL229W \| YHR018C \| YDR490C \| YLL024C \| YPL240C \| YPR119W |
| complex_1475 | YDL195W \| YGL019W \| YIL131C \| YOR061W \| YER148W \| YFL039C \| YLR399C \| YOR039W \| YIL118W \| YOL004W \| YHR023W \| YIL035C \| YPL131W \| YOL145C \| YOR326W |
| complex_1112 | YBL045C \| YJL095W \| YPR191W \| YDL087C \| YDR235W \| YHR086W \| YIL061C \| YKL173W \| YLR147C \| YML046W \| YPR182W \| YHR030C \| YPL140C \| YNL147W \| YPR054W |
| complex_1111 | YDR188W \| YDR142C \| YDR212W \| YGL137W \| YJR064W \| YKR036C \| YLL011W \| YNL317W \| YOR212W \| YBR198C \| YDR075W \| YFL033C \| YJL164C \| YIL142W \| YJL014W |
| complex_1472 | YAR019C \| YGR092W \| YHR169W \| YPR111W \| YBR160W \| YCR065W \| YDL155W \| YGL003C \| YJL187C \| YKL129C \| YNL102W \| YIL106W \| YPR119W \| YMR001C \| YNL298W |
| complex_593 | YAL005C \| YDL229W \| YER081W \| YER165W \| YJL187C \| YLL024C \| YLR310C \| YNL007C \| YNL041C \| YNL209W \| YPL106C \| YPR119W \| YPR128C \| YCL024W \| YDR146C |
| complex_2556 | YBL105C \| YOR231W \| YPL151C \| YPR119W \| YPR120C \| YBR160W \| YGL003C \| YJL187C \| YKL129C \| YNL068C \| YNL102W \| YNL298W \| YJL095W \| YMR001C \| YOL100W |
| complex_2552 | YAL005C \| YLR310C \| YPL106C \| YBL016W \| YDL159W \| YDR388W \| YGL158W \| YHR016C \| YLR362W \| YBL075C \| YNL064C \| YGR040W \| YDR477W \| YLL024C \| YNL209W |
| complex_2551 | YAL005C \| YLR310C \| YNL064C \| YPL106C \| YBL016W \| YBR200W \| YDL159W \| YLR362W \| YBL075C \| YPR119W \| YGR040W \| YHL007C \| YLL024C \| YMR139W \| YNL209W |
| complex_2550 | YAL017W \| YBR160W \| YAL024C \| YBL105C \| YCR065W \| YDL025C \| YDR432W \| YER165W \| YGR159C \| YPR119W \| YPR191W \| YKL025C \| YMR036C \| YOL045W \| YOL100W |
| complex_1107 | YAL005C \| YBL075C \| YBR155W \| YGR040W \| YLR310C \| YLR362W \| YOR027W \| YBL016W \| YDL159W \| YDL229W \| YDR477W \| YJR032W \| YPL240C \| YLL024C \| YPL106C |
| complex_1102 | YAL024C \| YBR160W \| YGR238C \| YJL187C \| YBL105C \| YJL095W \| YLR096W \| YCR065W \| YDR146C \| YPR119W \| YER165W \| YHR158C \| YKL129C \| YNL298W \| YOL100W |
| complex_1101 | YBR160W \| YCL024W \| YCR002C \| YDR507C \| YHR107C \| YNL298W \| YFR027W \| YPR119W \| YKL101W \| YMR001C \| YIL112W \| YJL187C \| YJR076C \| YLR314C \| YKL129C |
| complex_1100 | YAL005C \| YNL308C \| YBR155W \| YCL043C \| YER165W \| YDL229W \| YDR356W \| YDR477W \| YGL070C \| YPL106C \| YLL024C \| YER081W \| YNL209W \| YLR310C \| YOR185C |
| complex_229 | YAL005C \| YBR155W \| YDR477W \| YER081W \| YLL024C \| YLR310C \| YNL007C \| YNL175C \| YNL209W \| YNL308C \| YPL106C \| YEL030W \| YDL229W \| YGL070C \| YER028C |

| | |
|---|---|
| complex_228 | YAL005C \| YBL075C \| YDL229W \| YER081W \| YGR159C \| YLL024C \| YLR310C \| YNL007C \| YNL209W \| YOR027W \| YPR128C \| YDR477W \| YER028C \| YPL106C \| YNL041C |
| complex_227 | YDL030W \| YLL036C \| YLR117C \| YMR213W \| YDR378C \| YER146W \| YLR275W \| YNL147W \| YGL120C \| YKL173W \| YPL151C \| YPR182W \| YMR001C \| YDR356W \| YOR159C |
| complex_588 | YDL007W \| YFR004W \| YDL097C \| YDR394W \| YGL048C \| YHR030C \| YKL145W \| YOR117W \| YOR259C \| YOR261C \| YDL147W \| YDL029W \| YDR507C \| YGR092W \| YMR139W |
| complex_587 | YAR019C \| YGR092W \| YDL007W \| YFR004W \| YDL097C \| YGL048C \| YGR262C \| YKL145W \| YOR117W \| YOR259C \| YOR261C \| YDL147W \| YDL029W \| YDR394W \| YGL137W |
| complex_586 | YAL005C \| YBL075C \| YER165W \| YLL024C \| YLR310C \| YNL064C \| YNL209W \| YOR027W \| YBL050W \| YDR189W \| YLR026C \| YDR477W \| YER028C \| YPL106C \| YNL041C |
| complex_584 | YAL005C \| YBR085W \| YDL229W \| YER165W \| YLR310C \| YNL007C \| YOR027W \| YOR388C \| YPL106C \| YBL075C \| YLR362W \| YDL159W \| YLL024C \| YNL041C \| YNL064C |
| complex_582 | YAL005C \| YBR085W \| YBR155W \| YER081W \| YIL061C \| YLL024C \| YLR310C \| YNL007C \| YNL209W \| YDR146C \| YEL030W \| YPL106C \| YDR477W \| YHR102W \| YNL161W |
| complex_581 | YAL005C \| YDL229W \| YER081W \| YER165W \| YLL024C \| YLR310C \| YNL007C \| YNL209W \| YPR128C \| YDR477W \| YLR096W \| YER028C \| YPL106C \| YHL002W \| YNL041C |
| complex_2549 | YDL087C \| YIL061C \| YKL012W \| YKL173W \| YDL208W \| YPR178W \| YDR416W \| YLR117C \| YMR001C \| YPL151C \| YGL120C \| YHR086W \| YNL245C \| YLR147C \| YNL147W |
| complex_2548 | YAL005C \| YLR310C \| YNL209W \| YBL016W \| YGL158W \| YLR362W \| YBL075C \| YDL159W \| YGR040W \| YDR477W \| YLL024C \| YLL039C \| YMR139W \| YJL128C \| YJR032W |
| complex_2545 | YBL016W \| YJL187C \| YBR160W \| YBR200W \| YIL106W \| YIL112W \| YOR127W \| YPR119W \| YGR152C \| YLR229C \| YDL159W \| YLR362W \| YHL007C \| YKL129C \| YNL298W |
| complex_2544 | YAL005C \| YDR477W \| YLR362W \| YNL209W \| YNL298W \| YBR160W \| YCR065W \| YGR108W \| YHR018C \| YJL187C \| YKL129C \| YPR119W \| YBR200W \| YER129W \| YHL007C |
| complex_2543 | YBR160W \| YJL187C \| YLR131C \| YDR356W \| YPR119W \| YFL008W \| YDL074C \| YFR031C \| YMR001C \| YFL037W \| YJL074C \| YLR086W \| YML085C \| YOR195W \| YPR141C |
| complex_2541 | YAL024C \| YBR160W \| YNR047W \| YBL045C \| YCR065W \| YBL105C \| YGL003C \| YDL108W \| YFL029C \| YER165W \| YPR119W \| YJL060W \| YMR036C \| YOL100W \| YPR054W |
| complex_2540 | YAL005C \| YLR310C \| YPL106C \| YBL016W \| YDL159W \| YER020W \| YHR005C \| YBL075C \| YLR362W \| YGR040W \| YDR477W \| YLL024C \| YER036C \| YNL064C \| YNL209W |
| complex_1458 | YAL005C \| YBL075C \| YBR155W \| YBR274W \| YER081W \| YFL016C \| YNL209W \| YOR222W \| YJR045C \| YEL030W \| YHL007C \| YNL161W \| YLR131C \| YFL034C-B \| YHR102W |
| complex_1457 | YBR160W \| YNL102W \| YGL019W \| YGL254W \| YIL035C \| YIL118W \| YIL131C \| YML010W \| YML074C \| YNL068C \| YOR039W \| YOR061W \| YMR001C \| YPR119W \| YOL145C |
| complex_1456 | YBR065C \| YLR117C \| YLR424W \| YMR001C \| YMR213W \| YPR178W \| YDL030W \| YDR283C \| YKL173W \| YDR364C \| YDR473C \| YER013W \| YGL120C \| YPL151C \| YIR005W |
| complex_1455 | YBL045C \| YBL105C \| YCR065W \| YBR160W \| YNL102W \| YMR001C \| YER165W \| YJR059W \| YPR119W \| YHR030C \| YJL095W \| YJL187C \| YKL129C \| YNL298W \| YOL100W |

| | |
|---|---|
| complex_1454 | YAL005C \| YBL075C \| YJL128C \| YJR032W \| YLL024C \| YLR310C \| YNL007C \| YDL159W \| YBL016W \| YDR477W \| YGL158W \| YLL039C \| YGR040W \| YLR113W \| YLR362W |
| complex_1453 | YDR432W \| YER165W \| YHR086W \| YIL061C \| YMR290C \| YPR119W \| YER133W \| YJL033W \| YJR093C \| YKL059C \| YKR002W \| YNL317W \| YPR107C \| YGL049C \| YNL016W |
| complex_1452 | YAR019C \| YBR160W \| YCR065W \| YDL155W \| YDL185W \| YKL048C \| YDR146C \| YLR131C \| YPR119W \| YGR092W \| YHR018C \| YIL106W \| YNL161W \| YPR111W \| YKL129C |
| complex_578 | YAL005C \| YDL229W \| YDR356W \| YDR477W \| YER081W \| YER165W \| YLL024C \| YLR310C \| YNL007C \| YNL041C \| YNL209W \| YPL106C \| YPR128C \| YER028C \| YPR161C |
| complex_577 | YAL005C \| YDL229W \| YER081W \| YER165W \| YLL024C \| YLR310C \| YNL007C \| YOR027W \| YPL106C \| YPR128C \| YDL101C \| YPL153C \| YDR146C \| YNL209W \| YNL041C |
| complex_215 | YAL005C \| YBR155W \| YER107C \| YLR310C \| YNL064C \| YNL175C \| YNL308C \| YBL075C \| YDR477W \| YEL030W \| YLL024C \| YNL209W \| YER028C \| YGL070C \| YPL106C |
| complex_576 | YAL005C \| YBL075C \| YBR085W \| YBR155W \| YER081W \| YER165W \| YJR032W \| YLR310C \| YLR362W \| YNL041C \| YNL064C \| YPR119W \| YDR146C \| YEL030W |
| complex_574 | YAL005C \| YDL229W \| YER081W \| YER165W \| YLL024C \| YLR310C \| YNL007C \| YNL209W \| YPR119W \| YPR128C \| YCR091W \| YNR047W \| YDR146C \| YPL106C \| YNL041C |
| complex_573 | YAL005C \| YBR085W \| YER081W \| YER165W \| YLR310C \| YNL007C \| YNL209W \| YOR027W \| YPL106C \| YBL075C \| YLR362W \| YHL007C \| YLL024C \| YNL041C \| YNL064C |
| complex_572 | YAL005C \| YER081W \| YER165W \| YLL024C \| YLR310C \| YOR027W \| YOR254C \| YPR128C \| YDR477W \| YMR104C \| YNL209W \| YER028C \| YJL034W \| YKL073W \| YNL041C |
| complex_570 | YAL005C \| YDL229W \| YER081W \| YER165W \| YLL024C \| YLR310C \| YNL007C \| YOR027W \| YPL106C \| YPR128C \| YDR146C \| YNL209W \| YJL128C \| YLR113W \| YNL041C |
| complex_2539 | YAL005C \| YLR310C \| YAL021C \| YDR477W \| YOR027W \| YPL042C \| YBL075C \| YNL025C \| YNL064C \| YLL016W \| YIL013C \| YLL024C \| YMR037C \| YNL098C \| YOR101W |
| complex_2899 | YBL045C \| YCR065W \| YDL108W \| YPR054W \| YBR160W \| YBL105C \| YFL029C \| YGL003C \| YDL155W \| YDR146C \| YPR119W \| YER165W \| YJL060W \| YLR113W \| YOL100W |
| complex_2535 | YAL005C \| YBL016W \| YGL158W \| YLR310C \| YNL064C \| YDL159W \| YNL298W \| YBL075C \| YBR160W \| YHR018C \| YKL129C \| YPR119W \| YBR200W \| YGR040W \| YLR362W |
| complex_2897 | YBL105C \| YDL029W \| YJL095W \| YBR031W \| YBR160W \| YOR127W \| YCR065W \| YNL298W \| YDR216W \| YFR027W \| YER165W \| YPR119W \| YKL129C \| YMR109W \| YOL100W |
| complex_2534 | YBR160W \| YDL155W \| YER008C \| YGL003C \| YGR092W \| YJL187C \| YLR131C \| YLR210W \| YNL298W \| YPL209C \| YPR119W \| YMR304W \| YIL046W \| YHL035C \| YMR001C |
| complex_2895 | YBR160W \| YER008C \| YFL009W \| YGL003C \| YNL068C \| YNL298W \| YPL209C \| YPR120C \| YDL155W \| YMR304W \| YGR092W \| YMR001C \| YPR119W \| YJL187C \| YML092C |
| complex_2532 | YBR160W \| YGR092W \| YKL145W \| YDL007W \| YER122C \| YFR010W \| YOR117W \| YDL145C \| YGL137W \| YDR394W \| YDL029W \| YGL048C \| YOR259C \| YNL250W \| YIL007C |
| complex_2531 | YAL005C \| YLR310C \| YBL075C \| YJL128C \| YLR362W \| YNL064C \| YNR031C \| YOR027W \| YGR040W \| YPL240C \| YHR018C \| YLL024C \| YPL106C \| YMR022W \| YPR119W |

| | |
|---|---|
| complex_2530 | YBR160W \| YER008C \| YGL003C \| YNR047W \| YOL100W \| YPL209C \| YPR119W \| YPR120C \| YMR304W \| YHL035C \| YJL060W \| YJL187C \| YKR048C \| YMR001C \| YNL298W |
| complex_2892 | YBL045C \| YPR054W \| YPR191W \| YDL017W \| YDL029W \| YPL140C \| YDR394W \| YKL073W \| YOR027W \| YGL137W \| YHR030C \| YLR216C \| YJL095W \| YJL034W \| YPR178W |
| complex_2891 | YAL005C \| YBR160W \| YBL105C \| YER165W \| YHR018C \| YJL095W \| YOL100W \| YPR119W \| YCR065W \| YJR059W \| YKL129C \| YDR477W \| YDL192W \| YER129W \| YGL035C |
| complex_1449 | YAL005C \| YJL128C \| YLR310C \| YNL007C \| YBL016W \| YDL229W \| YGL158W \| YLL024C \| YPL106C \| YDR477W \| YHR018C \| YPR119W \| YLR113W \| YLR248W \| YNL209W |
| complex_1441 | YDL007W \| YDR394W \| YEL037C \| YDL029W \| YPL140C \| YDL047W \| YHR030C \| YOR259C \| YPL096W \| YGL048C \| YOR117W \| YIL007C \| YKL145W \| YOR027W \| YPR178W |
| complex_1440 | YAL024C \| YBR160W \| YBL045C \| YNL068C \| YBL105C \| YNL102W \| YCR065W \| YER165W \| YGR092W \| YMR001C \| YPR119W \| YJL187C \| YML064C \| YMR036C \| YOL100W |
| complex_568 | YAL005C \| YDL229W \| YER081W \| YER165W \| YLL024C \| YLR310C \| YNL007C \| YOR027W \| YPL106C \| YPR128C \| YDR146C \| YNL209W \| YKL101W \| YNL307C \| YNL041C |
| complex_205 | YBR160W \| YGL048C \| YKL145W \| YDL097C \| YDL007W \| YDR394W \| YEL037C \| YHR027C \| YOR117W \| YOR259C \| YOR261C \| YDL147W \| YGR092W \| YPL096W \| YER165W |
| complex_566 | YAL005C \| YNL308C \| YBR155W \| YDL229W \| YLR310C \| YNL007C \| YNL175C \| YDR477W \| YLL024C \| YNL209W \| YEL030W \| YGL070C \| YER165W \| YGR159C \| YPL106C |
| complex_203 | YDL007W \| YFR004W \| YDL097C \| YDL025C \| YDR394W \| YGL048C \| YGR262C \| YHR027C \| YKL145W \| YOR117W \| YOR259C \| YOR261C \| YDL147W \| YDL029W \| YPL026C |
| complex_564 | YAL005C \| YBL075C \| YDL229W \| YER165W \| YLR310C \| YNL064C \| YOR027W \| YPR128C \| YDR356W \| YOR257W \| YDR477W \| YER028C \| YLL024C \| YHR102W \| YIL149C |
| complex_2529 | YBL016W \| YCR057C \| YDL159W \| YLR129W \| YLR222C \| YLR362W \| YLR409C \| YBR247C \| YCL059C \| YNL178W \| YPR144C \| YGR040W \| YGR135W \| YMR128W \| YMR290C |
| complex_2528 | YBL105C \| YBR160W \| YJL095W \| YPR054W \| YPR119W \| YCR065W \| YKL129C \| YDL029W \| YHR030C \| YDR356W \| YHR086W \| YNL189W \| YPL140C \| YPR191W \| YLR096W |
| complex_2527 | YAL024C \| YMR001C \| YBR160W \| YBL105C \| YCR065W \| YGR092W \| YGR108W \| YJL187C \| YKL129C \| YNL298W \| YOL100W \| YOR231W \| YPR119W \| YER165W \| YJL095W |
| complex_2526 | YBR109C \| YGL019W \| YOR039W \| YOR061W \| YOR326W \| YDL195W \| YIL131C \| YER148W \| YFL039C \| YLR399C \| YIL118W \| YML010W \| YOL004W \| YIL035C \| YLR114C |
| complex_2525 | YBR160W \| YLR131C \| YDL155W \| YMR109W \| YNL161W \| YDR216W \| YDL029W \| YFR027W \| YNL007C \| YFL034C-B \| YPR119W \| YHL007C \| YIL106W \| YJL095W \| YKL129C |
| complex_2524 | YDR432W \| YHR086W \| YIL061C \| YIR001C \| YMR290C \| YNL016W \| YNL189W \| YER133W \| YJL033W \| YJR093C \| YKL059C \| YKR002W \| YNL317W \| YER165W \| YGL049C |
| complex_2885 | YBR160W \| YER008C \| YGL003C \| YJL187C \| YMR001C \| YNL298W \| YPR120C \| YDR054C \| YDR328C \| YFL009W \| YIL046W \| YMR304W \| YJR090C \| YGR092W \| YHL035C |
| complex_2521 | YDL029W \| YLL034C \| YPR178W \| YDL208W \| YKL173W \| YDR283C \| YDR473C \| YLR424W \| YFL017W-A \| YLR117C \| YLR147C \| YPL151C \| YGL120C \| YLR309C \| YNL147W |

| | |
|---|---|
| complex_2883 | YBL045C \| YBR160W \| YPR054W \| YPR191W \| YCL011C \| YDL084W \| YDR432W \| YKL139W \| YNL112W \| YDL108W \| YGL173C \| YOL123W \| YFL029C \| YGL137W \| YHR030C |
| complex_2520 | YBL045C \| YJL164C \| YBR028C \| YHR135C \| YDR490C \| YER165W \| YFL033C \| YGL137W \| YLR216C \| YPL031C \| YIL033C \| YMR022W \| YKL166C \| YPL203W \| YLL039C |
| complex_2880 | YAL005C \| YDR477W \| YLR310C \| YBL016W \| YGL158W \| YGR040W \| YBL075C \| YLR362W \| YDL159W \| YGR159C \| YJL128C \| YLR113W \| YMR139W \| YJR032W \| YLL024C |
| complex_1438 | YBR160W \| YDL155W \| YER008C \| YGL003C \| YGR092W \| YIL106W \| YJL187C \| YLR131C \| YMR001C \| YNL068C \| YPL209C \| YPR119W \| YPR120C \| YMR304W \| YNL298W |
| complex_1436 | YBR160W \| YDL155W \| YER008C \| YGL003C \| YGR092W \| YJL187C \| YKL129C \| YMR001C \| YNL068C \| YNL102W \| YPR111W \| YPR119W \| YPR120C \| YIL106W \| YKL101W |
| complex_1797 | YBL045C \| YCR065W \| YGR092W \| YBR160W \| YBL105C \| YGL003C \| YNL298W \| YDL155W \| YER165W \| YPR119W \| YIL106W \| YJL060W \| YJL187C \| YMR001C \| YPL151C |
| complex_1434 | YBL105C \| YBR160W \| YER165W \| YGL003C \| YJL095W \| YOL100W \| YOR231W \| YPR119W \| YER008C \| YJL187C \| YMR001C \| YMR304W \| YJL060W \| YKR048C \| YPR165W |
| complex_1433 | YAL005C \| YLR310C \| YBL016W \| YBL075C \| YLR362W \| YOR027W \| YPL141C \| YDL159W \| YGR040W \| YDL229W \| YDR477W \| YLL024C \| YPL106C \| YPL240C \| YJL138C |
| complex_1794 | YAL005C \| YBL075C \| YLL024C \| YLR310C \| YPR119W \| YGR040W \| YLR362W \| YBR200W \| YBL016W \| YDL159W \| YGL158W \| YHR018C \| YLR113W \| YJL128C \| YNL209W |
| complex_1793 | YBL045C \| YCR065W \| YMR001C \| YBL105C \| YOL100W \| YBR160W \| YGL003C \| YJL187C \| YNL102W \| YER165W \| YGR159C \| YPR119W \| YGR092W \| YIR005W \| YKL129C |
| complex_1430 | YBR109C \| YGL106W \| YBR160W \| YNL298W \| YCL024W \| YJR076C \| YCR002C \| YDR507C \| YLR229C \| YDL225W \| YHR107C \| YHL007C \| YLR362W \| YKL129C \| YLR314C |
| complex_1791 | YBR160W \| YER008C \| YGL003C \| YJL187C \| YMR001C \| YNL068C \| YNL298W \| YPL209C \| YPR119W \| YPR120C \| YCL024W \| YIL046W \| YKR048C \| YDR507C \| YMR304W |
| complex_2519 | YAL005C \| YBL075C \| YJL128C \| YLL024C \| YLR310C \| YNL064C \| YOR027W \| YBL016W \| YDL159W \| YGR040W \| YLR362W \| YNR031C \| YCR073C \| YFL039C \| YMR022W |
| complex_2516 | YCL011C \| YDL084W \| YDR432W \| YKL139W \| YNL004W \| YNL112W \| YPR191W \| YGL173C \| YHR086W \| YDR477W \| YER129W \| YPR054W \| YFL029C \| YFL039C \| YHR030C |
| complex_2515 | YDL195W \| YGL019W \| YIL131C \| YNL112W \| YOR061W \| YER148W \| YFL039C \| YLR399C \| YOR039W \| YIL118W \| YOL004W \| YIL035C \| YOR326W \| YLR429W \| YPL204W |
| complex_2514 | YBR066C \| YDL155W \| YDR146C \| YDR477W \| YMR001C \| YPR119W \| YBR160W \| YGL003C \| YLR131C \| YDR216W \| YIL106W \| YER028C \| YER129W \| YGL035C \| YGR092W |
| complex_2512 | YBL105C \| YOL100W \| YDR432W \| YGR159C \| YIR001C \| YNL016W \| YOL123W \| YOR204W \| YPR119W \| YER165W \| YJL033W \| YNL317W \| YGL049C \| YGR162W \| YDR390C |
| complex_2873 | YDL195W \| YGL019W \| YIL035C \| YIL131C \| YNL112W \| YER006W \| YDL031W \| YPL204W \| YIL118W \| YLR399C \| YOL004W \| YOR039W \| YOR326W \| YOL145C \| YOR061W |
| complex_2510 | YBR160W \| YDL155W \| YDR212W \| YER129W \| YGL003C \| YGR092W \| YIL106W \| YMR001C \| YPR119W \| YPR120C \| YDR477W \| YGL137W \| YIL142W \| YKR036C \| YPL042C |

| | |
|---|---|
| complex_2870 | YAR019C \| YBR160W \| YNL102W \| YPL209C \| YCR065W \| YDL155W \| YKL129C \| YDR146C \| YPR119W \| YGR092W \| YHL035C \| YIL106W \| YNL068C \| YPR111W \| YJR059W |
| complex_1789 | YBL075C \| YLR310C \| YNL064C \| YNL298W \| YBR160W \| YBR200W \| YCR065W \| YHL007C \| YHR018C \| YJL187C \| YJR059W \| YKL129C \| YNR047W \| YPR119W \| YLR362W |
| complex_1787 | YAL005C \| YDR172W \| YDR356W \| YER165W \| YNL209W \| YOL123W \| YBL045C \| YGR159C \| YBL105C \| YDL229W \| YPR119W \| YDR432W \| YOR204W \| YGL044C \| YPL106C |
| complex_1786 | YBL105C \| YOR231W \| YPL140C \| YCR065W \| YHR030C \| YDL029W \| YHR102W \| YPR054W \| YHR086W \| YPR119W \| YPR191W \| YJL095W \| YKL129C \| YMR109W \| YNL161W |
| complex_1423 | YBL045C \| YKL139W \| YOL123W \| YBL105C \| YOL100W \| YBR034C \| YGR159C \| YCL011C \| YDL084W \| YDR432W \| YPR119W \| YER165W \| YGL173C \| YOR204W \| YGL044C |
| complex_1420 | YBL045C \| YKL139W \| YOL123W \| YBL105C \| YOL100W \| YPL151C \| YCL011C \| YDL084W \| YDR432W \| YPR119W \| YER165W \| YGL173C \| YGR159C \| YOR204W \| YJL060W |
| complex_548 | YAL032C \| YKL173W \| YLL036C \| YLR117C \| YLR424W \| YMR001C \| YMR213W \| YPL151C \| YDL030W \| YDL208W \| YDR283C \| YPR178W \| YER013W \| YGL120C \| YMR240C |
| complex_543 | YAL005C \| YBR155W \| YDL229W \| YDR477W \| YER081W \| YER165W \| YLR310C \| YNL007C \| YNL209W \| YOR222W \| YPL106C \| YDR356W \| YFL037W \| YEL030W \| YIL095W |
| complex_2509 | YBR160W \| YGL003C \| YJR059W \| YNL068C \| YPR120C \| YDR212W \| YBR198C \| YJL106W \| YNL317W \| YPL115C \| YIL142W \| YJL187C \| YMR001C \| YPR119W \| YNL298W |
| complex_2508 | YDR188W \| YDL047W \| YDR142C \| YGL137W \| YJL164C \| YNL317W \| YOR212W \| YPL203W \| YDR212W \| YDR075W \| YJL106W \| YKL166C \| YIL142W \| YJL014W \| YJR064W |
| complex_2507 | YCR002C \| YDR218C \| YDR507C \| YHR107C \| YJR076C \| YDL225W \| YGR162W \| YMR116C \| YPR119W \| YHR013C \| YBR118W \| YHR023W \| YKL101W \| YMR001C \| YLR314C |
| complex_2506 | YAL041W \| YOR212W \| YBR160W \| YBR200W \| YHL007C \| YJL187C \| YKL129C \| YNL298W \| YLR362W \| YDL029W \| YFL034C-B \| YHR030C \| YHR102W \| YNL161W \| YNL007C |
| complex_2505 | YBR109C \| YGL019W \| YGR270W \| YOR039W \| YOR061W \| YBR160W \| YDR507C \| YER165W \| YIL035C \| YML010W \| YMR139W \| YGL254W \| YIL131C \| YML074C \| YOL145C |
| complex_2504 | YAR019C \| YBR160W \| YIL112W \| YJL187C \| YNL102W \| YCR065W \| YDL155W \| YGR092W \| YKL129C \| YML064C \| YMR001C \| YPR119W \| YGR270W \| YIL106W \| YPR111W |
| complex_2865 | YBL045C \| YKL095W \| YPL203W \| YDL101C \| YGL137W \| YKL166C \| YLR216C \| YHR086W \| YJL164C \| YHR135C \| YIL033C \| YMR226C \| YMR022W \| YLR347C \| YPL153C |
| complex_2864 | YBR109C \| YGL019W \| YGR270W \| YOR039W \| YOR061W \| YGL254W \| YIL035C \| YML010W \| YML074C \| YHL027W \| YIL131C \| YJL056C \| YOL145C |
| complex_2501 | YBL045C \| YAR019C \| YGR159C \| YDR432W \| YER165W \| YMR216C \| YOR204W \| YPR119W \| YDR477W \| YER129W \| YPL042C \| YGR092W \| YKR036C \| YGL137W \| YJL060W |
| complex_2863 | YBL016W \| YIL169C \| YBL075C \| YLR362W \| YNL298W \| YPR111W \| YBR160W \| YKL129C \| YBR200W \| YDL159W \| YPR119W \| YGR040W \| YHL007C \| YHR018C \| YIL106W |
| complex_2861 | YBR135W \| YBR160W \| YCR065W \| YNL161W \| YDL029W \| YKL129C \| YKL145W \| YDL155W \| YGR092W \| YDR146C \| YLR131C \| YPR119W \| YGR108W \| YHL007C \| YIL106W |

| | |
|---|---|
| complex_1417 | YDR212W \| YBR160W \| YFL033C \| YGL116W \| YGL137W \| YHR135C \| YJL106W \| YJL164C \| YKL166C \| YNL317W \| YNL027W \| YIL033C \| YIL142W \| YNL093W \| YPL203W |
| complex_1779 | YAR002C-A \| YAL007C \| YGL200C \| YLR026C \| YML012W \| YDL159W \| YER151C \| YLR208W \| YHR098C \| YJR048W \| YPR181C \| YIL109C \| YPL218W \| YNL049C \| YPR105C |
| complex_1776 | YAL024C \| YBR160W \| YJL095W \| YBL105C \| YER165W \| YOL100W \| YOR231W \| YPR119W \| YCR065W \| YNR047W \| YDR432W \| YGR159C \| YPR191W \| YJR059W \| YMR036C |
| complex_1413 | YBL045C \| YCR065W \| YOL100W \| YBL105C \| YBR160W \| YER165W \| YJL095W \| YMR001C \| YPL151C \| YPR119W \| YGL003C \| YJL187C \| YMR304W \| YHR030C \| YJL060W |
| complex_1775 | YBR160W \| YCR065W \| YDL155W \| YGL003C \| YGR092W \| YKL129C \| YMR001C \| YNL102W \| YMR304W \| YIL106W \| YDL028C \| YPR111W \| YPR119W \| YJL187C \| YNL298W |
| complex_1412 | YBR160W \| YLR210W \| YNL317W \| YDR188W \| YDR142C \| YGL137W \| YJL164C \| YKR036C \| YLL011W \| YDR212W \| YJL106W \| YGR092W \| YIL142W \| YJR064W \| YPL042C |
| complex_1774 | YBR160W \| YDL029W \| YDR146C \| YCR065W \| YDL155W \| YDR216W \| YGL216W \| YPR119W \| YGR092W \| YHL007C \| YKL129C \| YIL106W \| YNL161W \| YKR095W \| YLR131C |
| complex_1411 | YAL005C \| YLR310C \| YNL064C \| YBL016W \| YBL075C \| YLL024C \| YNL209W \| YPR119W \| YDL229W \| YDR356W \| YDR477W \| YPL106C \| YHR018C \| YLR362W \| YPL141C |
| complex_1773 | YAR019C \| YGR092W \| YNL161W \| YBR160W \| YCR065W \| YKL129C \| YPR120C \| YDL155W \| YDR146C \| YLR131C \| YPR119W \| YHL007C \| YIL106W \| YNL068C \| YDL029W |
| complex_1410 | YAR019C \| YGR092W \| YBL045C \| YCR065W \| YBR160W \| YGL003C \| YPR111W \| YDL155W \| YER165W \| YPR119W \| YMR304W \| YIL106W \| YJL060W \| YJL187C \| YMR001C |
| complex_1772 | YBR160W \| YBR200W \| YCR065W \| YHL007C \| YJL187C \| YKL129C \| YMR001C \| YNL298W \| YPR119W \| YAL041W \| YGR152C \| YDR379W \| YLR229C \| YLR362W \| YOR127W |
| complex_539 | YAL005C \| YDL229W \| YER081W \| YER165W \| YJR059W \| YLL024C \| YLR310C \| YNL007C \| YNL041C \| YNL209W \| YPL106C \| YPR119W \| YPR128C \| YBR160W \| YDR146C |
| complex_538 | YAL032C \| YKL173W \| YLL036C \| YLR117C \| YLR424W \| YMR001C \| YMR213W \| YBL105C \| YDL030W \| YDR364C \| YLR441C \| YER013W \| YGL120C \| YMR240C \| YPL151C |
| complex_899 | YCL024W \| YJL187C \| YDL087C \| YKL173W \| YDL208W \| YPR178W \| YDR235W \| YPR182W \| YER029C \| YMR001C \| YGL120C \| YHR086W \| YLR147C \| YNL147W \| YPL151C |
| complex_898 | YAL032C \| YKL173W \| YLL036C \| YLR117C \| YLR424W \| YMR213W \| YDL101C \| YER013W \| YMR001C \| YGL120C \| YHL002W \| YJL074C \| YFL008W \| YNL250W \| YPL151C |
| complex_897 | YAL005C \| YDL229W \| YER165W \| YLR310C \| YNL007C \| YNL209W \| YPR128C \| YBL105C \| YDR477W \| YOR244W \| YPR154W \| YER028C \| YPL106C \| YOL100W \| YPR112C |
| complex_893 | YBL016W \| YLR362W \| YDL097C \| YDL007W \| YDR394W \| YGL048C \| YGR040W \| YHR027C \| YHR030C \| YKL145W \| YOR117W \| YOR259C \| YOR261C \| YDL147W \| YDL159W |
| complex_890 | YAL005C \| YLR310C \| YNL064C \| YOR027W \| YPL106C \| YBL075C \| YPL240C \| YDR477W \| YER028C \| YJR002W \| YLL024C \| YNL096C \| YPL081W \| YHR148W \| YNL075W |
| complex_2857 | YBR160W \| YMR001C \| YCR065W \| YDL074C \| YFR031C \| YJL074C \| YOL069W \| YOR195W \| YDR356W \| YPR119W \| YFL008W \| YPR141C \| YIL144W \| YKL129C \| YLR086W |

| | |
|---|---|
| complex_2850 | YER081W \| YBR155W \| YFL037W \| YOR061W \| YHR030C \| YJL034W \| YKL073W \| YHR079C \| YIL061C \| YOR254C \| YPL204W \| YNL027W |
| complex_1408 | YEL030W \| YER081W \| YFL016C \| YIL061C \| YLR310C \| YNL161W \| YOR222W \| YHL007C \| YHR102W \| YHR030C \| YKL073W \| YJR045C \| YJL034W \| YNL007C \| YER036C |
| complex_1769 | YAL005C \| YBR155W \| YCR091W \| YDL229W \| YDR477W \| YER081W \| YNR047W \| YBL075C \| YPR119W \| YBR160W \| YDR356W \| YER129W \| YLL024C \| YNL209W \| YPL106C |
| complex_1406 | YAL041W \| YOR212W \| YBR160W \| YER008C \| YHL007C \| YJL187C \| YKL129C \| YNL298W \| YDL029W \| YMR304W \| YNL161W \| YJL095W \| YMR109W \| YLR229C \| YLR362W |
| complex_1768 | YAL005C \| YLL024C \| YLR310C \| YLR362W \| YNL064C \| YOR027W \| YPL042C \| YBL075C \| YDL229W \| YPL240C \| YDR216W \| YNL298W \| YDR477W \| YHR018C \| YPL106C |
| complex_1405 | YBR160W \| YGL003C \| YJL187C \| YNL102W \| YDL155W \| YKL129C \| YNL068C \| YGR092W \| YMR001C \| YPR119W \| YHL035C \| YIL106W \| YPR111W \| YNL298W \| YPR120C |
| complex_1403 | YBR135W \| YPR120C \| YBR160W \| YDL155W \| YGL003C \| YGR108W \| YHR158C \| YJL187C \| YLR131C \| YMR001C \| YNL068C \| YNL102W \| YPR119W \| YDR146C \| YGR092W |
| complex_1764 | YBL105C \| YOL100W \| YOR231W \| YPL151C \| YBR160W \| YCR065W \| YER165W \| YGR159C \| YKL025C \| YPR119W \| YJL060W \| YJL095W \| YMR109W \| YKL129C \| YDL029W |
| complex_1401 | YBR160W \| YLR210W \| YDR188W \| YBR198C \| YDR142C \| YGR040W \| YLL011W \| YNL317W \| YOR212W \| YDR212W \| YDR075W \| YIL142W \| YJL106W \| YJR064W \| YJL014W |
| complex_1763 | YAL005C \| YHR086W \| YLR310C \| YNL064C \| YPL106C \| YBL016W \| YHR018C \| YBL075C \| YPR119W \| YER029C \| YDR188W \| YGL254W \| YLL024C \| YNL209W \| YMR139W |
| complex_1761 | YAL005C \| YLR310C \| YNL064C \| YPL106C \| YBL016W \| YBL075C \| YDL159W \| YGR040W \| YDL229W \| YDR356W \| YDR477W \| YLL024C \| YNL209W \| YLR362W \| YNL127W |
| complex_889 | YAL041W \| YLR229C \| YNL298W \| YBR160W \| YDR507C \| YLR314C \| YMR001C \| YCL024W \| YCR002C \| YDL225W \| YHR107C \| YKL101W \| YJL187C \| YDL145C \| YJR076C |
| complex_888 | YDL097C \| YDL007W \| YDL025C \| YDR394W \| YGL048C \| YHR027C \| YHR030C \| YKL145W \| YOR117W \| YOR259C \| YOR261C \| YDL147W \| YPL140C \| YJL095W \| YOR231W |
| complex_525 | YAL005C \| YLR310C \| YNL308C \| YBL075C \| YGL070C \| YNL064C \| YOR027W \| YPL042C \| YPL240C \| YDL229W \| YDR477W \| YNL209W \| YPL106C \| YLL024C \| YPR054W |
| complex_886 | YBR160W \| YCL024W \| YMR001C \| YNL298W \| YCR002C \| YDR507C \| YHR107C \| YJR076C \| YLR314C \| YDL225W \| YDR054C \| YKL101W \| YJL187C \| YKL129C \| YOL133W |
| complex_524 | YDL007W \| YFR004W \| YDL097C \| YDR394W \| YGL048C \| YGR262C \| YKL145W \| YOR117W \| YOR259C \| YOR261C \| YDL147W \| YDL029W \| YPL026C \| YHR030C \| YLR309C |
| complex_885 | YAL032C \| YKL095W \| YKL173W \| YLR117C \| YMR213W \| YBR065C \| YDR364C \| YER013W \| YMR001C \| YFL017W-A \| YLR347C \| YPL153C \| YMR240C \| YPL151C \| YNL189W |
| complex_522 | YAL005C \| YBL075C \| YBR155W \| YDL229W \| YGL070C \| YLL024C \| YLR310C \| YNL007C \| YNL209W \| YNL308C \| YPL106C \| YER107C \| YNL175C \| YDR490C \| YHR205W |
| complex_521 | YAL005C \| YBL075C \| YBR155W \| YDR477W \| YER081W \| YFL016C \| YLL024C \| YLR310C \| YNL209W \| YOR027W \| YOR222W \| YPL106C \| YDL229W \| YEL030W \| YDL017W |

| | |
|---|---|
| complex_881 | YER012W \| YER049W \| YGL011C \| YGR135W \| YJL001W \| YLL024C \| YML092C \| YMR314W \| YOL038W \| YOR362C \| YPR103W \| YER129W \| YDR477W \| YOR157C \| YMR308C |
| complex_880 | YAL005C \| YER165W \| YNL308C \| YBR155W \| YDL229W \| YDR477W \| YLR310C \| YNL007C \| YPL106C \| YLL024C \| YFR010W \| YDL007W \| YMR116C \| YNL209W |
| complex_2843 | YER165W \| YIL035C \| YJL164C \| YLR212C \| YPL031C \| YGL019W \| YIL131C \| YOR039W \| YOR061W \| YIL033C \| YFL033C \| YMR022W \| YKL166C \| YNL027W \| YPL203W |
| complex_2842 | YCL011C \| YFL039C \| YNL004W \| YNL112W \| YPR119W \| YDL195W \| YGL019W \| YIL035C \| YIL131C \| YOR061W \| YIL118W \| YLR399C \| YOL004W \| YOR039W \| YOR326W |
| complex_2840 | YCL011C \| YDL084W \| YDR432W \| YDR477W \| YER165W \| YKL139W \| YNL112W \| YDL192W \| YDL229W \| YAL005C \| YDR356W \| YFR002W \| YIL149C \| YKR095W \| YNL004W |
| complex_1755 | YDL208W \| YKL173W \| YDR394W \| YDL029W \| YFL029C \| YDR283C \| YPR054W \| YHR030C \| YLR216C \| YPR191W \| YHR086W \| YLR147C \| YMR001C \| YNL147W \| YPR178W |
| complex_1753 | YBL105C \| YPL151C \| YBR065C \| YMR001C \| YPR119W \| YBR160W \| YCR065W \| YNL102W \| YOL100W \| YOL123W \| YDR432W \| YGR159C \| YOR204W \| YER165W \| YKL025C |
| complex_1752 | YBL105C \| YBR160W \| YER165W \| YJL095W \| YOL100W \| YOR231W \| YPL140C \| YPR119W \| YIL131C \| YKL129C \| YPR054W \| YDL029W \| YHR030C \| YPR191W \| YGL019W |
| complex_1751 | YBL105C \| YBR160W \| YER165W \| YOL100W \| YPL151C \| YPR119W \| YBR233W \| YDR432W \| YGR159C \| YHR086W \| YIR001C \| YOR204W \| YGL049C \| YNL016W \| YGR162W |
| complex_519 | YAL005C \| YBL075C \| YDL229W \| YER081W \| YER165W \| YLL024C \| YLR310C \| YNL064C \| YNL209W \| YOR027W \| YPR128C \| YBL016W \| YLR362W \| YNL041C \| YPL106C |
| complex_1750 | YBL016W \| YBR200W \| YDL159W \| YLR362W \| YOR362C \| YBL075C \| YBR160W \| YHL007C \| YKL129C \| YJL187C \| YPR119W \| YGR040W \| YHR018C \| YGL137W \| YNL298W |
| complex_879 | YBR160W \| YCL024W \| YGL003C \| YKL129C \| YMR001C \| YCR002C \| YDR507C \| YHR107C \| YNL298W \| YPR119W \| YGR092W \| YKL101W \| YJL187C \| YJR076C \| YLR314C |
| complex_878 | YAL005C \| YBR155W \| YDL229W \| YDR356W \| YER081W \| YLL024C \| YLR310C \| YNL209W \| YDR146C \| YPL106C \| YDR477W \| YDL192W \| YPR161C \| YER165W \| YFL037W |
| complex_877 | YAL005C \| YBR155W \| YER081W \| YLR310C \| YCL011C \| YDR432W \| YMR216C \| YDL229W \| YIL061C \| YOR254C \| YPL106C \| YPR128C \| YDR146C \| YJL034W \| YKL073W |
| complex_514 | YAL005C \| YDL229W \| YER081W \| YER165W \| YLL024C \| YLR310C \| YNL007C \| YNL209W \| YOR185C \| YPR128C \| YDR477W \| YOL128C \| YER028C \| YPL106C \| YNL041C |
| complex_512 | YAL005C \| YDL229W \| YER081W \| YER165W \| YLL024C \| YLR310C \| YNL007C \| YOR027W \| YPL106C \| YPR128C \| YBR160W \| YPR054W \| YDR146C \| YNL209W \| YNL041C |
| complex_510 | YBR160W \| YGL048C \| YKL145W \| YDL025C \| YDL097C \| YDL007W \| YDR394W \| YEL037C \| YER012W \| YHR027C \| YOR117W \| YOR259C \| YPL096W \| YGR092W \| YNL016W |
| complex_2839 | YBL045C \| YBR160W \| YCR065W \| YBL105C \| YER165W \| YIL033C \| YOL100W \| YPR119W \| YIL035C \| YJL164C \| YHR135C \| YMR022W \| YKL166C \| YNL027W \| YPL203W |
| complex_2838 | YBR160W \| YBR200W \| YCR065W \| YHL007C \| YHR018C \| YJL187C \| YKL129C \| YMR001C \| YNL102W \| YNL298W \| YPR119W \| YHR064C \| YAR018C \| YIL046W \| YDR054C |

| | |
|---|---|
| complex_2835 | YAR019C \| YGR092W \| YHR169W \| YPL042C \| YBR160W \| YDL155W \| YKL129C \| YNL068C \| YPR111W \| YCR065W \| YPR119W \| YDR146C \| YIL106W \| YKR036C \| YMR001C |
| complex_2832 | YBL105C \| YBR160W \| YHR018C \| YJL095W \| YOL100W \| YOR231W \| YPR119W \| YBR109C \| YKL129C \| YCR065W \| YGR108W \| YER103W \| YDL029W \| YMR109W \| YNL298W |
| complex_2831 | YBL105C \| YBR160W \| YJL095W \| YJL138C \| YOL100W \| YPR119W \| YCR065W \| YGL003C \| YER165W \| YGR159C \| YKL025C \| YJL060W \| YKL129C \| YMR109W \| YOR231W |
| complex_1745 | YAL005C \| YBR085W \| YBR155W \| YER081W \| YFL016C \| YLR310C \| YNL007C \| YCR084C \| YDL101C \| YJL034W \| YKL073W \| YEL030W \| YNL161W \| YOR232W \| YPL153C |
| complex_1744 | YBL105C \| YBR160W \| YER008C \| YGL003C \| YMR001C \| YJL187C \| YPL209C \| YDR432W \| YGR159C \| YOL123W \| YOR204W \| YPR119W \| YMR304W \| YER165W \| YOL100W |
| complex_1741 | YAL005C \| YBL075C \| YJR032W \| YLL024C \| YLR310C \| YMR139W \| YNL007C \| YBL016W \| YDL159W \| YJL128C \| YLR362W \| YEL030W \| YGR040W \| YNR031C |
| complex_1740 | YAL005C \| YDR172W \| YDL229W \| YDR432W \| YER165W \| YGR159C \| YOL123W \| YOR204W \| YPR119W \| YDR477W \| YNL209W \| YPL106C \| YER028C \| YLL024C \| YGL044C |
| complex_867 | YAL005C \| YBL075C \| YBR155W \| YER081W \| YLR310C \| YOR027W \| YDL229W \| YDR477W \| YNL209W \| YGL158W \| YJR032W \| YPL240C \| YLL024C \| YPL106C \| YLR347C |
| complex_866 | YAL032C \| YKL173W \| YLL036C \| YLR117C \| YLR424W \| YMR213W \| YPL151C \| YBL047C \| YPL153C \| YER013W \| YMR001C \| YGL120C \| YGR136W \| YJL074C \| YNL189W |
| complex_504 | YDL007W \| YFR004W \| YDL097C \| YDR394W \| YGL048C \| YGR262C \| YKL145W \| YOR117W \| YOR259C \| YOR261C \| YDL147W \| YDL029W \| YJL008C \| YPL026C \| YLR180W |
| complex_503 | YAL005C \| YBR085W \| YER107C \| YER165W \| YLL016W \| YLR310C \| YNL064C \| YOR027W \| YPL106C \| YBL075C \| YDL108W \| YDL155W \| YDR146C \| YLL024C \| YLR113W |
| complex_864 | YAL005C \| YER081W \| YER165W \| YHR030C \| YLL024C \| YLR310C \| YOR027W \| YOR254C \| YPL106C \| YPL140C \| YBL105C \| YJL034W \| YKL073W \| YJL095W \| YNL027W |
| complex_502 | YAL005C \| YBR155W \| YDL229W \| YDR356W \| YDR477W \| YGL070C \| YLL024C \| YLR310C \| YNL007C \| YNL209W \| YNL308C \| YPL106C \| YEL030W \| YNL175C \| YML085C |
| complex_863 | YAL005C \| YBR085W \| YBR155W \| YER081W \| YIR005W \| YJL141C \| YLR310C \| YNL007C \| YNL209W \| YDR146C \| YEL030W \| YHR064C \| YLL024C \| YDR477W \| YNL161W |
| complex_501 | YAL005C \| YCL011C \| YDL229W \| YLR310C \| YNL007C \| YNL209W \| YPR128C \| YDR432W \| YER165W \| YKL139W \| YDR477W \| YER028C \| YPL106C \| YNL004W \| YNL041C |
| complex_2828 | YBR109C \| YGL019W \| YGR270W \| YOR039W \| YOR061W \| YOR326W \| YGL254W \| YIL118W \| YIL131C \| YML010W \| YML074C \| YIL035C \| YNL189W \| YOL145C |
| complex_2827 | YBL045C \| YPR054W \| YPR191W \| YDL017W \| YDL029W \| YHR030C \| YPL140C \| YDL160C \| YDR394W \| YKL073W \| YOR027W \| YGL137W \| YMR216C \| YJL095W \| YPR178W |
| complex_2825 | YAL005C \| YBL105C \| YBL045C \| YBR160W \| YCR065W \| YDL108W \| YLR113W \| YDR477W \| YER129W \| YGL158W \| YER165W \| YPR119W \| YHR018C \| YKL129C \| YOL100W |
| complex_2821 | YBL045C \| YCR065W \| YNL298W \| YBR133C \| YER165W \| YHL007C \| YJL187C \| YBR160W \| YBL105C \| YDR054C \| YPR119W \| YGR040W \| YIL046W \| YLR362W \| YOL100W |

| | |
|---|---|
| complex_1739 | YBL030C \| YGL137W \| YDR188W \| YHR135C \| YJL106W \| YNL317W \| YOR212W \| YPL203W \| YDR212W \| YIL142W \| YJL164C \| YIL033C \| YNL027W \| YKL166C \| YNL093W |
| complex_1738 | YBR160W \| YDL029W \| YCR065W \| YDL155W \| YGR092W \| YIL106W \| YNL161W \| YNL298W \| YPR119W \| YJL187C \| YMR001C \| YKL129C \| YLR131C \| YOR127W \| YPR111W |
| complex_1737 | YBL045C \| YGR159C \| YDR432W \| YER165W \| YHR086W \| YIL061C \| YIR001C \| YJL033W \| YKR095W \| YNL016W \| YOR204W \| YPR119W \| YGL049C \| YGR162W \| YDR235W |
| complex_1736 | YBL075C \| YNL298W \| YBR160W \| YCR065W \| YHR018C \| YJL187C \| YKL129C \| YNL102W \| YPR119W \| YBR200W \| YDR054C \| YHL007C \| YIL046W \| YLR362W \| YMR001C |
| complex_1733 | YBL105C \| YER008C \| YOL100W \| YPL151C \| YBR065C \| YMR001C \| YPR119W \| YBR160W \| YCR065W \| YGL003C \| YJL187C \| YNL102W \| YER165W \| YKL025C \| YGL094C |
| complex_1730 | YBL045C \| YCR065W \| YPR054W \| YPR191W \| YBR160W \| YDL108W \| YLR096W \| YNL102W \| YDR432W \| YER165W \| YKL139W \| YPR119W \| YFL029C \| YGL137W \| YHR030C |
| complex_857 | YAL005C \| YDR477W \| YER081W \| YIL061C \| YLL024C \| YNL209W \| YOR027W \| YOR061W \| YOR254C \| YPL106C \| YDL229W \| YDR146C \| YJL034W \| YKL073W \| YHR079C |
| complex_856 | YAL005C \| YBL075C \| YIR005W \| YLR310C \| YNL064C \| YOR027W \| YPR119W \| YPR128C \| YAR018C \| YHR064C \| YDR146C \| YLL024C \| YER103W \| YNL135C \| YOR319W |
| complex_854 | YBR160W \| YGL003C \| YJL187C \| YMR001C \| YNL298W \| YCL024W \| YCR002C \| YDL225W \| YDR507C \| YHR107C \| YKL101W \| YNL064C \| YJR076C \| YLR314C \| YBL075C |
| complex_853 | YAL005C \| YER165W \| YLL016W \| YLR310C \| YNL064C \| YOR027W \| YPL106C \| YBL016W \| YLR362W \| YBL075C \| YBR160W \| YDL159W \| YGR040W \| YDR146C \| YLL024C |
| complex_850 | YAL005C \| YER081W \| YER165W \| YLR310C \| YNL007C \| YOR185C \| YPR128C \| YDL229W \| YDR477W \| YNL041C \| YPL106C \| YNL209W \| YER028C \| YLR131C \| YOL128C |
| complex_2817 | YCL011C \| YDL084W \| YDR432W \| YFL039C \| YGL008C \| YKL139W \| YNL004W \| YNL112W \| YPR191W \| YOL123W \| YFL029C \| YNL138W \| YHR030C \| YPR054W \| YJL081C |
| complex_2815 | YBL105C \| YBR160W \| YER165W \| YJL095W \| YOL100W \| YPR119W \| YCR065W \| YDR146C \| YDR432W \| YPL263C \| YGR159C \| YOR204W \| YKL129C \| YKR095W \| YOR231W |
| complex_2814 | YAL021C \| YCR093W \| YGR092W \| YOR027W \| YPL042C \| YBR160W \| YDL145C \| YDR394W \| YDL007W \| YGL048C \| YKL145W \| YOR117W \| YOR259C \| YEL037C \| YPL096W |
| complex_2810 | YAL024C \| YNR047W \| YBL045C \| YCR065W \| YBR160W \| YBL105C \| YCR091W \| YGL003C \| YJL060W \| YJR059W \| YKL116C \| YMR036C \| YPR119W \| YER165W \| YOL100W |
| complex_1726 | YBR109C \| YGL019W \| YGR270W \| YOR039W \| YOR061W \| YGL254W \| YIL035C \| YIL118W \| YIL131C \| YML010W \| YML074C \| YPL153C \| YHR086W \| YOL145C \| YDL101C |
| complex_1721 | YBL075C \| YLR362W \| YNL064C \| YNL298W \| YBR160W \| YJL187C \| YBR200W \| YPR119W \| YHL007C \| YKL129C \| YHL035C \| YPR120C \| YHR018C \| YMR001C \| YNL068C |
| complex_849 | YBR094W \| YPL240C \| YBR155W \| YEL037C \| YDL097C \| YDL007W \| YDR394W \| YGL048C \| YHR027C \| YKL145W \| YOR259C \| YOR261C \| YDL147W \| YGR123C \| YHR037W |
| complex_846 | YAL005C \| YLR310C \| YNL308C \| YBL075C \| YGL070C \| YNL064C \| YOR027W \| YPL042C \| YDL229W \| YDR477W \| YER165W \| YGR159C \| YNL209W \| YPL106C \| YLL024C |

| | |
|---|---|
| complex_842 | YBR160W \| YDR507C \| YGR092W \| YMR001C \| YCL024W \| YCR002C \| YDL225W \| YGL116W \| YIL112W \| YKL101W \| YHR107C \| YNL298W \| YJL187C \| YJR076C \| YLR314C |
| complex_841 | YAL005C \| YBL075C \| YLR310C \| YNL064C \| YOR027W \| YPL106C \| YPL240C \| YBL016W \| YNL298W \| YGR040W \| YDL159W \| YHR018C \| YLR362W \| YDR477W \| YLL024C |
| complex_2805 | YAL005C \| YBL075C \| YLR310C \| YNL064C \| YPL106C \| YPR119W \| YAL029C \| YBL016W \| YDR477W \| YDR507C \| YMR139W \| YER165W \| YLL024C \| YNL209W \| YOR027W |
| complex_2804 | YCL011C \| YDL084W \| YDR432W \| YKL139W \| YNL112W \| YPR119W \| YKL210W \| YDL175C \| YIL079C \| YGL173C \| YGR159C \| YOL123W \| YOR204W \| YFL039C \| YNL004W |
| complex_2803 | YBR160W \| YBR200W \| YCR065W \| YER129W \| YGL035C \| YJL187C \| YKL129C \| YNL298W \| YPR119W \| YLR362W \| YDR477W \| YHR018C \| YHL007C \| YNL209W \| YJR059W |
| complex_2802 | YBL016W \| YDL159W \| YBR160W \| YAL041W \| YBR200W \| YHL007C \| YJL187C \| YKL129C \| YNL298W \| YOR127W \| YLR229C \| YCR073C \| YJL128C \| YDR054C \| YLR362W |
| complex_2801 | YBL075C \| YHL007C \| YBR160W \| YCR065W \| YCR088W \| YHR018C \| YJL187C \| YKL129C \| YPR119W \| YBR200W \| YCR091W \| YDL029W \| YLR362W \| YNL298W \| YNR047W |
| complex_1718 | YBR160W \| YGL003C \| YJL187C \| YLR131C \| YMR001C \| YDL155W \| YNL161W \| YDR054C \| YER165W \| YPR119W \| YMR304W \| YGR092W \| YNL298W \| YIL046W \| YJL060W |
| complex_1714 | YBR160W \| YMR001C \| YNL298W \| YOR332W \| YCL024W \| YCR002C \| YDR507C \| YHR107C \| YLR229C \| YDL225W \| YIL112W \| YKL101W \| YLR069C \| YJR076C \| YLR314C |
| complex_1713 | YDL017W \| YKL166C \| YDL137W \| YDL192W \| YDR358W \| YHR108W \| YDR477W \| YIL033C \| YLL039C \| YGL137W \| YLR216C \| YHR135C \| YMR022W \| YJL164C \| YPL203W |
| complex_1711 | YAL041W \| YBR200W \| YHL007C \| YLR229C \| YNL298W \| YBR031W \| YBR160W \| YLR362W \| YOR127W \| YBL016W \| YCR065W \| YPR119W \| YDL159W \| YJL187C \| YKL129C |
| complex_831 | YBR160W \| YDR507C \| YLR314C \| YMR001C \| YCL024W \| YCR002C \| YDL225W \| YHR107C \| YNL298W \| YDL029W \| YFR027W \| YKL101W \| YJL187C \| YJR076C \| YKL129C |
| complex_830 | YAL005C \| YBL016W \| YBL075C \| YBR085W \| YER165W \| YLL024C \| YLR310C \| YNL064C \| YOR027W \| YOR388C \| YEL030W \| YLR362W \| YJL128C \| YFL037W |
| complex_1707 | YAL005C \| YLR310C \| YMR104C \| YNL007C \| YBL016W \| YDL229W \| YLL024C \| YPL106C \| YDR477W \| YKL126W \| YDR490C \| YHR018C \| YHR205W \| YPR119W \| YNL209W |
| complex_826 | YBR160W \| YGL048C \| YKL145W \| YDL097C \| YDL007W \| YDR394W \| YEL037C \| YER012W \| YHR027C \| YHR030C \| YOR117W \| YOR259C \| YKL073W \| YNL016W \| YOR208W |
| complex_824 | YAL005C \| YDL229W \| YER081W \| YER165W \| YLL024C \| YLR310C \| YNL007C \| YOR185C \| YPL106C \| YPR128C \| YDR146C \| YNL209W \| YNL041C \| YOL128C \| YPL153C |
| complex_823 | YAL005C \| YBL075C \| YBR085W \| YER165W \| YLL024C \| YLR310C \| YNL007C \| YOR027W \| YDL159W \| YLR362W \| YER036C \| YLR061W \| YNL041C \| YNL064C \| YPL106C |
| complex_822 | YAL017W \| YLR117C \| YAL032C \| YKL173W \| YLL036C \| YLR424W \| YMR213W \| YDL030W \| YER013W \| YMR001C \| YGL120C \| YGL174W \| YMR240C \| YOL045W \| YPL151C |
| complex_817 | YBL075C \| YAL005C \| YER081W \| YFL016C \| YLR310C \| YBR155W \| YDL229W \| YDR356W \| YDR477W \| YPL106C \| YLL024C \| YNL209W \| YEL030W \| YIL095W \| YOR185C |

| | |
|---|---|
| complex_815 | YEL030W \| YER081W \| YFL016C \| YIL035C \| YIL061C \| YLR310C \| YNL308C \| YOR232W \| YIL033C \| YJR045C \| YLL024C \| YLR369W \| YJL164C \| YPL203W \| YNL027W |
| complex_808 | YAL005C \| YER081W \| YER165W \| YLR310C \| YNL007C \| YNL209W \| YPL106C \| YPR128C \| YDL229W \| YGL158W \| YNL041C \| YDR477W \| YER028C \| YER129W \| YGL035C |
| complex_807 | YDL087C \| YKL173W \| YDL208W \| YPR178W \| YDR235W \| YPR182W \| YER029C \| YMR001C \| YMR213W \| YPL151C \| YHR086W \| YML046W \| YIL061C \| YLR147C \| YNL147W |
| complex_805 | YAL005C \| YDR477W \| YER165W \| YGL254W \| YLL024C \| YNL007C \| YNL209W \| YOR027W \| YPR119W \| YCR091W \| YNR047W \| YDL229W \| YPL106C \| YDR146C \| YER103W |
| complex_801 | YAL005C \| YBR155W \| YDR477W \| YER165W \| YIL074C \| YLL024C \| YLR310C \| YNL007C \| YPL106C \| YPR128C \| YEL030W \| YER129W \| YER028C \| YKL073W \| YFL037W |
| complex_800 | YBR160W \| YDL025C \| YGL048C \| YGR092W \| YHR027C \| YKL145W \| YDL097C \| YDL007W \| YDR394W \| YHR030C \| YOR117W \| YOR259C \| YOR261C \| YDL147W \| YLR096W |
| complex_4 | YDL029W \| YDR394W \| YOR259C \| YDL097C \| YDL007W \| YEL037C \| YFR004W \| YGL048C \| YGR262C \| YHR027C \| YKL145W \| YOR117W \| YOR261C \| YDL147W \| YGR092W |
| complex_0 | YBL026W \| YER112W \| YER146W \| YDL087C \| YKL173W \| YDR378C \| YJL124C \| YJR022W \| YLR275W \| YNL147W \| YPR178W \| YHR086W \| YPR182W \| YLR147C \| YOR159C |

**Supplementary Table 8. Novel human predicted human protein interactions.**

| Human | | Yeast | |
|---|---|---|---|
| NP_004517 | NP_006730 | YBL023C | YLR274W |
| NP_002096 | NP_874369 | YDR225W | YOR244W |
| NP_001782 | NP_056461 | YGR152C | YLR229C |
| NP_060561 | NP_996662 | YBL071W-A | YPL086C |
| NP_001734 | NP_065178 | YBR109C | YJR065C |
| NP_001249 | NP_001818 | YBR135W | YBR160W |
| NP_006243 | NP_006588 | YAL005C | YDR477W |
| NP_001354 | NP_127460 | YHR089C | YLR175W |
| NP_001354 | NP_008974 | YCL059C | YLR175W |
| NP_031381 | NP_036382 | YOR101W | YPL240C |
| NP_002567 | NP_006752 | YDR099W | YHL007C |
| NP_002097 | NP_003698 | YDR190C | YOL012C |
| NP_006436 | NP_037417 | YHR165C | YNR053C |
| NP_006157 | NP_055038 | YBL021C | YOR358W |
| NP_000928 | NP_689407 | YDL140C | YPL204W |
| NP_000958 | NP_002931 | YDR091C | YOR063W |
| NP_000971 | NP_002701 | YER133W | YMR242C |
| NP_002778 | NP_002783 | YML092C | YOL038W |
| NP_054753 | NP_853551 | YJL115W | YJR068W |
| NP_002097 | NP_003510 | YDR224C | YOL012C |
| NP_004955 | NP_061944 | YNL097C | YNL330C |
| NP_003698 | NP_006653 | YDR190C | YDR334W |
| NP_002795 | NP_006494 | YDR394W | YOR117W |
| NP_002098 | NP_003510 | YBR010W | YDR224C |
| NP_068803 | NP_115572 | YBR009C | YGL150C |
| NP_000928 | NP_006225 | YDL140C | YOL005C |
| NP_002085 | NP_004721 | YBR143C | YDR172W |
| NP_001002 | NP_008974 | YCL059C | YOR096W |
| NP_000975 | NP_031381 | YOL127W | YPL240C |
| NP_001249 | NP_003494 | YBR160W | YDL017W |
| NP_001818 | NP_002794 | YBR135W | YKL145W |
| NP_001406 | NP_056988 | YAL035W | YER025W |
| NP_001092 | NP_001403 | YFL039C | YMR260C |
| NP_001485 | NP_004152 | YER136W | YFL038C |
| NP_002781 | NP_002788 | YGR253C | YPR103W |
| NP_002097 | NP_003185 | YER148W | YOL012C |
| NP_002701 | NP_004209 | YER031C | YER133W |
| NP_004651 | NP_056175 | YJL050W | YOR204W |
| NP_002147 | NP_031381 | YLR259C | YPL240C |
| NP_000928 | NP_001249 | YBR160W | YDL140C |
| NP_002097 | NP_006653 | YDR334W | YOL012C |
| NP_008974 | NP_055568 | YCL059C | YPL217C |
| NP_001020 | NP_036454 | YER131W | YER146W |
| NP_000958 | NP_002077 | YOR063W | YPR154W |

| | | | |
|---|---|---|---|
| NP_001015 | NP_008974 | YCL059C | YKR057W |
| NP_006588 | NP_031381 | YAL005C | YPL240C |
| NP_001485 | NP_004628 | YER136W | YML001W |
| NP_002098 | NP_068803 | YBR009C | YBR010W |
| NP_003185 | NP_054753 | YER148W | YJL115W |
| NP_003185 | NP_006657 | YER148W | YPL235W |
| NP_002788 | NP_057067 | YBR082C | YPR103W |
| NP_003510 | NP_068803 | YBR009C | YDR224C |
| NP_008986 | NP_060552 | YOR116C | YOR207C |
| NP_057078 | NP_733779 | YDR328C | YEL051W |
| NP_002701 | NP_004949 | YER133W | YKL203C |
| NP_002779 | NP_002782 | YGL011C | YOR362C |
| NP_001092 | NP_002097 | YFL039C | YOL012C |
| NP_002788 | NP_003327 | YGL058W | YPR103W |
| NP_002496 | NP_006157 | YBL021C | YGL237C |
| NP_004209 | NP_031381 | YER031C | YPL240C |
| NP_057067 | NP_057560 | YBR082C | YDL008W |
| NP_004388 | NP_061874 | YDL160C | YGL173C |
| NP_002780 | NP_002788 | YGR135W | YPR103W |
| NP_001485 | NP_942599 | YER136W | YLR262C |
| NP_001006 | NP_008974 | YBR048W | YCL059C |
| NP_002866 | NP_003570 | YER095W | YGL163C |
| NP_002379 | NP_006730 | YEL032W | YLR274W |
| NP_004222 | NP_057078 | YEL051W | YGR020C |
| NP_006243 | NP_066289 | YDR477W | YLL039C |
| NP_003698 | NP_115572 | YDR190C | YGL150C |
| NP_002782 | NP_002783 | YGL011C | YOL038W |
| NP_002780 | NP_005866 | YGR135W | YNL244C |
| NP_002786 | NP_002790 | YER094C | YOR157C |
| NP_002096 | NP_115572 | YDR225W | YGL150C |
| NP_002096 | NP_003698 | YDR190C | YDR225W |
| NP_003907 | NP_115882 | YLR170C | YPL259C |
| NP_000928 | NP_003325 | YDL140C | YKL210W |
| NP_054753 | NP_852136 | YJL115W | YOL094C |
| NP_000958 | NP_002701 | YER133W | YOR063W |
| NP_005338 | NP_037468 | YJL034W | YLR378C |
| NP_001010 | NP_002701 | YER133W | YJL190C |
| NP_002778 | NP_002781 | YGR253C | YML092C |
| NP_002778 | NP_002790 | YML092C | YOR157C |
| NP_000976 | NP_037417 | YKL180W | YNR053C |
| NP_000039 | NP_031381 | YHR018C | YPL240C |
| NP_006588 | NP_008965 | YAL005C | YNL007C |
| NP_005796 | NP_037468 | YFR004W | YLR378C |
| NP_001427 | NP_219484 | YDL014W | YNL075W |
| NP_000928 | NP_066951 | YDL140C | YOR210W |
| NP_002098 | NP_003327 | YBR010W | YGL058W |
| NP_002779 | NP_002788 | YOR362C | YPR103W |
| NP_004209 | NP_055223 | YER031C | YKR068C |

| | | | |
|---|---|---|---|
| NP_002902 | NP_004387 | YMR080C | YNL112W |
| NP_002796 | NP_008882 | YBR198C | YGL048C |
| NP_004160 | NP_036454 | YER146W | YLR058C |
| NP_004955 | NP_005720 | YDR155C | YNL330C |
| NP_000282 | NP_031381 | YCR012W | YPL240C |
| NP_001681 | NP_733779 | YDL185W | YDR328C |
| NP_002100 | NP_031381 | YPL240C | YPR033C |
| NP_002777 | NP_002783 | YMR314W | YOL038W |
| NP_001340 | NP_031381 | YLL018C | YPL240C |
| NP_004152 | NP_055223 | YFL038C | YKR068C |
| NP_002778 | NP_002780 | YGR135W | YML092C |
| NP_057394 | NP_060552 | YDR045C | YOR207C |
| NP_002794 | NP_002796 | YGL048C | YKL145W |
| NP_003185 | XP_378195 | YER148W | YPL122C |
| NP_002098 | NP_054753 | YBR010W | YJL115W |
| NP_003185 | NP_003698 | YDR190C | YER148W |
| NP_001008 | NP_008974 | YCL059C | YDR064W |
| NP_002660 | NP_003901 | YCR063W | YPL151C |
| NP_002567 | NP_003510 | YDR224C | YHL007C |
| NP_002085 | NP_006588 | YAL005C | YDR172W |
| NP_001002 | NP_996662 | YBL071W-A | YOR096W |
| NP_002778 | NP_002782 | YGL011C | YML092C |
| NP_005991 | NP_006078 | YFL037W | YML085C |
| NP_000998 | NP_008974 | YCL059C | YHR203C |
| NP_003494 | NP_006730 | YDL017W | YLR274W |
| NP_004628 | NP_031381 | YML001W | YPL240C |
| NP_002559 | NP_056175 | YER165W | YJL050W |
| NP_006004 | NP_037417 | YLR075W | YNR053C |
| NP_001684 | NP_733779 | YBR127C | YDR328C |
| NP_002931 | NP_036555 | YDR091C | YIL133C |
| NP_002096 | NP_006653 | YDR225W | YDR334W |
| NP_002931 | NP_877578 | YDR091C | YML063W |
| NP_000652 | NP_003185 | YER148W | YNL067W |
| NP_003185 | NP_003510 | YDR224C | YER148W |
| NP_001734 | NP_055038 | YBR109C | YOR358W |
| NP_031381 | NP_060734 | YPL207W | YPL240C |
| NP_006383 | NP_008974 | YCL059C | YLR197W |
| NP_002779 | NP_002783 | YOL038W | YOR362C |
| NP_000958 | NP_037417 | YNR053C | YOR063W |
| NP_031381 | NP_689926 | YGL022W | YPL240C |
| NP_001000 | NP_008974 | YCL059C | YJR123W |
| NP_002779 | NP_002781 | YGR253C | YOR362C |
| NP_002780 | NP_002783 | YGR135W | YOL038W |
| NP_002945 | NP_009057 | YDL126C | YLR167W |
| NP_006436 | XP_498188 | YHR165C | YKL152C |
| NP_000963 | NP_002931 | YDR091C | YLL045C |
| NP_068803 | NP_874369 | YBR009C | YOR244W |
| NP_001681 | NP_001685 | YDL185W | YEL027W |

| | | | |
|---|---|---|---|
| NP_002796 | NP_005866 | YGL048C | YNL244C |
| NP_001681 | NP_004222 | YDL185W | YGR020C |
| NP_000963 | NP_002701 | YER133W | YLL045C |
| NP_000929 | NP_066951 | YOR151C | YOR210W |
| NP_000996 | NP_008974 | YCL059C | YNL178W |
| NP_000975 | NP_006089 | YMR116C | YOL127W |
| NP_031381 | NP_055223 | YKR068C | YPL240C |
| NP_009057 | NP_066289 | YDL126C | YLL039C |
| NP_002660 | NP_006436 | YHR165C | YPL151C |
| NP_036454 | NP_940933 | YER146W | YIL048W |
| NP_001681 | NP_001684 | YBR127C | YDL185W |
| NP_031381 | NP_942599 | YLR262C | YPL240C |
| NP_000981 | NP_008974 | YCL059C | YGL103W |
| NP_002793 | NP_002796 | YDL007W | YGL048C |
| NP_002096 | NP_002097 | YDR225W | YOL012C |
| NP_001403 | NP_005866 | YMR260C | YNL244C |
| NP_002796 | NP_002797 | YGL048C | YOR259C |
| NP_004748 | NP_031381 | YGL105W | YPL240C |
| NP_002096 | NP_002098 | YBR010W | YDR225W |
| NP_006243 | NP_006752 | YDR099W | YDR477W |
| NP_001681 | NP_057078 | YDL185W | YEL051W |
| NP_001734 | NP_005796 | YBR109C | YFR004W |
| NP_008974 | NP_078938 | YCL059C | YNL132W |
| NP_002797 | NP_006494 | YDR394W | YOR259C |
| NP_003698 | NP_006657 | YDR190C | YPL235W |
| NP_002782 | NP_002788 | YGL011C | YPR103W |
| NP_003698 | NP_031381 | YDR190C | YPL240C |
| NP_036454 | NP_061874 | YER146W | YGL173C |
| NP_055063 | NP_733779 | YDR328C | YOL133W |
| NP_003510 | NP_115572 | YDR224C | YGL150C |
| NP_067000 | NP_940933 | YBL026W | YIL048W |
| NP_001017 | NP_003185 | YER074W | YER148W |
| NP_000970 | NP_031381 | YOL120C | YPL240C |
| NP_000981 | NP_036454 | YER146W | YGL103W |
| NP_002931 | NP_006004 | YDR091C | YLR075W |
| NP_002796 | NP_003185 | YER148W | YGL048C |
| NP_001782 | NP_002567 | YHL007C | YLR229C |
| NP_006322 | NP_008974 | YCL059C | YLR186W |
| NP_006764 | NP_008974 | YCL059C | YMR290C |
| NP_000113 | NP_031381 | YIL143C | YPL240C |
| NP_000967 | NP_006004 | YEL054C | YLR075W |
| NP_002567 | NP_055815 | YHL007C | YNL161W |
| NP_002077 | NP_002783 | YOL038W | YPR154W |
| NP_001092 | NP_874369 | YFL039C | YOR244W |
| NP_002701 | NP_877578 | YER133W | YML063W |
| NP_002097 | NP_002098 | YBR010W | YOL012C |
| NP_031381 | XP_375085 | YHR070W | YPL240C |
| NP_006386 | NP_113600 | YBL078C | YHR171W |

| | | | |
|---|---|---|---|
| NP_002795 | NP_002796 | YGL048C | YOR117W |
| NP_003510 | NP_031381 | YDR224C | YPL240C |
| NP_003185 | NP_008882 | YBR198C | YER148W |
| NP_001249 | NP_060691 | YBR160W | YIL106W |
| NP_000265 | NP_031381 | YLR438W | YPL240C |
| NP_036454 | NP_067000 | YBL026W | YER146W |
| NP_006436 | NP_036454 | YER146W | YHR165C |
| NP_002777 | NP_002778 | YML092C | YMR314W |
| NP_002559 | NP_006588 | YAL005C | YER165W |
| NP_001677 | NP_004037 | YBL099W | YJR121W |
| NP_001354 | NP_031381 | YLR175W | YPL240C |
| NP_000975 | NP_008974 | YCL059C | YOL127W |
| NP_000929 | NP_068809 | YOR151C | YPR187W |
| NP_002098 | NP_006243 | YBR010W | YDR477W |
| NP_001427 | NP_061874 | YDL014W | YGL173C |
| NP_002096 | NP_068803 | YBR009C | YDR225W |
| NP_002793 | NP_006494 | YDL007W | YDR394W |
| NP_003185 | NP_031381 | YER148W | YPL240C |
| NP_001249 | NP_002379 | YBR160W | YEL032W |
| NP_000928 | NP_000929 | YDL140C | YOR151C |
| NP_004152 | NP_004209 | YER031C | YFL038C |
| NP_006494 | NP_872634 | YDR394W | YMR216C |
| NP_002097 | NP_031381 | YOL012C | YPL240C |
| NP_001003 | NP_008974 | YCL059C | YER102W |
| NP_002796 | NP_005796 | YFR004W | YGL048C |
| NP_852136 | NP_853551 | YJR068W | YOL094C |
| NP_001354 | NP_061118 | YHR072W-A | YLR175W |
| NP_001427 | NP_004999 | YDL014W | YEL026W |
| NP_005434 | NP_031381 | YKL001C | YPL240C |
| NP_001427 | NP_061118 | YDL014W | YHR072W-A |
| NP_037468 | NP_689926 | YGL022W | YLR378C |
| NP_002096 | NP_003185 | YDR225W | YER148W |
| NP_003325 | NP_057067 | YBR082C | YKL210W |
| NP_002098 | NP_874369 | YBR010W | YOR244W |
| NP_001734 | NP_066952 | YBR011C | YBR109C |
| NP_001485 | NP_057614 | YER136W | YFL005W |
| NP_055288 | NP_689407 | YPL204W | YPL266W |
| NP_001734 | NP_057177 | YBR109C | YGR034W |
| NP_006421 | NP_031381 | YDL143W | YPL240C |
| NP_036565 | NP_116147 | YMR288W | YPR094W |
| NP_001092 | NP_003698 | YDR190C | YFL039C |
| NP_002778 | NP_002795 | YML092C | YOR117W |
| NP_001010 | NP_008974 | YCL059C | YJL190C |
| NP_002097 | NP_068803 | YBR009C | YOL012C |
| NP_031381 | NP_477515 | YER100W | YPL240C |
| NP_004152 | NP_004573 | YFL038C | YPR176C |
| NP_006653 | NP_006657 | YDR334W | YPL235W |
| NP_000969 | NP_003185 | YER117W | YER148W |

| | | | |
|---|---|---|---|
| NP_004153 | NP_060242 | YOR070C | YOR089C |
| NP_004651 | NP_127460 | YHR089C | YOR204W |
| NP_004068 | NP_005909 | YKL085W | YNR001C |
| NP_004651 | NP_061874 | YGL173C | YOR204W |
| NP_003324 | NP_031381 | YIL148W | YPL240C |
| NP_004757 | NP_031381 | YGL137W | YPL240C |
| NP_002085 | NP_002902 | YDR172W | YMR080C |
| NP_031381 | NP_116045 | YMR128W | YPL240C |
| NP_002779 | NP_005866 | YNL244C | YOR362C |
| NP_001406 | NP_067000 | YBL026W | YER025W |
| NP_001734 | NP_002781 | YBR109C | YGR253C |
| NP_001092 | NP_006657 | YFL039C | YPL235W |
| NP_002795 | NP_002797 | YOR117W | YOR259C |
| NP_001427 | NP_008974 | YCL059C | YDL014W |
| NP_001249 | NP_001779 | YBR160W | YLR314C |
| NP_002085 | NP_003185 | YDR172W | YER148W |
| NP_001001 | NP_036454 | YER146W | YPL090C |
| NP_002777 | NP_002779 | YMR314W | YOR362C |
| NP_001485 | NP_004209 | YER031C | YER136W |
| NP_001013 | NP_996662 | YBL071W-A | YOL121C |
| NP_001092 | NP_006653 | YDR334W | YFL039C |
| NP_002777 | NP_002788 | YMR314W | YPR103W |
| NP_002077 | NP_066289 | YLL039C | YPR154W |
| NP_001734 | NP_808227 | YBR109C | YOR061W |
| NP_002794 | NP_005796 | YFR004W | YKL145W |
| NP_001427 | NP_006383 | YDL014W | YLR197W |
| NP_006422 | NP_110379 | YDR212W | YIL142W |
| NP_001403 | NP_056988 | YAL035W | YMR260C |
| NP_000115 | NP_000391 | YER171W | YJR035W |
| NP_002788 | NP_002796 | YGL048C | YPR103W |
| NP_000959 | NP_008974 | YBR031W | YCL059C |
| NP_000987 | NP_002701 | YER133W | YOR234C |
| NP_001782 | NP_066289 | YLL039C | YLR229C |
| NP_002902 | NP_004721 | YBR143C | YMR080C |
| NP_001249 | NP_006236 | YBR160W | YOR014W |
| NP_000981 | NP_037417 | YGL103W | YNR053C |
| NP_005709 | NP_065178 | YJR065C | YKL013C |
| NP_004651 | NP_689407 | YOR204W | YPL204W |
| NP_006316 | NP_006588 | YAL005C | YLR293C |
| NP_031381 | NP_722540 | YDR265W | YPL240C |
| NP_002778 | NP_002779 | YML092C | YOR362C |
| NP_004999 | NP_006383 | YEL026W | YLR197W |
| NP_002777 | NP_542165 | YDL084W | YMR314W |
| NP_002795 | NP_005796 | YFR004W | YOR117W |
| NP_002783 | NP_002788 | YOL038W | YPR103W |
| NP_001249 | NP_002567 | YBR160W | YHL007C |
| NP_000113 | NP_000391 | YER171W | YIL143C |
| NP_004628 | NP_060242 | YML001W | YOR070C |

| | | | |
|---|---|---|---|
| NP_000928 | NP_003327 | YDL140C | YGL058W |
| NP_000928 | XP_049380 | YDL140C | YHR119W |
| NP_002496 | NP_055038 | YGL237C | YOR358W |
| NP_002097 | NP_006657 | YOL012C | YPL235W |
| NP_001249 | NP_112494 | YBR160W | YGL216W |
| NP_031381 | NP_057501 | YPL059W | YPL240C |
| NP_001092 | NP_689996 | YDL178W | YFL039C |
| NP_002778 | NP_002794 | YKL145W | YML092C |
| NP_001249 | NP_002796 | YBR160W | YGL048C |
| NP_031381 | NP_056492 | YFR024C-A | YPL240C |
| NP_003185 | NP_808227 | YER148W | YOR061W |
| NP_000391 | XP_378195 | YER171W | YPL122C |
| NP_001406 | NP_036454 | YER025W | YER146W |
| NP_001092 | NP_031381 | YFL039C | YPL240C |
| NP_002622 | NP_031381 | YHR183W | YPL240C |
| NP_000928 | NP_068809 | YDL140C | YPR187W |
| NP_006750 | NP_031381 | YKL035W | YPL240C |
| NP_000928 | NP_003185 | YDL140C | YER148W |
| NP_002786 | NP_002788 | YER094C | YPR103W |
| NP_004949 | NP_463460 | YKL203C | YNL135C |
| NP_031381 | NP_733779 | YDR328C | YPL240C |
| NP_001354 | NP_001427 | YDL014W | YLR175W |
| NP_002788 | NP_002790 | YOR157C | YPR103W |
| NP_031381 | NP_057291 | YLR277C | YPL240C |
| NP_002796 | NP_006494 | YDR394W | YGL048C |
| NP_003185 | NP_055288 | YER148W | YPL266W |
| NP_002701 | NP_057291 | YER133W | YLR277C |
| NP_000998 | NP_002931 | YDR091C | YHR203C |
| NP_061874 | NP_067000 | YBL026W | YGL173C |
| NP_006657 | NP_115572 | YGL150C | YPL235W |
| NP_002037 | NP_031381 | YGR192C | YPL240C |
| NP_001427 | NP_127460 | YDL014W | YHR089C |
| NP_002701 | NP_004332 | YER133W | YJL130C |
| NP_002096 | NP_003510 | YDR224C | YDR225W |
| NP_001092 | NP_115572 | YFL039C | YGL150C |
| NP_002736 | NP_031381 | YGR040W | YPL240C |
| NP_001024 | NP_067000 | YBL026W | YIL066C |
| NP_001374 | NP_996662 | YBL071W-A | YIL103W |
| NP_000959 | NP_002931 | YBR031W | YDR091C |
| NP_005713 | NP_065178 | YDL029W | YJR065C |
| NP_057614 | NP_060242 | YFL005W | YOR070C |
| NP_031381 | NP_079272 | YLR069C | YPL240C |
| NP_000113 | XP_378195 | YIL143C | YPL122C |
| NP_003327 | NP_003510 | YDR224C | YGL058W |
| NP_001407 | NP_031381 | YJL138C | YPL240C |
| NP_003185 | NP_006078 | YER148W | YFL037W |
| NP_004152 | NP_060242 | YFL038C | YOR070C |
| NP_001485 | NP_004153 | YER136W | YOR089C |

| | | | |
|---|---|---|---|
| NP_002098 | NP_115572 | YBR010W | YGL150C |
| NP_008974 | NP_116045 | YCL059C | YMR128W |
| NP_001249 | NP_002794 | YBR160W | YKL145W |
| NP_061118 | NP_127460 | YHR072W-A | YHR089C |
| NP_001003 | NP_002931 | YDR091C | YER102W |
| NP_002783 | NP_002794 | YKL145W | YOL038W |

## Supplementary Table 9. Novel GO functional predictions for yeast proteins.

| protein | GO prediction | p-value | GO annotation |
|---|---|---|---|
| YBR276C | GO:0009059 | 9.28E-07 | macromolecule biosynthesis |
| YDR494W | GO:0050791 | 1.01E-06 | regulation of physiological process |
| YDR509W | GO:0051276 | 1.15E-06 | chromosome organization and biogenesis |
| YEL024W | GO:0006403 | 0 | RNA localization |
| YEL066W | GO:0050791 | 1.10E-06 | regulation of physiological process |
| YHR036W | GO:0006403 | 0 | RNA localization |
| YKL157W | GO:0050791 | 1.01E-06 | regulation of physiological process |
| YLR120C | GO:0016070 | 7.48E-07 | RNA metabolism |
| YMR237W | GO:0016070 | 7.48E-07 | RNA metabolism |
| YOR128C | GO:0051276 | 1.15E-06 | chromosome organization and biogenesis |
| YPL276W | GO:0050791 | 1.01E-06 | regulation of physiological process |
| YPR048W | GO:0050791 | 8.79E-07 | regulation of physiological process |
| YHR020W | GO:0007049 | 1.35E-07 | cell cycle |
| YPR086W | GO:0009058 | 4.84E-08 | biosynthesis |
| YER092W | GO:0051276 | 1.07E-06 | chromosome organization and biogenesis |
| YGL174W | GO:0016070 | 2.71E-07 | RNA metabolism |
| YIR018W | GO:0051276 | 1.15E-06 | chromosome organization and biogenesis |
| YKL205W | GO:0009059 | 9.10E-07 | macromolecule biosynthesis |
| YLR215C | GO:0009059 | 9.10E-07 | macromolecule biosynthesis |
| YMR057C | GO:0016070 | 2.71E-07 | RNA metabolism |
| YBR287W | GO:0051276 | 1.14E-06 | chromosome organization and biogenesis |
| YDR256C | GO:0009056 | 5.28E-12 | catabolism |
| YDR504C | GO:0009058 | 5.72E-07 | biosynthesis |
| YBL015W | GO:0007049 | 1.40E-07 | cell cycle |
| YDL072C | GO:0016043 | 8.70E-08 | cell organization and biogenesis |
| YDL135C | GO:0051301 | 0 | cell division |
| YER083C | GO:0042254 | 4.30E-07 | ribosome biogenesis and assembly |
| YGL234W | GO:0007049 | 1.40E-07 | cell cycle |
| YJL098W | GO:0050791 | 2.10E-07 | regulation of physiological process |
| YJR091C | GO:0051301 | 0 | cell division |
| YNL059C | GO:0051276 | 1.07E-06 | chromosome organization and biogenesis |
| YOR174W | GO:0050791 | 1.46E-07 | regulation of physiological process |
| YOR298W | GO:0007049 | 1.40E-07 | cell cycle |
| YPR017C | GO:0007028 | 4.03E-07 | cytoplasm organization and biogenesis |
| YGL081W | GO:0007028 | 7.68E-08 | cytoplasm organization and biogenesis |
| YGR184C | GO:0044257 | 8.65E-12 | cellular protein catabolism |
| YJL031C | GO:0051179 | 1.62E-07 | localization |
| YPR176C | GO:0051179 | 1.62E-07 | localization |
| YLR076C | GO:0044248 | 2.92E-11 | cellular catabolism |
| YDR271C | GO:0016043 | 1.10E-06 | cell organization and biogenesis |
| YJR082C | GO:0007028 | 1.95E-08 | cytoplasm organization and biogenesis |
| YKR021W | GO:0016043 | 1.10E-06 | cell organization and biogenesis |
| YLR241W | GO:0016043 | 2.05E-08 | cell organization and biogenesis |
| YNL040W | GO:0016043 | 2.05E-08 | cell organization and biogenesis |
| YPL177C | GO:0044257 | 8.65E-12 | cellular protein catabolism |

| | | | |
|---|---|---|---|
| Q0032 | GO:0007028 | 3.74E-09 | cytoplasm organization and biogenesis |
| YBL025W | GO:0044257 | 1.07E-11 | cellular protein catabolism |
| YBR204C | GO:0044248 | 5.12E-13 | cellular catabolism |
| YBR272C | GO:0044248 | 3.66E-12 | cellular catabolism |
| YDR489W | GO:0008380 | 3.95E-07 | RNA splicing |
| YHR161C | GO:0008380 | 3.95E-07 | RNA splicing |
| YIL085C | GO:0007028 | 4.80E-09 | cytoplasm organization and biogenesis |
| YIL131C | GO:0009058 | 1.25E-08 | biosynthesis |
| YLR325C | GO:0007028 | 4.80E-09 | cytoplasm organization and biogenesis |
| YLR356W | GO:0007028 | 1.93E-08 | cytoplasm organization and biogenesis |
| YMR108W | GO:0044257 | 7.72E-11 | cellular protein catabolism |
| YNL311C | GO:0044248 | 3.66E-12 | cellular catabolism |
| YPR028W | GO:0051179 | 8.11E-10 | localization |
| YHL030W | GO:0044248 | 3.34E-10 | cellular catabolism |
| YDR341C | GO:0008380 | 6.17E-07 | RNA splicing |
| YGL216W | GO:0008380 | 6.17E-07 | RNA splicing |
| YHR143W-A | GO:0050791 | 2.99E-08 | regulation of physiological process |
| YIL025C | GO:0050791 | 2.99E-08 | regulation of physiological process |
| YLR016C | GO:0008380 | 6.17E-07 | RNA splicing |
| YER044C-A | GO:0008380 | 6.60E-07 | RNA splicing |
| YER145C | GO:0044248 | 2.46E-10 | cellular catabolism |
| YGR061C | GO:0008380 | 3.95E-07 | RNA splicing |
| YJR021C | GO:0008380 | 6.60E-07 | RNA splicing |
| YLR386W | GO:0008380 | 3.95E-07 | RNA splicing |
| YMR133W | GO:0008380 | 6.60E-07 | RNA splicing |
| YMR154C | GO:0050791 | 1.69E-07 | regulation of physiological process |
| YPR180W | GO:0044248 | 2.46E-10 | cellular catabolism |
| YDR213W | GO:0008380 | 3.95E-07 | RNA splicing |
| YIL148W | GO:0009056 | 3.82E-10 | catabolism |
| YMR176W | GO:0008380 | 3.95E-07 | RNA splicing |
| YNR059W | GO:0009056 | 3.82E-10 | catabolism |
| YDR273W | GO:0044248 | 2.80E-10 | cellular catabolism |
| YMR032W | GO:0016043 | 5.35E-09 | cell organization and biogenesis |
| YMR039C | GO:0006379 | 3.38E-14 | mRNA cleavage |
| YMR316C-B | GO:0006379 | 3.38E-14 | mRNA cleavage |
| YNL127W | GO:0016070 | 8.30E-08 | RNA metabolism |
| YBL010C | GO:0008380 | 3.95E-07 | RNA splicing |
| YBR096W | GO:0044248 | 3.33E-10 | cellular catabolism |
| YGR052W | GO:0007059 | 0 | chromosome segregation |
| YHR156C | GO:0007049 | 1.35E-07 | cell cycle |
| YIL034C | GO:0044248 | 3.33E-10 | cellular catabolism |
| YPL060W | GO:0007059 | 0 | chromosome segregation |
| YBR082C | GO:0007059 | 0 | chromosome segregation |
| YHR191C | GO:0050896 | 5.50E-08 | response to stimulus |
| YKL034W | GO:0007059 | 0 | chromosome segregation |
| YBR245C | GO:0050791 | 4.98E-09 | regulation of physiological process |
| YDL044C | GO:0007059 | 0 | chromosome segregation |
| YDR121W | GO:0007049 | 1.35E-07 | cell cycle |

| | | | |
|---|---|---|---|
| YIL007C | GO:0044248 | 2.80E-10 | cellular catabolism |
| YKR001C | GO:0050791 | 4.98E-09 | regulation of physiological process |
| YLL051C | GO:0007059 | 0 | chromosome segregation |
| YNL215W | GO:0051276 | 7.15E-08 | chromosome organization and biogenesis |
| YOL130W | GO:0008380 | 1.72E-07 | RNA splicing |
| YOR253W | GO:0044248 | 2.10E-10 | cellular catabolism |
| YKR024C | GO:0044248 | 3.13E-10 | cellular catabolism |
| YLR024C | GO:0044248 | 2.80E-10 | cellular catabolism |
| YLR390W | GO:0007049 | 1.24E-07 | cell cycle |
| YMR181C | GO:0007049 | 1.24E-07 | cell cycle |
| YOL076W | GO:0044248 | 3.13E-10 | cellular catabolism |
| YBL091C | GO:0007049 | 1.35E-07 | cell cycle |
| YCL042W | GO:0007049 | 1.35E-07 | cell cycle |
| YNL175C | GO:0007028 | 2.75E-09 | cytoplasm organization and biogenesis |
| YGL251C | GO:0016070 | 8.30E-08 | RNA metabolism |
| YMR092C | GO:0007059 | 0 | chromosome segregation |
| YNL176C | GO:0007059 | 0 | chromosome segregation |
| YBR086C | GO:0008380 | 3.95E-07 | RNA splicing |
| YDR113C | GO:0008054 | 0 | cyclin catabolism |
| YPR052C | GO:0006368 | 4.34E-13 | RNA elongation from Pol II promoter |
| YDL237W | GO:0051276 | 1.77E-08 | chromosome organization and biogenesis |
| YAR042W | GO:0016043 | 7.98E-08 | cell organization and biogenesis |
| YFL039C | GO:0051276 | 4.37E-09 | chromosome organization and biogenesis |
| YLR397C | GO:0016043 | 7.98E-08 | cell organization and biogenesis |
| YLR410W | GO:0006368 | 2.54E-10 | RNA elongation from Pol II promoter |
| YOR292C | GO:0006368 | 2.54E-10 | RNA elongation from Pol II promoter |
| YPL013C | GO:0016043 | 7.98E-08 | cell organization and biogenesis |
| YIL160C | GO:0007031 | 0 | peroxisome organization and biogenesis |
| YER131W | GO:0016070 | 7.48E-07 | RNA metabolism |
| YHR204W | GO:0016043 | 7.22E-08 | cell organization and biogenesis |
| YLL027W | GO:0016070 | 7.48E-07 | RNA metabolism |
| YLR269C | GO:0016070 | 7.48E-07 | RNA metabolism |
| YPL105C | GO:0008380 | 2.42E-08 | RNA splicing |
| YGL143C | GO:0016070 | 7.48E-07 | RNA metabolism |
| YGR047C | GO:0006379 | 0 | mRNA cleavage |
| YHL008C | GO:0016070 | 7.48E-07 | RNA metabolism |
| YNL145W | GO:0016070 | 7.48E-07 | RNA metabolism |
| YGR214W | GO:0008380 | 4.33E-08 | RNA splicing |
| YLR039C | GO:0009058 | 4.33E-08 | biosynthesis |
| YOR141C | GO:0051276 | 7.15E-08 | chromosome organization and biogenesis |
| YBR267W | GO:0007028 | 7.68E-08 | cytoplasm organization and biogenesis |
| YDL102W | GO:0008054 | 4.52E-13 | cyclin catabolism |
| YER148W | GO:0043044 | 0 | ATP-dependent chromatin remodeling |
| YGR048W | GO:0019725 | 0 | cell homeostasis |
| YJL122W | GO:0007028 | 7.68E-08 | cytoplasm organization and biogenesis |
| YJR006W | GO:0008054 | 4.52E-13 | cyclin catabolism |
| YJR043C | GO:0008054 | 4.52E-13 | cyclin catabolism |
| YNL102W | GO:0008054 | 4.52E-13 | cyclin catabolism |

| | | | |
|---|---|---|---|
| YDR390C | GO:0019725 | 0 | cell homeostasis |
| YER072W | GO:0019725 | 0 | cell homeostasis |
| YJL012C | GO:0019725 | 0 | cell homeostasis |
| YPL019C | GO:0019725 | 0 | cell homeostasis |
| YBR094W | GO:0016070 | 7.48E-07 | RNA metabolism |
| YDR265W | GO:0007031 | 0 | peroxisome organization and biogenesis |
| YJL210W | GO:0007031 | 0 | peroxisome organization and biogenesis |
| YLL045C | GO:0007028 | 7.68E-08 | cytoplasm organization and biogenesis |
| YLR264W | GO:0016070 | 7.48E-07 | RNA metabolism |
| YMR026C | GO:0007031 | 0 | peroxisome organization and biogenesis |
| YNL214W | GO:0007031 | 0 | peroxisome organization and biogenesis |
| YOR167C | GO:0016070 | 7.48E-07 | RNA metabolism |
| YFL066C | GO:0008380 | 1.37E-08 | RNA splicing |
| YGL142C | GO:0051276 | 2.83E-07 | chromosome organization and biogenesis |
| YLR124W | GO:0051276 | 2.83E-07 | chromosome organization and biogenesis |
| YMR106C | GO:0043285 | 2.10E-08 | biopolymer catabolism |
| YNL128W | GO:0043285 | 2.10E-08 | biopolymer catabolism |
| YEL049W | GO:0008380 | 1.14E-08 | RNA splicing |
| YGL063W | GO:0051276 | 2.83E-07 | chromosome organization and biogenesis |
| YAL054C | GO:0007031 | 6.61E-14 | peroxisome organization and biogenesis |
| YBR172C | GO:0008380 | 1.11E-08 | RNA splicing |
| YDR310C | GO:0009100 | 3.76E-11 | glycoprotein metabolism |
| YHL003C | GO:0051276 | 2.83E-07 | chromosome organization and biogenesis |
| YKL008C | GO:0051276 | 2.83E-07 | chromosome organization and biogenesis |
| YKR011C | GO:0009100 | 3.76E-11 | glycoprotein metabolism |
| YLR049C | GO:0007031 | 6.61E-14 | peroxisome organization and biogenesis |
| YLR465C | GO:0007031 | 6.61E-14 | peroxisome organization and biogenesis |
| YML035C | GO:0007031 | 6.61E-14 | peroxisome organization and biogenesis |
| YMR298W | GO:0051276 | 2.83E-07 | chromosome organization and biogenesis |
| YOL068C | GO:0009100 | 3.76E-11 | glycoprotein metabolism |
| YPR071W | GO:0007031 | 6.61E-14 | peroxisome organization and biogenesis |
| YPR119W | GO:0019725 | 0 | cell homeostasis |
| YBR171W | GO:0009100 | 5.98E-11 | glycoprotein metabolism |
| YLR292C | GO:0009100 | 5.98E-11 | glycoprotein metabolism |
| YLR305C | GO:0008380 | 1.05E-08 | RNA splicing |
| YMR078C | GO:0050896 | 5.50E-08 | response to stimulus |
| YOL016C | GO:0009100 | 6.06E-10 | glycoprotein metabolism |
| YOR007C | GO:0009100 | 6.06E-10 | glycoprotein metabolism |
| YOR144C | GO:0050896 | 5.50E-08 | response to stimulus |
| YPL094C | GO:0009100 | 5.98E-11 | glycoprotein metabolism |
| YJR076C | GO:0019725 | 0 | cell homeostasis |
| YKL048C | GO:0019725 | 0 | cell homeostasis |
| YPR107C | GO:0008213 | 1.93E-14 | protein amino acid alkylation |
| YAL043C | GO:0008213 | 1.93E-14 | protein amino acid alkylation |
| YBR288C | GO:0006944 | 1.18E-13 | membrane fusion |
| YCR038C | GO:0008213 | 4.72E-14 | protein amino acid alkylation |
| YDR140W | GO:0008213 | 1.93E-14 | protein amino acid alkylation |
| YDR251W | GO:0006944 | 6.94E-14 | membrane fusion |

| | | | |
|---|---|---|---|
| YGL094C | GO:0006944 | 1.83E-13 | membrane fusion |
| YGR156W | GO:0008213 | 1.93E-14 | protein amino acid alkylation |
| YIL151C | GO:0008213 | 1.93E-14 | protein amino acid alkylation |
| YKL025C | GO:0006944 | 1.83E-13 | membrane fusion |
| YKL059C | GO:0008213 | 1.93E-14 | protein amino acid alkylation |
| YMR291W | GO:0006944 | 4.62E-14 | membrane fusion |
| YNL222W | GO:0008213 | 1.93E-14 | protein amino acid alkylation |
| YNL317W | GO:0008213 | 1.93E-14 | protein amino acid alkylation |
| YPL057C | GO:0006944 | 6.94E-14 | membrane fusion |
| YPL245W | GO:0006473 | 1.86E-10 | protein amino acid acetylation |
| YPL262W | GO:0006944 | 4.62E-14 | membrane fusion |
| YPR122W | GO:0008213 | 4.72E-14 | protein amino acid alkylation |
| YBL066C | GO:0019725 | 0 | cell homeostasis |
| YDR532C | GO:0006473 | 4.02E-10 | protein amino acid acetylation |
| YGL043W | GO:0006473 | 9.83E-11 | protein amino acid acetylation |
| YGR117C | GO:0019725 | 0 | cell homeostasis |
| YIL134W | GO:0006473 | 4.02E-10 | protein amino acid acetylation |
| YCR036W | GO:0007031 | 6.61E-14 | peroxisome organization and biogenesis |
| YCR077C | GO:0007031 | 6.61E-14 | peroxisome organization and biogenesis |
| YDL029W | GO:0007049 | 3.20E-10 | cell cycle |
| YDL139C | GO:0007031 | 6.61E-14 | peroxisome organization and biogenesis |
| YDL196W | GO:0006378 | 2.08E-09 | mRNA polyadenylylation |
| YER071C | GO:0006378 | 2.08E-09 | mRNA polyadenylylation |
| YGR105W | GO:0019725 | 0 | cell homeostasis |
| YGR218W | GO:0007031 | 3.98E-13 | peroxisome organization and biogenesis |
| YGR242W | GO:0007031 | 6.61E-14 | peroxisome organization and biogenesis |
| YLR063W | GO:0007031 | 6.61E-14 | peroxisome organization and biogenesis |
| YDR315C | GO:0008380 | 2.04E-07 | RNA splicing |
| YDR482C | GO:0008380 | 2.04E-07 | RNA splicing |
| YLR323C | GO:0008380 | 2.04E-07 | RNA splicing |
| YPL064C | GO:0008380 | 2.04E-07 | RNA splicing |
| YDR247W | GO:0008380 | 2.04E-07 | RNA splicing |
| YIL162W | GO:0045047 | 4.65E-11 | protein-ER targeting |
| YNL154C | GO:0007028 | 1.80E-08 | cytoplasm organization and biogenesis |
| YJL099W | GO:0008380 | 9.82E-08 | RNA splicing |
| YPL077C | GO:0008380 | 2.04E-07 | RNA splicing |
| YCR072C | GO:0007028 | 3.74E-09 | cytoplasm organization and biogenesis |
| YGL021W | GO:0008380 | 1.60E-07 | RNA splicing |
| YGR003W | GO:0008380 | 2.04E-07 | RNA splicing |
| YJL158C | GO:0008380 | 9.82E-08 | RNA splicing |
| YLR133W | GO:0008380 | 1.60E-07 | RNA splicing |
| YMR139W | GO:0008380 | 1.60E-07 | RNA splicing |
| YPL240C | GO:0007031 | 1.29E-13 | peroxisome organization and biogenesis |
| YBR162W-A | GO:0008380 | 1.72E-07 | RNA splicing |
| YGL128C | GO:0008380 | 9.82E-08 | RNA splicing |
| YLR090W | GO:0008380 | 1.72E-07 | RNA splicing |
| YBR193C | GO:0050791 | 2.40E-08 | regulation of physiological process |
| YDL089W | GO:0016192 | 2.29E-07 | vesicle-mediated transport |

| | | | |
|---|---|---|---|
| YDR034C | GO:0007031 | 6.61E-14 | peroxisome organization and biogenesis |
| YGR004W | GO:0016192 | 2.29E-07 | vesicle-mediated transport |
| YLR324W | GO:0016192 | 2.29E-07 | vesicle-mediated transport |
| YOR039W | GO:0006368 | 4.34E-13 | RNA elongation from Pol II promoter |
| YDR408C | GO:0008380 | 2.04E-07 | RNA splicing |
| YLR297W | GO:0008380 | 2.04E-07 | RNA splicing |
| YEL019C | GO:0044257 | 3.35E-08 | cellular protein catabolism |
| YEL034W | GO:0044257 | 3.35E-08 | cellular protein catabolism |
| YFL022C | GO:0044257 | 3.35E-08 | cellular protein catabolism |
| YMR284W | GO:0044257 | 3.35E-08 | cellular protein catabolism |
| YOR180C | GO:0007031 | 0 | peroxisome organization and biogenesis |
| YBR019C | GO:0006473 | 9.83E-11 | protein amino acid acetylation |
| YCR005C | GO:0006473 | 9.83E-11 | protein amino acid acetylation |
| YGR049W | GO:0008380 | 9.82E-08 | RNA splicing |
| YKL174C | GO:0006401 | 7.77E-16 | RNA catabolism |
| YLR284C | GO:0007031 | 0 | peroxisome organization and biogenesis |
| YNR010W | GO:0050791 | 2.40E-08 | regulation of physiological process |
| YBR010W | GO:0051276 | 7.15E-08 | chromosome organization and biogenesis |
| YGL241W | GO:0051276 | 7.15E-08 | chromosome organization and biogenesis |
| YDR283C | GO:0007028 | 3.74E-09 | cytoplasm organization and biogenesis |
| YNL091W | GO:0019222 | 1.77E-09 | regulation of metabolism |
| YNL164C | GO:0019222 | 1.77E-09 | regulation of metabolism |
| YDR260C | GO:0007059 | 0 | chromosome segregation |
| YIL029C | GO:0016070 | 2.05E-07 | RNA metabolism |
| YIR025W | GO:0007059 | 0 | chromosome segregation |
| YJL125C | GO:0009059 | 2.21E-07 | macromolecule biosynthesis |
| YKL209C | GO:0016070 | 2.05E-07 | RNA metabolism |
| YNL297C | GO:0016070 | 2.05E-07 | RNA metabolism |
| YOL087C | GO:0009058 | 4.33E-08 | biosynthesis |
| YJL010C | GO:0009059 | 2.18E-07 | macromolecule biosynthesis |
| YAL009W | GO:0019222 | 1.52E-09 | regulation of metabolism |
| YFR027W | GO:0007049 | 3.36E-09 | cell cycle |
| YGR102C | GO:0009058 | 1.39E-07 | biosynthesis |
| YHR004C | GO:0019222 | 1.52E-09 | regulation of metabolism |
| YMR165C | GO:0019222 | 1.52E-09 | regulation of metabolism |
| YDR194C | GO:0007028 | 6.74E-08 | cytoplasm organization and biogenesis |
| YHR180W | GO:0007028 | 6.74E-08 | cytoplasm organization and biogenesis |
| YIL004C | GO:0006944 | 0 | membrane fusion |
| YDR510W | GO:0051301 | 1.41E-10 | cell division |
| YLR078C | GO:0006944 | 0 | membrane fusion |
| YDR122W | GO:0006944 | 0 | membrane fusion |
| YGR054W | GO:0007028 | 2.09E-08 | cytoplasm organization and biogenesis |
| YIR026C | GO:0007028 | 1.46E-08 | cytoplasm organization and biogenesis |
| YKL180W | GO:0007028 | 1.46E-08 | cytoplasm organization and biogenesis |
| YNL132W | GO:0007028 | 2.09E-08 | cytoplasm organization and biogenesis |
| YOL102C | GO:0007028 | 2.09E-08 | cytoplasm organization and biogenesis |
| YML030W | GO:0006378 | 0 | mRNA polyadenylylation |
| YAL005C | GO:0043044 | 1.00E-11 | ATP-dependent chromatin remodeling |

| | | | |
|---|---|---|---|
| YIL139C | GO:0007049 | 3.36E-09 | cell cycle |
| YJL072C | GO:0006378 | 2.16E-10 | mRNA polyadenylylation |
| YLR256W | GO:0006378 | 2.16E-10 | mRNA polyadenylylation |
| YMR260C | GO:0006378 | 2.16E-10 | mRNA polyadenylylation |
| YNL064C | GO:0006378 | 2.16E-10 | mRNA polyadenylylation |
| YOL150C | GO:0006378 | 2.16E-10 | mRNA polyadenylylation |
| YOR146W | GO:0007049 | 3.36E-09 | cell cycle |
| YOR344C | GO:0006378 | 2.16E-10 | mRNA polyadenylylation |
| YPL167C | GO:0007049 | 3.36E-09 | cell cycle |
| YAR033W | GO:0006401 | 9.85E-12 | RNA catabolism |
| YBR084C-A | GO:0007028 | 6.76E-08 | cytoplasm organization and biogenesis |
| YDL229W | GO:0043044 | 1.00E-11 | ATP-dependent chromatin remodeling |
| YIL016W | GO:0043044 | 1.00E-11 | ATP-dependent chromatin remodeling |
| YPL106C | GO:0043044 | 1.00E-11 | ATP-dependent chromatin remodeling |
| YAR066W | GO:0007028 | 6.90E-08 | cytoplasm organization and biogenesis |
| YBL019W | GO:0043044 | 7.82E-12 | ATP-dependent chromatin remodeling |
| YBR113W | GO:0008380 | 5.46E-08 | RNA splicing |
| YDR074W | GO:0007028 | 6.90E-08 | cytoplasm organization and biogenesis |
| YER089C | GO:0006473 | 2.22E-10 | protein amino acid acetylation |
| YFL031W | GO:0006473 | 2.22E-10 | protein amino acid acetylation |
| YHR145C | GO:0043044 | 7.82E-12 | ATP-dependent chromatin remodeling |
| YBR088C | GO:0007049 | 3.36E-09 | cell cycle |
| YOR304W | GO:0006473 | 4.64E-12 | protein amino acid acetylation |
| YAR007C | GO:0007049 | 3.20E-10 | cell cycle |
| YDL078C | GO:0007031 | 2.78E-15 | peroxisome organization and biogenesis |
| YDR009W | GO:0051236 | 1.86E-11 | establishment of RNA localization |
| YLR433C | GO:0008380 | 2.72E-08 | RNA splicing |
| YPL248C | GO:0051236 | 1.86E-11 | establishment of RNA localization |
| YPR070W | GO:0050791 | 4.96E-09 | regulation of physiological process |
| YDL005C | GO:0050791 | 4.96E-09 | regulation of physiological process |
| YER066W | GO:0050791 | 4.96E-09 | regulation of physiological process |
| YHR147C | GO:0050791 | 4.96E-09 | regulation of physiological process |
| YBR190W | GO:0008380 | 4.11E-08 | RNA splicing |
| YDR159W | GO:0006473 | 4.64E-12 | protein amino acid acetylation |
| YOR257W | GO:0006473 | 4.64E-12 | protein amino acid acetylation |
| YER044C | GO:0007049 | 7.38E-09 | cell cycle |
| YGL001C | GO:0007049 | 7.38E-09 | cell cycle |
| YGR060W | GO:0007049 | 7.38E-09 | cell cycle |
| YBR205W | GO:0006378 | 1.19E-11 | mRNA polyadenylylation |
| YBR282W | GO:0006378 | 1.19E-11 | mRNA polyadenylylation |
| YDL064W | GO:0051301 | 1.41E-10 | cell division |
| YDR409W | GO:0051301 | 1.41E-10 | cell division |
| YER133W | GO:0006378 | 1.19E-11 | mRNA polyadenylylation |
| YGR187C | GO:0006378 | 1.19E-11 | mRNA polyadenylylation |
| YJL092W | GO:0051301 | 1.41E-10 | cell division |
| YKR055W | GO:0007091 | 1.21E-14 | mitotic metaphase/anaphase transition |
| YNL078W | GO:0051301 | 1.41E-10 | cell division |
| YOR156C | GO:0051301 | 1.41E-10 | cell division |

| | | | |
|---|---|---|---|
| YOR179C | GO:0006378 | 1.19E-11 | mRNA polyadenylylation |
| YDL094C | GO:0006378 | 4.28E-12 | mRNA polyadenylylation |
| YER143W | GO:0006944 | 0 | membrane fusion |
| YGR067C | GO:0044257 | 5.52E-10 | cellular protein catabolism |
| YGR225W | GO:0007059 | 0 | chromosome segregation |
| YHL035C | GO:0006378 | 4.28E-12 | mRNA polyadenylylation |
| YIL005W | GO:0007059 | 0 | chromosome segregation |
| YJL080C | GO:0044257 | 5.52E-10 | cellular protein catabolism |
| YLR226W | GO:0008380 | 4.57E-08 | RNA splicing |
| YNL085W | GO:0044257 | 5.52E-10 | cellular protein catabolism |
| YNL189W | GO:0006401 | 7.77E-16 | RNA catabolism |
| YPR161C | GO:0008380 | 4.57E-08 | RNA splicing |
| YAL064W | GO:0009100 | 1.73E-12 | glycoprotein metabolism |
| YBL105C | GO:0009100 | 2.93E-12 | glycoprotein metabolism |
| YDR012W | GO:0007028 | 1.26E-08 | cytoplasm organization and biogenesis |
| YDR021W | GO:0009100 | 1.73E-12 | glycoprotein metabolism |
| YDR086C | GO:0009100 | 1.73E-12 | glycoprotein metabolism |
| YER019C-A | GO:0009100 | 1.73E-12 | glycoprotein metabolism |
| YER087C-B | GO:0009100 | 1.73E-12 | glycoprotein metabolism |
| YGL141W | GO:0044248 | 3.80E-12 | cellular catabolism |
| YJR010C-A | GO:0009100 | 3.88E-12 | glycoprotein metabolism |
| YLR378C | GO:0009100 | 1.73E-12 | glycoprotein metabolism |
| YOR254C | GO:0009100 | 2.93E-12 | glycoprotein metabolism |
| YOR279C | GO:0009100 | 1.73E-12 | glycoprotein metabolism |
| YER105C | GO:0044257 | 7.72E-11 | cellular protein catabolism |
| YFL021W | GO:0044257 | 1.58E-10 | cellular protein catabolism |
| YJL030W | GO:0044257 | 7.72E-11 | cellular protein catabolism |
| YLR068W | GO:0044257 | 1.58E-10 | cellular protein catabolism |
| YMR276W | GO:0009056 | 1.06E-12 | catabolism |
| YOR160W | GO:0006997 | 0 | nuclear organization and biogenesis |
| YDR225W | GO:0006378 | 5.72E-13 | mRNA polyadenylylation |
| YGR161C-C | GO:0006997 | 0 | nuclear organization and biogenesis |
| YHL001W | GO:0006378 | 5.72E-13 | mRNA polyadenylylation |
| YMR182C | GO:0006378 | 5.72E-13 | mRNA polyadenylylation |
| YOR142W-A | GO:0006997 | 0 | nuclear organization and biogenesis |
| YBR105C | GO:0006378 | 8.99E-15 | mRNA polyadenylylation |
| YBR195C | GO:0006354 | 3.80E-13 | RNA elongation |
| YCL037C | GO:0006378 | 5.72E-13 | mRNA polyadenylylation |
| YDR063W | GO:0016043 | 1.82E-08 | cell organization and biogenesis |
| YDR117C | GO:0007028 | 9.16E-09 | cytoplasm organization and biogenesis |
| YER012W | GO:0006354 | 2.43E-14 | RNA elongation |
| YGL239C | GO:0016043 | 1.82E-08 | cell organization and biogenesis |
| YJL033W | GO:0006378 | 5.72E-13 | mRNA polyadenylylation |
| YKL006C-A | GO:0006944 | 0 | membrane fusion |
| YKL018W | GO:0006378 | 5.72E-13 | mRNA polyadenylylation |
| YNL183C | GO:0006354 | 3.80E-13 | RNA elongation |
| YOR171C | GO:0006378 | 8.99E-15 | mRNA polyadenylylation |
| YOR185C | GO:0006378 | 8.99E-15 | mRNA polyadenylylation |

| | | | |
|---|---|---|---|
| YDR314C | GO:0009056 | 7.72E-12 | catabolism |
| YDR496C | GO:0007028 | 9.16E-09 | cytoplasm organization and biogenesis |
| YIL035C | GO:0006354 | 1.40E-14 | RNA elongation |
| YKL185W | GO:0007028 | 9.16E-09 | cytoplasm organization and biogenesis |
| YOL108C | GO:0006352 | 5.40E-13 | transcription initiation |
| YOR005C | GO:0007028 | 5.65E-10 | cytoplasm organization and biogenesis |
| YOR320C | GO:0016070 | 5.62E-08 | RNA metabolism |
| YPL146C | GO:0007028 | 9.16E-09 | cytoplasm organization and biogenesis |
| YAL055W | GO:0007031 | 0 | peroxisome organization and biogenesis |
| YAL056W | GO:0051236 | 1.40E-12 | establishment of RNA localization |
| YBL078C | GO:0006944 | 0 | membrane fusion |
| YDL227C | GO:0009056 | 9.35E-13 | catabolism |
| YER020W | GO:0051236 | 1.40E-12 | establishment of RNA localization |
| YGL121C | GO:0051236 | 1.40E-12 | establishment of RNA localization |
| YIL130W | GO:0008380 | 1.17E-08 | RNA splicing |
| YAL032C | GO:0008380 | 4.11E-08 | RNA splicing |
| YAL034C | GO:0000114 | 7.53E-14 | G1-specific transcription in mitotic cell cycle |
| YDR321W | GO:0006944 | 0 | membrane fusion |
| YFL056C | GO:0006944 | 0 | membrane fusion |
| YGL180W | GO:0006379 | 1.43E-13 | mRNA cleavage |
| YKL201C | GO:0000114 | 7.53E-14 | G1-specific transcription in mitotic cell cycle |
| YLR140W | GO:0007028 | 1.50E-08 | cytoplasm organization and biogenesis |
| YLR423C | GO:0006379 | 1.43E-13 | mRNA cleavage |
| YNL034W | GO:0007028 | 9.16E-09 | cytoplasm organization and biogenesis |
| YNL320W | GO:0007028 | 1.50E-08 | cytoplasm organization and biogenesis |
| YPL198W | GO:0007028 | 1.50E-08 | cytoplasm organization and biogenesis |
| YPR049C | GO:0006379 | 1.43E-13 | mRNA cleavage |
| YPR185W | GO:0006379 | 1.43E-13 | mRNA cleavage |
| YAL002W | GO:0006944 | 0 | membrane fusion |
| YCL045C | GO:0051236 | 1.10E-12 | establishment of RNA localization |
| YDR120C | GO:0051236 | 1.10E-12 | establishment of RNA localization |
| YDR189W | GO:0006944 | 0 | membrane fusion |
| YER164W | GO:0006473 | 2.20E-14 | protein amino acid acetylation |
| YGL058W | GO:0006354 | 2.43E-14 | RNA elongation |
| YGL130W | GO:0006354 | 3.80E-13 | RNA elongation |
| YHR009C | GO:0006354 | 6.36E-14 | RNA elongation |
| YJL153C | GO:0007028 | 1.45E-08 | cytoplasm organization and biogenesis |
| YJL190C | GO:0007028 | 1.45E-08 | cytoplasm organization and biogenesis |
| YJR123W | GO:0051236 | 1.10E-12 | establishment of RNA localization |
| YLL010C | GO:0044248 | 2.92E-11 | cellular catabolism |
| YLR216C | GO:0050791 | 4.34E-09 | regulation of physiological process |
| YML029W | GO:0043044 | 8.08E-13 | ATP-dependent chromatin remodeling |
| YML051W | GO:0051236 | 1.10E-12 | establishment of RNA localization |
| YOL080C | GO:0006354 | 2.43E-14 | RNA elongation |
| YOR043W | GO:0044248 | 2.92E-11 | cellular catabolism |
| YOR074C | GO:0006354 | 6.36E-14 | RNA elongation |
| YPL139C | GO:0006473 | 4.79E-13 | protein amino acid acetylation |
| YPL181W | GO:0006473 | 4.79E-13 | protein amino acid acetylation |

| | | | |
|---|---|---|---|
| YPL228W | GO:0006354 | 3.80E-13 | RNA elongation |
| YBR102C | GO:0006944 | 0 | membrane fusion |
| YDL006W | GO:0006473 | 2.21E-12 | protein amino acid acetylation |
| YGL149W | GO:0044248 | 2.92E-11 | cellular catabolism |
| YGR261C | GO:0006944 | 0 | membrane fusion |
| YIL009C-A | GO:0044248 | 2.92E-11 | cellular catabolism |
| YIR023W | GO:0006473 | 2.21E-12 | protein amino acid acetylation |
| YLR196W | GO:0007028 | 1.26E-08 | cytoplasm organization and biogenesis |
| YML055W | GO:0046903 | 1.44E-15 | secretion |
| YPL259C | GO:0007028 | 1.26E-08 | cytoplasm organization and biogenesis |
| YPR032W | GO:0006944 | 0 | membrane fusion |
| YAL014C | GO:0006944 | 0 | membrane fusion |
| YBL042C | GO:0044248 | 3.06E-11 | cellular catabolism |
| YBL072C | GO:0007028 | 1.26E-08 | cytoplasm organization and biogenesis |
| YBR207W | GO:0044248 | 3.06E-11 | cellular catabolism |
| YDL028C | GO:0007059 | 7.44E-15 | chromosome segregation |
| YDR229W | GO:0006473 | 2.09E-13 | protein amino acid acetylation |
| YGL086W | GO:0007059 | 7.44E-15 | chromosome segregation |
| YHR051W | GO:0007028 | 1.26E-08 | cytoplasm organization and biogenesis |
| YKL010C | GO:0044248 | 5.10E-13 | cellular catabolism |
| YKL042W | GO:0007059 | 7.44E-15 | chromosome segregation |
| YNL076W | GO:0006473 | 2.09E-13 | protein amino acid acetylation |
| YOL072W | GO:0006473 | 2.09E-13 | protein amino acid acetylation |
| YOR227W | GO:0007028 | 1.26E-08 | cytoplasm organization and biogenesis |
| YBL022C | GO:0043044 | 8.08E-13 | ATP-dependent chromatin remodeling |
| YBR089C-A | GO:0043044 | 8.84E-13 | ATP-dependent chromatin remodeling |
| YBR109C | GO:0044248 | 3.24E-11 | cellular catabolism |
| YBR131W | GO:0006944 | 0 | membrane fusion |
| YDL035C | GO:0007059 | 0 | chromosome segregation |
| YDL188C | GO:0044248 | 8.57E-13 | cellular catabolism |
| YDR091C | GO:0007028 | 9.16E-09 | cytoplasm organization and biogenesis |
| YEL018W | GO:0050791 | 6.93E-10 | regulation of physiological process |
| YER041W | GO:0007028 | 1.26E-08 | cytoplasm organization and biogenesis |
| YER063W | GO:0007049 | 3.17E-11 | cell cycle |
| YGL019W | GO:0006354 | 1.40E-14 | RNA elongation |
| YGL071W | GO:0043044 | 8.84E-13 | ATP-dependent chromatin remodeling |
| YGL124C | GO:0006944 | 0 | membrane fusion |
| YGL147C | GO:0046903 | 1.44E-15 | secretion |
| YHR024C | GO:0006401 | 3.08E-11 | RNA catabolism |
| YIL066C | GO:0006401 | 3.08E-11 | RNA catabolism |
| YIL084C | GO:0043044 | 4.60E-12 | ATP-dependent chromatin remodeling |
| YLR177W | GO:0050791 | 7.30E-10 | regulation of physiological process |
| YOL041C | GO:0007028 | 1.26E-08 | cytoplasm organization and biogenesis |
| YOR061W | GO:0006354 | 1.40E-14 | RNA elongation |
| YPL113C | GO:0007049 | 3.51E-11 | cell cycle |
| YPL172C | GO:0007049 | 3.17E-11 | cell cycle |
| YPL195W | GO:0006944 | 0 | membrane fusion |
| YPL231W | GO:0050791 | 7.30E-10 | regulation of physiological process |

| | | | |
|---|---|---|---|
| YPL268W | GO:0007059 | 0 | chromosome segregation |
| YBR020W | GO:0051236 | 8.45E-14 | establishment of RNA localization |
| YDR027C | GO:0006944 | 0 | membrane fusion |
| YDR127W | GO:0043285 | 2.30E-09 | biopolymer catabolism |
| YDR143C | GO:0007049 | 3.51E-11 | cell cycle |
| YDR177W | GO:0043285 | 3.00E-09 | biopolymer catabolism |
| YDR226W | GO:0043285 | 3.00E-09 | biopolymer catabolism |
| YFL036W | GO:0051236 | 8.45E-14 | establishment of RNA localization |
| YGR002C | GO:0050791 | 6.93E-10 | regulation of physiological process |
| YIL096C | GO:0007028 | 1.66E-09 | cytoplasm organization and biogenesis |
| YJL008C | GO:0043285 | 2.30E-09 | biopolymer catabolism |
| YJL184W | GO:0043285 | 2.30E-09 | biopolymer catabolism |
| YJR031C | GO:0043044 | 1.28E-14 | ATP-dependent chromatin remodeling |
| YLR074C | GO:0007028 | 5.17E-10 | cytoplasm organization and biogenesis |
| YMR125W | GO:0051236 | 8.45E-14 | establishment of RNA localization |
| YMR228W | GO:0051236 | 8.45E-14 | establishment of RNA localization |
| YNL097C | GO:0051236 | 1.44E-13 | establishment of RNA localization |
| YOL015W | GO:0051236 | 8.45E-14 | establishment of RNA localization |
| YOL020W | GO:0051236 | 1.44E-13 | establishment of RNA localization |
| YBR160W | GO:0043285 | 3.00E-09 | biopolymer catabolism |
| YBR189W | GO:0007028 | 5.17E-10 | cytoplasm organization and biogenesis |
| YBR203W | GO:0046903 | 0 | secretion |
| YBR217W | GO:0043285 | 3.00E-09 | biopolymer catabolism |
| YDR064W | GO:0007028 | 2.36E-09 | cytoplasm organization and biogenesis |
| YDR099W | GO:0043044 | 1.38E-14 | ATP-dependent chromatin remodeling |
| YDR428C | GO:0044248 | 4.13E-12 | cellular catabolism |
| YEL009C | GO:0043044 | 1.38E-14 | ATP-dependent chromatin remodeling |
| YER007W | GO:0044248 | 4.85E-12 | cellular catabolism |
| YFR030W | GO:0043285 | 3.00E-09 | biopolymer catabolism |
| YGL016W | GO:0006352 | 1.53E-12 | transcription initiation |
| YGL057C | GO:0007028 | 2.75E-09 | cytoplasm organization and biogenesis |
| YGR006W | GO:0050791 | 5.11E-10 | regulation of physiological process |
| YGR262C | GO:0043285 | 3.00E-09 | biopolymer catabolism |
| YHR079C | GO:0006473 | 7.01E-14 | protein amino acid acetylation |
| YHR203C | GO:0007028 | 2.36E-09 | cytoplasm organization and biogenesis |
| YJL120W | GO:0044248 | 4.85E-12 | cellular catabolism |
| YJL135W | GO:0007028 | 2.75E-09 | cytoplasm organization and biogenesis |
| YJL174W | GO:0006352 | 1.53E-12 | transcription initiation |
| YJR133W | GO:0044257 | 4.00E-10 | cellular protein catabolism |
| YJR145C | GO:0007028 | 2.36E-09 | cytoplasm organization and biogenesis |
| YKL021C | GO:0044248 | 3.80E-12 | cellular catabolism |
| YKL023W | GO:0006473 | 2.20E-14 | protein amino acid acetylation |
| YKL176C | GO:0007028 | 1.66E-09 | cytoplasm organization and biogenesis |
| YLR125W | GO:0050791 | 4.53E-10 | regulation of physiological process |
| YLR295C | GO:0044257 | 4.00E-10 | cellular protein catabolism |
| YML001W | GO:0006944 | 0 | membrane fusion |
| YNL099C | GO:0050791 | 1.45E-12 | regulation of physiological process |
| YNL213C | GO:0007028 | 3.74E-09 | cytoplasm organization and biogenesis |

| | | | |
|---|---|---|---|
| YNL315C | GO:0050791 | 4.53E-10 | regulation of physiological process |
| YNR049C | GO:0006944 | 0 | membrane fusion |
| YOR252W | GO:0007028 | 4.39E-10 | cytoplasm organization and biogenesis |
| YOR298C-A | GO:0043044 | 1.38E-14 | ATP-dependent chromatin remodeling |
| YPL203W | GO:0050791 | 7.30E-10 | regulation of physiological process |
| YPR152C | GO:0007028 | 4.39E-10 | cytoplasm organization and biogenesis |
| YAL049C | GO:0044248 | 4.26E-12 | cellular catabolism |
| YBL050W | GO:0006944 | 0 | membrane fusion |
| YBR060C | GO:0007049 | 4.67E-11 | cell cycle |
| YBR073W | GO:0007059 | 0 | chromosome segregation |
| YBR269C | GO:0007028 | 2.35E-09 | cytoplasm organization and biogenesis |
| YBR278W | GO:0007028 | 6.26E-10 | cytoplasm organization and biogenesis |
| YDL025C | GO:0044248 | 4.26E-12 | cellular catabolism |
| YDL144C | GO:0044257 | 7.31E-12 | cellular protein catabolism |
| YDL164C | GO:0007028 | 2.35E-09 | cytoplasm organization and biogenesis |
| YDR088C | GO:0044257 | 7.31E-12 | cellular protein catabolism |
| YDR359C | GO:0050791 | 6.93E-10 | regulation of physiological process |
| YGL237C | GO:0007028 | 4.39E-10 | cytoplasm organization and biogenesis |
| YGR275W | GO:0043044 | 0 | ATP-dependent chromatin remodeling |
| YIL069C | GO:0007028 | 1.66E-09 | cytoplasm organization and biogenesis |
| YJR044C | GO:0007028 | 5.65E-10 | cytoplasm organization and biogenesis |
| YLR367W | GO:0007028 | 5.17E-10 | cytoplasm organization and biogenesis |
| YLR441C | GO:0007028 | 1.30E-08 | cytoplasm organization and biogenesis |
| YML032C | GO:0007059 | 0 | chromosome segregation |
| YMR138W | GO:0007028 | 6.93E-10 | cytoplasm organization and biogenesis |
| YNL226W | GO:0007028 | 5.65E-10 | cytoplasm organization and biogenesis |
| YNL261W | GO:0007049 | 4.67E-11 | cell cycle |
| YOR036W | GO:0006944 | 0 | membrane fusion |
| YPR131C | GO:0044248 | 6.32E-13 | cellular catabolism |
| Q0045 | GO:0007059 | 0 | chromosome segregation |
| YAL060W | GO:0007059 | 0 | chromosome segregation |
| YBL087C | GO:0007028 | 5.33E-11 | cytoplasm organization and biogenesis |
| YCR020W-B | GO:0043044 | 1.45E-14 | ATP-dependent chromatin remodeling |
| YDL040C | GO:0044248 | 5.27E-13 | cellular catabolism |
| YDR299W | GO:0007028 | 5.37E-10 | cytoplasm organization and biogenesis |
| YDR356W | GO:0007059 | 0 | chromosome segregation |
| YER081W | GO:0007059 | 0 | chromosome segregation |
| YFR008W | GO:0007059 | 0 | chromosome segregation |
| YFR053C | GO:0007059 | 0 | chromosome segregation |
| YGL023C | GO:0007028 | 2.35E-09 | cytoplasm organization and biogenesis |
| YGL252C | GO:0006473 | 2.20E-14 | protein amino acid acetylation |
| YGR130C | GO:0007059 | 0 | chromosome segregation |
| YKL139W | GO:0051236 | 2.89E-15 | establishment of RNA localization |
| YLR035C | GO:0007028 | 5.40E-10 | cytoplasm organization and biogenesis |
| YLR083C | GO:0007059 | 0 | chromosome segregation |
| YLR395C | GO:0007059 | 0 | chromosome segregation |
| YLR449W | GO:0007028 | 2.35E-09 | cytoplasm organization and biogenesis |
| YML060W | GO:0007028 | 1.21E-10 | cytoplasm organization and biogenesis |

| | | | |
|---|---|---|---|
| YML121W | GO:0007028 | 5.65E-10 | cytoplasm organization and biogenesis |
| YMR123W | GO:0051236 | 2.89E-15 | establishment of RNA localization |
| YMR163C | GO:0007028 | 4.36E-10 | cytoplasm organization and biogenesis |
| YMR242C | GO:0007028 | 4.39E-10 | cytoplasm organization and biogenesis |
| YMR316W | GO:0046903 | 0 | secretion |
| YNL112W | GO:0051236 | 2.89E-15 | establishment of RNA localization |
| YNR004W | GO:0007028 | 4.39E-10 | cytoplasm organization and biogenesis |
| YNR054C | GO:0007028 | 4.39E-10 | cytoplasm organization and biogenesis |
| YOL104C | GO:0007059 | 0 | chromosome segregation |
| YPL157W | GO:0007028 | 4.39E-10 | cytoplasm organization and biogenesis |
| YPL280W | GO:0051179 | 6.55E-10 | localization |
| Q0092 | GO:0007059 | 0 | chromosome segregation |
| YAR035W | GO:0007059 | 0 | chromosome segregation |
| YBR017C | GO:0006997 | 1.11E-16 | nuclear organization and biogenesis |
| YBR111W-A | GO:0006473 | 0 | protein amino acid acetylation |
| YBR181C | GO:0007028 | 1.21E-10 | cytoplasm organization and biogenesis |
| YBR237W | GO:0050791 | 1.08E-11 | regulation of physiological process |
| YBR281C | GO:0007028 | 4.50E-10 | cytoplasm organization and biogenesis |
| YCR060W | GO:0006401 | 7.77E-16 | RNA catabolism |
| YCR099C | GO:0007028 | 5.14E-10 | cytoplasm organization and biogenesis |
| YDL166C | GO:0046903 | 0 | secretion |
| YDL207W | GO:0006997 | 1.11E-16 | nuclear organization and biogenesis |
| YDR079C-A | GO:0007028 | 4.50E-10 | cytoplasm organization and biogenesis |
| YDR083W | GO:0006401 | 7.77E-16 | RNA catabolism |
| YDR179C | GO:0007028 | 9.67E-11 | cytoplasm organization and biogenesis |
| YDR300C | GO:0007028 | 2.98E-10 | cytoplasm organization and biogenesis |
| YDR370C | GO:0050791 | 6.64E-12 | regulation of physiological process |
| YDR376W | GO:0050791 | 9.43E-12 | regulation of physiological process |
| YDR383C | GO:0007059 | 0 | chromosome segregation |
| YDR459C | GO:0007028 | 5.37E-10 | cytoplasm organization and biogenesis |
| YEL032W | GO:0007049 | 4.47E-13 | cell cycle |
| YEL037C | GO:0044248 | 3.93E-12 | cellular catabolism |
| YEL041W | GO:0007028 | 5.33E-11 | cytoplasm organization and biogenesis |
| YEL055C | GO:0007028 | 4.38E-10 | cytoplasm organization and biogenesis |
| YEL077C | GO:0007059 | 0 | chromosome segregation |
| YER064C | GO:0007028 | 5.37E-10 | cytoplasm organization and biogenesis |
| YER087C-A | GO:0007049 | 3.72E-11 | cell cycle |
| YER088C | GO:0007049 | 4.47E-13 | cell cycle |
| YER100W | GO:0007028 | 4.38E-10 | cytoplasm organization and biogenesis |
| YER102W | GO:0007028 | 5.37E-10 | cytoplasm organization and biogenesis |
| YER125W | GO:0046903 | 0 | secretion |
| YFL046W | GO:0006401 | 4.61E-13 | RNA catabolism |
| YFL049W | GO:0050791 | 6.64E-12 | regulation of physiological process |
| YFL067W | GO:0043044 | 0 | ATP-dependent chromatin remodeling |
| YGL038C | GO:0046903 | 0 | secretion |
| YGL093W | GO:0007059 | 0 | chromosome segregation |
| YGL095C | GO:0006944 | 0 | membrane fusion |
| YGL146C | GO:0007028 | 2.98E-10 | cytoplasm organization and biogenesis |

| | | | |
|---|---|---|---|
| YGL184C | GO:0043044 | 1.45E-14 | ATP-dependent chromatin remodeling |
| YGR092W | GO:0050791 | 1.23E-12 | regulation of physiological process |
| YGR194C | GO:0006401 | 1.52E-14 | RNA catabolism |
| YGR263C | GO:0046903 | 0 | secretion |
| YHL039W | GO:0046903 | 0 | secretion |
| YHR034C | GO:0006401 | 7.77E-16 | RNA catabolism |
| YHR052W | GO:0007028 | 8.11E-11 | cytoplasm organization and biogenesis |
| YHR072W-A | GO:0007028 | 2.98E-10 | cytoplasm organization and biogenesis |
| YHR102W | GO:0007059 | 0 | chromosome segregation |
| YHR120W | GO:0006401 | 2.49E-14 | RNA catabolism |
| YHR134W | GO:0007049 | 4.43E-12 | cell cycle |
| YIL111W | GO:0007059 | 0 | chromosome segregation |
| YIR024C | GO:0050791 | 9.43E-12 | regulation of physiological process |
| YJL091C | GO:0007049 | 3.72E-11 | cell cycle |
| YJR055W | GO:0050791 | 8.79E-12 | regulation of physiological process |
| YJR060W | GO:0007059 | 0 | chromosome segregation |
| YKL089W | GO:0007059 | 0 | chromosome segregation |
| YKR039W | GO:0046903 | 2.22E-16 | secretion |
| YKR096W | GO:0007028 | 8.11E-11 | cytoplasm organization and biogenesis |
| YLR052W | GO:0007059 | 0 | chromosome segregation |
| YLR061W | GO:0007028 | 4.50E-10 | cytoplasm organization and biogenesis |
| YLR163C | GO:0006401 | 2.49E-14 | RNA catabolism |
| YLR202C | GO:0007059 | 0 | chromosome segregation |
| YLR288C | GO:0007059 | 0 | chromosome segregation |
| YLR315W | GO:0007059 | 0 | chromosome segregation |
| YLR389C | GO:0050791 | 1.06E-10 | regulation of physiological process |
| YML057W | GO:0044248 | 3.62E-13 | cellular catabolism |
| YML130C | GO:0046903 | 0 | secretion |
| YMR020W | GO:0046903 | 0 | secretion |
| YMR091C | GO:0043044 | 0 | ATP-dependent chromatin remodeling |
| YMR094W | GO:0007059 | 0 | chromosome segregation |
| YMR131C | GO:0006401 | 4.61E-13 | RNA catabolism |
| YMR168C | GO:0007059 | 0 | chromosome segregation |
| YMR223W | GO:0006473 | 0 | protein amino acid acetylation |
| YMR258C | GO:0007028 | 1.01E-10 | cytoplasm organization and biogenesis |
| YMR294W | GO:0006997 | 2.22E-16 | nuclear organization and biogenesis |
| YMR307W | GO:0046903 | 0 | secretion |
| YMR317W | GO:0007028 | 4.39E-10 | cytoplasm organization and biogenesis |
| YNL066W | GO:0046903 | 0 | secretion |
| YNL124W | GO:0007028 | 2.98E-10 | cytoplasm organization and biogenesis |
| YNL171C | GO:0046903 | 0 | secretion |
| YNL191W | GO:0007028 | 4.50E-10 | cytoplasm organization and biogenesis |
| YNL230C | GO:0007028 | 4.36E-10 | cytoplasm organization and biogenesis |
| YNL265C | GO:0006401 | 1.52E-14 | RNA catabolism |
| YNL284C | GO:0046903 | 0 | secretion |
| YOR001W | GO:0006401 | 0 | RNA catabolism |
| YOR026W | GO:0007059 | 0 | chromosome segregation |
| YOR080W | GO:0007028 | 2.98E-10 | cytoplasm organization and biogenesis |

| | | | |
|---|---|---|---|
| YOR269W | GO:0007059 | 0 | chromosome segregation |
| YOR312C | GO:0007028 | 8.11E-11 | cytoplasm organization and biogenesis |
| YPL047W | GO:0006473 | 0 | protein amino acid acetylation |
| YPL051W | GO:0046903 | 0 | secretion |
| YPL081W | GO:0007028 | 5.37E-10 | cytoplasm organization and biogenesis |
| YPL085W | GO:0046903 | 0 | secretion |
| YPL125W | GO:0006997 | 1.11E-16 | nuclear organization and biogenesis |
| YPL212C | GO:0007028 | 5.37E-10 | cytoplasm organization and biogenesis |
| YPL244C | GO:0046903 | 0 | secretion |
| YPR083W | GO:0006997 | 2.22E-16 | nuclear organization and biogenesis |
| YPR189W | GO:0006401 | 0 | RNA catabolism |

## Supplementary Table 10. Novel GO functional predictions for fly proteins.

| protein | GO prediction | p-value | GO annotation |
|---|---|---|---|
| CG6625 | GO:0051179 | 8.07E-07 | localization |
| CG6322 | GO:0006350 | 1.41E-06 | transcription |
| CG11489 | GO:0043283 | 1.53E-06 | biopolymer metabolism |
| CG33101 | GO:0043283 | 1.53E-06 | biopolymer metabolism |
| CG13281 | GO:0006139 | 2.83E-07 | nucleobase, nucleoside, nucleotide and nucleic acid metabolism |
| CG8770 | GO:0006139 | 2.83E-07 | nucleobase, nucleoside, nucleotide and nucleic acid metabolism |
| CG1472 | GO:0007049 | 3.39E-11 | cell cycle |
| CG10654 | GO:0050791 | 5.27E-10 | regulation of physiological process |
| CG11799 | GO:0050791 | 5.27E-10 | regulation of physiological process |
| CG15224 | GO:0050791 | 5.27E-10 | regulation of physiological process |
| CG2184 | GO:0050791 | 5.27E-10 | regulation of physiological process |
| CG5395 | GO:0050791 | 5.27E-10 | regulation of physiological process |
| CG6226 | GO:0050791 | 5.27E-10 | regulation of physiological process |
| CG7352 | GO:0050791 | 5.27E-10 | regulation of physiological process |
| CG7626 | GO:0050791 | 5.27E-10 | regulation of physiological process |
| CG1099 | GO:0016070 | 1.29E-07 | RNA metabolism |
| CG11678 | GO:0016070 | 4.69E-10 | RNA metabolism |
| CG1489 | GO:0016070 | 4.69E-10 | RNA metabolism |
| CG17596 | GO:0016070 | 4.44E-08 | RNA metabolism |
| CG7235 | GO:0016070 | 1.81E-07 | RNA metabolism |
| CG3427 | GO:0019538 | 1.37E-06 | protein metabolism |
| CG9916 | GO:0019538 | 1.37E-06 | protein metabolism |
| CG4701 | GO:0019538 | 1.37E-06 | protein metabolism |
| CG5094 | GO:0019538 | 1.37E-06 | protein metabolism |
| CG5798 | GO:0051301 | 0 | cell division |
| CG7717 | GO:0019538 | 1.37E-06 | protein metabolism |
| CG13597 | GO:0016043 | 4.08E-08 | cell organization and biogenesis |
| CG7605 | GO:0016043 | 4.08E-08 | cell organization and biogenesis |
| CG8815 | GO:0016043 | 4.08E-08 | cell organization and biogenesis |
| CG9099 | GO:0016043 | 4.08E-08 | cell organization and biogenesis |
| CG12010 | GO:0009056 | 2.01E-10 | catabolism |
| CG13164 | GO:0050896 | 4.83E-11 | response to stimulus |
| CG5289 | GO:0009056 | 2.01E-10 | catabolism |
| CG8103 | GO:0050896 | 4.83E-11 | response to stimulus |

| | | | |
|---|---|---|---|
| CG12051 | GO:0006139 | 6.81E-07 | nucleobase, nucleoside, nucleotide and nucleic acid metabolism |
| CG8882 | GO:0006139 | 6.81E-07 | nucleobase, nucleoside, nucleotide and nucleic acid metabolism |
| CG9423 | GO:0006139 | 6.81E-07 | nucleobase, nucleoside, nucleotide and nucleic acid metabolism |
| CG10033 | GO:0051301 | 0 | cell division |
| CG12047 | GO:0006139 | 1.76E-06 | nucleobase, nucleoside, nucleotide and nucleic acid metabolism |
| CG5072 | GO:0051301 | 0 | cell division |
| CG7111 | GO:0051301 | 0 | cell division |
| CG7765 | GO:0006139 | 1.76E-06 | nucleobase, nucleoside, nucleotide and nucleic acid metabolism |
| CG8203 | GO:0051301 | 0 | cell division |
| CG9096 | GO:0051301 | 0 | cell division |
| CG2843 | GO:0016070 | 2.46E-11 | RNA metabolism |
| CG11228 | GO:0051301 | 0 | cell division |
| CG3510 | GO:0051301 | 0 | cell division |
| CG3668 | GO:0051301 | 0 | cell division |
| CG5363 | GO:0051301 | 0 | cell division |
| CG3071 | GO:0051301 | 0 | cell division |
| CG32156 | GO:0051301 | 0 | cell division |
| CG10023 | GO:0051301 | 0 | cell division |
| CG10229 | GO:0043283 | 1.43E-07 | biopolymer metabolism |
| CG10261 | GO:0051301 | 1.34E-13 | cell division |
| CG10295 | GO:0051301 | 0 | cell division |
| CG10327 | GO:0009056 | 3.61E-11 | catabolism |
| CG10484 | GO:0009056 | 7.08E-09 | catabolism |
| CG10498 | GO:0007049 | 1.75E-13 | cell cycle |
| CG10578 | GO:0050896 | 8.79E-12 | response to stimulus |
| CG1057 | GO:0051301 | 0 | cell division |
| CG10673 | GO:0009056 | 1.54E-07 | catabolism |
| CG10882 | GO:0046903 | 0 | secretion |
| CG1088 | GO:0051301 | 0 | cell division |
| CG11495 | GO:0009056 | 1.54E-07 | catabolism |
| CG11526 | GO:0050896 | 8.79E-12 | response to stimulus |
| CG1171 | GO:0051301 | 0 | cell division |

| | | | |
|---|---|---|---|
| 1 | | | |
| CG12020 | GO:0007049 | 1.75E-13 | cell cycle |
| CG12235 | GO:0051301 | 0 | cell division |
| CG12244 | GO:0051301 | 0 | cell division |
| CG12306 | GO:0007049 | 9.39E-13 | cell cycle |
| CG12799 | GO:0051301 | 0 | cell division |
| CG13956 | GO:0043283 | 1.43E-07 | biopolymer metabolism |
| CG1403 | GO:0051301 | 0 | cell division |
| CG1461 | GO:0051301 | 0 | cell division |
| CG1467 | GO:0046903 | 0 | secretion |
| CG1490 | GO:0007049 | 3.37E-11 | cell cycle |
| CG14941 | GO:0051301 | 0 | cell division |
| CG14999 | GO:0043283 | 1.43E-07 | biopolymer metabolism |
| CG15437 | GO:0007049 | 8.98E-12 | cell cycle |
| CG16954 | GO:0007049 | 3.39E-11 | cell cycle |
| CG16983 | GO:0007049 | 0 | cell cycle |
| CG16988 | GO:0051301 | 0 | cell division |
| CG17161 | GO:0051301 | 0 | cell division |
| CG17342 | GO:0051301 | 0 | cell division |
| CG17520 | GO:0007049 | 1.75E-13 | cell cycle |
| CG17903 | GO:0046903 | 0 | secretion |
| CG18069 | GO:0050896 | 8.79E-12 | response to stimulus |
| CG18255 | GO:0009056 | 3.19E-08 | catabolism |
| CG1848 | GO:0051301 | 0 | cell division |
| CG1851 | GO:0051301 | 0 | cell division |
| CG18582 | GO:0009056 | 1.36E-09 | catabolism |
| CG1925 | GO:0007049 | 1.60E-13 | cell cycle |
| CG1954 | GO:0007049 | 1.75E-13 | cell cycle |
| CG2146 | GO:0051301 | 0 | cell division |
| CG2174 | GO:0051301 | 0 | cell division |
| CG2615 | GO:0051301 | 0 | cell division |
| CG2830 | GO:0051301 | 0 | cell division |
| CG3001 | GO:0009056 | 3.61E-11 | catabolism |

| | | | |
|---|---|---|---|
| 9 | | | |
| CG30084 | GO:0051301 | 0 | cell division |
| CG30115 | GO:0051301 | 0 | cell division |
| CG30132 | GO:0051301 | 0 | cell division |
| CG31135 | GO:0051301 | 0 | cell division |
| CG31175 | GO:0007049 | 2.95E-10 | cell cycle |
| CG3129 | GO:0051301 | 0 | cell division |
| CG31762 | GO:0019538 | 1.37E-06 | protein metabolism |
| CG31811 | GO:0051301 | 0 | cell division |
| CG31958 | GO:0051301 | 0 | cell division |
| CG32019 | GO:0009056 | 1.36E-09 | catabolism |
| CG3201 | GO:0051301 | 0 | cell division |
| CG32555 | GO:0051301 | 0 | cell division |
| CG32772 | GO:0007049 | 0 | cell cycle |
| CG33167 | GO:0007049 | 2.05E-13 | cell cycle |
| CG3320 | GO:0051301 | 0 | cell division |
| CG3403 | GO:0007049 | 0 | cell cycle |
| CG3412 | GO:0007049 | 8.98E-12 | cell cycle |
| CG3564 | GO:0046903 | 0 | secretion |
| CG3851 | GO:0007049 | 0 | cell cycle |
| CG4147 | GO:0009056 | 5.21E-07 | catabolism |
| CG4152 | GO:0007049 | 2.95E-10 | cell cycle |
| CG4173 | GO:0051301 | 0 | cell division |
| CG4523 | GO:0019538 | 1.37E-06 | protein metabolism |
| CG4719 | GO:0051301 | 0 | cell division |
| CG4799 | GO:0051301 | 0 | cell division |
| CG5105 | GO:0007049 | 1.75E-13 | cell cycle |
| CG5313 | GO:0043283 | 1.43E-07 | biopolymer metabolism |
| CG5330 | GO:0051301 | 0 | cell division |
| CG5408 | GO:0051301 | 0 | cell division |
| CG5486 | GO:0043283 | 1.43E-07 | biopolymer metabolism |
| CG5701 | GO:0051301 | 0 | cell division |
| CG5814 | GO:0007049 | 1.60E-13 | cell cycle |
| CG6258 | GO:0050896 | 8.79E-12 | response to stimulus |
| CG6703 | GO:0051301 | 0 | cell division |
| CG6899 | GO:0009056 | 5.21E-07 | catabolism |
| CG6944 | GO:0007049 | 2.95E-10 | cell cycle |
| CG6950 | GO:0007049 | 3.51E-11 | cell cycle |
| CG7001 | GO:0051301 | 0 | cell division |

| | | | |
|---|---|---|---|
| CG7073 | GO:0046903 | 0 | secretion |
| CG7375 | GO:0051301 | 0 | cell division |
| CG7425 | GO:0007049 | 1.75E-13 | cell cycle |
| CG7456 | GO:0046903 | 0 | secretion |
| CG7524 | GO:0051301 | 0 | cell division |
| CG7838 | GO:0007049 | 1.60E-13 | cell cycle |
| CG7865 | GO:0009056 | 3.61E-11 | catabolism |
| CG7940 | GO:0009056 | 7.08E-09 | catabolism |
| CG8173 | GO:0051301 | 0 | cell division |
| CG8280 | GO:0051301 | 0 | cell division |
| CG8287 | GO:0007049 | 1.60E-13 | cell cycle |
| CG8542 | GO:0051301 | 0 | cell division |
| CG8548 | GO:0051301 | 1.34E-13 | cell division |
| CG8598 | GO:0051301 | 0 | cell division |
| CG8606 | GO:0050896 | 8.79E-12 | response to stimulus |
| CG8637 | GO:0007049 | 1.60E-13 | cell cycle |
| CG8863 | GO:0051301 | 0 | cell division |
| CG8866 | GO:0051301 | 0 | cell division |
| CG9075 | GO:0051301 | 0 | cell division |
| CG9699 | GO:0006793 | 4.78E-12 | phosphorus metabolism |
| CG9769 | GO:0043283 | 1.43E-07 | biopolymer metabolism |
| CG8417 | GO:0016070 | 2.71E-09 | RNA metabolism |
| CG2903 | GO:0043283 | 5.35E-07 | biopolymer metabolism |
| CG2960 | GO:0043283 | 5.35E-07 | biopolymer metabolism |
| CG3949 | GO:0016070 | 4.69E-10 | RNA metabolism |
| CG4379 | GO:0016070 | 4.69E-10 | RNA metabolism |
| CG4832 | GO:0016070 | 4.69E-10 | RNA metabolism |
| CG6521 | GO:0043283 | 5.35E-07 | biopolymer metabolism |
| CG31033 | GO:0016070 | 1.28E-09 | RNA metabolism |
| CG2168 | GO:0016070 | 3.42E-08 | RNA metabolism |
| CG7826 | GO:0016070 | 3.42E-08 | RNA metabolism |
| CG12101 | GO:0016070 | 5.15E-09 | RNA metabolism |
| CG1523 | GO:0016070 | 3.42E-08 | RNA metabolism |
| CG3314 | GO:0016070 | 3.42E-08 | RNA metabolism |
| CG4157 | GO:0016070 | 4.78E-11 | RNA metabolism |
| CG5648 | GO:0016070 | 4.78E-11 | RNA metabolism |
| CG8264 | GO:0016070 | 2.47E-09 | RNA metabolism |
| CG1836 | GO:0009056 | 3.61E-11 | catabolism |
| CG6033 | GO:0016070 | 9.00E-09 | RNA metabolism |
| CG7762 | GO:0009056 | 3.61E-11 | catabolism |
| CG14884 | GO:0009056 | 2.01E-10 | catabolism |
| CG15010 | GO:0007049 | 0 | cell cycle |
| CG17256 | GO:0016070 | 2.47E-09 | RNA metabolism |
| CG1874 | GO:0050896 | 1.42E-12 | response to stimulus |

| | | | |
|---|---|---|---|
| 3 | | | |
| CG2863 | GO:0050896 | 1.07E-11 | response to stimulus |
| CG31359 | GO:0050896 | 1.42E-12 | response to stimulus |
| CG31449 | GO:0050896 | 1.42E-12 | response to stimulus |
| CG4006 | GO:0050896 | 1.42E-12 | response to stimulus |
| CG4488 | GO:0050896 | 1.42E-12 | response to stimulus |
| CG5680 | GO:0050896 | 1.42E-12 | response to stimulus |
| CG5834 | GO:0050896 | 1.42E-12 | response to stimulus |
| CG10703 | GO:0016070 | 3.18E-10 | RNA metabolism |
| CG12559 | GO:0016070 | 3.34E-10 | RNA metabolism |
| CG15440 | GO:0016070 | 2.65E-09 | RNA metabolism |
| CG17117 | GO:0016070 | 2.65E-09 | RNA metabolism |
| CG9927 | GO:0016070 | 3.18E-10 | RNA metabolism |
| CG10149 | GO:0009056 | 3.61E-11 | catabolism |
| CG10181 | GO:0043283 | 1.62E-08 | biopolymer metabolism |
| CG10206 | GO:0016070 | 1.09E-10 | RNA metabolism |
| CG10478 | GO:0016070 | 1.09E-10 | RNA metabolism |
| CG11092 | GO:0043283 | 1.62E-08 | biopolymer metabolism |
| CG11659 | GO:0016070 | 3.34E-10 | RNA metabolism |
| CG11870 | GO:0016070 | 3.34E-13 | RNA metabolism |
| CG12085 | GO:0016070 | 9.14E-11 | RNA metabolism |
| CG13032 | GO:0043283 | 1.62E-08 | biopolymer metabolism |
| CG13625 | GO:0016070 | 9.43E-09 | RNA metabolism |
| CG14641 | GO:0016070 | 3.34E-10 | RNA metabolism |
| CG15481 | GO:0016070 | 1.09E-10 | RNA metabolism |
| CG15855 | GO:0016070 | 1.09E-10 | RNA metabolism |
| CG16792 | GO:0016070 | 7.77E-16 | RNA metabolism |
| CG17348 | GO:0006139 | 7.82E-08 | nucleobase, nucleoside, nucleotide and nucleic acid metabolism |
| CG2238 | GO:0016070 | 7.77E-16 | RNA metabolism |
| CG2261 | GO:0016070 | 9.14E-11 | RNA metabolism |
| CG2677 | GO:0016070 | 1.09E-10 | RNA metabolism |
| CG2925 | GO:0016070 | 5.59E-12 | RNA metabolism |

| | | | |
|---|---|---|---|
| CG31291 | GO:0016070 | 5.59E-12 | RNA metabolism |
| CG3151 | GO:0016070 | 3.35E-13 | RNA metabolism |
| CG31638 | GO:0043283 | 1.62E-08 | biopolymer metabolism |
| CG3193 | GO:0016070 | 5.59E-12 | RNA metabolism |
| CG31990 | GO:0016070 | 7.77E-16 | RNA metabolism |
| CG33070 | GO:0016070 | 7.77E-16 | RNA metabolism |
| CG3612 | GO:0043283 | 1.62E-08 | biopolymer metabolism |
| CG3780 | GO:0016070 | 3.18E-10 | RNA metabolism |
| CG4274 | GO:0016070 | 3.34E-13 | RNA metabolism |
| CG5258 | GO:0016070 | 3.35E-13 | RNA metabolism |
| CG5352 | GO:0016070 | 3.34E-13 | RNA metabolism |
| CG5519 | GO:0016070 | 5.59E-12 | RNA metabolism |
| CG5939 | GO:0043283 | 1.62E-08 | biopolymer metabolism |
| CG6773 | GO:0016070 | 1.09E-10 | RNA metabolism |
| CG6932 | GO:0009056 | 3.61E-11 | catabolism |
| CG7238 | GO:0016070 | 4.69E-10 | RNA metabolism |
| CG8219 | GO:0006139 | 3.94E-08 | nucleobase, nucleoside, nucleotide and nucleic acid metabolism |
| CG8384 | GO:0016070 | 5.59E-12 | RNA metabolism |
| CG8427 | GO:0016070 | 7.77E-16 | RNA metabolism |
| CG8435 | GO:0016070 | 9.14E-11 | RNA metabolism |
| CG8725 | GO:0009056 | 3.61E-11 | catabolism |
| CG9045 | GO:0016070 | 3.34E-10 | RNA metabolism |
| CG9446 | GO:0016070 | 1.34E-09 | RNA metabolism |

## Supplementary Table 11. Publications documented in the HTP-GI dataset.

| PubMed ID | Article Title | Author | Year |
|---|---|---|---|
| 11743205 | Systematic genetic analysis with ordered arrays of yeast deletion mutants. | Tong AH et al | 2001 |
| 12077337 | Dissection of a complex phenotype by functional genomics reveals roles for the yeast cyclin-dependent protein kinase Pho85 in stress adaptation and cell integrity. | Huang D et al | 2002 |
| 12089449 | Systematic identification of pathways that couple cell growth and division in yeast. | Jorgensen P et al | 2002 |
| 12454058 | High-resolution genetic mapping with ordered arrays of Saccharomyces cerevisiae deletion mutants. | Jorgensen P et al | 2002 |
| 12686605 | Synthetic lethal analysis implicates Ste20p, a p21-activated potein kinase, in polarisome activation. | Goehring AS et al | 2003 |
| 12773564 | Methylation of histone H3 by Set2 in Saccharomyces cerevisiae is linked to transcriptional elongation by RNA polymerase II. | Krogan NJ et al | 2003 |
| 12857883 | Regulation of the yeast amphiphysin homologue Rvs167p by phosphorylation. | Friesen H et al | 2003 |
| 12912927 | Elg1 forms an alternative RFC complex important for DNA replication and genome integrity. | Bellaoui M et al | 2003 |
| 12949174 | A synthetic analysis of the Saccharomyces cerevisiae stress sensor Mid2p, and identification of a Mid2p-interacting protein, Zeo1p, that modulates the PKC1-MPK1 cell integrity pathway. | Green R et al | 2003 |
| 12960420 | Interaction between a Ras and a Rho GTPase couples selection of a growth site to the development of cell polarity in yeast. | Kozminski KG et al | 2003 |
| 14690608 | A Snf2 family ATPase complex required for recruitment of the histone H2A variant Htz1. | Krogan NJ et al | 2003 |
| 14661025 | Integration of chemical-genetic and genetic interaction data links bioactive compounds to cellular target pathways. | Parsons AB et al | 2004 |
| 14742714 | Identification of protein complexes required for efficient sister chromatid cohesion. | Mayer ML et al | 2004 |
| 14764870 | Global mapping of the yeast genetic interaction network. | Tong AH et al | 2004 |
| 15166135 | Analysis of beta-1,3-glucan assembly in Saccharomyces cerevisiae using a synthetic interaction network and altered sensitivity to caspofungin. | Lesage G et al | 2004 |

| | | | |
|---|---|---|---|
| 15238513 | The origin recognition complex links replication, sister chromatid cohesion and transcriptional silencing in Saccharomyces cerevisiae. | Suter B et al | 2004 |
| 15242642 | Exploration of essential gene functions via titratable promoter alleles. | Mnaimneh S et al | 2004 |
| 15282308 | Mrc1 is required for sister chromatid cohesion to aid in recombination repair of spontaneous damage. | Xu H et al | 2004 |
| 15372071 | Genome-wide lethality screen identifies new PI4,5P(2) effectors that regulatethe actin cytoskeleton. | Audhya A et al | 2004 |
| 15504911 | The GTPase Arf1p and the ER to Golgi cargo receptor Erv14p cooperate to recruitthe golgin Rud3p to the cis-Golgi. | Gillingham AK et al | 2004 |
| 15525520 | A robust toolkit for functional profiling of the yeast genome. | Pan X et al | 2004 |
| 15579722 | Uncovering novel cell cycle players through the inactivation of securin in budding yeast. | Sarin S et al | 2004 |
| 15353583 | Regulation of chromosome stability by the histone H2A variant Htz1, the Swr1 chromatin remodeling complex, and the histone acetyltransferase NuA4. | Krogan NJ et al | 2004 |
| 15563457 | Interaction of Fcp1 phosphatase with elongating RNA polymerase II holoenzyme, enzymatic mechanism of action, and genetic interaction with elongator. | Kong SE et al | 2004 |
| 15249665 | Bipolar orientation of chromosomes in Saccharomyces cerevisiae is monitored by Mad1 and Mad2, but not by Mad3. | Lee MS et al | 2004 |
| 15082542 | Genetic interactions of DST1 in Saccharomyces cerevisiae suggest a role of TFIIS in the initiation-elongation transition. | Malagon F et al | 2004 |
| 15082776 | The GTPase-activating enzyme Gyp1p is required for recycling of internalized membrane material by inactivation of the Rab/Ypt GTPase Ypt1p. | Lafourcade C et al | 2004 |
| 15579722 | Uncovering novel cell cycle players through the inactivation of securin in budding yeast. | Sarin S et al | 2004 |
| 15632065 | Histone H2B ubiquitylation is associated with elongating RNA polymerase II. | Xiao T et al | 2005 |
| 15766533 | Navigating the chaperone network: an integrative map of physical and genetic interactions mediated by the hsp90 chaperone. | Zhao R et al | 2005 |
| 16157669 | Diverse Functions of Spindle Assembly Checkpoint Genes in Saccharomyces cerevisiae. | Daniel JA et al | 2005 |

| | | | |
|---|---|---|---|
| 15725626 | Genetic network interactions among replication, repair and nuclear pore deficiencies in yeast. | Loeillet S et al | 2005 |
| 16040246 | BUR kinase selectively regulates H3 K4 trimethylation and H2B ubiquitylation through recruitment of the PAF elongation complex. | Laribee RN et al | 2005 |
| 15817685 | Reverse recruitment: the Nup84 nuclear pore subcomplex mediates Rap1/Gcr1/Gcr2 transcriptional activation. | Menon BB et al | 2005 |
| 16024805 | Disruption of mechanisms that prevent rereplication triggers a DNA damage response. | Archambault V et al | 2005 |
| 15998715 | Genome-wide synthetic lethal screens identify an interaction between the nuclear envelope protein, Apq12p, and the kinetochore in Saccharomyces cerevisiae. | Montpetit B et al | 2005 |
| 16024810 | Components of the ESCRT pathway, DFG16, and YGR122w are required for Rim101 to act as a corepressor with Nrg1 at the negative regulatory element of the DIT1 gene of Saccharomyces cerevisiae. | Rothfels K et al | 2005 |
| 15715908 | An interactional network of genes involved in chitin synthesis in Saccharomyces cerevisiae. | Lesage G et al | 2005 |
| 15657441 | H2B ubiquitin protease Ubp8 and Sgf11 constitute a discrete functional module within the Saccharomyces cerevisiae SAGA complex. | Ingvarsdottir K et al | 2005 |
| 15574876 | Synthetic genetic array analysis of the PtdIns 4-kinase Pik1p identifies components in a Golgi-specific Ypt31/rab-GTPase signaling pathway. | Sciorra VA et al | 2005 |
