# Supplementary material for: Comprehensive curation and analysis of global interaction networks in Saccharomyces cerevisiae
Source: J Biol. 2006 Jun 8;5(4):11. doi: 10.1186/jbiol36 (PMC1561585; doi:10.1186/jbiol36)
Supplement: Additional data file 2 — A comparison of the LC dataset with other curated datasets and details of functional predictions [file jbiol36-s2.pdf]

SUPPLEMENTARY INFORMATION FOR REGULY AND BREITKREUTZ *ET AL.*

1. Comparison to other curated datasets

The MIPS database is a significant repository of literature-derived *S. cerevisiae* interactions that has been assiduously curated since its inception [37]. To validate our curation approach, we analyzed the overlap between the 30,978 non-HTP interactions in the February 1, 2005 version of the LC dataset and the existing set of 4,468 non-HTP interactions in the April 27, 2004 release of the MIPS database (see http://mips.gsf.de/genre/proj/yeast/). The 929 shared publications (11 publications present in MIPS were missing from the LC dataset) contained a total of 5,711 reported interactions, of which only 2,116, or ~40%, were found in both datasets. The LC dataset contained 1,965 interactions that were not documented in MIPS, whereas 1,630 interactions were documented in MIPS but not the LC dataset (Supplementary Figure 1A). The reason for this discrepancy was investigated in detail. 487 interactions reported by MIPS were missed in the LC curation effort, while 481 of the MIPS-specific interactions were not supported by sufficient evidence to warrant inclusion in the LC dataset (e.g., interactions reported as data not shown or cited to reviews). An additional 111 interactions were over-represented in MIPS because of matrix or partial matrix representations (in contrast to the minimal spoke representation used for the LC dataset). Finally, electronic text was not available for 376 of the interactions reported by MIPS and 175 interactions were from 11 papers in MIPS that were not traceable because identifiers did not link to any known publication.

By extrapolation from this comparison to MIPS, we estimate that the LC dataset might lack 17% of bona fide interactions supported by all literature (863 (=487 +376) of

4,944 (=5,711-481-111-175), due to both incomplete curation and incomplete publication coverage. This value is likely to be an overestimate since the number of older publications for which e-text is unavailable is undoubtedly less than the corresponding proportion of interactions in the MIPS dataset. Thus, for shared available electronic publications with MIPS, the false negative rate in the LC dataset was 10% (487 of 4743 (=5711-481-376-111)), whereas the corresponding rate for MIPS was 44% (1965 of 4743).

We carried out a similar analysis for literature curated *S. cerevisiae* interactions extracted from the BIND database [41]. As of April 1, 2005 we were able to manually extract 263 curated non-HTP papers from BIND (see http://bind.ca/), corresponding to a total of 2,258 interactions from both LC and BIND datasets. For this set of 263 publications, 982 interactions were shared and the LC dataset contained 1,059 interactions not found in BIND, while BIND contained 98 interactions not found in LC-PI (Supplementary Figure 1B). Additional missing interactions in the LC dataset included 51 interactions from 4 papers present in BIND but not in the LC dataset, 18 interactions for which e-text was unavailable, and 50 interactions which were not supported by sufficient evidence to warrant inclusion in the LC dataset. Thus, in this comparison of 263 shared publications, the LC dataset had a false negative rate of 5% (98 of 2,139), whereas the corresponding rate for BIND was 50% (1,059 of 2,139).

A third major repository for protein interactions is the DIP database [40]. As of July 31, 2005, DIP contained 1,267 curated non-HTP papers (see http://dip.doe-mbi.ucla.edu) corresponding to a total of 7,546 interactions from both LC and DIP datasets. For this set of 1,267 publications, 3,455 interactions were shared between

both dataset, while the LC dataset contained 2,494 interactions not found in DIP and lacked 886 interactions not that were in DIP (Supplementary Figure 1C). Additional missing interactions in the LC dataset included 193 interactions for which e-text was unavailable and 518 interactions which were not supported by sufficient evidence to warrant inclusion in the LC dataset. Thus, in this comparison of shared publications, the LC dataset had a false negative error rate of 20% (886 of 6,845), whereas the corresponding rate for DIP was 36% (2,494 of 6,845). All told, the curation discrepancies between different databases highlights the inherent difficulties in manual curation of the primary literature and emphasizes the need for parallel curation efforts.

To address the issue of interaction reliability as supported by contextual information, we carefully re-curated a specific set of LC-PI interactions that were nucleated by a high confidence dataset derived from the overlap of HTP studies and MIPS, called the Filtered Yeast Interactome (FYI), which contains 2,493 interactions between 1,231 nodes [54]. A common set of 1,201 nodes retrieves 4,111 interactions from the LC-PI dataset, a subset we termed the LC-FYI dataset. Of the interactions in LC-FYI, 1,724 were validated by more than one publication or experimental method and were considered reliable. For the remaining 2,387 singly validated interactions, each publication was read in detail to assess data quality and supporting contextual information. Of this set, 1,769 were supported by well-controlled experiments and strong contextual evidence; 433 were classified as less reliable, mainly because they derived from publications that reported HTP-like datasets; and 22 could not be assessed because electronic text could not be re-accessed. 163 interactions were outright errors: 79 were curator reading errors; 61 were curator typographical errors; 23

were supported by weak indirect evidence that should not have been admitted (Figure 1D). This careful re-curation thus revealed an error rate of 4.0 % (163 of 4,111), making the reasonable assumption that all multiply validated interactions are correct.

Finally, we also compared the LC dataset to a facile automated method for data extraction. The pre-BIND database uses literature co-occurrence of gene names in abstracts and other qualifiers to predict publications that may contain interaction data [98]. When compared to manual curation, this basic text mining approach has expectedly high error rates. Thus, only 8% of the predicted interactions correctly corresponded to the publication source, while 33% of predicted interactions correspond to an interaction in the LC dataset (i.e., an overlap that maps to a different publication) and 67% of the predicted interactions are not actually documented in the literature (data not shown). The high error rates of automated methods is not surprising given that many interactions are described only in figure legends and tables. This comparison underscores the need for author-directed deposition of interaction data in conjunction with publication.

2. Details of functional predictions

Affinity precipitation, synthetic lethality, and yeast 2-hybrid data were obtained by merging data from the BioGRID and BIND databases [41,43,53]. Microarray datasets were collected from the Stanford Microarray Database (http://genome-www5.stanford.edu/). Our collection includes 11 different studies, totaling 30 distinct biological conditions [99-108]. Pearson correlation coefficients for each gene-pair were computed on each set of biological conditions separately and converted to standard normal z-scores. For example, for gene pair *i-j* in condition set *k,*

$$z_{ij_k} = \frac{\rho_{ij_k} - \overline{\rho}_k}{\sigma_{\rho_k}}$$

where ρ_{ij_k} is the Pearson correlation coefficient computed over the *k*th set of conditions, $\overline{\rho}_k$ is the average correlation over all pairs for those conditions, and σ_{ρ_k} is the standard deviation over all pairs for those conditions. The final combined score for each gene pair was then computed by summing the z-scores for all sets of conditions.

Constructing precision-recall curves. Precision and recall for each genomic data type were calculated as follows:

true positives (TP): gene pairs associated by data and annotated as positives in GO standard

false positives (FP): gene pairs associated by data and annotated as negatives in GO standard

$$\text{Precision} = \frac{\text{TP}}{\text{TP} + \text{FP}}$$

$$\text{Recall} = \frac{\text{TP}}{\text{total \# of GO standard positive pairs}}$$

For interaction data (e.g. curated literature pairs, synthetic lethality), precision and recall are computed at a single point by counting the number of true positives (TP) and false positives (FP) in the protein pairs associated by the data. For continuous-valued data (e.g. microarray correlation), the precision and recall calculations above are computed for a range of thresholds. Each threshold yields one point on the precision-recall curve by considering protein pairs whose correlation exceeds the threshold value as positive predictions and other pairs as negative.

F-score analysis. We used F-score analysis on the precision-recall results to analyze

the functional diversity in prediction performance for the different data types as compared to our literature curation. An F-score is used to obtain a combined measure of precision-recall performance and can be computed at any point along a precision-recall curve as follows:

$$\text{F-score} = \frac{(\beta^2 + 1) * \text{Precision} * \text{Recall}}{\beta^2 * \text{Precision} + \text{Recall}}$$

β can be adjusted to change the relative importance of precision and recall (we use $\beta = 1$ for all calculations here). We computed the maximum F-score achieved for each of the 146 GO terms we tested. Functional diversity was measured by varying an F-score threshold and counting the number of GO terms for which the predictive performance of each data type exceeded that threshold.

REFERENCES

37. Mewes HW, Frishman D, Guldener U, Mannhaupt G, Mayer K, Mokrejs M, Morgenstern B, Munsterkotter M, Rudd S, Weil B: **MIPS: a database for genomes and protein sequences**. *Nucleic Acids Res* 2002, **30**(1):31-34.

40. Xenarios I, Salwinski L, Duan XJ, Higney P, Kim SM, Eisenberg D: **DIP, the Database of Interacting Proteins: a research tool for studying cellular networks of protein interactions**. *Nucleic Acids Res* 2002, **30**(1):303-305.

41. Bader GD, Betel D, Hogue CW: **BIND: the Biomolecular Interaction Network Database**. *Nucleic Acids Res* 2003, **31**(1):248-250.

43. Stark C, Breitkreutz BJ, Reguly T, Boucher L, Breitkreutz A, Tyers M: **BioGRID: a general repository for interaction datasets**. *Nucleic Acids Res* 2006, **34**(Database issue):D535-539.

53. Breitkreutz BJ, Stark C, Tyers M: **The GRID: the General Repository for Interaction Datasets**. *Genome Biol* 2003, **4**(3):R23.

54. Han JD, Bertin N, Hao T, Goldberg DS, Berriz GF, Zhang LV, Dupuy D, Walhout AJ, Cusick ME, Roth FP *et al*: **Evidence for dynamically organized modularity in the yeast protein-protein interaction network**. *Nature* 2004, **430**(6995):88-93.

67. Ghaemmaghami S, Huh WK, Bower K, Howson RW, Belle A, Dephoure N, O'Shea EK, Weissman JS: **Global analysis of protein expression in yeast**. *Nature* 2003, **425**(6959):737-741.

71. Hughes TR, Marton MJ, Jones AR, Roberts CJ, Stoughton R, Armour CD, Bennett HA, Coffey E, Dai H, He YD *et al*: **Functional discovery via a compendium of expression profiles**. *Cell* 2000, **102**(1):109-126.

98. Donaldson I, Martin J, de Bruijn B, Wolting C, Lay V, Tuekam B, Zhang S, Baskin B, Bader GD, Michalickova K *et al*: **PreBIND and Textomy--mining the biomedical literature for protein-protein interactions using a support vector machine**. *BMC Bioinformatics* 2003, **4**(1):11.

99. Zhu G, Spellman PT, Volpe T, Brown PO, Botstein D, Davis TN, Futcher B: **Two yeast forkhead genes regulate the cell cycle and pseudohyphal growth**. *Nature* 2000, **406**(6791):90-94.

100. Yoshimoto H, Saltsman K, Gasch AP, Li HX, Ogawa N, Botstein D, Brown PO, Cyert MS: **Genome-wide analysis of gene expression regulated by the calcineurin/Crz1p signaling pathway in Saccharomyces cerevisiae**. *J Biol Chem* 2002, **277**(34):31079-31088.

101. Gasch AP, Huang M, Metzner S, Botstein D, Elledge SJ, Brown PO: **Genomic expression responses to DNA-damaging agents and the regulatory role of the yeast ATR homolog Mec1p**. *Mol Biol Cell* 2001, **12**(10):2987-3003.

102. Gasch AP, Spellman PT, Kao CM, Carmel-Harel O, Eisen MB, Storz G, Botstein D, Brown PO: **Genomic expression programs in the response of yeast cells to environmental changes**. *Mol Biol Cell* 2000, **11**(12):4241-4257.

103. Spellman PT, Sherlock G, Zhang MQ, Iyer VR, Anders K, Eisen MB, Brown PO, Botstein D, Futcher B: **Comprehensive identification of cell cycle-regulated genes of the yeast Saccharomyces cerevisiae by microarray hybridization**. *Mol Biol Cell* 1998, **9**(12):3273-3297.

104. Chu S, DeRisi J, Eisen M, Mulholland J, Botstein D, Brown PO, Herskowitz I: **The transcriptional program of sporulation in budding yeast**. *Science* 1998, **282**(5389):699-705.

105. DeRisi JL, Iyer VR, Brown PO: **Exploring the metabolic and genetic control of gene expression on a genomic scale**. *Science* 1997, **278**(5338):680-686.

106. Sudarsanam P, Iyer VR, Brown PO, Winston F: **Whole-genome expression analysis of snf/swi mutants of Saccharomyces cerevisiae**. *Proc Natl Acad Sci U S A* 2000, **97**(7):3364-3369.

107. Shakoury-Elizeh M, Tiedeman J, Rashford J, Ferea T, Demeter J, Garcia E, Rolfes R, Brown PO, Botstein D, Philpott CC: **Transcriptional remodeling in response to iron deprivation in Saccharomyces cerevisiae**. *Mol Biol Cell* 2004, **15**(3):1233-1243.

108. Ogawa N, DeRisi J, Brown PO: **New components of a system for phosphate accumulation and polyphosphate metabolism in Saccharomyces cerevisiae revealed by genomic expression analysis**. *Mol Biol Cell* 2000, **11**(12):4309-4321.
