# Supplementary material for: Comprehensive curation and analysis of global interaction networks in Saccharomyces cerevisiae
Source: J Biol. 2006 Jun 8;5(4):11. doi: 10.1186/jbiol36 (PMC1561585; doi:10.1186/jbiol36)
Supplement: Additional data file 3 — Supplementary Figure 1, Curation benchmarks for the LC dataset; Supplementary Figure 2, Distribution of terms in GO categories in LC-PI and LC-GI dataset; Supplementary Figure 3, Relative coverage and overlap of interaction datasets; Supplementary Figure 4, Raw distributions of interactions for each indicated dataset as a function of protein abundance; Supplementary Figure 5, Expression correlation for interaction pairs in LC versus HTP datasets; Supplementary Figure 6, Dense regions in the physical interaction network [file jbiol36-s3.pdf]

Additional data file 3

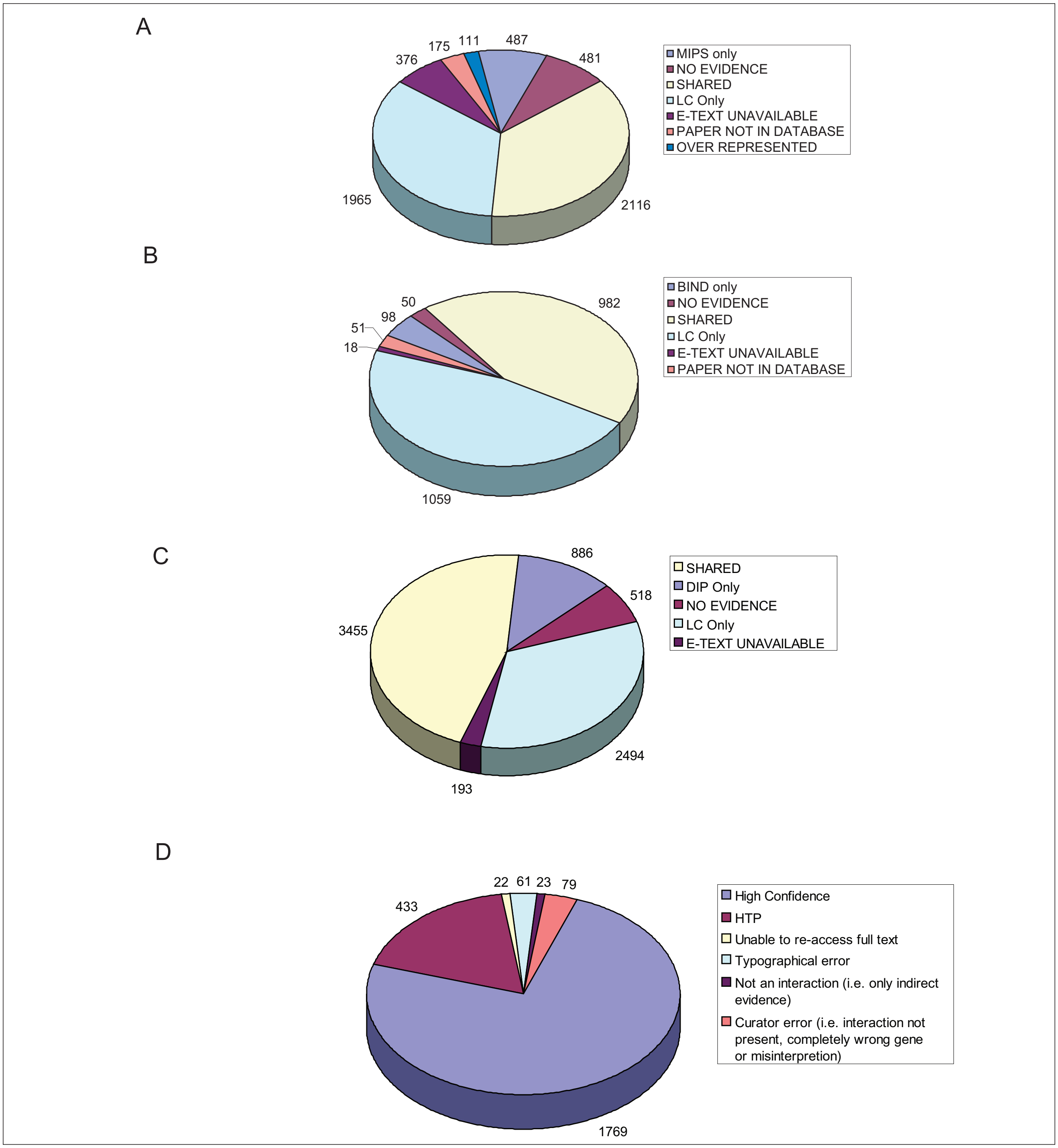

Supplementary Figure 1
Curation benchmarks for the LC dataset. **(a)** Comparison of interactions curated in the LC-PI dataset to those curated in the MIPS dataset for 931 shared publications. Categories of non-overlapping interactions and corresponding number of interactions are indicated. **(b)** Comparison of interactions curated in the LC-PI dataset to those curated in the BIND dataset for 263 shared publications. **(c)** Comparison of interactions curated in the LC-PI dataset to those curated in the DIP dataset for 1,267 shared publications. **(d)** Assessment of interaction confidence and curation error rate by re-examination of 2,387 singly validated interactions in the LC-FYI dataset.

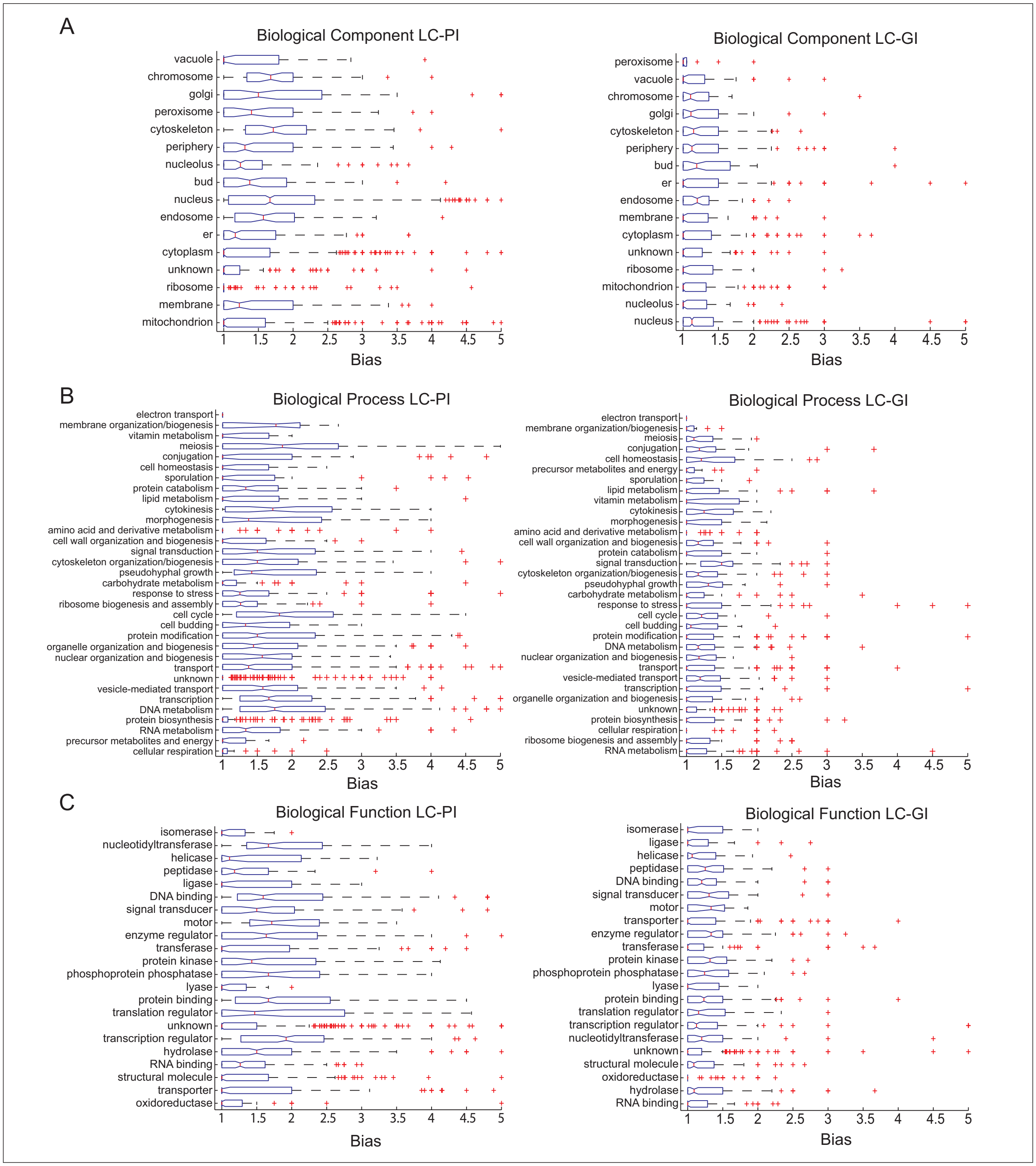

Supplementary Figure 2

Distribution of terms in GO categories in LC-PI and LC-GI dataset. **(a)** Cellular component (localization). **(b)** Biological process. **(c)** Molecular Function. GO terms in each category are as listed. Each bar in the box-plot shows the average bias (normalized by the degree) for each term category. Boxes have lines at the lower quartile, median, and upper quartile values. Outliers are indicated with a red cross.

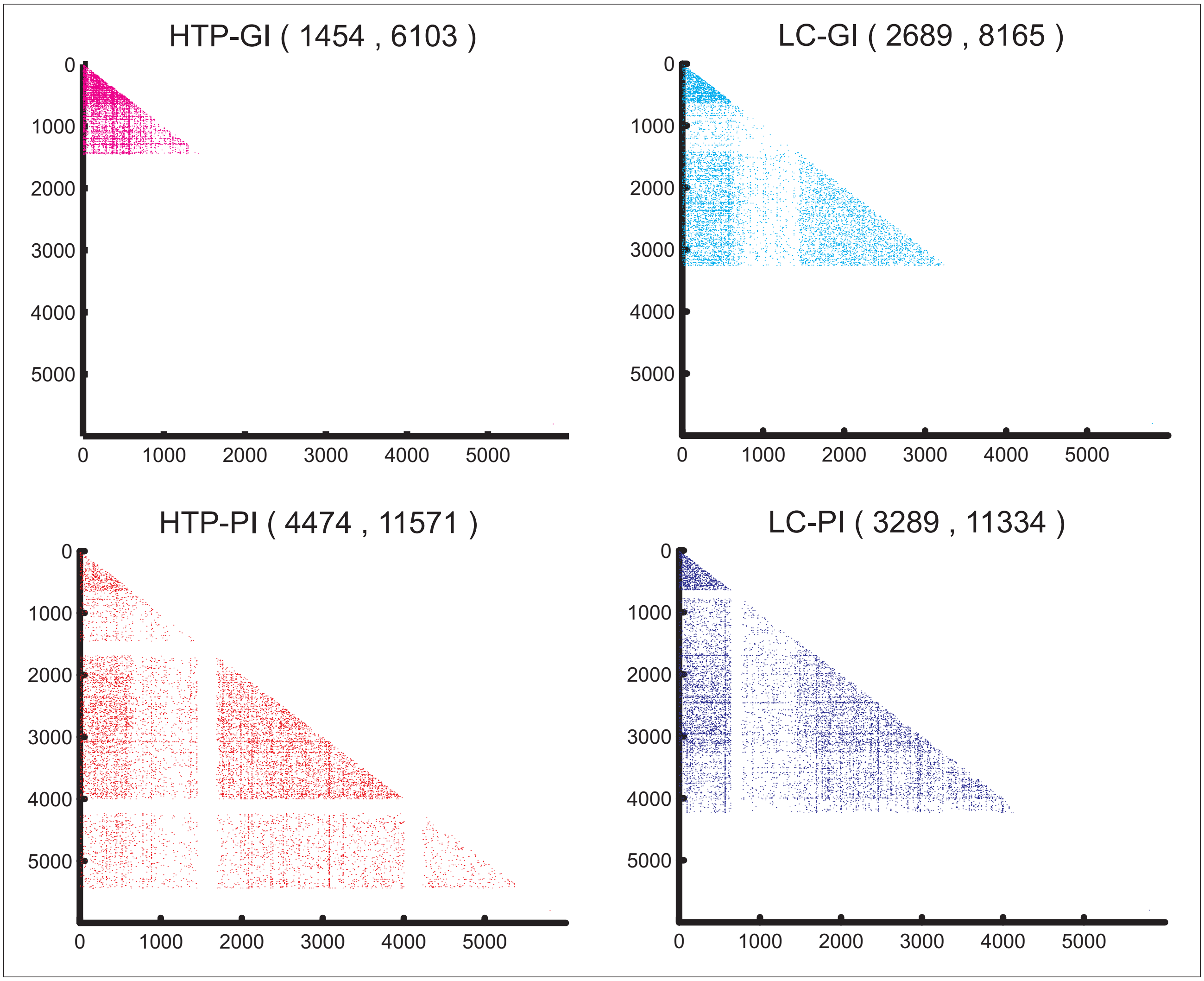

Supplementary Figure 3

Relative coverage and overlap of interaction datasets. Dot matrix representations of all interactions in each of the LC and HTP datasets were created and overlaid on the same ordinates, in order of increasing dataset size (HTP-GI < LC-GI < LC-PI < HTP-PI). Each point corresponds to an interaction pair between two genes/proteins. Blank regions correspond to proteins/genes present in the prior dataset(s) but absent from the visualized dataset. Total nodes and interactions are indicated in parentheses for each dataset.

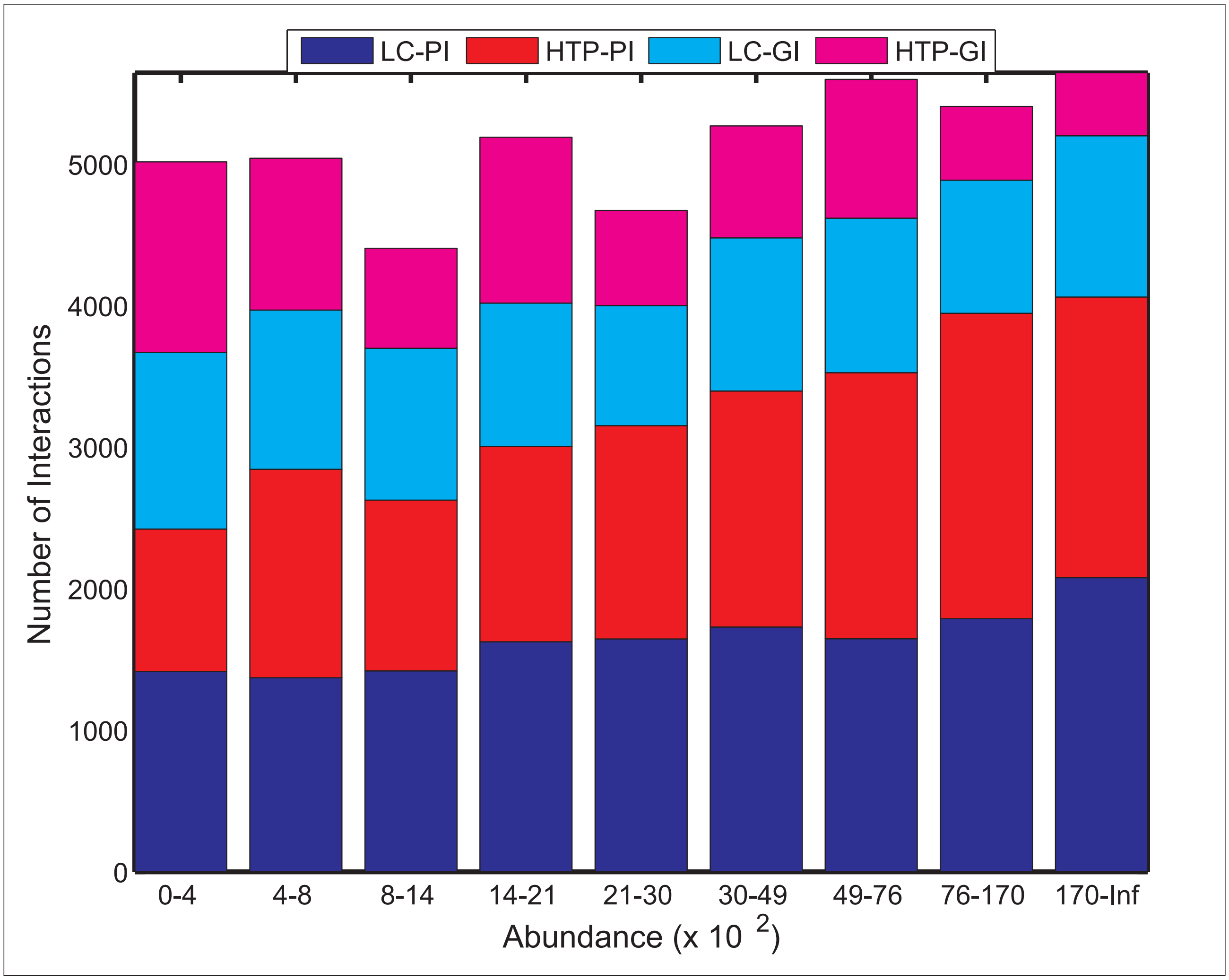

Supplementary Figure 4
Raw distributions of interactions for each indicated dataset as a function of protein abundance. Protein/gene pairs were separated into bins according to protein abundance determined in a large scale study [67]. Abundance is indicated as the estimated number of molecules per cell.

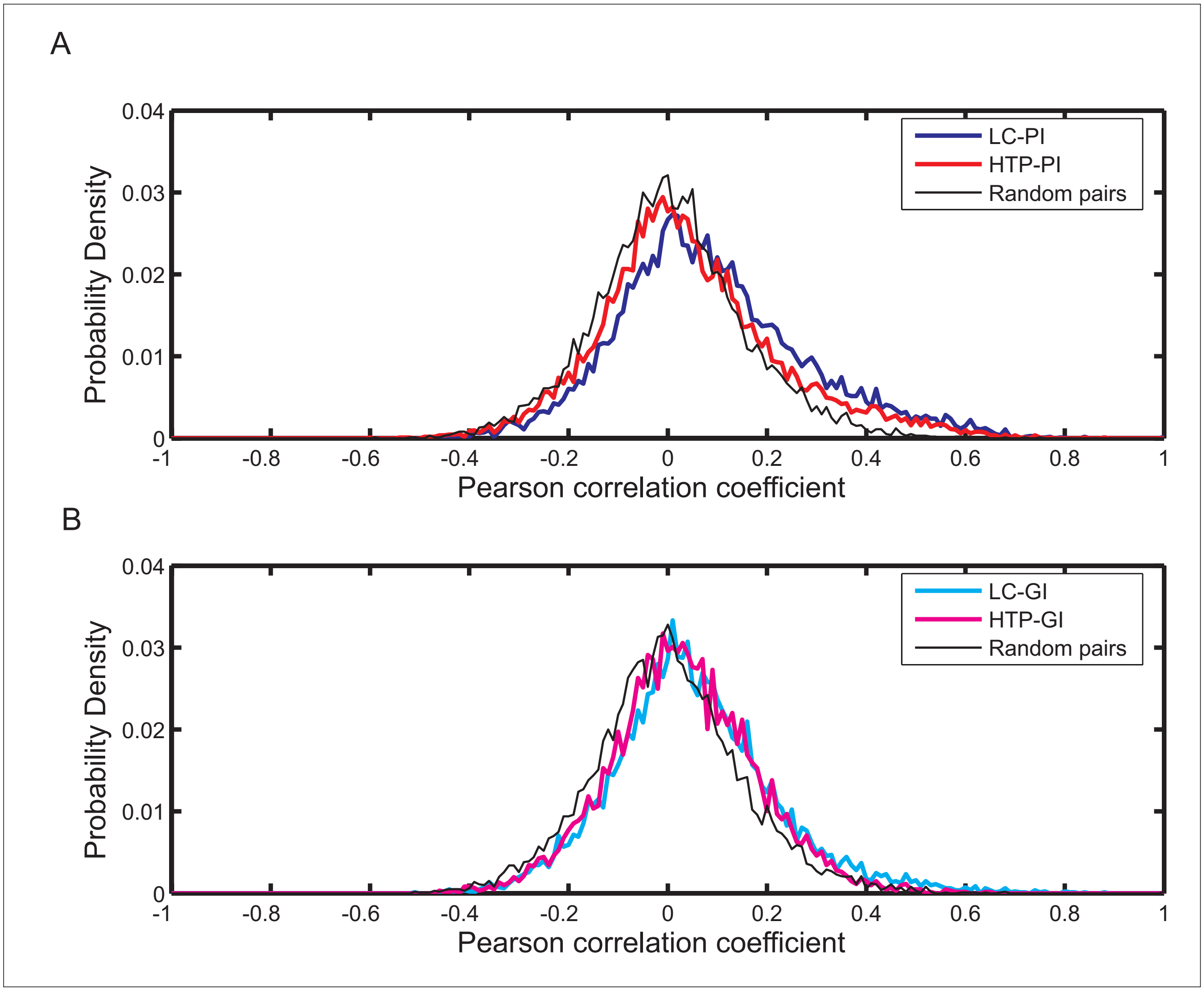

Supplementary Figure 5

Expression correlation for interaction pairs in LC versus HTP datasets. **(a)** LC-PI and HTP-PI datasets. **(b)** LC-GI and HTP-GI datasets. Probability densities of the average Pearson correlation coefficient (PCC) were calculated from a global expression profiling compendium representing over 300 different conditions [71].

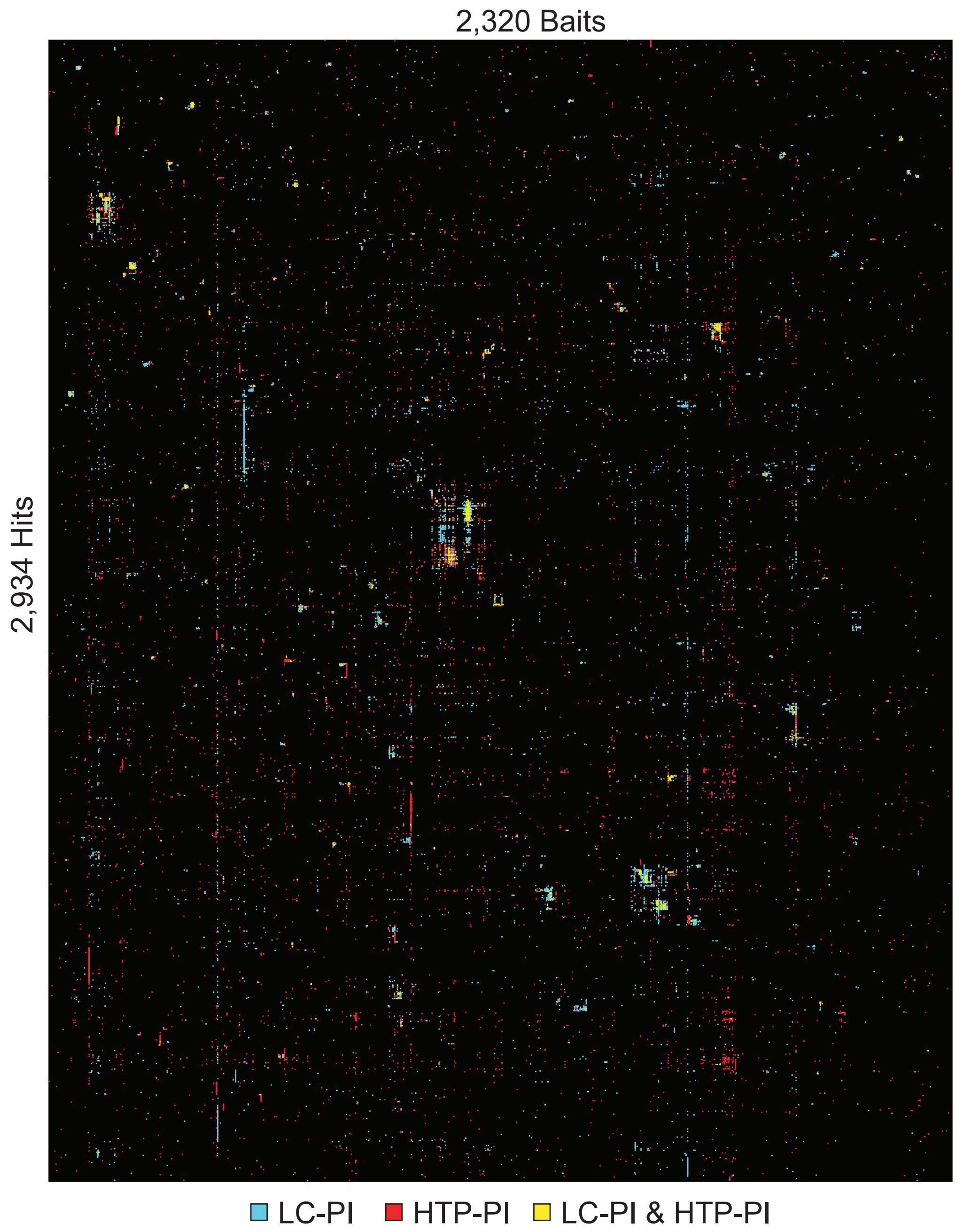

Supplementary Figure 6
Dense regions in the physical interaction network. The combined LC-PI and HTP-PI datasets were hierarchically clustered in two dimensions (corresponding to bait and prey in interaction pair). White pixels represent LC interactions, red pixels represent HTP interactions and yellow pixels represent overlap between LC and HTP interactions. In order to emphasize complexes in the largely empty potential interaction space, pixel size and spread in the clustergram was scaled non-linearly as a function of pixel density. The following GO biological process categories were enriched in the overlap ($P < 0.001$): DNA metabolism, RNA metabolism, cell cycle, cytoskeleton organization and biogenesis, nuclear organization and biogenesis, ribosome biogenesis and assembly, and transcription. The following GO function categories were enriched in the overlap ($P < 0.001$): DNA binding, RNA binding, enzyme regulator, nucleotidyltransferase, phosphoprotein phosphatase, protein binding, protein kinase and transcription regulator.
