# Supplementary material for: Comprehensive curation and analysis of global interaction networks in Saccharomyces cerevisiae
Source: J Biol. 2006 Jun 8;5(4):11. doi: 10.1186/jbiol36 (PMC1561585; doi:10.1186/jbiol36)
Supplement: Additional data file 4 — Flat files of the main datasets [file jbiol36-s4.zip › Dataset Notes.doc]

***Saccharomyces cerevisiae* Curation Datasets**

1.) **HTP_BIOCHEMICAL_011906.txt**

Contains the interactions from the following 5 HTP papers; 11805826, 11805837, 11283351, 10655498, 10688190

2.) **LC_BIOCHEMICAL_011906_MinusRNA.txt**

Contains the Literature Curated biochemical interactions minus a few RNA genes, see categories below

3.) **SGA_GENETIC_011906.txt**

Only contains Literature Curated genetic interactions resulting from a SGA screen (mainly synthetic lethal interactions but occasionally other types)

4.) **LC_NON_SGA_GENETIC_011906.txt**

Everything in the Literature Curated genetic set excluding the interactions resulting from a SGA screen

**NOTES:**

a.) All the datasets are formatted as tab delimited text files with the following columns:

| Bait gene/protein | Hit gene/protein | Bait Standard Name | Hit Standard Name | Experimental System | Source | PubMed ID |
| --- | --- | --- | --- | --- | --- | --- |

i.) Bait gene/protein
ii.) Hit gene/protein
iii.) Bait standard name
iv.) Hit standard name
v.) Experimental System	(See categories below)
vi.) Source	(Author)
vii.) PubMed ID	(PubMed ID)

b.) All Co-purification complexes are strictly represented in the datasets as a minimal spoke model. Because bait assignment for Co-purification complexes is arbitrary (i.e. there is no bait per se), the hub of each spoke was defined by the most highly connected protein within each complex defined by the LC_BIOCHEMICAL set minus the Co-purification information if no information was found in the LC_BIOCHEMICAL set then the HTP_BIOCHEMICAL set was used.

**BIOCHEMICAL systems include:**

Affinity Capture-MS

Affinity Capture-Western

Two-hybrid

Co-localization

FRET

Affinity Capture-RNA

Reconstituted Complex

Protein-peptide

Co-purification

Co-fractionation

Biochemical Activity

Co-crystal Structure

Far Western

Protein-RNA

**GENETIC systems include:**

Synthetic Lethality

Synthetic Growth Defect

Synthetic Rescue

Dosage Lethality

Dosage Growth Defect

Dosage Rescue

Phenotypic Enhancement

Phenotypic Suppression
